# A Combined Systematic-Stochastic Algorithm for the Conformational Search in Flexible Acyclic Molecules

**DOI:** 10.3389/fchem.2020.00016

**Published:** 2020-01-28

**Authors:** David Ferro-Costas, Antonio Fernández-Ramos

**Affiliations:** Center for Research in Biological Chemistry and Molecular Materials (CIQUS), Universidade de Santiago de Compostela, Santiago de Compostela, Spain

**Keywords:** conformational search, hindered rotors, torsional anharmonicity, stochastic methods, geometrical optimization

## Abstract

We propose an algorithm that is a combination of systematic variation of the torsions and Monte Carlo (or stochastic) search. It starts with a trial geometry in internal coordinates and with a set of preconditioned torsional angles, i.e., torsional angles at which minima are expected according to the chemical knowledge. Firstly, the optimization of those preconditioned geometries is carried out at a low electronic structure level, generating an initial set of conformers. Secondly, random points in the torsional space are generated outside the “area of influence” of the previously optimized minima (i.e., outside a hypercube about each minima). These random points are used to build the trial structure, which is optimized by an electronic structure software. The optimized structure may correspond to a new conformer (which would be stored) or to an already existing one. Initial torsional angles (and also final ones if a new conformer is found) are stored to prevent visiting the same region of the torsional space twice. The stochastic search can be repeated as many times as desired. Finally, the low-level geometries are recovered and used as the starting point for the high-level optimizations. The algorithm has been employed in the calculation of multi-structural quasi harmonic and multi-structural torsional anharmonic partition functions for a series of alcohols ranging from n-propanol to n-heptanol. It was also tested for the amino acid L-serine.

## 1. Introduction

Flexible molecules have many conformational minima which can be easily reached by torsional motions of the molecular framework in the potential energy surface (PES). For the last few years, there are methods, as the multi-structural harmonic-oscillator (MS-HO) approximation (Zheng et al., 2011a), which take into account the characteristics of all these equilibrium structures. Specifically, the MS-HO method incorporates the rotational and vibrational (rovibrational) partition function of each of the conformers within the rigid-rotor harmonic-oscillator approximation. This is a substantial improvement over the one-well harmonic oscillator (1W-HO) approximation in which the structure of the absolute minimum is the only one to be considered (Ferro-Costas et al., 2018b).

Locating all conformers is just the first step toward the evaluation of more accurate rovibrational partition functions. For instance, it has been shown that MS-HO partition functions improve over 1W-HO ones (Ferro-Costas et al., 2018b), additionally torsional anharmonicity should be also included (Yu et al., 2011; Zheng et al., 2011b; Zheng and Truhlar, 2013) to increase the accuracy of the results. The most reliable methods that incorporate torsional anharmonicity can only be applied to a reduced number of torsional degrees of freedom (Fernández-Ramos, 2013) and they require more information of the PES than just the minima. For instance, the extended two-dimensional torsional method (E2DT) (Simón-Carballido et al., 2017), implemented in the Q2DTor program (Ferro-Costas et al., 2018a), needs a fine grid of points for the construction of the torsional PES. The procedure also includes the location of all stationary points (i.e., minima, saddle points and maxima in the 2D-PES).

Therefore, the amount of information needed from the PES depends on the method, and it is crucial to devise algorithms that allow an efficient construction of such PES. For example, when the number of torsional degrees of freedom is only 2, so the E2DT method can be applied, geometry scans at a regular number of points along the PES can be carried out. These scans involve partial optimizations in which all degrees of freedom are optimized except the two torsional modes. When the torsional global PES is calculated by systematic mapping, if possible, it is essential to reduce the number of points to be calculated. This reduction depends on molecular geometry aspects as conformational enantiomerism, internal symmetry of the rotors and molecular symmetry. The rules to replicate points of a PES under some symmetry conditions are given in Ferro-Costas et al. (2018a). As the number of torsional degrees of freedom increases, the amount of information needed from the PES should be reduced in order to keep the problem tractable. For those cases, the multi-structural torsional method is a good choice (Zheng et al., 2012, 2013), because the model is built assuming that the only information at hand is the set of conformational minima.

This work is concerned with the search of conformational structures in the torsional PES of flexible acyclic molecules with more than 2 torsions (typically up to 10). Having the equilibrium geometries, it is possible to calculate accurate rovibrational partition functions in a wide range of temperatures. In this sense, the algorithm is not limited to the search of the most stable equilibrium structures, O'Boyle et al. (2011) which are the only ones that are relevant at low temperatures. It seeks for *all* conformers, because they are required for the calculation of partition functions at high temperatures and for the evaluation of torsional anharmonicity. Unfortunately, this algorithm cannot deal with large biological systems or with conformations originated from ring puckering (Kolossváry and Guida, 1996; Watts et al., 2010). For that purpose there is an extense list of algorithms and programs (see Loferer et al., 2007; Friedrich et al., 2019 and references therein).

The algorithm here presented is a combination of a systematic method that locates intuitively expected conformers plus a Monte Carlo method that finds unanticipated ones. A detailed description of the algorithm is given in the following section. The series of alcohols ranging from n-propanol to n-heptanol and the amino acid L-serine have been selected to test the algorithm.

## 2. Description of The Algorithm

The target systems for this algorithm are flexible acyclic molecules characterized by *t* dihedral angles. Internal rotations about these dihedrals guide the system toward different conformations; each of them being represented by a *t*-dimensional point **Φ** = (ϕ_1_, ⋯, ϕ_τ_, ⋯, ϕ_*t*_), where the τ-th dihedral angle runs from 1 to *t*.

The various geometries involved in the algorithm are:

- **Φ**^R^: the reference geometry, i.e., the initial geometry provided by the user.
- $\Phi^{{\text{G}}_{1}}$: a guess geometry during the systematic search. The total number of structures generated is *K*_1_ of which $K_{1}^{⋆}$ are the ones that pass the tests (see section 2.1) and turn into trial geometries. Notice that $K_{1}^{⋆}\leq K_{1}$.
- $\Phi^{{\text{G}}_{2}}$: a guess geometry during the stochastic search. The total number of geometries generated is *K*_2_ of which $K_{2}^{⋆}$ are the ones that pass the tests (see section 2.1) and turn into trial geometries. Notice that $K_{2}^{⋆}\leq K_{2}$.
- $\Phi_{k^{⋆}}^{0}$: the *k*^⋆^-th trial geometry, $k^{⋆}=1,\ldots ,K^{⋆};K^{⋆}=K_{1}^{⋆}+K_{2}^{⋆}$. The pool of trial geometries is represented by $\left\{\Phi_{k^{⋆}}^{0}\right\}$.
- **Φ**^⋆^: a trial geometry to be optimized.
- **Φ**^⋆,opt^: a trial geometry **Φ**^⋆^ after optimization.
- $\Phi_{j}^{\text{eq}}$: : the *j*-th equilibrium conformer, *j* = 1, …, *J*. The pool of such conformers is represented by $\left\{\Phi_{j}^{\text{eq}}\right\}$.
- $\Phi_{p}^{\text{st}}$: the *p*-th stored point, $p=1,\ldots ,P;P=K_{1}^{⋆}+K_{2}^{⋆}+J$. The pool of stored points is the union of the previous two sets, $\left\{\Phi_{p}^{\text{st}}\right\}=\left\{\Phi_{j}^{\text{eq}}\right\}\cup \left\{\Phi_{k^{⋆}}^{0}\right\}$.

**Figure 1:**
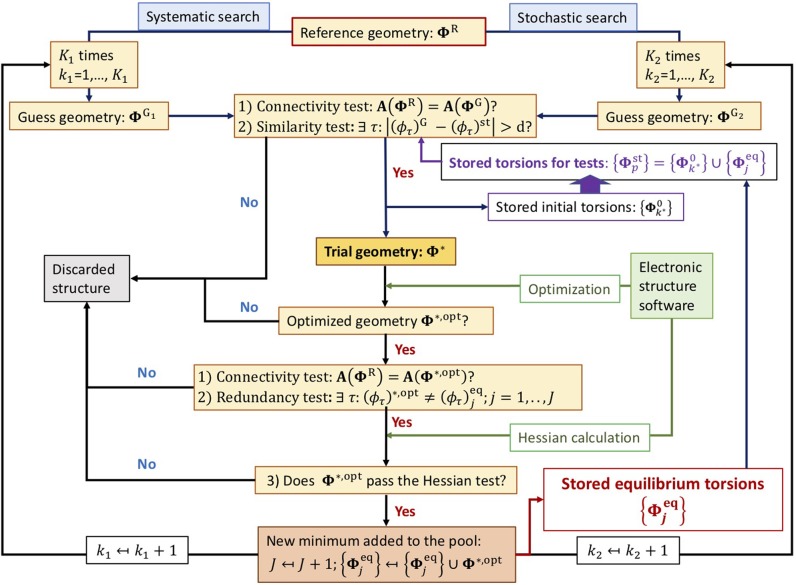
Flux diagram of the conformer search algorithm.

The setup of the algorithm is schematically shown in the flux diagram of Figure 1. It starts with a reference geometry given in the Z-matrix format where the *t* target torsions must be defined unambiguously. Only in this manner, it is possible to define the **Φ**^R^ torsional point univocally. Otherwise, the torsional analysis cannot be carried out. The algorithm consists of two well differentiated searching methods: systematic and stochastic.

### 2.1. The Tests

A guess structure, either $\Phi^{{\text{G}}_{1}}$ or $\Phi^{{\text{G}}_{2}}$, in general denoted as **Φ**^G^, must complete two tests before being considered a trial geometry **Φ**^⋆^:

1. *The connectivity test*: excludes structures having unphysical bond lengths (for instance, structures with superimposed atoms) or structures with different connectivity than the reference geometry. The detection and exclusion of these type of structures is carried out through the adjacency (or connectivity) matrix of the guess geometry, **A**(**Φ**^G^), which is compared to that of the reference structure, **A**(**Φ**^R^). Only if these two matrices are equal:
  (1) $$\begin{array}{l} \text{A}\left(\Phi^{\text{R}}\right)=\text{A}\left(\Phi^{\text{G}}\right) \end{array}$$
  the guess geometry passes the test. We highlight the importance of generating an adequate reference structure, as its connectivity matrix is used to accept or discard guess geometries.
2. *The similarity test*: performs a comparison between the guess point and the pool of the *P* stored points. If **Φ**^G^ falls outside of all the hypercubes generated about each stored point, the geometry is accepted. For hypercubes with edge size of 2*d*, this test is positive if:

(2) $$\begin{array}{l} \exists \;\tau :|{\left(\phi_{\tau }\right)}^{\text{G}}-{\left(\phi_{\tau }\right)}_{p}^{\text{st}}|>d,\;p=1,\cdots \;,P \end{array}$$

An optimized trial geometry, **Φ**^⋆,opt^, must complete three tests.

1. *The connectivity test*: assures that the optimized and reference geometries share the same connectivity.
2. *The redundancy test*: compares the current geometry with the pool of the *J* optimized equilibrium torsions. If
  (3) $$\begin{array}{l} \exists \;\tau :{\left(\phi_{\tau }\right)}^{⋆,\text{opt}}\neq {\left(\phi_{\tau }\right)}_{j}^{\text{eq}},\;j=1,\cdots \;,J \end{array}$$
  then **Φ**^⋆,opt^ is a candidate for being a new equilibrium structure in the torsional PES.
3. *The Hessian test*: assures that the optimized geometry is a new minimum. The electronic structure software is used to calculate the Hessian matrix; if the normal-mode frequencies of the diagonalized matrix are all real, the optimized structure is a minimum.

Notice that the order in which the tests are carried out is important because if the optimized structure fails to pass the first two tests, no time is lost in the evaluation of the Hessian matrix.

### 2.2. Systematic Search

The first part of the algorithm consists of a systematic search that makes use of a pool of *K*_1_ initial structures, each of them characterized by a set of torsional angles $\Phi^{{\text{G}}_{1}}$ which have their origin on basic molecular structure analysis. If there are *P*_τ_ initial chemical-intuitive guesses for a given torsion τ, the total number of preconditioned guesses is:

(4) $$\begin{array}{l} K_{1}=\overset{t}{\underset{\tau =1}{\prod }}P_{\tau } \end{array}$$

For instance, for a four *sp*^3^ carbon linear chain, the expected location of the minima is at dihedral angles of 180° and ±60°, which correspond to the *anti* (antiperiplanar ot T) and *gauche* (synclinal or G±) positions, and therefore *P*_τ_ = 3 for the torsion. Notice that only dihedral angles that generate new distinguishable structures should be included. In this context, methyl groups should be ignored because its internal rotation only generates indistinguishable structures. For instance, in the case of n-butanol we only need to consider three torsions (*t* = 3), each of them with three intuitive positions (T, G+, and G−), that is *P*_1_ = *P*_2_ = *P*_3_ = 3. Therefore, the number of geometries to be generated within the pool is *K*_1_ = 27.

During the generation of the initial structures, the algorithm should take into account the characteristics of the molecular geometry, as for instance, molecular symmetry. Returning to the previous example, the molecule of n-butanol has one structure given by the dihedrals (TTT) which has a plane of symmetry and, therefore, it belongs to the *C*_s_ point group symmetry. As a consequence, all structures, with exception of (TTT), have conformational enantiomers, that is, distinguishable optical isomers with the same electronic structure properties. Therefore, it is sufficient to locate one of the two isomers. The conformational enantiomer of the **Φ** structure is the −**Φ** structure (i.e., the value of the dihedral angle for each torsion is set to 360° - ϕτ). For instance, structure (TG+G-) has structure (TG-G+) as enantiomer. Consequently, only 14 of the 27 initial structures need to be tried. In general, for a molecule with a plane of symmetry, the initial number of structures of the preconditioned systematic search is reduced to (*K*_1_ + 1)/2. The rest of the structures are automatically generated from the calculated ones.

Each of the *K*_1_ structures leads to a guess point, $\Phi^{{\text{G}}_{1}}$, which is the current candidate to turn into a new minimum in the PES upon geometry optimization. If this point fails to pass the *connectivity* or the *similarity* tests, it is discarded. Otherwise, $\Phi^{{\text{G}}_{1}}$ is a suitable structure to be optimized by the electronic structure software:

(5) $$\begin{array}{l} \Phi^{⋆}\leftarrow \Phi^{{\text{G}}_{1}} \end{array}$$

and the $\left\{\Phi_{k^{⋆}}^{0}\right\}$ pool is updated:

(6) $$\begin{array}{l} \left\{\Phi_{k^{⋆}}^{0}\right\}\leftarrow \left\{\Phi_{k^{⋆}}^{0}\right\}\cup \left\{\Phi^{⋆}\right\} \end{array}$$

During the systematic search the *similarity test* only involves $\left\{\Phi_{j}^{\text{eq}}\right\}$ and not $\left\{\Phi_{p}^{\text{st}}\right\}$. The reason is that the hypercubes generated from these structures do not overlap.

If **Φ**^⋆^ successfully converges to an equilibrium structure, **Φ**^⋆,opt^, and passes all the required tests, then the resulting geometry is added to the list of conformers and the pool of equilibrium torsions is updated:

(7) $$\begin{array}{l} \left\{\Phi_{j}^{\text{eq}}\right\}\leftarrow \left\{\Phi_{j}^{\text{eq}}\right\}\cup \left\{\Phi^{⋆,\text{opt}}\right\} \end{array}$$

### 2.3. Stochastic Search

The algorithm can perform a series of *K*_2_ cycles performing a Monte Carlo search after the systematic procedure. At every cycle, the algorithm generates *t* random numbers, which are the components of the torsional point $\Phi^{{\text{G}}_{2}}$. Once they are generated, the procedure follows the same pattern as the systematic search. In this case, the *similarity test* accesses to the $\left\{\Phi_{p}^{\text{st}}\right\}$ pool, not just to the equilibrium structures, because it cannot be assured that the new point falls outside of the “area of influence” of previous trial geometries.

We highlight that it is possible to run several batches of the Monte Carlo search, each of them with different specifications for the hypercube edge size (2*d*).

### 2.4. Electronic Structure Calculations

The algorithm assumes that the initial set of conformers will be obtained at a low electronic structure level (LL). Those equilibrium geometries can be used as the starting point of high-level (HL) electronic structure calculations. Therefore, the LL calculations should produce a torsional PES which, at least qualitatively, has a similar topology than the HL torsional PES. For molecules of the size presented in this work, Hartree-Fock (HF) calculations are affordable as the LL. Other reason to choose HF as the LL is that they tend to overestimate torsional barriers, as well as to produce more minima than electronic correlated methods. However, we notice that the LL search is not restricted to HF. Molecular mechanics, semiempirical methods or other *ab initio* methods could be used as LL, depending on the molecular system and on the available computational resources. In principle, the algorithm was designed for locating minima in the torsional PES of flexible acyclic systems with up to 10 torsions. Bigger systems would require substantial computational cost. Two straightforward ways of reducing computer time are the parallelization of the algorithm and/or the use of inexpensive LL methods.

For the n-alcohols series and L-serine, HF/3-21G was chosen as the LL method. For n-alcohols the set of the $\left\{\Phi_{j}^{\text{eq}}\right\}$ conformers was re-optimized employing the MPWB1K functional (Zhao et al., 2004) in combination with the 6-31+G(d,p) basis set (Hehre et al., 1972). In the case of serine, B3LYP/6-31++G^**^ was the method of choice with the objective of establishing a direct comparison with previous calculations (Najbauer et al., 2015). Geometry optimizations and frequency calculations were carried out with the *Gaussian 09* package (Frisch et al., 2004).

## 3. Multi-Structural Partition Functions

It has been shown that for flexible molecules the incorporation of multiple conformers may have a substantial impact in the magnitude of the partition functions, thermochemical properties and thermal rate constants. The algorithm has been designed to obtain *all* the torsional PES minima of the molecule. After this information is accessible, it is possible to calculate multi-structural (MS) partition functions, i.e., partition functions that include multiple torsional conformers. Here we are concerned with the calculation of the MS partition functions using the following approximations: the harmonic-oscillator (MS-HO), the quasi-harmonic (MS-QH), and the coupled torsional anharmonic (MS-T(C); Zheng and Truhlar, 2013). Any of these methods, named in increasing order of accuracy, provides more reliable values of thermochemical properties than methods based on just one well. In the MS-HO approximation the rovibrational partition function is given by

(8) $$\begin{array}{l} Q^{\text{MS}-\text{HO}}=\overset{J_{c}}{\underset{j_{c}}{\sum }}Q_{j_{c}}^{\text{rot}}Q_{j_{c}}^{\text{HO}}e^{-\beta U_{j_{c}}} \end{array}$$

where *J*_*c*_ is the total number of conformers and *U*_*j*_*c*__ is the relative energy of conformer *j*_*c*_ relative to the global minimum. Without lost of generality, it is possible to sum just over *all* the distinguishable structures *J* that are not conformational enantiomers

(9) $$\begin{array}{l} Q^{\text{MS}-\text{HO}}=\overset{J}{\underset{j}{\sum }}w_{j}Q_{j}^{\text{rot}}Q_{j}^{\text{HO}}e^{-\beta U_{j}} \end{array}$$

where *w*_*j*_ = 1 if the *j* structure is unique and *w*_*j*_ = 2 if it has a conformational enantiomer. The rigid rotor rotational partition function $Q_{j}^{\text{rot}}$ is given by

(10) $$\begin{array}{l} Q_{j}^{\text{rot}}=\frac{8\pi^{2}}{\sigma_{\text{rot},j}}{\left(\frac{1}{2\pi \hbar^{2}\beta }\right)}^{3/2}\sqrt{I_{1,j}^{\text{rot}}I_{2,j}^{\text{rot}}I_{3,j}^{\text{rot}}} \end{array}$$

where ℏ is the Planck's constant divided by 2π, and $\beta ={\left(k_{\text{B}}T\right)}^{-1}$, with *k*_B_ being the Boltmann's constant and *T* the temperature; σ_rot,*j*_ is the symmetry number of rotation (Fernández-Ramos et al., 2007) and $I_{i,j}^{\text{rot}}$ (*i* = 1, 2 or 3) is the *i*-th principal moment of inertia of conformer *j*.

The harmonic oscillator partition $Q_{j}^{\text{HO}}$ is

(11) $$\begin{array}{l} Q_{j}^{\text{HO}}={\tilde{Q}}_{j}^{\text{HO}}e^{-\beta E_{j}^{\text{HO}}} \end{array}$$

where

(12) $$\begin{array}{l} {\tilde{Q}}_{j}^{\text{HO}}=\overset{3N-6}{\underset{m=1}{\prod }}\frac{1}{1-e^{-\beta \hbar \omega_{m,j}}} \end{array}$$

is the HO vibrational partition function calculated by taking the zero-point energy (ZPE) as the reference energy, which is given by

(13) $$\begin{array}{l} E_{j}^{\text{HO}}=\overset{3N-6}{\underset{m=1}{\sum }}\frac{1}{2}\hbar \omega_{m,j} \end{array}$$

where *N* is the number of atoms, and ω_*m, j*_ is the HO frequency of the *m*-th normal mode in the *j*-th conformer. A variant of the MS-HO partition function is the MS-QH one, in which the harmonic frequencies are multiplied by a scale parameter λ^ZPE^ which is dependent on the electronic structure method and that it was previously parametrized to reproduce experimental ZPEs. Thus,

(14) $$\begin{array}{l} Q_{j}^{\text{QH}}={\tilde{Q}}_{j}^{\text{QH}}e^{-\beta E_{j}^{\text{QH}}} \end{array}$$

(15) $$\begin{array}{l} {\tilde{Q}}_{j}^{\text{QH}}=\overset{3N-6}{\underset{m=1}{\prod }}\frac{1}{1-e^{-\beta \hbar \lambda^{\text{ZPE}}\omega_{m,j}}} \end{array}$$

(16) $$\begin{array}{l} E_{j}^{\text{QH}}=\lambda^{\text{ZPE}}\overset{3N-6}{\underset{m=1}{\sum }}\frac{1}{2}\hbar \omega_{m,j} \end{array}$$

and therefore

(17) $$\begin{array}{l} Q^{\text{MS}-\text{QH}}=\overset{J}{\underset{j}{\sum }}w_{j}Q_{j}^{\text{rot}}Q_{j}^{\text{QH}}e^{-\beta U_{j}} \end{array}$$

The MS-T(C) rovibrational partition function includes torsional anharmonicity on the HO or QH partition functions through a multiplicative factor $F_{\text{cl},j}^{\text{MS}-\text{T}\left(\text{C}\right)}$. For the QH case, it is given by

(18) $$\begin{array}{l} Q^{\text{MS}-\text{T}\left(\text{C}\right)}=\overset{J}{\underset{j=1}{\sum }}Q_{j}^{\text{rot}}Q_{j}^{\text{QH}}F_{\text{cl},j}^{\text{MS}-\text{T}\left(\text{C}\right)} \end{array}$$

where

(19) $$\begin{array}{l} F_{\text{cl},j}^{\text{MS}-\text{T}\left(\text{C}\right)}=\overset{t}{\underset{\eta =1}{\prod }}f_{j,\eta }=\overset{t}{\underset{\eta =1}{\prod }}\frac{q_{j,\eta }^{\text{RC}\left(\text{C}\right)}}{q_{j,\eta }^{\text{CHO}\left(\text{C}\right)}} \end{array}$$

The *f*_*j*,η_ factors are expressed as the ratio between the classical reference anharmonic (RC) and classical harmonic oscillator (CHO) torsional partition functions. Although the reference classical partition function involves some approximations, it incorporates couplings in the kinetic and potential energies between the torsions. Therefore, in flexible systems with multiple torsional modes, the MS-T(C) entails a substantial improvement over the MS-QH method.

## 4. Results and Discussion

The automatic protocol presented in this work was adopted to study the n-alcohols from 3 (n-propanol) to 7 (n-heptanol) carbon atoms. For the calculation of the partition functions, the frequencies were scaled by the recommended factor λ^ZPE^ = 0.951 (Alecu et al., 2010). With the exception of n-propanol and n-butanol, the number of previous studies on the conformations of n-alcohols is scarce. Thus, additionally to the seek for conformational minima of n-alcohols, we have benchmarked our algorithm against a previous study on the conformations of the amino acid L-serine, a molecule presenting several functional groups (section 4.2). However, we will center our attention on the n-alcohols when discussing about the efficiency of the algorithm.

### 4.1. Efficiency of the Algorithm

**Figure 2:**
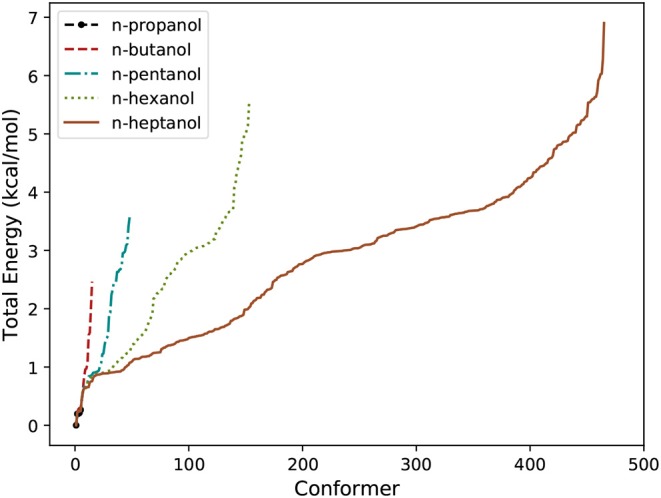
Number of conformers of n-alcohols and their relative total energy.

A summary about the efficiency of the algorithm for n-alcohols is provided in Table 1. In it, *J*_1_ and *J*_2_ represent the number of conformers found in the systematic and in the Monte-Carlo searchings, respectively. Similarly, *J*_LL_ and *J*_HL_ are the total number of conformers found at LL and HL, respectively. We highlight that the number of conformers shown in Table 1 excludes enantiomeric structures. Consequently, the total number of conformers is given by 2*J*_HL_ − 1. The relative total energy of the HL conformers is represented in Figure 2 for the five n-alcohols. We refer to the Supplementary Material for a list containing the electronic energy and Cartesian coordinates of all the HL conformers.

**Table 1:** Number of conformers obtained for the n-alcohol series that illustrate the efficiency of the algorithm.

**Alcohol**	***t***	***K*_**1**_**	**$K_{1}^{⋆}$**	***J*_**1**_**	***K*_**2**_**	**$K_{2}^{⋆}$**	***J*_**2**_**	***J*_**LL**_**	***J*_**HL**_**
n-propanol	2	5	5	5	200	80	0	5	5
n-butanol	3	14	14	14	200	167	1	15	15
n-pentanol	4	41	39	38	400	377	15	53	48
n-hexanol	5	122	110	106	4,500	3,988	59	165	153
n-heptanol	6	365	307	297	5,500	4,664	192	489	465

*See text for details about nomenclature*.

For the case of n-heptanol, according to the chemical intuition and excluding enantiomers, a total of (3^6^ + 1)/2 = 365 conformers are expected. The algorithm discarded 58 of them as a result of very strained geometries which did not pass the *connectivity test*. Therefore, geometric optimizations were performed on 84% of the initial geometries of which 97% of them led to a new conformer. From these results, we can state that the systematic search is very efficient because almost every geometry optimization that was carried out translated into a new conformer. This result is not surprising as the starting geometries of the systematic search arise from well-established chemical knowledge.

The performance of the stochastic search is more difficult to estimate. About 15% of the generated geometries were immediately discarded through the *connectivity test* saving a considerable amount of computational time. Notice that “all” minima with torsional angles lying close to the preconditioned values have already been found, so only about 4% of the geometry optimizations led to new conformers. However, this result should not be taken as poor performance of the algorithm, but as an inherent difficulty associated with the search of new conformers in partially explored PESs. Every new batch of calculations produces less new minima, and the algorithm is stopped when no new conformers are found. However, even in this situation, the location of all the conformers is not guaranteed.

Regarding to the HL optimizations from the LL geometries, we observe that the procedure is quite effective: for the five n-alcohols, more than 90% of the LL geometries leaded to a new HL conformer.

### 4.2. Benchmarking

Studies regarding to the conformational flexibility of n-alcohols beyond n-butanol are scarce in the literature. Even for n-butanol, some of these previous studies (see Black and Simmie, 2010) pointed toward the existence of 14 conformers (27 considering enantiomers), which are the number of conformers encountered after a systematic search. However, the total number of conformers is 15. This last conformer appears after a stochastic search.

Chen at al. (2015) claimed that n-pentanol has 41 minima, which are the hypothetical number of conformers generated by T, G+ and G- configurations for each of the torsions. Our algorithm, discarded two of them in the systematic search and encountered 38 conformers at HL. The stochastic search located another 10 conformers to reach a total of 48 conformers. The algorithm may discard some initial geometries if they do not pass the connectivity test; however, if there are minima close to these strained geometries, they will be encountered during the Monte Carlo search.

For n-hexanol there is a very recent work by Vaskivskyi et al. (2019), who made a systematic search starting from 122 structures and found 111 different conformers. Our algorithm located a total of 153 minima at the HL, 106 in the systematic search and 47 in the Monte Carlo search.

To the best of our knowledge this is the first work dealing with the conformational flexibility of n-heptanol.

Najbauer et al. (2015) reported the 14 conformers of L-serine with the lowest Gibbs free energies at 0 K, $\Delta G_{0\text{K}}^{\text{o}}$, calculated at the B3LYP/6-31++G^**^ level. Our algorithm found a total of 72 LL conformers, number that was reduced to 60 (listed in the Supplementary Material) after the HL reoptimizations, also at the B3LYP/6-31++G^**^ level. Of the total number of conformers obtained at the HL, 32 of them are within the range of free energies reported by Najbauer et al. (2015) (see Table 2). Specifically, Najbauer *et al* missed 18 conformers within a free energy window of 4 kcal/mol.

**Table 2:** L-serine low-energy conformers sorted out according to their relative harmonic Gibbs free energies at 0 K (in kcal/mol) calculated at the B3LYP/6-31++G^**^ level.

**This work**	**(ϕ_1_, ϕ_2_, ϕ_3_, ϕ_4_, ϕ_5_)**	**$\Delta G_{0\;\text{K}}^{\text{o}}$**	**Najbauer et al**.
1	(178, 289, 181, 044, 278)	0.00	1
2	(355, 145, 293, 082, 092)	0.17	2
3	(356, 145, 064, 305, 092)	0.56	3
4	(180, 306, 294, 314, 217)	0.92	4
5	(183, 100, 177, 043, 283)	1.56	5
6	(001, 108, 181, 179, 149)	1.61	6
7	(183, 194, 296, 068, 308)	1.63	7
8	(357, 145, 175, 185, 091)	1.68	8
9	(003, 106, 182, 281, 148)	1.71	9
10	(180, 181, 058, 291, 297)	2.12	10
11	(181, 306, 297, 187, 312)	2.20	
12	(358, 139, 176, 272, 097)	2.27	
13	(184, 323, 299, 083, 317)	2.39	11
14	(178, 161, 066, 295, 190)	2.47	
15	(179, 302, 072, 303, 294)	2.64	12
16	(179, 127, 290, 316, 206)	2.73	
17	(184, 214, 292, 062, 085)	2.74	
18	(181, 318, 075, 296, 208)	2.77	
19	(179, 126, 292, 181, 309)	3.06	13
20	(006, 316, 065, 189, 224)	3.12	
21	(177, 282, 182, 283, 039)	3.22	
22	(177, 280, 179, 174, 045)	3.25	
23	(358, 144, 170, 077, 090)	3.26	
24	(177, 043, 288, 066, 310)	3.27	
25	(184, 321, 174, 168, 192)	3.30	
26	(180, 169, 175, 172, 178)	3.32	
27	(177, 261, 051, 066, 287)	3.36	
28	(179, 245, 058, 190, 286)	3.48	
29	(177, 293, 074, 306, 041)	3.65	
30	(182, 125, 067, 309, 058)	3.72	
31	(007, 318, 061, 083, 221)	3.82	
32	(183, 318, 176, 278, 187)	3.97	14

*See the Supplementary Material for the labeling of the five dihedral angles, (ϕ_1_, ϕ_2_, ϕ_3_, ϕ_4_, ϕ_5_). Last column labels the 14 lowest-energy conformers according to the work of Najbauer et al. (2015)*.

### 4.3. Multiple Wells and Torsional Anharmonicity

The number of conformers increases with the size of the system, as shown in Table 1, although this does not imply that all of them are required in the calculation of thermodynamic properties. The importance of each of the *j*-th conformers can be estimated by its contribution, χ_*j*_, to the MS-QH partition function:

(20) $$\begin{array}{l} \chi_{j}=\frac{w_{j}Q_{j}^{\text{rot}}Q_{j}^{\text{QH}}e^{U_{j}/k_{B}T}}{\sum_{j}w_{j}Q_{j}^{\text{rot}}Q_{j}^{\text{QH}}e^{U_{j}/k_{B}T}}=\frac{w_{j}e^{-G_{j}/k_{B}T}}{\sum_{j}w_{j}e^{-G_{j}/k_{B}T}} \end{array}$$

where *G*_*j*_ is the rovibrational Gibbs free energy of the *j*-th conformer:

(21) $$\begin{array}{l} G_{j}=U_{j}-k_{B}T\ln\left[Q_{j}^{\text{rot}}Q_{j}^{\text{QH}}\right] \end{array}$$

We highlight that χ_*j*_ also represents the relative population of the *j*-th conformer.

**Figure 3:**
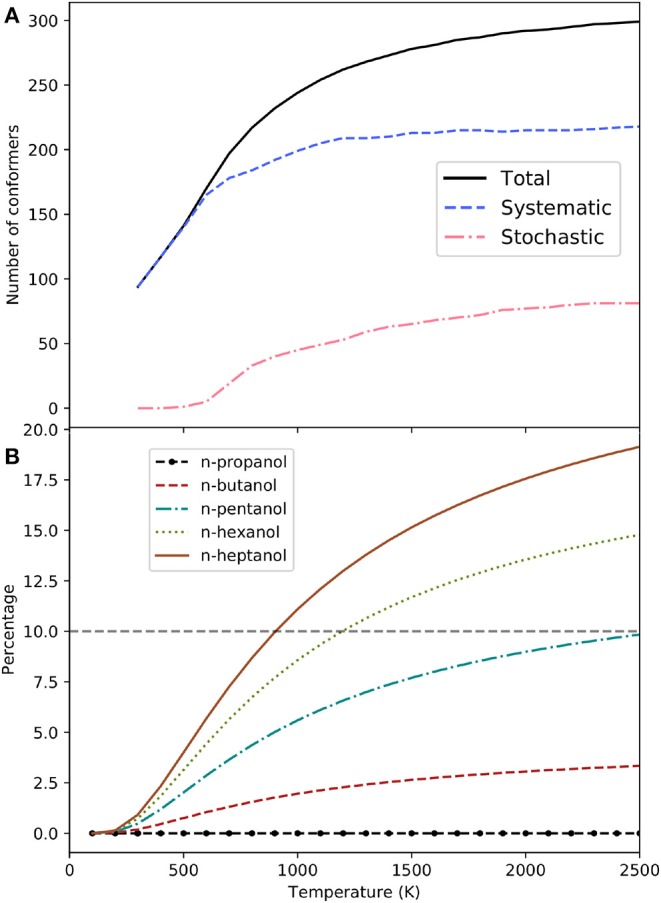
**(A)** Minimum number of n-heptanol conformers needed to achieve $\underset{j}{\sum }\chi_{j}\geq 0.9$ (solid line). Conformers collected by the systematic (dashed line) and stochastic (dash-dotted line) method are also indicated. **(B)** Contribution to the MS-QH partition function of the conformers found during the stochastic search of n-alcohols.

The conformers of each alcohol can be sorted out according to their χ_*j*_ value in such a way that χ_*j*_ ≥ χ_*j*+1_. Considering an error of 10% as acceptable in the evaluation of the MS-QH partition functions, it is possible to estimate the minimum number of conformers needed to recover 90% of the partition function. This number can be factorized into conformers obtained by the systematic method and into conformers obtained by the stochastic algorithm. The analysis has been performed in the range of temperatures between 100 and 2,500 K and it can be found in Table 3. For the specific case of n-heptanol the values are plotted in Figure 3A.

**Table 3:** Minimum number of HL conformer needed to achieve ∑ χ _*j*_ ≥ 0.9 at 300, 1,000, and 2,500 K.

	**300 K**		**1,000 K**		**2,500 K**	
**System**	***J*_1_**	***J*_2_**	***J*_1_**	***J*_2_**	***J*_1_**	***J*_2_**
n-propanol	5	0	5	0	5	0
n-butanol	10	0	12	0	13	0
n-pentanol	20	0	29	1	31	5
n-hexanol	40	0	75	12	84	20
n-heptanol	94	0	199	45	218	81

*The minimum number is split into conformers obtained by the systematic (J_1_) and stochastic (J_2_) algorithms, respectively*.

At the low temperatures regime, the number of conformers that contribute to the free energy is small, and most of them belong to the pool of conformations obtained in a systematic manner. In fact, the stochastic method is not needed for n-propanol and n-butanol in the whole range of temperatures studied here. For n-heptanol (Figure 3A), we notice that (i) the importance of conformers obtained by the stochastic method is negligible at temperatures smaller than 700 K, (ii) even at higher temperatures, only 64% of the total number of conformers are needed to recover 90% of the MS-QH partition function.

It is obvious that the stochastic algorithm is less efficient than the systematic procedure. Consequently, locating conformers arising from the stochastic search requires higher computational cost. Unfortunately, this search is compulsory for the largest alcohols studied. The equilibrium structures retrieved by the stochastic search account for 0, 7, 21, 31, and 37% of the total for n-propanol to n-heptanol HL structures, respectively. As expected (see Figure 3B), their contribution increases with temperature, as well as with system size. However, if we concede deviations up to 10% in the partition function, these conformations are essential for both n-hexanol (from 1,100 K) and n-heptanol (from 900 K), but not for the small n-alcohols.

**Figure 4:**
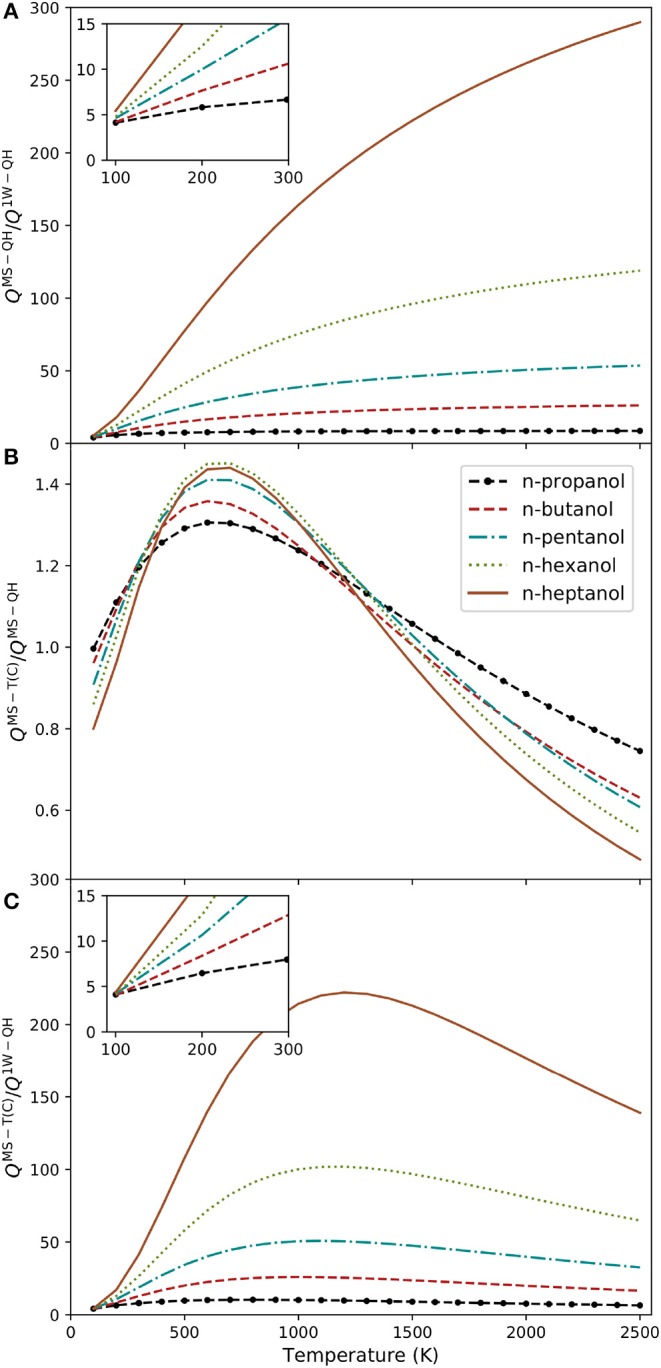
Alcohols partition function ratios plotted at several temperatures: **(A)** *Q*^MS−QH^/*Q*^1W−QH^; **(B)** *Q*^MS−T(C)^/*Q*^MS−QH^; and **(C)** *Q*^MS−T(C)^/*Q*^1W−QH^.

In order to study the repercussion of the multiple wells and torsional anharmonicity in the n-alcohol series, we have employed the MsTor program (Zheng et al., 2013), which can handle the calculation of MS-QH and MS-T(C) and partition functions. The effect of multiple wells was analyzed through the *Q*^MS−QH^ and *Q*^1W−QH^ ratio, where 1W-QH refers to the quasi-harmonic version of the absolute minimum. The evolution of this ratio with temperature is plotted in Figure 4A. The chart shows that the one-well approximation is unsatisfactory even at very low temperatures. The impact of the system size is also substantial; the single conformer approximation turned out worst with longer carbon chains. For instance, at 1,000 K, this ratio increases from 8 to 164 when moving from n-propanol to n-heptanol.

We have also analyzed the variation of the *Q*^MS−T(C)^/*Q*^MS−QH^ ratio with temperature (Figure 4B). Both partition functions are multi-structural, so they include the whole set of conformers. Therefore, the ratio shows the impact of the torsional anharmonicity in the partition functions. Torsional anharmonicity is slightly smaller than the unity at low temperatures (between 0.8 and 1.0) and increases to about 1.4 for n-hexanol and n-heptanol at 700 K. At higher temperatures the ratio declines again. The reason for this behavior is that at high temperatures the density of states of the hindered rotor partition function diminish with increasing temperature, whereas the density of states of the harmonic oscillator remains constant. Therefore, it is crucial to incorporate torsional anharmonicity in the harmonic partition function to retrieve the correct high temperature behavior (Figure 4C). As a general rule, there are two factors that require careful consideration: number of conformations, and torsional anharmonicity. We subscribe to the comment of S. J. Klippenstein, who in a recent review stated (Klippenstein, 2017) “*Historically, the uncertainties in theoretical predictions have been dominated by uncertainties in the barrier height predictions, but this is no longer the case. Uncertainties in the partition function evaluations are now often of comparable or even larger magnitude*.”

## 5. Conclusions

In this work we have presented a combined algorithm able to locate *all* torsional conformers of medium-size acyclic molecules. The algorithm accepts two different strategies for the generation of trial structures: a systematic one, based on the chemical knowledge, and a stochastic one. The torsional PES is efficiently visited, avoiding previously explored areas.

This algorithm was tested in the series of n-alcohols ranging from n-propanol to n-heptanol, as well as in L-serine. We have encountered that the number of conformers arising from the stochastic search is not negligible for n-hexanol and n-heptanol. At the low temperatures regime the contribution to the partition function of the conformers found during the stochastic search is negligible. However, at medium/high temperatures, their exclusion can lead to significant errors. In combination with the MSTor program, the algorithm allows an efficient computation of the MS-QH and MS-T(C) partition functions. The results indicate that the one-well approximation substantially underestimates the magnitude of the partition function when compared with the multi-structural methods. In the case of L-serine, the algorithm was able to locate additional conformers to those described in recent works. In fact, within a range of 4 kcal/mol, the algorithm was able to locate 32 conformers, unlike to the 14 conformers previously reported.

## Data Availability Statement

All datasets generated for this study are included in the article/Supplementary Material.

## Author Contributions

DF-C and AF-R have equally contributed to the development of the algorithm, the DFT calculations and the evaluation of multi-structural partition functions for the n-alcohols and L-serine here presented.

### Conflict of Interest

The authors declare that the research was conducted in the absence of any commercial or financial relationships that could be construed as a potential conflict of interest.

## Supplementary Material

It contains the MS-QH and MS-T(C) partition functions, as well as the electronic energy and the Cartesian coordinates of all conformations for the species ranging from n-propanol to n-heptanol. For L-serine, Cartesian coordinates of the 60 HL conformers are also listed, as well as a figure labeling the targeted torsions.
