# Supplementary material for: A Combined Systematic-Stochastic Algorithm for the Conformational Search in Flexible Acyclic Molecules
Source: Front Chem. 2020 Jan 28;8:16. doi: 10.3389/fchem.2020.00016 (PMC6997476; doi:10.3389/fchem.2020.00016)
Supplement: Supplementary file 1 [file Table_1.pdf]

Supplementary Material for 'A Combined Systematic-Stochastic Algorithm for the Conformational Search in Flexible Acyclic Molecules'

1 SUPPLEMENTARY FIGURES

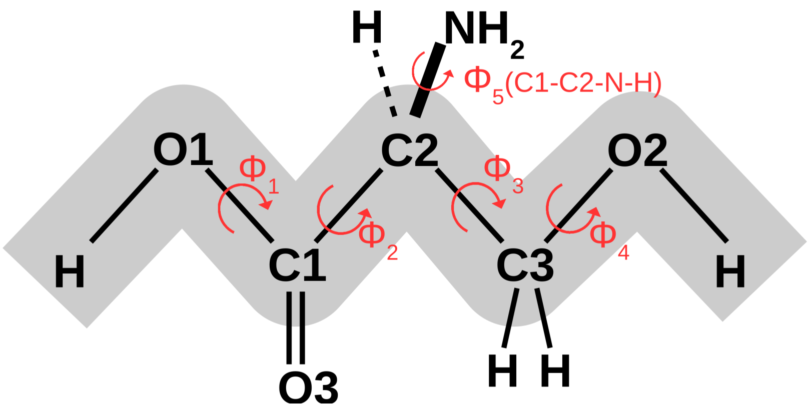

Figure S1. Target torsions in L-serine.

2 SUPPLEMENTARY TABLES

Table S1. Contribution of non-intuitive conformers to the MS-QH partition function in the 100-2500 K range ($\lambda^{\mathrm{ZPE}} = 0.951$).

T (K)	n-propanol	n-butanol	n-pentanol	n-hexanol	n-heptanol
100.00	0.0000	0.0000	0.0000	0.0000	0.0000
200.00	0.0000	0.0310	0.0760	0.1100	0.1400
298.15	0.0000	0.1880	0.4730	0.7230	0.9100
300.00	0.0000	0.1920	0.4840	0.7400	0.9310
400.00	0.0000	0.4570	1.1920	1.8470	2.3380
500.00	0.0000	0.7550	2.0240	3.1420	4.0060
600.00	0.0000	1.0460	2.8620	4.4360	5.6860
700.00	0.0000	1.3140	3.6500	5.6440	7.2610
800.00	0.0000	1.5540	4.3700	6.7360	8.6880
900.00	0.0000	1.7690	5.0170	7.7110	9.9640
1000.00	0.0000	1.9590	5.5950	8.5780	11.0960
1100.00	0.0000	2.1280	6.1120	9.3480	12.1020
1200.00	0.0000	2.2790	6.5750	10.0330	12.9960
1300.00	0.0000	2.4140	6.9900	10.6460	13.7930
1400.00	0.0000	2.5350	7.3630	11.1940	14.5060
1500.00	0.0000	2.6440	7.7010	11.6880	15.1480
1600.00	0.0000	2.7430	8.0070	12.1350	15.7270
1700.00	0.0000	2.8330	8.2850	12.5400	16.2510
1800.00	0.0000	2.9150	8.5390	12.9090	16.7280
1900.00	0.0000	2.9900	8.7720	13.2460	17.1630
2000.00	0.0000	3.0590	8.9860	13.5560	17.5620
2100.00	0.0000	3.1230	9.1840	13.8400	17.9280
2200.00	0.0000	3.1820	9.3660	14.1030	18.2660
2300.00	0.0000	3.2360	9.5350	14.3460	18.5780
2400.00	0.0000	3.2870	9.6930	14.5710	18.8680
2500.00	0.0000	3.3340	9.8390	14.7810	19.1370

Table S2. MS-QH and MS-T(C) rovibrational partition functions ($\lambda^{\rm ZPE} = 0.951$) for n-propanol in the 100-2500 K range.

T (K)	$Q^{\rm MS-QH}$	$Q^{\rm MS-T(C)}$	$Q^{\rm MS-T(C)}/Q^{\rm MS-QH}$
100.00	4.7775E+04	4.7786E+04	1.0002
200.00	4.2694E+05	4.7648E+05	1.1160
298.15	2.2552E+06	2.7108E+06	1.2020
300.00	2.3220E+06	2.7944E+06	1.2034
400.00	1.0600E+07	1.3373E+07	1.2616
500.00	4.5099E+07	5.8375E+07	1.2944
600.00	1.8445E+08	2.4094E+08	1.3063
700.00	7.2988E+08	9.5014E+08	1.3018
800.00	2.7945E+09	3.5910E+09	1.2850
900.00	1.0341E+10	1.3025E+10	1.2595
1000.00	3.6957E+10	4.5393E+10	1.2283
1100.00	1.2755E+11	1.5219E+11	1.1932
1200.00	4.2522E+11	4.9152E+11	1.1559
1300.00	1.3702E+12	1.5315E+12	1.1177
1400.00	4.2717E+12	4.6104E+12	1.0793
1500.00	1.2896E+13	1.3429E+13	1.0413
1600.00	3.7744E+13	3.7900E+13	1.0041
1700.00	1.0721E+14	1.0379E+14	0.9681
1800.00	2.9593E+14	2.7619E+14	0.9333
1900.00	7.9462E+14	7.1502E+14	0.8998
2000.00	2.0780E+15	1.8032E+15	0.8678
2100.00	5.2983E+15	4.4353E+15	0.8371
2200.00	1.3185E+16	1.0652E+16	0.8079
2300.00	3.2060E+16	2.5005E+16	0.7799
2400.00	7.6239E+16	5.7435E+16	0.7534
2500.00	1.7748E+17	1.2921E+17	0.7280

Table S3. MS-QH and MS-T(C) rovibrational partition functions ($\lambda^{\mathrm{ZPE}} = 0.951$) for n-butanol in the 100-2500 K range.

T (K)	$Q^{\mathrm{MS-QH}}$	$Q^{\mathrm{MS-T(C)}}$	$Q^{\mathrm{MS-T(C)}}/Q^{\mathrm{MS-QH}}$
100.00	1.5362E+05	1.4823E+05	0.9649
200.00	3.0531E+06	3.3611E+06	1.1009
298.15	3.1262E+07	3.8083E+07	1.2182
300.00	3.2561E+07	3.9727E+07	1.2201
400.00	2.6649E+08	3.4704E+08	1.3023
500.00	1.9308E+09	2.5999E+09	1.3465
600.00	1.3003E+10	1.7675E+10	1.3593
700.00	8.2572E+10	1.1134E+11	1.3484
800.00	4.9645E+11	6.5578E+11	1.3209
900.00	2.8300E+12	3.6290E+12	1.2823
1000.00	1.5311E+13	1.8939E+13	1.2370
1100.00	7.8723E+13	9.3492E+13	1.1876
1200.00	3.8520E+14	4.3783E+14	1.1366
1300.00	1.7967E+15	1.9502E+15	1.0854
1400.00	8.0027E+15	8.2830E+15	1.0350
1500.00	3.4099E+16	3.3625E+16	0.9861
1600.00	1.3925E+17	1.3076E+17	0.9390
1700.00	5.4594E+17	4.8812E+17	0.8941
1800.00	2.0588E+18	1.7528E+18	0.8514
1900.00	7.4802E+18	6.0655E+18	0.8109
2000.00	2.6229E+19	2.0265E+19	0.7726
2100.00	8.8899E+19	6.5478E+19	0.7365
2200.00	2.9170E+20	2.0493E+20	0.7025
2300.00	9.2792E+20	6.2221E+20	0.6705
2400.00	2.8658E+21	1.8353E+21	0.6404
2500.00	8.6037E+21	5.2658E+21	0.6120

Table S4. MS-QH and MS-T(C) rovibrational partition functions ($\lambda^{\mathrm{ZPE}} = 0.951$) for n-pentanol in the 100-2500 K range.

T (K)	$Q^{\mathrm{MS-QH}}$	$Q^{\mathrm{MS-T(C)}}$	$Q^{\mathrm{MS-T(C)}}/Q^{\mathrm{MS-QH}}$
100.00	5.3048E+05	4.8430E+05	0.9129
200.00	2.2845E+07	2.4570E+07	1.0755
298.15	4.3481E+08	5.3032E+08	1.2197
300.00	4.5771E+08	5.5934E+08	1.2220
400.00	6.4243E+09	8.5285E+09	1.3275
500.00	7.6400E+10	1.0615E+11	1.3894
600.00	8.2289E+11	1.1632E+12	1.4136
700.00	8.1961E+12	1.1544E+13	1.4085
800.00	7.5987E+13	1.0503E+14	1.3822
900.00	6.5755E+14	8.8205E+14	1.3414
1000.00	5.3213E+15	6.8726E+15	1.2915
1100.00	4.0353E+16	4.9890E+16	1.2363
1200.00	2.8737E+17	3.3872E+17	1.1787
1300.00	1.9263E+18	2.1583E+18	1.1204
1400.00	1.2183E+19	1.2951E+19	1.0630
1500.00	7.2870E+19	7.3401E+19	1.0073
1600.00	4.1324E+20	3.9410E+20	0.9537
1700.00	2.2270E+21	2.0101E+21	0.9026
1800.00	1.1431E+22	9.7641E+21	0.8542
1900.00	5.6012E+22	4.5283E+22	0.8085
2000.00	2.6256E+23	2.0096E+23	0.7654
2100.00	1.1798E+24	8.5530E+23	0.7250
2200.00	5.0916E+24	3.4980E+24	0.6870
2300.00	2.1144E+25	1.3774E+25	0.6514
2400.00	8.4632E+25	5.2313E+25	0.6181
2500.00	3.2707E+26	1.9196E+26	0.5869

Table S5. MS-QH and MS-T(C) rovibrational partition functions ($\lambda^{\mathrm{ZPE}} = 0.951$) for n-hexanol in the 100-2500 K range.

T (K)	$Q^{\mathrm{MS-QH}}$	$Q^{\mathrm{MS-T(C)}}$	$Q^{\mathrm{MS-T(C)}}/Q^{\mathrm{MS-QH}}$
100.00	1.6675E+06	1.4417E+06	0.8646
200.00	1.6417E+08	1.7004E+08	1.0358
298.15	5.9314E+09	7.1530E+09	1.2060
300.00	6.3128E+09	7.6313E+09	1.2089
400.00	1.5488E+11	2.0767E+11	1.3408
500.00	3.0743E+12	4.3679E+12	1.4208
600.00	5.3708E+13	7.8081E+13	1.4538
700.00	8.4886E+14	1.2308E+15	1.4499
800.00	1.2253E+16	1.7394E+16	1.4196
900.00	1.6227E+17	2.2254E+17	1.3714
1000.00	1.9776E+18	2.5954E+18	1.3124
1100.00	2.2248E+19	2.7752E+19	1.2474
1200.00	2.3174E+20	2.7345E+20	1.1800
1300.00	2.2421E+21	2.4941E+21	1.1124
1400.00	2.0211E+22	2.1147E+22	1.0463
1500.00	1.7028E+23	1.6733E+23	0.9827
1600.00	1.3450E+24	1.2402E+24	0.9221
1700.00	9.9893E+24	8.6391E+24	0.8648
1800.00	6.9961E+25	5.6743E+25	0.8111
1900.00	4.6332E+26	3.5247E+26	0.7607
2000.00	2.9091E+27	2.0765E+27	0.7138
2100.00	1.7360E+28	1.1633E+28	0.6701
2200.00	9.8704E+28	6.2128E+28	0.6294
2300.00	5.3586E+29	3.1704E+29	0.5916
2400.00	2.7838E+30	1.5493E+30	0.5565
2500.00	1.3866E+31	7.2651E+30	0.5240

Table S6. MS-QH and MS-T(C) rovibrational partition functions ($\lambda^{\mathrm{ZPE}} = 0.951$) for n-heptanol in the 100-2500 K range.

T (K)	$Q^{\mathrm{MS-QH}}$	$Q^{\mathrm{MS-T(C)}}$	$Q^{\mathrm{MS-T(C)}}/Q^{\mathrm{MS-QH}}$
100.00	5.6435E+06	4.5459E+06	0.8055
200.00	1.3044E+09	1.2701E+09	0.9737
298.15	9.0264E+10	1.0468E+11	1.1597
300.00	9.7137E+10	1.1296E+11	1.1629
400.00	4.1675E+12	5.4643E+12	1.3112
500.00	1.3769E+14	1.9312E+14	1.4026
600.00	3.8875E+15	5.6034E+15	1.4414
700.00	9.7141E+16	1.3975E+17	1.4386
800.00	2.1759E+18	3.0590E+18	1.4059
900.00	4.3960E+19	5.9491E+19	1.3533
1000.00	8.0461E+20	1.0368E+21	1.2886
1100.00	1.3395E+22	1.6310E+22	1.2176
1200.00	2.0364E+23	2.3299E+23	1.1441
1300.00	2.8381E+24	3.0393E+24	1.0709
1400.00	3.6401E+25	3.6389E+25	0.9997
1500.00	4.3127E+26	4.0175E+26	0.9316
1600.00	4.7377E+27	4.1082E+27	0.8671
1700.00	4.8428E+28	3.9067E+28	0.8067
1800.00	4.6221E+29	3.4681E+29	0.7503
1900.00	4.1325E+30	2.8844E+30	0.6980
2000.00	3.4719E+31	2.2550E+31	0.6495
2100.00	2.7491E+32	1.6623E+32	0.6047
2200.00	2.0573E+33	1.1589E+33	0.5633
2300.00	1.4590E+34	7.6622E+33	0.5252
2400.00	9.8295E+34	4.8164E+34	0.4900
2500.00	6.3061E+35	2.8855E+35	0.4576

3 CARTESIAN COORDINATES

3.1 Conformers of n-propanol

```
12
 * E =   +0.000  kcal/mol  ;  (1)  182_062
 H    +0.00000    +0.00000    +0.00000
 O    +0.00000    +0.00000    +0.95322
 C    -0.89694    +0.00000    +1.27591
 C    -0.85310    +0.05562    +2.78173
 H    -1.44326    +0.86645    +0.89135
 H    -1.42332    -0.90067    +0.95134
 C    -0.19011    +1.31530    +3.29974
 H    -1.87401    -0.01945    +3.15614
 H    -0.31829    -0.82314    +3.14081
 H    -0.15222    +1.32303    +4.38631
 H    +0.82542    +1.39286    +2.92204
 H    -0.73422    +2.20287    +2.97961
12
 * E =   +0.196  kcal/mol  ;  (2)  180_180
 H    +0.00000    +0.00000    +0.00000
 O    +0.00000    +0.00000    +0.95339
 C    -0.89791    +0.00000    +1.27388
 C    -0.86153    -0.00022    +2.77925
 H    -1.43679    +0.88295    +0.91819
 H    -1.43688    -0.88278    +0.91794
 C    -2.24952    -0.00020    +3.38528
 H    -0.30127    -0.87376    +3.10944
 H    -0.30114    +0.87315    +3.10968
 H    -2.20342    -0.00042    +4.47094
 H    -2.81552    +0.87866    +3.08083
 H    -2.81569    -0.87884    +3.08048
12
 * E =   +0.200  kcal/mol  ;  (3)  065_060
 H    +0.00000    +0.00000    +0.00000
 O    +0.00000    +0.00000    +0.95443
 C    -0.90188    +0.00000    +1.26676
 C    -1.64766    -1.26798    +0.91199
 H    -0.83293    +0.10126    +2.34784
 H    -1.43996    +0.87366    +0.89127
 C    -1.00259    -2.50914    +1.49287
 H    -2.67496    -1.17255    +1.26562
 H    -1.70887    -1.35229    -0.17547
 H    -1.53056    -3.41027    +1.19094
 H    +0.03101    -2.59355    +1.16807
 H    -1.00228    -2.47190    +2.58092
```

```
12
 * E =   +0.215  kcal/mol  ;  (4)  062_177
 H    +0.00000    +0.00000    +0.00000
 O    +0.00000    +0.00000    +0.95433
 C    −0.90150    +0.00000    +1.26744
 C    −1.67931    −1.22363    +0.83822
 H    −0.83520    +0.03632    +2.35249
 H    −1.42473    +0.90601    +0.94708
 C    −3.08712    −1.23272    +1.39870
 H    −1.71695    −1.25939    −0.25257
 H    −1.13437    −2.10993    +1.16009
 H    −3.63523    −2.11455    +1.07810
 H    −3.07574    −1.22975    +2.48713
 H    −3.64885    −0.35885    +1.07305
12
 * E =   +0.265  kcal/mol  ;  (5)  067_297
 H    +0.00000    +0.00000    +0.00000
 O    +0.00000    +0.00000    +0.95391
 C    −0.90030    +0.00000    +1.26918
 C    −1.64399    −1.27803    +0.94640
 H    −0.83317    +0.13238    +2.34552
 H    −1.44716    +0.85931    +0.86872
 C    −1.79254    −1.51974    −0.54258
 H    −1.11875    −2.11337    +1.40791
 H    −2.62895    −1.22496    +1.41115
 H    −2.36959    −2.41876    −0.74267
 H    −2.30019    −0.68735    −1.02919
 H    −0.82496    −1.65352    −1.02416
```

3.2 Conformers of n-butanol

```
15
 * E =   +0.000  kcal/mol ;  (1) 181_062_181
 H    +0.00000    +0.00000    +0.00000
 O    +0.00000    +0.00000    +0.95329
 C    -0.89718    +0.00000    +1.27553
 C    -0.85604    +0.03667    +2.78156
 H    -1.43987    +0.87267    +0.90013
 H    -1.42684    -0.89440    +0.93940
 C    -0.20404    +1.29052    +3.33054
 H    -1.87715    -0.04816    +3.15709
 H    -0.31594    -0.84331    +3.13335
 C    -0.14432    +1.29997    +4.84431
 H    +0.79855    +1.37300    +2.91526
 H    -0.75658    +2.16380    +2.97875
 H    +0.32388    +2.20687    +5.21977
 H    -1.14066    +1.23741    +5.27979
 H    +0.43006    +0.45364    +5.21743
15
 * E =   +0.214  kcal/mol ;  (2) 065_060_183
 H    +0.00000    +0.00000    +0.00000
 O    +0.00000    +0.00000    +0.95448
 C    -0.90214    +0.00000    +1.26621
 C    -1.65309    -1.26072    +0.89859
 H    -0.83379    +0.09130    +2.34818
 H    -1.43655    +0.87943    +0.89915
 C    -1.03293    -2.51783    +1.47566
 H    -2.68522    -1.16265    +1.24098
 H    -1.70405    -1.34324    -0.19094
 C    -1.75815    -3.77806    +1.05020
 H    +0.01235    -2.56616    +1.17456
 H    -1.03112    -2.44366    +2.56425
 H    -1.30067    -4.66610    +1.47982
 H    -2.80084    -3.75863    +1.36374
 H    -1.74441    -3.89146    -0.03276
15
 * E =   +0.277  kcal/mol ;  (3) 180_180_180
 H    +0.00000    +0.00000    +0.00000
 O    +0.00000    +0.00000    +0.95343
 C    -0.89812    +0.00000    +1.27345
 C    -0.86114    -0.00100    +2.77859
 H    -1.43594    +0.88326    +0.91741
 H    -1.43641    -0.88249    +0.91622
 C    -2.24383    -0.00091    +3.39958
 H    -0.30102    -0.87545    +3.11061
```

```
H   −0.30041   +0.87263   +3.11174
C   −2.20131   −0.00181   +4.91426
H   −2.79876   +0.87183   +3.05151
H   −2.79932   −0.87288   +3.05050
H   −3.20094   −0.00196   +5.34211
H   −1.67928   −0.88002   +5.28985
H   −1.67905   +0.87581   +5.29090
15
* E =  +0.291  kcal/mol ; (4) 061_177_180
H   +0.00000   +0.00000   +0.00000
O   +0.00000   +0.00000   +0.95436
C   −0.90187   +0.00000   +1.26649
C   −1.68522   −1.21172   +0.81539
H   −0.83571   +0.01792   +2.35190
H   −1.41839   +0.91461   +0.96059
C   −3.09687   −1.23830   +1.36908
H   −1.72528   −1.22870   −0.27707
H   −1.14537   −2.10857   +1.12021
C   −3.88489   −2.44780   +0.90819
H   −3.05517   −1.22400   +2.45907
H   −3.62013   −0.32764   +1.07349
H   −4.89170   −2.44833   +1.31879
H   −3.96973   −2.46949   −0.17690
H   −3.39980   −3.37158   +1.21803
15
* E =  +0.297  kcal/mol ; (5) 067_296_182
H   +0.00000   +0.00000   +0.00000
O   +0.00000   +0.00000   +0.95381
C   −0.90009   +0.00000   +1.26939
C   −1.64281   −1.28043   +0.95605
H   −0.83203   +0.13828   +2.34492
H   −1.44828   +0.85691   +0.86594
C   −1.82299   −1.53325   −0.52874
H   −1.10790   −2.11628   +1.40823
H   −2.62258   −1.23419   +1.43547
C   −2.61871   −2.79024   −0.81715
H   −2.31932   −0.67332   −0.98360
H   −0.84571   −1.61780   −1.00730
H   −2.73123   −2.95690   −1.88570
H   −2.12976   −3.66582   −0.39418
H   −3.61536   −2.72906   −0.38366
15
* E =  +0.424  kcal/mol ; (6) 180_056_060
H   +0.00000   +0.00000   +0.00000
O   +0.00000   +0.00000   +0.95330
```

```
C    −0.89730   +0.00000   +1.27523
C    −0.85534   −0.00603   +2.78319
H    −1.43312   +0.88246   +0.91631
H    −1.43682   −0.88114   +0.92048
C    −0.06223   +1.14628   +3.37690
H    −1.88305   +0.01831   +3.14987
H    −0.42265   −0.95028   +3.11225
C    −0.59717   +2.51444   +3.00259
H    −0.06020   +1.04066   +4.46145
H    +0.97276   +1.05987   +3.05172
H    −0.05470   +3.30174   +3.52077
H    −0.49642   +2.70236   +1.93566
H    −1.65096   +2.61398   +3.26175
15
* E =  +0.617 kcal/mol ; (7) 063_055_061
H    +0.00000   +0.00000   +0.00000
O    +0.00000   +0.00000   +0.95457
C    −0.90162   +0.00000   +1.26807
C    −1.66948   −1.23883   +0.85802
H    −0.83069   +0.06453   +2.35070
H    −1.42965   +0.89438   +0.92882
C    −1.00462   −2.54222   +1.26811
H    −2.66929   −1.17766   +1.29282
H    −1.80921   −1.22836   −0.22549
C    −0.82750   −2.69017   +2.76648
H    −1.60165   −3.37069   +0.88794
H    −0.03178   −2.61216   +0.78347
H    −0.43728   −3.67337   +3.01824
H    −0.12882   −1.95516   +3.15933
H    −1.77465   −2.56671   +3.29076
15
* E =  +0.830 kcal/mol ; (8) 066_301_301
H    +0.00000   +0.00000   +0.00000
O    +0.00000   +0.00000   +0.95436
C    −0.90107   +0.00000   +1.26879
C    −1.64839   −1.27178   +0.92587
H    −0.83544   +0.10932   +2.34778
H    −1.43884   +0.87371   +0.89179
C    −1.73613   −1.56150   −0.56443
H    −1.15976   −2.10697   +1.42624
H    −2.65534   −1.19560   +1.34087
C    −2.42225   −0.47207   −1.36716
H    −0.73439   −1.73883   −0.96008
H    −2.26979   −2.50060   −0.70404
H    −2.52639   −0.75898   −2.41067
```

```
 H    -3.41892    -0.26872    -0.97851
 H    -1.86971    +0.46614    -1.34470
15
 * E =  +0.957  kcal/mol ; (9) 059_181_296
 H    +0.00000    +0.00000    +0.00000
 O    +0.00000    +0.00000    +0.95447
 C    -0.90213    +0.00000    +1.26621
 C    -1.69113    -1.19265    +0.77238
 H    -0.83127    -0.00697    +2.35016
 H    -1.41218    +0.92580    +0.98127
 C    -3.13926    -1.20014    +1.23342
 H    -1.66458    -1.19770    -0.31974
 H    -1.18790    -2.10312    +1.09951
 C    -3.30393    -1.32281    +2.73688
 H    -3.63471    -0.29248    +0.88442
 H    -3.65409    -2.02873    +0.74894
 H    -4.35271    -1.41096    +3.01005
 H    -2.78849    -2.20472    +3.11415
 H    -2.90452    -0.45845    +3.26255
15
 * E =  +0.975  kcal/mol ; (10) 177_175_063
 H    +0.00000    +0.00000    +0.00000
 O    +0.00000    +0.00000    +0.95347
 C    -0.89792    +0.00000    +1.27416
 C    -0.85077    -0.06397    +2.77945
 H    -1.42136    +0.90931    +0.96244
 H    -1.44754    -0.85398    +0.87233
 C    -2.21420    +0.05261    +3.43857
 H    -0.37107    -0.99839    +3.07227
 H    -0.20239    +0.74055    +3.12410
 C    -3.16627    -1.07918    +3.09817
 H    -2.07280    +0.08669    +4.51799
 H    -2.66862    +1.00646    +3.16515
 H    -4.09058    -0.99902    +3.66547
 H    -3.43404    -1.08341    +2.04370
 H    -2.71987    -2.04565    +3.32799
15
 * E =  +1.064  kcal/mol ; (11) 062_174_065
 H    +0.00000    +0.00000    +0.00000
 O    +0.00000    +0.00000    +0.95439
 C    -0.90203    +0.00000    +1.26615
 C    -1.66632    -1.23195    +0.83345
 H    -0.83675    +0.03195    +2.35182
 H    -1.41591    +0.90924    +0.94655
 C    -3.05804    -1.33563    +1.43540
```

```
 H   -1.74323   -1.24280   -0.25731
 H   -1.07612   -2.10387   +1.11294
 C   -4.00803   -0.23692   +0.99549
 H   -3.48143   -2.30207   +1.16505
 H   -2.97841   -1.33654   +2.52366
 H   -5.00913   -0.40554   +1.38503
 H   -3.68751   +0.74313   +1.34136
 H   -4.07981   -0.19603   -0.09062
15
 * E =   +1.548  kcal/mol  ;  (12)  178_068_284
 H   +0.00000   +0.00000   +0.00000
 O   +0.00000   +0.00000   +0.95358
 C   -0.89965   +0.00000   +1.26972
 C   -0.91013   -0.03742   +2.77759
 H   -1.42654   +0.89492   +0.92476
 H   -1.43608   -0.87087   +0.88495
 C   -0.38959   +1.21485   +3.46781
 H   -1.94416   -0.20658   +3.08032
 H   -0.34214   -0.90856   +3.10718
 C   +1.12014   +1.36429   +3.46411
 H   -0.84895   +2.09091   +3.00553
 H   -0.73892   +1.20471   +4.50056
 H   +1.42194   +2.25378   +4.01360
 H   +1.59202   +0.50588   +3.93972
 H   +1.50959   +1.43150   +2.45402
15
 * E =   +1.589  kcal/mol  ;  (13)  061_068_290
 H   +0.00000   +0.00000   +0.00000
 O   +0.00000   +0.00000   +0.95446
 C   -0.90231   +0.00000   +1.26563
 C   -1.70828   -1.21123   +0.84485
 H   -0.83399   +0.04024   +2.35098
 H   -1.41384   +0.90998   +0.94138
 C   -1.28321   -2.53328   +1.46413
 H   -2.74755   -1.01303   +1.11200
 H   -1.69115   -1.29199   -0.24599
 C   +0.04712   -3.07341   +0.97401
 H   -1.25403   -2.41884   +2.54918
 H   -2.06092   -3.26997   +1.26148
 H   +0.24854   -4.05235   +1.40381
 H   +0.04250   -3.18684   -0.10987
 H   +0.86576   -2.40984   +1.23288
15
 * E =   +2.009  kcal/mol  ;  (14)  062_275_056
 H   +0.00000   +0.00000   +0.00000
```

```
O   +0.00000   +0.00000   +0.95320
C   −0.89769   +0.00000   +1.27372
C   −1.69064   −1.23116   +0.87510
H   −0.80749   +0.04761   +2.35522
H   −1.42315   +0.90646   +0.95508
C   −2.34179   −1.16637   −0.49779
H   −1.02031   −2.08947   +0.93699
H   −2.47256   −1.39436   +1.61728
C   −1.38921   −0.88594   −1.64552
H   −2.85381   −2.11008   −0.68320
H   −3.11806   −0.39980   −0.48688
H   −1.89220   −0.98097   −2.60480
H   −0.99813   +0.13112   −1.60471
H   −0.54976   −1.58046   −1.64578
15
* E =  +2.465 kcal/mol ; (15) 070_300_091
H   +0.00000   +0.00000   +0.00000
O   +0.00000   +0.00000   +0.95402
C   −0.90137   +0.00000   +1.26658
C   −1.63671   −1.30138   +1.02591
H   −0.84305   +0.20242   +2.33286
H   −1.45960   +0.82335   +0.81079
C   −1.73551   −1.69947   −0.44395
H   −1.14991   −2.09812   +1.58929
H   −2.63553   −1.18531   +1.44804
C   −0.59329   −2.57933   −0.92191
H   −2.67132   −2.22984   −0.61375
H   −1.79861   −0.79785   −1.05972
H   −0.67900   −2.79862   −1.98390
H   +0.37776   −2.12324   −0.74637
H   −0.59603   −3.52743   −0.38745
```

3.3 Conformers of n-pentanol

```
18
 * E =   +0.000  kcal/mol  ;  (1)  182_062_181_180
 H    +0.00000    +0.00000    +0.00000
 O    +0.00000    +0.00000    +0.95328
 C    -0.89721    +0.00000    +1.27538
 C    -0.85617    +0.03778    +2.78146
 H    -1.44019    +0.87207    +0.89916
 H    -1.42632    -0.89499    +0.93993
 C    -0.20545    +1.29263    +3.32858
 H    -1.87738    -0.04800    +3.15622
 H    -0.31538    -0.84179    +3.13293
 C    -0.13508    +1.31324    +4.84297
 H    +0.79722    +1.37980    +2.91125
 H    -0.76067    +2.16744    +2.98091
 C    +0.50957    +2.57282    +5.38523
 H    -1.14105    +1.21102    +5.25417
 H    +0.42223    +0.44047    +5.18675
 H    +0.55312    +2.56578    +6.47197
 H    +1.52682    +2.68155    +5.01312
 H    -0.04631    +3.45857    +5.08188
18
 * E =   +0.222  kcal/mol  ;  (2)  065_061_183_181
 H    +0.00000    +0.00000    +0.00000
 O    +0.00000    +0.00000    +0.95448
 C    -0.90208    +0.00000    +1.26637
 C    -1.65351    -1.26064    +0.89890
 H    -0.83361    +0.09145    +2.34830
 H    -1.43655    +0.87933    +0.89914
 C    -1.03330    -2.51719    +1.47595
 H    -2.68537    -1.16139    +1.24147
 H    -1.70434    -1.34218    -0.19062
 C    -1.74627    -3.78610    +1.05128
 H    +0.01466    -2.56543    +1.17967
 H    -1.03590    -2.44813    +2.56612
 C    -1.12690    -5.03574    +1.64359
 H    -2.79671    -3.72559    +1.34131
 H    -1.73761    -3.85508    -0.03789
 H    -1.65094    -5.93344    +1.32383
 H    -0.08505    -5.13503    +1.34389
 H    -1.15278    -5.00695    +2.73159
18
 * E =   +0.233  kcal/mol  ;  (3)  180_180_180_180
 H    +0.00000    +0.00000    +0.00000
 O    +0.00000    +0.00000    +0.95344
```

```
C    −0.89826   +0.00000   +1.27308
C    −0.86153   −0.00112   +2.77824
H    −1.43583   +0.88331   +0.91691
H    −1.43636   −0.88241   +0.91551
C    −2.24457   −0.00058   +3.39733
H    −0.30166   −0.87583   +3.10965
H    −0.30052   +0.87243   +3.11081
C    −2.21722   −0.00129   +4.91362
H    −2.80146   +0.87293   +3.05035
H    −2.80261   −0.87300   +3.04949
C    −3.60196   −0.00059   +5.52813
H    −1.66160   −0.87349   +5.26086
H    −1.66040   +0.86985   +5.26161
H    −3.55770   −0.00077   +6.61463
H    −4.16670   +0.87795   +5.22032
H    −4.16779   −0.87833   +5.22002
18
* E =  +0.243  kcal/mol ; (4) 061_177_180_180
H    +0.00000   +0.00000   +0.00000
O    +0.00000   +0.00000   +0.95437
C    −0.90198   +0.00000   +1.26624
C    −1.68400   −1.21376   +0.81817
H    −0.83614   +0.02101   +2.35159
H    −1.41905   +0.91334   +0.95763
C    −3.09534   −1.23864   +1.37149
H    −1.72273   −1.23354   −0.27419
H    −1.14302   −2.10866   +1.12639
C    −3.89359   −2.44764   +0.92256
H    −3.05677   −1.21997   +2.46279
H    −3.62237   −0.32992   +1.07194
C    −5.30132   −2.46516   +1.48252
H    −3.93250   −2.46666   −0.16762
H    −3.36789   −3.35509   +1.22265
H    −5.85415   −3.33964   +1.14769
H    −5.28948   −2.47812   +2.57105
H    −5.85778   −1.58267   +1.17121
18
* E =  +0.247  kcal/mol ; (5) 067_296_183_180
H    +0.00000   +0.00000   +0.00000
O    +0.00000   +0.00000   +0.95387
C    −0.90036   +0.00000   +1.26887
C    −1.64598   −1.27652   +0.94614
H    −0.83270   +0.13078   +2.34538
H    −1.44617   +0.86086   +0.87070
C    −1.82244   −1.51904   −0.54042
```

```
H   −1.11433   −2.11601   +1.39518
H   −2.62658   −1.22951   +1.42362
C   −2.62581   −2.76719   −0.85249
H   −2.31207   −0.65359   −0.99507
H   −0.84440   −1.60935   −1.01931
C   −2.78841   −3.00903   −2.33920
H   −2.14029   −3.62803   −0.39096
H   −3.60766   −2.68575   −0.38396
H   −3.36869   −3.90708   −2.53706
H   −3.29698   −2.17435   −2.81885
H   −1.82119   −3.12708   −2.82495
18
 * E =   +0.463  kcal/mol ; (6) 179_055_059_175
H   +0.00000   +0.00000   +0.00000
O   +0.00000   +0.00000   +0.95330
C   −0.89729   +0.00000   +1.27525
C   −0.85575   −0.01294   +2.78356
H   −1.43158   +0.88476   +0.92002
H   −1.43814   −0.87865   +0.91634
C   −0.04244   +1.12181   +3.38282
H   −1.88306   +0.02725   +3.14962
H   −0.44001   −0.96574   +3.10960
C   −0.53105   +2.50770   +3.00675
H   −0.05418   +1.02173   +4.46932
H   +0.99546   +1.01259   +3.07038
C   +0.22495   +3.60377   +3.73069
H   −0.42453   +2.65045   +1.93119
H   −1.59770   +2.59153   +3.22621
H   −0.12562   +4.59219   +3.44238
H   +0.10964   +3.51246   +4.80958
H   +1.28964   +3.55210   +3.50952
18
 * E =   +0.575  kcal/mol ; (7) 182_063_185_297
H   +0.00000   +0.00000   +0.00000
O   +0.00000   +0.00000   +0.95327
C   −0.89713   +0.00000   +1.27557
C   −0.85814   +0.04714   +2.78185
H   −1.44229   +0.86839   +0.89391
H   −1.42381   −0.89830   +0.94538
C   −0.21172   +1.31221   +3.31424
H   −1.88007   −0.04813   +3.14884
H   −0.30886   −0.82533   +3.13949
C   −0.04332   +1.32976   +4.82402
H   +0.76093   +1.42467   +2.83882
H   −0.80895   +2.17502   +3.00803
```

```
C   −1.35065   +1.28570   +5.59189
H   +0.58353   +0.48792   +5.12289
H   +0.50523   +2.22875   +5.10465
H   −1.18268   +1.37340   +6.66289
H   −2.00616   +2.10276   +5.29244
H   −1.88765   +0.35453   +5.42403
18
* E =  +0.645  kcal/mol ; (8) 063_054_060_175
H   +0.00000   +0.00000   +0.00000
O   +0.00000   +0.00000   +0.95456
C   −0.90142   +0.00000   +1.26860
C   −1.66933   −1.24021   +0.86137
H   −0.82958   +0.06631   +2.35100
H   −1.43027   +0.89356   +0.92840
C   −0.99384   −2.54300   +1.25420
H   −2.66384   −1.18522   +1.30869
H   −1.82310   −1.22455   −0.22010
C   −0.77770   −2.70617   +2.74684
H   −1.59738   −3.37465   +0.88728
H   −0.03038   −2.61370   +0.74885
C   −0.22362   −4.07050   +3.10491
H   −0.09170   −1.93723   +3.10107
H   −1.72233   −2.54471   +3.27050
H   −0.06337   −4.16952   +4.17597
H   −0.90282   −4.86363   +2.79614
H   +0.73058   −4.24427   +2.61048
18
* E =  +0.713  kcal/mol ; (9) 182_063_177_064
H   +0.00000   +0.00000   +0.00000
O   +0.00000   +0.00000   +0.95329
C   −0.89741   +0.00000   +1.27488
C   −0.85683   +0.03875   +2.78113
H   −1.44008   +0.87203   +0.89786
H   −1.42617   −0.89529   +0.93950
C   −0.20804   +1.29982   +3.31957
H   −1.87921   −0.04245   +3.15520
H   −0.32031   −0.84394   +3.12731
C   −0.21347   +1.39899   +4.83595
H   +0.81575   +1.35241   +2.95003
H   −0.72891   +2.16496   +2.90443
C   +0.60028   +0.32096   +5.52659
H   +0.17540   +2.37589   +5.12304
H   −1.24325   +1.36564   +5.19718
H   +0.63127   +0.47983   +6.60216
H   +0.18674   −0.67065   +5.35706
```

```
 H    +1.62643    +0.31542    +5.16204
18
 * E =   +0.730  kcal/mol  ;  (10)  179_055_055_058
 H    +0.00000    +0.00000    +0.00000
 O    +0.00000    +0.00000    +0.95334
 C    -0.89752    +0.00000    +1.27476
 C    -0.85808    -0.02333    +2.78228
 H    -1.42868    +0.89047    +0.92636
 H    -1.44143    -0.87340    +0.90787
 C    -0.04490    +1.11162    +3.38576
 H    -1.88490    +0.00065    +3.14841
 H    -0.43479    -0.97428    +3.10379
 C    -0.47434    +2.50442    +2.95380
 H    -0.10453    +1.03874    +4.47290
 H    +0.99993    +0.96713    +3.11799
 C    -1.92399    +2.82563    +3.26235
 H    +0.16817    +3.23196    +3.44924
 H    -0.28778    +2.62453    +1.88584
 H    -2.16380    +3.85619    +3.01057
 H    -2.60728    +2.18818    +2.70339
 H    -2.13934    +2.68470    +4.32096
18
 * E =   +0.740  kcal/mol  ;  (11)  065_061_187_298
 H    +0.00000    +0.00000    +0.00000
 O    +0.00000    +0.00000    +0.95447
 C    -0.90222    +0.00000    +1.26595
 C    -1.65194    -1.26453    +0.90639
 H    -0.83460    +0.09835    +2.34737
 H    -1.43783    +0.87612    +0.89300
 C    -1.02390    -2.51131    +1.49977
 H    -2.68412    -1.15519    +1.24121
 H    -1.69447    -1.35764    -0.18315
 C    -1.64711    -3.80951    +1.01601
 H    +0.03940    -2.50712    +1.26442
 H    -1.09560    -2.45988    +2.58877
 C    -3.11036    -3.96453    +1.38364
 H    -1.53506    -3.87839    -0.06766
 H    -1.08360    -4.64496    +1.43087
 H    -3.49341    -4.93573    +1.07892
 H    -3.25294    -3.87605    +2.45998
 H    -3.72957    -3.20780    +0.90647
18
 * E =   +0.839  kcal/mol  ;  (12)  181_179_175_063
 H    +0.00000    +0.00000    +0.00000
 O    +0.00000    +0.00000    +0.95348
```

```
C   −0.89766   +0.00000   +1.27492
C   −0.85945   +0.01563   +2.78051
H   −1.43999   +0.87743   +0.91109
H   −1.43367   −0.88787   +0.92766
C   −2.24678   +0.03859   +3.39338
H   −0.31311   −0.86426   +3.12241
H   −0.28296   +0.88441   +3.09438
C   −2.25232   −0.06088   +4.91011
H   −2.76144   +0.95456   +3.09381
H   −2.83216   −0.78696   +2.98453
C   −1.56434   +1.09875   +5.60404
H   −3.28510   −0.12116   +5.25289
H   −1.77814   −0.99725   +5.20832
H   −1.66288   +1.02405   +6.68452
H   −0.50161   +1.13335   +5.37602
H   −1.99888   +2.04955   +5.29775
18
 * E =  +0.844  kcal/mol ; (13) 065_302_302_185
H   +0.00000   +0.00000   +0.00000
O   +0.00000   +0.00000   +0.95456
C   −0.90199   +0.00000   +1.26697
C   −1.65213   −1.26727   +0.91215
H   −0.83912   +0.10193   +2.34685
H   −1.43651   +0.87743   +0.89406
C   −1.70836   −1.56144   −0.57854
H   −1.18239   −2.10566   +1.42499
H   −2.66684   −1.18122   +1.30538
C   −2.33502   −0.46135   −1.41661
H   −0.70238   −1.77273   −0.94913
H   −2.26911   −2.48420   −0.73135
C   −2.48138   −0.85207   −2.87347
H   −3.31266   −0.20728   −1.00342
H   −1.73800   +0.45026   −1.34905
H   −2.92222   −0.05073   −3.46147
H   −1.51499   −1.09280   −3.31358
H   −3.11622   −1.72991   −2.97974
18
 * E =  +0.850  kcal/mol ; (14) 061_177_175_063
H   +0.00000   +0.00000   +0.00000
O   +0.00000   +0.00000   +0.95434
C   −0.90195   +0.00000   +1.26619
C   −1.67940   −1.22198   +0.83184
H   −0.83653   +0.03295   +2.35137
H   −1.42231   +0.90808   +0.94774
C   −3.08608   −1.24397   +1.40030
```

```
H   −1.73078   −1.24700   −0.26045
H   −1.12280   −2.10598   +1.13935
C   −3.94250   −2.39325   +0.89417
H   −3.03193   −1.29058   +2.49019
H   −3.58202   −0.30170   +1.16081
C   −3.41640   −3.76434   +1.27211
H   −4.95190   −2.27655   +1.28796
H   −4.03071   −2.32265   −0.19128
H   −4.09963   −4.54840   +0.95425
H   −2.45169   −3.96828   +0.81323
H   −3.29319   −3.85068   +2.35087
18
 * E =   +0.869  kcal/mol ;  (15) 061_178_184_297
H   +0.00000   +0.00000   +0.00000
O   +0.00000   +0.00000   +0.95436
C   −0.90204   +0.00000   +1.26600
C   −1.68487   −1.21245   +0.81528
H   −0.83633   +0.01965   +2.35141
H   −1.41869   +0.91404   +0.95845
C   −3.10202   −1.21974   +1.35755
H   −1.70266   −1.23471   −0.27632
H   −1.15464   −2.10933   +1.13795
C   −3.89320   −2.47068   +1.01164
H   −3.06480   −1.11463   +2.44302
H   −3.63756   −0.34327   +0.98545
C   −4.11607   −2.66607   −0.47559
H   −3.38108   −3.34254   +1.42146
H   −4.85903   −2.42256   +1.51402
H   −4.75341   −3.52603   −0.66741
H   −4.59769   −1.79372   −0.91579
H   −3.18145   −2.83085   −1.00701
18
 * E =   +0.902  kcal/mol ;  (16) 066_296_187_297
H   +0.00000   +0.00000   +0.00000
O   +0.00000   +0.00000   +0.95398
C   −0.90083   +0.00000   +1.26796
C   −1.65169   −1.26754   +0.92190
H   −0.83380   +0.11363   +2.34644
H   −1.44122   +0.86981   +0.88169
C   −1.82016   −1.47354   −0.57259
H   −1.12472   −2.11178   +1.36450
H   −2.63607   −1.22173   +1.39290
C   −2.71169   −2.64815   −0.94212
H   −2.23579   −0.56284   −1.01023
H   −0.84128   −1.62384   −1.03466
```

```
C  −2.17586  −3.99161  −0.48641
H  −3.70463  −2.48822  −0.51870
H  −2.84214  −2.66297  −2.02396
H  −2.80721  −4.80480  −0.83657
H  −1.17184  −4.16242  −0.87243
H  −2.12738  −4.06107  +0.59778
18
* E =  +0.905  kcal/mol ; (17) 064_061_179_065
H  +0.00000  +0.00000  +0.00000
O  +0.00000  +0.00000  +0.95452
C  −0.90219  +0.00000  +1.26624
C  −1.65719  −1.25543  +0.88720
H  −0.83348  +0.08199  +2.34897
H  −1.43391  +0.88400  +0.90612
C  −1.03567  −2.51489  +1.45954
H  −2.68765  −1.15810  +1.23600
H  −1.71061  −1.31794  −0.20202
C  −1.78645  −3.79112  +1.11750
H  −0.00440  −2.59071  +1.11404
H  −0.98297  −2.41156  +2.54465
C  −1.79395  −4.12481  −0.36233
H  −1.33471  −4.61833  +1.66458
H  −2.81360  −3.71489  +1.47905
H  −2.27324  −5.08342  −0.54708
H  −2.32955  −3.37847  −0.94501
H  −0.77873  −4.18264  −0.75276
18
* E =  +0.910  kcal/mol ; (18) 067_296_179_064
H  +0.00000  +0.00000  +0.00000
O  +0.00000  +0.00000  +0.95375
C  −0.90003  +0.00000  +1.26931
C  −1.64253  −1.28361  +0.96584
H  −0.83146  +0.14521  +2.34389
H  −1.44980  +0.85339  +0.86074
C  −1.82232  −1.53957  −0.51894
H  −1.10210  −2.11619  +1.41841
H  −2.61523  −1.23015  +1.45559
C  −2.54183  −2.83880  −0.84467
H  −2.36885  −0.70458  −0.96575
H  −0.84298  −1.56424  −1.00046
C  −3.97621  −2.88782  −0.35479
H  −2.52887  −2.98480  −1.92449
H  −1.98127  −3.67241  −0.41907
H  −4.46920  −3.80181  −0.67743
H  −4.03614  −2.85381  +0.73058
```

```
H    −4.55040    −2.04802    −0.74414
18
* E =  +0.918  kcal/mol ; (19) 059_182_296_185
H    +0.00000    +0.00000    +0.00000
O    +0.00000    +0.00000    +0.95450
C    −0.90218    +0.00000    +1.26618
C    −1.69035    −1.19378    +0.77334
H    −0.83117    −0.00543    +2.35010
H    −1.41255    +0.92526    +0.97996
C    −3.14311    −1.19385    +1.21815
H    −1.65261    −1.20612    −0.31837
H    −1.19312    −2.10367    +1.11076
C    −3.33898    −1.30054    +2.72028
H    −3.63637    −0.28915    +0.85493
H    −3.65665    −2.02791    +0.73836
C    −4.79897    −1.42651    +3.10618
H    −2.78460    −2.16398    +3.09201
H    −2.91147    −0.42887    +3.21564
H    −4.92365    −1.49761    +4.18403
H    −5.36986    −0.56511    +2.76331
H    −5.24591    −2.31398    +2.66118
18
* E =  +0.923  kcal/mol ; (20) 064_052_054_058
H    +0.00000    +0.00000    +0.00000
O    +0.00000    +0.00000    +0.95453
C    −0.90160    +0.00000    +1.26798
C    −1.66301    −1.24870    +0.87781
H    −0.82859    +0.07573    +2.35049
H    −1.43350    +0.88875    +0.92069
C    −0.95457    −2.53955    +1.26001
H    −2.65035    −1.20127    +1.33963
H    −1.83854    −1.24321    −0.20020
C    −0.56297    −2.63567    +2.72547
H    −1.59976    −3.38054    +1.00123
H    −0.05484    −2.63991    +0.65475
C    −1.73227    −2.50557    +3.68239
H    −0.07105    −3.59434    +2.88783
H    +0.18626    −1.87536    +2.94740
H    −1.41893    −2.66081    +4.71209
H    −2.19116    −1.51951    +3.62913
H    −2.50645    −3.23842    +3.45774
18
* E =  +0.944  kcal/mol ; (21) 177_174_063_174
H    +0.00000    +0.00000    +0.00000
O    +0.00000    +0.00000    +0.95349
```

```
C   -0.89786   +0.00000   +1.27441
C   -0.84965   -0.06720   +2.77970
H   -1.42062   +0.91050   +0.96491
H   -1.44823   -0.85260   +0.87071
C   -2.20909   +0.06945   +3.44209
H   -0.38235   -1.00827   +3.07095
H   -0.18902   +0.72754   +3.12365
C   -3.19248   -1.03699   +3.10289
H   -2.06769   +0.09143   +4.52334
H   -2.64937   +1.03399   +3.17847
C   -4.47963   -0.93229   +3.89582
H   -3.42538   -1.01610   +2.03797
H   -2.72173   -2.00344   +3.29209
H   -5.17578   -1.72701   +3.63818
H   -4.28567   -0.99490   +4.96531
H   -4.97784   +0.01765   +3.70872
18
 * E =  +1.024  kcal/mol ; (22) 062_173_065_175
H   +0.00000   +0.00000   +0.00000
O   +0.00000   +0.00000   +0.95441
C   -0.90215   +0.00000   +1.26590
C   -1.66725   -1.23017   +0.82906
H   -0.83724   +0.02867   +2.35173
H   -1.41460   +0.91085   +0.94870
C   -3.05304   -1.34459   +1.44168
H   -1.75253   -1.23274   -0.26103
H   -1.07194   -2.10235   +1.09663
C   -4.02073   -0.24868   +1.03025
H   -3.47963   -2.30911   +1.16345
H   -2.96699   -1.35864   +2.53053
C   -5.41927   -0.48028   +1.56593
H   -3.65990   +0.71943   +1.37732
H   -4.05071   -0.18963   -0.05943
H   -6.10000   +0.31288   +1.26606
H   -5.82623   -1.42231   +1.20214
H   -5.41891   -0.52210   +2.65385
18
 * E =  +1.069  kcal/mol ; (23) 065_302_305_302
H   +0.00000   +0.00000   +0.00000
O   +0.00000   +0.00000   +0.95466
C   -0.90204   +0.00000   +1.26722
C   -1.65190   -1.26644   +0.91192
H   -0.83954   +0.10486   +2.34684
H   -1.43685   +0.87721   +0.89168
C   -1.70465   -1.55168   -0.58228
```

```
H   −1.17818   −2.10773   +1.41610
H   −2.66242   −1.18855   +1.31503
C   −2.26325   −0.42091   −1.43398
H   −0.70343   −1.80700   −0.93366
H   −2.30612   −2.44680   −0.74447
C   −3.67157   −0.00891   −1.05287
H   −1.60785   +0.45161   −1.37889
H   −2.24766   −0.73331   −2.47769
H   −4.06205   +0.74108   −1.73640
H   −4.34668   −0.86323   −1.07646
H   −3.71093   +0.41215   −0.05010
18
 * E =  +1.219  kcal/mol ; (24) 178_174_057_056
H   +0.00000   +0.00000   +0.00000
O   +0.00000   +0.00000   +0.95350
C   −0.89793   +0.00000   +1.27427
C   −0.85209   −0.04578   +2.77956
H   −1.42753   +0.90081   +0.94867
H   −1.44359   −0.86352   +0.88467
C   −2.21818   +0.08868   +3.43183
H   −0.36848   −0.97351   +3.08365
H   −0.20461   +0.76146   +3.11908
C   −3.24407   −0.95159   +3.00420
H   −2.08968   +0.03320   +4.51343
H   −2.61714   +1.08324   +3.22580
C   −2.79238   −2.38159   +3.22678
H   −4.16620   −0.77407   +3.55723
H   −3.49935   −0.81070   +1.95291
H   −3.58469   −3.08708   +2.98792
H   −1.93350   −2.63387   +2.60734
H   −2.50541   −2.54411   +4.26484
18
 * E =  +1.233  kcal/mol ; (25) 059_182_302_304
H   +0.00000   +0.00000   +0.00000
O   +0.00000   +0.00000   +0.95446
C   −0.90210   +0.00000   +1.26626
C   −1.69250   −1.19053   +0.77194
H   −0.83102   −0.01217   +2.35109
H   −1.41067   +0.92849   +0.98753
C   −3.14476   −1.18447   +1.22333
H   −1.66468   −1.20160   −0.31994
H   −1.19041   −2.10080   +1.09827
C   −3.34676   −1.13958   +2.73152
H   −3.65637   −0.33241   +0.77279
H   −3.63420   −2.07523   +0.82777
```

```
C  −2.65575  −2.26676  +3.47351
H  −3.00300  −0.18172  +3.12363
H  −4.41655  −1.17485  +2.93576
H  −2.89801  −2.24647  +4.53330
H  −2.95929  −3.23701  +3.08244
H  −1.57264  −2.20255  +3.38650
18
* E = +1.375 kcal/mol ; (26) 061_172_057_056
H  +0.00000  +0.00000  +0.00000
O  +0.00000  +0.00000  +0.95444
C  −0.90212  +0.00000  +1.26610
C  −1.67641  −1.21642  +0.81047
H  −0.83760  +0.01541  +2.35227
H  −1.41116  +0.91817  +0.96086
C  −3.05384  −1.33660  +1.44390
H  −1.76811  −1.19647  −0.27807
H  −1.08716  −2.10011  +1.05200
C  −3.97517  −0.14490  +1.22264
H  −3.53692  −2.23324  +1.05377
H  −2.93692  −1.49845  +2.51643
C  −4.19125  +0.19710  −0.23845
H  −4.93632  −0.36314  +1.68733
H  −3.58550  +0.72874  +1.74614
H  −4.92458  +0.99167  −0.35304
H  −3.27134  +0.53433  −0.71335
H  −4.54952  −0.66792  −0.79510
18
* E = +1.474 kcal/mol ; (27) 179_077_298_302
H  +0.00000  +0.00000  +0.00000
O  +0.00000  +0.00000  +0.95358
C  −0.89826  +0.00000  +1.27363
C  −0.88217  −0.02281  +2.78275
H  −1.43322  +0.88754  +0.92134
H  −1.42911  −0.87888  +0.90072
C  −0.54807  +1.30344  +3.44923
H  −1.86963  −0.33749  +3.12111
H  −0.18715  −0.80104  +3.09824
C  +0.82480  +1.88249  +3.14265
H  −1.31185  +2.03280  +3.17196
H  −0.63744  +1.17030  +4.52916
C  +1.96843  +0.96334  +3.52176
H  +0.89502  +2.11896  +2.08235
H  +0.92063  +2.82587  +3.68149
H  +2.92861  +1.45589  +3.38463
H  +1.90006  +0.65831  +4.56603
```

```
 H    +1.96734    +0.06958    +2.90332
18
 * E =   +1.499  kcal/mol  ;  (28) 179_068_284_183
 H    +0.00000    +0.00000    +0.00000
 O    +0.00000    +0.00000    +0.95357
 C    −0.89842    +0.00000    +1.27316
 C    −0.89961    −0.03524    +2.78124
 H    −1.42740    +0.89399    +0.92919
 H    −1.43578    −0.87203    +0.89248
 C    −0.37549    +1.21807    +3.46571
 H    −1.93104    −0.20504    +3.09192
 H    −0.32875    −0.90562    +3.10774
 C    +1.13367    +1.38576    +3.44960
 H    −0.84310    +2.09580    +3.01197
 H    −0.70897    +1.20786    +4.50529
 C    +1.57923    +2.61934    +4.20881
 H    +1.59102    +0.49863    +3.89078
 H    +1.48596    +1.42559    +2.42231
 H    +2.66177    +2.72470    +4.20021
 H    +1.15750    +3.52297    +3.77059
 H    +1.25893    +2.58113    +5.24936
18
 * E =   +1.538  kcal/mol  ;  (29) 062_068_290_185
 H    +0.00000    +0.00000    +0.00000
 O    +0.00000    +0.00000    +0.95446
 C    −0.90214    +0.00000    +1.26612
 C    −1.70550    −1.21498    +0.85131
 H    −0.83337    +0.04578    +2.35120
 H    −1.41550    +0.90725    +0.93730
 C    −1.27895    −2.53248    +1.47794
 H    −2.74543    −1.01755    +1.11617
 H    −1.68744    −1.30129    −0.23902
 C    +0.05218    −3.08438    +0.99919
 H    −1.25033    −2.41578    +2.56396
 H    −2.05368    −3.27539    +1.27816
 C    +0.35015    −4.44878    +1.58778
 H    +0.03563    −3.15351    −0.09077
 H    +0.84808    −2.38683    +1.24785
 H    +1.30344    −4.83643    +1.23602
 H    +0.39469    −4.40369    +2.67493
 H    −0.42031    −5.17120    +1.32156
18
 * E =   +1.921  kcal/mol  ;  (30) 064_275_056_172
 H    +0.00000    +0.00000    +0.00000
 O    +0.00000    +0.00000    +0.95331
```

```
C   −0.89791   +0.00000   +1.27355
C   −1.68220   −1.24382   +0.89816
H   −0.80864   +0.06749   +2.35412
H   −1.42970   +0.89676   +0.93852
C   −2.32515   −1.21708   −0.47942
H   −1.00863   −2.09725   +0.98591
H   −2.46830   −1.39228   +1.63904
C   −1.37687   −0.96734   −1.63931
H   −2.83335   −2.16835   −0.64477
H   −3.10599   −0.45357   −0.49669
C   −2.04810   −1.14736   −2.98633
H   −0.98805   +0.05260   −1.58265
H   −0.52107   −1.64118   −1.56380
H   −1.36448   −0.94288   −3.80665
H   −2.41396   −2.16563   −3.10411
H   −2.90044   −0.47808   −3.08930
18
 * E =  +1.932  kcal/mol ; (31) 067_073_298_299
H   +0.00000   +0.00000   +0.00000
O   +0.00000   +0.00000   +0.95410
C   −0.90181   +0.00000   +1.26562
C   −1.65878   −1.27432   +0.95222
H   −0.83745   +0.13976   +2.34329
H   −1.43951   +0.86102   +0.86148
C   −1.26379   −2.48068   +1.79069
H   −2.72038   −1.07801   +1.10967
H   −1.55476   −1.49563   −0.11267
C   +0.17921   −2.94467   +1.66150
H   −1.46796   −2.24984   +2.83786
H   −1.92424   −3.31038   +1.53107
C   +0.55647   −3.37164   +0.25723
H   +0.85048   −2.15283   +1.98776
H   +0.32629   −3.78408   +2.34163
H   +1.56388   −3.78006   +0.22754
H   −0.12273   −4.13517   −0.12126
H   +0.52937   −2.53144   −0.43342
18
 * E =  +2.238  kcal/mol ; (32) 071_278_060_057
H   +0.00000   +0.00000   +0.00000
O   +0.00000   +0.00000   +0.95300
C   −0.89734   +0.00000   +1.27393
C   −1.63223   −1.30840   +1.05539
H   −0.81319   +0.19621   +2.33938
H   −1.46431   +0.82898   +0.83870
C   −2.23148   −1.50640   −0.32956
```

```
 H   −0.95067   −2.12185   +1.30447
 H   −2.44568   −1.36015   +1.78017
 C   −1.25776   −1.49847   −1.49898
 H   −2.76652   −2.45736   −0.33759
 H   −2.98600   −0.73564   −0.49651
 C   −0.15625   −2.53544   −1.39167
 H   −1.82431   −1.67063   −2.41408
 H   −0.82955   −0.50053   −1.62488
 H   +0.46907   −2.54063   −2.28142
 H   +0.48721   −2.35433   −0.53357
 H   −0.57871   −3.53293   −1.28009
18
 * E =  +2.316  kcal/mol ; (33) 291_060_268_177
 H   +0.00000   +0.00000   +0.00000
 O   +0.00000   +0.00000   +0.95413
 C   −0.90145   +0.00000   +1.26678
 C   −1.64125   +1.29559   +1.00895
 H   −1.45639   −0.83144   +0.82183
 H   −0.84184   −0.18803   +2.33560
 C   −1.74617   +1.67006   −0.46630
 H   −2.63763   +1.18556   +1.43795
 H   −1.15265   +2.10147   +1.55764
 C   −0.62600   +2.56881   −0.96491
 H   −1.78537   +0.75975   −1.07370
 H   −2.69327   +2.17784   −0.65035
 C   −0.73197   +2.86737   −2.44627
 H   −0.65380   +3.50148   −0.40001
 H   +0.34299   +2.12373   −0.74105
 H   +0.06336   +3.53001   −2.77894
 H   −0.67082   +1.95503   −3.03790
 H   −1.68152   +3.34527   −2.68224
18
 * E =  +2.436  kcal/mol ; (34) 177_059_059_264
 H   +0.00000   +0.00000   +0.00000
 O   +0.00000   +0.00000   +0.95347
 C   −0.89722   +0.00000   +1.27613
 C   −0.85945   −0.08198   +2.78068
 H   −1.42089   +0.90816   +0.96319
 H   −1.45113   −0.85323   +0.87816
 C   −0.10399   +1.05211   +3.45678
 H   −1.88971   −0.09889   +3.14039
 H   −0.41008   −1.03432   +3.06096
 C   −0.65653   +2.44567   +3.16318
 H   −0.12584   +0.86640   +4.52991
 H   +0.94294   +1.00639   +3.15760
```

```
C    +0.05622    +3.16138    +2.02964
H    -1.72430    +2.37415    +2.94305
H    -0.58280    +3.06171    +4.05876
H    -0.40216    +4.12523    +1.81726
H    +1.09834    +3.33948    +2.28880
H    +0.05576    +2.57303    +1.11588
18
* E =   +2.452  kcal/mol ; (35) 063_056_058_264
H    +0.00000    +0.00000    +0.00000
O    +0.00000    +0.00000    +0.95448
C    -0.90117    +0.00000    +1.26901
C    -1.67171    -1.23779    +0.86675
H    -0.83175    +0.06541    +2.35261
H    -1.42828    +0.89472    +0.92944
C    -1.03916    -2.54960    +1.30596
H    -2.67559    -1.15835    +1.28894
H    -1.80017    -1.24178    -0.21841
C    -0.83177    -2.67311    +2.81447
H    -1.67610    -3.35902    +0.95141
H    -0.07972    -2.67231    +0.80245
C    +0.56553    -2.29291    +3.27174
H    -1.57241    -2.05969    +3.33284
H    -1.03134    -3.69789    +3.12555
H    +0.65210    -2.33244    +4.35569
H    +1.29971    -2.98153    +2.85689
H    +0.84614    -1.29663    +2.94051
18
* E =   +2.453  kcal/mol ; (36) 061_301_301_090
H    +0.00000    +0.00000    +0.00000
O    +0.00000    +0.00000    +0.95500
C    -0.90288    +0.00000    +1.26618
C    -1.68650    -1.21501    +0.82036
H    -0.83877    +0.01955    +2.35077
H    -1.41835    +0.91578    +0.96276
C    -1.79114    -1.40261    -0.68710
H    -1.22765    -2.10203    +1.25618
H    -2.69171    -1.13934    +1.23922
C    -2.43866    -0.25035    -1.45114
H    -0.80166    -1.60457    -1.10544
H    -2.36240    -2.31329    -0.86131
C    -1.47202    +0.81818    -1.93259
H    -2.95302    -0.65262    -2.32292
H    -3.21381    +0.20528    -0.83214
H    -1.98357    +1.56813    -2.53176
H    -0.98608    +1.34758    -1.11562
```

```
 H   −0.69540   +0.37995   −2.55896
18
 * E =  +2.631  kcal/mol ; (37) 063_061_098_300
 H   +0.00000   +0.00000   +0.00000
 O   +0.00000   +0.00000   +0.95454
 C   −0.90164   +0.00000   +1.26791
 C   −1.65706   −1.24999   +0.86326
 H   −0.82727   +0.07246   +2.34911
 H   −1.43121   +0.88892   +0.91638
 C   −1.06031   −2.53898   +1.42185
 H   −2.69895   −1.14778   +1.17538
 H   −1.67936   −1.28831   −0.22786
 C   −1.73963   −3.05123   +2.68251
 H   −1.11311   −3.32242   +0.66715
 H   +0.00157   −2.37549   +1.61169
 C   −1.70979   −2.08723   +3.85252
 H   −2.77686   −3.30030   +2.45000
 H   −1.26406   −3.98638   +2.97818
 H   −2.14696   −2.53646   +4.74136
 H   −0.68933   −1.79428   +4.09527
 H   −2.27369   −1.18040   +3.64054
18
 * E =  +2.632  kcal/mol ; (38) 185_065_098_300
 H   +0.00000   +0.00000   +0.00000
 O   +0.00000   +0.00000   +0.95332
 C   −0.89789   +0.00000   +1.27364
 C   −0.84960   +0.12425   +2.77736
 H   −1.45633   +0.83140   +0.83891
 H   −1.40336   −0.92797   +0.99550
 C   −0.28279   +1.45376   +3.26914
 H   −1.85501   −0.02859   +3.17435
 H   −0.24302   −0.70248   +3.14404
 C   −1.33351   +2.48106   +3.66080
 H   +0.35578   +1.27606   +4.13296
 H   +0.37024   +1.86122   +2.49635
 C   −2.27589   +2.88073   +2.54205
 H   −1.91712   +2.09000   +4.49655
 H   −0.82849   +3.37105   +4.03656
 H   −2.95920   +3.66314   +2.86470
 H   −1.72575   +3.25531   +1.67963
 H   −2.88269   +2.04042   +2.20896
18
 * E =  +2.666  kcal/mol ; (39) 060_176_261_059
 H   +0.00000   +0.00000   +0.00000
 O   +0.00000   +0.00000   +0.95448
```

C −0.90177 +0.00000 +1.26729
C −1.68895 −1.20830 +0.80526
H −0.83689 +0.02317 +2.35163
H −1.42026 +0.91285 +0.95887
C −3.09848 −1.26387 +1.39174
H −1.73792 −1.18583 −0.28487
H −1.13176 −2.10889 +1.06734
C −3.24204 −2.19264 +2.58770
H −3.41046 −0.25659 +1.68021
H −3.80266 −1.58204 +0.62492
C −2.35175 −1.84073 +3.76281
H −4.28319 −2.18915 +2.91029
H −3.02708 −3.21325 +2.26705
H −2.53563 −2.50269 +4.60581
H −1.29742 −1.92744 +3.50678
H −2.52930 −0.81998 +4.09950
18
* E = +2.677 kcal/mol ; (40) 181_182_100_300
H +0.00000 +0.00000 +0.00000
O +0.00000 +0.00000 +0.95351
C −0.89777 +0.00000 +1.27475
C −0.85788 +0.01866 +2.78224
H −1.43757 +0.88210 +0.91821
H −1.43808 −0.88120 +0.92056
C −2.24626 −0.04093 +3.41586
H −0.24743 −0.81886 +3.12253
H −0.33131 +0.92169 +3.08520
C −2.64915 −1.42004 +3.91502
H −2.29457 +0.65315 +4.25312
H −2.99133 +0.31134 +2.69747
C −2.68021 −2.48793 +2.83990
H −1.95720 −1.72368 +4.70219
H −3.63091 −1.35155 +4.38374
H −3.01968 −3.43996 +3.24147
H −3.35463 −2.21122 +2.03014
H −1.69342 −2.65023 +2.40996
18
* E = +2.729 kcal/mol ; (41) 062_179_100_300
H +0.00000 +0.00000 +0.00000
O +0.00000 +0.00000 +0.95438
C −0.90162 +0.00000 +1.26730
C −1.67411 −1.23148 +0.84380
H −0.83487 +0.03104 +2.35252
H −1.42317 +0.90788 +0.95379
C −3.11027 −1.24590 +1.36566

```
 H   −1.67942   −1.29758   −0.24797
 H   −1.12378   −2.10147   +1.19749
 C   −4.15618   −0.82505   +0.34484
 H   −3.35906   −2.24489   +1.71928
 H   −3.18346   −0.59543   +2.24101
 C   −3.97154   +0.57938   −0.19420
 H   −4.14310   −1.53396   −0.48477
 H   −5.14352   −0.90861   +0.79877
 H   −4.77239   +0.84626   −0.87981
 H   −3.96625   +1.31262   +0.61126
 H   −3.03359   +0.67944   −0.73797
18
 * E =   +2.953  kcal/mol ;  (42)  292_060_267_287
 H   +0.00000   +0.00000   +0.00000
 O   +0.00000   +0.00000   +0.95432
 C   −0.90213   +0.00000   +1.26559
 C   −1.64715   +1.28588   +0.97598
 H   −1.45040   −0.84445   +0.83688
 H   −0.84309   −0.16491   +2.33819
 C   −1.74077   +1.61495   −0.51218
 H   −2.64544   +1.18381   +1.40209
 H   −1.16451   +2.10309   +1.51160
 C   −0.63174   +2.52286   −1.03120
 H   −1.75235   +0.68156   −1.08215
 H   −2.69685   +2.09402   −0.72520
 C   −0.78472   +3.96196   −0.57770
 H   +0.33828   +2.14796   −0.70516
 H   −0.62838   +2.49394   −2.12085
 H   +0.02152   +4.58394   −0.95908
 H   −1.72494   +4.38241   −0.93136
 H   −0.77435   +4.04558   +0.50704
18
 * E =   +2.960  kcal/mol ;  (43)  177_174_059_266
 H   +0.00000   +0.00000   +0.00000
 O   +0.00000   +0.00000   +0.95350
 C   −0.89770   +0.00000   +1.27491
 C   −0.84309   −0.07151   +2.77906
 H   −1.42260   +0.90892   +0.96420
 H   −1.44681   −0.85579   +0.87454
 C   −2.18846   +0.06298   +3.47368
 H   −0.37656   −1.01666   +3.05841
 H   −0.17397   +0.71696   +3.12188
 C   −3.22453   −0.99257   +3.09174
 H   −2.00561   +0.01918   +4.54620
 H   −2.60165   +1.05610   +3.28293
```

```
C   -4.15393   -0.56989   +1.96822
H   -2.71333   -1.91916   +2.82292
H   -3.83285   -1.23260   +3.96290
H   -4.85817   -1.36035   +1.71776
H   -4.73141   +0.30610   +2.25879
H   -3.61261   -0.31306   +1.06060
18
 * E =  +2.988  kcal/mol ; (44) 058_182_301_095
H   +0.00000   +0.00000   +0.00000
O   +0.00000   +0.00000   +0.95453
C   -0.90234   +0.00000   +1.26585
C   -1.68752   -1.19011   +0.76260
H   -0.83131   -0.02073   +2.35013
H   -1.40969   +0.93032   +0.99076
C   -3.14744   -1.21710   +1.18878
H   -1.63943   -1.20062   -0.32906
H   -1.18687   -2.09758   +1.10181
C   -3.37443   -1.23387   +2.69956
H   -3.67059   -0.36090   +0.75693
H   -3.60307   -2.09970   +0.74258
C   -3.59705   +0.13941   +3.30716
H   -4.24272   -1.85116   +2.92613
H   -2.52829   -1.72290   +3.18619
H   -3.73362   +0.07768   +4.38454
H   -2.76414   +0.81296   +3.12007
H   -4.48860   +0.60287   +2.88809
18
 * E =  +3.088  kcal/mol ; (45) 064_290_261_060
H   +0.00000   +0.00000   +0.00000
O   +0.00000   +0.00000   +0.95431
C   -0.90089   +0.00000   +1.26912
C   -1.65652   -1.25398   +0.87761
H   -0.82777   +0.06586   +2.35133
H   -1.42501   +0.89322   +0.92166
C   -1.96152   -1.36856   -0.61560
H   -1.05871   -2.10234   +1.20699
H   -2.58960   -1.29410   +1.44341
C   -3.37314   -0.95650   -1.00370
H   -1.25008   -0.76793   -1.19101
H   -1.80153   -2.39391   -0.94442
C   -3.72107   +0.48184   -0.67493
H   -3.50606   -1.12278   -2.07264
H   -4.08008   -1.62097   -0.50399
H   -4.71852   +0.73387   -1.02726
H   -3.70071   +0.66534   +0.39763
```

```
 H    −3.02095    +1.17360    −1.14207
18
 * E =   +3.116  kcal/mol  ;  (46)  299_188_301_094
 H    +0.00000    +0.00000    +0.00000
 O    +0.00000    +0.00000    +0.95451
 C    −0.90220    +0.00000    +1.26616
 C    −1.67066    +1.21725    +0.80306
 H    −1.41053    −0.91704    +0.95857
 H    −0.83827    −0.01361    +2.35244
 C    −3.05431    +1.37600    +1.41472
 H    −1.06827    +2.09608    +1.03141
 H    −1.76658    +1.17984    −0.28569
 C    −4.01672    +0.21816    +1.15522
 H    −2.95802    +1.52798    +2.49194
 H    −3.48119    +2.29769    +1.02225
 C    −4.03880    −0.82793    +2.25509
 H    −5.02651    +0.60939    +1.03803
 H    −3.76913    −0.25127    +0.20084
 H    −4.72426    −1.63826    +2.01667
 H    −3.05871    −1.26657    +2.42639
 H    −4.36503    −0.38666    +3.19531
18
 * E =   +3.417  kcal/mol  ;  (47)  179_078_301_092
 H    +0.00000    +0.00000    +0.00000
 O    +0.00000    +0.00000    +0.95368
 C    −0.89761    +0.00000    +1.27587
 C    −0.87281    −0.03719    +2.78487
 H    −1.43489    +0.88904    +0.93048
 H    −1.42953    −0.87685    +0.90008
 C    −0.56215    +1.27126    +3.49641
 H    −1.84727    −0.38884    +3.12508
 H    −0.14869    −0.79879    +3.07724
 C    +0.78026    +1.92061    +3.16811
 H    −1.36327    +1.98702    +3.29556
 H    −0.61008    +1.06885    +4.56631
 C    +0.71519    +2.92087    +2.02966
 H    +1.14826    +2.43482    +4.05592
 H    +1.50899    +1.14640    +2.93158
 H    +1.69116    +3.36182    +1.83724
 H    +0.38337    +2.45049    +1.10841
 H    +0.02732    +3.73186    +2.26644
18
 * E =   +3.568  kcal/mol  ;  (48)  296_284_059_270
 H    +0.00000    +0.00000    +0.00000
 O    +0.00000    +0.00000    +0.95416
```

C	−0.90209	+0.00000	+1.26504
C	−1.67470	+1.25017	+0.89673
H	−1.42663	−0.88150	+0.88889
H	−0.84107	−0.10160	+2.34717
C	−1.36836	+2.50749	+1.69668
H	−1.52094	+1.44755	−0.16796
H	−2.73736	+1.02750	+1.00560
C	+0.07121	+3.01384	+1.65956
H	−2.03071	+3.28986	+1.32611
H	−1.65869	+2.34350	+2.73719
C	+0.96261	+2.43645	+2.74287
H	+0.50559	+2.80808	+0.68074
H	+0.05886	+4.09862	+1.76545
H	+1.96099	+2.86667	+2.69602
H	+0.55729	+2.65548	+3.73011
H	+1.06812	+1.36073	+2.64235

3.4 Conformers of n-hexanol

```
21
 * E =   +0.000  kcal/mol ; (1) 182_062_181_180_180
 H    +0.00000    +0.00000    +0.00000
 O    +0.00000    +0.00000    +0.95330
 C    -0.89730    +0.00000    +1.27523
 C    -0.85705    +0.04486    +2.78117
 H    -1.44163    +0.86930    +0.89460
 H    -1.42474    -0.89746    +0.94376
 C    -0.20937    +1.30383    +3.32266
 H    -1.87820    -0.04154    +3.15591
 H    -0.31447    -0.83182    +3.13700
 C    -0.14055    +1.33101    +4.83659
 H    +0.79313    +1.39047    +2.90504
 H    -0.76663    +2.17512    +2.96977
 C    +0.49865    +2.59115    +5.38704
 H    -1.14677    +1.22850    +5.25055
 H    +0.41977    +0.46153    +5.18777
 C    +0.56715    +2.60675    +6.90068
 H    +1.50299    +2.69243    +4.97325
 H    -0.06147    +3.45967    +5.03680
 H    +1.02845    +3.51890    +7.27224
 H    -0.42742    +2.53685    +7.33841
 H    +1.14895    +1.76576    +7.27450
21
 * E =   +0.225  kcal/mol ; (2) 065_061_183_181_180
 H    +0.00000    +0.00000    +0.00000
 O    +0.00000    +0.00000    +0.95448
 C    -0.90205    +0.00000    +1.26648
 C    -1.65231    -1.26239    +0.90264
 H    -0.83349    +0.09448    +2.34815
 H    -1.43738    +0.87782    +0.89695
 C    -1.03064    -2.51652    +1.48371
 H    -2.68428    -1.16314    +1.24477
 H    -1.70290    -1.34727    -0.18661
 C    -1.74148    -3.78696    +1.06155
 H    +0.01755    -2.56288    +1.18824
 H    -1.03454    -2.44355    +2.57354
 C    -1.13048    -5.04278    +1.65249
 H    -2.79400    -3.72970    +1.34954
 H    -1.73181    -3.86207    -0.02848
 C    -1.84499    -6.30726    +1.22064
 H    -0.07948    -5.09864    +1.36527
 H    -1.14233    -4.96879    +2.74099
 H    -1.39023    -7.19368    +1.65669
```

```
 H    −2.89074    −6.28895    +1.52334
 H    −1.81958    −6.42098    +0.13806
21
 * E =  +0.237  kcal/mol ; (3) 180_180_180_180_180
 H    +0.00000    +0.00000    +0.00000
 O    +0.00000    +0.00000    +0.95350
 C    −0.89787    +0.00000    +1.27443
 C    −0.86021    −0.00084    +2.77995
 H    −1.43655    +0.88301    +0.91905
 H    −1.43693    −0.88237    +0.91802
 C    −2.24325    −0.00060    +3.40020
 H    −0.29983    −0.87533    +3.11090
 H    −0.29914    +0.87285    +3.11181
 C    −2.21409    −0.00145    +4.91612
 H    −2.80003    +0.87288    +3.05332
 H    −2.80078    −0.87322    +3.05235
 C    −3.59371    −0.00140    +5.54508
 H    −1.65831    −0.87413    +5.26584
 H    −1.65780    +0.87052    +5.26680
 C    −3.55181    −0.00234    +7.05969
 H    −4.14771    +0.87094    +5.19459
 H    −4.14827    −0.87296    +5.19352
 H    −4.55121    −0.00235    +7.48850
 H    −3.03005    −0.88066    +7.43609
 H    −3.02961    +0.87526    +7.43715
21
 * E =  +0.244  kcal/mol ; (4) 061_177_180_180_180
 H    +0.00000    +0.00000    +0.00000
 O    +0.00000    +0.00000    +0.95437
 C    −0.90198    +0.00000    +1.26623
 C    −1.68505    −1.21200    +0.81522
 H    −0.83613    +0.01820    +2.35164
 H    −1.41839    +0.91455    +0.95999
 C    −3.09602    −1.23762    +1.36986
 H    −1.72468    −1.22860    −0.27714
 H    −1.14437    −2.10808    +1.12044
 C    −3.89416    −2.44539    +0.91890
 H    −3.05545    −1.22105    +2.46104
 H    −3.62227    −0.32781    +1.07258
 C    −5.30491    −2.47576    +1.47411
 H    −3.93637    −2.46404    −0.17244
 H    −3.36998    −3.35614    +1.21625
 C    −6.09293    −3.68644    +1.01666
 H    −5.26157    −2.45747    +2.56417
 H    −5.82713    −1.56499    +1.17684
```

```
 H    -7.10007   -3.68729   +1.42677
 H    -6.17748   -3.71131   -0.06852
 H    -5.60779   -4.60981   +1.32861
21
 * E =  +0.252  kcal/mol ; (5) 067_296_183_180_180
 H    +0.00000   +0.00000   +0.00000
 O    +0.00000   +0.00000   +0.95388
 C    -0.90034   +0.00000   +1.26897
 C    -1.64653   -1.27600   +0.94556
 H    -0.83248   +0.13005   +2.34556
 H    -1.44584   +0.86139   +0.87146
 C    -1.82419   -1.51698   -0.54130
 H    -1.11485   -2.11607   +1.39338
 H    -2.62672   -1.22916   +1.42379
 C    -2.62791   -2.76445   -0.85301
 H    -2.31383   -0.65051   -0.99376
 H    -0.84629   -1.60661   -1.02036
 C    -2.80013   -3.01638   -2.33831
 H    -2.14304   -3.62860   -0.39402
 H    -3.61121   -2.68546   -0.38411
 C    -3.60828   -4.26327   -2.63505
 H    -3.28146   -2.15053   -2.79581
 H    -1.81700   -3.09789   -2.80461
 H    -3.71854   -4.42396   -3.70486
 H    -3.13193   -5.14748   -2.21491
 H    -4.60658   -4.19396   -2.20620
21
 * E =  +0.457  kcal/mol ; (6) 180_055_059_174_179
 H    +0.00000   +0.00000   +0.00000
 O    +0.00000   +0.00000   +0.95330
 C    -0.89731   +0.00000   +1.27519
 C    -0.85553   -0.00509   +2.78359
 H    -1.43298   +0.88240   +0.91617
 H    -1.43673   -0.88124   +0.92051
 C    -0.04156   +1.13321   +3.37539
 H    -1.88266   +0.03714   +3.14979
 H    -0.43945   -0.95595   +3.11487
 C    -0.52885   +2.51587   +2.98752
 H    -0.05448   +1.04056   +4.46245
 H    +0.99607   +1.01966   +3.06403
 C    +0.21464   +3.62735   +3.70328
 H    -0.41589   +2.65434   +1.91076
 H    -1.59801   +2.60368   +3.19955
 C    -0.25478   +5.01007   +3.29871
 H    +0.09755   +3.50129   +4.78084
```

```
 H    +1.28220   +3.52876   +3.50139
 H    +0.29052   +5.78954   +3.82599
 H    −0.11566   +5.17345   +2.23124
 H    −1.31369   +5.14299   +3.51510
21
 * E =  +0.598  kcal/mol ; (7) 182_063_186_297_184
 H    +0.00000   +0.00000   +0.00000
 O    +0.00000   +0.00000   +0.95328
 C    −0.89723   +0.00000   +1.27534
 C    −0.85877   +0.04912   +2.78160
 H    −1.44272   +0.86756   +0.89225
 H    −1.42339   −0.89901   +0.94624
 C    −0.21504   +1.31658   +3.31232
 H    −1.88051   −0.04832   +3.14833
 H    −0.30750   −0.82162   +3.14038
 C    −0.03368   +1.33276   +4.82017
 H    +0.75336   +1.43460   +2.82967
 H    −0.81822   +2.17708   +3.01169
 C    −1.32757   +1.28364   +5.61205
 H    +0.59981   +0.49316   +5.11577
 H    +0.51328   +2.23471   +5.10010
 C    −1.10209   +1.40942   +7.10548
 H    −1.98535   +2.08592   +5.27163
 H    −1.85361   +0.35124   +5.40635
 H    −2.03780   +1.36890   +7.65841
 H    −0.46554   +0.60610   +7.47302
 H    −0.61317   +2.35122   +7.34936
21
 * E =  +0.632  kcal/mol ; (8) 062_053_058_174_179
 H    +0.00000   +0.00000   +0.00000
 O    +0.00000   +0.00000   +0.95457
 C    −0.90138   +0.00000   +1.26876
 C    −1.67323   −1.23489   +0.85296
 H    −0.82894   +0.05648   +2.35170
 H    −1.42793   +0.89786   +0.93639
 C    −0.99111   −2.54197   +1.22007
 H    −2.66165   −1.18656   +1.31420
 H    −1.84114   −1.20549   −0.22611
 C    −0.72179   −2.70553   +2.70344
 H    −1.61212   −3.36932   +0.87338
 H    −0.04680   −2.61731   +0.68068
 C    −0.17820   −4.07642   +3.05773
 H    −0.00797   −1.94796   +3.03008
 H    −1.64238   −2.52698   +3.26572
 C    +0.10941   −4.23356   +4.53705
```

```
 H    −0.89101    −4.83910    +2.73974
 H    +0.73354    −4.25653    +2.48623
 H    +0.49643    −5.22349    +4.76749
 H    +0.84519    −3.50428    +4.87167
 H    −0.79226    −4.08482    +5.12913
21
 * E =  +0.640  kcal/mol ; (9) 181_062_181_176_064
 H    +0.00000    +0.00000    +0.00000
 O    +0.00000    +0.00000    +0.95330
 C    −0.89730    +0.00000    +1.27523
 C    −0.85711    +0.02993    +2.78142
 H    −1.43851    +0.87487    +0.90299
 H    −1.42792    −0.89233    +0.93505
 C    −0.19945    +1.27845    +3.33573
 H    −1.87927    −0.05099    +3.15491
 H    −0.32255    −0.85483    +3.12939
 C    −0.14737    +1.28687    +4.85185
 H    +0.80994    +1.35343    +2.93133
 H    −0.74349    +2.15320    +2.97498
 C    +0.58779    +2.47723    +5.44540
 H    −1.16495    +1.25878    +5.24994
 H    +0.33543    +0.36870    +5.19070
 C    −0.06501    +3.81397    +5.14970
 H    +0.65603    +2.34515    +6.52527
 H    +1.61414    +2.48356    +5.07491
 H    +0.45195    +4.62545    +5.65691
 H    −0.05898    +4.04002    +4.08586
 H    −1.10206    +3.82336    +5.48341
21
 * E =  +0.641  kcal/mol ; (10) 181_062_181_184_297
 H    +0.00000    +0.00000    +0.00000
 O    +0.00000    +0.00000    +0.95329
 C    −0.89727    +0.00000    +1.27526
 C    −0.85739    +0.03475    +2.78149
 H    −1.43949    +0.87318    +0.90046
 H    −1.42692    −0.89391    +0.93777
 C    −0.20567    +1.28805    +3.33181
 H    −1.87888    −0.05132    +3.15549
 H    −0.31736    −0.84579    +3.13182
 C    −0.14055    +1.29600    +4.84740
 H    +0.79229    +1.37207    +2.90612
 H    −0.76398    +2.16388    +2.99035
 C    +0.41702    +2.57918    +5.44116
 H    −1.14255    +1.12734    +5.24678
 H    +0.46578    +0.45182    +5.18413
```

```
 C    +1.86022    +2.85447    +5.06464
 H    −0.20719    +3.41842    +5.12897
 H    +0.33486    +2.52884    +6.52697
 H    +2.23556    +3.74288    +5.56757
 H    +2.50138    +2.01975    +5.34496
 H    +1.97581    +3.01191    +3.99487
21
 * E =  +0.735  kcal/mol ; (11) 182_062_177_064_175
 H    +0.00000    +0.00000    +0.00000
 O    +0.00000    +0.00000    +0.95330
 C    −0.89726    +0.00000    +1.27534
 C    −0.85590    +0.03793    +2.78164
 H    −1.43992    +0.87243    +0.89915
 H    −1.42646    −0.89494    +0.93971
 C    −0.20427    +1.29778    +3.32012
 H    −1.87833    −0.04102    +3.15606
 H    −0.32141    −0.84606    +3.12752
 C    −0.22070    +1.40479    +4.83549
 H    +0.82197    +1.34475    +2.95706
 H    −0.71802    +2.16346    +2.89743
 C    +0.58429    +0.33479    +5.55061
 H    +0.16859    +2.38291    +5.12343
 H    −1.25324    +1.37488    +5.19283
 C    +0.62847    +0.54608    +7.05073
 H    +0.16593    −0.64906    +5.33895
 H    +1.59940    +0.32546    +5.15007
 H    +1.20531    −0.22988    +7.54871
 H    +1.08058    +1.50534    +7.29782
 H    −0.37378    +0.53775    +7.47654
21
 * E =  +0.760  kcal/mol ; (12) 065_061_188_297_184
 H    +0.00000    +0.00000    +0.00000
 O    +0.00000    +0.00000    +0.95448
 C    −0.90216    +0.00000    +1.26614
 C    −1.65302    −1.26337    +0.90478
 H    −0.83414    +0.09657    +2.34770
 H    −1.43734    +0.87708    +0.89483
 C    −1.02524    −2.51167    +1.49586
 H    −2.68495    −1.15346    +1.23997
 H    −1.69597    −1.35465    −0.18489
 C    −1.64052    −3.80977    +1.00315
 H    +0.03916    −2.50308    +1.26573
 H    −1.10159    −2.46515    +2.58466
 C    −3.10493    −3.98723    +1.36010
 H    −1.52621    −3.87512    −0.08176
```

```
 H    -1.07552   -4.64745   +1.41531
 C    -3.64808   -5.33816   +0.93968
 H    -3.22722   -3.86176   +2.43771
 H    -3.69834   -3.20070   +0.89336
 H    -4.69845   -5.44751   +1.19950
 H    -3.55722   -5.47800   -0.13635
 H    -3.10128   -6.14693   +1.42167
21
 * E =  +0.762 kcal/mol ; (13) 181_055_054_058_175
 H    +0.00000   +0.00000   +0.00000
 O    +0.00000   +0.00000   +0.95334
 C    -0.89733   +0.00000   +1.27531
 C    -0.85695   +0.01853   +2.78288
 H    -1.43691   +0.87637   +0.90473
 H    -1.43349   -0.88761   +0.93179
 C    -0.04957   +1.17543   +3.35259
 H    -1.88384   +0.04717   +3.14844
 H    -0.42808   -0.92036   +3.13132
 C    -0.47356   +2.55105   +2.86573
 H    -0.11714   +1.14176   +4.44106
 H    +0.99724   +1.02176   +3.09836
 C    -1.92207   +2.90139   +3.15125
 H    +0.16826   +3.29841   +3.33504
 H    -0.28358   +2.63129   +1.79335
 C    -2.26909   +4.32189   +2.75299
 H    -2.58170   +2.20974   +2.62479
 H    -2.12310   +2.75834   +4.21484
 H    -3.31152   +4.55474   +2.95806
 H    -1.65592   +5.04021   +3.29468
 H    -2.09675   +4.48236   +1.68981
21
 * E =  +0.819 kcal/mol ; (14) 065_302_302_186_181
 H    +0.00000   +0.00000   +0.00000
 O    +0.00000   +0.00000   +0.95457
 C    -0.90207   +0.00000   +1.26678
 C    -1.65129   -1.26826   +0.91361
 H    -0.83969   +0.10385   +2.34650
 H    -1.43728   +0.87613   +0.89183
 C    -1.70584   -1.56429   -0.57690
 H    -1.18147   -2.10570   +1.42797
 H    -2.66636   -1.18208   +1.30583
 C    -2.32865   -0.46346   -1.41617
 H    -0.69953   -1.77816   -0.94464
 H    -2.26826   -2.48612   -0.72863
 C    -2.48928   -0.84667   -2.87492
```

```
 H    −3.30467    −0.19943    −1.00200
 H    −1.72608    +0.44670    −1.35679
 C    −3.09580    +0.25950    −3.71400
 H    −1.51583    −1.12471    −3.28229
 H    −3.11035    −1.74105    −2.94207
 H    −3.20401    −0.04027    −4.75362
 H    −4.08224    +0.53470    −3.34463
 H    −2.47598    +1.15433    −3.69082
21
 * E =   +0.837  kcal/mol ;  (15) 065_061_183_185_297
 H    +0.00000    +0.00000    +0.00000
 O    +0.00000    +0.00000    +0.95446
 C    −0.90214    +0.00000    +1.26612
 C    −1.65063    −1.26406    +0.90486
 H    −0.83395    +0.09712    +2.34755
 H    −1.43781    +0.87654    +0.89411
 C    −1.02920    −2.51588    +1.49180
 H    −2.68375    −1.16460    +1.24359
 H    −1.69797    −1.35299    −0.18423
 C    −1.74142    −3.78470    +1.06247
 H    +0.02036    −2.55077    +1.20439
 H    −1.04489    −2.44384    +2.58213
 C    −1.21285    −5.04922    +1.71927
 H    −2.80544    −3.68748    +1.28620
 H    −1.66879    −3.88747    −0.02309
 C    +0.23147    −5.36158    +1.37742
 H    −1.31981    −4.96105    +2.80180
 H    −1.84056    −5.88881    +1.42066
 H    +0.54133    −6.31062    +1.80889
 H    +0.37050    −5.42814    +0.29900
 H    +0.90954    −4.59748    +1.75014
21
 * E =   +0.854  kcal/mol ;  (16) 180_179_174_063_175
 H    +0.00000    +0.00000    +0.00000
 O    +0.00000    +0.00000    +0.95348
 C    −0.89795    +0.00000    +1.27411
 C    −0.86195    −0.00101    +2.77980
 H    −1.43640    +0.88329    +0.91874
 H    −1.43712    −0.88207    +0.91710
 C    −2.25106    +0.02582    +3.38912
 H    −0.32328    −0.88897    +3.11296
 H    −0.27929    +0.85961    +3.10448
 C    −2.26552    −0.11688    +4.90178
 H    −2.75151    +0.95657    +3.11238
 H    −2.84659    −0.77943    +2.95522
```

```
C    -1.56543   +1.00600   +5.64480
H    -3.30130   -0.16950   +5.24137
H    -1.80785   -1.06940   +5.17827
C    -1.70020   +0.87941   +7.14902
H    -0.50895   +1.02388   +5.37846
H    -1.97838   +1.96251   +5.31849
H    -1.18951   +1.68794   +7.66682
H    -2.74583   +0.89947   +7.45207
H    -1.27522   -0.05908   +7.50142
21
 * E =   +0.854  kcal/mol ;  (17) 064_060_182_176_063
H    +0.00000   +0.00000   +0.00000
O    +0.00000   +0.00000   +0.95451
C    -0.90219   +0.00000   +1.26619
C    -1.65717   -1.25531   +0.88846
H    -0.83355   +0.08272   +2.34879
H    -1.43401   +0.88381   +0.90582
C    -1.03902   -2.51950   +1.45196
H    -2.68781   -1.15697   +1.23509
H    -1.71179   -1.32610   -0.20168
C    -1.77293   -3.77592   +1.02271
H    +0.00408   -2.57611   +1.13890
H    -1.02604   -2.44633   +2.54039
C    -1.12883   -5.06857   +1.49605
H    -2.80334   -3.73488   +1.38459
H    -1.83627   -3.79108   -0.06693
C    -1.09828   -5.22483   +3.00437
H    -1.66916   -5.90945   +1.06127
H    -0.11213   -5.12567   +1.10373
H    -0.70124   -6.19588   +3.29118
H    -0.47672   -4.46780   +3.47691
H    -2.09957   -5.14019   +3.42516
21
 * E =   +0.873  kcal/mol ;  (18) 061_177_174_063_176
H    +0.00000   +0.00000   +0.00000
O    +0.00000   +0.00000   +0.95437
C    -0.90192   +0.00000   +1.26641
C    -1.68456   -1.21463   +0.82100
H    -0.83604   +0.02282   +2.35184
H    -1.41947   +0.91271   +0.95657
C    -3.09000   -1.23587   +1.39344
H    -1.73914   -1.22726   -0.27140
H    -1.13087   -2.10432   +1.11714
C    -3.96402   -2.35714   +0.85745
H    -3.03321   -1.31315   +2.48132
```

```
H   −3.57413   −0.28070   +1.18257
C   −3.46482   −3.75262   +1.18364
H   −4.97145   −2.24216   +1.26065
H   −4.05924   −2.25434   −0.22606
C   −4.42130   −4.83408   +0.72285
H   −2.49012   −3.91446   +0.72372
H   −3.31019   −3.83393   +2.26120
H   −4.04739   −5.82733   +0.95981
H   −5.39500   −4.72431   +1.19748
H   −4.57499   −4.78697   −0.35408
21
* E =  +0.883  kcal/mol ; (19) 066_296_183_185_297
H   +0.00000   +0.00000   +0.00000
O   +0.00000   +0.00000   +0.95391
C   −0.90062   +0.00000   +1.26828
C   −1.64830   −1.27241   +0.93530
H   −0.83325   +0.12315   +2.34572
H   −1.44384   +0.86499   +0.87553
C   −1.82322   −1.50368   −0.55383
H   −1.11970   −2.11652   +1.37925
H   −2.62984   −1.22674   +1.41105
C   −2.63458   −2.74813   −0.86468
H   −2.30077   −0.62748   −0.99722
H   −0.84429   −1.60189   −1.03042
C   −2.74017   −3.07451   −2.34499
H   −2.18594   −3.59576   −0.34462
H   −3.63798   −2.63302   −0.44834
C   −3.46310   −2.01845   −3.15893
H   −1.73834   −3.22559   −2.75074
H   −3.25776   −4.02700   −2.45690
H   −3.58151   −2.33193   −4.19350
H   −4.45704   −1.82850   −2.75572
H   −2.92485   −1.07325   −3.16656
21
* E =  +0.886  kcal/mol ; (20) 061_177_179_176_063
H   +0.00000   +0.00000   +0.00000
O   +0.00000   +0.00000   +0.95439
C   −0.90196   +0.00000   +1.26638
C   −1.68603   −1.21045   +0.81311
H   −0.83586   +0.01625   +2.35183
H   −1.41766   +0.91553   +0.96185
C   −3.09667   −1.23702   +1.36920
H   −1.72754   −1.22359   −0.27933
H   −1.14526   −2.10787   +1.11435
C   −3.89308   −2.44018   +0.89885
```

```
H -3.05499 -1.23976 +2.46097
H -3.61134 -0.31768 +1.08611
C -5.27718 -2.55328 +1.51593
H -3.98626 -2.40535 -0.18914
H -3.32792 -3.34537 +1.12583
C -6.20486 -1.40637 +1.16311
H -5.72916 -3.49035 +1.19138
H -5.18079 -2.62371 +2.60071
H -7.20270 -1.57053 +1.56314
H -5.84805 -0.45919 +1.56130
H -6.29609 -1.29703 +0.08311
21
* E = +0.887 kcal/mol ; (21) 180_180_179_176_063
H +0.00000 +0.00000 +0.00000
O +0.00000 +0.00000 +0.95348
C -0.89798 +0.00000 +1.27402
C -0.86127 -0.00302 +2.77953
H -1.43593 +0.88385 +0.91956
H -1.43725 -0.88161 +0.91613
C -2.24496 +0.00187 +3.39887
H -0.30524 -0.88070 +3.10960
H -0.29645 +0.86755 +3.11322
C -2.20685 -0.01804 +4.91584
H -2.79205 +0.88784 +3.06704
H -2.80607 -0.85880 +3.03180
C -3.57070 +0.08919 +5.57748
H -1.71590 -0.93506 +5.24930
H -1.57872 +0.80439 +5.26137
C -4.49771 -1.07163 +5.27120
H -3.43176 +0.15927 +6.65618
H -4.04440 +1.02387 +5.27220
H -5.42858 -0.98924 +5.82753
H -4.75407 -1.11744 +4.21516
H -4.03528 -2.02066 +5.53977
21
* E = +0.889 kcal/mol ; (22) 061_177_180_184_297
H +0.00000 +0.00000 +0.00000
O +0.00000 +0.00000 +0.95437
C -0.90183 +0.00000 +1.26666
C -1.68369 -1.21460 +0.82082
H -0.83531 +0.02264 +2.35196
H -1.41932 +0.91279 +0.95703
C -3.09543 -1.23813 +1.37423
H -1.72171 -1.23733 -0.27153
H -1.14317 -2.10894 +1.13161
```

```
C   −3.88306   −2.45659   +0.92928
H   −3.04960   −1.20367   +2.46347
H   −3.62607   −0.33520   +1.06224
C   −5.33059   −2.46822   +1.39211
H   −3.85831   −2.51200   −0.16025
H   −3.38245   −3.35782   +1.29021
C   −5.49531   −2.52497   +2.89880
H   −5.83833   −1.58398   +1.00291
H   −5.83338   −3.32614   +0.94606
H   −6.54328   −2.60982   +3.17675
H   −4.97241   −3.38476   +3.31573
H   −5.10277   −1.63392   +3.38345
21
 * E =  +0.891  kcal/mol ; (23) 182_063_186_303_303
H   +0.00000   +0.00000   +0.00000
O   +0.00000   +0.00000   +0.95329
C   −0.89766   +0.00000   +1.27418
C   −0.86230   +0.04367   +2.78053
H   −1.44137   +0.86989   +0.89370
H   −1.42457   −0.89691   +0.94058
C   −0.22064   +1.30825   +3.31848
H   −1.88597   −0.05189   +3.14543
H   −0.31595   −0.83031   +3.13895
C   −0.04375   +1.30954   +4.82847
H   +0.75151   +1.43065   +2.84470
H   −0.81844   +2.16964   +3.01325
C   −1.32196   +1.10296   +5.62749
H   +0.67149   +0.53168   +5.10061
H   +0.40951   +2.25647   +5.12661
C   −2.40233   +2.12264   +5.32415
H   −1.70992   +0.09919   +5.44960
H   −1.07766   +1.14185   +6.68897
H   −3.26301   +1.99285   +5.97626
H   −2.03214   +3.13791   +5.46131
H   −2.75604   +2.04007   +4.29784
21
 * E =  +0.898  kcal/mol ; (24) 061_178_185_297_185
H   +0.00000   +0.00000   +0.00000
O   +0.00000   +0.00000   +0.95435
C   −0.90187   +0.00000   +1.26645
C   −1.68289   −1.21679   +0.82430
H   −0.83574   +0.02647   +2.35170
H   −1.42055   +0.91116   +0.95372
C   −3.10101   −1.21903   +1.36491
H   −1.69839   −1.24884   −0.26703
```

```
H   −1.15295   −2.11061   +1.15590
C   −3.88457   −2.48303   +1.05352
H   −3.06506   −1.08522   +2.44728
H   −3.64127   −0.35540   +0.97064
C   −4.11292   −2.73274   −0.42590
H   −3.36939   −3.34297   +1.48723
H   −4.85341   −2.42837   +1.55258
C   −4.99154   −3.94075   −0.68218
H   −4.56843   −1.84645   −0.87205
H   −3.15674   −2.87101   −0.93047
H   −5.14171   −4.10907   −1.74598
H   −4.54710   −4.84252   −0.26420
H   −5.97153   −3.81639   −0.22445
21
 * E =  +0.906 kcal/mol ; (25) 066_296_188_297_184
H   +0.00000   +0.00000   +0.00000
O   +0.00000   +0.00000   +0.95401
C   −0.90090   +0.00000   +1.26789
C   −1.65117   −1.26798   +0.92268
H   −0.83395   +0.11462   +2.34628
H   −1.44137   +0.86934   +0.88068
C   −1.81849   −1.47523   −0.57209
H   −1.12468   −2.11175   +1.36667
H   −2.63599   −1.22174   +1.39274
C   −2.72249   −2.63940   −0.94251
H   −2.22332   −0.56067   −1.01179
H   −0.84038   −1.63585   −1.03202
C   −2.21399   −3.99575   −0.48976
H   −3.71675   −2.47000   −0.52276
H   −2.85105   −2.65699   −2.02601
C   −3.09008   −5.13445   −0.97198
H   −1.19506   −4.13588   −0.85539
H   −2.15272   −4.02437   +0.59785
H   −2.71505   −6.09827   −0.63603
H   −4.10830   −5.02843   −0.60112
H   −3.13860   −5.15828   −2.05943
21
 * E =  +0.917 kcal/mol ; (26) 059_182_296_186_180
H   +0.00000   +0.00000   +0.00000
O   +0.00000   +0.00000   +0.95450
C   −0.90217   +0.00000   +1.26620
C   −1.69027   −1.19374   +0.77336
H   −0.83131   −0.00580   +2.35015
H   −1.41250   +0.92537   +0.98023
C   −3.14293   −1.19386   +1.21872
```

H −1.65305 −1.20602 −0.31839
H −1.19279 −2.10351 +1.11069
C −3.33729 −1.29629 +2.72093
H −3.63641 −0.29065 +0.85250
H −3.65511 −2.02983 +0.74097
C −4.79380 −1.43450 +3.12066
H −2.77604 −2.15403 +3.09920
H −2.91842 −0.41848 +3.21568
C −4.98902 −1.52498 +4.62048
H −5.35509 −0.58475 +2.72869
H −5.21350 −2.32064 +2.64186
H −6.03963 −1.62626 +4.88223
H −4.46095 −2.38318 +5.03268
H −4.60805 −0.63546 +5.11938
21
* E = +0.927 kcal/mol ; (27) 066_296_182_176_063
H +0.00000 +0.00000 +0.00000
O +0.00000 +0.00000 +0.95385
C −0.90041 +0.00000 +1.26865
C −1.64984 −1.27170 +0.93624
H −0.83206 +0.12243 +2.34609
H −1.44355 +0.86556 +0.87701
C −1.83626 −1.49829 −0.55213
H −1.11655 −2.11677 +1.37281
H −2.62725 −1.22877 +1.42055
C −2.63614 −2.75038 −0.86190
H −2.33978 −0.63239 −0.99133
H −0.85964 −1.56961 −1.03398
C −2.92119 −2.96313 −2.33939
H −2.10457 −3.61954 −0.46831
H −3.58250 −2.70317 −0.32079
C −1.67868 −3.17293 −3.18366
H −3.57536 −3.82801 −2.44761
H −3.48282 −2.10945 −2.72273
H −1.93804 −3.40085 −4.21486
H −1.04365 −2.28991 −3.19762
H −1.08315 −4.00117 −2.80190
21
* E = +0.929 kcal/mol ; (28) 064_061_178_065_176
H +0.00000 +0.00000 +0.00000
O +0.00000 +0.00000 +0.95455
C −0.90227 +0.00000 +1.26611
C −1.66082 −1.25032 +0.87758
H −0.83363 +0.07356 +2.34947
H −1.43162 +0.88811 +0.91256

```
 C    −1.04256   −2.51631   +1.43965
 H    −2.69066   −1.15287   +1.22815
 H    −1.71578   −1.30385   −0.21202
 C    −1.80334   −3.78670   +1.09976
 H    −0.01411   −2.59708   +1.08724
 H    −0.98107   −2.41726   +2.52465
 C    −1.82700   −4.13091   −0.37856
 H    −1.35537   −4.62055   +1.64271
 H    −2.83002   −3.70649   +1.46548
 C    −2.50526   −5.45753   −0.65628
 H    −2.33640   −3.34498   −0.93628
 H    −0.80250   −4.15692   −0.75453
 H    −2.51634   −5.68838   −1.71899
 H    −1.99488   −6.27225   −0.14529
 H    −3.53716   −5.44901   −0.30885
21
 * E =   +0.934  kcal/mol ; (29) 182_063_177_058_056
 H    +0.00000   +0.00000   +0.00000
 O    +0.00000   +0.00000   +0.95330
 C    −0.89716   +0.00000   +1.27561
 C    −0.85224   +0.04036   +2.78163
 H    −1.44064   +0.87130   +0.89824
 H    −1.42540   −0.89614   +0.94188
 C    −0.20649   +1.30382   +3.31694
 H    −1.87147   −0.04976   +3.16201
 H    −0.30712   −0.83905   +3.12549
 C    −0.20507   +1.39867   +4.83449
 H    +0.81036   +1.36620   +2.93305
 H    −0.73447   +2.16916   +2.91178
 C    +0.48730   +0.24672   +5.54833
 H    +0.27931   +2.33287   +5.12379
 H    −1.23457   +1.46685   +5.19128
 C    +1.92187   +0.03240   +5.10667
 H    +0.46269   +0.43977   +6.62081
 H    −0.07906   −0.67339   +5.39992
 H    +2.40556   −0.74018   +5.70002
 H    +1.97939   −0.27135   +4.06314
 H    +2.50465   +0.94652   +5.21422
21
 * E =   +0.936  kcal/mol ; (30) 067_296_178_064_175
 H    +0.00000   +0.00000   +0.00000
 O    +0.00000   +0.00000   +0.95375
 C    −0.90001   +0.00000   +1.26937
 C    −1.64328   −1.28267   +0.96363
 H    −0.83113   +0.14306   +2.34421
```

```
H    -1.44912    +0.85465    +0.86254
C    -1.82770    -1.53291    -0.52180
H    -1.10083    -2.11661    +1.41129
H    -2.61420    -1.23154    +1.45700
C    -2.52918    -2.83999    -0.85301
H    -2.38689    -0.70261    -0.96126
H    -0.85051    -1.54110    -1.00825
C    -3.96345    -2.92591    -0.36429
H    -2.52032    -2.98086    -1.93503
H    -1.95643    -3.67098    -0.43554
C    -4.65312    -4.19888    -0.81255
H    -3.98996    -2.86783    +0.72358
H    -4.51836    -2.05977    -0.72998
H    -5.67784    -4.24789    -0.45191
H    -4.68000    -4.26824    -1.89875
H    -4.12942    -5.07822    -0.44121
21
 * E =   +0.941  kcal/mol ; (31) 062_053_054_058_175
H    +0.00000    +0.00000    +0.00000
O    +0.00000    +0.00000    +0.95457
C    -0.90163    +0.00000    +1.26806
C    -1.67311    -1.23414    +0.85234
H    -0.82905    +0.05221    +2.35209
H    -1.42709    +0.89972    +0.93929
C    -0.98021    -2.53884    +1.21630
H    -2.66268    -1.18465    +1.30892
H    -1.84200    -1.20819    -0.22653
C    -0.59797    -2.66680    +2.68162
H    -1.63198    -3.36822    +0.93770
H    -0.07797    -2.63825    +0.61465
C    -1.76164    -2.53848    +3.64686
H    -0.12181    -3.63699    +2.83159
H    +0.16305    -1.92287    +2.92410
C    -1.35687    -2.79371    +5.08491
H    -2.19998    -1.54218    +3.56944
H    -2.54881    -3.23741    +3.35683
H    -2.19946    -2.68975    +5.76467
H    -0.95547    -3.79858    +5.20506
H    -0.58588    -2.09388    +5.40305
21
 * E =   +0.943  kcal/mol ; (32) 177_174_063_174_179
H    +0.00000    +0.00000    +0.00000
O    +0.00000    +0.00000    +0.95349
C    -0.89791    +0.00000    +1.27427
C    -0.85022    -0.06665    +2.77956
```

```
H   −1.42084   +0.91024   +0.96424
H   −1.44810   −0.85295   +0.87110
C   −2.21046   +0.06899   +3.44080
H   −0.38242   −1.00729   +3.07125
H   −0.19057   +0.72882   +3.12374
C   −3.19260   −1.03697   +3.09816
H   −2.06942   +0.08928   +4.52206
H   −2.64958   +1.03413   +3.17802
C   −4.48209   −0.95147   +3.89195
H   −3.43285   −1.01081   +2.03367
H   −2.72123   −2.00624   +3.27847
C   −5.46748   −2.04738   +3.54016
H   −4.24945   −0.99535   +4.95707
H   −4.94250   +0.02342   +3.72339
H   −6.38161   −1.96707   +4.12374
H   −5.74233   −2.00390   +2.48751
H   −5.04105   −3.03159   +3.72688
21
 * E =  +0.990  kcal/mol ; (33) 180_056_056_055_058
H   +0.00000   +0.00000   +0.00000
O   +0.00000   +0.00000   +0.95332
C   −0.89694   +0.00000   +1.27629
C   −0.85323   −0.00803   +2.78425
H   −1.43136   +0.88576   +0.92151
H   −1.43849   −0.87876   +0.91881
C   −0.04695   +1.13905   +3.37237
H   −1.88018   +0.01465   +3.15268
H   −0.42385   −0.95330   +3.11428
C   −0.50478   +2.52291   +2.93657
H   −0.07818   +1.06429   +4.45949
H   +0.99468   +1.01194   +3.08455
C   −1.97327   +2.82428   +3.19419
H   +0.10406   +3.26651   +3.45341
H   −0.29184   +2.65037   +1.87432
C   −2.37595   +2.69005   +4.64992
H   −2.18724   +3.83699   +2.85254
H   −2.59781   +2.16804   +2.58508
H   −3.41071   +2.98743   +4.80379
H   −2.27516   +1.66481   +5.00155
H   −1.75251   +3.31647   +5.28663
21
 * E =  +1.020  kcal/mol ; (34) 062_173_065_174_180
H   +0.00000   +0.00000   +0.00000
O   +0.00000   +0.00000   +0.95442
C   −0.90218   +0.00000   +1.26584
```

```
C   −1.66618   −1.23185   +0.83201
H   −0.83741   +0.03145   +2.35161
H   −1.41551   +0.90950   +0.94628
C   −3.05233   −1.34507   +1.44424
H   −1.75080   −1.23743   −0.25809
H   −1.07065   −2.10299   +1.10253
C   −4.01857   −0.24918   +1.03083
H   −3.47833   −2.30997   +1.16672
H   −2.96580   −1.35802   +2.53295
C   −5.42500   −0.47292   +1.55265
H   −3.66292   +0.72056   +1.38262
H   −4.04542   −0.18503   −0.05991
C   −6.38794   +0.62422   +1.14676
H   −5.79149   −1.43513   +1.19150
H   −5.39496   −0.55014   +2.64064
H   −7.38893   +0.44061   +1.52994
H   −6.06143   +1.59157   +1.52487
H   −6.45776   +0.70255   +0.06305
21
* E =  +1.056 kcal/mol ; (35) 064_302_306_301_185
H   +0.00000   +0.00000   +0.00000
O   +0.00000   +0.00000   +0.95466
C   −0.90223   +0.00000   +1.26667
C   −1.65906   −1.25658   +0.89193
H   −0.83998   +0.08774   +2.34781
H   −1.43235   +0.88565   +0.90429
C   −1.71285   −1.51880   −0.60677
H   −1.18957   −2.10795   +1.38310
H   −2.66913   −1.17942   +1.29628
C   −2.25659   −0.37084   −1.44405
H   −0.71362   −1.77923   −0.96006
H   −2.32221   −2.40565   −0.78343
C   −3.66469   +0.05734   −1.07535
H   −1.59525   +0.49776   −1.37468
H   −2.23997   −0.66672   −2.49400
C   −4.21453   +1.11809   −2.00742
H   −4.31739   −0.81754   −1.08647
H   −3.68048   +0.43333   −0.05175
H   −5.22166   +1.41594   −1.72579
H   −3.59060   +2.01035   −1.99562
H   −4.25026   +0.75693   −3.03382
21
* E =  +1.062 kcal/mol ; (36) 065_061_188_303_303
H   +0.00000   +0.00000   +0.00000
O   +0.00000   +0.00000   +0.95448
```

```
C  -0.90220  +0.00000  +1.26603
C  -1.65267  -1.26336  +0.90467
H  -0.83463  +0.09648  +2.34767
H  -1.43722  +0.87715  +0.89459
C  -1.02978  -2.51126  +1.49994
H  -2.68616  -1.15374  +1.23842
H  -1.69601  -1.35567  -0.18492
C  -1.64800  -3.80657  +0.99890
H  +0.03541  -2.51060  +1.27375
H  -1.10415  -2.45918  +2.58769
C  -3.14899  -3.92814  +1.21486
H  -1.43634  -3.91096  -0.06682
H  -1.15119  -4.64489  +1.49000
C  -3.56905  -3.79395  +2.66549
H  -3.66914  -3.17961  +0.61558
H  -3.47754  -4.89433  +0.83229
H  -4.63537  -3.97036  +2.78600
H  -3.04115  -4.51003  +3.29399
H  -3.35759  -2.79961  +3.05505
21
* E =  +1.078  kcal/mol ;  (37) 179_055_059_170_062
H  +0.00000  +0.00000  +0.00000
O  +0.00000  +0.00000  +0.95330
C  -0.89738  +0.00000  +1.27501
C  -0.85639  -0.01678  +2.78328
H  -1.43033  +0.88689  +0.92284
H  -1.43910  -0.87695  +0.91329
C  -0.04666  +1.12064  +3.38277
H  -1.88403  +0.02052  +3.14886
H  -0.43795  -0.96913  +3.10727
C  -0.55021  +2.50062  +3.00018
H  -0.06102  +1.02311  +4.47004
H  +0.98948  +1.00651  +3.07050
C  +0.09926  +3.62899  +3.78460
H  -0.37735  +2.66659  +1.93501
H  -1.63181  +2.54239  +3.14841
C  +1.59781  +3.73406  +3.57577
H  -0.36895  +4.57171  +3.50095
H  -0.11122  +3.49309  +4.84704
H  +2.00876  +4.59595  +4.09668
H  +2.11784  +2.85200  +3.94210
H  +1.83687  +3.83956  +2.51844
21
* E =  +1.115  kcal/mol ;  (38) 066_297_188_303_303
H  +0.00000  +0.00000  +0.00000
```

```
 O    +0.00000    +0.00000    +0.95402
 C    -0.90085    +0.00000    +1.26805
 C    -1.64469    -1.27565    +0.93777
 H    -0.83463    +0.12797    +2.34501
 H    -1.44523    +0.86210    +0.87043
 C    -1.80222    -1.50772    -0.55358
 H    -1.11569    -2.11176    +1.39544
 H    -2.63104    -1.22944    +1.40462
 C    -2.70017    -2.68463    -0.90358
 H    -2.21414    -0.60673    -1.01391
 H    -0.82038    -1.66146    -1.00564
 C    -2.26891    -4.01622    -0.30766
 H    -3.71668    -2.46260    -0.57482
 H    -2.74462    -2.78088    -1.98962
 C    -0.85636    -4.42199    -0.68019
 H    -2.36221    -3.98085    +0.77817
 H    -2.96614    -4.78449    -0.64112
 H    -0.62287    -5.41797    -0.31155
 H    -0.72277    -4.42957    -1.76137
 H    -0.11813    -3.74002    -0.26194
21
 * E =  +1.117  kcal/mol ; (39) 180_179_174_057_057
 H    +0.00000    +0.00000    +0.00000
 O    +0.00000    +0.00000    +0.95349
 C    -0.89764    +0.00000    +1.27502
 C    -0.86028    -0.00347    +2.78054
 H    -1.43630    +0.88402    +0.92167
 H    -1.43768    -0.88122    +0.91734
 C    -2.24702    +0.02792    +3.39348
 H    -0.32093    -0.89143    +3.11269
 H    -0.27578    +0.85652    +3.10691
 C    -2.25069    -0.12989    +4.90615
 H    -2.74418    +0.96004    +3.11918
 H    -2.85172    -0.77112    +2.96105
 C    -1.42052    +0.89838    +5.65914
 H    -3.28170    -0.08048    +5.26043
 H    -1.88890    -1.12795    +5.15807
 C    -1.82357    +2.33228    +5.37641
 H    -1.51268    +0.70109    +6.72702
 H    -0.36486    +0.76405    +5.42167
 H    -1.26726    +3.02844    +5.99962
 H    -1.63674    +2.60492    +4.33917
 H    -2.88418    +2.48748    +5.57090
21
 * E =  +1.128  kcal/mol ; (40) 061_176_173_057_057
```

```
 H    +0.00000   +0.00000   +0.00000
 O    +0.00000   +0.00000   +0.95438
 C    −0.90213   +0.00000   +1.26583
 C    −1.68527   −1.21260   +0.81629
 H    −0.83710   +0.01978   +2.35139
 H    −1.41869   +0.91398   +0.95815
 C    −3.08934   −1.23944   +1.38992
 H    −1.73863   −1.22346   −0.27622
 H    −1.13208   −2.10468   +1.10969
 C    −3.96006   −2.35658   +0.83573
 H    −3.03095   −1.32080   +2.47647
 H    −3.57901   −0.28518   +1.18868
 C    −3.39458   −3.75765   +1.01233
 H    −4.93888   −2.30703   +1.31544
 H    −4.13232   −2.17685   −0.22674
 C    −3.10229   −4.11980   +2.45525
 H    −4.10616   −4.47143   +0.59783
 H    −2.48574   −3.86544   +0.41939
 H    −2.78711   −5.15659   +2.54529
 H    −2.30864   −3.50235   +2.87220
 H    −3.98481   −3.98610   +3.07967
21
 * E =   +1.154  kcal/mol  ;  (41) 061_178_186_303_303
 H    +0.00000   +0.00000   +0.00000
 O    +0.00000   +0.00000   +0.95437
 C    −0.90177   +0.00000   +1.26684
 C    −1.68434   −1.21417   +0.82081
 H    −0.83537   +0.02272   +2.35213
 H    −1.41973   +0.91275   +0.95745
 C    −3.10289   −1.21728   +1.35844
 H    −1.70146   −1.24210   −0.27164
 H    −1.15472   −2.11043   +1.14638
 C    −3.87585   −2.49077   +1.05168
 H    −3.07421   −1.08156   +2.44066
 H    −3.64185   −0.35397   +0.96434
 C    −3.98166   −2.83698   −0.42565
 H    −3.40695   −3.32425   +1.57670
 H    −4.88122   −2.39661   +1.46524
 C    −4.60706   −1.74050   −1.26543
 H    −2.99371   −3.07868   −0.81911
 H    −4.57111   −3.74813   −0.52505
 H    −5.58325   −1.45593   −0.87482
 H    −3.98851   −0.84464   −1.28322
 H    −4.74345   −2.06129   −2.29548
21
```

∗ E = +1.167 kcal/mol ; (42) 063_054_056_055_058
H +0.00000 +0.00000 +0.00000
O +0.00000 +0.00000 +0.95457
C −0.90180 +0.00000 +1.26756
C −1.66942 −1.23898 +0.85763
H −0.83031 +0.05960 +2.35108
H −1.42908 +0.89610 +0.93182
C −0.98175 −2.53883 +1.24396
H −2.66427 −1.18469 +1.30417
H −1.82709 −1.22377 −0.22316
C −0.65955 −2.66324 +2.72574
H −1.60857 −3.37300 +0.92837
H −0.05213 −2.62673 +0.68376
C −1.84833 −2.47939 +3.65640
H −0.22244 −3.64738 +2.90163
H +0.11610 −1.94133 +2.98279
C −2.98412 −3.44884 +3.39273
H −1.50651 −2.59486 +4.68472
H −2.22188 −1.45635 +3.58246
H −3.77971 −3.33552 +4.12547
H −3.42338 −3.29583 +2.40843
H −2.63574 −4.47989 +3.43823
21
∗ E = +1.211 kcal/mol ; (43) 067_296_177_058_056
H +0.00000 +0.00000 +0.00000
O +0.00000 +0.00000 +0.95376
C −0.89988 +0.00000 +1.26979
C −1.64371 −1.28219 +0.96406
H −0.83034 +0.14293 +2.34461
H −1.44935 +0.85482 +0.86355
C −1.83515 −1.53010 −0.52017
H −1.10250 −2.11730 +1.41118
H −2.61491 −1.23152 +1.45909
C −2.52065 −2.84911 −0.84435
H −2.40071 −0.70220 −0.95357
H −0.86367 −1.52934 −1.01786
C −3.89261 −3.03230 −0.21329
H −2.61656 −2.93517 −1.92790
H −1.87419 −3.66939 −0.52925
C −4.87543 −1.93129 −0.56006
H −4.29527 −3.99126 −0.53856
H −3.79432 −3.10473 +0.87021
H −5.86549 −2.14780 −0.16595
H −4.56572 −0.97139 −0.15008
H −4.96684 −1.81247 −1.63902

```
21
 * E =   +1.214  kcal/mol  ;  (44)  179_055_059_177_294
 H    +0.00000    +0.00000    +0.00000
 O    +0.00000    +0.00000    +0.95331
 C    -0.89744    +0.00000    +1.27487
 C    -0.85698    -0.01905    +2.78311
 H    -1.43062    +0.88620    +0.92180
 H    -1.43915    -0.87687    +0.91288
 C    -0.03839    +1.10751    +3.39206
 H    -1.88441    +0.02396    +3.14858
 H    -0.44628    -0.97556    +3.10476
 C    -0.52459    +2.49609    +3.02090
 H    -0.05085    +0.98742    +4.47489
 H    +0.99841    +0.99782    +3.07517
 C    +0.24540    +3.62039    +3.69581
 H    -0.44463    +2.62241    +1.94100
 H    -1.58617    +2.59085    +3.26649
 C    +0.06582    +3.67812    +5.20090
 H    +1.30537    +3.51860    +3.45749
 H    -0.07141    +4.57040    +3.26531
 H    +0.58087    +4.53689    +5.62555
 H    -0.98800    +3.76213    +5.46388
 H    +0.45791    +2.79046    +5.69177
21
 * E =   +1.229  kcal/mol  ;  (45)  065_061_177_058_056
 H    +0.00000    +0.00000    +0.00000
 O    +0.00000    +0.00000    +0.95450
 C    -0.90214    +0.00000    +1.26630
 C    -1.65051    -1.26396    +0.90332
 H    -0.83394    +0.09601    +2.34790
 H    -1.43772    +0.87695    +0.89490
 C    -1.02323    -2.51287    +1.49137
 H    -2.68306    -1.16704    +1.24626
 H    -1.70044    -1.34240    -0.18597
 C    -1.79593    -3.78982    +1.20047
 H    +0.00082    -2.59896    +1.13055
 H    -0.94532    -2.38896    +2.57266
 C    -2.00521    -4.09416    -0.27575
 H    -1.26874    -4.62736    +1.66024
 H    -2.76994    -3.73832    +1.69033
 C    -0.71648    -4.16549    -1.07110
 H    -2.53523    -5.04255    -0.36159
 H    -2.66447    -3.34623    -0.71795
 H    -0.90244    -4.46751    -2.09915
 H    -0.20849    -3.20298    -1.09963
```

```
 H    −0.02527    −4.88453    −0.63346
21
 * E =  +1.241  kcal/mol ; (46) 177_174_057_057_175
 H    +0.00000    +0.00000    +0.00000
 O    +0.00000    +0.00000    +0.95350
 C    −0.89798    +0.00000    +1.27412
 C    −0.85328    −0.06259    +2.77895
 H    −1.42379    +0.90660    +0.95850
 H    −1.44702    −0.85675    +0.87446
 C    −2.21868    +0.07836    +3.43203
 H    −0.37842    −0.99778    +3.07358
 H    −0.19878    +0.73549    +3.12658
 C    −3.25614    −0.94459    +2.99248
 H    −2.09202    +0.01008    +4.51300
 H    −2.60641    +1.07945    +3.23671
 C    −2.83557    −2.38764    +3.19855
 H    −4.17910    −0.76308    +3.54563
 H    −3.50970    −0.79313    +1.94095
 C    −3.93991    −3.36968    +2.86296
 H    −1.95933    −2.60802    +2.58746
 H    −2.52279    −2.52545    +4.23525
 H    −3.61965    −4.39870    +3.00827
 H    −4.81549    −3.20327    +3.48831
 H    −4.25434    −3.26470    +1.82568
21
 * E =  +1.250  kcal/mol ; (47) 063_054_058_170_062
 H    +0.00000    +0.00000    +0.00000
 O    +0.00000    +0.00000    +0.95456
 C    −0.90147    +0.00000    +1.26847
 C    −1.67136    −1.23750    +0.85711
 H    −0.82949    +0.06008    +2.35130
 H    −1.42881    +0.89610    +0.93270
 C    −0.98875    −2.54065    +1.23734
 H    −2.66131    −1.18680    +1.31503
 H    −1.83517    −1.21490    −0.22275
 C    −0.72942    −2.68091    +2.72648
 H    −1.60912    −3.37309    +0.89974
 H    −0.04666    −2.61572    +0.69626
 C    −0.28229    −4.07290    +3.14166
 H    +0.03130    −1.95964    +3.02965
 H    −1.63861    −2.42199    +3.27389
 C    +1.02917    −4.50507    +2.51422
 H    −0.18676    −4.10277    +4.22703
 H    −1.06294    −4.79166    +2.88553
 H    +1.34920    −5.47160    +2.89679
```

```
 H    +0.94840    −4.59301    +1.43306
 H    +1.81812    −3.78513    +2.72692
21
 * E =   +1.251  kcal/mol ; (48) 059_182_302_304_185
 H    +0.00000    +0.00000    +0.00000
 O    +0.00000    +0.00000    +0.95446
 C    −0.90219    +0.00000    +1.26600
 C    −1.69241    −1.19024    +0.77067
 H    −0.83141    −0.01214    +2.35087
 H    −1.41045    +0.92860    +0.98712
 C    −3.14640    −1.18210    +1.21710
 H    −1.66134    −1.20163    −0.32116
 H    −1.19229    −2.10083    +1.09896
 C    −3.35601    −1.13376    +2.72366
 H    −3.65489    −0.33023    +0.76275
 H    −3.63561    −2.07245    +0.82055
 C    −2.67979    −2.25913    +3.48402
 H    −3.01083    −0.17663    +3.11971
 H    −4.42797    −1.16523    +2.92474
 C    −3.02099    −2.25015    +4.96054
 H    −2.97122    −3.21508    +3.04464
 H    −1.59795    −2.18858    +3.36459
 H    −2.52150    −3.05656    +5.49215
 H    −2.72048    −1.31235    +5.42503
 H    −4.09262    −2.36383    +5.11568
21
 * E =   +1.312  kcal/mol ; (49) 065_302_305_305_302
 H    +0.00000    +0.00000    +0.00000
 O    +0.00000    +0.00000    +0.95458
 C    −0.90214    +0.00000    +1.26662
 C    −1.65224    −1.26615    +0.90959
 H    −0.84008    +0.10318    +2.34641
 H    −1.43688    +0.87712    +0.89121
 C    −1.71294    −1.54453    −0.58494
 H    −1.17546    −2.10836    +1.40943
 H    −2.66200    −1.18984    +1.31730
 C    −2.29259    −0.41212    −1.42208
 H    −0.71100    −1.77896    −0.94846
 H    −2.29316    −2.45212    −0.74852
 C    −3.67980    +0.04626    −0.99876
 H    −1.61975    +0.44847    −1.40093
 H    −2.32703    −0.73369    −2.46402
 C    −4.71542    −1.06109    −1.00950
 H    −3.63280    +0.48921    −0.00277
 H    −3.99950    +0.84670    −1.66541
```

```
H   −5.70635   −0.67517   −0.78278
H   −4.76288   −1.54350   −1.98490
H   −4.48998   −1.83074   −0.27362
21
∗ E =   +1.384  kcal/mol  ;  (50)  066_302_302_189_297
H   +0.00000   +0.00000   +0.00000
O   +0.00000   +0.00000   +0.95464
C   −0.90217   +0.00000   +1.26678
C   −1.64752   −1.27334   +0.92326
H   −0.84050   +0.11289   +2.34565
H   −1.43976   +0.87137   +0.88416
C   −1.69672   −1.57934   −0.56546
H   −1.17643   −2.10514   +1.44551
H   −2.66380   −1.18614   +1.31233
C   −2.32665   −0.48020   −1.40411
H   −0.68728   −1.79260   −0.92082
H   −2.25745   −2.50326   −0.71499
C   −2.56382   −0.87089   −2.85357
H   −3.27692   −0.19145   −0.95109
H   −1.70125   +0.41647   −1.38156
C   −1.29227   −1.17939   −3.62051
H   −3.22720   −1.73676   −2.88508
H   −3.09614   −0.06225   −3.35383
H   −1.50369   −1.38399   −4.66732
H   −0.60089   −0.33795   −3.58395
H   −0.77733   −2.04857   −3.21757
21
∗ E =   +1.393  kcal/mol  ;  (51)  061_172_057_056_174
H   +0.00000   +0.00000   +0.00000
O   +0.00000   +0.00000   +0.95444
C   −0.90222   +0.00000   +1.26580
C   −1.67649   −1.21622   +0.80965
H   −0.83819   +0.01520   +2.35199
H   −1.41081   +0.91844   +0.96060
C   −3.05644   −1.33417   +1.43858
H   −1.76385   −1.19850   −0.27923
H   −1.08907   −2.10002   +1.05538
C   −3.97848   −0.14417   +1.21350
H   −3.53792   −2.23216   +1.04982
H   −2.94245   −1.49379   +2.51173
C   −4.20510   +0.20369   −0.24570
H   −4.94178   −0.36099   +1.67776
H   −3.59238   +0.73425   +1.73418
C   −5.23188   +1.30295   −0.42984
H   −3.26349   +0.51235   −0.70238
```

```
H    -4.52312   -0.69145   -0.78367
H    -5.37553   +1.54640   -1.47990
H    -6.19753   +1.00827   -0.02257
H    -4.92509   +2.21363   +0.08206
21
 * E =  +1.397  kcal/mol ; (52) 062_053_059_176_294
H    +0.00000   +0.00000   +0.00000
O    +0.00000   +0.00000   +0.95459
C    -0.90162   +0.00000   +1.26817
C    -1.67370   -1.23285   +0.84729
H    -0.83011   +0.05486   +2.35115
H    -1.42726   +0.89892   +0.93721
C    -0.99590   -2.54474   +1.20717
H    -2.66291   -1.18559   +1.30706
H    -1.84041   -1.19782   -0.23185
C    -0.73741   -2.71631   +2.69257
H    -1.61717   -3.36117   +0.84039
H    -0.04551   -2.61438   +0.67694
C    -0.14678   -4.06752   +3.06332
H    -0.05322   -1.93601   +3.02476
H    -1.66986   -2.56875   +3.24464
C    -1.08267   -5.23864   +2.83224
H    +0.77602   -4.21918   +2.50098
H    +0.14090   -4.04686   +4.11432
H    -0.63765   -6.17047   +3.17377
H    -2.01785   -5.10185   +3.37384
H    -1.32709   -5.36398   +1.77987
21
 * E =  +1.447  kcal/mol ; (53) 177_174_058_054_058
H    +0.00000   +0.00000   +0.00000
O    +0.00000   +0.00000   +0.95349
C    -0.89755   +0.00000   +1.27528
C    -0.85024   -0.06563   +2.77995
H    -1.42266   +0.90799   +0.96269
H    -1.44845   -0.85538   +0.87570
C    -2.21515   +0.06183   +3.43531
H    -0.36935   -1.00023   +3.06988
H    -0.19941   +0.73469   +3.12931
C    -3.23431   -0.98509   +3.00362
H    -2.08511   +0.01617   +4.51628
H    -2.62294   +1.05227   +3.22749
C    -2.76888   -2.42485   +3.15486
H    -4.14430   -0.84358   +3.58900
H    -3.52014   -0.81236   +1.96500
C    -2.37345   -2.78976   +4.57242
```

```
H  −3.56915  −3.08540  +2.82167
H  −1.92852  −2.61279  +2.48435
H  −2.12785  −3.84590  +4.65414
H  −1.50370  −2.22631  +4.90490
H  −3.18467  −2.58353  +5.26965
21
* E =  +1.459 kcal/mol ; (54) 059_182_302_306_302
H  +0.00000  +0.00000  +0.00000
O  +0.00000  +0.00000  +0.95447
C  −0.90216  +0.00000  +1.26611
C  −1.69188  −1.19241  +0.77489
H  −0.83210  −0.00889  +2.35096
H  −1.41160  +0.92690  +0.98381
C  −3.14193  −1.18889  +1.23206
H  −1.66669  −1.20467  −0.31705
H  −1.18669  −2.10163  +1.10199
C  −3.33213  −1.16232  +2.74350
H  −3.65252  −0.32641  +0.80089
H  −3.63974  −2.06576  +0.81889
C  −2.59794  −2.26125  +3.49573
H  −3.01901  −0.19399  +3.13560
H  −4.39938  −1.23818  +2.95738
C  −2.97933  −3.66027  +3.05263
H  −1.52045  −2.12683  +3.38709
H  −2.80484  −2.15063  +4.55986
H  −2.49235  −4.41463  +3.66591
H  −4.05496  −3.81496  +3.12840
H  −2.69304  −3.84726  +2.01923
21
* E =  +1.469 kcal/mol ; (55) 179_077_299_301_185
H  +0.00000  +0.00000  +0.00000
O  +0.00000  +0.00000  +0.95358
C  −0.89838  +0.00000  +1.27332
C  −0.88464  −0.03187  +2.78243
H  −1.43123  +0.89060  +0.92564
H  −1.43065  −0.87566  +0.89496
C  −0.54915  +1.28946  +3.45820
H  −1.87382  −0.34585  +3.11653
H  −0.19274  −0.81404  +3.09463
C  +0.82109  +1.87326  +3.15202
H  −1.31422  +2.02006  +3.18810
H  −0.63665  +1.14858  +4.53717
C  +1.97848  +0.96435  +3.51753
H  +0.89022  +2.11973  +2.09288
H  +0.92004  +2.81381  +3.69817
```

```
 C    +3.32343    +1.63427    +3.32023
 H    +1.87572    +0.64694    +4.55779
 H    +1.93098    +0.06528    +2.90535
 H    +4.14500    +0.96997    +3.57909
 H    +3.45646    +1.93508    +2.28230
 H    +3.41332    +2.52850    +3.93565
21
 * E =   +1.510  kcal/mol ; (56) 059_182_297_190_297
 H    +0.00000    +0.00000    +0.00000
 O    +0.00000    +0.00000    +0.95451
 C    -0.90223    +0.00000    +1.26608
 C    -1.69167    -1.19172    +0.77075
 H    -0.83208    -0.00905    +2.35014
 H    -1.41186    +0.92656    +0.98265
 C    -3.14289    -1.19006    +1.22152
 H    -1.65671    -1.20142    -0.32112
 H    -1.19440    -2.10255    +1.10573
 C    -3.31889    -1.28083    +2.72837
 H    -3.63435    -0.29218    +0.84343
 H    -3.65575    -2.03376    +0.75677
 C    -4.75350    -1.52962    +3.16376
 H    -2.68644    -2.08573    +3.10763
 H    -2.96149    -0.36431    +3.20130
 C    -5.71149    -0.41841    +2.77936
 H    -5.09826    -2.47218    +2.73481
 H    -4.77503    -1.66267    +4.24519
 H    -6.70826    -0.60410    +3.17256
 H    -5.37447    +0.54000    +3.17247
 H    -5.80154    -0.31845    +1.69993
21
 * E =   +1.531  kcal/mol ; (57) 178_174_062_169_063
 H    +0.00000    +0.00000    +0.00000
 O    +0.00000    +0.00000    +0.95350
 C    -0.89786    +0.00000    +1.27445
 C    -0.85030    -0.06256    +2.77987
 H    -1.42210    +0.90858    +0.96183
 H    -1.44752    -0.85489    +0.87441
 C    -2.21363    +0.06426    +3.43699
 H    -0.37718    -1.00002    +3.07356
 H    -0.19643    +0.73795    +3.12340
 C    -3.18028    -1.05357    +3.08206
 H    -2.07763    +0.07877    +4.51963
 H    -2.64974    +1.02962    +3.17513
 C    -4.44121    -1.06728    +3.93026
 H    -3.46473    -0.98082    +2.03040
```

```
H −2.66592 −2.01025 +3.19261
C −5.29603 +0.17603 +3.77607
H −5.03354 −1.94374 +3.66784
H −4.16421 −1.19068 +4.97872
H −6.21956 +0.09238 +4.34423
H −4.77888 +1.06696 +4.12529
H −5.56369 +0.33788 +2.73253
21
* E = +1.536 kcal/mol ; (58) 179_068_283_183_179
H +0.00000 +0.00000 +0.00000
O +0.00000 +0.00000 +0.95357
C −0.89819 +0.00000 +1.27380
C −0.89798 −0.02331 +2.78206
H −1.42971 +0.89014 +0.92379
H −1.43381 −0.87616 +0.90022
C −0.36711 +1.23370 +3.45465
H −1.93004 −0.18576 +3.09467
H −0.33085 −0.89340 +3.11560
C +1.14348 +1.38562 +3.44799
H −0.82320 +2.10936 +2.98571
H −0.70894 +1.23956 +4.49139
C +1.60865 +2.62268 +4.19177
H +1.59097 +0.50060 +3.90642
H +1.50692 +1.40826 +2.42276
C +3.11685 +2.77062 +4.20403
H +1.15950 +3.50657 +3.73507
H +1.23614 +2.59137 +5.21755
H +3.42936 +3.66425 +4.73981
H +3.58922 +1.91467 +4.68336
H +3.51070 +2.83570 +3.19121
21
* E = +1.552 kcal/mol ; (59) 062_067_290_185_180
H +0.00000 +0.00000 +0.00000
O +0.00000 +0.00000 +0.95445
C −0.90199 +0.00000 +1.26652
C −1.70336 −1.21743 +0.85495
H −0.83252 +0.04806 +2.35145
H −1.41690 +0.90579 +0.93608
C −1.26638 −2.53382 +1.47715
H −2.74234 −1.02463 +1.12687
H −1.69158 −1.30222 −0.23553
C +0.06273 −3.07934 +0.98652
H −1.22975 −2.41700 +2.56279
H −2.03998 −3.27920 +1.28290
C +0.38054 −4.44663 +1.56082
```

```
 H    +0.04057   -3.14648   -0.10479
 H    +0.86093   -2.38118   +1.23149
 C    +1.70290   -4.99950   +1.06892
 H    +0.39467   -4.38265   +2.65020
 H    -0.42324   -5.14114   +1.30950
 H    +1.91208   -5.97851   +1.49407
 H    +1.70459   -5.10235   -0.01512
 H    +2.52578   -4.33886   +1.33619
21
 * E =  +1.571  kcal/mol ; (60) 065_302_302_182_065
 H    +0.00000   +0.00000   +0.00000
 O    +0.00000   +0.00000   +0.95456
 C    -0.90208   +0.00000   +1.26670
 C    -1.65215   -1.26685   +0.91022
 H    -0.83948   +0.09932   +2.34687
 H    -1.43555   +0.87896   +0.89626
 C    -1.70642   -1.56699   -0.57999
 H    -1.18332   -2.10443   +1.42530
 H    -2.66762   -1.18079   +1.30161
 C    -2.34181   -0.47059   -1.41696
 H    -0.69770   -1.76734   -0.95032
 H    -2.25433   -2.49768   -0.71925
 C    -2.44342   -0.80141   -2.89780
 H    -3.33912   -0.25236   -1.02709
 H    -1.77138   +0.45385   -1.30782
 C    -3.37321   -1.95814   -3.21074
 H    -2.78763   +0.08442   -3.43103
 H    -1.44679   -1.01952   -3.28590
 H    -3.46703   -2.10561   -4.28405
 H    -3.01740   -2.89377   -2.78591
 H    -4.37082   -1.77345   -2.81458
21
 * E =  +1.583  kcal/mol ; (61) 061_173_058_054_058
 H    +0.00000   +0.00000   +0.00000
 O    +0.00000   +0.00000   +0.95445
 C    -0.90210   +0.00000   +1.26621
 C    -1.67721   -1.21576   +0.80995
 H    -0.83715   +0.01358   +2.35237
 H    -1.41122   +0.91838   +0.96232
 C    -3.05799   -1.33010   +1.43644
 H    -1.76441   -1.19638   -0.27980
 H    -1.09082   -2.10041   +1.05501
 C    -3.97645   -0.13933   +1.19142
 H    -3.53394   -2.23773   +1.06597
 H    -2.94864   -1.47065   +2.51265
```

```
 C    −4.15207   +0.23806   −0.27106
 H    −4.95483   −0.36563   +1.61809
 H    −3.60698   +0.72831   +1.73905
 C    −4.69042   −0.88941   −1.13014
 H    −4.82643   +1.09188   −0.33116
 H    −3.20024   +0.58347   −0.67907
 H    −4.87298   −0.55716   −2.14934
 H    −3.99508   −1.72545   −1.17937
 H    −5.63059   −1.26872   −0.73195
21
 * E =  +1.614 kcal/mol ; (62) 062_173_064_170_063
 H    +0.00000   +0.00000   +0.00000
 O    +0.00000   +0.00000   +0.95443
 C    −0.90217   +0.00000   +1.26593
 C    −1.66822   −1.22884   +0.82749
 H    −0.83735   +0.02742   +2.35183
 H    −1.41484   +0.91120   +0.94982
 C    −3.05255   −1.34097   +1.44427
 H    −1.75677   −1.22742   −0.26241
 H    −1.07331   −2.10246   +1.09129
 C    −4.00387   −0.22419   +1.04684
 H    −3.49082   −2.29575   +1.14886
 H    −2.95430   −1.37751   +2.53018
 C    −5.44471   −0.47027   +1.46308
 H    −3.67012   +0.72207   +1.47638
 H    −3.96367   −0.09782   −0.03706
 C    −5.64089   −0.55966   +2.96435
 H    −6.06751   +0.33195   +1.06772
 H    −5.79955   −1.38977   +0.99412
 H    −6.69314   −0.66354   +3.21847
 H    −5.11797   −1.41340   +3.38957
 H    −5.26808   +0.33582   +3.46008
21
 * E =  +1.682 kcal/mol ; (63) 059_182_295_182_065
 H    +0.00000   +0.00000   +0.00000
 O    +0.00000   +0.00000   +0.95451
 C    −0.90214   +0.00000   +1.26634
 C    −1.69012   −1.19321   +0.77153
 H    −0.83041   −0.00580   +2.35008
 H    −1.41237   +0.92548   +0.98063
 C    −3.14500   −1.19704   +1.21166
 H    −1.64993   −1.20308   −0.32017
 H    −1.19293   −2.10369   +1.10747
 C    −3.33891   −1.32711   +2.71266
 H    −3.63298   −0.28243   +0.86546
```

```
H   −3.65266   −2.01858   +0.70831
C   −4.79575   −1.38378   +3.14470
H   −2.82447   −2.22545   +3.06266
H   −2.86109   −0.48762   +3.21741
C   −5.53956   −2.61313   +2.65864
H   −4.83795   −1.35154   +4.23316
H   −5.30791   −0.48525   +2.79603
H   −6.55125   −2.64183   +3.05659
H   −5.61832   −2.64041   +1.57429
H   −5.03421   −3.52436   +2.97592
21
 * E =   +1.715  kcal/mol ; (64) 177_174_063_177_294
H   +0.00000   +0.00000   +0.00000
O   +0.00000   +0.00000   +0.95350
C   −0.89789   +0.00000   +1.27436
C   −0.84883   −0.06843   +2.77972
H   −1.42016   +0.91124   +0.96618
H   −1.44811   −0.85166   +0.86891
C   −2.20508   +0.07794   +3.44804
H   −0.38645   −1.01207   +3.07050
H   −0.18222   +0.72223   +3.12164
C   −3.18982   −1.03044   +3.11705
H   −2.04945   +0.11405   +4.52532
H   −2.64501   +1.04020   +3.17439
C   −4.51818   −0.91685   +3.84847
H   −3.38556   −1.03701   +2.04456
H   −2.73286   −1.99590   +3.34870
C   −4.41422   −1.08574   +5.35231
H   −4.97102   +0.04961   +3.61994
H   −5.19999   −1.67011   +3.45403
H   −5.39698   −1.06730   +5.81777
H   −3.94682   −2.03642   +5.60549
H   −3.82431   −0.29610   +5.81174
21
 * E =   +1.792  kcal/mol ; (65) 062_173_066_178_295
H   +0.00000   +0.00000   +0.00000
O   +0.00000   +0.00000   +0.95442
C   −0.90223   +0.00000   +1.26572
C   −1.66595   −1.23118   +0.82890
H   −0.83739   +0.02845   +2.35152
H   −1.41402   +0.91108   +0.94858
C   −3.05268   −1.35120   +1.43982
H   −1.74997   −1.23499   −0.26130
H   −1.06845   −2.10160   +1.09744
C   −4.02478   −0.26699   +1.00661
```

H −3.45837 −2.32683 +1.17605
H −2.96895 −1.34374 +2.52928
C −5.42215 −0.42213 +1.58618
H −3.63722 +0.70973 +1.29567
H −4.08775 −0.25910 −0.08467
C −6.15617 −1.66222 +1.11275
H −5.35889 −0.42868 +2.67570
H −6.00826 +0.45832 +1.32363
H −7.17389 −1.68507 +1.49538
H −6.21188 −1.69019 +0.02527
H −5.66717 −2.57628 +1.44141
21
* E = +1.806 kcal/mol ; (66) 179_074_295_305_304
H +0.00000 +0.00000 +0.00000
O +0.00000 +0.00000 +0.95356
C −0.89845 +0.00000 +1.27303
C −0.89041 −0.02990 +2.78209
H −1.43121 +0.89024 +0.92460
H −1.43108 −0.87605 +0.89570
C −0.50550 +1.27608 +3.46015
H −1.89384 −0.30447 +3.10868
H −0.23158 −0.83900 +3.10024
C +0.91710 +1.76326 +3.22100
H −1.20173 +2.05368 +3.14003
H −0.66773 +1.15983 +4.53245
C +2.00097 +0.75768 +3.57463
H +1.03433 +2.04528 +2.17662
H +1.06498 +2.67013 +3.81135
C +1.93482 +0.26144 +5.00624
H +1.94420 −0.08252 +2.88383
H +2.97119 +1.22334 +3.40078
H +2.77994 −0.38210 +5.24043
H +1.94518 +1.09137 +5.71246
H +1.02915 −0.31370 +5.19276
21
* E = +1.869 kcal/mol ; (67) 063_276_056_171_178
H +0.00000 +0.00000 +0.00000
O +0.00000 +0.00000 +0.95335
C −0.89825 +0.00000 +1.27277
C −1.68497 −1.23972 +0.88909
H −0.81000 +0.06089 +2.35384
H −1.42772 +0.89985 +0.94236
C −2.32389 −1.20421 −0.49036
H −1.01415 −2.09555 +0.97448
H −2.47365 −1.38933 +1.62701

C −1.37117 −0.95290 −1.64579
H −2.83542 −2.15275 −0.66070
H −3.10135 −0.43744 −0.50522
C −2.02522 −1.13174 −3.00301
H −0.98331 +0.06884 −1.59061
H −0.51256 −1.62510 −1.56959
C −1.08697 −0.84054 −4.15629
H −2.40092 −2.15267 −3.08287
H −2.89905 −0.48154 −3.06615
H −1.57779 −0.98182 −5.11618
H −0.72548 +0.18581 −4.11861
H −0.21808 −1.49599 −4.12968
21
* E = +1.907 kcal/mol ; (68) 066_073_299_299_185
H +0.00000 +0.00000 +0.00000
O +0.00000 +0.00000 +0.95418
C −0.90207 +0.00000 +1.26520
C −1.66749 −1.26451 +0.93244
H −0.83749 +0.12350 +2.34481
H −1.43462 +0.86990 +0.87338
C −1.28341 −2.48727 +1.75235
H −2.72756 −1.06230 +1.09291
H −1.56554 −1.47009 −0.13570
C +0.15756 −2.95670 +1.62683
H −1.49343 −2.27312 +2.80183
H −1.94695 −3.30855 +1.47495
C +0.55928 −3.36314 +0.22221
H +0.83295 −2.17659 +1.97519
H +0.29624 −3.81180 +2.29126
C +1.96343 −3.92977 +0.16141
H −0.15186 −4.09767 −0.16193
H +0.49422 −2.49864 −0.43882
H +2.24087 −4.21114 −0.85202
H +2.69032 −3.20096 +0.51541
H +2.05506 −4.81561 +0.78795
21
* E = +2.174 kcal/mol ; (69) 178_070_288_189_296
H +0.00000 +0.00000 +0.00000
O +0.00000 +0.00000 +0.95358
C −0.89911 +0.00000 +1.27126
C −0.90154 −0.05106 +2.77896
H −1.42588 +0.89805 +0.93424
H −1.43726 −0.86738 +0.88136
C −0.42822 +1.21298 +3.47968
H −1.92537 −0.26277 +3.08919

```
 H    -0.29719    -0.90302    +3.09360
 C    +1.06370    +1.48464    +3.38518
 H    -0.99029    +2.06212    +3.08617
 H    -0.69808    +1.13870    +4.53565
 C    +1.52629    +2.63155    +4.26862
 H    +1.60036    +0.57742    +3.66689
 H    +1.33745    +1.68030    +2.35012
 C    +0.93289    +3.97594    +3.89155
 H    +1.28407    +2.40744    +5.30954
 H    +2.61325    +2.69632    +4.21840
 H    +1.34983    +4.77677    +4.49829
 H    +1.13674    +4.21098    +2.84757
 H    -0.14632    +3.99387    +4.02791
21
 * E =   +2.186  kcal/mol ; (70) 061_067_290_189_296
 H    +0.00000    +0.00000    +0.00000
 O    +0.00000    +0.00000    +0.95446
 C    -0.90207    +0.00000    +1.26633
 C    -1.70716    -1.21101    +0.84412
 H    -0.83290    +0.03869    +2.35168
 H    -1.41406    +0.91018    +0.94337
 C    -1.27466    -2.53341    +1.45692
 H    -2.74570    -1.01686    +1.11688
 H    -1.69454    -1.28705    -0.24712
 C    +0.05625    -3.07130    +0.95858
 H    -1.24804    -2.42062    +2.54200
 H    -2.04733    -3.27742    +1.25050
 C    +0.35190    -4.48621    +1.42849
 H    +0.05067    -3.05628    -0.13372
 H    +0.85984    -2.40455    +1.26648
 C    +0.49508    -4.61463    +2.93325
 H    -0.43726    -5.15552    +1.07997
 H    +1.27120    -4.82844    +0.95329
 H    +0.78361    -5.62411    +3.21741
 H    +1.25672    -3.93352    +3.31042
 H    -0.43523    -4.38598    +3.44870
21
 * E =   +2.235  kcal/mol ; (71) 066_070_295_303_304
 H    +0.00000    +0.00000    +0.00000
 O    +0.00000    +0.00000    +0.95419
 C    -0.90131    +0.00000    +1.26742
 C    -1.67190    -1.26209    +0.93824
 H    -0.83332    +0.12107    +2.34695
 H    -1.43564    +0.87053    +0.87901
 C    -1.24153    -2.50371    +1.70403
```

H −2.72327 −1.06816 +1.15651
H −1.62293 −1.44073 −0.13937
C +0.16474 −3.01434 +1.42075
H −1.32846 −2.29710 +2.77217
H −1.95981 −3.29844 +1.49902
C +0.44779 −3.32686 −0.03991
H +0.89363 −2.28614 +1.76943
H +0.31744 −3.92184 +2.00823
C −0.51992 −4.31654 −0.65909
H +0.44200 −2.40196 −0.61796
H +1.46272 −3.71565 −0.11912
H −0.22466 −4.57790 −1.67276
H −0.56270 −5.23739 −0.07856
H −1.53083 −3.91513 −0.70835
21
* E = +2.264 kcal/mol ; (72) 180_067_279_177_063
H +0.00000 +0.00000 +0.00000
O +0.00000 +0.00000 +0.95361
C −0.90001 +0.00000 +1.26882
C −0.91450 −0.00509 +2.77707
H −1.43178 +0.88427 +0.90490
H −1.43003 −0.88216 +0.90111
C −0.37178 +1.25405 +3.43915
H −1.95246 −0.15117 +3.07774
H −0.36374 −0.87855 +3.12896
C +1.14349 +1.32038 +3.52806
H −0.74210 +2.13039 +2.90006
H −0.79155 +1.32000 +4.44286
C +1.66580 +2.61489 +4.12947
H +1.49694 +0.47816 +4.12829
H +1.56327 +1.18434 +2.53505
C +1.24645 +2.84911 +5.56859
H +2.75439 +2.61035 +4.07351
H +1.33659 +3.45429 +3.51385
H +1.71364 +3.74335 +5.97525
H +0.17013 +2.97527 +5.66322
H +1.53603 +2.00938 +6.19946
21
* E = +2.265 kcal/mol ; (73) 070_278_060_057_176
H +0.00000 +0.00000 +0.00000
O +0.00000 +0.00000 +0.95306
C −0.89768 +0.00000 +1.27320
C −1.63844 −1.30220 +1.03773
H −0.81355 +0.18298 +2.34099
H −1.46047 +0.83670 +0.84732

```
C   −2.23917   −1.47787   −0.34985
H   −0.96071   −2.12212   +1.27558
H   −2.45212   −1.35954   +1.76185
C   −1.26798   −1.45249   −1.52057
H   −2.77710   −2.42683   −0.37228
H   −2.99206   −0.70284   −0.50348
C   −0.16534   −2.49297   −1.44864
H   −1.83566   −1.60576   −2.43998
H   −0.83275   −0.45424   −1.63157
C   +0.71315   −2.49810   −2.68363
H   +0.44858   −2.32343   −0.56427
H   −0.61958   −3.47665   −1.31752
H   +1.49496   −3.25045   −2.61438
H   +0.13072   −2.70534   −3.57986
H   +1.19830   −1.53328   −2.82569
21
* E =  +2.286  kcal/mol ; (74) 176_059_060_263_176
H   +0.00000   +0.00000   +0.00000
O   +0.00000   +0.00000   +0.95345
C   −0.89721   +0.00000   +1.27608
C   −0.86023   −0.10008   +2.77958
H   −1.41659   +0.91433   +0.97339
H   −1.45455   −0.84613   +0.86800
C   −0.11373   +1.03138   +3.46947
H   −1.89046   −0.12895   +3.13854
H   −0.40398   −1.05273   +3.04744
C   −0.68622   +2.42115   +3.20002
H   −0.12399   +0.82834   +4.53944
H   +0.93139   +1.00318   +3.16166
C   +0.02907   +3.18158   +2.09635
H   −1.74888   +2.33910   +2.95360
H   −0.64314   +3.02193   +4.10938
C   −0.62048   +4.51467   +1.78718
H   +1.06537   +3.33887   +2.39860
H   +0.07868   +2.56843   +1.19760
H   −0.07991   +5.05569   +1.01375
H   −1.64532   +4.38170   +1.44304
H   −0.65419   +5.14903   +2.67187
21
* E =  +2.292  kcal/mol ; (75) 061_057_059_263_176
H   +0.00000   +0.00000   +0.00000
O   +0.00000   +0.00000   +0.95459
C   −0.90200   +0.00000   +1.26705
C   −1.68566   −1.21445   +0.82150
H   −0.83458   +0.02907   +2.35250
```

```
H   −1.41770   +0.91135   +0.95514
C   −1.07586   −2.54647   +1.23016
H   −2.69326   −1.13292   +1.23410
H   −1.80106   −1.18480   −0.26480
C   −0.90347   −2.71913   +2.73755
H   −1.71046   −3.33817   +0.83441
H   −0.10613   −2.66091   +0.74451
C   +0.49461   −2.39911   +3.23950
H   −1.63043   −2.09320   +3.26308
H   −1.14230   −3.74468   +3.02168
C   +0.60715   −2.48390   +4.74768
H   +1.19550   −3.09779   +2.77999
H   +0.79825   −1.41230   +2.89339
H   +1.61923   −2.27431   +5.08603
H   −0.05655   −1.76841   +5.23131
H   +0.33576   −3.47543   +5.10774
21
 * E =  +2.328 kcal/mol ; (76) 062_069_290_182_065
H   +0.00000   +0.00000   +0.00000
O   +0.00000   +0.00000   +0.95445
C   −0.90226   +0.00000   +1.26574
C   −1.70774   −1.21459   +0.85270
H   −0.83416   +0.04790   +2.35080
H   −1.41506   +0.90692   +0.93513
C   −1.30311   −2.53080   +1.49793
H   −2.74970   −1.00636   +1.10101
H   −1.67576   −1.31177   −0.23637
C   +0.03241   −3.09142   +1.04030
H   −1.28458   −2.40180   +2.58315
H   −2.08817   −3.25947   +1.29542
C   +0.38758   −4.42503   +1.67841
H   +0.01378   −3.21004   −0.04687
H   +0.81521   −2.36833   +1.25469
C   −0.54327   −5.56412   +1.30792
H   +1.40393   −4.68958   +1.38698
H   +0.40729   −4.30823   +2.76354
H   −0.19876   −6.50630   +1.72828
H   −1.55505   −5.39951   +1.67164
H   −0.59761   −5.68576   +0.22665
21
 * E =  +2.344 kcal/mol ; (77) 069_300_092_183_182
H   +0.00000   +0.00000   +0.00000
O   +0.00000   +0.00000   +0.95415
C   −0.90184   +0.00000   +1.26573
C   −1.64367   −1.29309   +1.00065
```

```
 H    −0.84352    +0.18313    +2.33548
 H    −1.45486    +0.83432    +0.82372
 C    −1.74250    −1.66220    −0.47661
 H    −1.15993    −2.10209    +1.54892
 H    −2.64188    −1.18167    +1.42509
 C    −0.62077    −2.55967    −0.97292
 H    −2.68939    −2.16812    −0.66601
 H    −1.77837    −0.74904    −1.07979
 C    −0.70353    −2.85302    −2.45806
 H    +0.35008    −2.12108    −0.73829
 H    −0.65475    −3.49932    −0.41751
 C    +0.39290    −3.78217    −2.93850
 H    −1.67832    −3.28875    −2.68369
 H    −0.65807    −1.91492    −3.01433
 H    +0.31530    −3.97557    −4.00579
 H    +1.37725    −3.35733    −2.74935
 H    +0.34622    −4.74039    −2.42398
21
 * E =  +2.345  kcal/mol ; (78) 061_300_301_093_177
 H    +0.00000    +0.00000    +0.00000
 O    +0.00000    +0.00000    +0.95503
 C    −0.90247    +0.00000    +1.26748
 C    −1.68572    −1.21530    +0.82278
 H    −0.83655    +0.02002    +2.35194
 H    −1.41845    +0.91555    +0.96353
 C    −1.81358    −1.38440    −0.68498
 H    −1.21376    −2.10331    +1.24229
 H    −2.68468    −1.15051    +1.25806
 C    −2.48945    −0.22737    −1.41634
 H    −0.82868    −1.56604    −1.12323
 H    −2.37385    −2.30077    −0.86379
 C    −1.54386    +0.83735    −1.94850
 H    −3.05364    −0.61916    −2.26338
 H    −3.22742    +0.23942    −0.75918
 C    −2.26583    +1.92676    −2.71578
 H    −0.98215    +1.29503    −1.13339
 H    −0.80960    +0.35994    −2.60066
 H    −1.57379    +2.67209    −3.09998
 H    −2.81089    +1.51138    −3.56167
 H    −2.98622    +2.43943    −2.08080
21
 * E =  +2.464  kcal/mol ; (79) 064_277_058_168_062
 H    +0.00000    +0.00000    +0.00000
 O    +0.00000    +0.00000    +0.95322
 C    −0.89832    +0.00000    +1.27205
```

C −1.68153 −1.24683 +0.90531
H −0.81110 +0.07443 +2.35243
H −1.43115 +0.89344 +0.93024
C −2.29986 −1.24372 −0.48414
H −1.01547 −2.10279 +1.02197
H −2.48190 −1.37393 +1.63500
C −1.31570 −1.06752 −1.62925
H −2.83476 −2.18447 −0.62878
H −3.05485 −0.45769 −0.53119
C −1.90881 −1.36213 −2.99779
H −0.94133 −0.03898 −1.63789
H −0.45587 −1.72078 −1.46760
C −3.06111 −0.45087 −3.37358
H −1.12359 −1.27746 −3.74860
H −2.24198 −2.40089 −3.02168
H −3.40573 −0.65053 −4.38535
H −3.91175 −0.58094 −2.70840
H −2.76248 +0.59575 −3.32706
21
∗ E = +2.507 kcal/mol ; (80) 070_280_063_054_058
H +0.00000 +0.00000 +0.00000
O +0.00000 +0.00000 +0.95304
C −0.89765 +0.00000 +1.27322
C −1.64142 −1.30066 +1.04081
H −0.81421 +0.18373 +2.34102
H −1.46074 +0.83594 +0.84648
C −2.20573 −1.50133 −0.35813
H −0.97774 −2.12260 +1.31287
H −2.47421 −1.33484 +1.74453
C −1.19398 −1.57524 −1.49447
H −2.79829 −2.41639 −0.35954
H −2.90967 −0.69493 −0.57105
C −0.09493 −2.61109 −1.31083
H −1.73553 −1.79648 −2.41592
H −0.74892 −0.59136 −1.66472
C −0.62380 −4.02001 −1.12478
H +0.55724 −2.58375 −2.18369
H +0.52739 −2.34221 −0.45714
H +0.18830 −4.74194 −1.08733
H −1.18880 −4.11805 −0.19968
H −1.28294 −4.30418 −1.94432
21
∗ E = +2.541 kcal/mol ; (81) 062_055_089_297_302
H +0.00000 +0.00000 +0.00000
O +0.00000 +0.00000 +0.95471

```
C    −0.90100    +0.00000    +1.27041
C    −1.67435    −1.23448    +0.84632
H    −0.82329    +0.06848    +2.35057
H    −1.42733    +0.89514    +0.93008
C    −1.03354    −2.56519    +1.22354
H    −2.68428    −1.16935    +1.25758
H    −1.79414    −1.19042    −0.23870
C    −1.41921    −3.11689    +2.58872
H    −1.31770    −3.30920    +0.48010
H    +0.04937    −2.46683    +1.14753
C    −1.02828    −2.27060    +3.78990
H    −2.49822    −3.28212    +2.60812
H    −0.96111    −4.10172    +2.69771
C    +0.45987    −1.99190    +3.87880
H    −1.58427    −1.33218    +3.77862
H    −1.35231    −2.78840    +4.69299
H    +0.70259    −1.43115    +4.77871
H    +1.02454    −2.92311    +3.90873
H    +0.82029    −1.41947    +3.02595
21
 * E =  +2.590  kcal/mol ; (82) 063_061_097_300_185
H    +0.00000    +0.00000    +0.00000
O    +0.00000    +0.00000    +0.95458
C    −0.90198    +0.00000    +1.26707
C    −1.66111    −1.24364    +0.84959
H    −0.82850    +0.06273    +2.34897
H    −1.42827    +0.89377    +0.92290
C    −1.06892    −2.54013    +1.39541
H    −2.70318    −1.14137    +1.16072
H    −1.68125    −1.27075    −0.24199
C    −1.74359    −3.06111    +2.65453
H    −1.13029    −3.31674    +0.63437
H    −0.00511    −2.38433    +1.58039
C    −1.70530    −2.12037    +3.84446
H    −2.78563    −3.30195    +2.42922
H    −1.27254    −4.00365    +2.93913
C    −2.29354    −2.74357    +5.09450
H    −0.67337    −1.81900    +4.03334
H    −2.25333    −1.20685    +3.60923
H    −2.26897    −2.05539    +5.93619
H    −3.33055    −3.03498    +4.93495
H    −1.74284    −3.63790    +5.38122
21
 * E =  +2.598  kcal/mol ; (83) 184_065_098_300_184
H    +0.00000    +0.00000    +0.00000
```

O	+0.00000	+0.00000	+0.95333
C	−0.89734	+0.00000	+1.27523
C	−0.84430	+0.11113	+2.77994
H	−1.45431	+0.83675	+0.84851
H	−1.40619	−0.92401	+0.99026
C	−0.27208	+1.43459	+3.28144
H	−1.84848	−0.04287	+3.17939
H	−0.23820	−0.72027	+3.13687
C	−1.31622	+2.46710	+3.67551
H	+0.36156	+1.24770	+4.14691
H	+0.38741	+1.84169	+2.51407
C	−2.25828	+2.89179	+2.56442
H	−1.90811	+2.07789	+4.50796
H	−0.80679	+3.35320	+4.05872
C	−3.20298	+3.99608	+2.99482
H	−1.67432	+3.22177	+1.70290
H	−2.84146	+2.03313	+2.22856
H	−3.87820	+4.28396	+2.19227
H	−3.81053	+3.68164	+3.84197
H	−2.65220	+4.88441	+3.29953

21

∗ E = +2.599 kcal/mol ; (84) 177_057_089_298_302

H	+0.00000	+0.00000	+0.00000
O	+0.00000	+0.00000	+0.95342
C	−0.89800	+0.00000	+1.27373
C	−0.85776	−0.07473	+2.78299
H	−1.42015	+0.89600	+0.93511
H	−1.44251	−0.86528	+0.88830
C	−0.07414	+1.03839	+3.47008
H	−1.88404	−0.10046	+3.15487
H	−0.41102	−1.03485	+3.03860
C	−0.87130	+2.28558	+3.82493
H	+0.33739	+0.64255	+4.39779
H	+0.78616	+1.29351	+2.85263
C	−1.47605	+3.05209	+2.65944
H	−1.67027	+2.00679	+4.51486
H	−0.21684	+2.96110	+4.37920
C	−0.45202	+3.51277	+1.64027
H	−2.23877	+2.44295	+2.17267
H	−2.00519	+3.91946	+3.05503
H	−0.91845	+4.08505	+0.84113
H	+0.29808	+4.14960	+2.10757
H	+0.07345	+2.67346	+1.18787

21

∗ E = +2.646 kcal/mol ; (85) 060_176_261_060_176

```
H    +0.00000   +0.00000   +0.00000
O    +0.00000   +0.00000   +0.95448
C    −0.90175   +0.00000   +1.26732
C    −1.68775   −1.21001   +0.80776
H    −0.83676   +0.02623   +2.35157
H    −1.42078   +0.91180   +0.95673
C    −3.09740   −1.26687   +1.39398
H    −1.73696   −1.18852   −0.28245
H    −1.12954   −2.10960   +1.07083
C    −3.24110   −2.18459   +2.59803
H    −3.41475   −0.25822   +1.67123
H    −3.79870   −1.59571   +0.62906
C    −2.36307   −1.82852   +3.78263
H    −4.28421   −2.18365   +2.91914
H    −3.02081   −3.20943   +2.29056
C    −2.61310   −2.72373   +4.97961
H    −1.31325   −1.89802   +3.49553
H    −2.53721   −0.78704   +4.06072
H    −1.97165   −2.46085   +5.81734
H    −3.64593   −2.64886   +5.31579
H    −2.42371   −3.76710   +4.73265
21
 * E =   +2.650  kcal/mol  ;  (86)  180_183_100_300_183
H    +0.00000   +0.00000   +0.00000
O    +0.00000   +0.00000   +0.95352
C    −0.89777   +0.00000   +1.27478
C    −0.85834   +0.01241   +2.78240
H    −1.43626   +0.88428   +0.92161
H    −1.43945   −0.87875   +0.91660
C    −2.24674   −0.05044   +3.41589
H    −0.24755   −0.82609   +3.11944
H    −0.33256   +0.91480   +3.08880
C    −2.65634   −1.43247   +3.90004
H    −2.29150   +0.63355   +4.26154
H    −2.99068   +0.31436   +2.70272
C    −2.70148   −2.49901   +2.82247
H    −1.96566   −1.75190   +4.68382
H    −3.63839   −1.36573   +4.37170
C    −3.18981   −3.83441   +3.34705
H    −3.35066   −2.16395   +2.01071
H    −1.70823   −2.62411   +2.38949
H    −3.21044   −4.58967   +2.56490
H    −2.54358   −4.20031   +4.14322
H    −4.19604   −3.75079   +3.75440
21
```

* E = +2.652 kcal/mol ; (87) 182_064_188_098_302

H	+0.00000	+0.00000	+0.00000
O	+0.00000	+0.00000	+0.95328
C	−0.89695	+0.00000	+1.27612
C	−0.84838	+0.05254	+2.78117
H	−1.44369	+0.86608	+0.89135
H	−1.42218	−0.90066	+0.95033
C	−0.24000	+1.34056	+3.30874
H	−1.86244	−0.05980	+3.16752
H	−0.28254	−0.80849	+3.13439
C	−0.00024	+1.33567	+4.81724
H	+0.69953	+1.52498	+2.78675
H	−0.90268	+2.16409	+3.03936
C	+1.42609	+0.99159	+5.21971
H	−0.24127	+2.31444	+5.23061
H	−0.68959	+0.63310	+5.29216
C	+1.90920	−0.35961	+4.73030
H	+2.09183	+1.76720	+4.83711
H	+1.50554	+1.03305	+6.30641
H	+2.91652	−0.56613	+5.08529
H	+1.26297	−1.16130	+5.08667
H	+1.92893	−0.40637	+3.64307

21

* E = +2.688 kcal/mol ; (88) 062_275_055_174_292

H	+0.00000	+0.00000	+0.00000
O	+0.00000	+0.00000	+0.95336
C	−0.89860	+0.00000	+1.27181
C	−1.69342	−1.22694	+0.86345
H	−0.81073	+0.04020	+2.35386
H	−1.42140	+0.90973	+0.95794
C	−2.35254	−1.15167	−0.50557
H	−1.02286	−2.08571	+0.91466
H	−2.47190	−1.39788	+1.60748
C	−1.41325	−0.84667	−1.65998
H	−2.86016	−2.09750	−0.69171
H	−3.13378	−0.38844	−0.48524
C	−2.06954	−0.93071	−3.03002
H	−1.01877	+0.16570	−1.54112
H	−0.56017	−1.53017	−1.63011
C	−2.47477	−2.33512	−3.43445
H	−2.94306	−0.27688	−3.04724
H	−1.37970	−0.53354	−3.77414
H	−2.88525	−2.34837	−4.44132
H	−1.61787	−3.00701	−3.41850
H	−3.23092	−2.74925	−2.77178

```
21
 * E =   +2.707  kcal/mol  ;  (89)  061_179_100_300_183
 H    +0.00000    +0.00000    +0.00000
 O    +0.00000    +0.00000    +0.95441
 C    -0.90199    +0.00000    +1.26637
 C    -1.67738    -1.22494    +0.82952
 H    -0.83574    +0.02047    +2.35185
 H    -1.41951    +0.91290    +0.96106
 C    -3.11552    -1.23946    +1.34574
 H    -1.67920    -1.28158    -0.26278
 H    -1.13138    -2.09980    +1.17798
 C    -4.15747    -0.79583    +0.33093
 H    -3.37066    -2.24329    +1.68047
 H    -3.18735    -0.60524    +2.23293
 C    -3.98145    +0.61821    -0.18888
 H    -4.14655    -1.48772    -0.51458
 H    -5.14818    -0.88457    +0.77985
 C    -5.09083    +1.03265    -1.13453
 H    -3.93935    +1.30999    +0.65465
 H    -3.02223    +0.70398    -0.70167
 H    -4.94644    +2.04542    -1.50316
 H    -5.13706    +0.36950    -1.99685
 H    -6.06005    +0.99467    -0.64025
21
 * E =   +2.800  kcal/mol  ;  (90)  178_298_181_097_301
 H    +0.00000    +0.00000    +0.00000
 O    +0.00000    +0.00000    +0.95326
 C    -0.89701    +0.00000    +1.27587
 C    -0.85675    -0.05930    +2.78122
 H    -1.42175    +0.90219    +0.95301
 H    -1.44438    -0.86399    +0.88725
 C    -0.19234    -1.31943    +3.30768
 H    -0.31296    +0.81335    +3.14523
 H    -1.87594    +0.02775    +3.15652
 C    -0.15846    -1.41308    +4.83198
 H    -0.70460    -2.19568    +2.90033
 H    +0.81773    -1.35050    +2.90567
 C    -1.27462    -2.24707    +5.44248
 H    +0.79195    -1.84039    +5.14937
 H    -0.18835    -0.40650    +5.25577
 C    -2.67422    -1.76157    +5.12079
 H    -1.16794    -3.27852    +5.10174
 H    -1.14303    -2.27164    +6.52447
 H    -3.42463    -2.35766    +5.63534
 H    -2.81077    -0.72374    +5.42268
```

H −2.88352 −1.82713 +4.05464
21
∗ E = +2.822 kcal/mol ; (91) 182_063_185_301_096
H +0.00000 +0.00000 +0.00000
O +0.00000 +0.00000 +0.95327
C −0.89743 +0.00000 +1.27473
C −0.85991 +0.04548 +2.78123
H −1.44201 +0.86905 +0.89357
H −1.42410 −0.89777 +0.94319
C −0.22210 +1.31440 +3.31301
H −1.88293 −0.04885 +3.14715
H −0.31024 −0.82652 +3.13918
C −0.04929 +1.35694 +4.82296
H +0.74855 +1.43693 +2.83542
H −0.82943 +2.16860 +3.00251
C −1.34395 +1.21855 +5.62318
H +0.64728 +0.57306 +5.12863
H +0.43492 +2.29980 +5.07495
C −1.63620 −0.19662 +6.08909
H −1.30005 +1.86341 +6.50017
H −2.17852 +1.59052 +5.02473
H −2.57696 −0.24958 +6.63331
H −1.69413 −0.89617 +5.25880
H −0.85028 −0.54949 +6.75487
21
∗ E = +2.833 kcal/mol ; (92) 184_301_300_096_072
H +0.00000 +0.00000 +0.00000
O +0.00000 +0.00000 +0.95347
C −0.89736 +0.00000 +1.27574
C −0.86064 +0.09544 +2.77968
H −1.45365 +0.84764 +0.86943
H −1.41678 −0.91319 +0.97025
C −0.09892 −1.03063 +3.46212
H −0.41529 +1.05210 +3.05156
H −1.89096 +0.11039 +3.13940
C −0.65866 −2.42407 +3.18058
H +0.94505 −0.98090 +3.15575
H −0.11096 −0.83591 +4.53357
C +0.04400 −3.17143 +2.05414
H −0.59494 −3.03514 +4.08213
H −1.72447 −2.34143 +2.95558
C +1.43059 −3.65131 +2.43764
H −0.56176 −4.03038 +1.76388
H +0.12125 −2.52813 +1.17838
H +1.90737 −4.18170 +1.61636

```
 H    +2.07958    -2.82115    +2.70839
 H    +1.38698    -4.32866    +3.28961
21
 * E =  +2.843  kcal/mol  ;  (93)  178_175_059_265_178
 H    +0.00000    +0.00000    +0.00000
 O    +0.00000    +0.00000    +0.95351
 C    -0.89817    +0.00000    +1.27361
 C    -0.84770    -0.05471    +2.77833
 H    -1.42607    +0.90329    +0.95193
 H    -1.44372    -0.86225    +0.88186
 C    -2.20085    +0.05968    +3.46120
 H    -0.36531    -0.98831    +3.06926
 H    -0.19590    +0.74984    +3.11704
 C    -3.20751    -1.02475    +3.08264
 H    -2.02719    +0.03647    +4.53569
 H    -2.63443    +1.04036    +3.25185
 C    -4.16524    -0.62944    +1.97132
 H    -2.67298    -1.93453    +2.79699
 H    -3.80214    -1.29472    +3.95600
 C    -5.10841    -1.75028    +1.58463
 H    -4.74097    +0.23758    +2.29889
 H    -3.60830    -0.30182    +1.09324
 H    -5.80163    -1.44128    +0.80576
 H    -4.55898    -2.61489    +1.21565
 H    -5.69656    -2.07809    +2.44033
21
 * E =  +2.863  kcal/mol  ;  (94)  059_181_300_096_182
 H    +0.00000    +0.00000    +0.00000
 O    +0.00000    +0.00000    +0.95450
 C    -0.90219    +0.00000    +1.26616
 C    -1.68763    -1.19213    +0.76854
 H    -0.83095    -0.01628    +2.35065
 H    -1.41067    +0.92881    +0.98810
 C    -3.14168    -1.22211    +1.21409
 H    -1.65201    -1.20066    -0.32355
 H    -1.18054    -2.09843    +1.10123
 C    -3.34406    -1.24766    +2.72795
 H    -3.66955    -0.36195    +0.79589
 H    -3.60554    -2.10019    +0.76789
 C    -3.59701    +0.11593    +3.34868
 H    -4.19013    -1.89016    +2.97374
 H    -2.47531    -1.70936    +3.20420
 C    -3.73756    +0.05536    +4.85586
 H    -2.79475    +0.80496    +3.08452
 H    -4.50518    +0.53472    +2.91230
```

```
H    −3.94089    +1.03610    +5.27923
H    −4.55223    −0.60630    +5.14604
H    −2.82788    −0.32306    +5.31918
21
* E =   +2.867  kcal/mol  ;  (95)  064_057_059_263_287
H    +0.00000    +0.00000    +0.00000
O    +0.00000    +0.00000    +0.95445
C    −0.90074    +0.00000    +1.27012
C    −1.66108    −1.25261    +0.89517
H    −0.82962    +0.08858    +2.35200
H    −1.43482    +0.88350    +0.91280
C    −1.01316    −2.54599    +1.36514
H    −2.66570    −1.17320    +1.31583
H    −1.78870    −1.28239    −0.18964
C    −0.81646    −2.62666    +2.87827
H    −1.63351    −3.37342    +1.02383
H    −0.05157    −2.66269    +0.86554
C    +0.57581    −2.22959    +3.35368
H    −1.56448    −1.99939    +3.36835
H    −1.01570    −3.64302    +3.22094
C    +1.62835    −3.27300    +3.03164
H    +0.86570    −1.28176    +2.90217
H    +0.55186    −2.07216    +4.43226
H    +2.61141    −2.96184    +3.37733
H    +1.39420    −4.22494    +3.50677
H    +1.70314    −3.44915    +1.96032
21
* E =   +2.918  kcal/mol  ;  (96)  292_060_267_285_179
H    +0.00000    +0.00000    +0.00000
O    +0.00000    +0.00000    +0.95434
C    −0.90203    +0.00000    +1.26597
C    −1.64760    +1.28531    +0.97559
H    −1.45018    −0.84498    +0.83815
H    −0.84242    −0.16403    +2.33869
C    −1.74345    +1.61197    −0.51320
H    −2.64517    +1.18409    +1.40365
H    −1.16334    +2.10338    +1.50854
C    −0.63299    +2.51602    −1.03474
H    −1.75852    +0.67741    −1.08094
H    −2.69918    +2.09199    −0.72560
C    −0.79265    +3.96658    −0.61687
H    +0.33658    +2.15588    −0.68708
H    −0.61052    +2.46971    −2.12483
C    +0.33412    +4.84702    −1.11782
H    −1.74676    +4.34018    −0.99267
```

```
H    −0.84807   +4.03352   +0.47002
H    +0.19570   +5.88329   −0.81861
H    +1.29346   +4.51423   −0.72551
H    +0.39737   +4.82197   −2.20458
21
* E =  +2.931  kcal/mol ; (97) 302_067_235_070_182
H    +0.00000   +0.00000   +0.00000
O    +0.00000   +0.00000   +0.95541
C    −0.90257   +0.00000   +1.26873
C    −1.69440   +1.18658   +0.76630
H    −1.40560   −0.93132   +0.99077
H    −0.83418   +0.02134   +2.35294
C    −1.93261   +1.17001   −0.74756
H    −2.64674   +1.21447   +1.29463
H    −1.15716   +2.09121   +1.05259
C    −1.47253   +2.43090   −1.46054
H    −1.42234   +0.31310   −1.19973
H    −2.98962   +1.00645   −0.94949
C    +0.03650   +2.58367   −1.50076
H    −1.85538   +2.43340   −2.48211
H    −1.91283   +3.30130   −0.96882
C    +0.48156   +3.86728   −2.17164
H    +0.44125   +2.53882   −0.48916
H    +0.46130   +1.72991   −2.03531
H    +1.56545   +3.94762   −2.20186
H    +0.11549   +3.92288   −3.19552
H    +0.10027   +4.73704   −1.63937
21
* E =  +2.939  kcal/mol ; (98) 183_062_177_059_263
H    +0.00000   +0.00000   +0.00000
O    +0.00000   +0.00000   +0.95329
C    −0.89721   +0.00000   +1.27545
C    −0.85528   +0.06259   +2.78115
H    −1.44517   +0.86309   +0.88587
H    −1.42111   −0.90332   +0.95436
C    −0.20936   +1.33428   +3.29567
H    −1.87601   −0.02151   +3.15913
H    −0.30846   −0.80932   +3.14012
C    −0.21157   +1.48398   +4.80887
H    +0.81605   +1.37158   +2.92903
H    −0.72213   +2.19185   +2.85487
C    +0.50852   +0.36583   +5.56267
H    +0.25184   +2.44061   +5.04776
H    −1.24123   +1.55307   +5.16818
C    −0.41476   −0.72090   +6.08342
```

```
 H   +1.26829   −0.07372   +4.91365
 H   +1.04972   +0.78766   +6.40880
 H   +0.14194   −1.50604   +6.59086
 H   −1.12722   −0.30868   +6.79631
 H   −0.98786   −1.18558   +5.28462
21
 * E =  +2.951 kcal/mol ; (99) 066_062_186_098_301
 H   +0.00000   +0.00000   +0.00000
 O   +0.00000   +0.00000   +0.95447
 C   −0.90233   +0.00000   +1.26563
 C   −1.64505   −1.26948   +0.91309
 H   −0.83524   +0.10611   +2.34647
 H   −1.43998   +0.87221   +0.88645
 C   −1.03259   −2.50931   +1.54045
 H   −2.68196   −1.16821   +1.23938
 H   −1.68320   −1.37216   −0.17352
 C   −1.67333   −3.81884   +1.08340
 H   +0.03719   −2.52345   +1.32735
 H   −1.11776   −2.41286   +2.62327
 C   −0.91183   −4.54095   −0.01786
 H   −1.76261   −4.49744   +1.93078
 H   −2.69515   −3.62515   +0.74800
 C   −0.72608   −3.73437   −1.28784
 H   +0.06753   −4.83077   +0.36628
 H   −1.43149   −5.46963   −0.25453
 H   −0.22448   −4.31987   −2.05501
 H   −1.68380   −3.40852   −1.69236
 H   −0.12057   −2.84766   −1.10830
21
 * E =  +2.967 kcal/mol ; (100) 069_301_093_181_067
 H   +0.00000   +0.00000   +0.00000
 O   +0.00000   +0.00000   +0.95433
 C   −0.90237   +0.00000   +1.26493
 C   −1.64478   −1.29090   +0.99063
 H   −0.84553   +0.17766   +2.33567
 H   −1.45327   +0.83737   +0.82603
 C   −1.72813   −1.65417   −0.48933
 H   −1.16843   −2.10243   +1.54172
 H   −2.64716   −1.17834   +1.40477
 C   −0.60468   −2.56306   −0.96468
 H   −2.67478   −2.15592   −0.69265
 H   −1.75386   −0.73586   −1.08242
 C   −0.66498   −2.90244   −2.44444
 H   +0.36477   −2.12052   −0.73039
 H   −0.64980   −3.48564   −0.38438
```

```
C    −0.41546    −1.71814    −3.35921
H    +0.07307    −3.67539    −2.65719
H    −1.63778    −3.34143    −2.67331
H    −0.40855    −2.02101    −4.40363
H    −1.17965    −0.95149    −3.25126
H    +0.54927    −1.25856    −3.14599
21
 * E =   +2.975  kcal/mol  ;  (101)  295_299_173_059_265
H    +0.00000    +0.00000    +0.00000
O    +0.00000    +0.00000    +0.95449
C    −0.90229    +0.00000    +1.26581
C    −1.65137    +1.26510    +0.90627
H    −1.43804    −0.87592    +0.89271
H    −0.83465    −0.09829    +2.34729
C    −1.02295    +2.50806    +1.50560
H    −1.69472    +1.35861    −0.18301
H    −2.68341    +1.15768    +1.24404
C    −1.63738    +3.82277    +1.05281
H    −1.09158    +2.44160    +2.59402
H    +0.04037    +2.50569    +1.26923
C    −3.12614    +3.97717    +1.36048
H    −1.08246    +4.62938    +1.53055
H    −1.47818    +3.94696    −0.02099
C    −4.04095    +3.59308    +0.21136
H    −3.37391    +3.38519    +2.24415
H    −3.33513    +5.01166    +1.63046
H    −5.08843    +3.70296    +0.48430
H    −3.85740    +4.22980    −0.65260
H    −3.88877    +2.56444    −0.10628
21
 * E =   +2.990  kcal/mol  ;  (102)  069_301_093_188_298
H    +0.00000    +0.00000    +0.00000
O    +0.00000    +0.00000    +0.95430
C    −0.90185    +0.00000    +1.26632
C    −1.64562    −1.29068    +0.99496
H    −0.84289    +0.17853    +2.33679
H    −1.45335    +0.83711    +0.82776
C    −1.73299    −1.65574    −0.48452
H    −1.16661    −2.10215    +1.54393
H    −2.64570    −1.17806    +1.41440
C    −0.61282    −2.57152    −0.95457
H    −2.68899    −2.13717    −0.68330
H    −1.73801    −0.74133    −1.08824
C    −0.57788    −2.79084    −2.45763
H    +0.34700    −2.17707    −0.62209
```

H −0.72108 −3.53564 −0.45266
C −1.82442 −3.45306 −3.01298
H −0.42342 −1.83272 −2.95815
H +0.29070 −3.40185 −2.70224
H −1.71915 −3.66217 −4.07495
H −2.02062 −4.39815 −2.50808
H −2.70477 −2.82580 −2.89170
21
* E = +2.994 kcal/mol ; (103) 299_187_301_096_182
H +0.00000 +0.00000 +0.00000
O +0.00000 +0.00000 +0.95449
C −0.90204 +0.00000 +1.26655
C −1.67058 +1.21951 +0.81020
H −1.41267 −0.91503 +0.95611
H −0.83763 −0.01829 +2.35273
C −3.06013 +1.35927 +1.41270
H −1.07518 +2.09887 +1.05402
H −1.75747 +1.19412 −0.27964
C −4.00836 +0.19706 +1.12388
H −2.97321 +1.49189 +2.49335
H −3.49088 +2.28496 +1.03469
C −4.06801 −0.85068 +2.22252
H −5.01835 +0.57812 +0.96931
H −3.72637 −0.27958 +0.18120
C −4.97750 −2.01142 +1.87514
H −3.06759 −1.22377 +2.44175
H −4.41634 −0.37403 +3.14002
H −5.02552 −2.73854 +2.68224
H −5.99176 −1.66795 +1.67770
H −4.62835 −2.52962 +0.98342
21
* E = +3.018 kcal/mol ; (104) 065_060_181_263_059
H +0.00000 +0.00000 +0.00000
O +0.00000 +0.00000 +0.95449
C −0.90229 +0.00000 +1.26582
C −1.65209 −1.26325 +0.90411
H −0.83447 +0.09764 +2.34736
H −1.43772 +0.87677 +0.89384
C −1.01545 −2.51765 +1.47576
H −2.68359 −1.16246 +1.24193
H −1.70127 −1.34969 −0.18514
C −1.73582 −3.81221 +1.10355
H +0.01729 −2.55039 +1.13438
H −0.96303 −2.42882 +2.56403
C −2.70899 −4.32398 +2.15479

```
 H    -2.26807    -3.67107    +0.15973
 H    -1.00210    -4.59544    +0.91663
 C    -3.82171    -3.35774    +2.50998
 H    -3.14435    -5.25941    +1.80279
 H    -2.14993    -4.57228    +3.05859
 H    -4.52197    -3.80652    +3.21088
 H    -3.43217    -2.45434    +2.97554
 H    -4.38327    -3.06003    +1.62513
21
 * E =  +3.028  kcal/mol  ;  (105) 180_182_183_097_301
 H    +0.00000    +0.00000    +0.00000
 O    +0.00000    +0.00000    +0.95347
 C    -0.89801    +0.00000    +1.27390
 C    -0.86297    +0.01219    +2.77928
 H    -1.43940    +0.87811    +0.91015
 H    -1.43473    -0.88690    +0.92451
 C    -2.25083    -0.03198    +3.39339
 H    -0.28497    -0.84800    +3.11748
 H    -0.32011    +0.89707    +3.10692
 C    -2.25530    +0.05474    +4.91911
 H    -2.86105    +0.77838    +2.98546
 H    -2.73630    -0.95593    +3.07761
 C    -2.51361    +1.44825    +5.47161
 H    -3.01725    -0.61257    +5.31992
 H    -1.30160    -0.31489    +5.30186
 C    -1.51247    +2.49792    +5.03162
 H    -3.51574    +1.76268    +5.17433
 H    -2.52457    +1.39594    +6.56045
 H    -1.71570    +3.45564    +5.50542
 H    -0.49573    +2.20736    +5.29250
 H    -1.54476    +2.65490    +3.95506
21
 * E =  +3.036  kcal/mol  ;  (106) 060_176_176_262_059
 H    +0.00000    +0.00000    +0.00000
 O    +0.00000    +0.00000    +0.95438
 C    -0.90203    +0.00000    +1.26612
 C    -1.68798    -1.20724    +0.80872
 H    -0.83671    +0.01337    +2.35185
 H    -1.41634    +0.91744    +0.96445
 C    -3.08972    -1.24512    +1.39233
 H    -1.73559    -1.21470    -0.28189
 H    -1.14468    -2.10650    +1.09984
 C    -3.94570    -2.40385    +0.88227
 H    -3.00315    -1.30177    +2.47758
 H    -3.60019    -0.30091    +1.18472
```

```
C −4.89044 −2.03853 −0.25245
H −3.29299 −3.21870 +0.56165
H −4.54227 −2.80394 +1.70100
C −4.20854 −1.49149 −1.49082
H −5.47075 −2.92095 −0.52225
H −5.61004 −1.30394 +0.11327
H −4.92819 −1.30584 −2.28485
H −3.70314 −0.54953 −1.28489
H −3.46571 −2.19052 −1.87325
21
* E = +3.047 kcal/mol ; (107) 067_295_180_263_059
H +0.00000 +0.00000 +0.00000
O +0.00000 +0.00000 +0.95381
C −0.90031 +0.00000 +1.26877
C −1.64116 −1.28163 +0.95735
H −0.83240 +0.14006 +2.34409
H −1.44804 +0.85644 +0.86345
C −1.84723 −1.51619 −0.52951
H −1.10188 −2.11510 +1.40467
H −2.61659 −1.23958 +1.44564
C −2.58959 −2.81006 −0.86279
H −2.39480 −0.66332 −0.93431
H −0.88149 −1.52819 −1.04253
C −1.68986 −3.98092 −1.22762
H −3.22027 −3.08902 −0.01583
H −3.26865 −2.63423 −1.69615
C −0.70098 −4.37851 −0.14968
H −2.31672 −4.83770 −1.47513
H −1.14468 −3.73280 −2.14016
H −0.13498 −5.25916 −0.44460
H +0.01543 −3.58438 +0.05113
H −1.20970 −4.60967 +0.78539
21
* E = +3.049 kcal/mol ; (108) 299_181_178_263_059
H +0.00000 +0.00000 +0.00000
O +0.00000 +0.00000 +0.95436
C −0.90193 +0.00000 +1.26632
C −1.68647 +1.21026 +0.81428
H −1.41788 −0.91560 +0.96163
H −0.83586 −0.01746 +2.35184
C −3.11260 +1.20741 +1.33676
H −1.15714 +2.10776 +1.12983
H −1.71114 +1.23448 −0.27847
C −3.92182 +2.44444 +0.94915
H −3.61119 +0.31156 +0.96538
```

```
H   −3.10140   +1.11022   +2.42541
C   −3.97399   +3.52550   +2.01788
H   −3.51412   +2.86655   +0.02791
H   −4.94491   +2.15178   +0.71647
C   −2.62152   +4.06923   +2.43347
H   −4.59509   +4.34475   +1.65540
H   −4.48421   +3.12547   +2.89590
H   −2.72927   +4.87895   +3.15151
H   −2.00753   +3.30153   +2.90060
H   −2.07163   +4.45675   +1.57678
21
 * E =   +3.052  kcal/mol ; (109) 180_181_185_301_096
H   +0.00000   +0.00000   +0.00000
O   +0.00000   +0.00000   +0.95347
C   −0.89752   +0.00000   +1.27527
C   −0.85824   −0.00299   +2.78099
H   −1.43659   +0.88342   +0.92158
H   −1.43772   −0.88197   +0.91909
C   −2.24533   −0.03888   +3.39217
H   −0.27664   −0.86680   +3.10111
H   −0.31856   +0.88345   +3.11631
C   −2.27801   +0.07974   +4.90821
H   −2.84600   +0.76725   +2.96633
H   −2.73890   −0.96948   +3.10254
C   −1.50622   −1.00866   +5.65311
H   −1.89224   +1.05835   +5.20202
H   −3.32184   +0.06402   +5.21979
C   −0.09235   −0.61220   +6.03857
H   −2.04544   −1.27694   +6.56088
H   −1.48298   −1.91462   +5.04390
H   +0.42071   −1.42385   +6.55008
H   +0.50543   −0.33673   +5.17355
H   −0.10577   +0.24416   +6.71092
21
 * E =   +3.053  kcal/mol ; (110) 296_069_099_300_184
H   +0.00000   +0.00000   +0.00000
O   +0.00000   +0.00000   +0.95429
C   −0.90093   +0.00000   +1.26893
C   −1.65128   +1.26129   +0.89038
H   −1.42832   −0.88798   +0.91292
H   −0.82839   −0.07675   +2.35050
C   −1.95080   +1.39652   −0.60204
H   −2.58606   +1.29810   +1.45358
H   −1.05080   +2.10294   +1.23199
C   −3.35604   +0.97916   −1.00531
```

H −1.79885 +2.42866 −0.91277
H −1.23000 +0.81348 −1.18360
C −3.70735 −0.46626 −0.70739
H −4.07493 +1.62913 −0.50089
H −3.48554 +1.15964 −2.07384
C −5.08439 −0.84475 −1.21463
H −2.95778 −1.12072 −1.15725
H −3.65994 −0.64235 +0.36782
H −5.32467 −1.88061 −0.98749
H −5.85237 −0.21981 −0.76178
H −5.15281 −0.71716 −2.29365
21
∗ E = +3.064 kcal/mol ; (111) 059_179_262_061_064
H +0.00000 +0.00000 +0.00000
O +0.00000 +0.00000 +0.95457
C −0.90218 +0.00000 +1.26646
C −1.69142 −1.19796 +0.78160
H −0.83475 +0.00861 +2.35050
H −1.41598 +0.91974 +0.96984
C −3.12891 −1.23739 +1.29709
H −1.69245 −1.17666 −0.31000
H −1.15981 −2.10638 +1.06854
C −3.33853 −2.12754 +2.51430
H −3.46709 −0.22171 +1.51349
H −3.78503 −1.59761 +0.50654
C −2.55601 −1.73966 +3.75816
H −4.40215 −2.14112 +2.75928
H −3.07723 −3.15037 +2.23844
C −2.94978 −0.39224 +4.33192
H −2.71219 −2.50651 +4.51671
H −1.48694 −1.75610 +3.54056
H −2.39469 −0.17223 +5.24079
H −2.76312 +0.42062 +3.63271
H −4.01047 −0.37150 +4.57826
21
∗ E = +3.072 kcal/mol ; (112) 061_176_175_059_264
H +0.00000 +0.00000 +0.00000
O +0.00000 +0.00000 +0.95436
C −0.90193 +0.00000 +1.26634
C −1.68279 −1.21732 +0.82482
H −0.83647 +0.02635 +2.35175
H −1.42074 +0.91090 +0.95338
C −3.08480 −1.24009 +1.40312
H −1.73709 −1.23506 −0.26742
H −1.12857 −2.10536 +1.12706

```
C   −3.97151   −2.36798   +0.89843
H   −3.01573   −1.30700   +2.49108
H   −3.57464   −0.28805   +1.18975
C   −3.43404   −3.77377   +1.16214
H   −4.94753   −2.25744   +1.36950
H   −4.14086   −2.24322   −0.17344
C   −2.66540   −4.37240   −0.00208
H   −2.80250   −3.75684   +2.05269
H   −4.26351   −4.43834   +1.40067
H   −2.29133   −5.36502   +0.23927
H   −3.30765   −4.46616   −0.87633
H   −1.81398   −3.75995   −0.28806
21
 * E =   +3.075  kcal/mol ; (113) 067_298_185_097_301
H   +0.00000   +0.00000   +0.00000
O   +0.00000   +0.00000   +0.95389
C   −0.90056   +0.00000   +1.26836
C   −1.64562   −1.27676   +0.94713
H   −0.83389   +0.13370   +2.34461
H   −1.44588   +0.86011   +0.86750
C   −1.79088   −1.53411   −0.54346
H   −1.12025   −2.11515   +1.40565
H   −2.62831   −1.22712   +1.41610
C   −2.64731   −2.75332   −0.88456
H   −2.21361   −0.64843   −1.02769
H   −0.79593   −1.66867   −0.96916
C   −4.09375   −2.43227   −1.22885
H   −2.20996   −3.28332   −1.72959
H   −2.62131   −3.45310   −0.04650
C   −4.86277   −1.72018   −0.13370
H   −4.11148   −1.82156   −2.13329
H   −4.60624   −3.36045   −1.48192
H   −5.90245   −1.57391   −0.41743
H   −4.84957   −2.29184   +0.79343
H   −4.44339   −0.73793   +0.07642
21
 * E =   +3.089  kcal/mol ; (114) 180_180_099_298_295
H   +0.00000   +0.00000   +0.00000
O   +0.00000   +0.00000   +0.95353
C   −0.89792   +0.00000   +1.27442
C   −0.85407   −0.00436   +2.78191
H   −1.43200   +0.89151   +0.93226
H   −1.44063   −0.87194   +0.90276
C   −2.23470   +0.00629   +3.43453
H   −0.28544   −0.87550   +3.11006
```

```
H   -0.27880   +0.86780   +3.08775
C   -2.72835   -1.35941   +3.89149
H   -2.21242   +0.65808   +4.30627
H   -2.96046   +0.45856   +2.75514
C   -2.89918   -2.39837   +2.79544
H   -2.02791   -1.74383   +4.63455
H   -3.68173   -1.23465   +4.40843
C   -3.97693   -2.05083   +1.78673
H   -1.94646   -2.55449   +2.28666
H   -3.14417   -3.35369   +3.25947
H   -4.09290   -2.83712   +1.04424
H   -4.93907   -1.91632   +2.27904
H   -3.75438   -1.12876   +1.25296
21
* E =   +3.092  kcal/mol ; (115) 061_302_300_089_063
H   +0.00000   +0.00000   +0.00000
O   +0.00000   +0.00000   +0.95535
C   -0.90258   +0.00000   +1.26847
C   -1.68320   -1.22115   +0.83396
H   -0.83600   +0.02826   +2.35276
H   -1.42075   +0.91170   +0.95876
C   -1.75977   -1.43979   -0.67106
H   -1.23467   -2.10099   +1.29442
H   -2.69503   -1.13414   +1.23452
C   -2.40324   -0.30855   -1.46940
H   -0.76416   -1.65883   -1.06075
H   -2.32844   -2.35365   -0.83657
C   -1.47447   +0.80277   -1.94652
H   -2.88097   -0.73222   -2.35385
H   -3.20920   +0.12859   -0.87679
C   -0.41144   +0.33783   -2.92483
H   -2.08272   +1.56622   -2.43121
H   -1.00226   +1.30673   -1.10309
H   +0.17915   +1.17473   -3.28932
H   +0.28156   -0.37599   -2.48097
H   -0.86337   -0.15052   -3.78667
21
* E =   +3.101  kcal/mol ; (116) 060_179_185_301_096
H   +0.00000   +0.00000   +0.00000
O   +0.00000   +0.00000   +0.95437
C   -0.90208   +0.00000   +1.26593
C   -1.68578   -1.21068   +0.81184
H   -0.83672   +0.01666   +2.35137
H   -1.41824   +0.91522   +0.96073
C   -3.10565   -1.21006   +1.34521
```

H −1.70265 −1.22985 −0.28046
H −1.15873 −2.10954 +1.13421
C −3.91568 −2.45467 +1.01620
H −3.07676 −1.08766 +2.42940
H −3.63175 −0.33521 +0.95630
C −4.07113 −2.74133 −0.47676
H −3.46119 −3.32033 +1.50272
H −4.89940 −2.33939 +1.46965
C −3.06184 −3.73456 −1.02418
H −5.07063 −3.12930 −0.66897
H −4.00694 −1.80331 −1.03248
H −3.19564 −3.88803 −2.09292
H −2.03755 −3.40815 −0.86182
H −3.17428 −4.70135 −0.53638
21
∗ E = +3.123 kcal/mol ; (117) 062_177_099_298_295
H +0.00000 +0.00000 +0.00000
O +0.00000 +0.00000 +0.95440
C −0.90176 +0.00000 +1.26699
C −1.66983 −1.23299 +0.84053
H −0.83490 +0.02984 +2.35235
H −1.41929 +0.90948 +0.95293
C −3.09413 −1.28927 +1.39125
H −1.69804 −1.28125 −0.25183
H −1.09707 −2.09876 +1.16871
C −4.16981 −0.84122 +0.41167
H −3.32199 −2.31089 +1.69026
H −3.15438 −0.69913 +2.30799
C −4.07053 +0.59995 −0.06090
H −4.13663 −1.50079 −0.45711
H −5.14874 −0.99239 +0.87000
C −4.27893 +1.62046 +1.04122
H −3.10434 +0.76336 −0.54198
H −4.81641 +0.76129 −0.83904
H −4.24004 +2.63555 +0.65292
H −5.24987 +1.48438 +1.51529
H −3.52284 +1.54095 +1.81974
21
∗ E = +3.130 kcal/mol ; (118) 066_296_186_300_096
H +0.00000 +0.00000 +0.00000
O +0.00000 +0.00000 +0.95397
C −0.90077 +0.00000 +1.26810
C −1.64720 −1.27401 +0.93523
H −0.83385 +0.12455 +2.34537
H −1.44449 +0.86390 +0.87340

C −1.81130 −1.49405 −0.55695
H −1.11332 −2.11252 +1.38195
H −2.63049 −1.22965 +1.40829
C −2.68404 −2.67887 −0.94236
H −2.23065 −0.58943 −1.00426
H −0.82707 −1.63706 −1.00986
C −2.19240 −4.03035 −0.42554
H −3.70280 −2.50657 −0.58815
H −2.74619 −2.70578 −2.02964
C −2.84385 −4.47156 +0.87262
H −2.38037 −4.79463 −1.17858
H −1.10816 −3.99608 −0.30203
H −2.44804 −5.42742 +1.20894
H −2.68980 −3.75117 +1.67203
H −3.91851 −4.58755 +0.74161
21
* E = +3.153 kcal/mol ; (119) 180_058_062_062_266
H +0.00000 +0.00000 +0.00000
O +0.00000 +0.00000 +0.95329
C −0.89758 +0.00000 +1.27439
C −0.85368 +0.00298 +2.78222
H −1.43414 +0.87862 +0.90753
H −1.43330 −0.88586 +0.92501
C −0.10182 +1.18582 +3.36919
H −1.87498 −0.03261 +3.16221
H −0.37103 −0.91843 +3.10720
C −0.67269 +2.56295 +3.06316
H −0.05946 +1.06246 +4.45253
H +0.92557 +1.14464 +3.01146
C −2.08346 +2.82036 +3.59036
H +0.00672 +3.29438 +3.49971
H −0.64518 +2.74839 +1.98736
C −3.20006 +2.53169 +2.60264
H −2.23873 +2.23831 +4.50127
H −2.16491 +3.86473 +3.89025
H −4.17055 +2.79112 +3.02083
H −3.06850 +3.11573 +1.69267
H −3.24053 +1.48410 +2.31537
21
* E = +3.157 kcal/mol ; (120) 067_295_178_059_263
H +0.00000 +0.00000 +0.00000
O +0.00000 +0.00000 +0.95374
C −0.89943 +0.00000 +1.27098
C −1.64400 −1.28277 +0.96803
H −0.82855 +0.14352 +2.34562

```
H   −1.44983   +0.85434   +0.86500
C   −1.84137   −1.52699   −0.51572
H   −1.10037   −2.11731   +1.41317
H   −2.61451   −1.23060   +1.46359
C   −2.53635   −2.83465   −0.86334
H   −2.41610   −0.69797   −0.93732
H   −0.87149   −1.51796   −1.01729
C   −3.93066   −2.99645   −0.25904
H   −2.59877   −2.89969   −1.94892
H   −1.90814   −3.67050   −0.54857
C   −3.95574   −3.79952   +1.02886
H   −4.36744   −2.01002   −0.08920
H   −4.58303   −3.48700   −0.98027
H   −4.96283   −3.86696   +1.43483
H   −3.60290   −4.81437   +0.85305
H   −3.31805   −3.36347   +1.79402
21
 * E =  +3.179 kcal/mol ; (121) 065_060_177_059_263
H   +0.00000   +0.00000   +0.00000
O   +0.00000   +0.00000   +0.95449
C   −0.90194   +0.00000   +1.26682
C   −1.65002   −1.26608   +0.90923
H   −0.83343   +0.09950   +2.34812
H   −1.43916   +0.87499   +0.89313
C   −1.01464   −2.51067   +1.49705
H   −2.68111   −1.16979   +1.25676
H   −1.70118   −1.34856   −0.17930
C   −1.76312   −3.80522   +1.22093
H   +0.00437   −2.59395   +1.11882
H   −0.92304   −2.37795   +2.57662
C   −1.93940   −4.13957   −0.26036
H   −1.22256   −4.61205   +1.71438
H   −2.74498   −3.76800   +1.69859
C   −3.28567   −3.72924   −0.82946
H   −1.13636   −3.67125   −0.83337
H   −1.81929   −5.21203   −0.40838
H   −3.35723   −3.96121   −1.89008
H   −4.09157   −4.25704   −0.32216
H   −3.47177   −2.66447   −0.70963
21
 * E =  +3.193 kcal/mol ; (122) 183_283_059_267_177
H   +0.00000   +0.00000   +0.00000
O   +0.00000   +0.00000   +0.95366
C   −0.89788   +0.00000   +1.27503
C   −0.87802   +0.07869   +2.78236
```

```
H    −1.43786    +0.86110    +0.87507
H    −1.42587    −0.90326    +0.95286
C    −0.53396    −1.20315    +3.52663
H    −0.17525    +0.86629    +3.05717
H    −1.86308    +0.41265    +3.10972
C    +0.82941    −1.81452    +3.21467
H    −0.59514    −0.97990    +4.59150
H    −1.31164    −1.94713    +3.33629
C    +0.79742    −2.86456    +2.11876
H    +1.52710    −1.02517    +2.93386
H    +1.23107    −2.27912    +4.11663
C    +2.17547    −3.39658    +1.78375
H    +0.15225    −3.68647    +2.43554
H    +0.34722    −2.44020    +1.22293
H    +2.13447    −4.16888    +1.01859
H    +2.81782    −2.59769    +1.41756
H    +2.65445    −3.82714    +2.66228
21
* E =   +3.194  kcal/mol ; (123) 177_297_286_262_060
H    +0.00000    +0.00000    +0.00000
O    +0.00000    +0.00000    +0.95323
C    −0.89716    +0.00000    +1.27534
C    −0.85421    −0.08522    +2.78116
H    −1.41406    +0.91396    +0.97408
H    −1.45209    −0.84703    +0.86294
C    −0.21871    −1.37165    +3.28970
H    −0.29046    +0.77339    +3.14508
H    −1.87155    +0.01787    +3.15841
C    −1.09262    −2.61601    +3.13451
H    +0.72219    −1.50223    +2.75908
H    +0.04202    −1.24986    +4.34240
C    −1.84286    −3.01183    +4.39713
H    −1.81286    −2.46459    +2.32677
H    −0.47796    −3.46044    +2.82554
C    −2.79202    −1.95133    +4.91916
H    −2.39970    −3.92919    +4.20456
H    −1.11660    −3.25606    +5.17449
H    −3.33511    −2.30506    +5.79267
H    −2.25978    −1.04782    +5.21095
H    −3.52599    −1.67368    +4.16326
21
* E =   +3.311  kcal/mol ; (124) 295_299_285_261_060
H    +0.00000    +0.00000    +0.00000
O    +0.00000    +0.00000    +0.95449
C    −0.90179    +0.00000    +1.26725
```

```
C  −1.65040  +1.26499  +0.90195
H  −1.43922  −0.87503  +0.89460
H  −0.83106  −0.10374  +2.34754
C  −1.01941  +2.52046  +1.48692
H  −1.68772  +1.34227  −0.18733
H  −2.68376  +1.16225  +1.23508
C  −1.25841  +2.70378  +2.98551
H  +0.04892  +2.47714  +1.28199
H  −1.39580  +3.39698  +0.95682
C  −2.39555  +3.65615  +3.32180
H  −1.45662  +1.73469  +3.44930
H  −0.35094  +3.07535  +3.45897
C  −3.74614  +3.23570  +2.77663
H  −2.46131  +3.75816  +4.40517
H  −2.14769  +4.64782  +2.93891
H  −4.52739  +3.92614  +3.08634
H  −3.74759  +3.21049  +1.68834
H  −4.02207  +2.24317  +3.13137
21
 * E =  +3.324  kcal/mol ; (125) 295_064_091_298_300
H  +0.00000  +0.00000  +0.00000
O  +0.00000  +0.00000  +0.95439
C  −0.90087  +0.00000  +1.26950
C  −1.65049  +1.27060  +0.91561
H  −1.42772  −0.88696  +0.91421
H  −0.82793  −0.08456  +2.35058
C  −1.85124  +1.53206  −0.57443
H  −2.62189  +1.25134  +1.41452
H  −1.09262  +2.09692  +1.35417
C  −3.13385  +0.97210  −1.17297
H  −1.85773  +2.60841  −0.73956
H  −0.98889  +1.17303  −1.14145
C  −3.29801  −0.53864  −1.12807
H  −3.98056  +1.43225  −0.66090
H  −3.19771  +1.29654  −2.21323
C  −2.20639  −1.29605  −1.85824
H  −3.35602  −0.87575  −0.09274
H  −4.26094  −0.79043  −1.57230
H  −2.40164  −2.36565  −1.86401
H  −2.12889  −0.96669  −2.89343
H  −1.23031  −1.15227  −1.39714
21
 * E =  +3.346  kcal/mol ; (126) 062_057_063_061_266
H  +0.00000  +0.00000  +0.00000
O  +0.00000  +0.00000  +0.95462
```

```
C    −0.90218   +0.00000   +1.26665
C    −1.66894   −1.23586   +0.84598
H    −0.83159   +0.05808   +2.34984
H    −1.42750   +0.89751   +0.93077
C    −1.02745   −2.54004   +1.28879
H    −2.68935   −1.16236   +1.22474
H    −1.75952   −1.23723   −0.24306
C    −0.91219   −2.74779   +2.79212
H    −1.60012   −3.36770   +0.86743
H    −0.03064   −2.60010   +0.85335
C    −2.23868   −2.77419   +3.55011
H    −0.39672   −3.69533   +2.94479
H    −0.25782   −1.98879   +3.22456
C    −2.66290   −1.44148   +4.14142
H    −3.02346   −3.15117   +2.89065
H    −2.16764   −3.49401   +4.36483
H    −3.58443   −1.54049   +4.71138
H    −1.89852   −1.06028   +4.81696
H    −2.83259   −0.68378   +3.38056
21
* E =  +3.383 kcal/mol ; (127) 297_285_060_268_178
H    +0.00000   +0.00000   +0.00000
O    +0.00000   +0.00000   +0.95427
C    −0.90271   +0.00000   +1.26370
C    −1.68653   +1.23557   +0.87155
H    −1.42042   −0.89265   +0.90451
H    −0.84220   −0.08016   +2.34771
C    −1.37223   +2.51695   +1.62918
H    −1.55162   +1.40319   −0.20085
H    −2.74583   +1.00990   +1.00449
C    +0.06281   +3.02784   +1.53611
H    −2.05182   +3.28347   +1.25732
H    −1.63214   +2.37806   +2.68132
C    +0.97527   +2.53265   +2.64363
H    +0.48942   +2.75347   +0.56979
H    +0.05564   +4.11867   +1.56544
C    +2.39776   +3.02907   +2.48677
H    +0.57377   +2.86505   +3.60293
H    +0.97595   +1.44517   +2.65868
H    +3.03165   +2.69365   +3.30445
H    +2.83403   +2.66245   +1.55927
H    +2.43588   +4.11759   +2.46260
21
* E =  +3.418 kcal/mol ; (128) 180_182_297_300_093
H    +0.00000   +0.00000   +0.00000
```

```
 O    +0.00000    +0.00000    +0.95353
 C    −0.89792    +0.00000    +1.27439
 C    −0.85155    +0.01115    +2.78031
 H    −1.43829    +0.87318    +0.90120
 H    −1.43337    −0.89095    +0.93277
 C    −2.22290    −0.02539    +3.43212
 H    −0.27421    −0.85510    +3.10121
 H    −0.29190    +0.88614    +3.10952
 C    −3.13307    +1.16669    +3.16333
 H    −2.73903    −0.93658    +3.12356
 H    −2.08546    −0.11144    +4.51062
 C    −2.58164    +2.51803    +3.61328
 H    −3.39091    +1.21752    +2.10350
 H    −4.07234    +0.97576    +3.68175
 C    −1.82403    +3.28602    +2.54458
 H    −3.40817    +3.14118    +3.95327
 H    −1.94163    +2.37226    +4.48591
 H    −1.50815    +4.25959    +2.91351
 H    −0.93337    +2.76279    +2.20677
 H    −2.45629    +3.45571    +1.67386
21
 * E =   +3.448  kcal/mol ; (129) 058_179_296_300_094
 H    +0.00000    +0.00000    +0.00000
 O    +0.00000    +0.00000    +0.95449
 C    −0.90221    +0.00000    +1.26604
 C    −1.69494    −1.18511    +0.76235
 H    −0.83303    −0.01495    +2.35052
 H    −1.40965    +0.92945    +0.98972
 C    −3.12502    −1.22296    +1.27475
 H    −1.71536    −1.15017    −0.32945
 H    −1.16843    −2.10215    +1.02607
 C    −3.29084    −1.39345    +2.77958
 H    −3.63777    −0.31021    +0.96529
 H    −3.64685    −2.04235    +0.77902
 C    −2.68105    −2.67041    +3.35526
 H    −2.88306    −0.52903    +3.30701
 H    −4.36037    −1.38263    +2.98719
 C    −1.26997    −2.51384    +3.89384
 H    −3.31340    −3.03061    +4.16594
 H    −2.70119    −3.45327    +2.59412
 H    −0.91203    −3.44469    +4.32857
 H    −0.55906    −2.21990    +3.12604
 H    −1.23980    −1.75535    +4.67481
21
 * E =   +3.516  kcal/mol ; (130) 067_300_300_297_093
```

```
H    +0.00000    +0.00000    +0.00000
O    +0.00000    +0.00000    +0.95426
C    −0.90126    +0.00000    +1.26785
C    −1.63685    −1.28397    +0.94527
H    −0.83698    +0.12837    +2.34498
H    −1.44195    +0.86391    +0.87353
C    −1.72858    −1.59125    −0.54104
H    −1.12172    −2.10583    +1.44162
H    −2.63545    −1.23582    +1.38104
C    −2.43754    −0.55737    −1.40614
H    −0.72105    −1.74961    −0.93122
H    −2.23563    −2.54878    −0.66565
C    −3.91665    −0.34397    −1.08944
H    −1.91913    +0.40393    −1.35777
H    −2.34277    −0.88581    −2.44066
C    −4.21080    +0.76998    −0.10072
H    −4.44410    −0.11735    −2.01533
H    −4.34362    −1.28124    −0.72709
H    −5.28285    +0.90801    +0.02243
H    −3.79404    +0.57523    +0.88369
H    −3.79716    +1.71478    −0.45068
21
* E =  +3.531 kcal/mol ; (131) 177_175_060_264_287
H    +0.00000    +0.00000    +0.00000
O    +0.00000    +0.00000    +0.95351
C    −0.89820    +0.00000    +1.27355
C    −0.84897    −0.07695    +2.77750
H    −1.42088    +0.91121    +0.96540
H    −1.44912    −0.85256    +0.86852
C    −2.20440    +0.02825    +3.45763
H    −0.36763    −1.01519    +3.05528
H    −0.19724    +0.72227    +3.12877
C    −3.19779    −1.06470    +3.06346
H    −2.03596    +0.00095    +4.53275
H    −2.63681    +1.00965    +3.25279
C    −4.17370    −0.66827    +1.96298
H    −2.64222    −1.95367    +2.75886
H    −3.78075    −1.36460    +3.93517
C    −5.23472    +0.30876    +2.43235
H    −3.63136    −0.23564    +1.12159
H    −4.66226    −1.56403    +1.57953
H    −5.90901    +0.58136    +1.62374
H    −5.83339    −0.12484    +3.23185
H    −4.79590    +1.22828    +2.81476
21
```

```
 * E =  +3.554  kcal/mol ; (132) 059_181_300_097_072
 H    +0.00000    +0.00000    +0.00000
 O    +0.00000    +0.00000    +0.95451
 C    −0.90226    +0.00000    +1.26597
 C    −1.68717    −1.19338    +0.77014
 H    −0.83201    −0.01290    +2.35057
 H    −1.41135    +0.92778    +0.98514
 C    −3.13883    −1.22360    +1.22352
 H    −1.65629    −1.20039    −0.32212
 H    −1.17738    −2.09929    +1.09998
 C    −3.32050    −1.26752    +2.74095
 H    −3.66293    −0.35739    +0.81487
 H    −3.61074    −2.09345    +0.76996
 C    −3.58783    +0.08559    +3.38734
 H    −4.15001    −1.92922    +2.99278
 H    −2.43436    −1.71979    +3.19004
 C    −4.97838    +0.61834    +3.09902
 H    −3.45963    −0.00388    +4.46592
 H    −2.84595    +0.81171    +3.05391
 H    −5.13620    +1.58764    +3.56616
 H    −5.15179    +0.73943    +2.03140
 H    −5.73981    −0.06109    +3.47884
21
 * E =  +3.608  kcal/mol ; (133) 060_176_285_261_060
 H    +0.00000    +0.00000    +0.00000
 O    +0.00000    +0.00000    +0.95442
 C    −0.90182    +0.00000    +1.26688
 C    −1.68922    −1.20693    +0.80486
 H    −0.83386    +0.01878    +2.35177
 H    −1.41845    +0.91552    +0.96285
 C    −3.09113    −1.24757    +1.39731
 H    −1.75187    −1.18553    −0.28543
 H    −1.13247    −2.10483    +1.07026
 C    −3.14280    −1.67063    +2.86662
 H    −3.54227    −0.26128    +1.27819
 H    −3.71295    −1.92576    +0.81140
 C    −3.50312    −3.13359    +3.07528
 H    −2.18007    −1.47123    +3.34178
 H    −3.87006    −1.06070    +3.40081
 C    −2.53764    −4.11150    +2.43533
 H    −3.55994    −3.33260    +4.14550
 H    −4.50598    −3.30783    +2.68103
 H    −2.81715    −5.13938    +2.65480
 H    −2.51802    −4.00395    +1.35234
 H    −1.52294    −3.95845    +2.80062
```

21
∗ E = +3.653 kcal/mol ; (134) 180_183_076_099_300

H	+0.00000	+0.00000	+0.00000
O	+0.00000	+0.00000	+0.95351
C	−0.89829	+0.00000	+1.27329
C	−0.86212	−0.00872	+2.78032
H	−1.43314	+0.88935	+0.92729
H	−1.43973	−0.87467	+0.90318
C	−2.25269	−0.07683	+3.39483
H	−0.25713	−0.85516	+3.10294
H	−0.34407	+0.89113	+3.10925
C	−2.90943	−1.45727	+3.32941
H	−2.19988	+0.23988	+4.43734
H	−2.88610	+0.66056	+2.89922
C	−2.79540	−2.25657	+4.61842
H	−3.96584	−1.35371	+3.08462
H	−2.46972	−2.03589	+2.51403
C	−1.37076	−2.53575	+5.05460
H	−3.31565	−1.71770	+5.41252
H	−3.32616	−3.20091	+4.49585
H	−1.34814	−3.14516	+5.95519
H	−0.81924	−3.06739	+4.28020
H	−0.83049	−1.61583	+5.27019

21
∗ E = +3.654 kcal/mol ; (135) 068_300_094_071_066

H	+0.00000	+0.00000	+0.00000
O	+0.00000	+0.00000	+0.95436
C	−0.90219	+0.00000	+1.26559
C	−1.64703	−1.28566	+0.97418
H	−0.84320	+0.16392	+2.33836
H	−1.45015	+0.84493	+0.83743
C	−1.74032	−1.61219	−0.51502
H	−1.16337	−2.10261	+1.50914
H	−2.64516	−1.18477	+1.40105
C	−0.63876	−2.53148	−1.03135
H	−2.70667	−2.06283	−0.73577
H	−1.72872	−0.67863	−1.08468
C	−0.75052	−3.97611	−0.56560
H	−0.64801	−2.51945	−2.12282
H	+0.33190	−2.14390	−0.72251
C	−1.97247	−4.70236	−1.09442
H	+0.14471	−4.50892	−0.88501
H	−0.74513	−4.01574	+0.52408
H	−1.96664	−5.74774	−0.79473
H	−2.89858	−4.26704	−0.72549

H −2.00461 −4.67097 −2.18271
21
∗ E = +3.664 kcal/mol ; (136) 062_174_064_060_266
H +0.00000 +0.00000 +0.00000
O +0.00000 +0.00000 +0.95444
C −0.90191 +0.00000 +1.26671
C −1.66672 −1.23268 +0.83954
H −0.83618 +0.03448 +2.35219
H −1.41771 +0.90874 +0.94746
C −3.06181 −1.32425 +1.43575
H −1.72477 −1.27001 −0.25095
H −1.08708 −2.10416 +1.14197
C −4.04512 −0.23109 +1.03600
H −3.49187 −2.28721 +1.15763
H −2.97675 −1.33805 +2.52381
C −4.33704 −0.13845 −0.46045
H −4.97542 −0.42796 +1.56782
H −3.70245 +0.74001 +1.39805
C −3.47479 +0.85686 −1.21587
H −4.23681 −1.12897 −0.90909
H −5.37915 +0.14567 −0.60195
H −3.76044 +0.90784 −2.26436
H −3.58320 +1.85597 −0.79658
H −2.41875 +0.60144 −1.17893
21
∗ E = +3.666 kcal/mol ; (137) 292_064_071_099_300
H +0.00000 +0.00000 +0.00000
O +0.00000 +0.00000 +0.95383
C −0.89989 +0.00000 +1.27003
C −1.63589 +1.28958 +0.96972
H −1.44531 −0.85920 +0.87055
H −0.83065 −0.13950 +2.34534
C −1.83001 +1.56129 −0.51605
H −2.60404 +1.25337 +1.46998
H −1.07379 +2.10717 +1.41997
C −2.83780 +0.64408 −1.21056
H −2.14113 +2.59832 −0.65057
H −0.85936 +1.49855 −1.01107
C −4.21659 +1.26174 −1.38872
H −2.46447 +0.36095 −2.19403
H −2.93705 −0.28854 −0.65100
C −4.89752 +1.65612 −0.09308
H −4.12700 +2.14083 −2.02932
H −4.85009 +0.55752 −1.92806
H −5.89663 +2.04402 −0.27755

H −4.99223 +0.80276 +0.57744
H −4.34076 +2.42974 +0.43246
21
∗ E = +3.688 kcal/mol ; (138) 300_187_300_097_072
H +0.00000 +0.00000 +0.00000
O +0.00000 +0.00000 +0.95449
C −0.90218 +0.00000 +1.26613
C −1.67296 +1.21604 +0.80383
H −1.41089 −0.91790 +0.96121
H −0.83775 −0.01210 +2.35249
C −3.06628 +1.34746 +1.39979
H −1.08278 +2.09820 +1.05027
H −1.75405 +1.18929 −0.28652
C −4.00373 +0.18131 +1.08501
H −2.98157 +1.47032 +2.48109
H −3.49773 +2.27686 +1.03213
C −4.09361 −0.87442 +2.17937
H −5.01024 +0.55834 +0.89984
H −3.68694 −0.28586 +0.15042
C −4.86401 −0.40365 +3.39817
H −4.57754 −1.76422 +1.77686
H −3.09293 −1.18472 +2.48117
H −4.90391 −1.17489 +4.16379
H −4.41071 +0.47779 +3.84752
H −5.88818 −0.14566 +3.13318
21
∗ E = +3.706 kcal/mol ; (139) 299_180_284_261_060
H +0.00000 +0.00000 +0.00000
O +0.00000 +0.00000 +0.95438
C −0.90157 +0.00000 +1.26745
C −1.68090 +1.21860 +0.82327
H −1.41509 −0.91633 +0.96400
H −0.83437 −0.01746 +2.35300
C −3.11567 +1.21038 +1.33259
H −1.15453 +2.10327 +1.17927
H −1.67584 +1.26841 −0.26783
C −4.04933 +0.25305 +0.58890
H −3.09883 +0.96316 +2.39490
H −3.52491 +2.21988 +1.27505
C −4.91749 +0.92881 −0.46132
H −3.46453 −0.53652 +0.11181
H −4.70344 −0.25118 +1.29885
C −4.13944 +1.62168 −1.56250
H −5.58054 +0.18474 −0.90279
H −5.56431 +1.65568 +0.03325

```
H   -4.80763   +2.05053   -2.30570
H   -3.52591   +2.43107   -1.17103
H   -3.47860   +0.92373   -2.07563
21
* E =  +4.078 kcal/mol ; (140) 177_076_299_095_073
H   +0.00000   +0.00000   +0.00000
O   +0.00000   +0.00000   +0.95362
C   -0.89826   +0.00000   +1.27380
C   -0.88364   -0.07709   +2.78137
H   -1.42552   +0.90342   +0.95043
H   -1.43853   -0.86080   +0.87333
C   -0.52974   +1.20295   +3.52391
H   -1.87400   -0.39959   +3.10443
H   -0.19089   -0.87197   +3.06099
C   +0.85882   +1.77384   +3.23823
H   -1.28946   +1.95754   +3.30886
H   -0.62345   +0.99174   +4.58875
C   +0.88476   +2.85156   +2.16381
H   +1.26883   +2.20138   +4.15482
H   +1.52265   +0.96109   +2.94877
C   +0.29027   +4.16981   +2.62192
H   +1.91610   +3.01660   +1.85244
H   +0.36110   +2.49254   +1.27896
H   +0.31212   +4.91348   +1.82809
H   -0.74674   +4.06004   +2.93479
H   +0.84413   +4.57326   +3.46853
21
* E =  +4.123 kcal/mol ; (141) 178_298_087_095_300
H   +0.00000   +0.00000   +0.00000
O   +0.00000   +0.00000   +0.95342
C   -0.89803   +0.00000   +1.27366
C   -0.88616   -0.04962   +2.78087
H   -1.42419   +0.89775   +0.93970
H   -1.43967   -0.86881   +0.88764
C   -0.23991   -1.30865   +3.35120
H   -0.38125   +0.84074   +3.15332
H   -1.92391   +0.01841   +3.10898
C   +1.27723   -1.23916   +3.52761
H   -0.69387   -1.53958   +4.31698
H   -0.49593   -2.14433   +2.69845
C   +1.71854   -0.83892   +4.92707
H   +1.71001   -2.21381   +3.30389
H   +1.69109   -0.55039   +2.79312
C   +1.22892   +0.52363   +5.37774
H   +1.37404   -1.59407   +5.63678
```

```
 H    +2.80784    -0.85909    +4.97023
 H    +1.61743    +0.77535    +6.36221
 H    +1.54578    +1.30315    +4.68602
 H    +0.14240    +0.55797    +5.43869
21
 * E =   +4.327  kcal/mol ; (142) 064_075_299_093_071
 H    +0.00000    +0.00000    +0.00000
 O    +0.00000    +0.00000    +0.95423
 C    -0.90258    +0.00000    +1.26390
 C    -1.67939    -1.24718    +0.89437
 H    -0.84253    +0.09913    +2.34647
 H    -1.42605    +0.88327    +0.89005
 C    -1.35636    -2.51044    +1.67827
 H    -2.73981    -1.02501    +1.02450
 H    -1.54418    -1.43449    -0.17474
 C    +0.07838    -3.02353    +1.56935
 H    -1.60718    -2.34382    +2.72753
 H    -2.04178    -3.28527    +1.33569
 C    +1.02379    -2.52086    +2.65129
 H    +0.06957    -4.11419    +1.60994
 H    +0.47820    -2.75883    +0.59062
 C    +0.74154    -3.11400    +4.01858
 H    +2.04534    -2.76963    +2.36393
 H    +0.98759    -1.43438    +2.69440
 H    +1.44154    -2.74005    +4.76251
 H    -0.26061    -2.87351    +4.36920
 H    +0.82783    -4.19976    +3.99968
21
 * E =   +4.387  kcal/mol ; (143) 066_300_088_098_300
 H    +0.00000    +0.00000    +0.00000
 O    +0.00000    +0.00000    +0.95457
 C    -0.90185    +0.00000    +1.26741
 C    -1.66350    -1.26612    +0.93656
 H    -0.83802    +0.12792    +2.34478
 H    -1.44107    +0.86492    +0.86879
 C    -1.77413    -1.53348    -0.56253
 H    -1.18263    -2.10859    +1.43231
 H    -2.65819    -1.16841    +1.37326
 C    -0.61471    -2.33619    -1.16084
 H    -2.70456    -2.06278    -0.77256
 H    -1.87196    -0.57432    -1.07721
 C    -0.91188    -3.82075    -1.30891
 H    -0.36224    -1.94239    -2.14560
 H    +0.27406    -2.21449    -0.54000
 C    -1.22101    -4.52615    -0.00306
```

```
 H   −1.75324   −3.94418   −1.99338
 H   −0.05931   −4.30450   −1.78556
 H   −1.38834   −5.58899   −0.16167
 H   −0.39909   −4.42121   +0.70380
 H   −2.11520   −4.12229   +0.46856
21
 * E =  +4.473  kcal/mol ; (144) 182_283_067_088_299
 H   +0.00000   +0.00000   +0.00000
 O   +0.00000   +0.00000   +0.95369
 C   −0.89889   +0.00000   +1.27231
 C   −0.89580   +0.04155   +2.78193
 H   −1.43023   +0.87425   +0.88935
 H   −1.42985   −0.89155   +0.92509
 C   −0.56147   −1.27519   +3.47293
 H   −0.22080   +0.83780   +3.08710
 H   −1.89489   +0.34448   +3.09785
 C   +0.85533   −1.81956   +3.30084
 H   −0.77141   −1.16957   +4.53998
 H   −1.27210   −2.01946   +3.10829
 C   +1.86649   −1.33185   +4.32755
 H   +0.81412   −2.90678   +3.37283
 H   +1.21339   −1.58876   +2.29979
 C   +2.08933   +0.16843   +4.34974
 H   +1.55614   −1.66354   +5.32073
 H   +2.81672   −1.82846   +4.12905
 H   +2.93398   +0.42846   +4.98439
 H   +2.28794   +0.54915   +3.34892
 H   +1.22099   +0.69604   +4.73889
21
 * E =  +4.557  kcal/mol ; (145) 061_068_293_291_087
 H   +0.00000   +0.00000   +0.00000
 O   +0.00000   +0.00000   +0.95441
 C   −0.90189   +0.00000   +1.26664
 C   −1.71183   −1.20618   +0.83925
 H   −0.83255   +0.03071   +2.35237
 H   −1.41148   +0.91409   +0.95101
 C   −1.28376   −2.53047   +1.45390
 H   −2.74528   −1.01416   +1.13353
 H   −1.72864   −1.26873   −0.24976
 C   +0.07741   −3.10100   +1.07398
 H   −1.31260   −2.41430   +2.53929
 H   −2.04317   −3.27810   +1.21594
 C   +0.22385   −3.58862   −0.36460
 H   +0.86016   −2.37794   +1.29712
 H   +0.24882   −3.95303   +1.73239
```

```
 C   +0.63344   -2.53967   -1.38383
 H   +0.98069   -4.37270   -0.38072
 H   -0.70432   -4.06824   -0.68238
 H   +0.84498   -2.99770   -2.34789
 H   -0.14144   -1.79545   -1.55137
 H   +1.53465   -2.02156   -1.05931
21
 * E =  +4.639  kcal/mol ; (146) 066_059_271_265_060
 H   +0.00000   +0.00000   +0.00000
 O   +0.00000   +0.00000   +0.95444
 C   -0.90181   +0.00000   +1.26701
 C   -1.66700   -1.26172   +0.92892
 H   -0.82952   +0.10461   +2.34768
 H   -1.43905   +0.87510   +0.89238
 C   -1.03540   -2.53240   +1.49035
 H   -2.67632   -1.13784   +1.32439
 H   -1.77669   -1.33898   -0.15453
 C   +0.00249   -3.20199   +0.58994
 H   -0.57964   -2.28505   +2.44927
 H   -1.81950   -3.25847   +1.71272
 C   -0.55648   -4.31889   -0.27776
 H   +0.47911   -2.44945   -0.03781
 H   +0.79976   -3.61683   +1.20506
 C   -1.65410   -3.88971   -1.23167
 H   +0.25959   -4.76393   -0.84747
 H   -0.93794   -5.10967   +0.37106
 H   -1.98182   -4.72019   -1.85293
 H   -2.52690   -3.51897   -0.69698
 H   -1.31154   -3.09637   -1.89551
21
 * E =  +4.893  kcal/mol ; (147) 182_185_094_297_089
 H   +0.00000   +0.00000   +0.00000
 O   +0.00000   +0.00000   +0.95358
 C   -0.89744   +0.00000   +1.27594
 C   -0.85297   +0.05058   +2.78289
 H   -1.44485   +0.87123   +0.90484
 H   -1.43128   -0.88995   +0.93527
 C   -2.23061   -0.05974   +3.42865
 H   -0.19479   -0.73890   +3.14457
 H   -0.37804   +0.99010   +3.06093
 C   -2.67524   -1.45457   +3.84885
 H   -2.25334   +0.56997   +4.31694
 H   -2.98025   +0.36205   +2.75363
 C   -2.83398   -2.49291   +2.74177
 H   -1.97819   -1.83916   +4.59692
```

```
 H   −3.63069   −1.34488   +4.36202
 C   −1.57416   −3.27534   +2.41404
 H   −3.59987   −3.20706   +3.04382
 H   −3.22376   −2.00987   +1.84341
 H   −1.76519   −4.01730   +1.64135
 H   −0.76132   −2.64323   +2.06654
 H   −1.21722   −3.80553   +3.29555
21
 * E =  +4.909 kcal/mol ; (148) 059_175_266_062_270
 H   +0.00000   +0.00000   +0.00000
 O   +0.00000   +0.00000   +0.95458
 C   −0.90193   +0.00000   +1.26723
 C   −1.69441   −1.19453   +0.77941
 H   −0.83538   +0.00805   +2.35124
 H   −1.41572   +0.92036   +0.97383
 C   −3.09757   −1.27390   +1.37385
 H   −1.76308   −1.12692   −0.30839
 H   −1.13564   −2.10590   +0.99173
 C   −3.25517   −2.12074   +2.62983
 H   −3.45520   −0.26190   +1.58169
 H   −3.77741   −1.67440   +0.62311
 C   −2.47476   −1.67737   +3.86443
 H   −4.31732   −2.13331   +2.87417
 H   −2.99146   −3.15489   +2.39676
 C   −1.07971   −2.26607   +3.98200
 H   −2.42946   −0.58698   +3.89479
 H   −3.03513   −1.96948   +4.75224
 H   −0.59130   −1.93424   +4.89571
 H   −1.12891   −3.35339   +4.01299
 H   −0.43342   −1.99457   +3.15160
21
 * E =  +4.995 kcal/mol ; (149) 298_299_268_063_268
 H   +0.00000   +0.00000   +0.00000
 O   +0.00000   +0.00000   +0.95462
 C   −0.90198   +0.00000   +1.26725
 C   −1.66425   +1.23777   +0.83595
 H   −1.42615   −0.89728   +0.92875
 H   −0.82702   −0.05915   +2.34878
 C   −1.07641   +2.54655   +1.35249
 H   −1.67354   +1.24968   −0.25657
 H   −2.70914   +1.14142   +1.13552
 C   −1.62743   +3.06799   +2.67288
 H   +0.00536   +2.42822   +1.43082
 H   −1.23870   +3.32266   +0.60507
 C   −1.40546   +2.19437   +3.90574
```

```
H   −1.16609   +4.03993   +2.84694
H   −2.69730   +3.26331   +2.56464
C   −2.52812   +1.21349   +4.19432
H   −0.45506   +1.66684   +3.81157
H   −1.29677   +2.83906   +4.77752
H   −2.31685   +0.62230   +5.08290
H   −3.46238   +1.74526   +4.36755
H   −2.70080   +0.52046   +3.37474
21
* E =   +4.998  kcal/mol ; (150) 176_295_267_063_269
H   +0.00000   +0.00000   +0.00000
O   +0.00000   +0.00000   +0.95332
C   −0.89776   +0.00000   +1.27401
C   −0.84465   −0.09567   +2.78052
H   −1.40811   +0.92118   +0.98221
H   −1.45156   −0.83868   +0.84859
C   −0.25790   −1.40253   +3.30406
H   −0.23431   +0.74009   +3.12088
H   −1.84337   +0.06625   +3.18744
C   −1.24283   −2.52332   +3.60830
H   +0.48942   −1.75257   +2.59098
H   +0.28768   −1.19323   +4.22350
C   −2.04700   −3.07599   +2.43348
H   −0.67028   −3.33814   +4.05132
H   −1.93604   −2.19240   +4.38585
C   −3.38194   −2.38996   +2.20318
H   −1.43967   −3.03765   +1.52750
H   −2.24376   −4.13346   +2.60871
H   −3.91138   −2.82847   +1.35966
H   −4.01862   −2.49396   +3.08034
H   −3.27737   −1.32585   +2.00712
21
* E =   +5.067  kcal/mol ; (151) 298_182_266_063_270
H   +0.00000   +0.00000   +0.00000
O   +0.00000   +0.00000   +0.95445
C   −0.90157   +0.00000   +1.26774
C   −1.66802   +1.23592   +0.84595
H   −1.41890   −0.91044   +0.95732
H   −0.83408   −0.02918   +2.35298
C   −3.09872   +1.27904   +1.37654
H   −1.10743   +2.09654   +1.20810
H   −1.67381   +1.31583   −0.24375
C   −4.18853   +0.72945   +0.46569
H   −3.13948   +0.75238   +2.33347
H   −3.35535   +2.31301   +1.60288
```

```
C   −4.10836   −0.75298   +0.11100
H   −5.14171   +0.91880   +0.95898
H   −4.21100   +1.31468   −0.45664
C   −3.31475   −1.06157   −1.14583
H   −3.70454   −1.30943   +0.95862
H   −5.11920   −1.13357   −0.03305
H   −3.30603   −2.12917   −1.35444
H   −3.75488   −0.56119   −2.00681
H   −2.28042   −0.73421   −1.07695
21
 * E =  +5.144 kcal/mol ; (152) 291_071_282_303_100
H   +0.00000   +0.00000   +0.00000
O   +0.00000   +0.00000   +0.95322
C   −0.89862   +0.00000   +1.27121
C   −1.65587   +1.28818   +1.02540
H   −1.45641   −0.84023   +0.84627
H   −0.82269   −0.17592   +2.34119
C   −2.04215   +1.56951   −0.42099
H   −2.57392   +1.22490   +1.61189
H   −1.08899   +2.12165   +1.44048
C   −0.96202   +2.10092   −1.35847
H   −2.47859   +0.66441   −0.85009
H   −2.84499   +2.30804   −0.41501
C   −0.29987   +3.39862   −0.89365
H   −0.18922   +1.35238   −1.54470
H   −1.43538   +2.26036   −2.32692
C   +1.04637   +3.20216   −0.22079
H   −0.16685   +4.06219   −1.74713
H   −0.97822   +3.92149   −0.21660
H   +1.45624   +4.14883   +0.12500
H   +0.98503   +2.52598   +0.62771
H   +1.76224   +2.77198   −0.92009
21
 * E =  +5.517 kcal/mol ; (153) 066_291_267_063_269
H   +0.00000   +0.00000   +0.00000
O   +0.00000   +0.00000   +0.95408
C   −0.90044   +0.00000   +1.26947
C   −1.63895   −1.27800   +0.92100
H   −0.82690   +0.09862   +2.34930
H   −1.43112   +0.87902   +0.89896
C   −1.93360   −1.46614   −0.56474
H   −1.01905   −2.09986   +1.27710
H   −2.56923   −1.32545   +1.48870
C   −3.27902   −0.96114   −1.06830
H   −1.14105   −1.00319   −1.16050
```

H	−1.87547	−2.52786	−0.80071
C	−3.53924	+0.53980	−0.96230
H	−3.35998	−1.25694	−2.11409
H	−4.07570	−1.49660	−0.54665
C	−4.21009	+0.97668	+0.32769
H	−2.60377	+1.08442	−1.10415
H	−4.18010	+0.84271	−1.78979
H	−4.37754	+2.05152	+0.33976
H	−5.17899	+0.49175	+0.43477
H	−3.62536	+0.72733	+1.20912

3.5 Conformers of n-heptanol

```
24
 * E =   +0.000  kcal/mol ; (1) 182_062_181_180_180_180
 H   +0.00000   +0.00000   +0.00000
 O   +0.00000   +0.00000   +0.95329
 C   −0.89717   +0.00000   +1.27555
 C   −0.85630   +0.04238   +2.78149
 H   −1.44114   +0.87019   +0.89645
 H   −1.42521   −0.89665   +0.94286
 C   −0.20843   +1.30032   +3.32513
 H   −1.87736   −0.04445   +3.15637
 H   −0.31383   −0.83505   +3.13567
 C   −0.14157   +1.32523   +4.83934
 H   +0.79455   +1.38714   +2.90880
 H   −0.76489   +2.17233   +2.97284
 C   +0.49569   +2.58495   +5.39187
 H   −1.14855   +1.22101   +5.25074
 H   +0.41874   +0.45527   +5.18903
 C   +0.56817   +2.61074   +6.90635
 H   +1.50226   +2.68982   +4.98108
 H   −0.06399   +3.45620   +5.04369
 C   +1.20724   +3.87412   +7.44636
 H   −0.43799   +2.50558   +7.31549
 H   +1.12768   +1.74048   +7.25304
 H   +1.24858   +3.87031   +8.53317
 H   +2.22520   +3.98613   +7.07674
 H   +0.64885   +4.75705   +7.13944
24
 * E =   +0.230  kcal/mol ; (2) 065_061_183_181_180_180
 H   +0.00000   +0.00000   +0.00000
 O   +0.00000   +0.00000   +0.95451
 C   −0.90218   +0.00000   +1.26622
 C   −1.65509   −1.25840   +0.89431
 H   −0.83379   +0.08773   +2.34846
 H   −1.43549   +0.88121   +0.90179
 C   −1.03718   −2.51774   +1.46804
 H   −2.68708   −1.15869   +1.23629
 H   −1.70520   −1.33642   −0.19550
 C   −1.75201   −3.78342   +1.03780
 H   +0.01096   −2.56566   +1.17276
 H   −1.04128   −2.45139   +2.55826
 C   −1.14621   −5.04398   +1.62292
 H   −2.80454   −3.72290   +1.32473
 H   −1.74080   −3.85158   −0.05258
 C   −1.85581   −6.31210   +1.18943
```

```
H   −0.09352   −5.10450   +1.33800
H   −1.15945   −4.97891   +2.71324
C   −1.24151   −7.56472   +1.78076
H   −2.90728   −6.25014   +1.47458
H   −1.84150   −6.37644   +0.10022
H   −1.76590   −8.46053   +1.45625
H   −0.19862   −7.66608   +1.48496
H   −1.27186   −7.53921   +2.86881
24
 * E =   +0.233  kcal/mol  ;  (3)  180_180_180_180_180_180
H   +0.00000   +0.00000   +0.00000
O   +0.00000   +0.00000   +0.95350
C   −0.89800   +0.00000   +1.27406
C   −0.86134   −0.00088   +2.77960
H   −1.43648   +0.88302   +0.91838
H   −1.43686   −0.88234   +0.91732
C   −2.24492   −0.00065   +3.39863
H   −0.30125   −0.87539   +3.11095
H   −0.30054   +0.87283   +3.11191
C   −2.21726   −0.00155   +4.91473
H   −2.80137   +0.87280   +3.05126
H   −2.80214   −0.87319   +3.05023
C   −3.59765   −0.00150   +5.54103
H   −1.66160   −0.87432   +5.26411
H   −1.66106   +0.87047   +5.26513
C   −3.57208   −0.00244   +7.05720
H   −4.15374   +0.87142   +5.19129
H   −4.15430   −0.87363   +5.19023
C   −4.95716   −0.00246   +7.67149
H   −3.01671   −0.87458   +7.40573
H   −3.01626   +0.86900   +7.40678
H   −4.91400   −0.00311   +8.75815
H   −5.52196   +0.87589   +7.36327
H   −5.52239   −0.88016   +7.36223
24
 * E =   +0.242  kcal/mol  ;  (4)  061_177_180_180_180_180
H   +0.00000   +0.00000   +0.00000
O   +0.00000   +0.00000   +0.95439
C   −0.90195   +0.00000   +1.26638
C   −1.68510   −1.21245   +0.81625
H   −0.83614   +0.01927   +2.35180
H   −1.41845   +0.91426   +0.95935
C   −3.09620   −1.23661   +1.37035
H   −1.72427   −1.23048   −0.27612
H   −1.14492   −2.10839   +1.12282
```

```
C   -3.89457  -2.44540  +0.92196
H   -3.05600  -1.21742  +2.46147
H   -3.62211  -0.32739  +1.07083
C   -5.30533  -2.47259  +1.47630
H   -3.93514  -2.46645  -0.16930
H   -3.37042  -3.35502  +1.22242
C   -6.10584  -3.68025  +1.02841
H   -5.26555  -2.45208  +2.56773
H   -5.82959  -1.56224  +1.17669
C   -7.51370  -3.69662  +1.58864
H   -6.14523  -3.69998  -0.06184
H   -5.58168  -4.58897  +1.32834
H   -8.06806  -4.57047  +1.25445
H   -7.50159  -3.70887  +2.67724
H   -8.06914  -2.81342  +1.27739
24
* E =  +0.244  kcal/mol ; (5) 067_296_183_180_180_180
H   +0.00000  +0.00000  +0.00000
O   +0.00000  +0.00000  +0.95388
C   -0.90040  +0.00000  +1.26878
C   -1.64640  -1.27596  +0.94478
H   -0.83280  +0.12982  +2.34541
H   -1.44571  +0.86150  +0.87128
C   -1.82366  -1.51614  -0.54223
H   -1.11458  -2.11611  +1.39234
H   -2.62670  -1.22956  +1.42285
C   -2.62543  -2.76480  -0.85483
H   -2.31465  -0.65013  -0.99404
H   -0.84566  -1.60384  -1.02143
C   -2.79832  -3.01438  -2.34002
H   -2.13802  -3.62798  -0.39692
H   -3.60800  -2.68748  -0.38431
C   -3.60425  -4.26064  -2.65148
H   -3.28232  -2.14914  -2.79886
H   -1.81557  -3.09506  -2.81025
C   -3.76910  -4.50116  -4.13837
H   -3.12066  -5.12411  -2.19214
H   -4.58605  -4.17906  -2.18251
H   -4.34975  -5.39888  -4.33717
H   -4.27790  -3.66551  -4.61610
H   -2.80244  -4.61779  -4.62554
24
* E =  +0.467  kcal/mol ; (6) 179_055_059_174_179_180
H   +0.00000  +0.00000  +0.00000
O   +0.00000  +0.00000  +0.95330
```

C	−0.89727	+0.00000	+1.27532
C	−0.85528	−0.02031	+2.78357
H	−1.42974	+0.88775	+0.92486
H	−1.43983	−0.87582	+0.91214
C	−0.03817	+1.10942	+3.38724
H	−1.88226	+0.02108	+3.15036
H	−0.44183	−0.97571	+3.10493
C	−0.52321	+2.49755	+3.01587
H	−0.05009	+1.00437	+4.47315
H	+0.99891	+0.99721	+3.07362
C	+0.22396	+3.59826	+3.74348
H	−0.41155	+2.64738	+1.94063
H	−1.59181	+2.58404	+3.23068
C	−0.23377	+4.99327	+3.36426
H	+0.10890	+3.46234	+4.82137
H	+1.29269	+3.50192	+3.53981
C	+0.51819	+6.08313	+4.10122
H	−0.11405	+5.12941	+2.28824
H	−1.30259	+5.08683	+3.56382
H	+0.17376	+7.07388	+3.81361
H	+0.38941	+5.98764	+5.17816
H	+1.58568	+6.03043	+3.89359

24

* E = +0.593 kcal/mol ; (7) 182_063_186_297_185_180

H	+0.00000	+0.00000	+0.00000
O	+0.00000	+0.00000	+0.95327
C	−0.89716	+0.00000	+1.27550
C	−0.85819	+0.05081	+2.78167
H	−1.44320	+0.86684	+0.89157
H	−1.42298	−0.89962	+0.94755
C	−0.21590	+1.31965	+3.31068
H	−1.87961	−0.04729	+3.14915
H	−0.30571	−0.81890	+3.14118
C	−0.03252	+1.33694	+4.81838
H	+0.75177	+1.43895	+2.82688
H	−0.82097	+2.17887	+3.01033
C	−1.32521	+1.28264	+5.61113
H	+0.60487	+0.49987	+5.11230
H	+0.51141	+2.24119	+5.09640
C	−1.11270	+1.41765	+7.10684
H	−1.99187	+2.07853	+5.26889
H	−1.84668	+0.34452	+5.41310
C	−2.40404	+1.35315	+7.89692
H	−0.43694	+0.63019	+7.44449
H	−0.60369	+2.36064	+7.31258

```
H    −2.22659    +1.45398    +8.96519
H    −3.08553    +2.14812    +7.59826
H    −2.91533    +0.40562    +7.73460
24
 * E =   +0.631  kcal/mol ;  (8)  062_054_059_174_179_180
H    +0.00000    +0.00000    +0.00000
O    +0.00000    +0.00000    +0.95458
C    −0.90159    +0.00000    +1.26820
C    −1.67313    −1.23470    +0.85142
H    −0.83017    +0.05688    +2.35118
H    −1.42780    +0.89778    +0.93507
C    −0.99529    −2.54240    +1.22394
H    −2.66374    −1.18419    +1.30773
H    −1.83572    −1.20681    −0.22852
C    −0.74279    −2.70879    +2.71015
H    −1.61269    −3.36901    +0.86922
H    −0.04524    −2.61708    +0.69466
C    −0.20232    −4.07983    +3.06685
H    −0.03372    −1.95119    +3.04646
H    −1.67040    −2.53191    +3.26109
C    +0.07123    −4.25400    +4.54814
H    −0.90969    −4.84521    +2.73894
H    +0.71836    −4.25982    +2.50736
C    +0.61015    −5.62785    +4.89201
H    +0.77965    −3.49074    +4.87350
H    −0.84831    −4.07112    +5.10660
H    +0.79833    −5.72977    +5.95832
H    −0.09307    −6.40768    +4.60400
H    +1.54605    −5.82232    +4.37078
24
 * E =   +0.648  kcal/mol ;  (9)  182_063_181_181_185_297
H    +0.00000    +0.00000    +0.00000
O    +0.00000    +0.00000    +0.95329
C    −0.89753    +0.00000    +1.27454
C    −0.85872    +0.04109    +2.78056
H    −1.44070    +0.87074    +0.89555
H    −1.42539    −0.89620    +0.94035
C    −0.21239    +1.29890    +3.32626
H    −1.88030    −0.04666    +3.15379
H    −0.31625    −0.83638    +3.13470
C    −0.14535    +1.32194    +4.84083
H    +0.79029    +1.38828    +2.90967
H    −0.77042    +2.17076    +2.97578
C    +0.48383    +2.59057    +5.38585
H    −1.15183    +1.20191    +5.24540
```

```
 H    +0.42760    +0.45902    +5.18919
 C    +0.66699    +2.60250    +6.89454
 H    +1.45526    +2.73155    +4.90940
 H    −0.12521    +3.44857    +5.09108
 C    −0.63297    +2.54140    +7.67368
 H    +1.30438    +1.76517    +7.18401
 H    +1.20852    +3.50582    +7.17544
 H    −0.45577    +2.62876    +8.74326
 H    −1.30007    +3.35181    +7.38210
 H    −1.16047    +1.60491    +7.50731
24
 * E =   +0.655  kcal/mol  ;  (10)  182_062_181_180_176_064
 H    +0.00000    +0.00000    +0.00000
 O    +0.00000    +0.00000    +0.95329
 C    −0.89734    +0.00000    +1.27507
 C    −0.85716    +0.03775    +2.78113
 H    −1.44001    +0.87205    +0.89840
 H    −1.42621    −0.89502    +0.93931
 C    −0.20689    +1.29276    +3.32866
 H    −1.87872    −0.04770    +3.15505
 H    −0.31697    −0.84196    +3.13307
 C    −0.14576    +1.31683    +4.84341
 H    +0.79831    +1.37602    +2.91677
 H    −0.75812    +2.16731    +2.97433
 C    +0.50619    +2.57351    +5.38927
 H    −1.15645    +1.23075    +5.25134
 H    +0.39725    +0.43592    +5.18876
 C    +0.49805    +2.67692    +6.90557
 H    +1.53653    +2.62695    +5.03022
 H    −0.00460    +3.44314    +4.97217
 C    +1.29389    +1.58927    +7.60151
 H    +0.89713    +3.64960    +7.19331
 H    −0.53400    +2.65628    +7.26033
 H    +1.32148    +1.74843    +8.67711
 H    +0.86774    +0.60345    +7.42988
 H    +2.32254    +1.57019    +7.24361
24
 * E =   +0.655  kcal/mol  ;  (11)  182_062_181_184_296_184
 H    +0.00000    +0.00000    +0.00000
 O    +0.00000    +0.00000    +0.95329
 C    −0.89714    +0.00000    +1.27563
 C    −0.85595    +0.04407    +2.78157
 H    −1.44116    +0.86989    +0.89593
 H    −1.42506    −0.89700    +0.94376
 C    −0.20256    +1.30023    +3.32329
```

```
 H   -1.87714   -0.03914   +3.15711
 H   -0.31608   -0.83459   +3.13687
 C   -0.13586   +1.31755   +4.83896
 H   +0.79488   +1.38039   +2.89583
 H   -0.76021   +2.17455   +2.97694
 C   +0.41874   +2.60479   +5.42561
 H   -1.13724   +1.14915   +5.23986
 H   +0.47156   +0.47610   +5.18028
 C   +1.86284   +2.89141   +5.05695
 H   -0.20514   +3.44428   +5.10923
 H   +0.33759   +2.56253   +6.51330
 C   +2.40447   +4.12798   +5.74580
 H   +2.47601   +2.02707   +5.31871
 H   +1.95313   +3.01130   +3.97778
 H   +3.43821   +4.32147   +5.46868
 H   +1.82128   +5.00907   +5.48234
 H   +2.36569   +4.02221   +6.82887
24
 * E =  +0.656  kcal/mol ; (12) 181_062_181_175_064_176
 H   +0.00000   +0.00000   +0.00000
 O   +0.00000   +0.00000   +0.95329
 C   -0.89738   +0.00000   +1.27496
 C   -0.85720   +0.03733   +2.78091
 H   -1.44001   +0.87222   +0.89858
 H   -1.42640   -0.89483   +0.93887
 C   -0.20154   +1.28974   +3.32883
 H   -1.87933   -0.04340   +3.15450
 H   -0.32130   -0.84479   +3.13353
 C   -0.15206   +1.30717   +4.84522
 H   +0.80850   +1.36310   +2.92573
 H   -0.74551   +2.16176   +2.96161
 C   +0.59471   +2.49212   +5.43402
 H   -1.17033   +1.29053   +5.24193
 H   +0.32091   +0.38609   +5.18986
 C   -0.03470   +3.84158   +5.14003
 H   +0.66139   +2.36669   +6.51626
 H   +1.62306   +2.48876   +5.06546
 C   +0.68194   +4.98172   +5.83542
 H   -0.03870   +4.02194   +4.06531
 H   -1.08144   +3.82275   +5.45015
 H   +0.22187   +5.94156   +5.61188
 H   +0.66884   +4.85135   +6.91638
 H   +1.72431   +5.03441   +5.52511
24
 * E =  +0.735  kcal/mol ; (13) 181_062_176_064_175_180
```

```
H   +0.00000   +0.00000   +0.00000
O   +0.00000   +0.00000   +0.95330
C   −0.89738   +0.00000   +1.27499
C   −0.85664   +0.03098   +2.78139
H   −1.43838   +0.87503   +0.90248
H   −1.42791   −0.89254   +0.93514
C   −0.20321   +1.28714   +3.32620
H   −1.87946   −0.04789   +3.15486
H   −0.32388   −0.85552   +3.12347
C   −0.22538   +1.38945   +4.84188
H   +0.82462   +1.33199   +2.96746
H   −0.71262   +2.15582   +2.90434
C   +0.56646   +0.30971   +5.55623
H   +0.17134   +2.36332   +5.13369
H   −1.25994   +1.36798   +5.19355
C   +0.62533   +0.51190   +7.05831
H   +0.13472   −0.67081   +5.34936
H   +1.58231   +0.28367   +5.15479
C   +1.40639   −0.57330   +7.77130
H   +1.07131   +1.48469   +7.27166
H   −0.39070   +0.55231   +7.45465
H   +1.43745   −0.40592   +8.84552
H   +0.96066   −1.55209   +7.60157
H   +2.43360   −0.61522   +7.41281
24
 * E =   +0.752  kcal/mol ;  (14)  179_055_054_058_175_179
H   +0.00000   +0.00000   +0.00000
O   +0.00000   +0.00000   +0.95335
C   −0.89738   +0.00000   +1.27521
C   −0.85725   −0.01296   +2.78287
H   −1.43026   +0.88779   +0.92236
H   −1.44003   −0.87640   +0.91370
C   −0.03623   +1.12205   +3.37636
H   −1.88376   +0.02035   +3.14905
H   −0.43939   −0.96378   +3.11184
C   −0.44310   +2.51265   +2.91778
H   −0.10347   +1.06616   +4.46389
H   +1.00855   +0.96124   +3.11807
C   −1.88729   +2.87463   +3.20831
H   +0.20755   +3.24162   +3.40341
H   −0.24933   +2.61144   +1.84779
C   −2.22837   +4.30603   +2.84097
H   −2.55777   +2.20290   +2.66735
H   −2.09420   +2.71474   +4.26966
C   −3.67467   +4.66214   +3.11919
```

```
H    −1.57202    +4.98229    +3.39083
H    −2.00824    +4.46605    +1.78410
H    −3.89298    +5.69348    +2.85171
H    −4.35126    +4.02347    +2.55353
H    −3.91217    +4.53681    +4.17439
24
 * E =   +0.755  kcal/mol ; (15) 065_061_188_297_185_180
H    +0.00000    +0.00000    +0.00000
O    +0.00000    +0.00000    +0.95447
C    −0.90224    +0.00000    +1.26588
C    −1.65218    −1.26437    +0.90608
H    −0.83468    +0.09824    +2.34733
H    −1.43777    +0.87623    +0.89307
C    −1.02442    −2.51116    +1.50019
H    −2.68454    −1.15471    +1.24009
H    −1.69395    −1.35764    −0.18347
C    −1.63754    −3.81061    +1.00805
H    +0.04040    −2.50212    +1.27202
H    −1.10306    −2.46304    +2.58872
C    −3.10305    −3.98660    +1.35955
H    −1.51817    −3.87774    −0.07607
H    −1.07345    −4.64654    +1.42474
C    −3.65508    −5.33939    +0.95249
H    −3.23370    −3.85336    +2.43653
H    −3.69821    −3.20527    +0.88339
C    −5.12112    −5.50841    +1.29607
H    −3.51225    −5.47593    −0.12074
H    −3.07215    −6.12443    +1.43669
H    −5.49331    −6.48549    +0.99683
H    −5.28620    −5.40540    +2.36734
H    −5.72952    −4.75573    +0.79724
24
 * E =   +0.816  kcal/mol ; (16) 065_302_302_186_181_180
H    +0.00000    +0.00000    +0.00000
O    +0.00000    +0.00000    +0.95458
C    −0.90199    +0.00000    +1.26705
C    −1.65251    −1.26637    +0.91008
H    −0.83904    +0.10003    +2.34711
H    −1.43620    +0.87809    +0.89532
C    −1.71040    −1.55680    −0.58133
H    −1.18193    −2.10591    +1.42034
H    −2.66682    −1.18142    +1.30461
C    −2.33796    −0.45386    −1.41455
H    −0.70473    −1.76736    −0.95282
H    −2.27129    −2.47920    −0.73502
```

```
C    −2.50186   −0.83231   −2.87372
H    −3.31303   −0.19427   −0.99561
H    −1.73683   +0.45691   −1.35273
C    −3.11446   +0.26799   −3.71844
H    −1.52882   −1.10576   −3.28853
H    −3.12126   −1.72906   −2.94520
C    −3.27631   −0.12518   −5.17282
H    −4.08559   +0.54048   −3.30231
H    −2.49377   +1.16267   −3.64803
H    −3.71707   +0.67822   −5.75827
H    −2.31529   −0.37231   −5.62084
H    −3.91860   −0.99852   −5.27304
24
 * E =  +0.848  kcal/mol ; (17) 180_179_174_063_175_180
H    +0.00000   +0.00000   +0.00000
O    +0.00000   +0.00000   +0.95348
C    −0.89786   +0.00000   +1.27436
C    −0.86095   +0.00413   +2.78002
H    −1.43746   +0.88146   +0.91624
H    −1.43599   −0.88392   +0.92038
C    −2.24943   +0.02773   +3.39069
H    −0.31869   −0.88068   +3.11570
H    −0.28143   +0.86800   +3.10165
C    −2.26093   −0.10510   +4.90440
H    −2.75497   +0.95409   +3.10865
H    −2.84130   −0.78351   +2.96292
C    −1.56482   +1.02584   +5.63811
H    −3.29615   −0.16026   +5.24506
H    −1.79874   −1.05393   +5.18551
C    −1.69758   +0.92516   +7.14572
H    −0.50575   +1.04425   +5.37704
H    −1.97707   +1.98108   +5.30305
C    −0.99274   +2.04911   +7.87781
H    −2.75551   +0.92004   +7.41316
H    −1.29771   −0.03469   +7.47666
H    −1.10319   +1.95696   +8.95582
H    +0.07258   +2.05461   +7.65345
H    −1.39382   +3.01828   +7.58568
24
 * E =  +0.851  kcal/mol ; (18) 065_060_183_184_296_184
H    +0.00000   +0.00000   +0.00000
O    +0.00000   +0.00000   +0.95448
C    −0.90185   +0.00000   +1.26705
C    −1.65231   −1.26203   +0.90232
H    −0.83229   +0.09317   +2.34875
```

```
H    -1.43709    +0.87845    +0.89890
C    -1.02764    -2.51713    +1.47858
H    -2.68378    -1.16444    +1.24667
H    -1.70515    -1.34483    -0.18704
C    -1.74387    -3.78347    +1.04790
H    +0.01970    -2.55080    +1.18330
H    -1.03481    -2.45089    +2.56933
C    -1.20810    -5.05326    +1.68774
H    -2.80511    -3.68823    +1.28499
H    -1.68514    -3.87725    -0.03927
C    +0.22875    -5.38082    +1.32486
H    -1.29409    -4.97423    +2.77406
H    -1.84250    -5.89199    +1.39564
C    +0.68627    -6.70611    +1.90040
H    +0.32656    -5.39984    +0.23764
H    +0.88975    -4.58888    +1.67572
H    +1.71816    -6.92394    +1.63492
H    +0.61826    -6.70409    +2.98714
H    +0.06953    -7.52598    +1.53555
24
 * E =  +0.866  kcal/mol ; (19) 065_060_182_176_064_175
H    +0.00000    +0.00000    +0.00000
O    +0.00000    +0.00000    +0.95449
C    -0.90208    +0.00000    +1.26643
C    -1.65402    -1.25979    +0.89774
H    -0.83342    +0.09031    +2.34843
H    -1.43610    +0.88002    +0.90014
C    -1.03162    -2.51814    +1.46943
H    -2.68459    -1.16192    +1.24480
H    -1.70944    -1.33837    -0.19180
C    -1.76492    -3.77954    +1.05315
H    +0.01043    -2.57553    +1.15307
H    -1.01468    -2.43576    +2.55710
C    -1.10962    -5.06795    +1.52125
H    -2.79136    -3.74173    +1.42618
H    -1.84099    -3.79810    -0.03560
C    -1.05486    -5.23377    +3.02893
H    -1.65097    -5.91505    +1.09623
H    -0.09585    -5.12410    +1.11801
C    -0.49538    -6.57915    +3.44582
H    -0.44866    -4.44129    +3.46703
H    -2.05957    -5.11150    +3.43844
H    -0.45855    -6.68069    +4.52798
H    -1.10363    -7.39411    +3.05659
H    +0.51607    -6.71565    +3.06650
```

```
24
 * E =   +0.870  kcal/mol  ;  (20)  065_061_183_181_184_297
 H    +0.00000    +0.00000    +0.00000
 O    +0.00000    +0.00000    +0.95449
 C    -0.90200    +0.00000    +1.26665
 C    -1.65524    -1.25891    +0.89715
 H    -0.83298    +0.08914    +2.34874
 H    -1.43571    +0.88067    +0.90147
 C    -1.03805    -2.51751    +1.47317
 H    -2.68702    -1.15794    +1.23940
 H    -1.70585    -1.33896    -0.19250
 C    -1.75572    -3.78376    +1.04819
 H    +0.00975    -2.56779    +1.17702
 H    -1.04079    -2.44820    +2.56330
 C    -1.14859    -5.03797    +1.64881
 H    -2.80794    -3.70880    +1.32745
 H    -1.73585    -3.86236    -0.04191
 C    -1.77654    -6.33379    +1.16260
 H    -0.08133    -5.05352    +1.42223
 H    -1.22573    -4.98760    +2.73746
 C    -3.23907    -6.48716    +1.53417
 H    -1.66751    -6.39979    +0.07861
 H    -1.21455    -7.17202    +1.57432
 H    -3.62209    -7.45953    +1.23296
 H    -3.37878    -6.39623    +2.61070
 H    -3.85933    -5.73189    +1.05682
24
 * E =   +0.870  kcal/mol  ;  (21)  061_177_174_063_175_180
 H    +0.00000    +0.00000    +0.00000
 O    +0.00000    +0.00000    +0.95437
 C    -0.90198    +0.00000    +1.26622
 C    -1.68342    -1.21611    +0.82270
 H    -0.83653    +0.02480    +2.35162
 H    -1.42009    +0.91181    +0.95461
 C    -3.08997    -1.23677    +1.39223
 H    -1.73579    -1.23181    -0.26976
 H    -1.13013    -2.10485    +1.12241
 C    -3.96028    -2.36331    +0.86093
 H    -3.03541    -1.30755    +2.48065
 H    -3.57580    -0.28399    +1.17447
 C    -3.45769    -3.75501    +1.19605
 H    -4.96840    -2.24780    +1.26206
 H    -4.05363    -2.26587    -0.22312
 C    -4.40746    -4.85136    +0.75273
 H    -2.48400    -3.92238    +0.73301
```

H −3.29778 −3.83075 +2.27452
C −3.89939 −6.24117 +1.07850
H −5.37916 −4.69630 +1.22431
H −4.57630 −4.76576 −0.32198
H −4.59691 −7.00942 +0.75305
H −2.94471 −6.43320 +0.59164
H −3.75041 −6.36199 +2.15022
24
* E = +0.874 kcal/mol ; (22) 064_061_183_180_176_063
H +0.00000 +0.00000 +0.00000
O +0.00000 +0.00000 +0.95452
C −0.90231 +0.00000 +1.26588
C −1.65604 −1.25658 +0.88951
H −0.83409 +0.08416 +2.34841
H −1.43448 +0.88296 +0.90406
C −1.03919 −2.51856 +1.45856
H −2.68792 −1.15745 +1.23203
H −1.70639 −1.33041 −0.20059
C −1.75766 −3.78213 +1.02714
H +0.00817 −2.56807 +1.16054
H −1.04018 −2.45471 +2.54897
C −1.14105 −5.04120 +1.60759
H −2.80725 −3.72528 +1.32750
H −1.75692 −3.83580 −0.06270
C −1.89730 −6.31821 +1.28028
H −0.11136 −5.13156 +1.25401
H −1.07950 −4.93385 +2.69180
C −1.94734 −6.64230 −0.20060
H −1.43098 −7.14797 +1.81131
H −2.91381 −6.24228 +1.67045
H −2.42855 −7.60145 −0.37772
H −2.50204 −5.89260 −0.76029
H −0.94414 −6.69321 −0.62215
24
* E = +0.880 kcal/mol ; (23) 180_180_180_180_176_063
H +0.00000 +0.00000 +0.00000
O +0.00000 +0.00000 +0.95349
C −0.89792 +0.00000 +1.27424
C −0.86096 −0.00495 +2.77984
H −1.43586 +0.88435 +0.92099
H −1.43781 −0.88094 +0.91555
C −2.24448 −0.00380 +3.39887
H −0.30266 −0.88162 +3.10858
H −0.29837 +0.86671 +3.11439
C −2.21734 −0.01759 +4.91527

```
H   −2.79693   +0.87522   +3.05908
H   −2.80582   −0.87069   +3.04298
C   −3.60330   −0.00684   +5.53235
H   −1.67710   −0.90323   +5.25800
H   −1.64527   +0.84216   +5.26629
C   −3.61516   −0.12487   +7.04747
H   −4.11984   +0.91033   +5.23932
H   −4.18271   −0.82893   +5.10858
C   −2.94761   +1.03530   +7.76084
H   −4.64848   −0.20406   +7.38503
H   −3.12963   −1.05837   +7.33722
H   −3.04703   +0.94332   +8.83995
H   −1.88484   +1.09086   +7.53649
H   −3.39664   +1.98339   +7.46731
24
* E =   +0.887  kcal/mol ; (24) 067_296_183_181_184_297
H   +0.00000   +0.00000   +0.00000
O   +0.00000   +0.00000   +0.95389
C   −0.90048   +0.00000   +1.26859
C   −1.64673   −1.27538   +0.94288
H   −0.83304   +0.12886   +2.34534
H   −1.44537   +0.86205   +0.87170
C   −1.82347   −1.51349   −0.54452
H   −1.11528   −2.11618   +1.38964
H   −2.62721   −1.22919   +1.42065
C   −2.62846   −2.75968   −0.86008
H   −2.31107   −0.64544   −0.99618
H   −0.84514   −1.60382   −1.02275
C   −2.78544   −3.00398   −2.34923
H   −2.14773   −3.61783   −0.38897
H   −3.61700   −2.67458   −0.40236
C   −3.67455   −4.18593   −2.69909
H   −3.19419   −2.10396   −2.81187
H   −1.79881   −3.15260   −2.79446
C   −3.14112   −5.52160   −2.21763
H   −4.67007   −4.02001   −2.28375
H   −3.80060   −4.22134   −3.78108
H   −3.76971   −6.34154   −2.55720
H   −2.13496   −5.69845   −2.59570
H   −3.09876   −5.57347   −1.13208
24
* E =   +0.889  kcal/mol ; (25) 061_178_185_297_185_180
H   +0.00000   +0.00000   +0.00000
O   +0.00000   +0.00000   +0.95435
C   −0.90192   +0.00000   +1.26631
```

C	−1.68371	−1.21494	+0.82053
H	−0.83608	+0.02362	+2.35164
H	−1.41988	+0.91234	+0.95579
C	−3.10206	−1.21828	+1.36034
H	−1.69914	−1.24369	−0.27091
H	−1.15437	−2.11011	+1.14947
C	−3.88620	−2.48046	+1.04252
H	−3.06684	−1.08959	+2.44337
H	−3.64136	−0.35255	+0.96949
C	−4.11123	−2.72348	−0.43809
H	−3.37222	−3.34184	+1.47446
H	−4.85563	−2.42609	+1.54027
C	−4.99805	−3.92260	−0.71446
H	−4.55957	−1.83298	−0.88623
H	−3.15474	−2.86902	−0.94276
C	−5.21227	−4.17136	−2.19365
H	−4.55611	−4.80784	−0.25440
H	−5.96160	−3.77633	−0.22386
H	−5.85186	−5.03371	−2.36655
H	−5.67965	−3.31182	−2.67140
H	−4.26645	−4.35417	−2.70101

24

∗ E = +0.889 kcal/mol ; (26) 061_177_180_180_184_297

H	+0.00000	+0.00000	+0.00000
O	+0.00000	+0.00000	+0.95436
C	−0.90188	+0.00000	+1.26648
C	−1.68164	−1.21824	+0.82667
H	−0.83568	+0.02795	+2.35169
H	−1.42094	+0.91040	+0.95251
C	−3.09264	−1.24151	+1.38116
H	−1.72056	−1.24577	−0.26547
H	−1.13887	−2.10988	+1.14113
C	−3.88554	−2.46071	+0.95076
H	−3.05280	−1.20589	+2.47194
H	−3.62328	−0.33948	+1.06825
C	−5.29841	−2.47267	+1.50366
H	−3.91103	−2.50009	−0.13910
H	−3.36355	−3.36361	+1.27623
C	−6.08692	−3.73108	+1.18042
H	−5.25197	−2.35245	+2.58731
H	−5.83951	−1.60216	+1.12527
C	−6.33216	−3.94127	−0.30154
H	−5.56262	−4.59659	+1.58913
H	−7.04536	−3.68650	+1.69736
H	−6.96303	−4.80968	−0.47657

H −6.83018 −3.07749 −0.74034
H −5.40455 −4.09919 −0.84707
24
∗ E = +0.891 kcal/mol ; (27) 061_177_179_179_176_063
H +0.00000 +0.00000 +0.00000
O +0.00000 +0.00000 +0.95438
C −0.90202 +0.00000 +1.26615
C −1.68543 −1.21145 +0.81430
H −0.83625 +0.01772 +2.35158
H −1.41794 +0.91494 +0.96028
C −3.09650 −1.23653 +1.36852
H −1.72485 −1.22733 −0.27813
H −1.14517 −2.10792 +1.11911
C −3.89767 −2.44109 +0.91291
H −3.05603 −1.22469 +2.45980
H −3.62088 −0.32430 +1.07536
C −5.30413 −2.46562 +1.48148
H −3.95071 −2.44784 −0.17854
H −3.36391 −3.34877 +1.19715
C −6.16861 −3.60383 +0.96480
H −5.24967 −2.52174 +2.57121
H −5.79243 −1.51710 +1.25143
C −5.65855 −4.98287 +1.33707
H −7.17925 −3.48062 +1.35372
H −6.25067 −3.52657 −0.12079
H −6.34855 −5.75832 +1.01250
H −4.69431 −5.19496 +0.88075
H −5.54050 −5.07632 +2.41592
24
∗ E = +0.891 kcal/mol ; (28) 066_296_182_180_176_063
H +0.00000 +0.00000 +0.00000
O +0.00000 +0.00000 +0.95390
C −0.90055 +0.00000 +1.26845
C −1.64765 −1.27407 +0.93962
H −0.83310 +0.12617 +2.34552
H −1.44454 +0.86345 +0.87343
C −1.82692 −1.50766 −0.54820
H −1.11566 −2.11642 +1.38288
H −2.62733 −1.22919 +1.41907
C −2.62379 −2.75853 −0.86632
H −2.32296 −0.64161 −0.99449
H −0.84972 −1.58821 −1.03035
C −2.80072 −2.98709 −2.35576
H −2.12428 −3.62422 −0.42520
H −3.59793 −2.68878 −0.38052

```
C  −3.50382  −4.28694  −2.71038
H  −3.35614  −2.14946  −2.78434
H  −1.81872  −2.97478  −2.83184
C  −4.93301  −4.37088  −2.20947
H  −3.49831  −4.40393  −3.79393
H  −2.92854  −5.12465  −2.31254
H  −5.41677  −5.28269  −2.55192
H  −4.98225  −4.36605  −1.12295
H  −5.52247  −3.52884  −2.57031
24
 * E =  +0.893  kcal/mol ; (29) 066_296_183_185_297_185
H  +0.00000  +0.00000  +0.00000
O  +0.00000  +0.00000  +0.95392
C  −0.90058  +0.00000  +1.26842
C  −1.64942  −1.27106  +0.93305
H  −0.83288  +0.12095  +2.34607
H  −1.44312  +0.86625  +0.87750
C  −1.82418  −1.49938  −0.55653
H  −1.12188  −2.11660  +1.37554
H  −2.63104  −1.22516  +1.40864
C  −2.63977  −2.74088  −0.86946
H  −2.29831  −0.62081  −0.99878
H  −0.84531  −1.60023  −1.03271
C  −2.73480  −3.07425  −2.34845
H  −2.19965  −3.58843  −0.34211
H  −3.64555  −2.61933  −0.46099
C  −3.44458  −2.02598  −3.18574
H  −1.73055  −3.23341  −2.74837
H  −3.25781  −4.02517  −2.46261
C  −3.61322  −2.45191  −4.63039
H  −4.42306  −1.82101  −2.74706
H  −2.89299  −1.08652  −3.15063
H  −4.11889  −1.68882  −5.21741
H  −2.64795  −2.64240  −5.09679
H  −4.19772  −3.36759  −4.70276
24
 * E =  +0.899  kcal/mol ; (30) 181_062_182_185_303_303
H  +0.00000  +0.00000  +0.00000
O  +0.00000  +0.00000  +0.95329
C  −0.89726  +0.00000  +1.27528
C  −0.85666  +0.03604  +2.78143
H  −1.43991  +0.87257  +0.89973
H  −1.42656  −0.89449  +0.93879
C  −0.20731  +1.29084  +3.33079
H  −1.87751  −0.05228  +3.15651
```

H −0.31430 −0.84307 +3.13189
C −0.12557 +1.29260 +4.84513
H +0.78772 +1.38214 +2.89737
H −0.77119 +2.16583 +2.99623
C +0.40162 +2.59097 +5.43615
H −1.11703 +1.09921 +5.25889
H +0.49909 +0.45840 +5.16972
C +1.77529 +3.01195 +4.93573
H −0.31025 +3.39110 +5.22569
H +0.43658 +2.49291 +6.52268
C +2.84963 +1.96467 +5.15380
H +1.72062 +3.25925 +3.87527
H +2.06031 +3.93380 +5.44278
H +3.82947 +2.33932 +4.86702
H +2.90125 +1.66782 +6.20080
H +2.66006 +1.06815 +4.56642
24
* E = +0.904 kcal/mol ; (31) 066_296_188_297_185_180
H +0.00000 +0.00000 +0.00000
O +0.00000 +0.00000 +0.95402
C −0.90089 +0.00000 +1.26796
C −1.65084 −1.26845 +0.92379
H −0.83412 +0.11558 +2.34627
H −1.44166 +0.86882 +0.87996
C −1.81775 −1.47693 −0.57077
H −1.12428 −2.11191 +1.36830
H −2.63569 −1.22201 +1.39379
C −2.72249 −2.64081 −0.94039
H −2.22192 −0.56256 −1.01159
H −0.83955 −1.63874 −1.03002
C −2.21647 −3.99586 −0.48237
H −3.71725 −2.46805 −0.52353
H −2.84790 −2.66019 −2.02413
C −3.07937 −5.14547 −0.96648
H −1.19303 −4.13811 −0.83821
H −2.16492 −4.02666 +0.60698
C −2.57914 −6.49723 −0.49922
H −4.10352 −4.99464 −0.62127
H −3.12237 −5.12725 −2.05666
H −3.21155 −7.30515 −0.85976
H −1.56752 −6.68453 −0.85545
H −2.55884 −6.55277 +0.58789
24
* E = +0.905 kcal/mol ; (32) 061_177_179_175_064_175
H +0.00000 +0.00000 +0.00000

O	+0.00000	+0.00000	+0.95439
C	−0.90205	+0.00000	+1.26610
C	−1.68529	−1.21119	+0.81334
H	−0.83643	+0.01752	+2.35157
H	−1.41787	+0.91502	+0.96026
C	−3.09700	−1.23727	+1.36659
H	−1.72477	−1.22598	−0.27917
H	−1.14510	−2.10820	+1.11688
C	−3.89176	−2.44142	+0.89513
H	−3.05769	−1.23922	+2.45844
H	−3.61112	−0.31820	+1.08177
C	−5.27234	−2.56375	+1.51727
H	−3.98855	−2.40369	−0.19234
H	−3.32199	−3.34506	+1.11658
C	−6.21959	−1.42756	+1.17725
H	−5.72315	−3.50310	+1.19271
H	−5.17432	−2.63611	+2.60299
C	−7.61432	−1.64931	+1.72724
H	−5.82652	−0.48768	+1.56450
H	−6.26676	−1.31438	+0.09238
H	−8.27937	−0.82547	+1.47898
H	−8.05271	−2.56188	+1.32665
H	−7.59588	−1.74400	+2.81177

24

* E = +0.906 kcal/mol ; (33) 061_177_180_184_296_185

H	+0.00000	+0.00000	+0.00000
O	+0.00000	+0.00000	+0.95438
C	−0.90198	+0.00000	+1.26625
C	−1.68334	−1.21446	+0.81922
H	−0.83609	+0.02226	+2.35160
H	−1.41925	+0.91293	+0.95662
C	−3.09633	−1.23806	+1.36938
H	−1.71930	−1.23727	−0.27322
H	−1.14355	−2.10891	+1.13101
C	−3.88169	−2.45764	+0.92260
H	−3.05339	−1.20258	+2.45866
H	−3.62668	−0.33572	+1.05521
C	−5.33475	−2.46455	+1.36609
H	−3.84371	−2.51894	−0.16621
H	−3.38676	−3.35785	+1.29348
C	−5.53414	−2.51554	+2.86989
H	−5.83888	−1.58099	+0.96734
H	−5.83579	−3.32447	+0.91838
C	−6.99418	−2.63338	+3.25882
H	−4.97691	−3.36216	+3.27546

H −5.11079 −1.62355 +3.33135
H −7.12051 −2.66483 +4.33848
H −7.56797 −1.78761 +2.88328
H −7.43742 −3.53835 +2.84649
24
* E = +0.910 kcal/mol ; (34) 180_180_179_175_064_175
H +0.00000 +0.00000 +0.00000
O +0.00000 +0.00000 +0.95348
C −0.89786 +0.00000 +1.27437
C −0.86017 −0.00214 +2.77984
H −1.43620 +0.88352 +0.91967
H −1.43711 −0.88193 +0.91723
C −2.24326 +0.00045 +3.40049
H −0.30213 −0.87854 +3.10993
H −0.29680 +0.86968 +3.11278
C −2.20263 −0.01577 +4.91769
H −2.79302 +0.88436 +3.06754
H −2.80263 −0.86229 +3.03576
C −3.56341 +0.10001 +5.58305
H −1.71374 −0.93316 +5.25287
H −1.57029 +0.80511 +5.25907
C −4.50581 −1.05462 +5.29579
H −3.42287 +0.17525 +6.66268
H −4.03803 +1.03486 +5.27534
C −5.80574 −0.95008 +6.06799
H −4.72461 −1.09878 +4.22900
H −4.00636 −1.99326 +5.54347
H −6.47131 −1.78169 +5.84843
H −5.62403 −0.94515 +7.14150
H −6.33349 −0.03011 +5.82146
24
* E = +0.912 kcal/mol ; (35) 181_063_187_303_303_185
H +0.00000 +0.00000 +0.00000
O +0.00000 +0.00000 +0.95328
C −0.89715 +0.00000 +1.27556
C −0.85734 +0.03388 +2.78206
H −1.43972 +0.87329 +0.90138
H −1.42650 −0.89385 +0.93768
C −0.21665 +1.29626 +3.32650
H −1.87925 −0.06707 +3.15047
H −0.30719 −0.84124 +3.13189
C −0.01345 +1.27768 +4.83324
H +0.74682 +1.43194 +2.83866
H −0.82594 +2.15693 +3.04297
C −1.27278 +1.04257 +5.65297

```
H    +0.71575   +0.50438   +5.07992
H    +0.43493   +2.22480   +5.13777
C    -2.37817   +2.05106   +5.40210
H    -1.65698   +0.03783   +5.46492
H    -1.00960   +1.06387   +6.71192
C    -3.56538   +1.85704   +6.32389
H    -1.97858   +3.05973   +5.52364
H    -2.71192   +1.98203   +4.36576
H    -4.35168   +2.58177   +6.12537
H    -3.99388   +0.86302   +6.20488
H    -3.27214   +1.96328   +7.36704
24
* E =  +0.918 kcal/mol ; (36) 062_053_054_058_175_179
H    +0.00000   +0.00000   +0.00000
O    +0.00000   +0.00000   +0.95455
C    -0.90135   +0.00000   +1.26878
C    -1.67297   -1.23488   +0.85535
H    -0.82754   +0.05377   +2.35265
H    -1.42751   +0.89912   +0.93944
C    -0.97582   -2.53887   +1.21364
H    -2.65999   -1.18759   +1.31769
H    -1.84775   -1.20774   -0.22254
C    -0.58191   -2.66632   +2.67590
H    -1.62854   -3.36903   +0.93968
H    -0.07798   -2.63681   +0.60517
C    -1.73857   -2.54262   +3.64954
H    -0.10057   -3.63463   +2.82094
H    +0.17784   -1.91906   +2.91143
C    -1.33568   -2.80122   +5.08872
H    -2.18004   -1.54564   +3.58163
H    -2.52973   -3.24134   +3.36505
C    -2.48894   -2.66086   +6.06142
H    -0.91029   -3.80296   +5.16623
H    -0.53631   -2.11199   +5.36533
H    -2.17610   -2.85330   +7.08507
H    -2.90987   -1.65722   +6.02761
H    -3.28925   -3.35968   +5.82313
24
* E =  +0.920 kcal/mol ; (37) 063_061_178_065_175_180
H    +0.00000   +0.00000   +0.00000
O    +0.00000   +0.00000   +0.95457
C    -0.90240   +0.00000   +1.26583
C    -1.66344   -1.24631   +0.86951
H    -0.83380   +0.06709   +2.34959
H    -1.42971   +0.89131   +0.91728
```

C	−1.04826	−2.51732	+1.42344
H	−2.69316	−1.14876	+1.22048
H	−1.71838	−1.29286	−0.22040
C	−1.81435	−3.78333	+1.07868
H	−0.02094	−2.59983	+1.06823
H	−0.98409	−2.42430	+2.50883
C	−1.84733	−4.11551	−0.40182
H	−1.36465	−4.62157	+1.61321
H	−2.83821	−3.70263	+1.45171
C	−2.51617	−5.44417	−0.69858
H	−2.36792	−3.32978	−0.95194
H	−0.82558	−4.13227	−0.78938
C	−2.55675	−5.76879	−2.17807
H	−1.99080	−6.23680	−0.16360
H	−3.53128	−5.43124	−0.29837
H	−3.03786	−6.72587	−2.36584
H	−3.10617	−5.00847	−2.73090
H	−1.55239	−5.81587	−2.59584

24

* E = +0.920 kcal/mol ; (38) 059_182_296_186_181_180

H	+0.00000	+0.00000	+0.00000
O	+0.00000	+0.00000	+0.95450
C	−0.90210	+0.00000	+1.26640
C	−1.69069	−1.19326	+0.77324
H	−0.83093	−0.00622	+2.35033
H	−1.41251	+0.92552	+0.98099
C	−3.14358	−1.19245	+1.21785
H	−1.65292	−1.20534	−0.31850
H	−1.19396	−2.10340	+1.11066
C	−3.33831	−1.29429	+2.72018
H	−3.63633	−0.28902	+0.85128
H	−3.65610	−2.02820	+0.74016
C	−4.79486	−1.43004	+3.11898
H	−2.77792	−2.15275	+3.09782
H	−2.91763	−0.41680	+3.21377
C	−5.00451	−1.51977	+4.61812
H	−5.35774	−0.57960	+2.72724
H	−5.21844	−2.31693	+2.64214
C	−6.46342	−1.65957	+5.00291
H	−4.43968	−2.36760	+5.00883
H	−4.58404	−0.63190	+5.09286
H	−6.58929	−1.72150	+6.08129
H	−7.04442	−0.80899	+4.65021
H	−6.89895	−2.55708	+4.56682

24

```
 * E =  +0.923  kcal/mol ; (39) 067_296_178_064_175_179
 H   +0.00000   +0.00000   +0.00000
 O   +0.00000   +0.00000   +0.95376
 C   −0.90008   +0.00000   +1.26922
 C   −1.64548   −1.28007   +0.95772
 H   −0.83112   +0.13833   +2.34465
 H   −1.44785   +0.85726   +0.86605
 C   −1.83074   −1.52294   −0.52881
 H   −1.10434   −2.11703   +1.40136
 H   −2.61637   −1.22983   +1.45127
 C   −2.53370   −2.82783   −0.86570
 H   −2.38937   −0.69003   −0.96386
 H   −0.85393   −1.53010   −1.01613
 C   −3.96559   −2.91577   −0.37158
 H   −2.52839   −2.96095   −1.94864
 H   −1.95883   −3.66092   −0.45576
 C   −4.67360   −4.17666   −0.82964
 H   −3.98943   −2.87473   +0.71842
 H   −4.52257   −2.04275   −0.72123
 C   −6.09958   −4.27014   −0.32599
 H   −4.66647   −4.21573   −1.92004
 H   −4.10828   −5.04691   −0.49247
 H   −6.58681   −5.17991   −0.66884
 H   −6.13138   −4.26786   +0.76228
 H   −6.69331   −3.42597   −0.67288
24
 * E =  +0.923  kcal/mol ; (40) 182_062_180_175_057_057
 H   +0.00000   +0.00000   +0.00000
 O   +0.00000   +0.00000   +0.95329
 C   −0.89721   +0.00000   +1.27543
 C   −0.85597   +0.03750   +2.78136
 H   −1.44006   +0.87220   +0.89929
 H   −1.42641   −0.89487   +0.93973
 C   −0.20125   +1.29038   +3.32901
 H   −1.87765   −0.04423   +3.15607
 H   −0.31888   −0.84402   +3.13352
 C   −0.15609   +1.31158   +4.84479
 H   +0.80859   +1.36604   +2.92567
 H   −0.74597   +2.16296   +2.96115
 C   +0.60628   +2.49167   +5.42718
 H   −1.17497   +1.29885   +5.23738
 H   +0.30882   +0.38984   +5.19817
 C   +0.09583   +3.86006   +5.00112
 H   +0.57438   +2.42680   +6.51629
 H   +1.65735   +2.40582   +5.14721
```

```
 C    −1.36846   +4.09080   +5.31970
 H    +0.69705   +4.62307   +5.49543
 H    +0.26111   +3.99752   +3.93216
 H    −1.66956   +5.10800   +5.07992
 H    −2.01236   +3.41881   +4.75484
 H    −1.57047   +3.92563   +6.37739
24
 * E =   +0.943  kcal/mol ; (41) 067_296_182_176_064_175
 H    +0.00000   +0.00000   +0.00000
 O    +0.00000   +0.00000   +0.95387
 C    −0.90075   +0.00000   +1.26773
 C    −1.64627   −1.27585   +0.94247
 H    −0.83429   +0.12960   +2.34447
 H    −1.44533   +0.86169   +0.86969
 C    −1.82204   −1.51646   −0.54492
 H    −1.11447   −2.11604   +1.39009
 H    −2.62688   −1.23008   +1.42009
 C    −2.62078   −2.77103   −0.84864
 H    −2.32176   −0.65465   −0.99640
 H    −0.84173   −1.59273   −1.01816
 C    −2.89538   −2.99933   −2.32536
 H    −2.09404   −3.63663   −0.44113
 H    −3.57109   −2.71682   −0.31527
 C    −1.65472   −3.23132   −3.16845
 H    −3.55503   −3.86216   −2.43144
 H    −3.44816   −2.14674   −2.72702
 C    −1.98501   −3.56520   −4.60942
 H    −1.01682   −2.34789   −3.14260
 H    −1.07030   −4.04158   −2.72852
 H    −1.08621   −3.72424   −5.20054
 H    −2.58817   −4.46936   −4.67325
 H    −2.55065   −2.76094   −5.07719
24
 * E =   +0.947  kcal/mol ; (42) 177_174_063_174_179_180
 H    +0.00000   +0.00000   +0.00000
 O    +0.00000   +0.00000   +0.95349
 C    −0.89799   +0.00000   +1.27406
 C    −0.85076   −0.06657   +2.77934
 H    −1.42086   +0.91021   +0.96384
 H    −1.44802   −0.85298   +0.87071
 C    −2.21110   +0.06988   +3.43999
 H    −0.38361   −1.00745   +3.07137
 H    −0.19079   +0.72861   +3.12365
 C    −3.19372   −1.03545   +3.09591
 H    −2.07061   +0.08968   +4.52129
```

H −2.64929 +1.03543 +3.17729
C −4.48405 −0.94808 +3.88740
H −3.43105 −1.00884 +2.03087
H −2.72265 −2.00453 +3.27740
C −5.47998 −2.03841 +3.54270
H −4.25588 −0.99280 +4.95474
H −4.94593 +0.02760 +3.71911
C −6.76260 −1.94195 +4.34383
H −5.70957 −1.99067 +2.47701
H −5.01677 −3.01230 +3.70868
H −7.46090 −2.73274 +4.07983
H −6.56374 −2.01894 +5.41150
H −7.26081 −0.98929 +4.17162
24
* E = +0.962 kcal/mol ; (43) 182_063_177_058_056_175
H +0.00000 +0.00000 +0.00000
O +0.00000 +0.00000 +0.95330
C −0.89722 +0.00000 +1.27545
C −0.85316 +0.05796 +2.78089
H −1.44428 +0.86465 +0.88813
H −1.42177 −0.90201 +0.95176
C −0.21022 +1.32887 +3.30147
H −1.87253 −0.02989 +3.16149
H −0.30657 −0.81617 +3.13584
C −0.21800 +1.44645 +4.81769
H +0.80852 +1.38568 +2.92167
H −0.73613 +2.18816 +2.88085
C +0.46422 +0.30431 +5.55562
H +0.26607 +2.38392 +5.09635
H −1.24988 +1.52311 +5.16565
C +1.90570 +0.07098 +5.14392
H +0.43001 +0.51185 +6.62661
H −0.10043 −0.61978 +5.41644
C +2.58706 −0.98875 +5.98619
H +1.94651 −0.22043 +4.09392
H +2.45661 +1.01064 +5.21865
H +3.61631 −1.14922 +5.67365
H +2.59957 −0.70630 +7.03779
H +2.06652 −1.94224 +5.90992
24
* E = +0.994 kcal/mol ; (44) 177_051_056_176_056_054
H +0.00000 +0.00000 +0.00000
O +0.00000 +0.00000 +0.95340
C −0.89763 +0.00000 +1.27471
C −0.85897 −0.07336 +2.78254

H −1.41471 +0.90822 +0.95563
H −1.45208 −0.85325 +0.87819
C +0.04935 +0.97132 +3.40926
H −1.87983 +0.03894 +3.15123
H −0.52194 −1.06643 +3.07766
C −0.29003 +2.39634 +3.01771
H +0.00803 +0.87162 +4.49536
H +1.07383 +0.75029 +3.11384
C +0.56257 +3.44097 +3.72106
H −0.17010 +2.50716 +1.93928
H −1.34280 +2.59424 +3.23040
C +2.06170 +3.26195 +3.52710
H +0.27440 +4.42958 +3.35922
H +0.33915 +3.42974 +4.78934
C +2.47715 +3.16992 +2.07124
H +2.57589 +4.09711 +4.00283
H +2.39539 +2.36744 +4.05369
H +3.55976 +3.13113 +1.97350
H +2.07126 +2.27724 +1.59718
H +2.12508 +4.03432 +1.50854
24
∗ E = +1.004 kcal/mol ; (45) 180_056_056_055_058_175
H +0.00000 +0.00000 +0.00000
O +0.00000 +0.00000 +0.95333
C −0.89727 +0.00000 +1.27543
C −0.85530 −0.00782 +2.78341
H −1.43116 +0.88569 +0.91978
H −1.43830 −0.87888 +0.91748
C −0.04863 +1.13849 +3.37231
H −1.88265 +0.01576 +3.15084
H −0.42714 −0.95342 +3.11415
C −0.50400 +2.52328 +2.93607
H −0.08037 +1.06350 +4.45937
H +0.99296 +1.01017 +3.08464
C −1.97270 +2.82783 +3.18666
H +0.10421 +3.26587 +3.45494
H −0.28635 +2.65124 +1.87493
C −2.39583 +2.70200 +4.63829
H −2.18525 +3.84181 +2.84334
H −2.59960 +2.17253 +2.57674
C −3.83352 +3.12336 +4.86653
H −2.26443 +1.67198 +4.97227
H −1.73203 +3.30737 +5.25848
H −4.12197 +3.02028 +5.91008
H −3.98676 +4.16289 +4.58124

H −4.51704 +2.51767 +4.27332
24
∗ E = +1.026 kcal/mol ; (46) 062_173_065_174_180_180
H +0.00000 +0.00000 +0.00000
O +0.00000 +0.00000 +0.95443
C −0.90226 +0.00000 +1.26566
C −1.66692 −1.23052 +0.82925
H −0.83770 +0.02938 +2.35150
H −1.41472 +0.91054 +0.94769
C −3.05363 −1.34398 +1.44021
H −1.75078 −1.23416 −0.26094
H −1.07220 −2.10254 +1.09868
C −4.01925 −0.24742 +1.02658
H −3.47951 −2.30858 +1.16165
H −2.96808 −1.35773 +2.52897
C −5.42520 −0.47052 +1.54876
H −3.66202 +0.72169 +1.37818
H −4.04527 −0.18412 −0.06413
C −6.39857 +0.62132 +1.14889
H −5.79537 −1.43309 +1.18849
H −5.39789 −0.54709 +2.63813
C −7.80023 +0.38405 +1.67341
H −6.02938 +1.58165 +1.51221
H −6.42299 +0.69884 +0.06074
H −8.47987 +1.17876 +1.37482
H −8.20440 −0.55510 +1.29921
H −7.80764 +0.33347 +2.76095
24
∗ E = +1.035 kcal/mol ; (47) 065_302_306_301_185_181
H +0.00000 +0.00000 +0.00000
O +0.00000 +0.00000 +0.95464
C −0.90244 +0.00000 +1.26599
C −1.65608 −1.26089 +0.89925
H −0.84122 +0.09572 +2.34650
H −1.43446 +0.88165 +0.89667
C −1.70317 −1.53629 −0.59734
H −1.18756 −2.10738 +1.39972
H −2.66793 −1.18134 +1.29863
C −2.24404 −0.39601 −1.44716
H −0.70207 −1.79860 −0.94382
H −2.31104 −2.42511 −0.76904
C −3.65375 +0.03384 −1.08843
H −1.58314 +0.47310 −1.38189
H −2.22160 −0.70160 −2.49414
C −4.21542 +1.08104 −2.03078

```
H  −4.30780  −0.84168  −1.08871
H  −3.67588  +0.42600  −0.06969
C  −5.61902  +1.51621  −1.66166
H  −3.55312  +1.94832  −2.03796
H  −4.20902  +0.68755  −3.04841
H  −6.00054  +2.26512  −2.35163
H  −6.30683  +0.67237  −1.67576
H  −5.64593  +1.94422  −0.66094
24
* E =  +1.075  kcal/mol ; (48) 064_061_188_303_302_185
H  +0.00000  +0.00000  +0.00000
O  +0.00000  +0.00000  +0.95452
C  −0.90250  +0.00000  +1.26534
C  −1.65690  −1.25679  +0.88951
H  −0.83538  +0.08478  +2.34797
H  −1.43400  +0.88292  +0.90261
C  −1.03984  −2.51347  +1.47234
H  −2.69086  −1.14728  +1.22192
H  −1.69809  −1.33713  −0.20113
C  −1.65419  −3.80088  +0.94635
H  +0.02714  −2.51037  +1.25454
H  −1.12318  −2.47703  +2.55999
C  −3.15732  −3.92518  +1.14193
H  −1.43021  −3.88963  −0.11823
H  −1.16312  −4.64677  +1.42996
C  −3.60935  −3.81943  +2.58636
H  −3.67339  −3.16607  +0.55021
H  −3.48402  −4.88542  +0.73928
C  −5.09449  −4.07126  +2.75255
H  −3.04516  −4.53091  +3.19232
H  −3.36593  −2.83066  +2.97771
H  −5.40332  −3.98316  +3.79158
H  −5.67892  −3.35889  +2.17223
H  −5.36280  −5.06941  +2.41033
24
* E =  +1.079  kcal/mol ; (49) 180_056_059_169_062_176
H  +0.00000  +0.00000  +0.00000
O  +0.00000  +0.00000  +0.95330
C  −0.89741  +0.00000  +1.27491
C  −0.85687  −0.00840  +2.78323
H  −1.43222  +0.88375  +0.91778
H  −1.43715  −0.88011  +0.91789
C  −0.05660  +1.13876  +3.37688
H  −1.88492  +0.02334  +3.14823
H  −0.43152  −0.95588  +3.11242
```

```
C  −0.57229  +2.51215  +2.98571
H  −0.07117  +1.04750  +4.46466
H  +0.98083  +1.03140  +3.06653
C  +0.05581  +3.65115  +3.77124
H  −0.39413  +2.67637  +1.92126
H  −1.65542  +2.54142  +3.12528
C  +1.55516  +3.78851  +3.58044
H  −0.42182  +4.58922  +3.48230
H  −0.15868  +3.51770  +4.83445
C  +2.12461  +4.99627  +4.29725
H  +2.05631  +2.88838  +3.93540
H  +1.77448  +3.85618  +2.51335
H  +3.19970  +5.07791  +4.15441
H  +1.67109  +5.91744  +3.93450
H  +1.93698  +4.93946  +5.36843
24
* E =  +1.094  kcal/mol ; (50) 179_055_059_174_175_063
H  +0.00000  +0.00000  +0.00000
O  +0.00000  +0.00000  +0.95330
C  −0.89742  +0.00000  +1.27489
C  −0.85673  −0.01761  +2.78309
H  −1.43027  +0.88696  +0.92281
H  −1.43923  −0.87671  +0.91271
C  −0.04513  +1.11671  +3.38542
H  −1.88425  +0.02044  +3.14875
H  −0.43999  −0.97088  +3.10662
C  −0.53338  +2.50246  +3.00787
H  −0.06074  +1.01583  +4.47177
H  +0.99372  +1.00702  +3.07669
C  +0.20810  +3.60072  +3.74774
H  −0.40626  +2.65479  +1.93411
H  −1.60452  +2.57551  +3.20778
C  −0.15528  +5.00950  +3.30899
H  +0.02394  +3.49890  +4.81999
H  +1.28008  +3.45133  +3.60932
C  −1.60432  +5.37869  +3.56312
H  +0.48956  +5.71747  +3.82970
H  +0.06796  +5.12173  +2.24653
H  −1.79843  +6.41679  +3.30289
H  −2.28604  +4.76425  +2.97931
H  −1.86086  +5.24789  +4.61377
24
* E =  +1.118  kcal/mol ; (51) 065_061_184_186_303_303
H  +0.00000  +0.00000  +0.00000
O  +0.00000  +0.00000  +0.95449
```

C −0.90211 +0.00000 +1.26633
C −1.64970 −1.26540 +0.90780
H −0.83410 +0.09960 +2.34759
H −1.43825 +0.87540 +0.89241
C −1.02866 −2.51547 +1.49856
H −2.68334 −1.16587 +1.24479
H −1.69565 −1.35740 −0.18118
C −1.73148 −3.78645 +1.06180
H +0.02366 −2.54805 +1.21740
H −1.04915 −2.44416 +2.58890
C −1.21824 −5.04447 +1.74521
H −2.80030 −3.69271 +1.26217
H −1.64141 −3.89133 −0.02116
C +0.27235 −5.30119 +1.58104
H −1.45050 −4.98837 +2.81004
H −1.76960 −5.90334 +1.35844
C +0.72109 −5.38227 +0.13510
H +0.83870 −4.52304 +2.09322
H +0.52042 −6.23324 +2.08855
H +1.77126 −5.65516 +0.06277
H +0.14571 −6.12728 −0.41336
H +0.59729 −4.42948 −0.37667
24
* E = +1.133 kcal/mol ; (52) 066_297_188_303_303_185
H +0.00000 +0.00000 +0.00000
O +0.00000 +0.00000 +0.95403
C −0.90089 +0.00000 +1.26800
C −1.64866 −1.27092 +0.92828
H −0.83439 +0.12004 +2.34585
H −1.44323 +0.86643 +0.87683
C −1.80814 −1.49028 −0.56478
H −1.12217 −2.11255 +1.37872
H −2.63448 −1.22539 +1.39632
C −2.71251 −2.65976 −0.92484
H −2.21553 −0.58330 −1.01747
H −0.82728 −1.64556 −1.01848
C −2.29174 −3.99911 −0.33995
H −3.72780 −2.43392 −0.59499
H −2.75774 −2.74644 −2.01155
C −0.88340 −4.42565 −0.70936
H −2.38450 −3.97551 +0.74746
H −2.99377 −4.76212 −0.68010
C −0.54993 −5.82007 −0.21865
H −0.76684 −4.37986 −1.79407
H −0.16334 −3.71796 −0.29640

```
 H    +0.46462    -6.10710    -0.48473
 H    -0.63776    -5.88393    +0.86477
 H    -1.22676    -6.55812    -0.64601
24
 * E =   +1.139  kcal/mol  ;  (53)  179_055_059_175_183_296
 H    +0.00000    +0.00000    +0.00000
 O    +0.00000    +0.00000    +0.95331
 C    -0.89736    +0.00000    +1.27511
 C    -0.85603    -0.01607    +2.78346
 H    -1.43078    +0.88604    +0.92173
 H    -1.43899    -0.87739    +0.91427
 C    -0.03832    +1.11433    +3.38508
 H    -1.88310    +0.02655    +3.14982
 H    -0.44312    -0.97087    +3.10739
 C    -0.52372    +2.50267    +3.01390
 H    -0.04799    +1.00952    +4.47107
 H    +0.99824    +1.00203    +3.06951
 C    +0.23700    +3.59806    +3.73809
 H    -0.42413    +2.64061    +1.93726
 H    -1.58958    +2.59396    +3.24248
 C    -0.26221    +5.00493    +3.45273
 H    +0.18069    +3.41331    +4.81225
 H    +1.29502    +3.53128    +3.47442
 C    -0.10537    +5.43384    +2.00629
 H    -1.31204    +5.07831    +3.74248
 H    +0.27690    +5.70469    +4.09138
 H    -0.40350    +6.47077    +1.86842
 H    +0.93083    +5.34161    +1.68336
 H    -0.71311    +4.82938    +1.33678
24
 * E =   +1.139  kcal/mol  ;  (54)  183_063_185_302_306_302
 H    +0.00000    +0.00000    +0.00000
 O    +0.00000    +0.00000    +0.95329
 C    -0.89754    +0.00000    +1.27450
 C    -0.86154    +0.08133    +2.77919
 H    -1.44894    +0.85550    +0.87320
 H    -1.41630    -0.90944    +0.96280
 C    -0.23540    +1.36706    +3.28449
 H    -1.88305    -0.01832    +3.14860
 H    -0.30297    -0.77608    +3.15814
 C    -0.06933    +1.41526    +4.79459
 H    +0.73774    +1.48598    +2.81183
 H    -0.84180    +2.21303    +2.95107
 C    -1.35791    +1.23845    +5.58628
 H    +0.63523    +0.63974    +5.09890
```

```
H    +0.39645   +2.36293   +5.06643
C    -2.47334   +2.20039   +5.20806
H    -1.71908   +0.21566   +5.47164
H    -1.13295   +1.35713   +6.64762
C    -2.09181   +3.66095   +5.35043
H    -2.79164   +2.00810   +4.18185
H    -3.34154   +1.99102   +5.83289
H    -2.93748   +4.31277   +5.14356
H    -1.74542   +3.87759   +6.36022
H    -1.29255   +3.93500   +4.66432
24
 * E =   +1.140  kcal/mol ;  (55) 064_060_182_175_057_057
H    +0.00000   +0.00000   +0.00000
O    +0.00000   +0.00000   +0.95452
C    -0.90228   +0.00000   +1.26596
C    -1.65648   -1.25596   +0.88891
H    -0.83402   +0.08361   +2.34855
H    -1.43431   +0.88332   +0.90471
C    -1.03869   -2.51945   +1.45402
H    -2.68762   -1.15773   +1.23419
H    -1.70992   -1.32799   -0.20117
C    -1.77400   -3.77629   +1.02999
H    +0.00521   -2.57673   +1.14353
H    -1.02620   -2.44549   +2.54339
C    -1.10851   -5.06715   +1.48156
H    -2.79921   -3.73824   +1.40357
H    -1.85632   -3.79030   -0.05824
C    -0.89548   -5.18857   +2.98303
H    -1.71246   -5.91022   +1.14137
H    -0.14364   -5.16158   +0.98078
C    -2.17029   -5.05102   +3.79211
H    -0.43925   -6.15639   +3.19033
H    -0.17253   -4.44236   +3.31360
H    -1.98834   -5.22979   +4.84927
H    -2.59848   -4.05432   +3.69965
H    -2.92449   -5.76356   +3.46007
24
 * E =   +1.141  kcal/mol ;  (56) 179_178_173_056_057_175
H    +0.00000   +0.00000   +0.00000
O    +0.00000   +0.00000   +0.95348
C    -0.89778   +0.00000   +1.27461
C    -0.86131   -0.02467   +2.77995
H    -1.43157   +0.89172   +0.93329
H    -1.44227   -0.87326   +0.90449
C    -2.24813   +0.01573   +3.39216
```

H −0.33312 −0.92357 +3.10040
H −0.26674 +0.82371 +3.11844
C −2.25601 −0.17274 +4.90163
H −2.73039 +0.96063 +3.13569
H −2.86436 −0.76532 +2.94338
C −1.40260 +0.81872 +5.67680
H −3.28566 −0.10858 +5.25707
H −1.91685 −1.18380 +5.13206
C −1.76470 +2.27258 +5.43835
H −1.49704 +0.60078 +6.74190
H −0.34855 +0.66871 +5.43587
C −0.97695 +3.22106 +6.31949
H −1.59249 +2.52769 +4.39177
H −2.83378 +2.41045 +5.61215
H −1.24154 +4.25878 +6.13009
H −1.15982 +3.01895 +7.37360
H +0.09291 +3.11577 +6.14689
24
* E = +1.150 kcal/mol ; (57) 061_176_173_057_057_175
H +0.00000 +0.00000 +0.00000
O +0.00000 +0.00000 +0.95438
C −0.90200 +0.00000 +1.26621
C −1.68400 −1.21496 +0.82130
H −0.83648 +0.02335 +2.35168
H −1.41961 +0.91241 +0.95579
C −3.08733 −1.24172 +1.39672
H −1.73857 −1.22967 −0.27109
H −1.12945 −2.10541 +1.11729
C −3.95666 −2.36343 +0.84918
H −3.02739 −1.31712 +2.48360
H −3.57910 −0.28951 +1.19073
C −3.38773 −3.76231 +1.02744
H −4.93444 −2.31443 +1.33084
H −4.13239 −2.18738 −0.21330
C −3.08699 −4.13370 +2.46738
H −4.09853 −4.48166 +0.61748
H −2.47878 −3.87244 +0.43278
C −2.64182 −5.57456 +2.61666
H −2.31245 −3.47561 +2.86357
H −3.97534 −3.95769 +3.07717
H −2.42246 −5.82152 +3.65274
H −3.41212 −6.26039 +2.26785
H −1.74290 −5.76771 +2.03344
24
* E = +1.163 kcal/mol ; (58) 066_296_183_186_303_303

```
H    +0.00000    +0.00000    +0.00000
O    +0.00000    +0.00000    +0.95394
C    −0.90079    +0.00000    +1.26791
C    −1.65044    −1.26965    +0.92894
H    −0.83384    +0.11845    +2.34590
H    −1.44238    +0.86756    +0.87851
C    −1.82250    −1.49568    −0.56121
H    −1.12498    −2.11664    +1.37105
H    −2.63303    −1.22345    +1.40258
C    −2.64586    −2.72995    −0.87904
H    −2.29221    −0.61406    −1.00466
H    −0.84344    −1.59913    −1.03655
C    −2.71786    −3.06581    −2.36029
H    −2.22255    −3.58305    −0.34712
H    −3.65246    −2.59927    −0.47802
C    −3.26436    −1.95555    −3.24524
H    −1.72074    −3.33759    −2.71071
H    −3.33719    −3.95524    −2.48717
C    −4.64675    −1.48011    −2.84321
H    −2.57509    −1.11033    −3.24469
H    −3.29125    −2.31486    −4.27372
H    −5.03439    −0.75036    −3.55012
H    −5.35069    −2.31044    −2.80424
H    −4.64053    −1.01028    −1.86110
24
 * E =   +1.175  kcal/mol ;  (59)  182_063_178_058_053_058
H    +0.00000    +0.00000    +0.00000
O    +0.00000    +0.00000    +0.95330
C    −0.89709    +0.00000    +1.27582
C    −0.85147    +0.03903    +2.78182
H    −1.44059    +0.87162    +0.89918
H    −1.42572    −0.89577    +0.94169
C    −0.20632    +1.30252    +3.31794
H    −1.87033    −0.05187    +3.16283
H    −0.30538    −0.83989    +3.12515
C    −0.18205    +1.38503    +4.83568
H    +0.80551    +1.37257    +2.91988
H    −0.74468    +2.16767    +2.92622
C    +0.54454    +0.23707    +5.52433
H    +0.27778    +2.32992    +5.12665
H    −1.20620    +1.42414    +5.21127
C    +1.95951    −0.00796    +5.02383
H    +0.57614    +0.44062    +6.59617
H    −0.03383    −0.68077    +5.41291
C    +2.86774    +1.19898    +5.15678
```

```
H    +2.38528   -0.84301   +5.58019
H    +1.93030   -0.32613   +3.98064
H    +3.88648   +0.96066   +4.86008
H    +2.53154   +2.02381   +4.53121
H    +2.89641   +1.55674   +6.18549
24
 * E =  +1.175  kcal/mol ; (60) 180_180_179_175_057_057
H    +0.00000   +0.00000   +0.00000
O    +0.00000   +0.00000   +0.95350
C    -0.89793   +0.00000   +1.27424
C    -0.86136   -0.00532   +2.77978
H    -1.43577   +0.88450   +0.92117
H    -1.43786   -0.88080   +0.91523
C    -2.24490   -0.00224   +3.39927
H    -0.30479   -0.88320   +3.10864
H    -0.29701   +0.86487   +3.11521
C    -2.20790   -0.03257   +4.91563
H    -2.79330   +0.88415   +3.07058
H    -2.80744   -0.86169   +3.02852
C    -3.57229   +0.10077   +5.57348
H    -1.72675   -0.95590   +5.24266
H    -1.56843   +0.77673   +5.27079
C    -4.59126   -0.94992   +5.15860
H    -3.44360   +0.05878   +6.65638
H    -3.97700   +1.09018   +5.35368
C    -4.12948   -2.37391   +5.39843
H    -5.51501   -0.77215   +5.70897
H    -4.84413   -0.82248   +4.10546
H    -4.91711   -3.08827   +5.17041
H    -3.27069   -2.62726   +4.77914
H    -3.83832   -2.52176   +6.43765
24
 * E =  +1.177  kcal/mol ; (61) 061_177_180_185_303_303
H    +0.00000   +0.00000   +0.00000
O    +0.00000   +0.00000   +0.95437
C    -0.90179   +0.00000   +1.26678
C    -1.68396   -1.21440   +0.82087
H    -0.83520   +0.02250   +2.35208
H    -1.41943   +0.91285   +0.95748
C    -3.09615   -1.23713   +1.37281
H    -1.72114   -1.23766   -0.27145
H    -1.14432   -2.10910   +1.13236
C    -3.88109   -2.45983   +0.93625
H    -3.05200   -1.19788   +2.46295
H    -3.62801   -0.33576   +1.05828
```

```
C    −5.33791    −2.45096    +1.37246
H    −3.84317    −2.53650    −0.15146
H    −3.38602    −3.35580    +1.31443
C    −5.55884    −2.32437    +2.87239
H    −5.85381    −1.63111    +0.86982
H    −5.81263    −3.36908    +1.02235
C    −4.86748    −3.40303    +3.68299
H    −5.22526    −1.34360    +3.21297
H    −6.63054    −2.35703    +3.06750
H    −5.12170    −3.32807    +4.73763
H    −5.15713    −4.39614    +3.34178
H    −3.78395    −3.33299    +3.60392
24
 * E =   +1.178  kcal/mol  ;  (62)  060_177_179_174_057_057
H    +0.00000    +0.00000    +0.00000
O    +0.00000    +0.00000    +0.95439
C    −0.90201    +0.00000    +1.26623
C    −1.68689    −1.20921    +0.81109
H    −0.83612    +0.01468    +2.35174
H    −1.41707    +0.91632    +0.96295
C    −3.09758    −1.23558    +1.36665
H    −1.72833    −1.22089    −0.28143
H    −1.14717    −2.10763    +1.11110
C    −3.89835    −2.43179    +0.88747
H    −3.05752    −1.24222    +2.45850
H    −3.61102    −0.31307    +1.08788
C    −5.27069    −2.55828    +1.53048
H    −4.00086    −2.38190    −0.19804
H    −3.33233    −3.34189    +1.09071
C    −6.17688    −1.34870    +1.35565
H    −5.77029    −3.43594    +1.11695
H    −5.14582    −2.75389    +2.59675
C    −6.40838    −0.96494    −0.09273
H    −7.13448    −1.56298    +1.82968
H    −5.76292    −0.49655    +1.89570
H    −7.12791    −0.15385    −0.17615
H    −5.48872    −0.63451    −0.57271
H    −6.79163    −1.80856    −0.66546
24
 * E =   +1.182  kcal/mol  ;  (63)  063_054_056_055_058_175
H    +0.00000    +0.00000    +0.00000
O    +0.00000    +0.00000    +0.95458
C    −0.90192    +0.00000    +1.26724
C    −1.66822    −1.24048    +0.85966
H    −0.83070    +0.06211    +2.35061
```

```
H    -1.42977   +0.89491   +0.92923
C    -0.98055   -2.53924   +1.24933
H    -2.66363   -1.18582   +1.30508
H    -1.82498   -1.22782   -0.22130
C    -0.65832   -2.66073   +2.73160
H    -1.60652   -3.37427   +0.93447
H    -0.05036   -2.62752   +0.69004
C    -1.84386   -2.47070   +3.66456
H    -0.22285   -3.64512   +2.90957
H    +0.11986   -1.94061   +2.98574
C    -2.99045   -3.43380   +3.41972
H    -1.50105   -2.58336   +4.69438
H    -2.21847   -1.44677   +3.58985
C    -4.10474   -3.28619   +4.43618
H    -3.39396   -3.27975   +2.41794
H    -2.60903   -4.45650   +3.43749
H    -4.91955   -3.98021   +4.24328
H    -3.74124   -3.47390   +5.44515
H    -4.51750   -2.27862   +4.41996
24
 * E =  +1.186 kcal/mol ; (64) 067_296_181_175_057_056
H    +0.00000   +0.00000   +0.00000
O    +0.00000   +0.00000   +0.95387
C    -0.90041   +0.00000   +1.26872
C    -1.64663   -1.27548   +0.94356
H    -0.83239   +0.12852   +2.34549
H    -1.44536   +0.86222   +0.87235
C    -1.83316   -1.51083   -0.54323
H    -1.11016   -2.11648   +1.38421
H    -2.62382   -1.23350   +1.42832
C    -2.61276   -2.77654   -0.84701
H    -2.34741   -0.65345   -0.98674
H    -0.85583   -1.57129   -1.02731
C    -2.92227   -2.97930   -2.32198
H    -2.06079   -3.63545   -0.46179
H    -3.55232   -2.75450   -0.29288
C    -1.70658   -3.00971   -3.23627
H    -3.47302   -3.91428   -2.43709
H    -3.59494   -2.18740   -2.65570
C    -0.68339   -4.06171   -2.85540
H    -2.04609   -3.18859   -4.25625
H    -1.23108   -2.02826   -3.25097
H    +0.12365   -4.10893   -3.58268
H    -0.23510   -3.85491   -1.88509
H    -1.13905   -5.04962   -2.80136
```

```
24
 * E =   +1.186  kcal/mol  ;  (65)  062_179_187_303_303_185
 H    +0.00000    +0.00000    +0.00000
 O    +0.00000    +0.00000    +0.95432
 C    -0.90168    +0.00000    +1.26690
 C    -1.67549    -1.22894    +0.84680
 H    -0.83524    +0.04542    +2.35148
 H    -1.42623    +0.90254    +0.93923
 C    -3.09548    -1.22792    +1.38064
 H    -1.68959    -1.28278    -0.24473
 H    -1.14122    -2.11404    +1.19457
 C    -3.85595    -2.51773    +1.11211
 H    -3.07106    -1.05859    +2.45828
 H    -3.64073    -0.38199    +0.95853
 C    -3.94478    -2.91909    -0.35179
 H    -3.38475    -3.32758    +1.67088
 H    -4.86545    -2.41805    +1.51394
 C    -4.57560    -1.87046    -1.24854
 H    -2.95058    -3.16365    -0.73107
 H    -4.52357    -3.84119    -0.42503
 C    -4.75808    -2.35474    -2.67289
 H    -5.54135    -1.57605    -0.83326
 H    -3.96084    -0.96903    -1.25065
 H    -5.20345    -1.58906    -3.30380
 H    -3.80354    -2.63473    -3.11581
 H    -5.40383    -3.23053    -2.70840
24
 * E =   +1.203  kcal/mol  ;  (66)  064_051_056_174_057_055
 H    +0.00000    +0.00000    +0.00000
 O    +0.00000    +0.00000    +0.95461
 C    -0.90133    +0.00000    +1.26907
 C    -1.66165    -1.25195    +0.88162
 H    -0.82767    +0.07984    +2.35028
 H    -1.43440    +0.88677    +0.91868
 C    -0.92752    -2.53668    +1.22675
 H    -2.63240    -1.22570    +1.38049
 H    -1.87008    -1.23095    -0.19036
 C    -0.56135    -2.65640    +2.69360
 H    -1.54332    -3.38859    +0.93301
 H    -0.01901    -2.59122    +0.62722
 C    +0.04923    -4.00001    +3.06030
 H    +0.13831    -1.86162    +2.95352
 H    -1.45137    -2.49246    +3.30484
 C    +1.29072    -4.36714    +2.25994
 H    +0.30295    -3.99071    +4.12167
```

```
H    −0.70020    −4.78314    +2.93211
C    +2.37912    −3.31208    +2.31051
H    +1.68039    −5.31229    +2.63808
H    +1.01551    −4.55373    +1.22124
H    +3.27629    −3.64804    +1.79543
H    +2.05980    −2.38185    +1.84293
H    +2.65499    −3.08382    +3.33952
24
* E =  +1.209  kcal/mol ; (67) 182_063_186_298_189_297
H    +0.00000    +0.00000    +0.00000
O    +0.00000    +0.00000    +0.95328
C    −0.89723    +0.00000    +1.27533
C    −0.85871    +0.04290    +2.78167
H    −1.44135    +0.86992    +0.89570
H    −1.42469    −0.89685    +0.94249
C    −0.21367    +1.30705    +3.31847
H    −1.88038    −0.05392    +3.14910
H    −0.30889    −0.83021    +3.13698
C    −0.03696    +1.31298    +4.82721
H    +0.75575    +1.42655    +2.83820
H    −0.81637    +2.16930    +3.02176
C    −1.34049    +1.24403    +5.60379
H    +0.60812    +0.47958    +5.10985
H    +0.49601    +2.22085    +5.11699
C    −1.18182    +1.47561    +7.09759
H    −2.02916    +1.98936    +5.19995
H    −1.81539    +0.27373    +5.44632
C    −0.32625    +0.43278    +7.79113
H    −0.75250    +2.46558    +7.26197
H    −2.16911    +1.49275    +7.55932
H    −0.29956    +0.59600    +8.86611
H    −0.71722    −0.56925    +7.61835
H    +0.70066    +0.45116    +7.43323
24
* E =  +1.231  kcal/mol ; (68) 068_295_176_057_057_175
H    +0.00000    +0.00000    +0.00000
O    +0.00000    +0.00000    +0.95374
C    −0.89964    +0.00000    +1.27039
C    −1.64115    −1.28481    +0.97005
H    −0.82939    +0.14672    +2.34465
H    −1.45062    +0.85259    +0.86163
C    −1.83934    −1.53585    −0.51279
H    −1.09448    −2.11712    +1.41576
H    −2.61003    −1.23707    +1.47001
C    −2.50615    −2.86588    −0.83150
```

H −2.42009 −0.71663 −0.94233
H −0.87133 −1.52091 −1.01709
C −3.86663 −3.07646 −0.18564
H −2.61163 −2.95297 −1.91400
H −1.84132 −3.67449 −0.52467
C −4.88837 −2.00544 −0.51848
H −4.25442 −4.04560 −0.50352
H −3.75840 −3.14351 +0.89853
C −6.26373 −2.32040 +0.03455
H −4.55536 −1.04311 −0.12687
H −4.94561 −1.88732 −1.60233
H −6.98143 −1.53914 −0.20451
H −6.64399 −3.25595 −0.37216
H −6.23489 −2.42283 +1.11818
24
* E = +1.241 kcal/mol ; (69) 178_174_057_057_175_179
H +0.00000 +0.00000 +0.00000
O +0.00000 +0.00000 +0.95350
C −0.89801 +0.00000 +1.27404
C −0.85340 −0.05302 +2.77916
H −1.42561 +0.90347 +0.95259
H −1.44522 −0.86047 +0.87996
C −2.21901 +0.08988 +3.43106
H −0.37752 −0.98585 +3.07969
H −0.20020 +0.74820 +3.12199
C −3.25453 −0.93853 +2.99949
H −2.09212 +0.03000 +4.51251
H −2.60892 +1.08863 +3.22820
C −2.82988 −2.37852 +3.21565
H −4.17717 −0.75428 +3.55210
H −3.50860 −0.79433 +1.94716
C −3.92643 −3.37657 +2.89652
H −1.95543 −2.60545 +2.60208
H −2.51165 −2.51011 +4.25284
C −3.49528 −4.81470 +3.10111
H −4.79756 −3.16299 +3.51789
H −4.25032 −3.23456 +1.86402
H −4.29743 −5.51099 +2.86777
H −2.64619 −5.06294 +2.46647
H −3.19472 −4.98979 +4.13279
24
* E = +1.243 kcal/mol ; (70) 059_182_302_304_186_181
H +0.00000 +0.00000 +0.00000
O +0.00000 +0.00000 +0.95446
C −0.90222 +0.00000 +1.26589

C	−1.69324	−1.18874	+0.76846
H	−0.83185	−0.01441	+2.35075
H	−1.40977	+0.92950	+0.98866
C	−3.14673	−1.18092	+1.21632
H	−1.66324	−1.19773	−0.32343
H	−1.19354	−2.10034	+1.09462
C	−3.35482	−1.13678	+2.72341
H	−3.65510	−0.32730	+0.76508
H	−3.63691	−2.06975	+0.81768
C	−2.67710	−2.26392	+3.47910
H	−3.00970	−0.18033	+3.12089
H	−4.42663	−1.16906	+2.92469
C	−3.01311	−2.27402	+4.95811
H	−2.96437	−3.22106	+3.03648
H	−1.59360	−2.19171	+3.36405
C	−2.32140	−3.39062	+5.71349
H	−2.73815	−1.31210	+5.39388
H	−4.09378	−2.36304	+5.07980
H	−2.57837	−3.37872	+6.77012
H	−2.60196	−4.36447	+5.31558
H	−1.23901	−3.30412	+5.63502

24

* E = +1.246 kcal/mol ; (71) 180_055_056_056_055_058

H	+0.00000	+0.00000	+0.00000
O	+0.00000	+0.00000	+0.95335
C	−0.89770	+0.00000	+1.27431
C	−0.85934	−0.00584	+2.78226
H	−1.43153	+0.88505	+0.91685
H	−1.43799	−0.87938	+0.91648
C	−0.05158	+1.13978	+3.37181
H	−1.88764	+0.02171	+3.14623
H	−0.43456	−0.95200	+3.11575
C	−0.50220	+2.52397	+2.93162
H	−0.09041	+1.06746	+4.45978
H	+0.99120	+1.00743	+3.09042
C	−1.96950	+2.83065	+3.19617
H	+0.12038	+3.26671	+3.43094
H	−0.30023	+2.64403	+1.86653
C	−2.40695	+2.64755	+4.64138
H	−2.17231	+3.85914	+2.89235
H	−2.59462	+2.20306	+2.55804
C	−1.62523	+3.49504	+5.62619
H	−3.46677	+2.89030	+4.71833
H	−2.32060	+1.59622	+4.92075
H	−2.02142	+3.39563	+6.63412

H −0.57598 +3.20782 +5.65842
H −1.66861 +4.54930 +5.35544
24
∗ E = +1.246 kcal/mol ; (72) 065_061_177_058_056_175
H +0.00000 +0.00000 +0.00000
O +0.00000 +0.00000 +0.95449
C −0.90210 +0.00000 +1.26637
C −1.64875 −1.26642 +0.90859
H −0.83384 +0.10041 +2.34758
H −1.43873 +0.87483 +0.89160
C −1.01839 −2.51191 +1.50043
H −2.68097 −1.17000 +1.25269
H −1.70015 −1.34920 −0.18032
C −1.79209 −3.79083 +1.22049
H +0.00439 −2.59902 +1.13635
H −0.93672 −2.38202 +2.58079
C −2.01337 −4.10402 −0.25155
H −1.26217 −4.62573 +1.68172
H −2.76252 −3.73679 +1.71712
C −0.73821 −4.18953 −1.06893
H −2.54685 −5.05287 −0.32891
H −2.67438 −3.35799 −0.69746
C −0.98954 −4.62695 −2.49798
H −0.23596 −3.22105 −1.07036
H −0.04815 −4.88446 −0.58683
H −0.06703 −4.67478 −3.07194
H −1.45127 −5.61239 −2.53002
H −1.66027 −3.93579 −3.00635
24
∗ E = +1.246 kcal/mol ; (73) 179_055_060_178_294_184
H +0.00000 +0.00000 +0.00000
O +0.00000 +0.00000 +0.95331
C −0.89733 +0.00000 +1.27518
C −0.85536 −0.01257 +2.78351
H −1.43219 +0.88360 +0.91831
H −1.43778 −0.87944 +0.91755
C −0.04010 +1.11940 +3.38701
H −1.88240 +0.02731 +3.15041
H −0.44001 −0.96613 +3.10792
C −0.54159 +2.50493 +3.02395
H −0.04062 +0.99690 +4.46958
H +0.99427 +1.01852 +3.05926
C +0.23789 +3.63544 +3.67619
H −0.48757 +2.62898 +1.94226
H −1.59782 +2.59462 +3.29276

```
C +0.09974 +3.71112 +5.18589
H +1.29449 +3.53967 +3.41593
H −0.09234 +4.58470 +3.25045
C +0.81882 +4.90791 +5.77574
H −0.95982 +3.75589 +5.44516
H +0.48631 +2.80014 +5.64190
H +0.71130 +4.94833 +6.85727
H +1.88351 +4.87353 +5.55027
H +0.42833 +5.83927 +5.36858
24
∗ E = +1.253 kcal/mol ; (74) 063_054_058_169_062_175
H +0.00000 +0.00000 +0.00000
O +0.00000 +0.00000 +0.95454
C −0.90146 +0.00000 +1.26842
C −1.66657 −1.24453 +0.86960
H −0.82982 +0.07087 +2.35064
H −1.43186 +0.89092 +0.92392
C −0.97989 −2.54064 +1.26647
H −2.65725 −1.19208 +1.32577
H −1.82885 −1.23488 −0.21066
C −0.71906 −2.65906 +2.75752
H −1.59812 −3.37945 +0.94103
H −0.03822 −2.62037 +0.72538
C −0.28209 −4.04650 +3.19628
H +0.04590 −1.93740 +3.04845
H −1.62596 −2.38513 +3.30141
C +1.02746 −4.51051 +2.58532
H −0.18621 −4.06113 +4.28340
H −1.06644 −4.76726 +2.95208
C +1.48202 −5.84902 +3.13171
H +0.92568 −4.57932 +1.50234
H +1.79471 −3.75680 +2.77060
H +2.41825 −6.16968 +2.68063
H +1.63253 −5.80154 +4.20908
H +0.74009 −6.62251 +2.93903
24
∗ E = +1.253 kcal/mol ; (75) 063_053_059_174_175_063
H +0.00000 +0.00000 +0.00000
O +0.00000 +0.00000 +0.95458
C −0.90156 +0.00000 +1.26828
C −1.67131 −1.23699 +0.85515
H −0.82987 +0.05948 +2.35113
H −1.42864 +0.89645 +0.93303
C −0.98875 −2.54134 +1.23020
H −2.66128 −1.18793 +1.31300
```

H −1.83567 −1.21201 −0.22458
C −0.72836 −2.70001 +2.71624
H −1.60597 −3.37131 +0.88285
H −0.04119 −2.61644 +0.69651
C −0.19020 −4.07493 +3.06717
H −0.01002 −1.94607 +3.04296
H −1.65267 −2.50959 +3.26555
C +0.20230 −4.24084 +4.52584
H −0.93579 −4.83023 +2.80684
H +0.67965 −4.27907 +2.44070
C −0.95600 −4.09794 +5.49461
H +0.65820 −5.22213 +4.65745
H +0.97580 −3.51178 +4.77283
H −0.64223 −4.29934 +6.51634
H −1.37704 −3.09522 +5.47607
H −1.75607 −4.79608 +5.25100
24
* E = +1.308 kcal/mol ; (76) 062_053_059_174_183_296
H +0.00000 +0.00000 +0.00000
O +0.00000 +0.00000 +0.95458
C −0.90145 +0.00000 +1.26860
C −1.67346 −1.23449 +0.85209
H −0.82922 +0.05668 +2.35155
H −1.42774 +0.89800 +0.93619
C −0.99266 −2.54263 +1.21766
H −2.66212 −1.18617 +1.31285
H −1.84095 −1.20399 −0.22704
C −0.72884 −2.71182 +2.70182
H −1.61207 −3.36873 +0.86515
H −0.04616 −2.61572 +0.68173
C −0.18157 −4.08561 +3.04254
H −0.02521 −1.94697 +3.02884
H −1.65441 −2.54490 +3.26022
C +0.03259 −4.32474 +4.52810
H −0.86634 −4.84286 +2.65655
H +0.76353 −4.23383 +2.51496
C +1.07669 −3.42005 +5.15423
H −0.91750 −4.20169 +5.05124
H +0.32670 −5.36377 +4.67680
H +1.24837 −3.68007 +6.19631
H +2.02834 −3.50366 +4.63111
H +0.77764 −2.37487 +5.12518
24
* E = +1.316 kcal/mol ; (77) 065_061_187_302_305_302
H +0.00000 +0.00000 +0.00000

```
O  +0.00000  +0.00000  +0.95446
C  −0.90210  +0.00000  +1.26623
C  −1.65010  −1.26713  +0.91338
H  −0.83396  +0.10314  +2.34723
H  −1.43898  +0.87385  +0.88982
C  −1.02175  −2.51051  +1.51247
H  −2.68270  −1.15930  +1.25009
H  −1.69637  −1.36548  −0.17547
C  −1.65174  −3.80878  +1.03565
H  +0.04028  −2.51465  +1.27201
H  −1.08181  −2.44636  +2.60136
C  −3.14683  −3.92615  +1.29812
H  −1.47759  −3.91615  −0.03641
H  −1.13340  −4.64484  +1.50575
C  −3.55532  −3.72122  +2.74856
H  −3.68352  −3.20941  +0.67508
H  −3.48056  −4.91279  +0.97231
C  −2.88583  −4.68249  +3.71126
H  −3.33652  −2.69550  +3.05070
H  −4.63723  −3.82892  +2.82416
H  −3.26209  −4.55402  +4.72347
H  −3.06565  −5.71643  +3.41938
H  −1.80811  −4.53357  +3.74257
24
* E =  +1.332 kcal/mol ; (78) 180_055_055_058_171_063
H  +0.00000  +0.00000  +0.00000
O  +0.00000  +0.00000  +0.95335
C  −0.89740  +0.00000  +1.27513
C  −0.85740  −0.01081  +2.78298
H  −1.43063  +0.88696  +0.92088
H  −1.43949  −0.87727  +0.91480
C  −0.03381  +1.12355  +3.37428
H  −1.88400  +0.02656  +3.14872
H  −0.44237  −0.96216  +3.11397
C  −0.44349  +2.51295  +2.91384
H  −0.10054  +1.06937  +4.46200
H  +1.01032  +0.96131  +3.11438
C  −1.88899  +2.86379  +3.21885
H  +0.20623  +3.24543  +3.39675
H  −0.24992  +2.60338  +1.84435
C  −2.24320  +4.31757  +2.95207
H  −2.55684  +2.22614  +2.63452
H  −2.09324  +2.63361  +4.26667
C  −2.11176  +4.72183  +1.49611
H  −3.26605  +4.49891  +3.28233
```

H −1.60776 +4.95847 +3.56578
H −2.44321 +5.74578 +1.33927
H −1.08248 +4.65608 +1.15098
H −2.71600 +4.07805 +0.85758
24
* E = +1.333 kcal/mol ; (79) 065_302_305_305_302_185
H +0.00000 +0.00000 +0.00000
O +0.00000 +0.00000 +0.95457
C −0.90230 +0.00000 +1.26613
C −1.65156 −1.26736 +0.91142
H −0.84073 +0.10541 +2.34574
H −1.43716 +0.87613 +0.88868
C −1.71123 −1.54947 −0.58234
H −1.17477 −2.10819 +1.41357
H −2.66180 −1.19077 +1.31804
C −2.29314 −0.42056 −1.42307
H −0.70847 −1.78226 −0.94470
H −2.28898 −2.45899 −0.74375
C −3.67910 +0.04114 −1.00081
H −1.62017 +0.44000 −1.40649
H −2.32809 −0.74584 −2.46376
C −4.72772 −1.05544 −1.00461
H −3.63469 +0.48814 −0.00515
H −4.00042 +0.84066 −1.67011
C −6.11672 −0.53300 −0.69755
H −4.72718 −1.54950 −1.97808
H −4.46243 −1.82230 −0.27576
H −6.85439 −1.33194 −0.69571
H −6.14652 −0.05323 +0.27944
H −6.42906 +0.20530 −1.43418
24
* E = +1.350 kcal/mol ; (80) 182_062_176_063_170_063
H +0.00000 +0.00000 +0.00000
O +0.00000 +0.00000 +0.95330
C −0.89736 +0.00000 +1.27506
C −0.85669 +0.04752 +2.78100
H −1.44172 +0.86901 +0.89345
H −1.42452 −0.89806 +0.94460
C −0.20533 +1.31068 +3.31181
H −1.87930 −0.02942 +3.15548
H −0.32186 −0.83363 +3.13383
C −0.23197 +1.42842 +4.82642
H +0.82357 +1.35053 +2.95536
H −0.71358 +2.17440 +2.87847
C +0.54656 +0.33932 +5.54461

H +0.18134 +2.39817 +5.11137
H −1.26906 +1.42892 +5.16707
C +0.72216 +0.58846 +7.03364
H +0.05342 −0.62424 +5.40425
H +1.53002 +0.24767 +5.07983
C −0.58260 +0.63189 +7.80584
H +1.35787 −0.19319 +7.44957
H +1.26102 +1.52667 +7.17791
H −0.40600 +0.73961 +8.87364
H −1.20797 +1.46532 +7.49358
H −1.15440 −0.28325 +7.65594
24
* E = +1.358 kcal/mol ; (81) 182_063_186_296_181_065
H +0.00000 +0.00000 +0.00000
O +0.00000 +0.00000 +0.95328
C −0.89731 +0.00000 +1.27510
C −0.85993 +0.04719 +2.78147
H −1.44233 +0.86820 +0.89278
H −1.42368 −0.89840 +0.94466
C −0.21702 +1.31438 +3.31396
H −1.88196 −0.05209 +3.14661
H −0.30814 −0.82362 +3.13928
C −0.03106 +1.33127 +4.82209
H +0.75051 +1.43336 +2.82980
H −0.82076 +2.17506 +3.01499
C −1.32761 +1.29605 +5.61126
H +0.59025 +0.48192 +5.11701
H +0.53199 +2.22514 +5.08931
C −1.13893 +1.33412 +7.11966
H −1.95478 +2.13813 +5.30646
H −1.88408 +0.39434 +5.35510
C −0.52759 +2.62291 +7.63555
H −2.10697 +1.18474 +7.59796
H −0.51830 +0.48922 +7.42337
H −0.48297 +2.62910 +8.72229
H +0.48597 +2.76744 +7.26896
H −1.11675 +3.48471 +7.32433
24
* E = +1.371 kcal/mol ; (82) 066_296_187_302_306_302
H +0.00000 +0.00000 +0.00000
O +0.00000 +0.00000 +0.95403
C −0.90097 +0.00000 +1.26777
C −1.65088 −1.26791 +0.92169
H −0.83418 +0.11411 +2.34621
H −1.44133 +0.86961 +0.88104

```
C   -1.81607   -1.47768   -0.57219
H   -1.12400   -2.11315   +1.36463
H   -2.63544   -1.22452   +1.39240
C   -2.70415   -2.65742   -0.93568
H   -2.23702   -0.57255   -1.01578
H   -0.83516   -1.61796   -1.03284
C   -2.24109   -3.99551   -0.37570
H   -3.71676   -2.46139   -0.57997
H   -2.77609   -2.72253   -2.02157
C   -0.80358   -4.36078   -0.71174
H   -2.36296   -3.99854   +0.70786
H   -2.90406   -4.77555   -0.75343
C   -0.51843   -4.40405   -2.20041
H   -0.12328   -3.65572   -0.23109
H   -0.58073   -5.33323   -0.27339
H   +0.49649   -4.74047   -2.39845
H   -1.19902   -5.08505   -2.70995
H   -0.63218   -3.42379   -2.66033
24
 * E =  +1.372 kcal/mol ; (83) 066_061_187_298_189_297
H   +0.00000   +0.00000   +0.00000
O   +0.00000   +0.00000   +0.95445
C   -0.90211   +0.00000   +1.26617
C   -1.64887   -1.26879   +0.91592
H   -0.83416   +0.10577   +2.34688
H   -1.43980   +0.87242   +0.88765
C   -1.01706   -2.50993   +1.51739
H   -2.68104   -1.16068   +1.25128
H   -1.69250   -1.36973   -0.17284
C   -1.63848   -3.81199   +1.04226
H   +0.04602   -2.50467   +1.28113
H   -1.08835   -2.45099   +2.60600
C   -3.10500   -3.96524   +1.40668
H   -1.51546   -3.88903   -0.03948
H   -1.08102   -4.64720   +1.47086
C   -3.66881   -5.34949   +1.13046
H   -3.22785   -3.73659   +2.46745
H   -3.70044   -3.22735   +0.86544
C   -3.65120   -5.73682   -0.33591
H   -3.10677   -6.08584   +1.70761
H   -4.69354   -5.39316   +1.49931
H   -4.12957   -6.70051   -0.49488
H   -4.18148   -5.00101   -0.93944
H   -2.63679   -5.80966   -0.72153
24
```

```
 * E =   +1.373  kcal/mol  ;  (84)  180_179_175_058_054_058
 H    +0.00000    +0.00000    +0.00000
 O    +0.00000    +0.00000    +0.95350
 C    −0.89823    +0.00000    +1.27340
 C    −0.86503    +0.00045    +2.77898
 H    −1.43684    +0.88275    +0.91678
 H    −1.43679    −0.88246    +0.91647
 C    −2.25441    +0.02520    +3.38646
 H    −0.32283    −0.88412    +3.11531
 H    −0.28657    +0.86415    +3.10594
 C    −2.26310    −0.10205    +4.90156
 H    −2.76228    +0.94637    +3.09195
 H    −2.84666    −0.78944    +2.96612
 C    −1.46605    +0.96773    +5.63505
 H    −3.29649    −0.08830    +5.24900
 H    −1.86797    −1.08075    +5.17754
 C    −1.84852    +2.39733    +5.28525
 H    −1.59451    +0.81887    +6.70849
 H    −0.40266    +0.82765    +5.43911
 C    −3.30938    +2.71633    +5.53703
 H    −1.22509    +3.07622    +5.86666
 H    −1.60815    +2.59629    +4.23942
 H    −3.52376    +3.76516    +5.34549
 H    −3.96517    +2.12757    +4.89802
 H    −3.58330    +2.50523    +6.57001
24
 * E =   +1.382  kcal/mol  ;  (85)  060_172_057_056_174_179
 H    +0.00000    +0.00000    +0.00000
 O    +0.00000    +0.00000    +0.95446
 C    −0.90205    +0.00000    +1.26640
 C    −1.67805    −1.21252    +0.80354
 H    −0.83699    +0.00869    +2.35263
 H    −1.40961    +0.92087    +0.96688
 C    −3.05319    −1.33916    +1.44092
 H    −1.77355    −1.18380    −0.28446
 H    −1.08760    −2.09778    +1.03649
 C    −3.97755    −0.14722    +1.23658
 H    −3.53684    −2.23293    +1.04522
 H    −2.93116    −1.51103    +2.51131
 C    −4.21398    +0.21721    −0.21665
 H    −4.93696    −0.37077    +1.70549
 H    −3.58746    +0.72450    +1.76534
 C    −5.24882    +1.31161    −0.39507
 H    −3.27740    +0.54005    −0.67699
 H    −4.52964    −0.67249    −0.76746
```

```
 C    −5.46900    +1.68707    −1.84639
 H    −6.19211    +0.98747    +0.04707
 H    −4.93831    +2.19289    +0.16852
 H    −6.21628    +2.47042    −1.94879
 H    −4.54804    +2.04738    −2.30192
 H    −5.80728    +0.82942    −2.42556
24
 * E =   +1.383  kcal/mol  ;  (86) 061_177_174_058_054_058
 H    +0.00000    +0.00000    +0.00000
 O    +0.00000    +0.00000    +0.95437
 C    −0.90179    +0.00000    +1.26677
 C    −1.68568    −1.21370    +0.82172
 H    −0.83536    +0.02212    +2.35220
 H    −1.41909    +0.91317    +0.95792
 C    −3.09012    −1.23542    +1.39510
 H    −1.73958    −1.22884    −0.27063
 H    −1.13442    −2.10564    +1.11882
 C    −3.95558    −2.36737    +0.86481
 H    −3.03097    −1.29556    +2.48387
 H    −3.58312    −0.28695    +1.17539
 C    −3.38999    −3.76220    +1.09357
 H    −4.94358    −2.29622    +1.32019
 H    −4.10976    −2.22513    −0.20595
 C    −3.04476    −4.07880    +2.54031
 H    −4.11655    −4.49278    +0.73418
 H    −2.49956    −3.89935    +0.47915
 C    −4.21864    −3.94307    +3.49046
 H    −2.65583    −5.09555    +2.58999
 H    −2.23047    −3.43331    +2.87397
 H    −3.94927    −4.25227    +4.49760
 H    −4.57234    −2.91523    +3.54881
 H    −5.05642    −4.55977    +3.16734
24
 * E =   +1.393  kcal/mol  ;  (87) 065_302_302_189_297_184
 H    +0.00000    +0.00000    +0.00000
 O    +0.00000    +0.00000    +0.95465
 C    −0.90206    +0.00000    +1.26713
 C    −1.65285    −1.26604    +0.90917
 H    −0.83919    +0.10005    +2.34719
 H    −1.43630    +0.87795    +0.89500
 C    −1.70942    −1.55032    −0.58366
 H    −1.18262    −2.10661    +1.41800
 H    −2.66743    −1.18076    +1.30310
 C    −2.33736    −0.43500    −1.40275
 H    −0.70281    −1.76394    −0.94677
```

H −2.27567 −2.46898 −0.74436
C −2.59854 −0.80563 −2.85286
H −3.27846 −0.14114 −0.93419
H −1.70236 +0.45465 −1.37730
C −1.34808 −1.12236 −3.65269
H −3.27349 −1.66389 −2.88865
H −3.12736 +0.01544 −3.33954
C −1.64351 −1.38335 −5.11604
H −0.64708 −0.28954 −3.56448
H −0.84575 −1.99087 −3.22699
H −0.73822 −1.61218 −5.67327
H −2.32495 −2.22445 −5.23243
H −2.11002 −0.51697 −5.58191
24
* E = +1.402 kcal/mol ; (88) 061_178_185_302_306_302
H +0.00000 +0.00000 +0.00000
O +0.00000 +0.00000 +0.95437
C −0.90187 +0.00000 +1.26654
C −1.68577 −1.21229 +0.81799
H −0.83554 +0.02052 +2.35190
H −1.41879 +0.91390 +0.95883
C −3.10336 −1.21583 +1.35842
H −1.70553 −1.23729 −0.27435
H −1.15687 −2.11006 +1.14040
C −3.88799 −2.47498 +1.02517
H −3.07129 −1.10219 +2.44308
H −3.63586 −0.33940 +0.98247
C −4.01174 −2.77457 −0.46231
H −3.41496 −3.32780 +1.51391
H −4.88296 −2.39386 +1.46343
C −4.58072 −1.63639 −1.29503
H −3.03525 −3.05215 −0.86067
H −4.64487 −3.65463 −0.58598
C −5.95498 −1.18024 −0.84504
H −3.89361 −0.78834 −1.27744
H −4.62947 −1.95707 −2.33545
H −6.35677 −0.42004 −1.51080
H −6.65730 −2.01267 −0.83027
H −5.92955 −0.75574 +0.15696
24
* E = +1.423 kcal/mol ; (89) 065_302_303_186_185_297
H +0.00000 +0.00000 +0.00000
O +0.00000 +0.00000 +0.95459
C −0.90211 +0.00000 +1.26673
C −1.65184 −1.26724 +0.91127

H	−0.83979	+0.10257	+2.34658
H	−1.43688	+0.87676	+0.89249
C	−1.70682	−1.55853	−0.58007
H	−1.18221	−2.10614	+1.42345
H	−2.66679	−1.18157	+1.30395
C	−2.32672	−0.45288	−1.41605
H	−0.70115	−1.77418	−0.94860
H	−2.27202	−2.47812	−0.73537
C	−2.49088	−0.84449	−2.87311
H	−3.29432	−0.18413	−0.98905
H	−1.71486	+0.45207	−1.36282
C	−2.99291	+0.27461	−3.77021
H	−1.53104	−1.20030	−3.25184
H	−3.17473	−1.69379	−2.93893
C	−4.37914	+0.77480	−3.41178
H	−2.28604	+1.10556	−3.73633
H	−2.99575	−0.07749	−4.80157
H	−4.72556	+1.51877	−4.12528
H	−5.09973	−0.04209	−3.41023
H	−4.40104	+1.23532	−2.42662

24

∗ E = +1.432 kcal/mol ; (90) 063_054_056_055_055_058

H	+0.00000	+0.00000	+0.00000
O	+0.00000	+0.00000	+0.95458
C	−0.90194	+0.00000	+1.26719
C	−1.67088	−1.23768	+0.85648
H	−0.83126	+0.05982	+2.35071
H	−1.42878	+0.89637	+0.93134
C	−0.98711	−2.53947	+1.24434
H	−2.66612	−1.18107	+1.30141
H	−1.82640	−1.22228	−0.22464
C	−0.67123	−2.66682	+2.72676
H	−1.61673	−3.37186	+0.92639
H	−0.05645	−2.62964	+0.68614
C	−1.86915	−2.48876	+3.64842
H	−0.21916	−3.64318	+2.90188
H	+0.09513	−1.93849	+2.99277
C	−3.04241	−3.41006	+3.35142
H	−1.54411	−2.64886	+4.67774
H	−2.21431	−1.45404	+3.60368
C	−2.69114	−4.88373	+3.41574
H	−3.84112	−3.19817	+4.06209
H	−3.45067	−3.17869	+2.36607
H	−3.57169	−5.50612	+3.27435
H	−1.97060	−5.15872	+2.64772

H −2.25543 −5.14000 +4.38062
24
∗ E = +1.434 kcal/mol ; (91) 062_053_059_176_293_184
H +0.00000 +0.00000 +0.00000
O +0.00000 +0.00000 +0.95460
C −0.90158 +0.00000 +1.26831
C −1.67656 −1.22921 +0.84199
H −0.82966 +0.05009 +2.35148
H −1.42569 +0.90133 +0.94144
C −1.00273 −2.54476 +1.19592
H −2.66579 −1.18129 +1.30164
H −1.84292 −1.18898 −0.23701
C −0.74622 −2.72419 +2.68100
H −1.62581 −3.35743 +0.82412
H −0.05194 −2.61460 +0.66645
C −0.15440 −4.07577 +3.04669
H −0.06298 −1.94488 +3.01727
H −1.67899 −2.57874 +3.23284
C −1.08488 −5.25503 +2.82837
H +0.76502 −4.23138 +2.47757
H +0.14207 −4.05878 +4.09679
C −0.47913 −6.56616 +3.28798
H −2.01905 −5.07552 +3.36401
H −1.34920 −5.33276 +1.77401
H −1.15763 −7.40066 +3.12636
H +0.44282 −6.78029 +2.74976
H −0.23826 −6.53537 +4.34938
24
∗ E = +1.450 kcal/mol ; (92) 065_061_178_059_054_058
H +0.00000 +0.00000 +0.00000
O +0.00000 +0.00000 +0.95452
C −0.90223 +0.00000 +1.26612
C −1.65187 −1.26145 +0.89732
H −0.83423 +0.09149 +2.34814
H −1.43651 +0.87917 +0.89814
C −1.02702 −2.51317 +1.48226
H −2.68468 −1.16483 +1.23934
H −1.70043 −1.33599 −0.19215
C −1.78406 −3.79217 +1.16240
H +0.00435 −2.58745 +1.13750
H −0.96854 −2.40028 +2.56601
C −1.93924 −4.09079 −0.32300
H −1.27833 −4.62755 +1.64725
H −2.77713 −3.74400 +1.61246
C −0.63707 −4.10057 −1.10830

```
H    -2.42505   -5.06190   -0.43175
H    -2.61950   -3.36691   -0.77329
C    +0.38199   -5.09367   -0.58464
H    -0.86057   -4.32726   -2.15072
H    -0.19960   -3.10051   -1.10714
H    +1.26519   -5.12470   -1.21830
H    +0.71120   -4.83777   +0.42061
H    -0.03561   -6.09909   -0.54807
24
* E =   +1.452  kcal/mol ; (93) 067_296_178_058_054_058
H    +0.00000   +0.00000   +0.00000
O    +0.00000   +0.00000   +0.95378
C    -0.90001   +0.00000   +1.26949
C    -1.64645   -1.27885   +0.95668
H    -0.83064   +0.13731   +2.34505
H    -1.44758   +0.85795   +0.86729
C    -1.83389   -1.52015   -0.52935
H    -1.10901   -2.11758   +1.40141
H    -2.61919   -1.22765   +1.44841
C    -2.54580   -2.82293   -0.86023
H    -2.38173   -0.67954   -0.96335
H    -0.85972   -1.53706   -1.02139
C    -3.93268   -2.96548   -0.24843
H    -2.61547   -2.91863   -1.94399
H    -1.92916   -3.65815   -0.52555
C    -4.88168   -1.81624   -0.54990
H    -4.37502   -3.89488   -0.61067
H    -3.84550   -3.07931   +0.83244
C    -5.10034   -1.57922   -2.03151
H    -5.83928   -2.02117   -0.07183
H    -4.50882   -0.90063   -0.08710
H    -5.84003   -0.80059   -2.20216
H    -4.18246   -1.27153   -2.52934
H    -5.45225   -2.48460   -2.52435
24
* E =   +1.456  kcal/mol ; (94) 179_077_299_301_185_181
H    +0.00000   +0.00000   +0.00000
O    +0.00000   +0.00000   +0.95360
C    -0.89850   +0.00000   +1.27304
C    -0.88513   -0.02937   +2.78226
H    -1.43170   +0.88973   +0.92364
H    -1.42995   -0.87656   +0.89569
C    -0.55771   +1.29544   +3.45515
H    -1.87234   -0.34877   +3.11701
H    -0.18822   -0.80666   +3.09555
```

```
C +0.80805 +1.88747 +3.14453
H -1.32792 +2.02024 +3.18403
H -0.64280 +1.15679 +4.53457
C +1.97168 +0.99193 +3.52108
H +0.87482 +2.12228 +2.08271
H +0.89801 +2.83463 +3.68049
C +3.31896 +1.65906 +3.32215
H +1.87328 +0.68368 +4.56583
H +1.93455 +0.08420 +2.91914
C +4.48311 +0.75868 +3.68321
H +3.40842 +1.97248 +2.28088
H +3.36290 +2.57194 +3.91901
H +5.43874 +1.25597 +3.53200
H +4.43143 +0.45182 +4.72682
H +4.47951 -0.14496 +3.07599
24
* E = +1.457 kcal/mol ; (95) 179_175_058_053_058_175
H +0.00000 +0.00000 +0.00000
O +0.00000 +0.00000 +0.95351
C -0.89809 +0.00000 +1.27384
C -0.85406 -0.03380 +2.77964
H -1.42926 +0.89755 +0.94201
H -1.44167 -0.86738 +0.89044
C -2.22263 +0.08986 +3.42799
H -0.36288 -0.95613 +3.09100
H -0.21460 +0.78197 +3.11392
C -3.22364 -0.98542 +3.02347
H -2.09513 +0.07787 +4.51006
H -2.64575 +1.06721 +3.19057
C -2.73426 -2.41301 +3.20813
H -4.13587 -0.84488 +3.60542
H -3.51290 -0.84307 +1.98130
C -2.32487 -2.75024 +4.62965
H -3.52468 -3.09736 +2.89543
H -1.89221 -2.60708 +2.53952
C -1.94936 -4.20872 +4.79772
H -1.48267 -2.12475 +4.92780
H -3.14352 -2.50027 +5.30736
H -1.65414 -4.43190 +5.82030
H -2.78436 -4.85979 +4.54393
H -1.11732 -4.47471 +4.14776
24
* E = +1.463 kcal/mol ; (96) 180_179_174_062_171_063
H +0.00000 +0.00000 +0.00000
O +0.00000 +0.00000 +0.95349
```

```
C   −0.89788   +0.00000   +1.27435
C   −0.86144   −0.00105   +2.78003
H   −1.43661   +0.88316   +0.91907
H   −1.43703   −0.88216   +0.91750
C   −2.24993   +0.02161   +3.39074
H   −0.31982   −0.88728   +3.11305
H   −0.28177   +0.86135   +3.10556
C   −2.25791   −0.11337   +4.90440
H   −2.75466   +0.94927   +3.11118
H   −2.84254   −0.78850   +2.96184
C   −1.54930   +1.01870   +5.62739
H   −3.29278   −0.15737   +5.24976
H   −1.80510   −1.06800   +5.17619
C   −1.74907   +1.00604   +7.13404
H   −0.48013   +0.98340   +5.41200
H   −1.90922   +1.96955   +5.22881
C   −1.18971   −0.22822   +7.81540
H   −1.27904   +1.89288   +7.55892
H   −2.81462   +1.09192   +7.35468
H   −1.28873   −0.16069   +8.89636
H   −1.70401   −1.13244   +7.49746
H   −0.13197   −0.35331   +7.58720
24
* E =   +1.468  kcal/mol ; (97) 065_302_302_185_177_064
H   +0.00000   +0.00000   +0.00000
O   +0.00000   +0.00000   +0.95460
C   −0.90211   +0.00000   +1.26678
C   −1.65114   −1.26856   +0.91422
H   −0.83982   +0.10412   +2.34651
H   −1.43719   +0.87619   +0.89174
C   −1.70314   −1.56830   −0.57555
H   −1.18234   −2.10507   +1.43108
H   −2.66685   −1.18158   +1.30464
C   −2.32668   −0.47066   −1.41914
H   −0.69564   −1.78055   −0.94121
H   −2.26272   −2.49213   −0.72571
C   −2.47138   −0.86409   −2.87764
H   −3.30963   −0.21645   −1.01429
H   −1.72920   +0.43995   −1.34571
C   −3.15499   +0.17999   −3.74501
H   −1.48366   −1.08335   −3.29048
H   −3.03492   −1.79678   −2.93315
C   −2.38736   +1.48278   −3.86384
H   −3.30459   −0.23670   −4.74083
H   −4.15172   +0.37886   −3.34754
```

```
H    -2.87624    +2.16536    -4.55498
H    -2.30540    +1.99502    -2.90782
H    -1.37744    +1.30773    -4.23282
24
 * E =  +1.473  kcal/mol ;  (98)  059_182_301_307_302_185
H    +0.00000    +0.00000    +0.00000
O    +0.00000    +0.00000    +0.95447
C    -0.90234    +0.00000    +1.26558
C    -1.69247    -1.19124    +0.77205
H    -0.83293    -0.01007    +2.35046
H    -1.41122    +0.92743    +0.98397
C    -3.14287    -1.18753    +1.22783
H    -1.66595    -1.20208    -0.31989
H    -1.18910    -2.10163    +1.09901
C    -3.33457    -1.17249    +2.73940
H    -3.65058    -0.32008    +0.80311
H    -3.64280    -2.05937    +0.80676
C    -2.59725    -2.27266    +3.48607
H    -3.02550    -0.20567    +3.13826
H    -4.40174    -1.25279    +2.95159
C    -2.95978    -3.67683    +3.03998
H    -1.51834    -2.13345    +3.38558
H    -2.80945    -2.17389    +4.55179
C    -2.29086    -4.74620    +3.87987
H    -4.04387    -3.79849    +3.08376
H    -2.68167    -3.81405    +1.99419
H    -2.55583    -5.74560    +3.54277
H    -1.20671    -4.65768    +3.83305
H    -2.58122    -4.66099    +4.92572
24
 * E =  +1.478  kcal/mol ;  (99)  061_177_174_062_171_063
H    +0.00000    +0.00000    +0.00000
O    +0.00000    +0.00000    +0.95436
C    -0.90201    +0.00000    +1.26611
C    -1.68420    -1.21508    +0.82132
H    -0.83654    +0.02362    +2.35154
H    -1.41963    +0.91241    +0.95546
C    -3.09115    -1.23517    +1.38985
H    -1.73585    -1.23035    -0.27120
H    -1.13277    -2.10508    +1.12095
C    -3.95811    -2.36436    +0.85815
H    -3.03692    -1.30573    +2.47838
H    -3.57749    -0.28290    +1.17097
C    -3.43765    -3.75103    +1.19381
H    -4.96497    -2.25868    +1.26681
```

```
 H   -4.05611   -2.25617   -0.22319
 C   -4.40983   -4.87044   +0.85932
 H   -2.49760   -3.93076   +0.66932
 H   -3.20359   -3.78945   +2.25965
 C   -4.72886   -4.98331   -0.61918
 H   -3.99253   -5.81491   +1.20810
 H   -5.33401   -4.72130   +1.42050
 H   -5.36814   -5.83985   -0.82011
 H   -5.24363   -4.09957   -0.98962
 H   -3.81901   -5.10515   -1.20572
24
 * E =  +1.498  kcal/mol ; (100) 181_062_176_065_179_295
 H   +0.00000   +0.00000   +0.00000
 O   +0.00000   +0.00000   +0.95330
 C   -0.89722   +0.00000   +1.27544
 C   -0.85578   +0.03537   +2.78182
 H   -1.43915   +0.87350   +0.90077
 H   -1.42717   -0.89395   +0.93835
 C   -0.20020   +1.29298   +3.32080
 H   -1.87857   -0.04008   +3.15603
 H   -0.32490   -0.85146   +3.12565
 C   -0.22040   +1.40622   +4.83639
 H   +0.82716   +1.33536   +2.96031
 H   -0.70937   +2.15952   +2.89421
 C   +0.59130   +0.33984   +5.55033
 H   +0.15634   +2.39084   +5.11233
 H   -1.25378   +1.36596   +5.19137
 C   +0.58586   +0.46632   +7.06571
 H   +0.21100   -0.64604   +5.28347
 H   +1.62205   +0.37541   +5.18930
 C   +1.25168   +1.72620   +7.58566
 H   -0.44401   +0.42365   +7.42497
 H   +1.08984   -0.40159   +7.49096
 H   +1.28886   +1.73092   +8.67272
 H   +2.27465   +1.80468   +7.21965
 H   +0.72160   +2.62388   +7.27601
24
 * E =  +1.504  kcal/mol ; (101) 061_178_185_298_190_297
 H   +0.00000   +0.00000   +0.00000
 O   +0.00000   +0.00000   +0.95433
 C   -0.90188   +0.00000   +1.26636
 C   -1.68178   -1.21813   +0.82607
 H   -0.83571   +0.02854   +2.35154
 H   -1.42109   +0.91026   +0.95190
 C   -3.10015   -1.22138   +1.36567
```

H	−1.69800	−1.25226	−0.26525
H	−1.15108	−2.11112	+1.15880
C	−3.87930	−2.48780	+1.05182
H	−3.06620	−1.08683	+2.44804
H	−3.64108	−0.35924	+0.96894
C	−4.08525	−2.73359	−0.43270
H	−3.36619	−3.33910	+1.50132
H	−4.85583	−2.42987	+1.53653
C	−5.05353	−3.86419	−0.73998
H	−4.45416	−1.81457	−0.89318
H	−3.12723	−2.95044	−0.90865
C	−4.59234	−5.21875	−0.23713
H	−6.02761	−3.62805	−0.30796
H	−5.20445	−3.91525	−1.81818
H	−5.27746	−6.00626	−0.54230
H	−3.60745	−5.46618	−0.63134
H	−4.52860	−5.24580	+0.84837

24

* E = +1.511 kcal/mol ; (102) 179_055_054_058_178_295

H	+0.00000	+0.00000	+0.00000
O	+0.00000	+0.00000	+0.95334
C	−0.89745	+0.00000	+1.27497
C	−0.85795	−0.01675	+2.78252
H	−1.42978	+0.88883	+0.92385
H	−1.44058	−0.87523	+0.91126
C	−0.04007	+1.11850	+3.38027
H	−1.88463	+0.01056	+3.14836
H	−0.43683	−0.96713	+3.10871
C	−0.43966	+2.51194	+2.92163
H	−0.11368	+1.06252	+4.46744
H	+1.00615	+0.95636	+3.12877
C	−1.88526	+2.87332	+3.21032
H	+0.22038	+3.22936	+3.40830
H	−0.24727	+2.61028	+1.85091
C	−2.27056	+4.28347	+2.79158
H	−2.54387	+2.16880	+2.69999
H	−2.08084	+2.74897	+4.27876
C	−1.55473	+5.37592	+3.56288
H	−2.07689	+4.40470	+1.72415
H	−3.34611	+4.40641	+2.91928
H	−1.91758	+6.36074	+3.27743
H	−1.71403	+5.26322	+4.63455
H	−0.48187	+5.36191	+3.38623

24

* E = +1.514 kcal/mol ; (103) 059_182_297_191_297_184

```
H    +0.00000   +0.00000   +0.00000
O    +0.00000   +0.00000   +0.95449
C    −0.90197   +0.00000   +1.26675
C    −1.69085   −1.19441   +0.77714
H    −0.83068   −0.00491   +2.35077
H    −1.41296   +0.92495   +0.98049
C    −3.14076   −1.19243   +1.23199
H    −1.65863   −1.20803   −0.31475
H    −1.19176   −2.10345   +1.11431
C    −3.31145   −1.27599   +2.74013
H    −3.63444   −0.29705   +0.85090
H    −3.65399   −2.03903   +0.77298
C    −4.74122   −1.53618   +3.18305
H    −2.67037   −2.07300   +3.12149
H    −2.95972   −0.35410   +3.20668
C    −5.72230   −0.44131   +2.80578
H    −5.08249   −2.48419   +2.76026
H    −4.75914   −1.66479   +4.26654
C    −7.11227   −0.69061   +3.35584
H    −5.34810   +0.51600   +3.17389
H    −5.77599   −0.35016   +1.72092
H    −7.80357   +0.09983   +3.07310
H    −7.51608   −1.63137   +2.98506
H    −7.09885   −0.74628   +4.44309
24
 * E =   +1.515  kcal/mol ;  (104) 066_296_187_297_189_297
H    +0.00000   +0.00000   +0.00000
O    +0.00000   +0.00000   +0.95400
C    −0.90083   +0.00000   +1.26804
C    −1.65167   −1.26751   +0.92236
H    −0.83378   +0.11389   +2.34651
H    −1.44117   +0.86978   +0.88158
C    −1.82191   −1.47375   −0.57209
H    −1.12461   −2.11239   +1.36377
H    −2.63561   −1.22222   +1.39438
C    −2.72068   −2.64363   −0.93801
H    −2.23250   −0.56105   −1.01030
H    −0.84417   −1.63016   −1.03447
C    −2.19605   −3.99055   −0.47185
H    −3.71448   −2.46778   −0.52335
H    −2.84333   −2.67047   −2.02255
C    −2.97045   −5.17599   −1.02393
H    −1.14864   −4.08065   −0.76679
H    −2.20955   −4.03598   +0.61837
C    −4.42260   −5.21583   −0.58791
```

H −2.91805 −5.16154 −2.11404
H −2.47730 −6.09542 −0.70900
H −4.91619 −6.11717 −0.94381
H −4.50362 −5.20168 +0.49832
H −4.98290 −4.36577 −0.97097
24
* E = +1.520 kcal/mol ; (105) 065_061_188_297_181_065
H +0.00000 +0.00000 +0.00000
O +0.00000 +0.00000 +0.95449
C −0.90218 +0.00000 +1.26614
C −1.65530 −1.26095 +0.90096
H −0.83411 +0.09304 +2.34802
H −1.43618 +0.87899 +0.89762
C −1.03082 −2.51182 +1.49027
H −2.68728 −1.14807 +1.23461
H −1.69668 −1.34967 −0.18900
C −1.63891 −3.81108 +0.98918
H +0.03492 −2.50066 +1.26643
H −1.11322 −2.47004 +2.57882
C −3.10322 −3.99181 +1.34697
H −1.52781 −3.86593 −0.09698
H −1.06061 −4.64090 +1.39440
C −3.70404 −5.30328 +0.86600
H −3.21726 −3.92092 +2.43190
H −3.68334 −3.17019 +0.92626
C −3.10119 −6.53563 +1.51309
H −4.77636 −5.28939 +1.06079
H −3.59486 −5.37027 −0.21797
H −3.61537 −7.43879 +1.19240
H −2.05004 −6.65473 +1.26113
H −3.17591 −6.48096 +2.59843
24
* E = +1.521 kcal/mol ; (106) 063_053_054_058_171_063
H +0.00000 +0.00000 +0.00000
O +0.00000 +0.00000 +0.95455
C −0.90151 +0.00000 +1.26831
C −1.67149 −1.23700 +0.85781
H −0.82868 +0.05768 +2.35201
H −1.42850 +0.89739 +0.93556
C −0.97361 −2.53909 +1.22159
H −2.65914 −1.18949 +1.31890
H −1.84469 −1.21341 −0.22042
C −0.59131 −2.66234 +2.68754
H −1.62281 −3.37104 +0.94441
H −0.07134 −2.63481 +0.61935

C −1.76617 −2.54487 +3.64223
H −0.10864 −3.62945 +2.84042
H +0.16390 −1.91158 +2.92102
C −1.42521 −2.87878 +5.08532
H −2.17656 −1.53286 +3.60331
H −2.56493 −3.20748 +3.30196
C −0.40686 −1.94159 +5.70582
H −2.33948 −2.85707 +5.67845
H −1.05450 −3.90408 +5.13623
H −0.24601 −2.17401 +6.75605
H +0.55721 −2.00530 +5.20664
H −0.74160 −0.90667 +5.64438
24
* E = +1.531 kcal/mol ; (107) 177_174_062_169_063_176
H +0.00000 +0.00000 +0.00000
O +0.00000 +0.00000 +0.95350
C −0.89801 +0.00000 +1.27402
C −0.85217 −0.06883 +2.77922
H −1.42088 +0.91054 +0.96469
H −1.44864 −0.85248 +0.87014
C −2.21634 +0.05721 +3.43471
H −0.38108 −1.00835 +3.06962
H −0.19751 +0.72921 +3.12698
C −3.18396 −1.05756 +3.07173
H −2.08246 +0.06592 +4.51768
H −2.65025 +1.02462 +3.17695
C −4.44207 −1.08229 +3.92306
H −3.47078 −0.97516 +2.02152
H −2.66840 −2.01477 +3.17120
C −5.31018 +0.15574 +3.79221
H −5.03650 −1.95725 +3.65440
H −4.16454 −1.21556 +4.97143
C −6.60119 +0.04735 +4.57849
H −4.75679 +1.03254 +4.12827
H −5.53594 +0.32387 +2.73728
H −7.20915 +0.94341 +4.47771
H −7.19722 −0.79791 +4.23819
H −6.40145 −0.09909 +5.63878
24
* E = +1.531 kcal/mol ; (108) 180_057_063_181_302_304
H +0.00000 +0.00000 +0.00000
O +0.00000 +0.00000 +0.95330
C −0.89743 +0.00000 +1.27485
C −0.85633 −0.00852 +2.78314
H −1.43413 +0.88046 +0.91333

```
H   −1.43551   −0.88238   +0.92076
C   −0.07378   +1.14485   +3.38921
H   −1.88409   +0.00206   +3.15031
H   −0.41487   −0.95090   +3.10580
C   −0.65457   +2.51380   +3.08958
H   −0.03229   +0.99513   +4.46850
H   +0.95364   +1.10020   +3.02820
C   +0.14799   +3.66471   +3.67844
H   −0.70728   +2.65873   +2.01054
H   −1.68480   +2.55762   +3.45026
C   +0.33847   +3.61776   +5.18720
H   +1.12842   +3.69078   +3.20046
H   −0.34396   +4.60306   +3.41582
C   −0.96166   +3.54482   +5.96376
H   +0.96890   +2.76930   +5.45406
H   +0.89193   +4.50568   +5.49267
H   −0.78593   +3.60412   +7.03540
H   −1.62788   +4.36254   +5.69100
H   −1.49130   +2.61325   +5.77174
24
* E =   +1.538  kcal/mol ; (109) 064_061_178_064_170_063
H   +0.00000   +0.00000   +0.00000
O   +0.00000   +0.00000   +0.95453
C   −0.90229   +0.00000   +1.26597
C   −1.65833   −1.25417   +0.88534
H   −0.83378   +0.08034   +2.34886
H   −1.43333   +0.88494   +0.90707
C   −1.03765   −2.51575   +1.45442
H   −2.68837   −1.15655   +1.23553
H   −1.71354   −1.31541   −0.20389
C   −1.80578   −3.78639   +1.13143
H   −0.01289   −2.60226   +1.09273
H   −0.96630   −2.40717   +2.53795
C   −1.86036   −4.11758   −0.35028
H   −1.33984   −4.62190   +1.65766
H   −2.81829   −3.70366   +1.53034
C   −2.41575   −5.49992   −0.65204
H   −2.46260   −3.37446   −0.87628
H   −0.85235   −4.04173   −0.76327
C   −3.85516   −5.69055   −0.21367
H   −2.34098   −5.68400   −1.72377
H   −1.78658   −6.25053   −0.17048
H   −4.23316   −6.66494   −0.51452
H   −3.96034   −5.62004   +0.86662
H   −4.50107   −4.93398   −0.65780
```

```
24
 * E =   +1.538  kcal/mol  ;  (110) 059_182_297_186_185_297
 H    +0.00000    +0.00000    +0.00000
 O    +0.00000    +0.00000    +0.95450
 C    −0.90245    +0.00000    +1.26540
 C    −1.68982    −1.19480    +0.77408
 H    −0.83286    −0.00383    +2.34946
 H    −1.41294    +0.92467    +0.97732
 C    −3.14504    −1.18824    +1.21050
 H    −1.64593    −1.21329    −0.31736
 H    −1.19664    −2.10388    +1.11942
 C    −3.34920    −1.26742    +2.71327
 H    −3.63638    −0.29083    +0.82753
 H    −3.65461    −2.03144    +0.74262
 C    −4.81175    −1.40722    +3.09338
 H    −2.77862    −2.11184    +3.10342
 H    −2.94397    −0.37567    +3.19539
 C    −5.07709    −1.37168    +4.58924
 H    −5.37660    −0.60582    +2.61384
 H    −5.20106    −2.34086    +2.68042
 C    −4.44031    −2.51597    +5.35443
 H    −4.72276    −0.42172    +4.99314
 H    −6.15424    −1.38740    +4.75474
 H    −4.71983    −2.49043    +6.40511
 H    −4.75649    −3.47749    +4.95176
 H    −3.35431    −2.48000    +5.30676
24
 * E =   +1.542  kcal/mol  ;  (111) 178_068_284_183_179_180
 H    +0.00000    +0.00000    +0.00000
 O    +0.00000    +0.00000    +0.95358
 C    −0.89867    +0.00000    +1.27250
 C    −0.90145    −0.03990    +2.78051
 H    −1.42618    +0.89570    +0.93074
 H    −1.43657    −0.87020    +0.88848
 C    −0.37598    +1.21055    +3.46946
 H    −1.93364    −0.20862    +3.08926
 H    −0.33261    −0.91229    +3.10506
 C    +1.13357    +1.37306    +3.45814
 H    −0.84015    +2.08983    +3.01557
 H    −0.71331    +1.19869    +4.50761
 C    +1.59296    +2.60087    +4.21980
 H    +1.58860    +0.48332    +3.89939
 H    +1.49100    +1.41480    +2.43155
 C    +3.09979    +2.77128    +4.23468
 H    +1.13646    +3.49165    +3.78101
```

```
 H    +1.22722    +2.55193    +5.24868
 C    +3.54920    +4.00189    +4.99622
 H    +3.55614    +1.88231    +4.67296
 H    +3.46408    +2.82179    +3.20760
 H    +4.63220    +4.10289    +4.99379
 H    +3.13150    +4.90712    +4.55826
 H    +3.22310    +3.96057    +6.03434
24
 * E =   +1.543  kcal/mol  ;  (112) 068_296_178_063_170_063
 H    +0.00000    +0.00000    +0.00000
 O    +0.00000    +0.00000    +0.95375
 C    -0.90003    +0.00000    +1.26931
 C    -1.64179    -1.28422    +0.96679
 H    -0.83139    +0.14572    +2.34382
 H    -1.44990    +0.85319    +0.86046
 C    -1.82710    -1.53849    -0.51781
 H    -1.09806    -2.11642    +1.41624
 H    -2.61310    -1.23406    +1.45975
 C    -2.52221    -2.85097    -0.84158
 H    -2.39320    -0.71278    -0.95715
 H    -0.85118    -1.54199    -1.00700
 C    -3.94737    -2.93964    -0.32397
 H    -2.53428    -2.98622    -1.92487
 H    -1.92818    -3.67248    -0.43890
 C    -4.71484    -4.14418    -0.84404
 H    -3.94569    -2.96721    +0.76697
 H    -4.48085    -2.02938    -0.60570
 C    -4.12217    -5.47558    -0.42394
 H    -5.74463    -4.08405    -0.49223
 H    -4.76275    -4.09465    -1.93331
 H    -4.74273    -6.30497    -0.75516
 H    -3.12918    -5.62520    -0.84190
 H    -4.03641    -5.53917    +0.66014
24
 * E =   +1.553  kcal/mol  ;  (113) 062_068_290_185_180_180
 H    +0.00000    +0.00000    +0.00000
 O    +0.00000    +0.00000    +0.95447
 C    -0.90199    +0.00000    +1.26659
 C    -1.70554    -1.21474    +0.85153
 H    -0.83260    +0.04514    +2.35168
 H    -1.41558    +0.90744    +0.93858
 C    -1.27584    -2.53309    +1.47442
 H    -2.74483    -1.01862    +1.11993
 H    -1.69040    -1.29883    -0.23900
 C    +0.05314    -3.08300    +0.98810
```

```
 H   -1.24289   -2.41723   +2.56027
 H   -2.05157   -3.27529   +1.27672
 C   +0.36304   -4.45172   +1.56225
 H   +0.03411   -3.14821   -0.10326
 H   +0.85237   -2.38760   +1.23698
 C   +1.68344   -5.02171   +1.08135
 H   +0.37507   -4.39127   +2.65315
 H   -0.44213   -5.14571   +1.30828
 C   +1.98453   -6.38836   +1.66255
 H   +1.67199   -5.08178   -0.00823
 H   +2.48644   -4.32908   +1.33727
 H   +2.93480   -6.77695   +1.30353
 H   +2.03280   -6.34890   +2.74958
 H   +1.21099   -7.10661   +1.39532
24
 * E =  +1.570  kcal/mol ; (114) 177_174_062_173_175_063
 H   +0.00000   +0.00000   +0.00000
 O   +0.00000   +0.00000   +0.95349
 C   -0.89785   +0.00000   +1.27445
 C   -0.85013   -0.06433   +2.77981
 H   -1.42117   +0.90959   +0.96314
 H   -1.44801   -0.85369   +0.87272
 C   -2.20995   +0.07825   +3.44002
 H   -0.38625   -1.00640   +3.07318
 H   -0.18727   +0.72902   +3.12275
 C   -3.20048   -1.01902   +3.09160
 H   -2.07035   +0.09280   +4.52163
 H   -2.64182   +1.04771   +3.18111
 C   -4.48409   -0.91988   +3.89507
 H   -3.44718   -0.97929   +2.02849
 H   -2.72774   -1.98833   +3.25932
 C   -5.55367   -1.92285   +3.49622
 H   -4.25227   -1.04287   +4.95566
 H   -4.88672   +0.08903   +3.78901
 C   -5.15296   -3.36922   +3.71508
 H   -6.46016   -1.71385   +4.06415
 H   -5.81267   -1.77161   +2.44676
 H   -5.97458   -4.04428   +3.48696
 H   -4.31293   -3.65589   +3.08642
 H   -4.86266   -3.54039   +4.75094
24
 * E =  +1.573  kcal/mol ; (115) 066_302_304_304_305_302
 H   +0.00000   +0.00000   +0.00000
 O   +0.00000   +0.00000   +0.95458
 C   -0.90241   +0.00000   +1.26583
```

C −1.64964 −1.27003 +0.91657
H −0.84179 +0.11029 +2.34501
H −1.43864 +0.87357 +0.88435
C −1.70238 −1.56231 −0.57574
H −1.17500 −2.10734 +1.42656
H −2.66176 −1.19009 +1.31743
C −2.28341 −0.44094 −1.42571
H −0.69819 −1.79777 −0.93219
H −2.28026 −2.47360 −0.73253
C −3.68073 +0.00224 −1.01619
H −1.62445 +0.42994 −1.39700
H −2.29200 −0.76165 −2.46747
C −4.71158 −1.11495 −0.96744
H −3.63711 +0.48571 −0.03898
H −4.02090 +0.76873 −1.71422
C −4.87631 −1.84979 −2.28348
H −4.44628 −1.82712 −0.18443
H −5.66945 −0.68930 −0.66945
H −5.67777 −2.58260 −2.22828
H −5.11473 −1.15819 −3.09051
H −3.96873 −2.38101 −2.56445
24
* E = +1.577 kcal/mol ; (116) 059_182_296_185_177_064
H +0.00000 +0.00000 +0.00000
O +0.00000 +0.00000 +0.95450
C −0.90226 +0.00000 +1.26596
C −1.69034 −1.19288 +0.77093
H −0.83144 −0.00699 +2.34987
H −1.41213 +0.92588 +0.98092
C −3.14365 −1.19440 +1.21425
H −1.65199 −1.20306 −0.32083
H −1.19310 −2.10330 +1.10687
C −3.34057 −1.30605 +2.71588
H −3.63556 −0.28813 +0.85334
H −3.65613 −2.02641 +0.72993
C −4.80190 −1.43525 +3.10387
H −2.79079 −2.17473 +3.08711
H −2.90768 −0.43681 +3.21037
C −5.04056 −1.63204 +4.59189
H −5.34229 −0.54702 +2.76774
H −5.23537 −2.27659 +2.56046
C −4.60361 −0.45823 +5.44736
H −6.10252 −1.81537 +4.75472
H −4.52366 −2.53517 +4.92086
H −4.86459 −0.61411 +6.49154

```
H    −3.52828    −0.30156    +5.40233
H    −5.08528    +0.46255    +5.12059
24
 * E =   +1.596  kcal/mol ;  (117) 065_302_301_182_066_176
H    +0.00000    +0.00000    +0.00000
O    +0.00000    +0.00000    +0.95451
C    −0.90175    +0.00000    +1.26745
C    −1.65104    −1.26836    +0.91486
H    −0.83784    +0.10106    +2.34739
H    −1.43607    +0.87820    +0.89652
C    −1.71566    −1.56872    −0.57488
H    −1.17562    −2.10450    +1.42628
H    −2.66386    −1.18590    +1.31383
C    −2.37575    −0.48164    −1.40525
H    −0.70787    −1.75430    −0.95549
H    −2.25090    −2.50746    −0.70898
C    −2.47306    −0.80510    −2.88752
H    −3.37622    −0.28601    −1.01176
H    −1.82470    +0.45374    −1.29185
C    −3.38132    −1.97481    −3.22065
H    −2.83477    +0.07722    −3.41795
H    −1.47332    −1.00401    −3.28133
C    −3.52464    −2.19057    −4.71395
H    −2.99984    −2.88656    −2.76194
H    −4.36429    −1.79945    −2.77950
H    −4.17651    −3.03203    −4.93646
H    −3.94272    −1.30989    −5.19872
H    −2.55843    −2.39015    −5.17447
24
 * E =   +1.606  kcal/mol ;  (118) 061_173_059_053_057_176
H    +0.00000    +0.00000    +0.00000
O    +0.00000    +0.00000    +0.95443
C    −0.90202    +0.00000    +1.26634
C    −1.67273    −1.22275    +0.82137
H    −0.83699    +0.02329    +2.35230
H    −1.41410    +0.91409    +0.95451
C    −3.05357    −1.33579    +1.44753
H    −1.76020    −1.21462    −0.26857
H    −1.08323    −2.10270    +1.07570
C    −3.98240    −0.15873    +1.17531
H    −3.52059    −2.25539    +1.09587
H    −2.94519    −1.45250    +2.52676
C    −4.15296    +0.19235    −0.29400
H    −4.96085    −0.38714    +1.60050
H    −3.62559    +0.72211    +1.70994
```

```
 C    −4.66748    −0.94929    −1.15063
 H    −4.84163    +1.03478    −0.37509
 H    −3.20437    +0.54737    −0.70462
 C    −4.92732    −0.53242    −2.58417
 H    −3.95025    −1.77106    −1.13812
 H    −5.58534    −1.34245    −0.70947
 H    −5.28805    −1.36390    −3.18505
 H    −5.67249    +0.25983    −2.63264
 H    −4.01919    −0.15584    −3.05247
24
 * E =  +1.607  kcal/mol ;  (119)  177_174_063_174_183_296
 H    +0.00000    +0.00000    +0.00000
 O    +0.00000    +0.00000    +0.95348
 C    −0.89758    +0.00000    +1.27514
 C    −0.84737    −0.06784    +2.78031
 H    −1.42056    +0.91064    +0.96652
 H    −1.44830    −0.85243    +0.87162
 C    −2.20562    +0.06735    +3.44563
 H    −0.37849    −1.00846    +3.07042
 H    −0.18703    +0.72757    +3.12337
 C    −3.18691    −1.04233    +3.11087
 H    −2.06076    +0.09302    +4.52627
 H    −2.64790    +1.03038    +3.18015
 C    −4.47527    −0.94212    +3.90634
 H    −3.41459    −1.02415    +2.04501
 H    −2.71667    −2.00959    +3.30665
 C    −5.45401    −2.07903    +3.66280
 H    −4.22842    −0.90786    +4.96881
 H    −4.96443    +0.00811    +3.67879
 C    −5.98135    −2.14567    +2.24218
 H    −4.97398    −3.02537    +3.91794
 H    −6.29377    −1.97152    +4.34922
 H    −6.73313    −2.92458    +2.13766
 H    −6.44159    −1.20161    +1.95296
 H    −5.19107    −2.36001    +1.52625
24
 * E =  +1.609  kcal/mol ;  (120)  181_179_174_064_179_295
 H    +0.00000    +0.00000    +0.00000
 O    +0.00000    +0.00000    +0.95349
 C    −0.89773    +0.00000    +1.27477
 C    −0.86019    +0.01328    +2.78049
 H    −1.43958    +0.87813    +0.91187
 H    −1.43422    −0.88711    +0.92626
 C    −2.24905    +0.03744    +3.39065
 H    −0.31648    −0.86867    +3.12125
```

H −0.28123 +0.87985 +3.09533
C −2.26636 −0.08945 +4.90552
H −2.75685 +0.96098 +3.10328
H −2.83805 −0.77707 +2.96517
C −1.59240 +1.06023 +5.63241
H −3.30284 −0.16798 +5.23242
H −1.78371 −1.02652 +5.19351
C −1.63537 +0.94524 +7.14800
H −0.55100 +1.12636 +5.31875
H −2.06276 +1.99957 +5.32992
C −3.03151 +1.01662 +7.73672
H −1.15969 +0.01023 +7.44918
H −1.02904 +1.74333 +7.57604
H −3.00013 +1.00599 +8.82383
H −3.53665 +1.93175 +7.43007
H −3.64932 +0.17842 +7.42284
24
* E = +1.615 kcal/mol ; (121) 062_173_063_169_063_176
H +0.00000 +0.00000 +0.00000
O +0.00000 +0.00000 +0.95444
C −0.90225 +0.00000 +1.26572
C −1.66934 −1.22772 +0.82609
H −0.83769 +0.02652 +2.35166
H −1.41447 +0.91172 +0.95030
C −3.05545 −1.33579 +1.43956
H −1.75570 −1.22623 −0.26401
H −1.07703 −2.10266 +1.09132
C −4.00034 −0.21326 +1.04200
H −3.49727 −2.28787 +1.14080
H −2.95992 −1.37543 +2.52559
C −5.44631 −0.45806 +1.43900
H −3.66814 +0.72855 +1.48217
H −3.94785 −0.07793 −0.04028
C −5.67531 −0.55892 +2.93602
H −6.06486 +0.34930 +1.04341
H −5.80124 −1.37373 +0.95988
C −7.14174 −0.69860 +3.29209
H −5.12495 −1.40940 +3.33823
H −5.26269 +0.32789 +3.42101
H −7.28819 −0.77363 +4.36704
H −7.71389 +0.15755 +2.93838
H −7.57106 −1.58969 +2.83691
24
* E = +1.626 kcal/mol ; (122) 061_177_174_064_179_295
H +0.00000 +0.00000 +0.00000

```
O    +0.00000    +0.00000    +0.95437
C    −0.90207    +0.00000    +1.26596
C    −1.68183    −1.21819    +0.82497
H    −0.83691    +0.02740    +2.35132
H    −1.42080    +0.91062    +0.95201
C    −3.08866    −1.23855    +1.39403
H    −1.73391    −1.23635    −0.26744
H    −1.12615    −2.10458    +1.12672
C    −3.96244    −2.36544    +0.86710
H    −3.03474    −1.30548    +2.48277
H    −3.57361    −0.28600    +1.17330
C    −3.46672    −3.75478    +1.22536
H    −4.97123    −2.22842    +1.25532
H    −4.04076    −2.28271    −0.21982
C    −4.35679    −4.87962    +0.72067
H    −2.46453    −3.89738    +0.82176
H    −3.37164    −3.83149    +2.31158
C    −5.73931    −4.90459    +1.34427
H    −4.44716    −4.80488    −0.36449
H    −3.86232    −5.83076    +0.91705
H    −6.30645    −5.76900    +1.00667
H    −5.67520    −4.95569    +2.43044
H    −6.31734    −4.01924    +1.08972
24
* E =  +1.648  kcal/mol ; (123) 062_173_064_174_175_063
H    +0.00000    +0.00000    +0.00000
O    +0.00000    +0.00000    +0.95443
C    −0.90221    +0.00000    +1.26581
C    −1.66870    −1.22797    +0.82571
H    −0.83740    +0.02592    +2.35174
H    −1.41405    +0.91202    +0.95087
C    −3.05399    −1.34179    +1.43935
H    −1.75505    −1.22666    −0.26431
H    −1.07457    −2.10199    +1.09001
C    −4.01585    −0.23544    +1.04228
H    −3.48583    −2.30072    +1.15014
H    −2.96557    −1.36966    +2.52763
C    −5.42197    −0.47532    +1.56044
H    −3.66131    +0.72598    +1.41869
H    −4.02848    −0.15197    −0.04603
C    −6.39428    +0.65946    +1.28480
H    −5.81163    −1.39794    +1.12370
H    −5.37374    −0.64752    +2.63705
C    −6.63999    +0.91466    −0.18998
H    −7.34340    +0.43381    +1.77069
```

```
H    -6.02291   +1.57146   +1.75538
H    -7.39717   +1.68153   -0.33594
H    -5.73832   +1.24820   -0.69875
H    -6.98525   +0.01031   -0.68958
24
 * E =   +1.648  kcal/mol ; (124) 066_303_303_189_303_303
H    +0.00000   +0.00000   +0.00000
O    +0.00000   +0.00000   +0.95495
C    -0.90246   +0.00000   +1.26721
C    -1.64605   -1.27606   +0.92953
H    -0.84080   +0.11808   +2.34553
H    -1.44119   +0.86871   +0.88041
C    -1.68458   -1.59297   -0.55710
H    -1.17922   -2.10420   +1.46134
H    -2.66483   -1.18499   +1.31107
C    -2.29458   -0.49487   -1.41050
H    -0.67246   -1.81498   -0.90205
H    -2.24862   -2.51492   -0.70630
C    -2.52041   -0.89989   -2.85888
H    -3.24935   -0.19333   -0.97604
H    -1.66586   +0.39793   -1.38044
C    -1.27421   -1.37339   -3.59236
H    -3.26989   -1.69224   -2.89124
H    -2.94800   -0.05312   -3.39812
C    -0.14543   -0.36135   -3.59631
H    -0.92202   -2.30850   -3.15612
H    -1.54741   -1.60952   -4.62045
H    +0.68788   -0.70006   -4.20690
H    -0.47750   +0.59889   -3.98851
H    +0.24343   -0.18648   -2.59364
24
 * E =   +1.650  kcal/mol ; (125) 065_302_305_301_189_297
H    +0.00000   +0.00000   +0.00000
O    +0.00000   +0.00000   +0.95463
C    -0.90225   +0.00000   +1.26650
C    -1.65466   -1.26288   +0.90420
H    -0.84039   +0.09864   +2.34673
H    -1.43553   +0.87994   +0.89493
C    -1.70831   -1.53990   -0.59172
H    -1.18233   -2.10766   +1.40398
H    -2.66527   -1.18475   +1.30726
C    -2.26509   -0.40283   -1.43589
H    -0.70750   -1.79479   -0.94459
H    -2.31103   -2.43322   -0.75873
C    -3.67668   +0.00670   -1.05509
```

H −1.60451 +0.46519 −1.37496
H −2.25457 −0.70771 −2.48390
C −4.32123 +0.98125 −2.02692
H −4.29434 −0.89102 −0.98634
H −3.67566 +0.45053 −0.05720
C −3.60656 +2.31576 −2.12103
H −4.36753 +0.52227 −3.01601
H −5.35398 +1.14968 −1.72240
H −4.14449 +3.00564 −2.76697
H −3.51964 +2.78169 −1.14017
H −2.60246 +2.20932 −2.52562
24
* E = +1.652 kcal/mol ; (126) 061_178_185_296_181_065
H +0.00000 +0.00000 +0.00000
O +0.00000 +0.00000 +0.95436
C −0.90197 +0.00000 +1.26620
C −1.68398 −1.21465 +0.81983
H −0.83613 +0.02309 +2.35154
H −1.41949 +0.91268 +0.95604
C −3.10142 −1.21801 +1.36229
H −1.69928 −1.24202 −0.27151
H −1.15430 −2.10991 +1.14791
C −3.89072 −2.47896 +1.04897
H −3.06210 −1.08932 +2.44519
H −3.64125 −0.35135 +0.97409
C −4.13834 −2.70611 −0.43141
H −3.36575 −3.34410 +1.46093
H −4.84506 −2.42510 +1.57188
C −4.95372 −3.95221 −0.73936
H −4.64681 −1.83230 −0.84745
H −3.18353 −2.77985 −0.95182
C −6.37717 −3.90717 −0.21728
H −4.97683 −4.09637 −1.81942
H −4.44193 −4.82460 −0.32930
H −6.93745 −4.78538 −0.52972
H −6.41187 −3.87122 +0.86908
H −6.90204 −3.02990 −0.59341
24
* E = +1.661 kcal/mol ; (127) 066_296_188_296_181_065
H +0.00000 +0.00000 +0.00000
O +0.00000 +0.00000 +0.95403
C −0.90090 +0.00000 +1.26796
C −1.65195 −1.26695 +0.92042
H −0.83392 +0.11277 +2.34655
H −1.44089 +0.87032 +0.88223

```
C   −1.81966   −1.46901   −0.57507
H   −1.12556   −2.11137   +1.36302
H   −2.63663   −1.22092   +1.39078
C   −2.73152   −2.62494   −0.95462
H   −2.21839   −0.54981   −1.01085
H   −0.84227   −1.63325   −1.03536
C   −2.22017   −3.98622   −0.51868
H   −3.72092   −2.45831   −0.52157
H   −2.86733   −2.61675   −2.03568
C   −3.11952   −5.14396   −0.92239
H   −1.22215   −4.14379   −0.93561
H   −2.10206   −3.99926   +0.56452
C   −3.23262   −5.34933   −2.42101
H   −2.73711   −6.05747   −0.46747
H   −4.11364   −4.98939   −0.49919
H   −3.82164   −6.23381   −2.65238
H   −3.71007   −4.50529   −2.91337
H   −2.24936   −5.48088   −2.87096
24
 * E =  +1.676 kcal/mol ; (128) 062_052_053_058_178_295
H   +0.00000   +0.00000   +0.00000
O   +0.00000   +0.00000   +0.95457
C   −0.90154   +0.00000   +1.26830
C   −1.67377   −1.23319   +0.85117
H   −0.82842   +0.05051   +2.35241
H   −1.42657   +0.90060   +0.94121
C   −0.97526   −2.53818   +1.20403
H   −2.66090   −1.18678   +1.31308
H   −1.84892   −1.20287   −0.22659
C   −0.55872   −2.66676   +2.66052
H   −1.63273   −3.36770   +0.93957
H   −0.08578   −2.63877   +0.58377
C   −1.70408   −2.54155   +3.64855
H   −0.07117   −3.63278   +2.78733
H   +0.20165   −1.91628   +2.88556
C   −1.29233   −2.71595   +5.10199
H   −2.17374   −1.56289   +3.53750
H   −2.47548   −3.27616   +3.40213
C   −0.78519   −4.10507   +5.43946
H   −0.52559   −1.97879   +5.34653
H   −2.14669   −2.48626   +5.73871
H   −0.57436   −4.19832   +6.50236
H   −1.52448   −4.86248   +5.18176
H   +0.13151   −4.34397   +4.90556
24
```

```
 * E =  +1.682  kcal/mol ; (129) 062_173_065_175_184_296
 H   +0.00000   +0.00000   +0.00000
 O   +0.00000   +0.00000   +0.95442
 C   -0.90229   +0.00000   +1.26553
 C   -1.66617   -1.23111   +0.82924
 H   -0.83810   +0.03009   +2.35136
 H   -1.41499   +0.91022   +0.94691
 C   -3.05189   -1.34765   +1.44170
 H   -1.75104   -1.23438   -0.26086
 H   -1.06978   -2.10237   +1.09757
 C   -4.02068   -0.25303   +1.02955
 H   -3.47565   -2.31352   +1.16413
 H   -2.96494   -1.36080   +2.53043
 C   -5.42199   -0.48348   +1.56436
 H   -3.65390   +0.71399   +1.37279
 H   -4.05724   -0.19700   -0.06176
 C   -6.44503   +0.54289   +1.10637
 H   -5.75434   -1.47701   +1.25865
 H   -5.39108   -0.49747   +2.65642
 C   -6.16559   +1.95209   +1.59289
 H   -6.49501   +0.53672   +0.01612
 H   -7.43039   +0.23473   +1.45558
 H   -6.96276   +2.63315   +1.30409
 H   -6.08443   +1.98037   +2.67879
 H   -5.23841   +2.34635   +1.18310
24
 * E =  +1.687  kcal/mol ; (130) 064_061_179_067_180_296
 H   +0.00000   +0.00000   +0.00000
 O   +0.00000   +0.00000   +0.95453
 C   -0.90213   +0.00000   +1.26643
 C   -1.66049   -1.25134   +0.88076
 H   -0.83291   +0.07576   +2.34961
 H   -1.43212   +0.88721   +0.91157
 C   -1.04214   -2.51543   +1.44707
 H   -2.69016   -1.15301   +1.23145
 H   -1.71583   -1.30658   -0.20862
 C   -1.79768   -3.79046   +1.10932
 H   -0.01179   -2.59470   +1.09978
 H   -0.98560   -2.41268   +2.53200
 C   -1.78288   -4.15112   -0.36570
 H   -1.36404   -4.60868   +1.68346
 H   -2.83408   -3.69979   +1.44520
 C   -2.53631   -5.42893   -0.70023
 H   -2.21373   -3.33465   -0.94534
 H   -0.74618   -4.24756   -0.69827
```

```
C    −1.93752   −6.68064   −0.08749
H    −3.57433   −5.32637   −0.37880
H    −2.56606   −5.54405   −1.78371
H    −2.46516   −7.57155   −0.42031
H    −0.89168   −6.79085   −0.37140
H    −1.98370   −6.66404   +0.99888
24
 * E =   +1.688  kcal/mol ; (131) 068_296_178_065_179_295
H    +0.00000   +0.00000   +0.00000
O    +0.00000   +0.00000   +0.95376
C    −0.90008   +0.00000   +1.26922
C    −1.64250   −1.28383   +0.96612
H    −0.83150   +0.14528   +2.34379
H    −1.44964   +0.85347   +0.86058
C    −1.82366   −1.53839   −0.51901
H    −1.10012   −2.11621   +1.41668
H    −2.61368   −1.23153   +1.45868
C    −2.52854   −2.84413   −0.85191
H    −2.37736   −0.70710   −0.96364
H    −0.84433   −1.55178   −1.00095
C    −3.97331   −2.90619   −0.38984
H    −2.48725   −2.99276   −1.93046
H    −1.97359   −3.67419   −0.40837
C    −4.67843   −4.20815   −0.73648
H    −4.01671   −2.76608   +0.69000
H    −4.52520   −2.07092   −0.82877
C    −4.83921   −4.44964   −2.22531
H    −4.13267   −5.04042   −0.28864
H    −5.66300   −4.20689   −0.26915
H    −5.41679   −5.35115   −2.41620
H    −5.35841   −3.61832   −2.70063
H    −3.88043   −4.56832   −2.72466
24
 * E =   +1.708  kcal/mol ; (132) 062_057_066_182_302_304
H    +0.00000   +0.00000   +0.00000
O    +0.00000   +0.00000   +0.95461
C    −0.90188   +0.00000   +1.26749
C    −1.67017   −1.23704   +0.85182
H    −0.83169   +0.06527   +2.34991
H    −1.42892   +0.89465   +0.92725
C    −1.03236   −2.54544   +1.28871
H    −2.68260   −1.16283   +1.25425
H    −1.77718   −1.23219   −0.23548
C    −1.01503   −2.74937   +2.79221
H    −1.57424   −3.36575   +0.81722
```

```
H   -0.01093   -2.58859   +0.90836
C   -0.33121   -4.03621   +3.22954
H   -0.49994   -1.91575   +3.26859
H   -2.03953   -2.72712   +3.17014
C   -0.91720   -5.31262   +2.64450
H   +0.72609   -3.98006   +2.96646
H   -0.37082   -4.09851   +4.31841
C   -2.39794   -5.48574   +2.92050
H   -0.74248   -5.34104   +1.56862
H   -0.37189   -6.16219   +3.05529
H   -2.75627   -6.44932   +2.56571
H   -2.60722   -5.42647   +3.98795
H   -2.99047   -4.71661   +2.42791
24
 * E =   +1.713  kcal/mol  ;  (133)  059_182_295_181_065_176
H   +0.00000   +0.00000   +0.00000
O   +0.00000   +0.00000   +0.95451
C   -0.90217   +0.00000   +1.26623
C   -1.69067   -1.19222   +0.76979
H   -0.83057   -0.00690   +2.34997
H   -1.41192   +0.92597   +0.98137
C   -3.14539   -1.19662   +1.21057
H   -1.65113   -1.19979   -0.32196
H   -1.19338   -2.10347   +1.10352
C   -3.33843   -1.33789   +2.71094
H   -3.63148   -0.27814   +0.87195
H   -3.65524   -2.01282   +0.70091
C   -4.79403   -1.37274   +3.14765
H   -2.83759   -2.24744   +3.05125
H   -2.84485   -0.51149   +3.22194
C   -5.57265   -2.58215   +2.66265
H   -4.83454   -1.34866   +4.23786
H   -5.29515   -0.46306   +2.80819
C   -6.98473   -2.62105   +3.21194
H   -5.61205   -2.58951   +1.57367
H   -5.04048   -3.48941   +2.95467
H   -7.53041   -3.49115   +2.85447
H   -6.98071   -2.65609   +4.30013
H   -7.54387   -1.73511   +2.91522
24
 * E =   +1.724  kcal/mol  ;  (134)  177_175_058_055_055_058
H   +0.00000   +0.00000   +0.00000
O   +0.00000   +0.00000   +0.95351
C   -0.89809   +0.00000   +1.27384
C   -0.85406   -0.07176   +2.77837
```

H	−1.42153	+0.90979	+0.96367
H	−1.44894	−0.85314	+0.86944
C	−2.22078	+0.05342	+3.43104
H	−0.37451	−1.00770	+3.06559
H	−0.20393	+0.72722	+3.13216
C	−3.23628	−0.99658	+3.00041
H	−2.09207	+0.00949	+4.51312
H	−2.62989	+1.04298	+3.22137
C	−2.76960	−2.43445	+3.17496
H	−4.15684	−0.84091	+3.56344
H	−3.50615	−0.84132	+1.95496
C	−2.31809	−2.78930	+4.58331
H	−3.58279	−3.10169	+2.88433
H	−1.95237	−2.63867	+2.48091
C	−3.38483	−2.57164	+5.63875
H	−2.00633	−3.83346	+4.59511
H	−1.42962	−2.21074	+4.84068
H	−3.04930	−2.91339	+6.61507
H	−3.64708	−1.51954	+5.73485
H	−4.29607	−3.11541	+5.39253

24

∗ E = +1.736 kcal/mol ; (135) 059_182_301_306_305_302

H	+0.00000	+0.00000	+0.00000
O	+0.00000	+0.00000	+0.95445
C	−0.90192	+0.00000	+1.26673
C	−1.68790	−1.19907	+0.78554
H	−0.83043	+0.00053	+2.35148
H	−1.41435	+0.92304	+0.97727
C	−3.13852	−1.19673	+1.24171
H	−1.66163	−1.22021	−0.30625
H	−1.18009	−2.10358	+1.12100
C	−3.33102	−1.16978	+2.75212
H	−3.65014	−0.33613	+0.80788
H	−3.63439	−2.07607	+0.82905
C	−2.60770	−2.27989	+3.50058
H	−3.00181	−0.20871	+3.14849
H	−4.39888	−1.22108	+2.96540
C	−2.93803	−3.68642	+3.02552
H	−1.52963	−2.12651	+3.42825
H	−2.85007	−2.19785	+4.56131
C	−4.41330	−4.02720	+3.10786
H	−2.59199	−3.81882	+1.99917
H	−2.36939	−4.39735	+3.62442
H	−4.59448	−5.06445	+2.83609
H	−4.79360	−3.87804	+4.11764

```
 H    -5.00777    -3.40706    +2.43923
24
 * E =   +1.745  kcal/mol  ;  (136) 177_173_064_178_294_184
 H    +0.00000    +0.00000    +0.00000
 O    +0.00000    +0.00000    +0.95351
 C    -0.89793    +0.00000    +1.27430
 C    -0.84928    -0.07289    +2.77952
 H    -1.41904    +0.91278    +0.96876
 H    -1.44901    -0.84975    +0.86607
 C    -2.20503    +0.07586    +3.44845
 H    -0.38930    -1.01846    +3.06782
 H    -0.18054    +0.71512    +3.12338
 C    -3.19154    -1.03140    +3.11802
 H    -2.04904    +0.11271    +4.52559
 H    -2.64370    +1.03852    +3.17413
 C    -4.52671    -0.90717    +3.83421
 H    -3.37688    -1.04562    +2.04382
 H    -2.74031    -1.99675    +3.36057
 C    -4.45469    -1.06980    +5.34166
 H    -4.97421    +0.06142    +3.59850
 H    -5.21121    -1.65875    +3.43719
 C    -5.82186    -1.03403    +5.99486
 H    -3.96138    -2.01519    +5.57496
 H    -3.83144    -0.28692    +5.77285
 H    -5.75408    -1.15070    +7.07396
 H    -6.32553    -0.08952    +5.79537
 H    -6.45868    -1.83109    +5.61448
24
 * E =   +1.793  kcal/mol  ;  (137) 064_302_306_301_182_065
 H    +0.00000    +0.00000    +0.00000
 O    +0.00000    +0.00000    +0.95468
 C    -0.90245    +0.00000    +1.26613
 C    -1.65830    -1.25716    +0.89166
 H    -0.84106    +0.08918    +2.34720
 H    -1.43273    +0.88488    +0.90199
 C    -1.70613    -1.52218    -0.60688
 H    -1.18999    -2.10782    +1.38524
 H    -2.66904    -1.18014    +1.29400
 C    -2.23246    -0.37305    -1.45524
 H    -0.70679    -1.79247    -0.95240
 H    -2.32179    -2.40435    -0.78520
 C    -3.64118    +0.06443    -1.09822
 H    -1.56721    +0.49239    -1.37845
 H    -2.19686    -0.67863    -2.50028
 C    -4.18696    +1.17917    -1.97643
```

H −4.30819 −0.79961 −1.15501
H −3.66429 +0.39666 −0.05977
C −4.37460 +0.78814 −3.42989
H −5.14412 +1.50614 −1.57082
H −3.52203 +2.04246 −1.91338
H −4.83577 +1.59391 −3.99619
H −3.42968 +0.55277 −3.91411
H −5.01743 −0.08677 −3.51764
24
* E = +1.794 kcal/mol ; (138) 059_182_297_191_303_303
H +0.00000 +0.00000 +0.00000
O +0.00000 +0.00000 +0.95451
C −0.90203 +0.00000 +1.26665
C −1.69153 −1.19311 +0.77470
H −0.83112 −0.00654 +2.35067
H −1.41253 +0.92567 +0.98143
C −3.14271 −1.18850 +1.22502
H −1.65604 −1.20646 −0.31709
H −1.19510 −2.10314 +1.11302
C −3.32074 −1.26965 +2.73160
H −3.63418 −0.29090 +0.84339
H −3.65759 −2.03223 +0.76262
C −4.75681 −1.52614 +3.16117
H −2.68963 −2.07067 +3.12071
H −2.96081 −0.35256 +3.19911
C −5.76449 −0.49940 +2.66637
H −5.05947 −2.51432 +2.81050
H −4.79640 −1.56363 +4.25101
C −5.44199 +0.92188 +3.08445
H −5.83852 −0.55067 +1.57950
H −6.74966 −0.77189 +3.04433
H −6.22959 +1.60945 +2.78530
H −5.32949 +0.99674 +4.16530
H −4.51528 +1.27339 +2.63394
24
* E = +1.809 kcal/mol ; (139) 177_174_062_169_057_057
H +0.00000 +0.00000 +0.00000
O +0.00000 +0.00000 +0.95351
C −0.89800 +0.00000 +1.27411
C −0.85211 −0.07239 +2.77914
H −1.41991 +0.91194 +0.96705
H −1.44945 −0.85095 +0.86824
C −2.21581 +0.05646 +3.43484
H −0.38391 −1.01396 +3.06742
H −0.19493 +0.72284 +3.12853

```
C   −3.18966   −1.05031   +3.06759
H   −2.08291   +0.06367   +4.51794
H   −2.64725   +1.02680   +3.17957
C   −4.44671   −1.06462   +3.92335
H   −3.46817   −0.96782   +2.01617
H   −2.68596   −2.01357   +3.16799
C   −5.24473   +0.23055   +3.90778
H   −5.09051   −1.88060   +3.59064
H   −4.16966   −1.29500   +4.95349
C   −5.68026   +0.66176   +2.52099
H   −6.12425   +0.10275   +4.53852
H   −4.66088   +1.02764   +4.36924
H   −6.31884   +1.54098   +2.56344
H   −4.82756   +0.90961   +1.89107
H   −6.23806   −0.12938   +2.02134
24
* E =  +1.826  kcal/mol ; (140) 062_173_066_179_295_184
H   +0.00000   +0.00000   +0.00000
O   +0.00000   +0.00000   +0.95443
C   −0.90216   +0.00000   +1.26596
C   −1.66622   −1.23101   +0.82924
H   −0.83715   +0.02802   +2.35175
H   −1.41379   +0.91133   +0.94935
C   −3.05395   −1.34983   +1.43841
H   −1.74853   −1.23612   −0.26111
H   −1.06946   −2.10133   +1.09963
C   −4.02860   −0.27273   +0.99249
H   −3.45597   −2.32931   +1.18362
H   −2.97253   −1.33142   +2.52796
C   −5.42101   −0.41265   +1.58620
H   −3.63689   +0.70854   +1.25949
H   −4.09963   −0.28500   −0.09813
C   −6.17429   −1.65609   +1.14974
H   −5.35160   −0.39888   +2.67649
H   −6.01018   +0.46400   +1.31194
C   −7.59130   −1.69367   +1.68622
H   −6.19396   −1.69679   +0.05900
H   −5.64358   −2.54930   +1.47820
H   −8.11730   −2.59046   +1.36730
H   −7.59733   −1.67629   +2.77487
H   −8.16333   −0.83354   +1.34213
24
* E =  +1.830  kcal/mol ; (141) 179_075_296_305_304_185
H   +0.00000   +0.00000   +0.00000
O   +0.00000   +0.00000   +0.95355
```

```
C   −0.89786   +0.00000   +1.27464
C   −0.88592   −0.03135   +2.78373
H   −1.43113   +0.89061   +0.92799
H   −1.43143   −0.87560   +0.89764
C   −0.50886   +1.27643   +3.46269
H   −1.88623   −0.31365   +3.11329
H   −0.22016   −0.83590   +3.09911
C   +0.90759   +1.77776   +3.21625
H   −1.21405   +2.04830   +3.14826
H   −0.66361   +1.15624   +4.53566
C   +2.00447   +0.78294   +3.55760
H   +1.01427   +2.06488   +2.17211
H   +1.05057   +2.68459   +3.80767
C   +1.96581   +0.27207   +4.98590
H   +1.95462   −0.05714   +2.86430
H   +2.97070   +1.25907   +3.38062
C   +3.15074   −0.61195   +5.31999
H   +1.93414   +1.11964   +5.67402
H   +1.04372   −0.28681   +5.15381
H   +3.10395   −0.98057   +6.34237
H   +3.19117   −1.47515   +4.65750
H   +4.08766   −0.06907   +5.20547
24
* E =  +1.838  kcal/mol ; (142) 178_175_057_057_171_063
H   +0.00000   +0.00000   +0.00000
O   +0.00000   +0.00000   +0.95350
C   −0.89795   +0.00000   +1.27419
C   −0.85259   −0.06207   +2.77900
H   −1.42395   +0.90624   +0.95803
H   −1.44670   −0.85728   +0.87536
C   −2.21916   +0.06475   +3.43266
H   −0.36979   −0.99351   +3.07283
H   −0.20526   +0.74163   +3.12717
C   −3.24087   −0.97643   +2.99768
H   −2.09034   +0.00251   +4.51381
H   −2.62057   +1.05955   +3.23286
C   −2.78578   −2.40810   +3.21703
H   −4.16595   −0.80994   +3.55305
H   −3.49481   −0.82509   +1.94774
C   −3.87508   −3.44386   +2.99238
H   −1.94308   −2.63255   +2.55921
H   −2.40659   −2.50420   +4.23638
C   −4.40131   −3.48090   +1.57030
H   −3.48510   −4.42725   +3.25441
H   −4.69999   −3.25113   +3.68056
```

```
H   -5.12135   -4.28503   +1.43723
H   -4.89801   -2.55201   +1.29890
H   -3.59136   -3.64245   +0.85975
24
 * E =  +1.843  kcal/mol ; (143) 059_182_302_304_189_297
H   +0.00000   +0.00000   +0.00000
O   +0.00000   +0.00000   +0.95445
C   -0.90205   +0.00000   +1.26636
C   -1.69131   -1.19176   +0.77311
H   -0.83093   -0.01057   +2.35121
H   -1.41100   +0.92786   +0.98628
C   -3.14344   -1.18864   +1.22577
H   -1.66425   -1.20259   -0.31884
H   -1.18782   -2.10116   +1.09983
C   -3.34277   -1.15145   +2.73435
H   -3.65551   -0.33461   +0.77954
H   -3.63256   -2.07774   +0.82622
C   -2.65574   -2.28800   +3.47008
H   -2.99860   -0.19406   +3.12676
H   -4.41349   -1.18984   +2.94361
C   -3.03360   -2.38915   +4.93874
H   -2.90119   -3.22865   +2.97278
H   -1.57193   -2.17765   +3.39115
C   -2.63763   -1.17564   +5.75795
H   -4.11016   -2.54885   +5.02129
H   -2.56214   -3.27558   +5.36264
H   -2.85684   -1.32411   +6.81275
H   -1.57058   -0.97590   +5.66752
H   -3.16868   -0.28158   +5.43881
24
 * E =  +1.858  kcal/mol ; (144) 061_173_059_055_055_058
H   +0.00000   +0.00000   +0.00000
O   +0.00000   +0.00000   +0.95444
C   -0.90231   +0.00000   +1.26557
C   -1.67164   -1.22257   +0.81748
H   -0.83830   +0.02225   +2.35159
H   -1.41364   +0.91459   +0.95406
C   -3.05231   -1.34132   +1.44371
H   -1.75816   -1.21090   -0.27221
H   -1.08014   -2.10195   +1.06924
C   -3.98567   -0.16815   +1.17519
H   -3.51666   -2.26193   +1.08818
H   -2.94306   -1.46212   +2.52240
C   -4.17591   +0.16411   -0.29753
H   -4.95359   -0.38619   +1.62683
```

```
H    -3.61908    +0.72070    +1.68937
C    -4.64942    -0.99866    -1.15627
H    -4.89437    +0.98122    -0.37947
H    -3.24025    +0.54901    -0.70780
C    -5.96383    -1.59902    -0.69680
H    -4.75092    -0.65276    -2.18474
H    -3.88433    -1.77639    -1.17756
H    -6.30431    -2.37295    -1.38058
H    -5.87629    -2.05157    +0.28926
H    -6.74278    -0.83968    -0.64113
24
 * E =   +1.871  kcal/mol  ;  (145) 063_275_056_172_178_180
H    +0.00000    +0.00000    +0.00000
O    +0.00000    +0.00000    +0.95332
C    -0.89821    +0.00000    +1.27276
C    -1.68386    -1.24138    +0.89224
H    -0.80985    +0.06361    +2.35370
H    -1.42839    +0.89861    +0.94016
C    -2.32570    -1.20901    -0.48592
H    -1.01157    -2.09604    +0.97760
H    -2.47093    -1.39115    +1.63188
C    -1.37572    -0.95761    -1.64379
H    -2.83604    -2.15857    -0.65381
H    -3.10442    -0.44353    -0.50032
C    -2.03323    -1.13807    -2.99858
H    -0.98905    +0.06449    -1.58918
H    -0.51661    -1.62908    -1.56808
C    -1.10812    -0.84767    -4.16433
H    -2.40777    -2.16064    -3.08018
H    -2.90977    -0.48945    -3.06234
C    -1.77674    -1.04187    -5.51014
H    -0.73925    +0.17615    -4.08379
H    -0.23044    -1.49224    -4.09511
H    -1.09563    -0.82726    -6.33014
H    -2.12598    -2.06608    -5.62941
H    -2.63977    -0.38680    -5.61655
24
 * E =   +1.873  kcal/mol  ;  (146) 066_301_300_179_057_056
H    +0.00000    +0.00000    +0.00000
O    +0.00000    +0.00000    +0.95441
C    -0.90132    +0.00000    +1.26828
C    -1.64978    -1.27031    +0.92109
H    -0.83591    +0.10381    +2.34788
H    -1.43683    +0.87683    +0.89602
C    -1.72801    -1.57102    -0.56789
```

```
 H    −1.16647   −2.10450   +1.42827
 H    −2.65918   −1.19165   +1.32959
 C    −2.42738   −0.50196   −1.38782
 H    −0.72241   −1.73733   −0.96344
 H    −2.24573   −2.52155   −0.69547
 C    −2.48483   −0.80525   −2.87787
 H    −3.43715   −0.35745   −0.99884
 H    −1.92482   +0.45779   −1.25723
 C    −3.16079   −2.11768   −3.24586
 H    −3.00685   +0.01177   −3.37855
 H    −1.46995   −0.80782   −3.27952
 C    −4.57393   −2.24579   −2.71187
 H    −3.17603   −2.20336   −4.33213
 H    −2.56062   −2.95604   −2.89147
 H    −5.05112   −3.15365   −3.07309
 H    −4.59093   −2.28056   −1.62386
 H    −5.18869   −1.40185   −3.02241
24
 * E =  +1.884  kcal/mol  ;  (147)  065_074_299_299_184_181
 H    +0.00000   +0.00000   +0.00000
 O    +0.00000   +0.00000   +0.95422
 C    −0.90222   +0.00000   +1.26492
 C    −1.67449   −1.25510   +0.91288
 H    −0.83731   +0.10724   +2.34624
 H    −1.42994   +0.87844   +0.88574
 C    −1.30034   −2.49184   +1.71619
 H    −2.73373   −1.04867   +1.07334
 H    −1.57097   −1.44606   −0.15786
 C    +0.13924   −2.96565   +1.59016
 H    −1.51372   −2.29193   +2.76780
 H    −1.96665   −3.30564   +1.42390
 C    +0.54288   −3.35860   +0.18268
 H    +0.81514   −2.19101   +1.94934
 H    +0.27217   −3.82792   +2.24627
 C    +1.94626   −3.92801   +0.10515
 H    −0.16706   −4.09026   −0.21259
 H    +0.48115   −2.48877   −0.47383
 C    +2.35491   −4.30803   −1.30359
 H    +2.64836   −3.19628   +0.50693
 H    +2.01455   −4.80226   +0.75458
 H    +3.36344   −4.71406   −1.33347
 H    +1.68353   −5.05876   −1.71764
 H    +2.32760   −3.44383   −1.96563
24
 * E =  +1.895  kcal/mol  ;  (148)  062_173_064_169_057_057
```

```
H    +0.00000    +0.00000    +0.00000
O    +0.00000    +0.00000    +0.95444
C    -0.90237    +0.00000    +1.26538
C    -1.66854    -1.22870    +0.82690
H    -0.83819    +0.02772    +2.35135
H    -1.41485    +0.91104    +0.94851
C    -3.05631    -1.33508    +1.43671
H    -1.75202    -1.22992    -0.26339
H    -1.07693    -2.10300    +1.09576
C    -4.00193    -0.21603    +1.03402
H    -3.49596    -2.29008    +1.14412
H    -2.96488    -1.36810    +2.52435
C    -5.44755    -0.46868    +1.43326
H    -3.66711    +0.72870    +1.46340
H    -3.95585    -0.08499    -0.04893
C    -5.66798    -0.70040    +2.92067
H    -6.05441    +0.38020    +1.11392
H    -5.81980    -1.33447    +0.88285
C    -5.17845    +0.43792    +3.79440
H    -6.73306    -0.85462    +3.09233
H    -5.18065    -1.62740    +3.22473
H    -5.43329    +0.27154    +4.83836
H    -4.09721    +0.55186    +3.73845
H    -5.62384    +1.38465    +3.49125
24
* E =   +1.984  kcal/mol ;  (149)  060_173_058_056_170_063
H    +0.00000    +0.00000    +0.00000
O    +0.00000    +0.00000    +0.95447
C    -0.90258    +0.00000    +1.26490
C    -1.67706    -1.21326    +0.80142
H    -0.83958    +0.00978    +2.35120
H    -1.40939    +0.92054    +0.96333
C    -3.05952    -1.33294    +1.42449
H    -1.76197    -1.18971    -0.28765
H    -1.09110    -2.09874    +1.04456
C    -3.98039    -0.14351    +1.19043
H    -3.53785    -2.23181    +1.03369
H    -2.95066    -1.49087    +2.49845
C    -4.18980    +0.18952    -0.27590
H    -4.94880    -0.36050    +1.64514
H    -3.59484    +0.72990    +1.71699
C    -5.28389    +1.21435    -0.52619
H    -3.25618    +0.55869    -0.70725
H    -4.43039    -0.72879    -0.81559
C    -5.00413    +2.57167    +0.09004
```

H −5.41845 +1.33049 −1.60149
H −6.22956 +0.82815 −0.14195
H −5.77377 +3.29180 −0.17770
H −4.96863 +2.52278 +1.17603
H −4.04883 +2.96667 −0.25382
24
* E = +1.985 kcal/mol ; (150) 178_174_057_057_177_294
H +0.00000 +0.00000 +0.00000
O +0.00000 +0.00000 +0.95350
C −0.89799 +0.00000 +1.27409
C −0.85345 −0.05939 +2.77900
H −1.42465 +0.90537 +0.95635
H −1.44628 −0.85819 +0.87643
C −2.21916 +0.08096 +3.43147
H −0.37592 −0.99249 +3.07562
H −0.20094 +0.74096 +3.12525
C −3.26223 −0.93628 +2.98936
H −2.09329 +0.00927 +4.51234
H −2.60434 +1.08366 +3.23926
C −2.84223 −2.38035 +3.19308
H −4.18094 −0.74012 +3.54141
H −3.51114 −0.78347 +1.93658
C −3.90613 −3.39624 +2.80840
H −1.94270 −2.58059 +2.60955
H −2.56306 −2.52935 +4.23935
C −5.14950 −3.35079 +3.67598
H −4.18441 −3.24482 +1.76377
H −3.47245 −4.39461 +2.86300
H −5.84576 −4.14151 +3.40608
H −4.89484 −3.47994 +4.72713
H −5.67938 −2.40602 +3.57919
24
* E = +1.988 kcal/mol ; (151) 059_182_293_179_057_056
H +0.00000 +0.00000 +0.00000
O +0.00000 +0.00000 +0.95451
C −0.90233 +0.00000 +1.26578
C −1.68982 −1.19342 +0.77039
H −0.83160 −0.00507 +2.34955
H −1.41240 +0.92512 +0.97898
C −3.14226 −1.20251 +1.21841
H −1.65490 −1.19830 −0.32151
H −1.18842 −2.10400 +1.09994
C −3.32878 −1.38080 +2.71489
H −3.62845 −0.27637 +0.90100
H −3.65837 −2.00660 +0.69399

```
C  −4.78342  −1.36139  +3.16037
H  −2.85913  −2.31721  +3.02195
H  −2.79973  −0.59339  +3.25116
C  −5.67624  −2.40350  +2.50306
H  −4.81673  −1.50216  +4.24196
H  −5.19927  −0.37045  +2.97044
C  −5.17965  −3.82579  +2.67369
H  −6.67621  −2.31682  +2.92758
H  −5.78581  −2.18160  +1.44112
H  −5.89061  −4.54318  +2.27067
H  −4.23181  −3.98464  +2.16221
H  −5.02773  −4.06440  +3.72562
24
∗ E =  +1.995 kcal/mol ; (152) 058_183_303_303_183_066
H  +0.00000  +0.00000  +0.00000
O  +0.00000  +0.00000  +0.95447
C  −0.90233  +0.00000  +1.26562
C  −1.69449  −1.18754  +0.76694
H  −0.83245  −0.01489  +2.35055
H  −1.40941  +0.93000  +0.98892
C  −3.15201  −1.16937  +1.20142
H  −1.65581  −1.20101  −0.32465
H  −1.20148  −2.10027  +1.09979
C  −3.37922  −1.10085  +2.70531
H  −3.65252  −0.32077  +0.73227
H  −3.64154  −2.06204  +0.81054
C  −2.71752  −2.22390  +3.48266
H  −3.02614  −0.14303  +3.09360
H  −4.45385  −1.11429  +2.88430
C  −3.00062  −2.19350  +4.97629
H  −3.04174  −3.18445  +3.07372
H  −1.63800  −2.18030  +3.33306
C  −4.45373  −2.43628  +5.33710
H  −2.38366  −2.94778  +5.46416
H  −2.67906  −1.23254  +5.38178
H  −4.58886  −2.47560  +6.41552
H  −5.10445  −1.65157  +4.95821
H  −4.80370  −3.38235  +4.92604
24
∗ E =  +2.022 kcal/mol ; (153) 177_174_065_180_302_304
H  +0.00000  +0.00000  +0.00000
O  +0.00000  +0.00000  +0.95349
C  −0.89792  +0.00000  +1.27424
C  −0.84847  −0.06271  +2.77988
H  −1.42090  +0.90965  +0.96305
```

```
H   −1.44704   −0.85354   +0.87147
C   −2.20642   +0.06915   +3.44783
H   −0.37421   −0.99952   +3.07384
H   −0.19150   +0.73728   +3.11871
C   −3.16231   −1.07419   +3.15604
H   −2.04962   +0.13939   +4.52413
H   −2.67078   +1.01296   +3.15058
C   −4.52186   −0.92724   +3.82319
H   −3.32087   −1.15974   +2.08099
H   −2.69767   −2.01309   +3.46366
C   −4.48905   −0.79455   +5.33862
H   −5.03039   −0.05722   +3.40419
H   −5.13319   −1.79208   +3.55986
C   −3.77251   −1.93525   +6.03427
H   −4.02380   +0.15104   +5.61817
H   −5.51519   −0.73772   +5.70135
H   −3.85118   −1.84988   +7.11551
H   −4.19437   −2.89728   +5.74580
H   −2.71280   −1.95483   +5.78576
24
 * E =  +2.037  kcal/mol ;  (154)  179_074_295_305_306_302
H   +0.00000   +0.00000   +0.00000
O   +0.00000   +0.00000   +0.95357
C   −0.89830   +0.00000   +1.27350
C   −0.88784   −0.03062   +2.78225
H   −1.43103   +0.89050   +0.92569
H   −1.43138   −0.87579   +0.89629
C   −0.50089   +1.27550   +3.45984
H   −1.89037   −0.30557   +3.11122
H   −0.22755   −0.83898   +3.09879
C   +0.92127   +1.76145   +3.21838
H   −1.19820   +2.05326   +3.14237
H   −0.65987   +1.15785   +4.53340
C   +2.00178   +0.75372   +3.58112
H   +1.04270   +2.02846   +2.17052
H   +1.06652   +2.67911   +3.79062
C   +1.91695   +0.20593   +4.99737
H   +1.96512   −0.06979   +2.86906
H   +2.97538   +1.22816   +3.44453
C   +1.97004   +1.27560   +6.07105
H   +1.00102   −0.37666   +5.11319
H   +2.73580   −0.49751   +5.14870
H   +1.98142   +0.83869   +7.06714
H   +2.86472   +1.88843   +5.96731
H   +1.11050   +1.94154   +6.01658
```

```
24
 * E =   +2.091  kcal/mol ;  (155)  062_174_067_181_303_304
 H    +0.00000    +0.00000    +0.00000
 O    +0.00000    +0.00000    +0.95443
 C    -0.90234    +0.00000    +1.26543
 C    -1.66627    -1.23054    +0.82704
 H    -0.83790    +0.02696    +2.35122
 H    -1.41386    +0.91160    +0.94945
 C    -3.06043    -1.34092    +1.42288
 H    -1.73824    -1.23997    -0.26408
 H    -1.07493    -2.10168    +1.10649
 C    -4.03374    -0.27976    +0.93992
 H    -3.45789    -2.32714    +1.18327
 H    -2.99284    -1.30190    +2.51295
 C    -5.41859    -0.38556    +1.56127
 H    -3.63974    +0.71268    +1.15748
 H    -4.11084    -0.33791    -0.14778
 C    -6.12086    -1.71968    +1.35602
 H    -5.34254    -0.18821    +2.63183
 H    -6.04483    +0.40802    +1.15025
 C    -6.25769    -2.11864    -0.10024
 H    -5.59380    -2.50407    +1.89986
 H    -7.11048    -1.66041    +1.80854
 H    -6.84460    -3.02784    -0.20693
 H    -6.75022    -1.33634    -0.67654
 H    -5.28777    -2.30214    -0.55950
24
 * E =   +2.097  kcal/mol ;  (156)  178_076_299_301_188_297
 H    +0.00000    +0.00000    +0.00000
 O    +0.00000    +0.00000    +0.95360
 C    -0.89808    +0.00000    +1.27422
 C    -0.88335    -0.04008    +2.78336
 H    -1.42914    +0.89372    +0.93181
 H    -1.43255    -0.87240    +0.89158
 C    -0.53990    +1.27571    +3.46625
 H    -1.87391    -0.35093    +3.11631
 H    -0.19475    -0.82708    +3.09073
 C    +0.83472    +1.84926    +3.15872
 H    -1.30036    +2.01254    +3.20011
 H    -0.62665    +1.12956    +4.54458
 C    +1.97767    +0.92343    +3.53098
 H    +0.89552    +2.09328    +2.09982
 H    +0.94371    +2.78890    +3.70541
 C    +3.35033    +1.56445    +3.40872
 H    +1.83626    +0.57868    +4.55812
```

```
 H    +1.94167    +0.04016    +2.89286
 C    +3.70008    +1.98579    +1.99377
 H    +3.40737    +2.43000    +4.07182
 H    +4.10066    +0.85890    +3.76618
 H    +4.72512    +2.34480    +1.93056
 H    +3.59422    +1.14892    +1.30500
 H    +3.05129    +2.78370    +1.63961
24
 * E =  +2.132  kcal/mol ; (157) 178_069_284_182_176_063
 H    +0.00000    +0.00000    +0.00000
 O    +0.00000    +0.00000    +0.95360
 C    -0.89938    +0.00000    +1.27056
 C    -0.90835    -0.05635    +2.77803
 H    -1.42308    +0.90081    +0.93643
 H    -1.43895    -0.86440    +0.87599
 C    -0.39520    +1.18922    +3.48461
 H    -1.94089    -0.23507    +3.08002
 H    -0.33572    -0.92900    +3.09514
 C    +1.11281    +1.36533    +3.47617
 H    -0.86649    +2.07043    +3.04176
 H    -0.73404    +1.16062    +4.52199
 C    +1.55354    +2.59575    +4.24684
 H    +1.57581    +0.47932    +3.91799
 H    +1.46387    +1.40870    +2.44889
 C    +3.06102    +2.75609    +4.35619
 H    +1.13265    +3.48672    +3.77375
 H    +1.12764    +2.55804    +5.25160
 C    +3.75593    +2.95387    +3.02257
 H    +3.27951    +3.60657    +5.00233
 H    +3.47777    +1.88021    +4.85667
 H    +4.82073    +3.13209    +3.15571
 H    +3.64768    +2.08409    +2.37897
 H    +3.34125    +3.81002    +2.49160
24
 * E =  +2.140  kcal/mol ; (158) 060_172_057_057_177_294
 H    +0.00000    +0.00000    +0.00000
 O    +0.00000    +0.00000    +0.95445
 C    -0.90193    +0.00000    +1.26670
 C    -1.67908    -1.21229    +0.80513
 H    -0.83636    +0.00915    +2.35292
 H    -1.40917    +0.92107    +0.96722
 C    -3.05142    -1.34008    +1.44870
 H    -1.77739    -1.18327    -0.28251
 H    -1.08784    -2.09770    +1.03555
 C    -3.97586    -0.14410    +1.26563
```

H −3.54047 −2.22863 +1.04783
H −2.92273 −1.52304 +2.51649
C −4.22547 +0.22986 −0.18381
H −4.92320 −0.37378 +1.75225
H −3.57446 +0.72401 +1.79276
C −5.20291 +1.38000 −0.36829
H −3.27937 +0.50047 −0.65508
H −4.59327 −0.64642 −0.72406
C −6.61862 +1.06762 +0.07758
H −4.83389 +2.25299 +0.17299
H −5.21723 +1.66172 −1.42102
H −7.29010 +1.89460 −0.14136
H −7.00237 +0.18636 −0.43481
H −6.67658 +0.87848 +1.14690
24
* E = +2.154 kcal/mol ; (159) 177_070_287_184_184_297
H +0.00000 +0.00000 +0.00000
O +0.00000 +0.00000 +0.95358
C −0.89879 +0.00000 +1.27214
C −0.90155 −0.06323 +2.77949
H −1.42278 +0.90252 +0.94286
H −1.44005 −0.86236 +0.87552
C −0.41047 +1.18781 +3.49087
H −1.92857 −0.26329 +3.08685
H −0.31005 −0.92624 +3.08822
C +1.08707 +1.43046 +3.42869
H −0.93689 +2.05714 +3.08856
H −0.70521 +1.12162 +4.54007
C +1.50653 +2.63477 +4.25116
H +1.59988 +0.53631 +3.78596
H +1.39754 +1.56042 +2.39363
C +2.98121 +2.98536 +4.13924
H +0.91459 +3.49773 +3.93962
H +1.25753 +2.46178 +5.30145
C +3.91099 +1.90302 +4.65358
H +3.21825 +3.20162 +3.09619
H +3.16723 +3.90748 +4.69030
H +4.94858 +2.22831 +4.62488
H +3.67426 +1.64420 +5.68508
H +3.83795 +0.99433 +4.06067
24
* E = +2.183 kcal/mol ; (160) 061_069_291_185_176_064
H +0.00000 +0.00000 +0.00000
O +0.00000 +0.00000 +0.95447
C −0.90229 +0.00000 +1.26573

C −1.71063 −1.20678 +0.83656
H −0.83418 +0.03419 +2.35131
H −1.41136 +0.91289 +0.94602
C −1.30529 −2.53034 +1.46442
H −2.75252 −1.00172 +1.08756
H −1.67793 −1.28905 −0.25375
C +0.04054 −3.07749 +1.02343
H −1.31092 −2.42192 +2.55159
H −2.07483 −3.26974 +1.23433
C +0.32381 −4.44990 +1.60553
H +0.05978 −3.14217 −0.06845
H +0.82319 −2.37604 +1.30077
C +1.62190 −5.07627 +1.12311
H +0.34393 −4.37993 +2.69595
H −0.50493 −5.11720 +1.36078
C +2.86450 −4.30979 +1.53418
H +1.68645 −6.09373 +1.50902
H +1.59244 −5.16654 +0.03565
H +3.76822 −4.82910 +1.22316
H +2.89102 −3.31571 +1.09421
H +2.90750 −4.19074 +2.61606
24
* E = +2.184 kcal/mol ; (161) 062_067_289_190_296_185
H +0.00000 +0.00000 +0.00000
O +0.00000 +0.00000 +0.95445
C −0.90187 +0.00000 +1.26686
C −1.69973 −1.22193 +0.86265
H −0.83205 +0.05399 +2.35152
H −1.41922 +0.90271 +0.93183
C −1.24939 −2.53401 +1.48461
H −2.73775 −1.03433 +1.14191
H −1.69475 −1.30885 −0.22781
C +0.08061 −3.06576 +0.97610
H −1.21198 −2.40970 +2.56807
H −2.01780 −3.28668 +1.29432
C +0.38616 −4.48022 +1.43940
H +0.06641 −3.04877 −0.11605
H +0.88374 −2.39570 +1.27774
C +0.54604 −4.62639 +2.94185
H −0.40242 −5.15518 +1.09659
H +1.30448 −4.81954 +0.95688
C +0.96098 −6.02573 +3.35003
H +1.28699 −3.90590 +3.29293
H −0.38844 −4.36922 +3.44069
H +1.06655 −6.11512 +4.42881

```
H    +0.22541    -6.76114    +3.02761
H    +1.91435    -6.29795    +2.89998
24
* E =   +2.187  kcal/mol ; (162) 062_068_290_185_184_297
H    +0.00000    +0.00000    +0.00000
O    +0.00000    +0.00000    +0.95444
C    -0.90225    +0.00000    +1.26574
C    -1.70362    -1.21658    +0.85170
H    -0.83412    +0.04731    +2.35081
H    -1.41608    +0.90639    +0.93534
C    -1.27759    -2.53135    +1.48411
H    -2.74480    -1.01972    +1.11222
H    -1.68107    -1.30582    -0.23827
C    +0.06331    -3.07533    +1.02363
H    -1.26544    -2.41392    +2.57027
H    -2.04537    -3.27839    +1.27347
C    +0.35465    -4.44718    +1.60360
H    +0.06476    -3.12600    -0.06725
H    +0.85656    -2.38178    +1.29790
C    +1.72968    -4.99389    +1.25699
H    +0.25904    -4.39584    +2.68978
H    -0.40857    -5.15314    +1.26611
C    +1.94266    -5.22674    -0.22658
H    +2.49134    -4.30755    +1.63036
H    +1.87953    -5.93279    +1.79016
H    +2.90826    -5.68923    -0.41822
H    +1.17479    -5.88479    -0.63195
H    +1.91063    -4.29670    -0.78956
24
* E =   +2.189  kcal/mol ; (163) 178_069_288_190_296_185
H    +0.00000    +0.00000    +0.00000
O    +0.00000    +0.00000    +0.95356
C    -0.89808    +0.00000    +1.27407
C    -0.89424    -0.04294    +2.78205
H    -1.42694    +0.89611    +0.93517
H    -1.43713    -0.86962    +0.89035
C    -0.39501    +1.21728    +3.47200
H    -1.92020    -0.23537    +3.09776
H    -0.30277    -0.90281    +3.09968
C    +1.10327    +1.45465    +3.37806
H    -0.93788    +2.07474    +3.06978
H    -0.66793    +1.15868    +4.52818
C    +1.59453    +2.58547    +4.26584
H    +1.61771    +0.53389    +3.65720
H    +1.38207    +1.64526    +2.34346
```

C +1.04177 +3.95273 +3.90610
H +1.34911 +2.36494 +5.30812
H +2.68412 +2.62638 +4.21538
C +1.64310 +5.06397 +4.74307
H +1.23069 +4.14608 +2.84840
H −0.04160 +3.95816 +4.02724
H +1.23330 +6.03571 +4.47670
H +1.44997 +4.90286 +5.80260
H +2.72297 +5.11211 +4.61171
24
* E = +2.233 kcal/mol ; (164) 180_077_300_300_182_065
H +0.00000 +0.00000 +0.00000
O +0.00000 +0.00000 +0.95361
C −0.89858 +0.00000 +1.27288
C −0.88419 −0.01222 +2.78225
H −1.43553 +0.88348 +0.91352
H −1.42614 −0.88308 +0.90546
C −0.56948 +1.32398 +3.43839
H −1.86838 −0.33596 +3.12170
H −0.18135 −0.78063 +3.10386
C +0.78295 +1.93790 +3.11017
H −1.35367 +2.03356 +3.16697
H −0.64322 +1.19561 +4.51997
C +1.96266 +1.06606 +3.49581
H +0.84108 +2.15255 +2.04308
H +0.84634 +2.89594 +3.62703
C +3.31566 +1.69491 +3.20464
H +1.90092 +0.82187 +4.56032
H +1.89373 +0.12640 +2.95065
C +3.60996 +2.94437 +4.01307
H +4.09386 +0.95647 +3.39827
H +3.37640 +1.92497 +2.13963
H +4.61605 +3.31070 +3.82046
H +2.92081 +3.75231 +3.77790
H +3.53046 +2.74455 +5.08115
24
* E = +2.242 kcal/mol ; (165) 070_277_060_058_176_180
H +0.00000 +0.00000 +0.00000
O +0.00000 +0.00000 +0.95308
C −0.89813 +0.00000 +1.27201
C −1.64086 −1.29933 +1.02526
H −0.81467 +0.17581 +2.34096
H −1.45839 +0.84086 +0.85085
C −2.24801 −1.45599 −0.36176
H −0.96113 −2.12178 +1.24826

H −2.45059 −1.36714 +1.75277
C −1.28134 −1.40428 −1.53527
H −2.78111 −2.40723 −0.39791
H −3.00512 −0.68207 −0.49909
C −0.17742 −2.44419 −1.48868
H −1.85230 −1.53847 −2.45550
H −0.84950 −0.40241 −1.62513
C +0.71130 −2.42538 −2.71793
H +0.43736 −2.29917 −0.59894
H −0.62815 −3.43408 −1.38506
C +1.80885 −3.46877 −2.66733
H +0.09882 −2.57874 −3.60789
H +1.15585 −1.43410 −2.82619
H +2.43373 −3.43613 −3.55670
H +2.45314 −3.31678 −1.80325
H +1.39127 −4.47144 −2.59275
24
∗ E = +2.243 kcal/mol ; (166) 064_071_295_303_304_186
H +0.00000 +0.00000 +0.00000
O +0.00000 +0.00000 +0.95430
C −0.90271 +0.00000 +1.26382
C −1.68826 −1.23965 +0.88801
H −0.83817 +0.08377 +2.34708
H −1.42447 +0.88980 +0.90272
C −1.28587 −2.51300 +1.61565
H −2.73868 −1.03670 +1.10252
H −1.63212 −1.38367 −0.19458
C +0.11615 −3.03526 +1.33258
H −1.38327 −2.34160 +2.68909
H −2.01315 −3.28909 +1.37425
C +0.41649 −3.30121 −0.13335
H +0.85063 −2.33140 +1.71733
H +0.24748 −3.96453 +1.89024
C −0.54802 −4.25510 −0.81306
H +0.43357 −2.35827 −0.68383
H +1.42767 −3.70345 −0.21319
C −0.12948 −4.59856 −2.22854
H −0.62614 −5.16899 −0.22079
H −1.54857 −3.82033 −0.82904
H −0.83621 −5.27418 −2.70492
H −0.06233 −3.70284 −2.84442
H +0.84821 −5.07738 −2.24190
24
∗ E = +2.285 kcal/mol ; (167) 180_066_279_176_063_175
H +0.00000 +0.00000 +0.00000

```
O    +0.00000    +0.00000    +0.95360
C    −0.89952    +0.00000    +1.27017
C    −0.91006    +0.00805    +2.77844
H    −1.43397    +0.88004    +0.90009
H    −1.42791    −0.88646    +0.91060
C    −0.35487    +1.26947    +3.42613
H    −1.94813    −0.12783    +3.08340
H    −0.36448    −0.86597    +3.13696
C    +1.16121    +1.31687    +3.51990
H    −0.71144    +2.14296    +2.87331
H    −0.77787    +1.35485    +4.42694
C    +1.70112    +2.61786    +4.08963
H    +1.50042    +0.48454    +4.14164
H    +1.58210    +1.15035    +2.53200
C    +1.28870    +2.90736    +5.52159
H    +2.79131    +2.59720    +4.03953
H    +1.38784    +3.44891    +3.45258
C    +1.94935    +4.15314    +6.07736
H    +0.20614    +3.01870    +5.58248
H    +1.54180    +2.04835    +6.14626
H    +1.64173    +4.34922    +7.10208
H    +3.03399    +4.05749    +6.06910
H    +1.69431    +5.02867    +5.48216
24
* E =   +2.300  kcal/mol  ;  (168) 179_058_270_179_301_307
H    +0.00000    +0.00000    +0.00000
O    +0.00000    +0.00000    +0.95354
C    −0.89743    +0.00000    +1.27580
C    −0.88057    −0.01524    +2.78381
H    −1.42182    +0.89609    +0.93092
H    −1.44093    −0.87116    +0.90238
C    −0.12648    +1.15862    +3.39843
H    −1.91965    −0.01225    +3.11307
H    −0.44748    −0.95593    +3.12618
C    +1.34956    +0.88256    +3.62751
H    −0.24384    +2.02733    +2.74702
H    −0.58342    +1.43111    +4.35132
C    +2.11146    +2.06165    +4.21161
H    +1.44269    +0.03351    +4.30711
H    +1.80182    +0.56905    +2.68895
C    +2.06318    +3.32909    +3.36988
H    +1.72816    +2.28468    +5.20937
H    +3.15427    +1.76834    +4.34514
C    +2.49550    +3.11562    +1.93212
H    +1.05493    +3.74443    +3.38619
```

H	+2.70019	+4.08132	+3.83550
H	+2.51487	+4.05436	+1.38261
H	+3.49446	+2.68317	+1.88509
H	+1.82239	+2.43631	+1.41117

24

* E = +2.327 kcal/mol ; (169) 299_303_301_097_185_182

H	+0.00000	+0.00000	+0.00000
O	+0.00000	+0.00000	+0.95459
C	−0.90182	+0.00000	+1.26757
C	−1.68421	+1.21720	+0.82719
H	−1.41892	−0.90966	+0.95303
H	−0.83359	−0.03292	+2.35289
C	−1.07082	+2.54690	+1.23817
H	−1.80192	+1.19091	−0.25894
H	−2.69110	+1.13640	+1.24170
C	−0.89430	+2.71485	+2.74589
H	−0.10230	+2.66077	+0.75005
H	−1.70519	+3.34085	+0.84653
C	+0.50421	+2.39054	+3.24277
H	−1.13086	+3.73997	+3.03283
H	−1.62170	+2.08876	+3.27044
C	+0.63148	+2.45692	+4.75184
H	+0.81107	+1.40671	+2.88730
H	+1.20656	+3.09493	+2.79116
C	+2.03958	+2.17914	+5.23827
H	+0.31152	+3.44142	+5.09836
H	−0.05841	+1.74072	+5.20207
H	+2.10664	+2.22606	+6.32288
H	+2.37210	+1.19046	+4.92691
H	+2.74327	+2.90343	+4.83126

24

* E = +2.328 kcal/mol ; (170) 061_300_301_092_177_181

H	+0.00000	+0.00000	+0.00000
O	+0.00000	+0.00000	+0.95508
C	−0.90240	+0.00000	+1.26789
C	−1.68493	−1.21777	+0.82854
H	−0.83609	+0.02430	+2.35225
H	−1.41972	+0.91371	+0.96070
C	−1.81111	−1.39545	−0.67837
H	−1.21333	−2.10357	+1.25307
H	−2.68430	−1.15082	+1.26260
C	−2.48416	−0.24200	−1.41824
H	−0.82608	−1.58164	−1.11413
H	−2.37318	−2.31175	−0.85202
C	−1.53636	+0.81976	−1.95109

```
H   -3.04386   -0.63935   -2.26557
H   -3.22549   +0.22674   -0.76646
C   -2.24201   +1.90747   -2.73860
H   -0.98204   +1.28938   -1.13612
H   -0.79321   +0.34035   -2.59383
C   -1.29174   +2.95506   -3.28191
H   -2.79491   +1.45028   -3.56059
H   -2.98755   +2.38391   -2.10028
H   -1.82059   +3.72203   -3.84264
H   -0.75006   +3.44837   -2.47668
H   -0.55492   +2.50809   -3.94723
24
* E =  +2.336  kcal/mol ; (171) 176_059_060_263_176_178
H   +0.00000   +0.00000   +0.00000
O   +0.00000   +0.00000   +0.95345
C   -0.89725   +0.00000   +1.27596
C   -0.85972   -0.09202   +2.77994
H   -1.41835   +0.91181   +0.96864
H   -1.45308   -0.84924   +0.87226
C   -0.11155   +1.04264   +3.46272
H   -1.88970   -0.11831   +3.13981
H   -0.40374   -1.04336   +3.05283
C   -0.68341   +2.43175   +3.18728
H   -0.12077   +0.84529   +4.53376
H   +0.93302   +1.01165   +3.15346
C   +0.02870   +3.18579   +2.07767
H   -1.74701   +2.34875   +2.94572
H   -0.63612   +3.03625   +4.09382
C   -0.61220   +4.52102   +1.75481
H   +1.06754   +3.34611   +2.37429
H   +0.07612   +2.57041   +1.17882
C   +0.12558   +5.28640   +0.67476
H   -1.64649   +4.35807   +1.44613
H   -0.66140   +5.12524   +2.66247
H   -0.35290   +6.23890   +0.45839
H   +1.15225   +5.49167   +0.97333
H   +0.16328   +4.71649   -0.25229
24
* E =  +2.342  kcal/mol ; (172) 069_300_092_183_182_180
H   +0.00000   +0.00000   +0.00000
O   +0.00000   +0.00000   +0.95417
C   -0.90203   +0.00000   +1.26526
C   -1.64315   -1.29318   +0.99884
H   -0.84430   +0.18266   +2.33511
H   -1.45465   +0.83453   +0.82317
```

C	−1.73892	−1.66142	−0.47883
H	−1.15980	−2.10217	+1.54749
H	−2.64201	−1.18235	+1.42181
C	−0.61888	−2.56350	−0.97089
H	−2.68726	−2.16317	−0.67171
H	−1.76877	−0.74806	−1.08201
C	−0.69748	−2.85608	−2.45596
H	+0.35209	−2.12797	−0.73152
H	−0.65947	−3.50251	−0.41503
C	+0.39161	−3.79126	−2.94516
H	−1.67388	−3.28769	−2.68856
H	−0.64398	−1.91867	−3.01517
C	+0.30549	−4.07200	−4.43167
H	+1.36633	−3.36168	−2.70895
H	+0.33320	−4.72925	−2.39104
H	+1.09469	−4.74485	−4.75878
H	−0.64797	−4.53047	−4.68887
H	+0.39347	−3.15337	−5.00947

24

∗ E = +2.360 kcal/mol ; (173) 062_069_290_181_065_176

H	+0.00000	+0.00000	+0.00000
O	+0.00000	+0.00000	+0.95445
C	−0.90225	+0.00000	+1.26578
C	−1.70675	−1.21625	+0.85563
H	−0.83413	+0.05049	+2.35072
H	−1.41575	+0.90576	+0.93312
C	−1.30232	−2.53033	+1.50539
H	−2.74908	−1.00778	+1.10221
H	−1.67358	−1.31667	−0.23311
C	+0.03424	−3.09146	+1.05052
H	−1.28454	−2.39781	+2.59019
H	−2.08711	−3.25973	+1.30484
C	+0.39657	−4.41714	+1.69992
H	+0.01476	−3.21888	−0.03554
H	+0.81473	−2.36373	+1.25730
C	−0.51714	−5.57557	+1.34296
H	+1.41650	−4.67988	+1.41385
H	+0.41307	−4.29457	+2.78571
C	−0.04579	−6.89021	+1.93194
H	−1.53113	−5.37265	+1.68712
H	−0.57600	−5.66122	+0.25610
H	−0.71037	−7.71012	+1.66924
H	+0.95112	−7.14374	+1.57488
H	+0.00034	−6.83687	+3.01853

24

```
* E =  +2.461  kcal/mol ; (174) 065_071_295_303_307_302
H   +0.00000   +0.00000   +0.00000
O   +0.00000   +0.00000   +0.95423
C   -0.90200   +0.00000   +1.26560
C   -1.67572   -1.25556   +0.91989
H   -0.83595   +0.10898   +2.34655
H   -1.43226   +0.87670   +0.88555
C   -1.25282   -2.50727   +1.67385
H   -2.72760   -1.06054   +1.13455
H   -1.62104   -1.42302   -0.15917
C   +0.15311   -3.01808   +1.39166
H   -1.34651   -2.31333   +2.74381
H   -1.97189   -3.29840   +1.45345
C   +0.43282   -3.32436   -0.07221
H   +0.88246   -2.28816   +1.73569
H   +0.31107   -3.91965   +1.98499
C   -0.56391   -4.26170   -0.73625
H   +0.46970   -2.39027   -0.63427
H   +1.43223   -3.75533   -0.15061
C   -0.68910   -5.60527   -0.04455
H   -1.54510   -3.78530   -0.78018
H   -0.26203   -4.41616   -1.77221
H   -1.35383   -6.27101   -0.59020
H   +0.28027   -6.09567   +0.03466
H   -1.08620   -5.50136   +0.96360
24
* E =  +2.471  kcal/mol ; (175) 064_277_059_167_062_176
H   +0.00000   +0.00000   +0.00000
O   +0.00000   +0.00000   +0.95322
C   -0.89850   +0.00000   +1.27153
C   -1.68200   -1.24590   +0.90234
H   -0.81198   +0.07273   +2.35208
H   -1.43082   +0.89415   +0.93070
C   -2.30031   -1.23987   -0.48700
H   -1.01643   -2.10247   +1.01758
H   -2.48259   -1.37391   +1.63164
C   -1.31525   -1.06690   -1.63218
H   -2.83868   -2.17853   -0.63212
H   -3.05255   -0.45111   -0.53349
C   -1.90598   -1.36426   -3.00058
H   -0.93902   -0.03915   -1.64219
H   -0.45627   -1.72057   -1.46796
C   -3.05665   -0.45682   -3.39485
H   -1.11986   -1.28362   -3.75301
H   -2.24310   -2.40303   -3.02478
```

```
C   -3.54871   -0.72169   -4.80350
H   -3.88288   -0.58231   -2.69526
H   -2.73814   +0.58384   -3.30691
H   -4.37563   -0.06729   -5.06880
H   -2.75482   -0.56495   -5.53186
H   -3.89274   -1.74914   -4.91024
24
* E =  +2.492 kcal/mol ; (176) 069_279_063_054_057_175
H   +0.00000   +0.00000   +0.00000
O   +0.00000   +0.00000   +0.95310
C   -0.89778   +0.00000   +1.27310
C   -1.64960   -1.29244   +1.02034
H   -0.81321   +0.16716   +2.34350
H   -1.45608   +0.84552   +0.85895
C   -2.22292   -1.46274   -0.37889
H   -0.98892   -2.12315   +1.27248
H   -2.47867   -1.33607   +1.72789
C   -1.21779   -1.52047   -1.52228
H   -2.82132   -2.37384   -0.39475
H   -2.92269   -0.64773   -0.57196
C   -0.12216   -2.56367   -1.36498
H   -1.76545   -1.72135   -2.44464
H   -0.76975   -0.53534   -1.67662
C   -0.64289   -3.97897   -1.19640
H   +0.52409   -2.52776   -2.24369
H   +0.51161   -2.31423   -0.51229
C   +0.46970   -5.00760   -1.17301
H   -1.21895   -4.05120   -0.27303
H   -1.33847   -4.20667   -2.00668
H   +0.08174   -6.01493   -1.04128
H   +1.03874   -4.98995   -2.10102
H   +1.16512   -4.81109   -0.35879
24
* E =  +2.495 kcal/mol ; (177) 064_276_057_171_174_063
H   +0.00000   +0.00000   +0.00000
O   +0.00000   +0.00000   +0.95329
C   -0.89830   +0.00000   +1.27238
C   -1.68171   -1.24446   +0.89773
H   -0.81055   +0.06898   +2.35303
H   -1.42996   +0.89598   +0.93517
C   -2.31626   -1.22309   -0.48382
H   -1.01007   -2.09855   +0.99349
H   -2.47257   -1.38783   +1.63465
C   -1.35979   -0.98836   -1.64033
H   -2.82958   -2.17235   -0.64503
```

H	−3.09217	−0.45498	−0.51013
C	−2.02243	−1.18653	−2.99170
H	−0.97475	+0.03569	−1.60089
H	−0.50314	−1.65810	−1.54360
C	−1.14569	−0.82893	−4.18000
H	−2.34358	−2.22681	−3.07938
H	−2.93205	−0.58463	−3.02558
C	+0.09647	−1.68957	−4.30967
H	−1.73769	−0.91758	−5.09063
H	−0.85663	+0.22122	−4.10921
H	+0.65091	−1.44624	−5.21291
H	+0.77338	−1.55588	−3.46871
H	−0.16467	−2.74595	−4.35684

24

* E = +2.534 kcal/mol ; (178) 063_055_090_299_301_181

H	+0.00000	+0.00000	+0.00000
O	+0.00000	+0.00000	+0.95470
C	−0.90145	+0.00000	+1.26907
C	−1.66897	−1.24151	+0.85510
H	−0.82624	+0.07913	+2.34886
H	−1.43027	+0.89005	+0.91932
C	−1.02821	−2.56425	+1.26053
H	−2.68317	−1.17241	+1.25511
H	−1.77766	−1.21318	−0.23154
C	−1.44972	−3.10488	+2.61999
H	−1.28569	−3.31781	+0.51700
H	+0.05535	−2.45722	+1.21229
C	−1.12280	−2.23209	+3.82046
H	−2.52534	−3.29071	+2.60278
H	−0.97816	−4.07916	+2.76058
C	+0.35568	−1.93740	+3.99421
H	−1.67930	−1.29413	+3.76177
H	−1.49029	−2.72919	+4.72014
C	+0.63831	−1.08978	+5.21821
H	+0.89764	−2.88218	+4.06803
H	+0.74628	−1.43516	+3.10876
H	+1.70194	−0.89570	+5.33390
H	+0.13417	−0.12653	+5.15036
H	+0.28804	−1.57873	+6.12607

24

* E = +2.539 kcal/mol ; (179) 065_058_273_181_301_306

H	+0.00000	+0.00000	+0.00000
O	+0.00000	+0.00000	+0.95466
C	−0.90194	+0.00000	+1.26752
C	−1.67613	−1.25252	+0.91617

H	−0.82755	+0.08988	+2.34926
H	−1.43433	+0.88318	+0.90534
C	−1.04626	−2.53916	+1.43389
H	−2.67756	−1.13271	+1.33168
H	−1.80758	−1.31355	−0.16764
C	−0.00698	−3.14254	+0.50494
H	−0.59724	−2.33742	+2.40832
H	−1.82646	−3.28219	+1.60781
C	+0.60008	−4.43597	+1.02523
H	−0.47393	−3.33822	−0.46250
H	+0.78108	−2.41346	+0.32518
C	+1.29440	−4.31330	+2.37427
H	−0.17853	−5.19812	+1.09438
H	+1.32022	−4.80193	+0.29133
C	+2.35637	−3.23135	+2.41333
H	+0.55547	−4.12424	+3.15366
H	+1.74435	−5.27501	+2.62093
H	+2.88208	−3.23056	+3.36551
H	+3.09616	−3.38208	+1.62771
H	+1.92840	−2.24013	+2.27195

24

∗ E = +2.551 kcal/mol ; (180) 181_066_278_175_056_057

H	+0.00000	+0.00000	+0.00000
O	+0.00000	+0.00000	+0.95358
C	−0.89909	+0.00000	+1.27130
C	−0.90650	+0.02630	+2.77938
H	−1.43760	+0.87373	+0.89228
H	−1.42476	−0.89256	+0.92294
C	−0.34646	+1.29550	+3.40720
H	−1.94405	−0.10433	+3.08829
H	−0.36092	−0.84364	+3.14779
C	+1.16858	+1.33167	+3.51239
H	−0.69340	+2.16175	+2.83709
H	−0.77450	+1.40420	+4.40473
C	+1.71164	+2.65466	+4.02984
H	+1.49368	+0.51808	+4.16411
H	+1.59963	+1.12462	+2.53654
C	+1.17054	+3.09672	+5.38159
H	+2.79903	+2.58393	+4.09284
H	+1.50147	+3.43450	+3.29545
C	+1.38897	+2.08132	+6.48617
H	+1.64880	+4.03689	+5.65654
H	+0.10599	+3.31908	+5.29898
H	+1.06516	+2.46848	+7.44965
H	+0.83551	+1.16235	+6.30087

H +2.44215 +1.81639 +6.57160
24
* E = +2.569 kcal/mol ; (181) 071_275_056_060_182_068
H +0.00000 +0.00000 +0.00000
O +0.00000 +0.00000 +0.95383
C −0.89632 +0.00000 +1.28003
C −1.63566 −1.30696 +1.05880
H −0.80467 +0.19126 +2.34570
H −1.46418 +0.83258 +0.85250
C −2.28964 −1.46449 −0.30728
H −0.93847 −2.12106 +1.25612
H −2.41761 −1.38662 +1.81483
C −1.36824 −1.31416 −1.50771
H −2.76983 −2.44316 −0.35259
H −3.09267 −0.73062 −0.39315
C −0.23420 −2.32335 −1.55315
H −1.96136 −1.41408 −2.41841
H −0.98228 −0.29200 −1.54118
C +0.70524 −2.14299 −2.73476
H +0.34771 −2.27393 −0.63125
H −0.66808 −3.32405 −1.58348
C +1.53023 −0.87139 −2.66166
H +1.37933 −2.99764 −2.78154
H +0.13141 −2.15653 −3.66291
H +2.23729 −0.80917 −3.48547
H +0.90944 +0.02185 −2.70981
H +2.10480 −0.83433 −1.73611
24
* E = +2.570 kcal/mol ; (182) 062_275_056_172_182_295
H +0.00000 +0.00000 +0.00000
O +0.00000 +0.00000 +0.95335
C −0.89861 +0.00000 +1.27174
C −1.68984 −1.23120 +0.87027
H −0.81122 +0.04649 +2.35359
H −1.42344 +0.90648 +0.95222
C −2.33244 −1.17175 −0.50677
H −1.02096 −2.08997 +0.94005
H −2.47706 −1.39118 +1.60758
C −1.38330 −0.89537 −1.66004
H −2.84272 −2.11795 −0.69313
H −3.11141 −0.40634 −0.50543
C −2.05053 −1.04808 −3.01487
H −0.99481 +0.12235 −1.57379
H −0.52728 −1.57359 −1.60416
C −1.12854 −0.81629 −4.20059

H −2.47184 −2.05218 −3.08183
H −2.89642 −0.35939 −3.07590
C −0.59691 +0.60063 −4.30274
H −0.29366 −1.51717 −4.14828
H −1.66927 −1.05906 −5.11503
H −0.00846 +0.73506 −5.20722
H −1.41258 +1.32201 −4.32929
H +0.04282 +0.85812 −3.46149
24
* E = +2.573 kcal/mol ; (183) 066_073_298_299_190_298
H +0.00000 +0.00000 +0.00000
O +0.00000 +0.00000 +0.95416
C −0.90222 +0.00000 +1.26468
C −1.67083 −1.26030 +0.92367
H −0.83801 +0.11665 +2.34507
H −1.43287 +0.87342 +0.87798
C −1.28663 −2.49099 +1.73139
H −2.72986 −1.05688 +1.08947
H −1.57239 −1.45754 −0.14648
C +0.14792 −2.97298 +1.57885
H −1.47858 −2.28060 +2.78512
H −1.96187 −3.30444 +1.45895
C +0.50840 −3.37905 +0.16182
H +0.82954 −2.19769 +1.92206
H +0.28992 −3.83373 +2.23612
C +1.85508 −4.07370 +0.04390
H −0.26885 −4.03945 −0.22958
H +0.51188 −2.49583 −0.47986
C +3.02825 −3.20731 +0.46020
H +1.84179 −4.98307 +0.64757
H +1.99635 −4.39738 −0.98756
H +3.97399 −3.71632 +0.28804
H +3.04442 −2.27524 −0.10297
H +2.98286 −2.94726 +1.51495
24
* E = +2.579 kcal/mol ; (184) 179_058_090_299_301_180
H +0.00000 +0.00000 +0.00000
O +0.00000 +0.00000 +0.95342
C −0.89796 +0.00000 +1.27385
C −0.85610 −0.02269 +2.78482
H −1.43178 +0.87720 +0.90514
H −1.43162 −0.88471 +0.91833
C −0.09478 +1.12960 +3.43157
H −1.88149 −0.05667 +3.15849
H −0.39007 −0.96452 +3.07200

```
C   −0.92205   +2.36638   +3.75433
H   +0.33381   +0.77340   +4.36758
H   +0.75411   +1.38596   +2.79918
C   −1.57929   +3.06211   +2.57378
H   −1.69636   +2.09076   +4.47289
H   −0.27843   +3.08399   +4.26658
C   −0.60628   +3.53761   +1.51106
H   −2.32195   +2.40200   +2.12053
H   −2.14303   +3.92040   +2.94422
C   −1.30116   +4.22002   +0.34998
H   +0.10877   +4.22458   +1.96732
H   −0.01822   +2.69663   +1.14193
H   −0.59061   +4.56594   −0.39727
H   −1.99548   +3.54051   −0.14351
H   −1.87495   +5.08230   +0.68606
24
* E =  +2.583  kcal/mol ; (185) 063_061_097_300_185_181
H   +0.00000   +0.00000   +0.00000
O   +0.00000   +0.00000   +0.95455
C   −0.90193   +0.00000   +1.26711
C   −1.65614   −1.25111   +0.86338
H   −0.82830   +0.07483   +2.34818
H   −1.43136   +0.88802   +0.91324
C   −1.05780   −2.53886   +1.42257
H   −2.69837   −1.15014   +1.17463
H   −1.67686   −1.29024   −0.22778
C   −1.72412   −3.04436   +2.69244
H   −1.12220   −3.32556   +0.67224
H   +0.00671   −2.37811   +1.59901
C   −1.68266   −2.08601   +3.86753
H   −2.76615   −3.29195   +2.47513
H   −1.24771   −3.98100   +2.98705
C   −2.25174   −2.68427   +5.13991
H   −0.65129   −1.77162   +4.04504
H   −2.24137   −1.17897   +3.62641
C   −2.22874   −1.72030   +6.30871
H   −3.27611   −3.01177   +4.95457
H   −1.68871   −3.58336   +5.39497
H   −2.63837   −2.17203   +7.20920
H   −1.21227   −1.40036   +6.53167
H   −2.81283   −0.82744   +6.09149
24
* E =  +2.591  kcal/mol ; (186) 185_065_098_300_185_181
H   +0.00000   +0.00000   +0.00000
O   +0.00000   +0.00000   +0.95332
```

C −0.89772 +0.00000 +1.27412
C −0.84755 +0.12448 +2.77791
H −1.45686 +0.83078 +0.83907
H −1.40275 −0.92841 +0.99673
C −0.28209 +1.45457 +3.26911
H −1.85180 −0.03030 +3.17699
H −0.23827 −0.70098 +3.14312
C −1.33122 +2.48989 +3.64274
H +0.34423 +1.27849 +4.14217
H +0.38320 +1.85386 +2.50267
C −2.26590 +2.89767 +2.51971
H −1.92745 +2.10920 +4.47581
H −0.82546 +3.38123 +4.01835
C −3.21702 +4.01157 +2.91386
H −1.67835 +3.21372 +1.65384
H −2.85151 +2.03545 +2.19320
C −4.16092 +4.40780 +1.79670
H −3.79259 +3.69823 +3.78642
H −2.63821 +4.88060 +3.23036
H −4.83093 +5.20817 +2.10212
H −3.61008 +4.75294 +0.92319
H −4.77455 +3.56410 +1.48479
24
∗ E = +2.629 kcal/mol ; (187) 182_064_188_097_301_183
H +0.00000 +0.00000 +0.00000
O +0.00000 +0.00000 +0.95328
C −0.89672 +0.00000 +1.27675
C −0.84628 +0.03845 +2.78213
H −1.44069 +0.87146 +0.90030
H −1.42528 −0.89585 +0.94324
C −0.23158 +1.31872 +3.32120
H −1.86034 −0.07297 +3.16872
H −0.28393 −0.82832 +3.12668
C +0.01475 +1.29575 +4.82846
H +0.70611 +1.50666 +2.79733
H −0.89352 +2.14691 +3.06446
C +1.43882 +0.93573 +5.22318
H −0.21549 +2.27259 +5.25227
H −0.67969 +0.59558 +5.29933
C +1.92072 −0.41597 +4.73071
H +2.11389 +1.70692 +4.84447
H +1.52455 +0.96759 +6.31120
C +3.31775 −0.74387 +5.21844
H +1.22576 −1.18991 +5.06354
H +1.90431 −0.43591 +3.64061

```
H    +3.65284   -1.71209   +4.85367
H    +4.03402   +0.00273   +4.87888
H    +3.35903   -0.76620   +6.30643
24
 * E =  +2.632 kcal/mol ; (188) 062_069_288_179_057_056
H    +0.00000   +0.00000   +0.00000
O    +0.00000   +0.00000   +0.95447
C    -0.90227   +0.00000   +1.26579
C    -1.70381   -1.22238   +0.86809
H    -0.83443   +0.06102   +2.35018
H    -1.41870   +0.90094   +0.92464
C    -1.29110   -2.52917   +1.52716
H    -2.74564   -1.01482   +1.11742
H    -1.67475   -1.33171   -0.21992
C    +0.03032   -3.10419   +1.04840
H    -1.25125   -2.38373   +2.60984
H    -2.08287   -3.25922   +1.35289
C    +0.42293   -4.39603   +1.74975
H    -0.02819   -3.27328   -0.02952
H    +0.81785   -2.36924   +1.19481
C    -0.58971   -5.52764   +1.65215
H    +1.37305   -4.73902   +1.33657
H    +0.61171   -4.18267   +2.80334
C    -0.95576   -5.89637   +0.22780
H    -0.18018   -6.40242   +2.15715
H    -1.49380   -5.26532   +2.20256
H    -1.61061   -6.76425   +0.19942
H    -1.47226   -5.08173   -0.27711
H    -0.06702   -6.13187   -0.35653
24
 * E =  +2.640 kcal/mol ; (189) 180_183_099_300_183_180
H    +0.00000   +0.00000   +0.00000
O    +0.00000   +0.00000   +0.95351
C    -0.89775   +0.00000   +1.27480
C    -0.85788   -0.00720   +2.78246
H    -1.43214   +0.89123   +0.93295
H    -1.44333   -0.87155   +0.90567
C    -2.24611   -0.07454   +3.41561
H    -0.24973   -0.85206   +3.10833
H    -0.32935   +0.88952   +3.10063
C    -2.66290   -1.46344   +3.87416
H    -2.28632   +0.59352   +4.27416
H    -2.98849   +0.30783   +2.71009
C    -2.71371   -2.50868   +2.77675
H    -1.97340   -1.80015   +4.65151
```

H −3.64428 −1.39940 +4.34740
C −3.21391 −3.85591 +3.26193
H −3.35839 −2.15530 +1.96746
H −1.71989 −2.63632 +2.34255
C −3.25242 −4.90187 +2.16632
H −2.57528 −4.20176 +4.07626
H −4.21049 −3.73559 +3.68964
H −3.61424 −5.85802 +2.53722
H −3.90765 −4.59310 +1.35335
H −2.26156 −5.06397 +1.74524
24
* E = +2.643 kcal/mol ; (190) 298_185_099_300_184_181
H +0.00000 +0.00000 +0.00000
O +0.00000 +0.00000 +0.95443
C −0.90147 +0.00000 +1.26793
C −1.67762 +1.22636 +0.83619
H −1.42784 −0.90061 +0.93790
H −0.83586 −0.05053 +2.35121
C −3.08544 +1.28360 +1.42663
H −1.11045 +2.11482 +1.11735
H −1.72912 +1.22875 −0.25409
C −3.21723 +2.17463 +2.65192
H −3.78439 +1.63826 +0.67112
H −3.41324 +0.27207 +1.68030
C −2.34106 +1.78073 +3.82521
H −2.98584 +3.20342 +2.36694
H −4.25999 +2.17765 +2.97391
C −2.56923 +2.64341 +5.05174
H −2.52394 +0.73384 +4.08152
H −1.28931 +1.84613 +3.53926
C −1.68072 +2.25975 +6.21754
H −2.39869 +3.68887 +4.78966
H −3.61665 +2.57354 +5.34952
H −1.86328 +2.89002 +7.08478
H −1.85230 +1.22714 +6.51703
H −0.62838 +2.35394 +5.95517
24
* E = +2.647 kcal/mol ; (191) 066_074_300_298_181_065
H +0.00000 +0.00000 +0.00000
O +0.00000 +0.00000 +0.95418
C −0.90204 +0.00000 +1.26529
C −1.66820 −1.26300 +0.92801
H −0.83705 +0.11977 +2.34533
H −1.43351 +0.87192 +0.87657
C −1.29223 −2.48542 +1.75222

```
H    -2.72905    -1.05935    +1.08136
H    -1.56021    -1.46791    -0.13958
C    +0.15367    -2.94877    +1.65630
H    -1.52264    -2.27271    +2.79773
H    -1.94676    -3.30980    +1.46281
C    +0.57423    -3.36837    +0.26054
H    +0.81842    -2.15783    +2.00193
H    +0.27892    -3.78823    +2.34037
C    +2.01101    -3.85671    +0.16748
H    -0.09752    -4.15129    -0.10242
H    +0.45330    -2.52317    -0.41724
C    +2.28223    -5.13367    +0.94000
H    +2.25993    -4.01834    -0.88153
H    +2.67667    -3.06738    +0.51988
H    +3.30099    -5.48007    +0.78118
H    +2.15028    -4.99625    +2.01065
H    +1.60958    -5.93033    +0.62418
24
 * E =  +2.688  kcal/mol ; (192) 182_063_184_301_098_182
H    +0.00000    +0.00000    +0.00000
O    +0.00000    +0.00000    +0.95325
C    -0.89712    +0.00000    +1.27554
C    -0.85629    +0.04680    +2.78185
H    -1.44219    +0.86871    +0.89446
H    -1.42388    -0.89822    +0.94540
C    -0.21603    +1.31551    +3.31110
H    -1.87820    -0.04714    +3.15150
H    -0.30591    -0.82503    +3.13919
C    -0.05501    +1.36178    +4.82222
H    +0.75834    +1.43086    +2.83948
H    -0.81707    +2.17130    +2.99290
C    -1.35947    +1.24126    +5.60862
H    +0.62785    +0.56889    +5.13568
H    +0.44056    +2.29784    +5.07660
C    -1.65394    -0.15889    +6.11954
H    -1.33853    +1.91452    +6.46648
H    -2.19203    +1.58273    +4.98730
C    -2.98116    -0.25277    +6.84411
H    -1.63587    -0.86869    +5.29283
H    -0.84835    -0.46201    +6.79013
H    -3.16331    -1.25660    +7.22092
H    -3.01297    +0.43003    +7.69186
H    -3.80714    +0.00802    +6.18407
24
 * E =  +2.703  kcal/mol ; (193) 061_179_099_300_184_181
```

```
H   +0.00000   +0.00000   +0.00000
O   +0.00000   +0.00000   +0.95443
C   -0.90219   +0.00000   +1.26585
C   -1.68055   -1.21935   +0.81887
H   -0.83675   +0.01125   +2.35155
H   -1.41685   +0.91666   +0.96764
C   -3.12011   -1.23295   +1.33089
H   -1.68000   -1.26858   -0.27386
H   -1.13839   -2.09836   +1.16292
C   -4.15688   -0.76892   +0.31971
H   -3.38187   -2.24016   +1.65008
H   -3.19066   -0.61134   +2.22705
C   -3.96841   +0.64934   -0.18267
H   -4.14948   -1.45127   -0.53338
H   -5.14851   -0.85591   +0.76678
C   -5.07233   +1.09992   -1.12022
H   -3.91576   +1.33316   +0.66835
H   -3.01019   +0.73427   -0.70023
C   -4.87276   +2.51134   -1.63393
H   -5.13141   +0.40808   -1.96201
H   -6.03084   +1.02959   -0.60383
H   -5.67731   +2.81264   -2.30061
H   -4.83847   +3.22548   -0.81287
H   -3.93724   +2.59957   -2.18393
24
* E =   +2.716  kcal/mol ; (194) 064_276_058_168_057_058
H   +0.00000   +0.00000   +0.00000
O   +0.00000   +0.00000   +0.95327
C   -0.89831   +0.00000   +1.27226
C   -1.68147   -1.24668   +0.90449
H   -0.81122   +0.07440   +2.35265
H   -1.43145   +0.89354   +0.93064
C   -2.30754   -1.23647   -0.48118
H   -1.01271   -2.10164   +1.01241
H   -2.47708   -1.37980   +1.63825
C   -1.33266   -1.04214   -1.63046
H   -2.83982   -2.17782   -0.63099
H   -3.06782   -0.45355   -0.51832
C   -1.93782   -1.32894   -2.99654
H   -0.95707   -0.01511   -1.62473
H   -0.46898   -1.69448   -1.48741
C   -3.17565   -0.51020   -3.33051
H   -1.17999   -1.15427   -3.76191
H   -2.18917   -2.38923   -3.05153
C   -2.94825   +0.98798   -3.27978
```

```
H   −3.51525   −0.78982   −4.32745
H   −3.98756   −0.77802   −2.65363
H   −3.83011   +1.53183   −3.60971
H   −2.71802   +1.32693   −2.27101
H   −2.11878   +1.28133   −3.92206
24
* E =  +2.722  kcal/mol ; (195) 062_274_055_174_291_183
H   +0.00000   +0.00000   +0.00000
O   +0.00000   +0.00000   +0.95340
C   −0.89841   +0.00000   +1.27250
C   −1.69163   −1.22971   +0.86945
H   −0.80971   +0.04456   +2.35429
H   −1.42265   +0.90780   +0.95547
C   −2.35262   −1.15979   −0.49884
H   −1.01930   −2.08700   +0.92247
H   −2.46874   −1.39980   +1.61512
C   −1.41504   −0.85414   −1.65488
H   −2.85819   −2.10723   −0.68208
H   −3.13581   −0.39854   −0.47954
C   −2.07115   −0.94238   −3.02425
H   −1.02455   +0.15985   −1.53723
H   −0.55873   −1.53345   −1.62361
C   −2.45050   −2.34883   −3.45091
H   −2.96042   −0.30793   −3.03815
H   −1.39243   −0.52594   −3.77010
C   −3.04143   −2.39104   −4.84593
H   −1.56437   −2.98455   −3.40877
H   −3.16421   −2.77475   −2.74636
H   −3.30456   −3.40471   −5.13856
H   −3.94346   −1.78441   −4.90700
H   −2.33733   −2.00589   −5.58164
24
* E =  +2.742  kcal/mol ; (196) 181_063_183_064_059_266
H   +0.00000   +0.00000   +0.00000
O   +0.00000   +0.00000   +0.95327
C   −0.89713   +0.00000   +1.27556
C   −0.84806   +0.02716   +2.78160
H   −1.43774   +0.87565   +0.90449
H   −1.42704   −0.89217   +0.93457
C   −0.20970   +1.29085   +3.32566
H   −1.86461   −0.07060   +3.16614
H   −0.29887   −0.85290   +3.11587
C   −0.08934   +1.31759   +4.83966
H   +0.77078   +1.42162   +2.86965
H   −0.80562   +2.14790   +3.00573
```

```
C    +0.82122    +0.26279    +5.45460
H    +0.27902    +2.30010    +5.13971
H    −1.08473    +1.22184    +5.27866
C    +2.27154    +0.30637    +4.97626
H    +0.79515    +0.40439    +6.53492
H    +0.41471    −0.73543    +5.28114
C    +2.58280    −0.61625    +3.81051
H    +2.53102    +1.33396    +4.71236
H    +2.92645    +0.03948    +5.80535
H    +3.63840    −0.57007    +3.54943
H    +2.35726    −1.64958    +4.07242
H    +2.01503    −0.37338    +2.91595
24
* E =   +2.762  kcal/mol  ;  (197)  069_279_063_054_055_058
H    +0.00000    +0.00000    +0.00000
O    +0.00000    +0.00000    +0.95301
C    −0.89786    +0.00000    +1.27251
C    −1.64792    −1.29438    +1.02443
H    −0.81399    +0.17098    +2.34237
H    −1.45688    +0.84354    +0.85533
C    −2.22302    −1.46890    −0.37385
H    −0.98499    −2.12291    +1.27701
H    −2.47564    −1.33768    +1.73358
C    −1.21975    −1.53132    −1.51754
H    −2.82044    −2.38178    −0.38590
H    −2.92437    −0.65553    −0.56810
C    −0.13620    −2.58868    −1.35736
H    −1.76856    −1.71206    −2.44239
H    −0.75607    −0.55285    −1.66625
C    −0.66366    −3.99765    −1.13307
H    +0.49017    −2.58335    −2.25103
H    +0.51520    −2.32083    −0.52516
C    −1.57825    −4.49286    −2.23644
H    +0.18631    −4.67278    −1.03858
H    −1.18827    −4.04509    −0.17751
H    −1.86308    −5.53006    −2.07647
H    −2.49573    −3.90958    −2.29239
H    −1.08965    −4.42952    −3.20795
24
* E =   +2.763  kcal/mol  ;  (198)  179_298_177_177_262_059
H    +0.00000    +0.00000    +0.00000
O    +0.00000    +0.00000    +0.95329
C    −0.89725    +0.00000    +1.27531
C    −0.85631    −0.02307    +2.78174
H    −1.42928    +0.88992    +0.93118
```

```
H   −1.43694   −0.87751   +0.90714
C   −0.20175   −1.26994   +3.34179
H   −0.31869   +0.86196   +3.12480
H   −1.87788   +0.06351   +3.15564
C   −0.10277   −1.25318   +4.85708
H   −0.75794   −2.14883   +3.01146
H   +0.79695   −1.36355   +2.91658
C   +0.49635   −2.52394   +5.45809
H   +0.49811   −0.39031   +5.14580
H   −1.09318   −1.08109   +5.28786
C   −0.52848   −3.52107   +5.97699
H   +1.12694   −3.01055   +4.71062
H   +1.15866   −2.26268   +6.28267
C   −1.50826   −4.01775   +4.93241
H   −0.00264   −4.37240   +6.40982
H   −1.08297   −3.05985   +6.79632
H   −2.17871   −4.76695   +5.34771
H   −2.12454   −3.20747   +4.54711
H   −0.98846   −4.46813   +4.08774
24
 * E =   +2.766  kcal/mol  ;  (199)  177_298_181_097_301_184
H   +0.00000   +0.00000   +0.00000
O   +0.00000   +0.00000   +0.95328
C   −0.89741   +0.00000   +1.27482
C   −0.85996   −0.06766   +2.77991
H   −1.41987   +0.90503   +0.95613
H   −1.44607   −0.86070   +0.88079
C   −0.19957   −1.33230   +3.30061
H   −0.31440   +0.80151   +3.14958
H   −1.87968   +0.02066   +3.15360
C   −0.16879   −1.43417   +4.82439
H   −0.71239   −2.20536   +2.88732
H   +0.81143   −1.36241   +2.90072
C   −1.29287   −2.25912   +5.43171
H   +0.77664   −1.87374   +5.13976
H   −0.18682   −0.42952   +5.25318
C   −2.69440   −1.76694   +5.12408
H   −1.20024   −3.29192   +5.08705
H   −1.16186   −2.29114   +6.51506
C   −3.76175   −2.56950   +5.84093
H   −2.77717   −0.71417   +5.40163
H   −2.87043   −1.81502   +4.04876
H   −4.76124   −2.21256   +5.60324
H   −3.71110   −3.62127   +5.56362
H   −3.63639   −2.50990   +6.92081
```

```
24
 * E =   +2.768  kcal/mol  ;  (200)  179_298_180_182_098_301
 H    +0.00000    +0.00000    +0.00000
 O    +0.00000    +0.00000    +0.95330
 C    −0.89727    +0.00000    +1.27530
 C    −0.85612    −0.02822    +2.78153
 H    −1.42840    +0.89167    +0.93425
 H    −1.43811    −0.87564    +0.90435
 C    −0.19813    −1.27546    +3.33672
 H    −0.32054    +0.85683    +3.12748
 H    −1.87777    +0.05456    +3.15631
 C    −0.15756    −1.29464    +4.85464
 H    −0.74309    −2.15575    +2.98689
 H    +0.80582    −1.35681    +2.92531
 C    +0.55305    −2.51144    +5.44569
 H    +0.32594    −0.38296    +5.21545
 H    −1.18274    −1.25063    +5.22495
 C    +2.00348    −2.26836    +5.83438
 H    +0.01957    −2.85240    +6.33240
 H    +0.50493    −3.33672    +4.73147
 C    +2.89593    −1.82336    +4.69279
 H    +2.03238    −1.51668    +6.62543
 H    +2.40697    −3.18208    +6.27160
 H    +3.92838    −1.72160    +5.01992
 H    +2.87616    −2.54124    +3.87392
 H    +2.58484    −0.86048    +4.29262
24
 * E =   +2.786  kcal/mol  ;  (201)  178_297_183_300_099_183
 H    +0.00000    +0.00000    +0.00000
 O    +0.00000    +0.00000    +0.95328
 C    −0.89729    +0.00000    +1.27517
 C    −0.85535    −0.04777    +2.78136
 H    −1.42412    +0.89853    +0.94557
 H    −1.44220    −0.86852    +0.89347
 C    −0.20791    −1.31282    +3.30997
 H    −0.31036    +0.82899    +3.13159
 H    −1.87616    +0.03858    +3.15863
 C    −0.19872    −1.43503    +4.82567
 H    −0.72679    −2.17595    +2.88770
 H    +0.81459    −1.35878    +2.93620
 C    +0.53779    −0.30856    +5.54955
 H    −1.22645    −1.48581    +5.19373
 H    +0.25561    −2.39176    +5.08025
 C    −0.36993    +0.78296    +6.09063
 H    +1.10816    −0.71634    +6.38499
```

```
H    +1.27582    +0.13405    +4.87587
C    +0.39746    +1.91179    +6.74792
H    −0.99214    +1.18060    +5.28892
H    −1.05873    +0.33943    +6.81156
H    −0.26960    +2.67182    +7.14835
H    +1.00915    +1.54186    +7.56944
H    +1.06435    +2.39715    +6.03714
24
* E =  +2.803  kcal/mol ; (202) 182_301_300_097_074_182
H    +0.00000    +0.00000    +0.00000
O    +0.00000    +0.00000    +0.95351
C    −0.89783    +0.00000    +1.27458
C    −0.86344    +0.06151    +2.78036
H    −1.44576    +0.86157    +0.88659
H    −1.42437    −0.90140    +0.94737
C    −0.12848    −1.09552    +3.43993
H    −0.39887    +1.00251    +3.07386
H    −1.89427    +0.09059    +3.13783
C    −0.72235    −2.46893    +3.12925
H    +0.91610    −1.06540    +3.13230
H    −0.13464    −0.92211    +4.51506
C    −0.02277    −3.21219    +1.99902
H    −0.68844    −3.09651    +4.02094
H    −1.78221    −2.35367    +2.89177
C    +1.32705    −3.77904    +2.39920
H    −0.65670    −4.03118    +1.65397
H    +0.11754    −2.54224    +1.14988
C    +2.02969    −4.48227    +1.25565
H    +1.96326    −2.97773    +2.77578
H    +1.18922    −4.47430    +3.22937
H    +2.98975    −4.88998    +1.56396
H    +1.42812    −5.30513    +0.87210
H    +2.21169    −3.79586    +0.43040
24
* E =  +2.816  kcal/mol ; (203) 181_062_182_184_301_096
H    +0.00000    +0.00000    +0.00000
O    +0.00000    +0.00000    +0.95330
C    −0.89731    +0.00000    +1.27519
C    −0.85686    +0.02325    +2.78156
H    −1.43695    +0.87752    +0.90692
H    −1.42944    −0.88986    +0.93112
C    −0.19693    +1.26814    +3.34119
H    −1.87865    −0.05902    +3.15548
H    −0.32220    −0.86315    +3.12541
C    −0.12288    +1.25565    +4.85564
```

```
H    +0.80201    +1.34892    +2.91564
H    −0.75017    +2.15087    +3.00943
C    +0.43407    +2.52322    +5.48499
H    −1.12033    +1.07188    +5.26042
H    +0.49040    +0.40781    +5.16983
C    +1.85026    +2.88920    +5.04232
H    −0.23598    +3.35887    +5.26972
H    +0.41274    +2.39515    +6.56688
C    +1.90279    +3.89415    +3.90578
H    +2.39939    +3.30102    +5.88843
H    +2.38334    +1.98057    +4.75555
H    +2.92870    +4.10879    +3.61377
H    +1.37615    +3.53808    +3.02403
H    +1.44144    +4.83419    +4.20476
24
 * E =   +2.824  kcal/mol ; (204) 063_057_059_263_285_179
H    +0.00000    +0.00000    +0.00000
O    +0.00000    +0.00000    +0.95448
C    −0.90127    +0.00000    +1.26873
C    −1.66856    −1.24174    +0.87182
H    −0.83190    +0.07160    +2.35190
H    −1.42937    +0.89191    +0.92350
C    −1.03400    −2.54690    +1.32743
H    −2.67547    −1.16078    +1.28657
H    −1.78868    −1.25454    −0.21419
C    −0.86146    −2.65578    +2.84196
H    −1.65270    −3.36504    +0.96149
H    −0.06488    −2.65867    +0.84126
C    +0.52657    −2.27763    +3.34279
H    −1.61189    −2.03073    +3.33083
H    −1.07460    −3.67587    +3.16440
C    +1.57604    −3.33537    +3.05338
H    +0.84249    −1.33778    +2.88927
H    +0.48946    −2.11100    +4.42087
C    +2.95792    −2.93176    +3.52633
H    +1.28152    −4.27134    +3.53198
H    +1.60688    −3.53876    +1.98236
H    +3.69502    −3.70431    +3.31894
H    +3.28532    −2.01809    +3.03364
H    +2.96553    −2.74671    +4.59947
24
 * E =   +2.828  kcal/mol ; (205) 183_309_303_178_262_058
H    +0.00000    +0.00000    +0.00000
O    +0.00000    +0.00000    +0.95347
C    −0.89784    +0.00000    +1.27438
```

C	−0.86040	+0.06860	+2.78247
H	−1.45110	+0.85522	+0.88039
H	−1.41593	−0.90607	+0.95111
C	+0.05798	−0.96594	+3.41077
H	−0.53390	+1.06408	+3.08127
H	−1.88064	−0.05430	+3.14981
C	−0.26967	−2.40080	+3.03764
H	+1.08271	−0.73888	+3.12492
H	+0.01027	−0.86391	+4.49590
C	+0.69708	−3.42293	+3.63415
H	−1.29045	−2.61978	+3.35558
H	−0.26046	−2.50548	+1.95047
C	+1.79130	−3.86903	+2.67598
H	+1.15313	−3.00552	+4.53495
H	+0.14756	−4.30617	+3.95827
C	+2.65883	−2.74211	+2.15008
H	+2.41800	−4.60884	+3.17494
H	+1.32641	−4.38523	+1.83365
H	+3.44137	−3.12430	+1.49778
H	+2.07638	−2.01846	+1.58187
H	+3.14271	−2.20693	+2.96653

24

* E = +2.840 kcal/mol ; (206) 181_062_180_175_059_264

H	+0.00000	+0.00000	+0.00000
O	+0.00000	+0.00000	+0.95330
C	−0.89734	+0.00000	+1.27511
C	−0.85705	+0.02511	+2.78132
H	−1.43730	+0.87691	+0.90583
H	−1.42918	−0.89043	+0.93193
C	−0.19320	+1.26874	+3.33935
H	−1.87975	−0.05134	+3.15438
H	−0.32719	−0.86316	+3.12742
C	−0.16121	+1.27959	+4.85539
H	+0.81999	+1.33627	+2.94327
H	−0.72926	+2.14779	+2.97527
C	+0.58715	+2.44857	+5.47701
H	−1.18706	+1.27724	+5.23269
H	+0.28993	+0.34910	+5.20495
C	+0.04614	+3.82774	+5.10172
H	+0.55534	+2.32727	+6.55939
H	+1.64196	+2.38988	+5.19980
C	+0.77848	+4.48418	+3.94521
H	−1.01732	+3.74553	+4.86709
H	+0.10783	+4.48957	+5.96495
H	+0.34294	+5.44977	+3.69614

```
H    +1.82381    +4.65049    +4.20048
H    +0.75837    +3.86902    +3.04912
24
 * E =   +2.849  kcal/mol ; (207) 295_299_174_059_262_178
H    +0.00000    +0.00000    +0.00000
O    +0.00000    +0.00000    +0.95452
C    -0.90246    +0.00000    +1.26544
C    -1.65383    +1.26099    +0.89674
H    -1.43601    -0.87953    +0.89759
H    -0.83547    -0.09102    +2.34758
C    -1.03052    +2.50988    +1.48870
H    -1.69559    +1.34752    -0.19323
H    -2.68672    +1.15411    +1.23274
C    -1.66560    +3.81601    +1.03953
H    -1.08784    +2.44432    +2.57781
H    +0.03032    +2.51791    +1.24177
C    -3.15161    +3.95071    +1.36797
H    -1.11319    +4.63213    +1.50338
H    -1.52395    +3.93559    -0.03731
C    -4.08143    +3.59963    +0.21898
H    -3.39118    +3.32511    +2.23212
H    -3.37070    +4.97356    +1.67656
C    -5.54509    +3.69337    +0.59877
H    -3.87554    +4.27280    -0.61476
H    -3.86112    +2.59663    -0.14600
H    -6.19424    +3.46065    -0.24219
H    -5.78590    +3.00149    +1.40441
H    -5.79599    +4.69541    +0.94332
24
 * E =   +2.863  kcal/mol ; (208) 177_175_059_264_177_179
H    +0.00000    +0.00000    +0.00000
O    +0.00000    +0.00000    +0.95351
C    -0.89806    +0.00000    +1.27392
C    -0.84746    -0.06814    +2.77815
H    -1.42326    +0.90771    +0.96031
H    -1.44683    -0.85695    +0.87499
C    -2.20004    +0.04356    +3.46271
H    -0.36735    -1.00558    +3.06044
H    -0.19342    +0.73160    +3.12382
C    -3.21015    -1.03393    +3.07273
H    -2.02614    +0.00815    +4.53684
H    -2.63047    +1.02775    +3.26402
C    -4.16807    -0.62193    +1.96834
H    -2.67733    -1.94073    +2.77505
H    -3.80304    -1.31308    +3.94425
```

```
C   -5.11788   -1.72928   +1.55541
H   -4.74624   +0.24055   +2.30759
H   -3.61351   -0.28055   +1.09249
C   -6.09429   -1.30092   +0.47868
H   -4.53936   -2.58604   +1.20606
H   -5.66770   -2.07454   +2.43249
H   -6.76329   -2.11121   +0.19851
H   -6.70767   -0.46703   +0.81593
H   -5.57016   -0.97921   -0.41987
24
* E =  +2.874 kcal/mol ; (209) 059_181_300_096_183_181
H   +0.00000   +0.00000   +0.00000
O   +0.00000   +0.00000   +0.95450
C   -0.90235   +0.00000   +1.26567
C   -1.68613   -1.19346   +0.76872
H   -0.83228   -0.01437   +2.35033
H   -1.41069   +0.92809   +0.98530
C   -3.13958   -1.22664   +1.21575
H   -1.65140   -1.20202   -0.32340
H   -1.17674   -2.09855   +1.10123
C   -3.33917   -1.25220   +2.73019
H   -3.67019   -0.36819   +0.79771
H   -3.60135   -2.10635   +0.77056
C   -3.59890   +0.11062   +3.34844
H   -4.18081   -1.89969   +2.97729
H   -2.46650   -1.70838   +3.20440
C   -3.72988   +0.06708   +4.85832
H   -2.80428   +0.80782   +3.07772
H   -4.51480   +0.52305   +2.91907
C   -4.02463   +1.42376   +5.46547
H   -4.51983   -0.63547   +5.12905
H   -2.80966   -0.33376   +5.28620
H   -4.11209   +1.36775   +6.54796
H   -3.23452   +2.13609   +5.23390
H   -4.95719   +1.83134   +5.07867
24
* E =  +2.895 kcal/mol ; (210) 062_055_088_295_305_302
H   +0.00000   +0.00000   +0.00000
O   +0.00000   +0.00000   +0.95473
C   -0.90146   +0.00000   +1.26918
C   -1.67449   -1.23138   +0.83759
H   -0.82689   +0.06333   +2.34989
H   -1.42581   +0.89723   +0.93131
C   -1.04586   -2.56635   +1.22014
H   -2.68852   -1.16278   +1.23786
```

```
H   −1.78242   −1.18684   −0.24875
C   −1.42823   −3.10744   +2.59026
H   −1.34411   −3.30997   +0.48176
H   +0.03846   −2.48084   +1.13632
C   −0.97173   −2.28717   +3.78810
H   −2.51332   −3.21823   +2.63470
H   −1.02510   −4.11668   +2.68087
C   +0.52233   −2.00392   +3.83269
H   −1.52142   −1.34565   +3.82118
H   −1.25640   −2.82308   +4.69570
C   +1.37365   −3.25909   +3.84337
H   +0.81231   −1.38257   +2.98466
H   +0.73682   −1.41616   +4.72539
H   +2.42651   −3.01783   +3.96880
H   +1.08570   −3.92467   +4.65663
H   +1.27782   −3.81616   +2.91311
24
 * E =   +2.907  kcal/mol ;  (211)  180_179_176_059_263_178
H   +0.00000   +0.00000   +0.00000
O   +0.00000   +0.00000   +0.95347
C   −0.89731   +0.00000   +1.27586
C   −0.85629   +0.00658   +2.78148
H   −1.43846   +0.88073   +0.91817
H   −1.43584   −0.88469   +0.92454
C   −2.24178   +0.03678   +3.39623
H   −0.31179   −0.87648   +3.11802
H   −0.27797   +0.87358   +3.09954
C   −2.26305   −0.05707   +4.91414
H   −2.74694   +0.95730   +3.09453
H   −2.83486   −0.78286   +2.98603
C   −1.50197   +1.05667   +5.63111
H   −3.30414   −0.05466   +5.23448
H   −1.85784   −1.02393   +5.22069
C   −0.09064   +0.68098   +6.04992
H   −1.46624   +1.94286   +4.99184
H   −2.04890   +1.36033   +6.52454
C   +0.64783   +1.82684   +6.71080
H   −0.14442   −0.16498   +6.73711
H   +0.47429   +0.33046   +5.18680
H   +1.64701   +1.53271   +7.02341
H   +0.74957   +2.67087   +6.03047
H   +0.11481   +2.17958   +7.59258
24
 * E =   +2.916  kcal/mol ;  (212)  065_062_186_098_301_184
H   +0.00000   +0.00000   +0.00000
```

```
O   +0.00000   +0.00000   +0.95452
C   −0.90240   +0.00000   +1.26563
C   −1.65271   −1.25830   +0.89021
H   −0.83460   +0.08660   +2.34816
H   −1.43454   +0.88207   +0.90177
C   −1.04765   −2.51361   +1.49364
H   −2.68898   −1.15710   +1.21848
H   −1.69190   −1.34048   −0.19812
C   −1.69140   −3.81024   +1.00547
H   +0.02296   −2.52799   +1.28493
H   −1.13773   −2.44006   +2.57788
C   −0.93597   −4.50719   −0.11545
H   −1.77814   −4.50955   +1.83605
H   −2.71454   −3.60722   +0.67990
C   −0.74840   −3.68344   −1.37538
H   +0.04648   −4.80873   +0.25510
H   −1.45756   −5.43083   −0.37342
C   −0.06599   −4.46243   −2.48191
H   −1.72021   −3.32612   −1.72236
H   −0.15854   −2.79553   −1.14349
H   +0.06911   −3.85774   −3.37576
H   +0.91623   −4.80961   −2.16553
H   −0.64906   −5.33889   −2.75972
24
 * E =  +2.918 kcal/mol ; (213) 068_300_093_075_181_181
H   +0.00000   +0.00000   +0.00000
O   +0.00000   +0.00000   +0.95437
C   −0.90226   +0.00000   +1.26542
C   −1.65103   −1.28093   +0.96388
H   −0.84285   +0.15515   +2.33947
H   −1.44780   +0.84991   +0.84403
C   −1.74802   −1.59386   −0.52778
H   −1.16887   −2.10505   +1.48938
H   −2.64836   −1.18106   +1.39277
C   −0.63966   −2.49530   −1.05873
H   −2.70480   −2.06982   −0.74422
H   −1.76106   −0.65399   −1.08678
C   −0.80079   −3.94859   −0.65271
H   −0.61963   −2.43882   −2.14825
H   +0.33073   −2.13903   −0.70972
C   +0.32042   −4.83732   −1.15539
H   −0.85605   −4.02746   +0.43464
H   −1.75536   −4.32160   −1.03164
C   +0.14339   −6.29003   −0.76258
H   +0.38259   −4.75674   −2.24191
```

```
 H    +1.27093    -4.46638    -0.76933
 H    +0.95852    -6.90737    -1.13284
 H    +0.10977    -6.40116    +0.31995
 H    -0.78546    -6.69352    -1.16247
24
 * E =   +2.921  kcal/mol  ;  (214) 069_300_092_183_185_296
 H    +0.00000    +0.00000    +0.00000
 O    +0.00000    +0.00000    +0.95419
 C    -0.90184    +0.00000    +1.26591
 C    -1.64213    -1.29484    +1.00499
 H    -0.84355    +0.18669    +2.33504
 H    -1.45571    +0.83229    +0.82118
 C    -1.73919    -1.66923    -0.47083
 H    -1.15796    -2.10143    +1.55650
 H    -2.64073    -1.18290    +1.42842
 C    -0.61440    -2.56615    -0.96221
 H    -2.68525    -2.17682    -0.65993
 H    -1.77531    -0.75834    -1.07752
 C    -0.69806    -2.85276    -2.44940
 H    +0.34971    -2.12442    -0.71302
 H    -0.65434    -3.50862    -0.41114
 C    +0.33681    -3.84466    -2.95437
 H    -1.69569    -3.23196    -2.67834
 H    -0.59756    -1.91629    -3.00415
 C    +1.76866    -3.36503    -2.81219
 H    +0.21477    -4.79004    -2.42305
 H    +0.13470    -4.05908    -4.00367
 H    +2.46632    -4.07330    -3.25299
 H    +1.91039    -2.40826    -3.31366
 H    +2.05187    -3.23648    -1.77004
24
 * E =   +2.928  kcal/mol  ;  (215) 061_177_175_059_263_178
 H    +0.00000    +0.00000    +0.00000
 O    +0.00000    +0.00000    +0.95436
 C    -0.90209    +0.00000    +1.26587
 C    -1.68037    -1.21972    +0.82680
 H    -0.83735    +0.02929    +2.35123
 H    -1.42172    +0.90945    +0.95017
 C    -3.08470    -1.24440    +1.39896
 H    -1.73085    -1.24197    -0.26561
 H    -1.12598    -2.10628    +1.13364
 C    -3.95452    -2.39081    +0.90674
 H    -3.02130    -1.29318    +2.48824
 H    -3.58224    -0.30087    +1.16688
 C    -3.40059    -3.78331    +1.20302
```

```
H   −4.93778   −2.28298   +1.36270
H   −4.11156   −2.28800   −0.16939
C   −2.64596   −4.41630   +0.04621
H   −2.74819   −3.73670   +2.07892
H   −4.21613   −4.45270   +1.47900
C   −2.07172   −5.77413   +0.39500
H   −3.32526   −4.51276   −0.80234
H   −1.84579   −3.75443   −0.28419
H   −1.55465   −6.22056   −0.45114
H   −1.35984   −5.69907   +1.21540
H   −2.85638   −6.46275   +0.70470
24
 * E =   +2.930  kcal/mol  ;  (216)  302_067_235_070_182_181
H   +0.00000   +0.00000   +0.00000
O   +0.00000   +0.00000   +0.95547
C   −0.90267   +0.00000   +1.26869
C   −1.69533   +1.18510   +0.76361
H   −1.40524   −0.93230   +0.99317
H   −0.83403   +0.02404   +2.35282
C   −1.93809   +1.16344   −0.74962
H   −2.64621   +1.21517   +1.29441
H   −1.15715   +2.09082   +1.04498
C   −1.46393   +2.41448   −1.47136
H   −1.44085   +0.29709   −1.19833
H   −2.99785   +1.01305   −0.94734
C   +0.04652   +2.54606   −1.51721
H   −1.85095   +2.41603   −2.49122
H   −1.89143   +3.29255   −0.98227
C   +0.52124   +3.82133   −2.18670
H   +0.45726   +2.49478   −0.50693
H   +0.45979   +1.68742   −2.05571
C   +2.03142   +3.92518   −2.25422
H   +0.10389   +3.87644   −3.19341
H   +0.11876   +4.67867   −1.64506
H   +2.34817   +4.84883   −2.73289
H   +2.47014   +3.89990   −1.25834
H   +2.45567   +3.09720   −2.81978
24
 * E =   +2.944  kcal/mol  ;  (217)  178_058_088_295_306_301
H   +0.00000   +0.00000   +0.00000
O   +0.00000   +0.00000   +0.95343
C   −0.89744   +0.00000   +1.27535
C   −0.85221   −0.05315   +2.78476
H   −1.42593   +0.88838   +0.92651
H   −1.43804   −0.87364   +0.90356
```

C −0.08812 +1.08087 +3.45925
H −1.87672 −0.09254 +3.16002
H −0.38743 −1.00176 +3.05140
C −0.89678 +2.33057 +3.77626
H +0.31010 +0.70532 +4.40119
H +0.78122 +1.32909 +2.85003
C −1.43508 +3.10617 +2.58257
H −1.73483 +2.05444 +4.41911
H −0.27363 +2.99564 +4.37539
C −0.38125 +3.51111 +1.56320
H −2.21464 +2.52581 +2.08754
H −1.93056 +4.00559 +2.95358
C +0.71906 +4.37783 +2.14488
H +0.06531 +2.62082 +1.11853
H −0.87104 +4.04765 +0.75008
H +1.40044 +4.72198 +1.37032
H +0.30754 +5.25689 +2.63984
H +1.31029 +3.83323 +2.87853
24
* E = +2.948 kcal/mol ; (218) 061_057_059_263_170_061
H +0.00000 +0.00000 +0.00000
O +0.00000 +0.00000 +0.95457
C −0.90144 +0.00000 +1.26859
C −1.68607 −1.21492 +0.82588
H −0.83184 +0.03037 +2.35395
H −1.41758 +0.91096 +0.95640
C −1.08224 −2.54758 +1.24203
H −2.69467 −1.12918 +1.23527
H −1.79831 −1.18927 −0.26086
C −0.91560 −2.71342 +2.75135
H −1.71824 −3.33848 +0.84688
H −0.11093 −2.66735 +0.76061
C +0.48449 −2.38316 +3.24564
H −1.64289 −2.08199 +3.27106
H −1.16073 −3.73640 +3.03347
C +0.60869 −2.31939 +4.75818
H +1.17445 −3.13743 +2.85958
H +0.81141 −1.43936 +2.81162
C +0.27838 −3.62316 +5.45976
H +1.62522 −2.02185 +5.01510
H −0.04209 −1.52891 +5.13763
H +0.45273 −3.55102 +6.53096
H −0.76280 −3.90511 +5.31826
H +0.89444 −4.43693 +5.07866
24

∗ E = +2.956 kcal/mol ; (219) 176_060_060_263_170_061

H	+0.00000	+0.00000	+0.00000
O	+0.00000	+0.00000	+0.95348
C	−0.89733	+0.00000	+1.27585
C	−0.86151	−0.10122	+2.77929
H	−1.41604	+0.91481	+0.97318
H	−1.45431	−0.84587	+0.86684
C	−0.12579	+1.03511	+3.47302
H	−1.89212	−0.13840	+3.13647
H	−0.39859	−1.05089	+3.04631
C	−0.71052	+2.42019	+3.20292
H	−0.13643	+0.82968	+4.54251
H	+0.92022	+1.01681	+3.16706
C	+0.00799	+3.17561	+2.09599
H	−1.77117	+2.32757	+2.94913
H	−0.67784	+3.01354	+4.11574
C	−0.69431	+4.44798	+1.65416
H	+1.01563	+3.42036	+2.43992
H	+0.14557	+2.51633	+1.23989
C	−0.84392	+5.48180	+2.75412
H	−0.14050	+4.88689	+0.82406
H	−1.68051	+4.19423	+1.25973
H	−1.28170	+6.40239	+2.37477
H	−1.48301	+5.12460	+3.55865
H	+0.12389	+5.73032	+3.18821

24

∗ E = +2.958 kcal/mol ; (220) 060_178_184_301_098_182

H	+0.00000	+0.00000	+0.00000
O	+0.00000	+0.00000	+0.95439
C	−0.90217	+0.00000	+1.26577
C	−1.68657	−1.20851	+0.80729
H	−0.83717	+0.01319	+2.35130
H	−1.41733	+0.91663	+0.96321
C	−3.10560	−1.21260	+1.34250
H	−1.70509	−1.22308	−0.28526
H	−1.15878	−2.10888	+1.12430
C	−3.91653	−2.44997	+0.98959
H	−3.07523	−1.11007	+2.42864
H	−3.63028	−0.32997	+0.96961
C	−4.07480	−2.70082	−0.50899
H	−3.45800	−3.32563	+1.45405
H	−4.89828	−2.34789	+1.45002
C	−3.09160	−3.70775	−1.08154
H	−5.08342	−3.05870	−0.71846
H	−3.98085	−1.75448	−1.04871

```
C    −3.24175    −3.88748    −2.57829
H    −2.07046    −3.40697    −0.84808
H    −3.24212    −4.66543    −0.58088
H    −2.54542    −4.62755    −2.96560
H    −4.24851    −4.21429    −2.83401
H    −3.05852    −2.95261    −3.10589
24
* E =  +2.960  kcal/mol ; (221) 291_060_268_177_182_296
H    +0.00000    +0.00000    +0.00000
O    +0.00000    +0.00000    +0.95415
C    −0.90198    +0.00000    +1.26532
C    −1.64115    +1.29522    +1.00393
H    −1.45573    −0.83206    +0.82001
H    −0.84446    −0.18690    +2.33444
C    −1.73607    +1.66946    −0.47226
H    −2.64028    +1.18407    +1.42627
H    −1.15686    +2.10131    +1.55600
C    −0.61361    +2.57016    −0.96208
H    −1.77006    +0.75856    −1.07902
H    −2.68267    +2.17570    −0.66246
C    −0.70551    +2.86967    −2.44624
H    −0.64852    +3.49868    −0.39113
H    +0.35710    +2.12549    −0.73631
C    +0.43049    +3.72408    −2.98476
H    −0.73104    +1.92688    −2.99617
H    −1.65730    +3.36473    −2.65408
C    +0.48668    +5.11866    −2.39112
H    +1.37858    +3.21408    −2.80564
H    +0.32816    +3.80118    −4.06708
H    +1.26434    +5.71521    −2.86235
H    −0.45968    +5.63978    −2.53077
H    +0.69688    +5.09637    −1.32435
24
* E =  +2.966  kcal/mol ; (222) 063_301_301_091_172_064
H    +0.00000    +0.00000    +0.00000
O    +0.00000    +0.00000    +0.95515
C    −0.90277    +0.00000    +1.26711
C    −1.67464    −1.23435    +0.85555
H    −0.83845    +0.05010    +2.35074
H    −1.42716    +0.90189    +0.93841
C    −1.77729    −1.46228    −0.64625
H    −1.20612    −2.10457    +1.31434
H    −2.68019    −1.15765    +1.27351
C    −2.43710    −0.33653    −1.43923
H    −0.78533    −1.66491    −1.05799
```

```
H   −2.33902   −2.38292   −0.79710
C   −1.47766   +0.71566   −1.97532
H   −2.96278   −0.76503   −2.29400
H   −3.20430   +0.13461   −0.82221
C   −2.12510   +1.71597   −2.91961
H   −1.01326   +1.26846   −1.15618
H   −0.66991   +0.20632   −2.50602
C   −3.18605   +2.57933   −2.26450
H   −1.34931   +2.35769   −3.33650
H   −2.56102   +1.17819   −3.76325
H   −3.56837   +3.32502   −2.95751
H   −4.03334   +1.98946   −1.92240
H   −2.78100   +3.10744   −1.40223
24
* E =  +2.966  kcal/mol ; (223) 066_296_183_183_098_301
H   +0.00000   +0.00000   +0.00000
O   +0.00000   +0.00000   +0.95395
C   −0.90085   +0.00000   +1.26779
C   −1.64805   −1.27246   +0.93319
H   −0.83409   +0.12257   +2.34529
H   −1.44374   +0.86523   +0.87510
C   −1.82088   −1.50196   −0.55569
H   −1.11838   −2.11646   +1.37634
H   −2.62972   −1.22898   +1.40914
C   −2.64227   −2.73932   −0.87552
H   −2.30754   −0.63145   −1.00362
H   −0.84359   −1.58742   −1.03314
C   −2.76858   −3.03898   −2.36834
H   −2.20617   −3.60259   −0.36670
H   −3.63264   −2.60845   −0.43866
C   −1.79346   −4.08501   −2.88679
H   −3.77882   −3.38217   −2.58779
H   −2.63875   −2.11283   −2.93328
C   −0.33129   −3.73511   −2.69351
H   −2.00231   −5.03453   −2.39086
H   −1.98667   −4.24925   −3.94698
H   +0.31425   −4.49236   −3.13245
H   −0.08902   −2.78204   −3.16250
H   −0.07316   −3.66156   −1.63864
24
* E =  +2.968  kcal/mol ; (224) 297_300_176_177_262_059
H   +0.00000   +0.00000   +0.00000
O   +0.00000   +0.00000   +0.95456
C   −0.90236   +0.00000   +1.26591
C   −1.66385   +1.24526   +0.86790
```

```
H    −1.42918   −0.89208   +0.91883
H    −0.83375   −0.06553   +2.34966
C    −1.05499   +2.52123   +1.41311
H    −1.71546   +1.29942   −0.22357
H    −2.69526   +1.14573   +1.21194
C    −1.76401   +3.77258   +0.92569
H    −1.06404   +2.48405   +2.50319
H    −0.00488   +2.56565   +1.12407
C    −1.21092   +5.07199   +1.50931
H    −1.69373   +3.79876   −0.16248
H    −2.83174   +3.70007   +1.15033
C    −1.99386   +5.61540   +2.69483
H    −0.17049   +4.91786   +1.80401
H    −1.19197   +5.84107   +0.73797
C    −2.08186   +4.67172   +3.87783
H    −1.53842   +6.55239   +3.01592
H    −3.00260   +5.86784   +2.36292
H    −2.60084   +5.13704   +4.71284
H    −2.62462   +3.76299   +3.62392
H    −1.09110   +4.37970   +4.22353
24
 * E =   +2.974 kcal/mol ; (225) 298_303_302_097_187_069
H    +0.00000   +0.00000   +0.00000
O    +0.00000   +0.00000   +0.95455
C    −0.90146   +0.00000   +1.26846
C    −1.68146   +1.22230   +0.83750
H    −1.42091   −0.90602   +0.94751
H    −0.83246   −0.04127   +2.35383
C    −1.06949   +2.54786   +1.26487
H    −1.79707   +1.20680   −0.24903
H    −2.68913   +1.13764   +1.24945
C    −0.88184   +2.68622   +2.77418
H    −0.10539   +2.67473   +0.77102
H    −1.71052   +3.34593   +0.89290
C    +0.52601   +2.35293   +3.24369
H    −1.11271   +3.70578   +3.08513
H    −1.61027   +2.05163   +3.28373
C    +0.68534   +2.32982   +4.75461
H    +0.84298   +1.39688   +2.82593
H    +1.20569   +3.09504   +2.82184
C    −0.02799   +1.17066   +5.42563
H    +1.74679   +2.27698   +4.99604
H    +0.32624   +3.27236   +5.17273
H    +0.15312   +1.16166   +6.49817
H    −1.10533   +1.21660   +5.28076
```

```
 H    +0.31915    +0.21927    +5.02431
24
 * E =  +2.980  kcal/mol ; (226) 065_060_181_264_059_176
 H    +0.00000    +0.00000    +0.00000
 O    +0.00000    +0.00000    +0.95448
 C    -0.90215    +0.00000    +1.26619
 C    -1.65002    -1.26660    +0.91259
 H    -0.83408    +0.10377    +2.34716
 H    -1.43936    +0.87373    +0.88967
 C    -1.01232    -2.51655    +1.49262
 H    -2.68178    -1.16443    +1.24948
 H    -1.69892    -1.36020    -0.17605
 C    -1.73659    -3.81302    +1.13487
 H    +0.01871    -2.55376    +1.14646
 H    -0.95484    -2.41824    +2.57975
 C    -2.71160    -4.31206    +2.18999
 H    -2.26695    -3.68125    +0.18871
 H    -1.00477    -4.60016    +0.95744
 C    -3.83115    -3.35084    +2.54183
 H    -3.14962    -5.25187    +1.84854
 H    -2.15625    -4.55225    +3.09974
 C    -4.82517    -3.95018    +3.51644
 H    -3.41020    -2.44157    +2.97256
 H    -4.34805    -3.04744    +1.62910
 H    -5.61550    -3.24682    +3.76820
 H    -5.29347    -4.84028    +3.09956
 H    -4.33452    -4.24250    +4.44346
24
 * E =  +2.981  kcal/mol ; (227) 292_060_267_179_292_183
 H    +0.00000    +0.00000    +0.00000
 O    +0.00000    +0.00000    +0.95433
 C    -0.90199    +0.00000    +1.26604
 C    -1.65062    +1.28489    +0.98110
 H    -1.45052    -0.84371    +0.83628
 H    -0.84266    -0.16702    +2.33835
 C    -1.74651    +1.63037    -0.50243
 H    -2.64957    +1.17336    +1.40373
 H    -1.17357    +2.10461    +1.51936
 C    -0.63512    +2.54480    -0.99557
 H    -1.76764    +0.70522    -1.08479
 H    -2.69903    +2.12100    -0.70536
 C    -0.70975    +2.87099    -2.47726
 H    -0.68430    +3.47195    -0.42307
 H    +0.34042    +2.11433    -0.76473
 C    -0.44287    +1.69185    -3.39562
```

```
 H    −1.69234    +3.29005    −2.70751
 H    +0.01306    +3.65645    −2.70335
 C    −0.44857    +2.08062    −4.86051
 H    +0.52372    +1.25298    −3.13919
 H    −1.18703    +0.91283    −3.22955
 H    −0.25445    +1.22553    −5.50369
 H    −1.41100    +2.50005    −5.14887
 H    +0.31155    +2.83172    −5.06858
24
 * E =   +2.984  kcal/mol  ;  (228) 294_064_174_060_263_178
 H    +0.00000    +0.00000    +0.00000
 O    +0.00000    +0.00000    +0.95395
 C    −0.90079    +0.00000    +1.26796
 C    −1.64665    +1.27518    +0.93847
 H    −1.44504    −0.86258    +0.87118
 H    −0.83429    −0.12722    +2.34496
 C    −1.81062    +1.50194    −0.55259
 H    −2.63040    +1.22882    +1.41043
 H    −1.11406    +2.11266    +1.38900
 C    −2.67325    +2.69752    −0.92757
 H    −0.82626    +1.63862    −1.00709
 H    −2.23928    +0.60329    −1.00293
 C    −2.16185    +4.03920    −0.40549
 H    −2.74495    +2.73054    −2.01392
 H    −3.69076    +2.53414    −0.56546
 C    −2.82383    +4.50408    +0.88049
 H    −1.08035    +3.98399    −0.25701
 H    −2.31641    +4.81037    −1.16092
 C    −2.25612    +5.81281    +1.39050
 H    −3.89476    +4.61385    +0.70217
 H    −2.72513    +3.73782    +1.64888
 H    −2.75717    +6.14078    +2.29825
 H    −1.19468    +5.71844    +1.61385
 H    −2.36528    +6.60212    +0.64828
24
 * E =   +2.985  kcal/mol  ;  (229) 071_277_059_059_180_296
 H    +0.00000    +0.00000    +0.00000
 O    +0.00000    +0.00000    +0.95310
 C    −0.89742    +0.00000    +1.27409
 C    −1.63464    −1.30588    +1.04668
 H    −0.81234    +0.18979    +2.34060
 H    −1.46261    +0.83283    +0.84379
 C    −2.25507    −1.47922    −0.33261
 H    −0.94837    −2.12165    +1.27346
 H    −2.43754    −1.37101    +1.78205
```

```
C   -1.30788   -1.41273   -1.52189
H   -2.77222   -2.43962   -0.35878
H   -3.02772   -0.71941   -0.46205
C   -0.19564   -2.44579   -1.49439
H   -1.89954   -1.53990   -2.42818
H   -0.88186   -0.40765   -1.60925
C   +0.74847   -2.37372   -2.68412
H   +0.38376   -2.33369   -0.57821
H   -0.64298   -3.44180   -1.45013
C   +0.09149   -2.67214   -4.01822
H   +1.20927   -1.38426   -2.71925
H   +1.56474   -3.07741   -2.52374
H   +0.82373   -2.68522   -4.82214
H   -0.39759   -3.64528   -4.00142
H   -0.66016   -1.93007   -4.27765
24
* E =  +2.987  kcal/mol ; (230) 064_060_181_177_262_059
H   +0.00000   +0.00000   +0.00000
O   +0.00000   +0.00000   +0.95450
C   -0.90190   +0.00000   +1.26699
C   -1.65747   -1.25551   +0.89071
H   -0.83230   +0.08303   +2.34952
H   -1.43416   +0.88364   +0.90685
C   -1.03775   -2.51933   +1.45165
H   -2.68764   -1.15740   +1.23930
H   -1.71459   -1.32606   -0.19936
C   -1.78897   -3.77748   +1.05296
H   +0.00215   -2.57765   +1.13513
H   -1.01505   -2.45135   +2.54159
C   -1.14913   -5.07140   +1.55451
H   -2.80957   -3.70011   +1.42986
H   -1.87940   -3.81729   -0.03601
C   -0.28025   -5.78389   +0.52921
H   -0.55304   -4.85510   +2.44394
H   -1.92614   -5.76366   +1.87677
C   +0.87810   -4.95947   +0.00363
H   +0.10450   -6.70223   +0.97320
H   -0.90856   -6.09327   -0.30809
H   +1.49362   -5.53968   -0.68021
H   +0.52990   -4.08146   -0.53726
H   +1.51732   -4.61417   +0.81495
24
* E =  +2.987  kcal/mol ; (231) 180_180_181_183_098_301
H   +0.00000   +0.00000   +0.00000
O   +0.00000   +0.00000   +0.95348
```

C	−0.89790	+0.00000	+1.27425
C	−0.86088	−0.00634	+2.77980
H	−1.43535	+0.88499	+0.92192
H	−1.43808	−0.88034	+0.91457
C	−2.24370	−0.00848	+3.39964
H	−0.30047	−0.88214	+3.10759
H	−0.29946	+0.86591	+3.11549
C	−2.21001	+0.01940	+4.91814
H	−2.80442	+0.86030	+3.04731
H	−2.79854	−0.88283	+3.05687
C	−3.58783	−0.04461	+5.57544
H	−1.59492	−0.80757	+5.28177
H	−1.69408	+0.92852	+5.22772
C	−3.99993	−1.43145	+6.04528
H	−3.61450	+0.62380	+6.43519
H	−4.33691	+0.33669	+4.87743
C	−4.05113	−2.47845	+4.95033
H	−3.30568	−1.75928	+6.82102
H	−4.97718	−1.36379	+6.52371
H	−4.40735	−3.43133	+5.33527
H	−4.71978	−2.17478	+4.14569
H	−3.06782	−2.65098	+4.51665

24

∗ E = +2.987 kcal/mol ; (232) 296_311_302_177_262_058

H	+0.00000	+0.00000	+0.00000
O	+0.00000	+0.00000	+0.95466
C	−0.90092	+0.00000	+1.27045
C	−1.66336	+1.25003	+0.87956
H	−1.43357	−0.88861	+0.92417
H	−0.82465	−0.07726	+2.35151
C	−0.91680	+2.53795	+1.18316
H	−1.89501	+1.21295	−0.18716
H	−2.62376	+1.23648	+1.39873
C	−0.53277	+2.70666	+2.64254
H	−0.01921	+2.58187	+0.56837
H	−1.53499	+3.38409	+0.88000
C	+0.27419	+3.97533	+2.91614
H	−1.44315	+2.70190	+3.24409
H	+0.05233	+1.84449	+2.96810
C	+1.77575	+3.74413	+2.99822
H	+0.06368	+4.71242	+2.13749
H	−0.05300	+4.42706	+3.85197
C	+2.38122	+3.12168	+1.75502
H	+2.26841	+4.69405	+3.20766
H	+1.98233	+3.10015	+3.85500

```
 H    +3.45922    +3.01686    +1.85748
 H    +1.97139    +2.13118    +1.56273
 H    +2.19213    +3.73912    +0.87700
24
 * E =   +2.988  kcal/mol  ;  (233) 061_177_178_177_262_059
 H    +0.00000    +0.00000    +0.00000
 O    +0.00000    +0.00000    +0.95439
 C    -0.90205    +0.00000    +1.26610
 C    -1.68446    -1.21296    +0.81669
 H    -0.83623    +0.02006    +2.35148
 H    -1.41860    +0.91393    +0.95832
 C    -3.09511    -1.23935    +1.37111
 H    -1.72403    -1.23133    -0.27588
 H    -1.14292    -2.10842    +1.12263
 C    -3.90114    -2.43385    +0.89028
 H    -3.05634    -1.23107    +2.46090
 H    -3.61839    -0.32497    +1.08256
 C    -5.30639    -2.51928    +1.48418
 H    -3.96197    -2.38514    -0.19736
 H    -3.35504    -3.35345    +1.11503
 C    -5.43397    -3.46070    +2.67217
 H    -5.63263    -1.51942    +1.77991
 H    -6.00789    -2.84694    +0.71791
 C    -4.54298    -3.11537    +3.84884
 H    -6.47361    -3.47229    +2.99986
 H    -5.21043    -4.47612    +2.34022
 H    -4.72208    -3.78600    +4.68616
 H    -3.48897    -3.19572    +3.58926
 H    -4.72285    -2.09810    +4.19448
24
 * E =   +2.990  kcal/mol  ;  (234) 061_177_181_182_098_301
 H    +0.00000    +0.00000    +0.00000
 O    +0.00000    +0.00000    +0.95438
 C    -0.90201    +0.00000    +1.26618
 C    -1.68579    -1.21080    +0.81337
 H    -0.83605    +0.01686    +2.35161
 H    -1.41762    +0.91536    +0.96107
 C    -3.09850    -1.23394    +1.36271
 H    -1.72186    -1.22755    -0.27928
 H    -1.14768    -2.10825    +1.11982
 C    -3.88382    -2.46129    +0.93297
 H    -3.06234    -1.20611    +2.45386
 H    -3.62548    -0.32917    +1.05723
 C    -5.33066    -2.48060    +1.42389
 H    -3.86952    -2.53756    -0.15738
```

```
 H    −3.35751    −3.34377    +1.29724
 C    −6.35128    −2.00583    +0.40077
 H    −5.60109    −3.49178    +1.72534
 H    −5.41217    −1.86852    +2.32511
 C    −6.13468    −0.58998    −0.09543
 H    −6.33825    −2.68893    −0.45038
 H    −7.34744    −2.08533    +0.83627
 H    −6.92617    −0.28810    −0.77763
 H    −6.12046    +0.11961    +0.73095
 H    −5.19154    −0.49433    −0.63047
24
 * E =   +2.992  kcal/mol ;  (235) 181_182_183_097_301_184
 H    +0.00000    +0.00000    +0.00000
 O    +0.00000    +0.00000    +0.95345
 C    −0.89771    +0.00000    +1.27468
 C    −0.85974    +0.02622    +2.77975
 H    −1.44221    +0.87308    +0.90372
 H    −1.43149    −0.89190    +0.93373
 C    −2.24522    −0.02858    +3.39836
 H    −0.27132    −0.82447    +3.12391
 H    −0.32566    +0.91989    +3.09804
 C    −2.24536    +0.07949    +4.92260
 H    −2.86846    +0.76705    +2.98128
 H    −2.71791    −0.96403    +3.09743
 C    −2.51262    +1.47714    +5.45891
 H    −3.00119    −0.58795    +5.33452
 H    −1.28810    −0.27889    +5.30702
 C    −1.52558    +2.53967    +5.01426
 H    −3.51814    +1.78430    +5.16091
 H    −2.52123    +1.43944    +6.54980
 C    −1.79329    +3.88468    +5.65938
 H    −0.51130    +2.21325    +5.25136
 H    −1.56570    +2.64640    +3.92960
 H    −1.08342    +4.63799    +5.32592
 H    −2.79328    +4.24173    +5.41785
 H    −1.72246    +3.81920    +6.74387
24
 * E =   +2.998  kcal/mol ;  (236) 069_301_093_188_298_185
 H    +0.00000    +0.00000    +0.00000
 O    +0.00000    +0.00000    +0.95428
 C    −0.90186    +0.00000    +1.26621
 C    −1.64461    −1.29187    +0.99751
 H    −0.84300    +0.18065    +2.33635
 H    −1.45402    +0.83586    +0.82610
 C    −1.73484    −1.65834    −0.48141
```

H −1.16339 −2.10221 +1.54624
H −2.64397 −1.18014 +1.41893
C −0.61308 −2.57144 −0.95397
H −2.69018 −2.14223 −0.67736
H −1.74379 −0.74436 −1.08570
C −0.57184 −2.78149 −2.45774
H +0.34542 −2.17868 −0.61589
H −0.72198 −3.53831 −0.45772
C −1.81025 −3.44212 −3.03557
H −0.41618 −1.82055 −2.95522
H +0.29831 −3.39167 −2.70532
C −1.67548 −3.73666 −4.51606
H −2.00582 −4.36918 −2.49315
H −2.68026 −2.80575 −2.87390
H −2.57189 −4.20534 −4.91530
H −1.50021 −2.82297 −5.08184
H −0.83807 −4.40559 −4.70750
24
* E = +3.000 kcal/mol ; (237) 183_300_301_098_186_069
H +0.00000 +0.00000 +0.00000
O +0.00000 +0.00000 +0.95349
C −0.89726 +0.00000 +1.27608
C −0.85913 +0.06718 +2.78151
H −1.44741 +0.85938 +0.88631
H −1.42412 −0.90369 +0.95363
C −0.12993 −1.09081 +3.44621
H −0.39033 +1.00745 +3.07055
H −1.88924 +0.10198 +3.14036
C −0.71589 −2.46434 +3.12556
H +0.91877 −1.06428 +3.15051
H −0.15062 −0.92037 +4.52170
C +0.01475 −3.19083 +2.00735
H −0.69009 −3.09654 +4.01408
H −1.77309 −2.35252 +2.87478
C −0.64244 −4.49119 +1.57651
H +0.12306 −2.53063 +1.14597
H +1.03156 −3.39575 +2.34534
C −1.97337 −4.29999 +0.87314
H +0.03506 −5.02560 +0.91056
H −0.77928 −5.13432 +2.44800
H −2.37996 −5.24856 +0.52954
H −2.71670 −3.84630 +1.52493
H −1.86160 −3.65493 +0.00197
24
* E = +3.002 kcal/mol ; (238) 066_296_181_178_262_059

```
H    +0.00000    +0.00000    +0.00000
O    +0.00000    +0.00000    +0.95389
C    -0.90051    +0.00000    +1.26850
C    -1.65195    -1.26899    +0.92986
H    -0.83223    +0.11754    +2.34647
H    -1.44218    +0.86806    +0.88042
C    -1.83677    -1.48809    -0.55919
H    -1.12187    -2.11730    +1.36434
H    -2.63023    -1.22561    +1.41258
C    -2.61866    -2.74989    -0.88283
H    -2.33276    -0.61921    -0.99599
H    -0.86138    -1.56257    -1.04612
C    -2.86681    -2.96809    -2.37460
H    -2.07362    -3.59868    -0.46948
H    -3.57538    -2.72695    -0.35477
C    -4.23727    -2.51928    -2.85878
H    -2.09458    -2.44892    -2.94712
H    -2.75423    -4.02481    -2.61354
C    -4.53625    -1.05094    -2.63039
H    -4.32424    -2.74285    -3.92213
H    -4.99926    -3.12090    -2.36020
H    -5.50385    -0.77839    -3.04554
H    -4.55884    -0.80868    -1.56939
H    -3.78405    -0.41769    -3.09934
24
* E =   +3.005  kcal/mol ;  (239)  060_176_176_263_059_176
H    +0.00000    +0.00000    +0.00000
O    +0.00000    +0.00000    +0.95441
C    -0.90225    +0.00000    +1.26561
C    -1.69119    -1.19994    +0.79454
H    -0.83759    +0.00189    +2.35143
H    -1.41316    +0.92217    +0.97275
C    -3.09270    -1.24303    +1.37846
H    -1.73933    -1.19386    -0.29617
H    -1.14944    -2.10378    +1.07414
C    -3.95214    -2.38998    +0.84819
H    -3.00520    -1.31970    +2.46241
H    -3.60043    -0.29379    +1.18810
C    -4.89174    -2.00637    -0.28421
H    -3.30194    -3.20362    +0.51969
H    -4.55363    -2.79841    +1.65907
C    -4.21702    -1.44669    -1.52220
H    -5.47663    -2.88296    -0.56853
H    -5.61121    -1.27209    +0.08605
C    -5.19885    -1.17359    -2.64402
```

```
H   -3.69563   -0.52273   -1.26820
H   -3.45215   -2.14649   -1.86478
H   -4.70322   -0.76463   -3.52137
H   -5.70977   -2.08597   -2.94694
H   -5.96016   -0.46070   -2.33144
24
* E =  +3.007  kcal/mol ; (240) 061_179_182_097_301_184
H   +0.00000   +0.00000   +0.00000
O   +0.00000   +0.00000   +0.95436
C   -0.90217   +0.00000   +1.26563
C   -1.68297   -1.21462   +0.81873
H   -0.83738   +0.02335   +2.35112
H   -1.41986   +0.91282   +0.95557
C   -3.11200   -1.21086   +1.33332
H   -1.70200   -1.24650   -0.27395
H   -1.15330   -2.10896   +1.14287
C   -3.91513   -2.45353   +0.95143
H   -3.10791   -1.10399   +2.42107
H   -3.61064   -0.31987   +0.95039
C   -3.96429   -3.53093   +2.02334
H   -4.93946   -2.16618   +0.71778
H   -3.50619   -2.87692   +0.03139
C   -2.61622   -4.07952   +2.45065
H   -4.47686   -3.13255   +2.90227
H   -4.58216   -4.35591   +1.66392
C   -2.74267   -5.21582   +3.44549
H   -2.06852   -4.42074   +1.57004
H   -2.01830   -3.28107   +2.89152
H   -1.76908   -5.59257   +3.74983
H   -3.26783   -4.89258   +4.34290
H   -3.30238   -6.04744   +3.02054
24
* E =  +3.009  kcal/mol ; (241) 060_173_059_264_177_178
H   +0.00000   +0.00000   +0.00000
O   +0.00000   +0.00000   +0.95451
C   -0.90219   +0.00000   +1.26618
C   -1.67331   -1.21410   +0.79986
H   -0.83832   +0.01017   +2.35250
H   -1.41049   +0.91858   +0.96224
C   -3.06521   -1.35350   +1.39687
H   -1.75689   -1.18085   -0.29006
H   -1.08151   -2.09710   +1.03930
C   -4.00738   -0.18441   +1.11480
H   -3.49840   -2.27403   +1.00917
H   -2.98232   -1.49602   +2.47650
```

C −4.06817 +0.85054 +2.22479
H −3.71689 +0.30054 +0.17906
H −5.01752 −0.56020 +0.94975
C −4.95978 +2.03134 +1.89376
H −4.43186 +0.36864 +3.13509
H −3.06631 +1.21293 +2.46078
C −5.04529 +3.04172 +3.01980
H −4.58804 +2.51928 +0.99121
H −5.95959 +1.66804 +1.65019
H −5.68814 +3.87911 +2.75888
H −5.44547 +2.58665 +3.92427
H −4.06228 +3.44211 +3.26200
24
* E = +3.009 kcal/mol ; (242) 068_295_178_059_262_178
H +0.00000 +0.00000 +0.00000
O +0.00000 +0.00000 +0.95375
C −0.89974 +0.00000 +1.27014
C −1.64111 −1.28499 +0.96907
H −0.83000 +0.14624 +2.34449
H −1.45073 +0.85255 +0.86129
C −1.83705 −1.53225 −0.51442
H −1.09514 −2.11744 +1.41530
H −2.61185 −1.23560 +1.46479
C −2.53871 −2.83781 −0.85754
H −2.40698 −0.70186 −0.93977
H −0.86606 −1.52972 −1.01384
C −3.93747 −2.98415 −0.26062
H −2.59246 −2.91379 −1.94271
H −1.91836 −3.67455 −0.52961
C −3.98783 −3.80963 +1.01374
H −4.35804 −1.99345 −0.06811
H −4.60272 −3.44819 −0.98949
C −5.37849 −3.88408 +1.60998
H −3.63007 −4.81615 +0.79148
H −3.29387 −3.40249 +1.74883
H −5.39767 −4.49699 +2.50806
H −5.74386 −2.89376 +1.87739
H −6.08554 −4.31269 +0.90135
24
* E = +3.016 kcal/mol ; (243) 067_295_180_263_059_176
H +0.00000 +0.00000 +0.00000
O +0.00000 +0.00000 +0.95383
C −0.90032 +0.00000 +1.26881
C −1.64642 −1.27553 +0.94493
H −0.83188 +0.12946 +2.34542

H −1.44508 +0.86227 +0.87184
C −1.85580 −1.49274 −0.54420
H −1.11001 −2.11574 +1.38327
H −2.62107 −1.23445 +1.43485
C −2.61032 −2.77593 −0.89089
H −2.39669 −0.63076 −0.93866
H −0.89103 −1.50770 −1.05894
C −1.72427 −3.95603 −1.25760
H −3.25121 −3.05184 −0.05070
H −3.28092 −2.58626 −1.72801
C −0.74294 −4.38573 −0.18360
H −2.36011 −4.80530 −1.51437
H −1.16791 −3.71249 −2.16608
C +0.03315 −5.62796 −0.57292
H −0.04366 −3.57471 +0.02172
H −1.28330 −4.56673 +0.74765
H +0.73887 −5.91855 +0.20161
H −0.63551 −6.46966 −0.74596
H +0.59750 −5.46444 −1.48969
24
* E = +3.028 kcal/mol ; (244) 064_061_178_060_261_177
H +0.00000 +0.00000 +0.00000
O +0.00000 +0.00000 +0.95452
C −0.90231 +0.00000 +1.26588
C −1.65547 −1.25784 +0.89043
H −0.83438 +0.08554 +2.34837
H −1.43496 +0.88210 +0.90254
C −1.03062 −2.51330 +1.46613
H −2.68780 −1.16033 +1.23392
H −1.70244 −1.32688 −0.19943
C −1.77839 −3.79980 +1.15315
H −0.00601 −2.59223 +1.10234
H −0.95645 −2.39991 +2.54918
C −1.91915 −4.10774 −0.33705
H −1.25626 −4.61747 +1.64813
H −2.77182 −3.76282 +1.60653
C −3.26256 −3.71829 −0.92997
H −1.11946 −3.60671 −0.88924
H −1.77117 −5.17413 −0.51068
C −3.34404 −3.97677 −2.42045
H −4.04578 −4.27912 −0.41752
H −3.46824 −2.66714 −0.72722
H −4.31988 −3.71296 −2.82155
H −2.59644 −3.39751 −2.96028
H −3.16674 −5.02742 −2.64496

```
24
 * E =   +3.034  kcal/mol  ;  (245)  059_179_263_060_065_180
 H    +0.00000    +0.00000    +0.00000
 O    +0.00000    +0.00000    +0.95457
 C    −0.90194    +0.00000    +1.26715
 C    −1.68998    −1.20038    +0.78608
 H    −0.83289    +0.01253    +2.35115
 H    −1.41691    +0.91829    +0.96823
 C    −3.12782    −1.24025    +1.29993
 H    −1.69064    −1.18164    −0.30559
 H    −1.15756    −2.10759    +1.07538
 C    −3.33525    −2.11410    +2.52975
 H    −3.47196    −0.22326    +1.49925
 H    −3.77996    −1.61657    +0.51348
 C    −2.53794    −1.72078    +3.76194
 H    −4.39707    −2.11722    +2.78237
 H    −3.08436    −3.14190    +2.26309
 C    −2.90998    −0.36904    +4.34267
 H    −2.68583    −2.48116    +4.53049
 H    −1.47001    −1.74143    +3.53338
 C    −2.09298    −0.01776    +5.56965
 H    −2.78248    +0.41062    +3.59084
 H    −3.97203    −0.37130    +4.59417
 H    −2.38060    +0.94686    +5.98111
 H    −2.22092    −0.76404    +6.35203
 H    −1.03126    +0.02732    +5.33165
24
 * E =   +3.037  kcal/mol  ;  (246)  064_060_183_185_301_096
 H    +0.00000    +0.00000    +0.00000
 O    +0.00000    +0.00000    +0.95452
 C    −0.90206    +0.00000    +1.26661
 C    −1.65589    −1.25724    +0.89273
 H    −0.83315    +0.08561    +2.34899
 H    −1.43497    +0.88228    +0.90422
 C    −1.03624    −2.51873    +1.46054
 H    −2.68727    −1.15904    +1.23697
 H    −1.70839    −1.33257    −0.19727
 C    −1.75287    −3.77716    +1.01095
 H    +0.01162    −2.55526    +1.16501
 H    −1.04528    −2.46206    +2.55184
 C    −1.24543    −5.06720    +1.63650
 H    −2.81845    −3.67730    +1.22724
 H    −1.67328    −3.85835    −0.07598
 C    +0.23207    −5.36448    +1.38300
 H    −1.42624    −5.04216    +2.71353
```

H −1.85298 −5.88552 +1.25120
C +1.15511 −4.91392 +2.50080
H +0.36641 −6.43605 +1.23949
H +0.53642 −4.89904 +0.44319
H +2.19538 −5.13068 +2.26729
H +1.07530 −3.84655 +2.69027
H +0.91146 −5.42930 +3.42855
24
* E = +3.037 kcal/mol ; (247) 062_273_055_176_301_305
H +0.00000 +0.00000 +0.00000
O +0.00000 +0.00000 +0.95349
C −0.89821 +0.00000 +1.27343
C −1.69523 −1.22304 +0.85684
H −0.80790 +0.03276 +2.35546
H −1.41953 +0.91299 +0.96667
C −2.37951 −1.12461 −0.49834
H −1.01859 −2.07834 +0.87897
H −2.45872 −1.41446 +1.61124
C −1.46423 −0.77723 −1.65880
H −2.87287 −2.07555 −0.70046
H −3.17507 −0.37763 −0.44617
C −2.15741 −0.76761 −3.01411
H −1.04503 +0.21988 −1.50289
H −0.62277 −1.47375 −1.68084
C −2.81710 −2.07922 −3.41367
H −2.90896 +0.02333 −3.02165
H −1.42388 −0.49739 −3.77531
C −1.87718 −3.26834 −3.38372
H −3.67356 −2.27648 −2.76882
H −3.22428 −1.96471 −4.41793
H −2.36094 −4.16272 −3.76883
H −0.99079 −3.08399 −3.98949
H −1.54234 −3.49033 −2.37185
24
* E = +3.038 kcal/mol ; (248) 293_062_175_263_059_176
H +0.00000 +0.00000 +0.00000
O +0.00000 +0.00000 +0.95390
C −0.90077 +0.00000 +1.26781
C −1.64699 +1.27512 +0.94224
H −1.44492 −0.86195 +0.86931
H −0.83446 −0.13025 +2.34450
C −1.79072 +1.52771 −0.54933
H −2.62969 +1.22566 +1.41135
H −1.12264 +2.11534 +1.39861
C −2.64694 +2.74531 −0.89585

```
 H   −0.79508   +1.66249   −0.97348
 H   −2.21176   +0.64041   −1.03195
 C   −4.09714   +2.42798   −1.22571
 H   −2.61047   +3.45435   −0.06604
 H   −2.21468   +3.26425   −1.75025
 C   −4.87486   +1.73153   −0.12519
 H   −4.60920   +3.35658   −1.48427
 H   −4.12830   +1.80764   −2.12474
 C   −6.33450   +1.53546   −0.48294
 H   −4.42346   +0.76111   +0.08507
 H   −4.79660   +2.31196   +0.79618
 H   −6.87751   +1.02691   +0.31018
 H   −6.82518   +2.49083   −0.66115
 H   −6.43782   +0.94056   −1.38904
24
 * E =  +3.038 kcal/mol ; (249) 181_059_064_062_263_182
 H   +0.00000   +0.00000   +0.00000
 O   +0.00000   +0.00000   +0.95330
 C   −0.89785   +0.00000   +1.27368
 C   −0.85560   +0.02738   +2.78145
 H   −1.43902   +0.86921   +0.89153
 H   −1.42771   −0.89463   +0.93802
 C   −0.12606   +1.23248   +3.35078
 H   −1.87699   −0.01931   +3.16097
 H   −0.35787   −0.88103   +3.11995
 C   −0.75158   +2.58848   +3.05684
 H   −0.05636   +1.11255   +4.43303
 H   +0.89395   +1.22247   +2.97043
 C   −2.15975   +2.78723   +3.61577
 H   −0.09020   +3.34624   +3.47516
 H   −0.75694   +2.77241   +1.98047
 C   −3.28685   +2.52518   +2.63125
 H   −2.29452   +2.15204   +4.49539
 H   −2.26699   +3.81192   +3.97441
 C   −4.65551   +2.77525   +3.23179
 H   −3.14742   +3.16651   +1.75911
 H   −3.23646   +1.50101   +2.26274
 H   −5.44983   +2.59686   +2.51062
 H   −4.83240   +2.12470   +4.08685
 H   −4.74749   +3.80317   +3.57903
24
 * E =  +3.045 kcal/mol ; (250) 296_069_098_300_184_181
 H   +0.00000   +0.00000   +0.00000
 O   +0.00000   +0.00000   +0.95428
 C   −0.90099   +0.00000   +1.26872
```

```
C   -1.65292   +1.25912   +0.88583
H   -1.42694   -0.88991   +0.91584
H   -0.82824   -0.07269   +2.35055
C   -1.95468   +1.38842   -0.60660
H   -2.58715   +1.29740   +1.44982
H   -1.05270   +2.10269   +1.22323
C   -3.35668   +0.95827   -1.00860
H   -1.81186   +2.42131   -0.91931
H   -1.22903   +0.81057   -1.18725
C   -3.69573   -0.48872   -0.70637
H   -4.07994   +1.60431   -0.50571
H   -3.48693   +1.13525   -2.07754
C   -5.06557   -0.89553   -1.21512
H   -2.93860   -1.14122   -1.14940
H   -3.65395   -0.66237   +0.37073
C   -5.40873   -2.33747   -0.90084
H   -5.81848   -0.23617   -0.78035
H   -5.10948   -0.73449   -2.29336
H   -6.39342   -2.60584   -1.27619
H   -4.68699   -3.01829   -1.34911
H   -5.40423   -2.51605   +0.17314
24
* E =   +3.055  kcal/mol ; (251) 065_060_182_176_059_264
H   +0.00000   +0.00000   +0.00000
O   +0.00000   +0.00000   +0.95450
C   -0.90204   +0.00000   +1.26657
C   -1.65265   -1.26197   +0.90267
H   -0.83369   +0.09421   +2.34831
H   -1.43741   +0.87801   +0.89742
C   -1.02727   -2.51784   +1.47670
H   -2.68279   -1.16500   +1.25136
H   -1.70992   -1.34398   -0.18648
C   -1.76689   -3.77850   +1.07271
H   +0.01392   -2.57675   +1.15783
H   -1.00943   -2.43255   +2.56479
C   -1.12832   -5.07823   +1.53754
H   -2.78825   -3.72997   +1.45845
H   -1.85786   -3.80195   -0.01509
C   -0.96339   -5.20489   +3.05172
H   -1.73953   -5.90079   +1.16740
H   -0.15243   -5.19189   +1.06030
C   +0.41145   -4.80791   +3.55856
H   -1.72842   -4.60440   +3.54856
H   -1.15469   -6.23424   +3.35283
H   +0.47419   -4.89021   +4.64168
```

H +1.17641 −5.45645 +3.13457
H +0.66713 −3.78653 +3.28806
24
∗ E = +3.069 kcal/mol ; (252) 180_179_098_300_295_180
H +0.00000 +0.00000 +0.00000
O +0.00000 +0.00000 +0.95353
C −0.89780 +0.00000 +1.27473
C −0.85216 −0.01168 +2.78246
H −1.43043 +0.89428 +0.93753
H −1.44201 −0.86893 +0.89775
C −2.23077 +0.00645 +3.43890
H −0.28878 −0.88778 +3.10645
H −0.27061 +0.85556 +3.09044
C −2.74371 −1.36034 +3.87262
H −2.19657 +0.64153 +4.32254
H −2.95068 +0.48229 +2.76974
C −2.90613 −2.38560 +2.76310
H −2.05665 −1.75959 +4.62020
H −3.70217 −1.23293 +4.37916
C −3.96888 −2.03480 +1.73835
H −1.94818 −2.53910 +2.26088
H −3.15924 −3.34850 +3.20984
C −4.09453 −3.07981 +0.64816
H −4.92698 −1.91535 +2.24711
H −3.74813 −1.06724 +1.28602
H −4.86767 −2.82020 −0.07127
H −3.15786 −3.18946 +0.10313
H −4.34363 −4.05378 +1.06607
24
∗ E = +3.081 kcal/mol ; (253) 181_064_182_097_299_295
H +0.00000 +0.00000 +0.00000
O +0.00000 +0.00000 +0.95329
C −0.89724 +0.00000 +1.27535
C −0.85372 +0.03527 +2.78132
H −1.43960 +0.87319 +0.90038
H −1.42651 −0.89434 +0.93864
C −0.22675 +1.30780 +3.32304
H −1.87321 −0.06074 +3.15858
H −0.30759 −0.84169 +3.12555
C −0.12457 +1.36145 +4.84642
H +0.76236 +1.42859 +2.88040
H −0.82144 +2.15036 +2.96643
C +1.23277 +0.95371 +5.40313
H −0.32441 +2.37777 +5.18426
H −0.91176 +0.74725 +5.28866

```
C    +1.67510   −0.46712   +5.09345
H    +1.98101   +1.64603   +5.01368
H    +1.22563   +1.09157   +6.48633
C    +0.79911   −1.53011   +5.72796
H    +1.71648   −0.61028   +4.01297
H    +2.69808   −0.59515   +5.44776
H    +1.17027   −2.52950   +5.51198
H    +0.77129   −1.41400   +6.81069
H    −0.22713   −1.47897   +5.36941
24
* E =  +3.090  kcal/mol ; (254) 062_177_098_299_294_180
H    +0.00000   +0.00000   +0.00000
O    +0.00000   +0.00000   +0.95443
C    −0.90189   +0.00000   +1.26673
C    −1.67133   −1.22898   +0.83078
H    −0.83576   +0.02191   +2.35235
H    −1.41641   +0.91349   +0.95893
C    −3.09414   −1.29352   +1.38350
H    −1.70175   −1.26792   −0.26197
H    −1.09704   −2.09692   +1.15062
C    −4.17132   −0.81274   +0.42060
H    −3.32595   −2.32260   +1.65272
H    −3.14756   −0.73103   +2.31768
C    −4.05182   +0.63210   −0.03421
H    −4.15602   −1.46003   −0.45780
H    −5.14854   −0.95629   +0.88486
C    −4.23961   +1.65408   +1.07153
H    −3.08361   +0.78875   −0.51623
H    −4.79671   +0.81817   −0.80962
C    −4.11268   +3.08000   +0.57486
H    −5.22078   +1.50743   +1.52629
H    −3.51244   +1.48407   +1.86605
H    −4.26428   +3.79940   +1.37604
H    −3.12500   +3.25964   +0.15245
H    −4.84433   +3.29070   −0.20344
24
* E =  +3.096  kcal/mol ; (255) 180_180_179_175_059_264
H    +0.00000   +0.00000   +0.00000
O    +0.00000   +0.00000   +0.95349
C    −0.89781   +0.00000   +1.27455
C    −0.85990   −0.00365   +2.78005
H    −1.43581   +0.88421   +0.92101
H    −1.43770   −0.88119   +0.91654
C    −2.24277   +0.00812   +3.40121
H    −0.30753   −0.88379   +3.10993
```

H −0.29083 +0.86429 +3.11334
C −2.19913 −0.02331 +4.91724
H −2.78559 +0.89807 +3.07311
H −2.80987 −0.84971 +3.03467
C −3.54941 +0.09835 +5.60570
H −1.72235 −0.95321 +5.23523
H −1.55132 +0.78196 +5.26762
C −4.55700 −0.99036 +5.23859
H −3.37585 +0.08580 +6.68118
H −3.98195 +1.07682 +5.38444
C −5.52338 −0.59231 +4.13758
H −4.01940 −1.89591 +4.94964
H −5.13704 −1.26021 +6.12032
H −6.20491 −1.40479 +3.89428
H −6.12505 +0.26075 +4.44710
H −5.00585 −0.30924 +3.22422
24
* E = +3.097 kcal/mol ; (256) 061_303_300_088_064_176
H +0.00000 +0.00000 +0.00000
O +0.00000 +0.00000 +0.95543
C −0.90295 +0.00000 +1.26773
C −1.68670 −1.21483 +0.82117
H −0.83761 +0.01827 +2.35232
H −1.41780 +0.91605 +0.96562
C −1.75502 −1.42486 −0.68540
H −1.24514 −2.09963 +1.27894
H −2.70050 −1.12553 +1.21621
C −2.39142 −0.28810 −1.48200
H −0.75804 −1.64583 −1.07023
H −2.32626 −2.33572 −0.85863
C −1.46312 +0.83116 −1.93983
H −2.85912 −0.70608 −2.37442
H −3.20517 +0.14120 −0.89437
C −0.38757 +0.39522 −2.91969
H −2.06990 +1.60022 −2.42065
H −0.99508 +1.32927 −1.08898
C +0.44481 +1.55720 −3.42389
H +0.27173 −0.34430 −2.46089
H −0.85871 −0.11426 −3.76181
H +1.20995 +1.22769 −4.12252
H −0.17753 +2.29005 −3.93436
H +0.94456 +2.06807 −2.60248
24
* E = +3.099 kcal/mol ; (257) 299_182_179_176_059_264
H +0.00000 +0.00000 +0.00000

```
O   +0.00000   +0.00000   +0.95438
C   -0.90210   +0.00000   +1.26593
C   -1.68321   +1.21454   +0.81870
H   -1.41936   -0.91282   +0.95597
H   -0.83665   -0.02246   +2.35129
C   -3.09796   +1.23628   +1.36451
H   -1.14523   +2.10921   +1.13315
H   -1.71622   +1.23894   -0.27375
C   -3.87615   +2.46231   +0.92580
H   -3.62867   +0.33593   +1.04532
H   -3.05876   +1.19858   +2.45454
C   -5.33433   +2.48989   +1.35629
H   -3.37604   +3.35268   +1.31336
H   -3.82887   +2.53979   -0.16190
C   -5.55822   +2.42494   +2.86666
H   -5.77964   +3.40223   +0.96105
H   -5.86863   +1.66545   +0.87860
C   -5.84103   +1.02835   +3.39023
H   -4.68860   +2.84288   +3.37808
H   -6.39590   +3.06666   +3.13695
H   -5.97083   +1.02808   +4.47041
H   -6.75414   +0.63233   +2.94877
H   -5.03895   +0.33357   +3.15326
24
 * E =   +3.101  kcal/mol  ;  (258) 060_177_179_175_059_264
H   +0.00000   +0.00000   +0.00000
O   +0.00000   +0.00000   +0.95440
C   -0.90195   +0.00000   +1.26643
C   -1.68655   -1.20889   +0.81015
H   -0.83551   +0.01347   +2.35187
H   -1.41683   +0.91673   +0.96416
C   -3.09591   -1.23933   +1.36931
H   -1.73065   -1.21790   -0.28230
H   -1.14481   -2.10727   +1.10668
C   -3.89213   -2.43640   +0.88524
H   -3.05288   -1.24936   +2.46096
H   -3.61303   -0.31907   +1.09135
C   -5.27595   -2.58246   +1.49845
H   -3.99183   -2.37611   -0.20105
H   -3.32052   -3.34418   +1.08542
C   -6.20852   -1.39403   +1.26748
H   -5.72728   -3.48483   +1.08750
H   -5.17911   -2.75926   +2.57202
C   -6.23687   -0.39854   +2.41325
H   -5.92356   -0.88674   +0.34329
```

H −7.22308 −1.75558 +1.10456
H −6.89523 +0.43964 +2.19486
H −6.59954 −0.87364 +3.32328
H −5.24989 +0.00322 +2.62910
24
∗ E = +3.101 kcal/mol ; (259) 060_298_300_094_182_297
H +0.00000 +0.00000 +0.00000
O +0.00000 +0.00000 +0.95497
C −0.90210 +0.00000 +1.26832
C −1.69175 −1.20470 +0.80676
H −0.83405 +0.00314 +2.35279
H −1.41380 +0.92266 +0.97870
C −1.84916 −1.33426 −0.70225
H −1.21037 −2.10177 +1.19520
H −2.68273 −1.15330 +1.26163
C −2.55575 −0.16366 −1.38310
H −0.87172 −1.48929 −1.16694
H −2.40021 −2.25336 −0.89494
C −1.62109 +0.90361 −1.93015
H −3.16034 −0.54476 −2.20448
H −3.25810 +0.29600 −0.68258
C −2.33177 +2.09364 −2.55516
H −0.97123 +1.27493 −1.13667
H −0.96580 +0.44670 −2.67633
C −3.16814 +1.74535 −3.77164
H −2.96287 +2.56822 −1.80192
H −1.58610 +2.83684 −2.83624
H −3.58468 +2.63968 −4.22897
H −2.56732 +1.23837 −4.52568
H −4.00085 +1.09290 −3.51964
24
∗ E = +3.103 kcal/mol ; (260) 294_064_177_175_059_264
H +0.00000 +0.00000 +0.00000
O +0.00000 +0.00000 +0.95393
C −0.90046 +0.00000 +1.26882
C −1.65341 +1.26569 +0.92263
H −1.44063 −0.87133 +0.88593
H −0.83165 −0.11115 +2.34746
C −1.83670 +1.47700 −0.56853
H −2.63270 +1.22368 +1.40345
H −1.12529 +2.11713 +1.35290
C −2.65883 +2.71158 −0.88755
H −0.86164 +1.57115 −1.05385
H −2.31723 +0.59447 −0.99721
C −2.78565 +3.04146 −2.36643

H −3.65868 +2.58464 −0.46610
H −2.22058 +3.56732 −0.37139
C −3.42505 +1.94359 −3.21592
H −3.37095 +3.95614 −2.45316
H −1.79885 +3.27930 −2.77008
C −2.42470 +1.04001 −3.91400
H −4.09035 +1.34503 −2.59010
H −4.06212 +2.39651 −3.97453
H −2.92434 +0.26243 −4.48787
H −1.80751 +1.61340 −4.60365
H −1.75526 +0.55220 −3.20950
24
* E = +3.116 kcal/mol ; (261) 067_062_182_063_060_268
H +0.00000 +0.00000 +0.00000
O +0.00000 +0.00000 +0.95457
C −0.90225 +0.00000 +1.26625
C −1.63257 −1.28501 +0.94344
H −0.83633 +0.13356 +2.34402
H −1.44697 +0.85772 +0.86512
C −1.01427 −2.49920 +1.60904
H −2.67447 −1.18327 +1.25429
H −1.65250 −1.41356 −0.14132
C −1.69586 −3.81205 +1.26178
H +0.04629 −2.54590 +1.36469
H −1.06006 −2.35957 +2.69053
C −1.65424 −4.23107 −0.20199
H −1.23923 −4.60591 +1.85518
H −2.73969 −3.76262 +1.57845
C −0.25712 −4.42488 −0.78647
H −2.20397 −5.16859 −0.28241
H −2.20597 −3.51686 −0.81657
C +0.33776 −3.19137 −1.44288
H +0.41412 −4.78422 −0.00393
H −0.29446 −5.21779 −1.53291
H +1.30122 −3.41590 −1.89578
H −0.31790 −2.82356 −2.23164
H +0.49342 −2.38284 −0.73341
24
* E = +3.120 kcal/mol ; (262) 182_062_183_265_061_063
H +0.00000 +0.00000 +0.00000
O +0.00000 +0.00000 +0.95326
C −0.89730 +0.00000 +1.27506
C −0.85961 +0.03994 +2.78173
H −1.44054 +0.87086 +0.89625
H −1.42541 −0.89603 +0.94101

C	−0.19662	+1.29585	+3.32100
H	−1.88066	−0.05645	+3.14841
H	−0.31381	−0.83676	+3.13370
C	−0.06140	+1.34448	+4.84115
H	+0.78740	+1.36275	+2.86192
H	−0.75121	+2.17339	+2.97701
C	−1.18426	+2.07418	+5.56579
H	+0.03872	+0.32923	+5.22965
H	+0.87041	+1.84675	+5.09861
C	−2.57823	+1.49269	+5.39562
H	−0.94888	+2.10817	+6.63142
H	−1.19359	+3.11109	+5.22515
C	−2.72613	+0.08892	+5.94949
H	−3.28954	+2.14888	+5.89758
H	−2.85708	+1.50965	+4.34096
H	−3.74753	−0.27148	+5.84898
H	−2.07713	−0.61952	+5.43873
H	−2.46851	+0.06163	+7.00741

24

∗ E = +3.122 kcal/mol ; (263) 067_296_182_176_059_263

H	+0.00000	+0.00000	+0.00000
O	+0.00000	+0.00000	+0.95386
C	−0.90013	+0.00000	+1.26948
C	−1.64455	−1.27840	+0.95204
H	−0.83133	+0.13421	+2.34551
H	−1.44705	+0.85917	+0.86925
C	−1.83373	−1.52207	−0.53322
H	−1.10603	−2.11628	+1.39612
H	−2.62100	−1.23539	+1.43829
C	−2.61479	−2.78986	−0.82358
H	−2.34772	−0.66676	−0.98092
H	−0.85759	−1.59040	−1.01783
C	−2.91694	−3.04216	−2.29241
H	−2.05913	−3.64155	−0.42505
H	−3.55506	−2.75794	−0.27038
C	−1.68474	−3.14797	−3.19038
H	−3.49626	−3.96235	−2.35852
H	−3.56909	−2.25104	−2.66936
C	−1.32885	−1.85632	−3.90410
H	−0.83433	−3.48964	−2.59670
H	−1.84803	−3.91893	−3.94229
H	−0.43082	−1.96957	−4.50769
H	−2.13589	−1.55127	−4.56816
H	−1.15914	−1.03917	−3.20685

24

```
 * E =   +3.156  kcal/mol  ;  (264)  182_062_182_297_299_094
 H    +0.00000    +0.00000    +0.00000
 O    +0.00000    +0.00000    +0.95329
 C    -0.89739    +0.00000    +1.27493
 C    -0.86131    +0.04296    +2.78107
 H    -1.44084    +0.87097    +0.89579
 H    -1.42571    -0.89582    +0.94063
 C    -0.19365    +1.29393    +3.31904
 H    -1.88550    -0.03702    +3.14649
 H    -0.32884    -0.83889    +3.14059
 C    -0.09367    +1.33238    +4.83434
 H    +0.80680    +1.35480    +2.89455
 H    -0.72549    +2.17736    +2.95890
 C    -1.41102    +1.34287    +5.59837
 H    +0.49584    +0.47591    +5.16689
 H    +0.47465    +2.21822    +5.12300
 C    -2.31612    +2.54002    +5.31332
 H    -1.96350    +0.41979    +5.41132
 H    -1.16855    +1.32649    +6.66066
 C    -3.35058    +2.31191    +4.22543
 H    -2.84411    +2.81193    +6.22685
 H    -1.69914    +3.40426    +5.05872
 H    -3.99235    +3.18241    +4.10552
 H    -2.89645    +2.10652    +3.25943
 H    -3.98919    +1.46531    +4.47414
24
 * E =   +3.165  kcal/mol  ;  (265)  177_297_286_262_060_177
 H    +0.00000    +0.00000    +0.00000
 O    +0.00000    +0.00000    +0.95326
 C    -0.89734    +0.00000    +1.27496
 C    -0.85568    -0.07553    +2.78136
 H    -1.41613    +0.91092    +0.96780
 H    -1.45020    -0.85068    +0.86733
 C    -0.21870    -1.35775    +3.29895
 H    -0.29362    +0.78634    +3.14014
 H    -1.87367    +0.02856    +3.15668
 C    -1.09058    -2.60452    +3.15162
 H    +0.72248    -1.49037    +2.76929
 H    +0.04125    -1.22887    +4.35091
 C    -1.84907    -2.98994    +4.41219
 H    -1.80462    -2.46316    +2.33679
 H    -0.47249    -3.45118    +2.85598
 C    -2.81054    -1.93610    +4.92828
 H    -2.40411    -3.91143    +4.22613
 H    -1.12941    -3.22546    +5.20007
```

```
C   −3.59162   −2.40519   +6.13942
H   −2.25860   −1.03089   +5.18407
H   −3.50236   −1.65738   +4.13055
H   −4.27152   −1.63747   +6.50157
H   −4.18322   −3.28880   +5.90548
H   −2.92233   −2.66706   +6.95738
24
* E =  +3.213  kcal/mol ; (266) 179_055_057_171_262_059
H   +0.00000   +0.00000   +0.00000
O   +0.00000   +0.00000   +0.95329
C   −0.89751   +0.00000   +1.27458
C   −0.85767   −0.01924   +2.78290
H   −1.42954   +0.88789   +0.92352
H   −1.43956   −0.87583   +0.91074
C   −0.03999   +1.10967   +3.38664
H   −1.88564   +0.02635   +3.14708
H   −0.44840   −0.97550   +3.10708
C   −0.50299   +2.49833   +2.97895
H   −0.06284   +1.01493   +4.47163
H   +1.00044   +0.98836   +3.08730
C   +0.18460   +3.62488   +3.74910
H   −0.31616   +2.62120   +1.91263
H   −1.58665   +2.58028   +3.10815
C   −0.61909   +4.16844   +4.92067
H   +1.15430   +3.27228   +4.10788
H   +0.39931   +4.45366   +3.07541
C   −0.96783   +3.14019   +5.97849
H   −0.06084   +4.98325   +5.38236
H   −1.53976   +4.61228   +4.53717
H   −1.50094   +3.59742   +6.80915
H   −1.60426   +2.35408   +5.57608
H   −0.07192   +2.66736   +6.37881
24
* E =  +3.221  kcal/mol ; (267) 063_057_065_061_262_181
H   +0.00000   +0.00000   +0.00000
O   +0.00000   +0.00000   +0.95459
C   −0.90171   +0.00000   +1.26791
C   −1.66710   −1.23996   +0.85626
H   −0.82876   +0.06499   +2.35059
H   −1.42918   +0.89453   +0.92764
C   −1.02880   −2.53902   +1.31785
H   −2.68915   −1.16233   +1.23086
H   −1.75254   −1.25199   −0.23306
C   −0.96850   −2.74289   +2.82505
H   −1.57989   −3.37167   +0.87809
```

H −0.01728 −2.58954 +0.91653
C −2.32656 −2.77288 +3.52503
H −0.45067 −3.68443 +3.00297
H −0.33899 −1.97609 +3.28000
C −2.74967 −1.45879 +4.15955
H −3.09465 −3.09561 +2.81688
H −2.31477 −3.53019 +4.30995
C −4.08877 −1.55487 +4.86230
H −1.98329 −1.15051 +4.87257
H −2.79312 −0.67191 +3.40686
H −4.36924 −0.61062 +5.32320
H −4.87827 −1.83047 +4.16484
H −4.06539 −2.31173 +5.64475
24
* E = +3.226 kcal/mol ; (268) 184_284_060_266_176_178
H +0.00000 +0.00000 +0.00000
O +0.00000 +0.00000 +0.95364
C −0.89795 +0.00000 +1.27476
C −0.88020 +0.09267 +2.78135
H −1.44096 +0.85543 +0.86683
H −1.42235 −0.90814 +0.96058
C −0.52388 −1.17768 +3.53938
H −0.18591 +0.89006 +3.04953
H −1.86907 +0.41976 +3.10418
C +0.84820 −1.77534 +3.23794
H −0.59149 −0.94409 +4.60163
H −1.29203 −1.93261 +3.35404
C +0.82902 −2.84594 +2.16259
H +1.53311 −0.98107 +2.94073
H +1.25750 −2.21573 +4.14838
C +2.21103 −3.35788 +1.80921
H +0.20919 −3.68042 +2.50082
H +0.35682 −2.45218 +1.26280
C +2.18735 −4.45554 +0.76471
H +2.81466 −2.52262 +1.45180
H +2.70231 −3.72353 +2.71287
H +3.18933 −4.80347 +0.52345
H +1.61356 −5.31387 +1.11095
H +1.72752 −4.10619 −0.15859
24
* E = +3.236 kcal/mol ; (269) 063_061_097_299_188_297
H +0.00000 +0.00000 +0.00000
O +0.00000 +0.00000 +0.95457
C −0.90183 +0.00000 +1.26748
C −1.65792 −1.24951 +0.86206

H	−0.82856	+0.07403	+2.34845
H	−1.43114	+0.88855	+0.91456
C	−1.05995	−2.54200	+1.41115
H	−2.69863	−1.14909	+1.17849
H	−1.68356	−1.28351	−0.22923
C	−1.71322	−3.04707	+2.68844
H	−1.14010	−3.32573	+0.65910
H	+0.00785	−2.38848	+1.57393
C	−1.62399	−2.09624	+3.86878
H	−2.76038	−3.27389	+2.47992
H	−1.24804	−3.99470	+2.96805
C	−2.10047	−2.69868	+5.18063
H	−0.58872	−1.76913	+3.98195
H	−2.20955	−1.19712	+3.66299
C	−3.56548	−3.09145	+5.18046
H	−1.48959	−3.57234	+5.41438
H	−1.92283	−1.98174	+5.98208
H	−3.87847	−3.44491	+6.16021
H	−4.19638	−2.24271	+4.91863
H	−3.77062	−3.88658	+4.46725

24

* E = +3.247 kcal/mol ; (270) 291_059_267_171_056_057

H	+0.00000	+0.00000	+0.00000
O	+0.00000	+0.00000	+0.95434
C	−0.90247	+0.00000	+1.26469
C	−1.64471	+1.29110	+0.99068
H	−1.45328	−0.83709	+0.82526
H	−0.84556	−0.17808	+2.33533
C	−1.72350	+1.65404	−0.48955
H	−2.64686	+1.17903	+1.40530
H	−1.16760	+2.10272	+1.54109
C	−0.60044	+2.56808	−0.95465
H	−1.72880	+0.73903	−1.09237
H	−2.67777	+2.13791	−0.69629
C	−0.54315	+2.75798	−2.46189
H	−0.71601	+3.53545	−0.46286
H	+0.35726	+2.18363	−0.60524
C	−1.82381	+3.28477	−3.09201
H	+0.27407	+3.44136	−2.69815
H	−0.28748	+1.80599	−2.93163
C	−2.28580	+4.60872	−2.51539
H	−1.66175	+3.39527	−4.16399
H	−2.61835	+2.54512	−2.98649
H	−3.15118	+4.99167	−3.05111
H	−2.56673	+4.51556	−1.46772

```
 H    −1.49867    +5.35905    −2.57757
24
 * E =  +3.252  kcal/mol ; (271) 185_065_097_299_188_297
 H    +0.00000    +0.00000    +0.00000
 O    +0.00000    +0.00000    +0.95332
 C    −0.89782    +0.00000    +1.27384
 C    −0.84904    +0.11712    +2.77819
 H    −1.45592    +0.83278    +0.84185
 H    −1.40399    −0.92675    +0.99279
 C    −0.27516    +1.43982    +3.27987
 H    −1.85522    −0.03265    +3.17435
 H    −0.24624    −0.71450    +3.14020
 C    −1.31778    +2.48379    +3.64851
 H    +0.33977    +1.25228    +4.15867
 H    +0.40202    +1.83570    +2.52205
 C    −2.21481    +2.92236    +2.50469
 H    −1.93048    +2.09349    +4.46325
 H    −0.80780    +3.36307    +4.04801
 C    −3.11263    +4.10150    +2.84346
 H    −1.59123    +3.18479    +1.64763
 H    −2.84005    +2.08544    +2.18486
 C    −4.10657    +3.81778    +3.95353
 H    −2.49111    +4.95457    +3.12117
 H    −3.65490    +4.40094    +1.94654
 H    −4.77601    +4.66095    +4.10769
 H    −4.71914    +2.94890    +3.71532
 H    −3.60905    +3.62009    +4.90019
24
 * E =  +3.252  kcal/mol ; (272) 066_297_269_061_060_178
 H    +0.00000    +0.00000    +0.00000
 O    +0.00000    +0.00000    +0.95439
 C    −0.90084    +0.00000    +1.26958
 C    −1.64402    −1.27974    +0.93448
 H    −0.82848    +0.10028    +2.34939
 H    −1.43212    +0.87941    +0.90180
 C    −1.84000    −1.56624    −0.55149
 H    −1.08280    −2.09607    +1.38722
 H    −2.61640    −1.25732    +1.43144
 C    −3.12270    −1.01907    −1.16253
 H    −0.97576    −1.21556    −1.12046
 H    −1.84426    −2.64525    −0.69866
 C    −3.30334    +0.48925    −1.11938
 H    −3.17647    −1.34735    −2.20205
 H    −3.96853    −1.48610    −0.65538
 C    −2.22944    +1.27277    −1.85033
```

H −4.27091 +0.73349 −1.56100
H −3.36223 +0.82881 −0.08359
C −2.47568 +2.76757 −1.82170
H −1.25100 +1.06591 −1.41327
H −2.17482 +0.92571 −2.88363
H −1.70089 +3.31274 −2.35526
H −3.43179 +3.01454 −2.27992
H −2.49699 +3.13949 −0.79852
24
* E = +3.252 kcal/mol ; (273) 071_296_180_295_301_095
H +0.00000 +0.00000 +0.00000
O +0.00000 +0.00000 +0.95382
C −0.90025 +0.00000 +1.26898
C −1.60771 −1.31503 +1.02487
H −0.83649 +0.19892 +2.33524
H −1.46448 +0.82164 +0.81789
C −1.77399 −1.64074 −0.44730
H −1.04739 −2.10255 +1.52772
H −2.59062 −1.27467 +1.49850
C −2.47298 −2.96314 −0.71530
H −2.34301 −0.83932 −0.92345
H −0.79896 −1.65461 −0.93959
C −1.73242 −4.20895 −0.24764
H −3.45992 −2.94143 −0.24954
H −2.64657 −3.05287 −1.78887
C −0.34738 −4.40039 −0.86469
H −1.64653 −4.21080 +0.84037
H −2.35762 −5.06689 −0.49293
C +0.80046 −3.87201 −0.02203
H −0.17846 −5.46259 −1.03809
H −0.32664 −3.93396 −1.85230
H +1.75841 −4.06627 −0.50019
H +0.73570 −2.80154 +0.15765
H +0.81667 −4.36151 +0.95077
24
* E = +3.254 kcal/mol ; (274) 069_299_092_177_058_055
H +0.00000 +0.00000 +0.00000
O +0.00000 +0.00000 +0.95443
C −0.90182 +0.00000 +1.26692
C −1.64665 −1.29027 +0.99793
H −0.84146 +0.17780 +2.33749
H −1.45405 +0.83752 +0.82989
C −1.76678 −1.64616 −0.48133
H −1.15702 −2.10385 +1.53405
H −2.63988 −1.18158 +1.43473

```
C   −0.64884   −2.53612   −1.00066
H   −2.71432   −2.15636   −0.65991
H   −1.81966   −0.72386   −1.06814
C   −0.81557   −2.95021   −2.45416
H   +0.31788   −2.05352   −0.85807
H   −0.61090   −3.43494   −0.38404
C   −0.91917   −1.80043   −3.44582
H   +0.02868   −3.58204   −2.73454
H   −1.70618   −3.57415   −2.54663
C   +0.23910   −0.82529   −3.37381
H   −0.97653   −2.21554   −4.45172
H   −1.85547   −1.26274   −3.29370
H   +0.18192   −0.08127   −4.16451
H   +0.24961   −0.28496   −2.42751
H   +1.19503   −1.33858   −3.46633
24
* E =  +3.265  kcal/mol ; (275) 179_055_059_171_060_265
H   +0.00000   +0.00000   +0.00000
O   +0.00000   +0.00000   +0.95332
C   −0.89746   +0.00000   +1.27486
C   −0.85674   −0.01613   +2.78324
H   −1.43082   +0.88606   +0.92153
H   −1.43872   −0.87749   +0.91382
C   −0.04238   +1.11782   +3.38373
H   −1.88418   +0.02557   +3.14885
H   −0.44233   −0.96999   +3.10792
C   −0.53955   +2.49817   +2.99757
H   −0.05623   +1.01924   +4.47065
H   +0.99341   +1.00134   +3.06887
C   +0.12378   +3.64598   +3.74333
H   −0.38282   +2.64679   +1.92740
H   −1.61885   +2.54973   +3.16050
C   +1.63744   +3.74121   +3.55431
H   −0.34145   +4.57342   +3.41022
H   −0.10115   +3.56415   +4.80940
C   +2.44488   +3.06331   +4.64662
H   +1.90442   +3.31905   +2.58361
H   +1.93107   +4.78973   +3.51451
H   +3.51334   +3.15200   +4.46106
H   +2.23955   +3.51926   +5.61403
H   +2.21103   +2.00507   +4.72847
24
* E =  +3.282  kcal/mol ; (276) 064_061_075_098_300_183
H   +0.00000   +0.00000   +0.00000
O   +0.00000   +0.00000   +0.95453
```

C	−0.90197	+0.00000	+1.26689
C	−1.65642	−1.25643	+0.88476
H	−0.83145	+0.08958	+2.34846
H	−1.43525	+0.88217	+0.90519
C	−1.03493	−2.52242	+1.45740
H	−2.69023	−1.15104	+1.21560
H	−1.69068	−1.32053	−0.20551
C	−1.28400	−2.72354	+2.95202
H	−1.41187	−3.39059	+0.91425
H	+0.03491	−2.48113	+1.25965
C	−2.43444	−3.66513	+3.27202
H	−0.38375	−3.11414	+3.42386
H	−1.46991	−1.75833	+3.42863
C	−3.78556	−3.23204	+2.73514
H	−2.20047	−4.65692	+2.87728
H	−2.50725	−3.78180	+4.35489
C	−4.90236	−4.17013	+3.14709
H	−4.00631	−2.22179	+3.08624
H	−3.74616	−3.17538	+1.64654
H	−5.86298	−3.85067	+2.74975
H	−4.71507	−5.18077	+2.78775
H	−4.99170	−4.21991	+4.23102

24

∗ E = +3.290 kcal/mol ; (277) 060_177_262_061_173_063

H	+0.00000	+0.00000	+0.00000
O	+0.00000	+0.00000	+0.95452
C	−0.90222	+0.00000	+1.26613
C	−1.68732	−1.20770	+0.79883
H	−0.83878	+0.02245	+2.35032
H	−1.41959	+0.91326	+0.95702
C	−3.10812	−1.25890	+1.35802
H	−1.71806	−1.18806	−0.29222
H	−1.13799	−2.10949	+1.07279
C	−3.27380	−2.15477	+2.57590
H	−3.43536	−0.24640	+1.60860
H	−3.79085	−1.60631	+0.58448
C	−2.43307	−1.75265	+3.77384
H	−4.32561	−2.15895	+2.86921
H	−3.03439	−3.17934	+2.28741
C	−2.73900	−2.54500	+5.03455
H	−1.37363	−1.86640	+3.53414
H	−2.59027	−0.69090	+3.97547
C	−2.44372	−4.02792	+4.91484
H	−2.15810	−2.13099	+5.85844
H	−3.78784	−2.40256	+5.30085

```
 H   -2.60360   -4.53826   +5.86178
 H   -3.08001   -4.50896   +4.17536
 H   -1.40888   -4.19621   +4.61934
24
 * E =  +3.301  kcal/mol ; (278) 064_060_186_267_061_062
 H   +0.00000   +0.00000   +0.00000
 O   +0.00000   +0.00000   +0.95455
 C   -0.90224   +0.00000   +1.26622
 C   -1.65945   -1.25549   +0.89020
 H   -0.83414   +0.08485   +2.34882
 H   -1.43423   +0.88303   +0.90435
 C   -1.03146   -2.51567   +1.45988
 H   -2.69160   -1.14225   +1.22055
 H   -1.69943   -1.33288   -0.20053
 C   -1.66510   -3.82334   +0.99181
 H   +0.02309   -2.50256   +1.18996
 H   -1.05881   -2.46743   +2.55155
 C   -2.75635   -4.37679   +1.89799
 H   -2.04838   -3.69896   -0.02300
 H   -0.88963   -4.58506   +0.91806
 C   -3.97823   -3.49407   +2.09262
 H   -3.08268   -5.34005   +1.50096
 H   -2.31641   -4.58611   +2.87458
 C   -4.74051   -3.21457   +0.81215
 H   -4.64525   -3.98174   +2.80370
 H   -3.68321   -2.55575   +2.56429
 H   -5.63328   -2.62330   +1.00318
 H   -4.13517   -2.66937   +0.09066
 H   -5.05431   -4.14350   +0.33786
24
 * E =  +3.303  kcal/mol ; (279) 179_179_065_060_264_181
 H   +0.00000   +0.00000   +0.00000
 O   +0.00000   +0.00000   +0.95351
 C   -0.89781   +0.00000   +1.27463
 C   -0.85038   -0.01303   +2.78059
 H   -1.43302   +0.89148   +0.93422
 H   -1.43877   -0.87242   +0.90037
 C   -2.22172   +0.00403   +3.43286
 H   -0.28093   -0.88290   +3.10742
 H   -0.28259   +0.85899   +3.10278
 C   -3.09548   -1.22071   +3.19068
 H   -2.08636   +0.11703   +4.50907
 H   -2.76229   +0.89396   +3.10470
 C   -2.49675   -2.54070   +3.67236
 H   -4.04228   -1.04826   +3.70132
```

H −3.34772 −1.30414 +2.13164
C −1.76796 −3.34202 +2.60662
H −1.81748 −2.34795 +4.50691
H −3.28951 −3.17121 +4.07724
C −1.22719 −4.65490 +3.13590
H −2.45709 −3.53654 +1.78263
H −0.94966 −2.75903 +2.18578
H −0.72271 −5.22331 +2.35811
H −0.51155 −4.48725 +3.93914
H −2.02795 −5.27579 +3.53488
24
* E = +3.305 kcal/mol ; (280) 180_182_099_299_187_297
H +0.00000 +0.00000 +0.00000
O +0.00000 +0.00000 +0.95350
C −0.89766 +0.00000 +1.27501
C −0.85512 −0.00295 +2.78262
H −1.43213 +0.89054 +0.93161
H −1.44237 −0.87260 +0.90750
C −2.24103 −0.04410 +3.42321
H −0.25886 −0.85527 +3.11093
H −0.31173 +0.88724 +3.09428
C −2.68367 −1.42524 +3.88111
H −2.26076 +0.62205 +4.28400
H −2.97954 +0.35614 +2.72350
C −2.78078 −2.45522 +2.77069
H −1.98843 −1.77545 +4.64550
H −3.65671 −1.34114 +4.36957
C −3.38130 −3.78111 +3.20944
H −3.38501 −2.04328 +1.95936
H −1.78829 −2.63768 +2.35259
C −2.56025 −4.51156 +4.25492
H −4.38845 −3.60801 +3.59274
H −3.49591 −4.42134 +2.33484
H −2.98592 −5.48711 +4.47828
H −1.53926 −4.66773 +3.90885
H −2.50985 −3.95745 +5.18941
24
* E = +3.326 kcal/mol ; (281) 058_178_295_300_097_179
H +0.00000 +0.00000 +0.00000
O +0.00000 +0.00000 +0.95450
C −0.90223 +0.00000 +1.26604
C −1.69554 −1.18404 +0.76055
H −0.83315 −0.01576 +2.35051
H −1.40910 +0.92997 +0.99059
C −3.12358 −1.22540 +1.27804

```
H   -1.71879   -1.14502   -0.33104
H   -1.16734   -2.10155   +1.02019
C   -3.27888   -1.43392   +2.77920
H   -3.63205   -0.30196   +0.99427
H   -3.65336   -2.02909   +0.76544
C   -2.67489   -2.73272   +3.30999
H   -2.85518   -0.58879   +3.32497
H   -4.34566   -1.41578   +2.99902
C   -1.27747   -2.59826   +3.89135
H   -3.32091   -3.14118   +4.08815
H   -2.66471   -3.48086   +2.51281
C   -0.74069   -3.91180   +4.42277
H   -0.58991   -2.20420   +3.14395
H   -1.30243   -1.86158   +4.69626
H   +0.25284   -3.79483   +4.84884
H   -1.38990   -4.31498   +5.19858
H   -0.67506   -4.65651   +3.63112
24
* E =  +3.349  kcal/mol ; (282) 065_061_182_097_299_295
H   +0.00000   +0.00000   +0.00000
O   +0.00000   +0.00000   +0.95448
C   -0.90222   +0.00000   +1.26598
C   -1.65342   -1.26023   +0.89734
H   -0.83395   +0.09057   +2.34818
H   -1.43598   +0.87991   +0.89911
C   -1.03947   -2.51229   +1.49781
H   -2.68591   -1.15888   +1.23815
H   -1.70341   -1.33857   -0.19050
C   -1.74671   -3.81237   +1.11851
H   +0.01245   -2.56441   +1.21395
H   -1.04721   -2.39530   +2.58222
C   -1.10667   -4.56970   -0.03694
H   -1.75531   -4.47946   +1.97962
H   -2.79645   -3.60810   +0.89816
C   -1.05824   -3.83404   -1.36601
H   -0.08912   -4.83635   +0.25315
H   -1.63891   -5.51237   -0.17857
C   -2.42522   -3.54681   -1.95627
H   -0.49756   -2.90528   -1.24831
H   -0.48628   -4.43451   -2.07343
H   -2.34471   -3.05505   -2.92323
H   -2.98492   -4.46977   -2.10105
H   -3.02091   -2.90433   -1.31085
24
* E =  +3.352  kcal/mol ; (283) 061_178_099_299_187_297
```

```
H    +0.00000    +0.00000    +0.00000
O    +0.00000    +0.00000    +0.95442
C    −0.90184    +0.00000    +1.26683
C    −1.67736    −1.22340    +0.82583
H    −0.83499    +0.01597    +2.35237
H    −1.41818    +0.91449    +0.96518
C    −3.11198    −1.25084    +1.35161
H    −1.68680    −1.27165    −0.26686
H    −1.12514    −2.09850    +1.16396
C    −4.16192    −0.78959    +0.35271
H    −3.36182    −2.26255    +1.66616
H    −3.17902    −0.63573    +2.25260
C    −3.99479    +0.64298    −0.11965
H    −4.14453    −1.46369    −0.50519
H    −5.15022    −0.89754    +0.80401
C    −5.13883    +1.14509    −0.98539
H    −3.89651    +1.29318    +0.75202
H    −3.06113    +0.73901    −0.67914
C    −5.29714    +0.39196    −2.29232
H    −6.06857    +1.08741    −0.41682
H    −4.97971    +2.20182    −1.19921
H    −6.07540    +0.83478    −2.90943
H    −4.37146    +0.40877    −2.86651
H    −5.56496    −0.64955    −2.12972
24
* E =   +3.353  kcal/mol ;  (284) 068_295_184_266_061_063
H    +0.00000    +0.00000    +0.00000
O    +0.00000    +0.00000    +0.95387
C    −0.90047    +0.00000    +1.26854
C    −1.63766    −1.28548    +0.96245
H    −0.83401    +0.14585    +2.34319
H    −1.45009    +0.85259    +0.85766
C    −1.83005    −1.52570    −0.52557
H    −1.09719    −2.11029    +1.42302
H    −2.61680    −1.23994    +1.44368
C    −2.65341    −2.76363    −0.87803
H    −2.31378    −0.64150    −0.94572
H    −0.85693    −1.60424    −1.01793
C    −1.83931    −4.01978    −1.15572
H    −3.37880    −2.95298    −0.08487
H    −3.24301    −2.55443    −1.76986
C    −0.98541    −4.53134    −0.00710
H    −2.52188    −4.81400    −1.46409
H    −1.19397    −3.82616    −2.01447
C    −1.78769    −4.95212    +1.20871
```

H	−0.40693	−5.38391	−0.36302
H	−0.25156	−3.77481	+0.27335
H	−1.14396	−5.35685	+1.98623
H	−2.33673	−4.11813	+1.64135
H	−2.51413	−5.72035	+0.94695

24
∗ E = +3.360 kcal/mol ; (285) 063_061_099_300_182_066

H	+0.00000	+0.00000	+0.00000
O	+0.00000	+0.00000	+0.95458
C	−0.90184	+0.00000	+1.26748
C	−1.66103	−1.24367	+0.85062
H	−0.82742	+0.06157	+2.34948
H	−1.42800	+0.89418	+0.92417
C	−1.06780	−2.53961	+1.39730
H	−2.70300	−1.14137	+1.16220
H	−1.68192	−1.27136	−0.24090
C	−1.75750	−3.07626	+2.64246
H	−1.11011	−3.31144	+0.63014
H	−0.00831	−2.37715	+1.60047
C	−1.74611	−2.13420	+3.83230
H	−2.79468	−3.32223	+2.39951
H	−1.28029	−4.01633	+2.91856
C	−2.39304	−2.70878	+5.08298
H	−0.71543	−1.84838	+4.05748
H	−2.26818	−1.21390	+3.56673
C	−1.65626	−3.89653	+5.67221
H	−2.45716	−1.92324	+5.83578
H	−3.42181	−2.99373	+4.85507
H	−2.11489	−4.21959	+6.60398
H	−1.65523	−4.75047	+4.99889
H	−0.61857	−3.64337	+5.88533

24
∗ E = +3.362 kcal/mol ; (286) 062_053_057_171_262_059

H	+0.00000	+0.00000	+0.00000
O	+0.00000	+0.00000	+0.95460
C	−0.90170	+0.00000	+1.26796
C	−1.67515	−1.23056	+0.84228
H	−0.82977	+0.04773	+2.35142
H	−1.42548	+0.90178	+0.94206
C	−0.99088	−2.54058	+1.19314
H	−2.66111	−1.18761	+1.30976
H	−1.84932	−1.19000	−0.23543
C	−0.68120	−2.69795	+2.67244
H	−1.61641	−3.36513	+0.85343
H	−0.05579	−2.61744	+0.63784

C −0.19460 −4.09587 +3.05128
H +0.07615 −1.96333 +2.94217
H −1.56869 −2.45071 +3.26291
C −1.27439 −5.00801 +3.61338
H +0.25724 −4.56720 +2.17531
H +0.60060 −4.01924 +3.79166
C −2.44433 −5.25128 +2.68061
H −0.82329 −5.96395 +3.88030
H −1.64478 −4.57795 +4.54575
H −3.15795 −5.94524 +3.11920
H −2.97948 −4.32866 +2.46278
H −2.11163 −5.67248 +1.73269
24
* E = +3.362 kcal/mol ; (287) 181_181_179_096_298_296
H +0.00000 +0.00000 +0.00000
O +0.00000 +0.00000 +0.95347
C −0.89782 +0.00000 +1.27444
C −0.86070 +0.01996 +2.78010
H −1.44102 +0.87547 +0.90718
H −1.43322 −0.88959 +0.93009
C −2.25001 −0.00144 +3.39221
H −0.29421 −0.84667 +3.12207
H −0.30314 +0.89959 +3.09558
C −2.27107 −0.00123 +4.91998
H −2.82676 +0.85168 +3.02530
H −2.76733 −0.88918 +3.02584
C −2.43832 +1.37193 +5.55598
H −3.09655 −0.62253 +5.26536
H −1.36587 −0.48166 +5.29506
C −1.34195 +2.38200 +5.25941
H −3.39470 +1.78495 +5.23012
H −2.51533 +1.24826 +6.63795
C +0.01864 +1.98073 +5.79492
H −1.28274 +2.55630 +4.18407
H −1.62783 +3.33870 +5.69690
H +0.76302 +2.74909 +5.59936
H −0.02065 +1.82380 +6.87205
H +0.37727 +1.05812 +5.34342
24
* E = +3.365 kcal/mol ; (288) 181_063_184_300_099_072
H +0.00000 +0.00000 +0.00000
O +0.00000 +0.00000 +0.95329
C −0.89750 +0.00000 +1.27463
C −0.86006 +0.03555 +2.78124
H −1.43987 +0.87281 +0.89903

H	−1.42632	−0.89433	+0.93720
C	−0.21379	+1.29575	+3.32336
H	−1.88327	−0.05618	+3.14775
H	−0.31559	−0.84207	+3.13367
C	−0.07603	+1.32979	+4.83710
H	+0.76836	+1.40252	+2.86604
H	−0.80112	+2.15954	+3.00091
C	−1.40349	+1.23677	+5.59100
H	+0.58407	+0.51926	+5.15225
H	+0.43879	+2.25065	+5.10760
C	−1.73766	−0.15294	+6.11716
H	−1.39830	+1.92413	+6.43805
H	−2.20638	+1.58166	+4.93615
C	−0.87926	−0.56310	+7.29854
H	−2.78605	−0.18093	+6.41494
H	−1.62794	−0.88632	+5.31795
H	−1.12988	−1.56366	+7.64359
H	+0.17955	−0.56059	+7.04699
H	−1.01827	+0.12106	+8.13431

24

∗ E = +3.366 kcal/mol ; (289) 065_060_185_298_298_093

H	+0.00000	+0.00000	+0.00000
O	+0.00000	+0.00000	+0.95449
C	−0.90218	+0.00000	+1.26613
C	−1.65496	−1.26110	+0.90211
H	−0.83392	+0.09371	+2.34809
H	−1.43641	+0.87883	+0.89752
C	−1.02507	−2.51424	+1.47836
H	−2.68569	−1.15407	+1.24303
H	−1.70650	−1.34668	−0.18749
C	−1.68976	−3.80286	+1.02531
H	+0.02509	−2.53467	+1.19031
H	−1.02984	−2.45498	+2.56844
C	−3.15004	−3.98371	+1.41735
H	−1.61000	−3.87331	−0.06145
H	−1.12252	−4.64609	+1.42287
C	−3.41954	−4.01856	+2.92056
H	−3.76311	−3.20532	+0.95825
H	−3.48904	−4.92022	+0.97516
C	−3.77659	−2.67766	+3.53692
H	−4.24068	−4.70722	+3.11709
H	−2.55114	−4.43900	+3.43180
H	−4.00911	−2.78225	+4.59465
H	−2.96986	−1.95433	+3.45179
H	−4.65245	−2.25162	+3.04930

```
24
 * E =  +3.367  kcal/mol ; (290) 061_177_179_297_300_094
 H   +0.00000   +0.00000   +0.00000
 O   +0.00000   +0.00000   +0.95440
 C   −0.90205   +0.00000   +1.26614
 C   −1.68304   −1.21633   +0.82383
 H   −0.83719   +0.02913   +2.35145
 H   −1.42139   +0.91053   +0.95107
 C   −3.09081   −1.23518   +1.38844
 H   −1.71634   −1.23723   −0.26766
 H   −1.14367   −2.11144   +1.13509
 C   −3.91604   −2.43617   +0.95696
 H   −3.03125   −1.22901   +2.47790
 H   −3.61278   −0.31547   +1.11680
 C   −4.20323   −2.55211   −0.53391
 H   −3.41278   −3.34588   +1.28886
 H   −4.86987   −2.40845   +1.48622
 C   −4.97197   −1.37865   −1.13829
 H   −3.27404   −2.69956   −1.08764
 H   −4.77714   −3.46663   −0.68031
 C   −4.10220   −0.29148   −1.74453
 H   −5.63383   −1.75245   −1.91873
 H   −5.62543   −0.94620   −0.37772
 H   −4.70951   +0.48658   −2.20223
 H   −3.46221   +0.18761   −1.00765
 H   −3.45817   −0.70352   −2.52031
24
 * E =  +3.369  kcal/mol ; (291) 184_065_099_300_182_066
 H   +0.00000   +0.00000   +0.00000
 O   +0.00000   +0.00000   +0.95334
 C   −0.89774   +0.00000   +1.27415
 C   −0.84701   +0.10259   +2.77942
 H   −1.45189   +0.84089   +0.85181
 H   −1.40816   −0.92119   +0.98285
 C   −0.27183   +1.42195   +3.28900
 H   −1.85246   −0.05082   +3.17600
 H   −0.24411   −0.73244   +3.13316
 C   −1.31187   +2.44840   +3.71160
 H   +0.37542   +1.22663   +4.14241
 H   +0.37527   +1.84087   +2.51745
 C   −2.27032   +2.87437   +2.61464
 H   −1.89239   +2.04649   +4.54640
 H   −0.79124   +3.32387   +4.09940
 C   −3.27440   +3.93250   +3.04479
 H   −1.69907   +3.24556   +1.75948
```

H	−2.82035	+2.00157	+2.26009
C	−2.65268	+5.27088	+3.39552
H	−3.99667	+4.07741	+2.24136
H	−3.84297	+3.55933	+3.89854
H	−3.41666	+6.01121	+3.62181
H	−2.00101	+5.20223	+4.26331
H	−2.05829	+5.65266	+2.56640

24

* E = +3.373 kcal/mol ; (292) 179_054_055_058_064_269

H	+0.00000	+0.00000	+0.00000
O	+0.00000	+0.00000	+0.95335
C	−0.89750	+0.00000	+1.27487
C	−0.85979	−0.01273	+2.78300
H	−1.43046	+0.88694	+0.92074
H	−1.43940	−0.87699	+0.91375
C	−0.03056	+1.11578	+3.37603
H	−1.88823	+0.03008	+3.14305
H	−0.45142	−0.96727	+3.11353
C	−0.43828	+2.50931	+2.92593
H	−0.06514	+1.04968	+4.46363
H	+1.00877	+0.95809	+3.09377
C	−1.87635	+2.92307	+3.20603
H	+0.22549	+3.23275	+3.40232
H	−0.24721	+2.59747	+1.85541
C	−2.25625	+3.02325	+4.68169
H	−2.02863	+3.89650	+2.73993
H	−2.56987	+2.24556	+2.70121
C	−2.81096	+1.75020	+5.29543
H	−1.38788	+3.35868	+5.25231
H	−3.00682	+3.80480	+4.79771
H	−3.12623	+1.91858	+6.32315
H	−3.68076	+1.40224	+4.73941
H	−2.08429	+0.94274	+5.30701

24

* E = +3.377 kcal/mol ; (293) 062_178_179_095_299_297

H	+0.00000	+0.00000	+0.00000
O	+0.00000	+0.00000	+0.95433
C	−0.90175	+0.00000	+1.26672
C	−1.67854	−1.22347	+0.83570
H	−0.83535	+0.03593	+2.35176
H	−1.42370	+0.90691	+0.94691
C	−3.09825	−1.22569	+1.37555
H	−1.71421	−1.25909	−0.25666
H	−1.13128	−2.10780	+1.15621
C	−3.94594	−2.42158	+0.94473

```
H   -3.07151   -1.17781   +2.46707
H   -3.58386   -0.30455   +1.05058
C   -3.97558   -3.57497   +1.93819
H   -4.97409   -2.09401   +0.79555
H   -3.60682   -2.77472   -0.03077
C   -2.63819   -4.23200   +2.23759
H   -4.40491   -3.20967   +2.87277
H   -4.66356   -4.33700   +1.56699
C   -2.00344   -4.90032   +1.03372
H   -1.95247   -3.49668   +2.66092
H   -2.78812   -4.97703   +3.01900
H   -1.07169   -5.39359   +1.30058
H   -2.66872   -5.65345   +0.61358
H   -1.77936   -4.18628   +0.24381
24
* E =   +3.384  kcal/mol  ;  (294)  181_181_180_063_060_266
H   +0.00000   +0.00000   +0.00000
O   +0.00000   +0.00000   +0.95347
C   -0.89764   +0.00000   +1.27496
C   -0.86043   +0.01769   +2.78031
H   -1.44026   +0.87760   +0.91058
H   -1.43445   -0.88759   +0.92798
C   -2.24760   -0.00521   +3.39231
H   -0.28745   -0.84402   +3.12389
H   -0.30999   +0.90288   +3.09621
C   -2.24956   +0.01366   +4.91190
H   -2.83549   +0.83458   +3.01627
H   -2.76845   -0.90375   +3.05700
C   -1.66414   +1.25774   +5.56655
H   -3.27776   -0.10211   +5.25867
H   -1.70759   -0.86154   +5.27448
C   -2.36769   +2.56631   +5.21045
H   -1.71398   +1.10790   +6.64470
H   -0.60167   +1.34289   +5.33192
C   -1.75312   +3.32236   +4.04571
H   -3.42089   +2.36397   +5.00395
H   -2.35809   +3.22312   +6.07967
H   -2.27148   +4.26310   +3.87176
H   -0.70862   +3.55506   +4.24718
H   -1.78608   +2.75571   +3.11855
24
* E =   +3.387  kcal/mol  ;  (295)  060_176_260_060_179_295
H   +0.00000   +0.00000   +0.00000
O   +0.00000   +0.00000   +0.95450
C   -0.90201   +0.00000   +1.26665
```

```
C    −1.69110   −1.20434   +0.79812
H    −0.83867   +0.01758   +2.35131
H    −1.41842   +0.91543   +0.96245
C    −3.10085   −1.26024   +1.38468
H    −1.74042   −1.17519   −0.29188
H    −1.13539   −2.10735   +1.05463
C    −3.25676   −2.20747   +2.56485
H    −3.40563   −0.25572   +1.68970
H    −3.80726   −1.55932   +0.61232
C    −2.36853   −1.88703   +3.75258
H    −4.30128   −2.19978   +2.87565
H    −3.04526   −3.22645   +2.23167
C    −2.55646   −2.82293   +4.93630
H    −1.32410   −1.92827   +3.44137
H    −2.55313   −0.85858   +4.07481
C    −3.92472   −2.73556   +5.58509
H    −2.36990   −3.84875   +4.61366
H    −1.79497   −2.59849   +5.68282
H    −3.98095   −3.36744   +6.46848
H    −4.14362   −1.71462   +5.89537
H    −4.71682   −3.05189   +4.91036
24
* E =   +3.388  kcal/mol ; (296) 065_075_300_092_182_182
H    +0.00000   +0.00000   +0.00000
O    +0.00000   +0.00000   +0.95421
C    −0.90244   +0.00000   +1.26423
C    −1.67509   −1.25097   +0.89953
H    −0.84243   +0.10463   +2.34613
H    −1.42744   +0.88023   +0.88565
C    −1.34894   −2.51106   +1.68745
H    −2.73675   −1.03265   +1.02567
H    −1.53608   −1.44196   −0.16836
C    +0.09434   −3.00200   +1.61649
H    −1.61929   −2.35323   +2.73423
H    −2.01408   −3.29546   +1.32686
C    +0.99203   −2.46149   +2.71435
H    +0.10274   −4.09145   +1.67651
H    +0.52103   −2.74768   +0.64491
C    +2.42838   −2.92896   +2.58696
H    +0.97494   −1.37287   +2.70379
H    +0.59493   −2.77654   +3.68279
C    +3.31499   −2.42317   +3.70689
H    +2.45386   −4.02022   +2.56308
H    +2.82463   −2.59332   +1.62760
H    +4.34121   −2.76545   +3.59307
```

H +3.32888 −1.33492 +3.73012
H +2.95693 −2.76787 +4.67588
24
∗ E = +3.390 kcal/mol ; (297) 181_183_101_300_181_065
H +0.00000 +0.00000 +0.00000
O +0.00000 +0.00000 +0.95351
C −0.89745 +0.00000 +1.27565
C −0.85737 +0.03502 +2.78276
H −1.44147 +0.87561 +0.90968
H −1.43494 −0.88725 +0.93185
C −2.24579 −0.03396 +3.41600
H −0.23760 −0.79201 +3.13145
H −0.34110 +0.94700 +3.07653
C −2.63005 −1.40546 +3.95041
H −2.30790 +0.68005 +4.23530
H −2.99558 +0.28790 +2.68837
C −2.64760 −2.50552 +2.90553
H −1.93190 −1.68458 +4.74335
H −3.61049 −1.33054 +4.42039
C −3.04990 −3.86847 +3.44687
H −3.32830 −2.22457 +2.09703
H −1.65674 −2.58927 +2.45734
C −4.48029 −3.93983 +3.94655
H −2.91109 −4.61151 +2.66172
H −2.36764 −4.14834 +4.25142
H −4.73901 −4.95079 +4.25297
H −4.64525 −3.29011 +4.80287
H −5.18018 −3.64197 +3.16679
24
∗ E = +3.392 kcal/mol ; (298) 061_177_180_264_061_064
H +0.00000 +0.00000 +0.00000
O +0.00000 +0.00000 +0.95436
C −0.90198 +0.00000 +1.26620
C −1.68447 −1.21315 +0.81690
H −0.83664 +0.02043 +2.35177
H −1.41871 +0.91390 +0.95826
C −3.09038 −1.23944 +1.39109
H −1.71776 −1.23085 −0.27369
H −1.14338 −2.10827 +1.12520
C −3.92601 −2.45099 +0.98032
H −3.00725 −1.21046 +2.47809
H −3.61894 −0.32515 +1.10910
C −4.85065 −2.21522 −0.20606
H −3.26592 −3.29687 +0.78114
H −4.54709 −2.75713 +1.82113

C −4.17304 −1.84254 −1.51453
H −5.44938 −3.11376 −0.36664
H −5.55538 −1.42609 +0.06181
C −3.27391 −2.93095 −2.06793
H −4.94457 −1.61224 −2.24926
H −3.60652 −0.91896 −1.38460
H −2.84152 −2.64057 −3.02281
H −2.45218 −3.16230 −1.39319
H −3.83394 −3.85158 −2.22555
24
* E = +3.404 kcal/mol ; (299) 067_300_299_297_095_178
H +0.00000 +0.00000 +0.00000
O +0.00000 +0.00000 +0.95422
C −0.90122 +0.00000 +1.26781
C −1.63629 −1.28536 +0.94905
H −0.83697 +0.13082 +2.34467
H −1.44200 +0.86318 +0.87183
C −1.73592 −1.59371 −0.53626
H −1.11738 −2.10549 +1.44430
H −2.63337 −1.23776 +1.38917
C −2.48535 −0.57662 −1.38700
H −0.72944 −1.72489 −0.93916
H −2.21920 −2.56396 −0.65655
C −3.96388 −0.40942 −1.04115
H −1.99365 +0.39846 −1.33952
H −2.39640 −0.89520 −2.42489
C −4.28385 +0.73214 −0.09111
H −4.53013 −0.24342 −1.95852
H −4.34561 −1.34499 −0.62423
C −5.77102 +0.87531 +0.16233
H −3.76884 +0.59217 +0.85830
H −3.89381 +1.66044 −0.51228
H −5.98531 +1.70406 +0.83290
H −6.31063 +1.05222 −0.76671
H −6.18040 −0.02817 +0.61137
24
* E = +3.411 kcal/mol ; (300) 062_178_179_063_060_266
H +0.00000 +0.00000 +0.00000
O +0.00000 +0.00000 +0.95433
C −0.90197 +0.00000 +1.26609
C −1.67799 −1.22446 +0.83743
H −0.83635 +0.03647 +2.35150
H −1.42348 +0.90685 +0.94583
C −3.09515 −1.22938 +1.37826
H −1.71054 −1.26847 −0.25469

H −1.13215 −2.10776 +1.16681
C −3.91365 −2.43902 +0.95857
H −3.07411 −1.16135 +2.46757
H −3.60752 −0.32837 +1.03666
C −3.41292 −3.78950 +1.45257
H −4.93687 −2.30445 +1.31304
H −3.97252 −2.46304 −0.13117
C −3.34606 −3.93624 +2.97179
H −4.08316 −4.54821 +1.04960
H −2.43279 −4.00814 +1.02473
C −1.99415 −3.61327 +3.58308
H −4.11615 −3.31039 +3.42790
H −3.60070 −4.96068 +3.24120
H −2.00061 −3.77911 +4.65833
H −1.21841 −4.24820 +3.15781
H −1.69689 −2.58119 +3.41512
24
* E = +3.422 kcal/mol ; (301) 179_298_180_072_100_300
H +0.00000 +0.00000 +0.00000
O +0.00000 +0.00000 +0.95331
C −0.89772 +0.00000 +1.27408
C −0.86071 −0.03102 +2.78024
H −1.42731 +0.89277 +0.93345
H −1.43860 −0.87452 +0.90045
C −0.19929 −1.28019 +3.33203
H −0.32394 +0.85172 +3.13057
H −1.88420 +0.05612 +3.14694
C −0.16508 −1.31441 +4.85327
H −0.72448 −2.15887 +2.95245
H +0.81371 −1.33113 +2.93718
C −1.51830 −1.57357 +5.51795
H +0.54150 −2.07883 +5.18180
H +0.24506 −0.36642 +5.20342
C −1.72327 −3.01196 +5.96855
H −1.63559 −0.92603 +6.38606
H −2.32354 −1.29921 +4.83281
C −1.65829 −4.03198 +4.84892
H −0.96966 −3.25538 +6.71976
H −2.68765 −3.09075 +6.47102
H −1.85637 −5.03542 +5.21910
H −2.39308 −3.81273 +4.07500
H −0.67751 −4.04605 +4.37746
24
* E = +3.432 kcal/mol ; (302) 178_297_176_286_261_060
H +0.00000 +0.00000 +0.00000

O	+0.00000	+0.00000	+0.95328
C	−0.89713	+0.00000	+1.27562
C	−0.85467	−0.05520	+2.78130
H	−1.42234	+0.90104	+0.95050
H	−1.44379	−0.86537	+0.88931
C	−0.23079	−1.33308	+3.31044
H	−0.30028	+0.81353	+3.13703
H	−1.87310	+0.04415	+3.16049
C	−0.10059	−1.34858	+4.82707
H	−0.84251	−2.17807	+2.98849
H	+0.74547	−1.46177	+2.84642
C	+1.01035	−0.45043	+5.37380
H	−1.05999	−1.05697	+5.25716
H	+0.07486	−2.37153	+5.16544
C	+2.29311	−1.19231	+5.71720
H	+1.23804	+0.33357	+4.64889
H	+0.66439	+0.06305	+6.27018
C	+2.93098	−1.91091	+4.54450
H	+3.00780	−0.48356	+6.13624
H	+2.08147	−1.91321	+6.50931
H	+3.86887	−2.37947	+4.83454
H	+2.28242	−2.69332	+4.15469
H	+3.14044	−1.22150	+3.72787

24

* E = +3.440 kcal/mol ; (303) 063_053_058_171_060_265

H	+0.00000	+0.00000	+0.00000
O	+0.00000	+0.00000	+0.95458
C	−0.90156	+0.00000	+1.26829
C	−1.67092	−1.23785	+0.85670
H	−0.82994	+0.06119	+2.35101
H	−1.42899	+0.89570	+0.93171
C	−0.98709	−2.54197	+1.23240
H	−2.66018	−1.18924	+1.31627
H	−1.83662	−1.21355	−0.22280
C	−0.72850	−2.68538	+2.72050
H	−1.60459	−3.37367	+0.88872
H	−0.04177	−2.61166	+0.69486
C	−0.24049	−4.05981	+3.15182
H	+0.01229	−1.94456	+3.02564
H	−1.64474	−2.44913	+3.26689
C	+1.07673	−4.49901	+2.51305
H	−0.13069	−4.04850	+4.23576
H	−1.01108	−4.80386	+2.93625
C	+0.91029	−5.36552	+1.27764
H	+1.66816	−3.61497	+2.26750

H +1.66357 −5.05614 +3.24253
H +1.87440 −5.64209 +0.85584
H +0.38212 −6.28566 +1.52303
H +0.34175 −4.86193 +0.49989
24
* E = +3.443 kcal/mol ; (304) 068_297_182_096_298_296
H +0.00000 +0.00000 +0.00000
O +0.00000 +0.00000 +0.95379
C −0.90017 +0.00000 +1.26910
C −1.64019 −1.28569 +0.96964
H −0.83275 +0.14938 +2.34322
H −1.45044 +0.85141 +0.85690
C −1.79651 −1.55958 −0.51657
H −1.10363 −2.11429 +1.43350
H −2.61634 −1.23255 +1.45097
C −2.57540 −2.83071 −0.85264
H −2.27885 −0.70263 −0.99621
H −0.80077 −1.63490 −0.95559
C −4.05305 −2.61310 −1.14815
H −2.13352 −3.30083 −1.73019
H −2.45504 −3.55194 −0.04258
C −4.88217 −2.03204 −0.01451
H −4.13448 −1.95605 −2.01587
H −4.49263 −3.56606 −1.44855
C −4.96944 −2.93290 +1.20212
H −4.48494 −1.05641 +0.27001
H −5.88866 −1.84110 −0.38690
H −5.60371 −2.49874 +1.97160
H −5.38890 −3.90207 +0.93596
H −3.99270 −3.11270 +1.64676
24
* E = +3.444 kcal/mol ; (305) 066_298_184_064_059_265
H +0.00000 +0.00000 +0.00000
O +0.00000 +0.00000 +0.95393
C −0.90078 +0.00000 +1.26790
C −1.65160 −1.27182 +0.93928
H −0.83464 +0.12881 +2.34472
H −1.44504 +0.86304 +0.87059
C −1.79235 −1.51639 −0.55169
H −1.13884 −2.11671 +1.40043
H −2.63672 −1.20777 +1.40303
C −2.62584 −2.73753 −0.90477
H −2.21791 −0.63203 −1.03288
H −0.80047 −1.65107 −0.98672
C −4.09228 −2.68499 −0.49793

```
H   −2.57731   −2.89019   −1.98411
H   −2.16601   −3.61852   −0.45387
C   −4.88706   −1.52807   −1.10128
H   −4.54463   −3.62790   −0.80347
H   −4.18273   −2.65753   +0.58950
C   −4.97935   −0.29478   −0.22020
H   −4.45475   −1.25940   −2.06751
H   −5.90070   −1.86512   −1.31503
H   −5.58737   +0.47707   −0.68736
H   −5.43841   −0.54003   +0.73642
H   −4.00489   +0.14019   −0.01144
24
 * E =  +3.445 kcal/mol ; (306) 062_179_101_300_181_065
H   +0.00000   +0.00000   +0.00000
O   +0.00000   +0.00000   +0.95441
C   −0.90196   +0.00000   +1.26644
C   −1.67363   −1.23201   +0.84349
H   −0.83615   +0.03270   +2.35168
H   −1.42392   +0.90738   +0.95158
C   −3.11241   −1.24152   +1.35886
H   −1.67380   −1.30186   −0.24797
H   −1.12644   −2.10164   +1.20283
C   −4.15976   −0.84791   +0.32820
H   −3.35772   −2.23374   +1.73313
H   −3.19225   −0.57354   +2.22029
C   −3.99044   +0.54927   −0.23871
H   −4.13719   −1.56773   −0.49385
H   −5.14477   −0.93965   +0.78515
C   −5.05283   +0.94007   −1.25407
H   −3.99033   +1.27366   +0.58008
H   −3.01174   +0.62731   −0.71466
C   −6.45188   +1.04385   −0.67743
H   −4.77932   +1.89839   −1.69520
H   −5.04788   +0.21795   −2.07247
H   −7.15875   +1.39675   −1.42475
H   −6.81545   +0.08491   −0.31566
H   −6.47772   +1.74293   +0.15743
24
 * E =  +3.459 kcal/mol ; (307) 060_294_124_292_286_176
H   +0.00000   +0.00000   +0.00000
O   +0.00000   +0.00000   +0.95549
C   −0.90288   +0.00000   +1.26816
C   −1.68267   −1.21053   +0.80513
H   −0.83499   +0.01442   +2.35258
H   −1.41516   +0.91662   +0.96028
```

```
 C   −1.90846   −1.25774   −0.71034
 H   −1.14359   −2.10032   +1.13200
 H   −2.63906   −1.22198   +1.32697
 C   −1.39724   −2.53227   −1.36694
 H   −2.97005   −1.15012   −0.92578
 H   −1.43897   −0.39282   −1.18728
 C   +0.11793   −2.66419   −1.36469
 H   −1.83635   −3.38523   −0.84773
 H   −1.75827   −2.58392   −2.39543
 C   +0.81980   −1.74782   −2.35185
 H   +0.50271   −2.47459   −0.36035
 H   +0.39111   −3.69342   −1.60074
 C   +2.32965   −1.83048   −2.25212
 H   +0.49978   −2.00233   −3.36350
 H   +0.50491   −0.71208   −2.20382
 H   +2.81668   −1.18290   −2.97733
 H   +2.67111   −1.53833   −1.26048
 H   +2.67532   −2.84720   −2.43075
24
 * E =   +3.470  kcal/mol ;  (308) 180_303_296_179_059_262
 H   +0.00000   +0.00000   +0.00000
 O   +0.00000   +0.00000   +0.95330
 C   −0.89747   +0.00000   +1.27474
 C   −0.85550   +0.00207   +2.78312
 H   −1.43380   +0.88510   +0.92483
 H   −1.43572   −0.87771   +0.90888
 C   −0.08514   −1.16223   +3.38456
 H   −0.40278   +0.93842   +3.10774
 H   −1.88273   +0.00219   +3.15188
 C   −0.70222   −2.51975   +3.10641
 H   +0.93650   −1.14025   +3.00514
 H   −0.02525   −1.00716   +4.46187
 C   +0.06965   −3.69871   +3.68059
 H   −1.72043   −2.53631   +3.50461
 H   −0.79342   −2.66426   +2.02954
 C   +0.25445   −3.66786   +5.19772
 H   −0.45364   −4.61098   +3.39560
 H   +1.04923   −3.75333   +3.20088
 C   +1.59844   −3.12054   +5.64336
 H   −0.55048   −3.08249   +5.64718
 H   +0.14547   −4.67512   +5.59833
 H   +1.67530   −3.08821   +6.72830
 H   +2.40776   −3.74841   +5.27419
 H   +1.77199   −2.11537   +5.26712
24
```

```
 * E =   +3.474  kcal/mol  ;  (309)  064_302_301_090_058_054
 H    +0.00000    +0.00000    +0.00000
 O    +0.00000    +0.00000    +0.95520
 C    -0.90277    +0.00000    +1.26731
 C    -1.66758    -1.24509    +0.87542
 H    -0.83914    +0.06728    +2.35010
 H    -1.43158    +0.89367    +0.92446
 C    -1.74756    -1.51196    -0.62167
 H    -1.20609    -2.10344    +1.36281
 H    -2.67922    -1.15804    +1.27658
 C    -2.39118    -0.40411    -1.45230
 H    -0.75247    -1.74336    -1.00625
 H    -2.31851    -2.42962    -0.75621
 C    -1.45492    +0.67430    -1.98945
 H    -2.90274    -0.85438    -2.30229
 H    -3.17470    +0.07515    -0.86232
 C    -0.31896    +0.16861    -2.86916
 H    -2.05376    +1.37288    -2.57618
 H    -1.03915    +1.26794    -1.17450
 C    -0.78171    -0.67785    -4.03848
 H    +0.23552    +1.03004    -3.24001
 H    +0.39855    -0.40857    -2.28148
 H    +0.04697    -0.92416    -4.69769
 H    -1.22300    -1.61478    -3.70457
 H    -1.53123    -0.15210    -4.62873
24
 * E =   +3.479  kcal/mol  ;  (310)  182_062_178_061_260_288
 H    +0.00000    +0.00000    +0.00000
 O    +0.00000    +0.00000    +0.95328
 C    -0.89715    +0.00000    +1.27557
 C    -0.85432    +0.05163    +2.78166
 H    -1.44305    +0.86691    +0.89164
 H    -1.42299    -0.89997    +0.94846
 C    -0.20654    +1.31804    +3.30699
 H    -1.87504    -0.03274    +3.15982
 H    -0.31062    -0.82485    +3.13455
 C    -0.18489    +1.43215    +4.82330
 H    +0.81224    +1.36896    +2.92366
 H    -0.73328    +2.18016    +2.89238
 C    +0.58403    +0.31020    +5.52342
 H    +0.25129    +2.39605    +5.08194
 H    -1.21095    +1.46242    +5.19667
 C    -0.29441    -0.79114    +6.10184
 H    +1.29550    -0.12265    +4.81811
 H    +1.18485    +0.72243    +6.33517
```

```
 C    −1.05775   −0.35508   +7.33783
 H    −0.99801   −1.13699   +5.34409
 H    +0.32736   −1.65022   +6.35430
 H    −1.68686   −1.15576   +7.72026
 H    −0.37294   −0.06267   +8.13244
 H    −1.70268   +0.49746   +7.13291
24
 * E =  +3.495  kcal/mol ; (311) 181_058_066_092_298_298
 H    +0.00000   +0.00000   +0.00000
 O    +0.00000   +0.00000   +0.95327
 C    −0.89698   +0.00000   +1.27600
 C    −0.85672   +0.02440   +2.78405
 H    −1.44169   +0.86801   +0.89416
 H    −1.42815   −0.89431   +0.94191
 C    −0.11798   +1.22590   +3.35927
 H    −1.88101   −0.02812   +3.14990
 H    −0.35866   −0.88588   +3.11759
 C    −0.75504   +2.59246   +3.11743
 H    +0.00537   +1.07908   +4.43395
 H    +0.88344   +1.21519   +2.93389
 C    −1.69811   +3.06871   +4.21404
 H    +0.03762   +3.33420   +3.02648
 H    −1.26803   +2.60631   +2.15491
 C    −2.93128   +2.21328   +4.45502
 H    −1.13358   +3.13940   +5.14550
 H    −2.02128   +4.08478   +3.97860
 C    −3.85160   +2.11799   +3.25375
 H    −2.63053   +1.21514   +4.77540
 H    −3.48583   +2.63574   +5.29317
 H    −4.74342   +1.53839   +3.48142
 H    −4.17295   +3.10786   +2.93236
 H    −3.36234   +1.64482   +2.40401
24
 * E =  +3.522  kcal/mol ; (312) 178_176_060_263_286_178
 H    +0.00000   +0.00000   +0.00000
 O    +0.00000   +0.00000   +0.95352
 C    −0.89805   +0.00000   +1.27399
 C    −0.84793   −0.05453   +2.77888
 H    −1.42578   +0.90391   +0.95315
 H    −1.44501   −0.86103   +0.88176
 C    −2.20553   +0.03883   +3.45625
 H    −0.35275   −0.98151   +3.06996
 H    −0.20860   +0.75928   +3.11921
 C    −3.17775   −1.07994   +3.08029
 H    −2.03812   +0.03540   +4.53187
```

```
H   −2.65615   +1.00772   +3.23150
C   −4.16929   −0.71394   +1.98414
H   −2.60477   −1.95879   +2.77906
H   −3.74649   −1.38591   +3.95917
C   −5.27264   +0.21775   +2.45229
H   −3.64656   −0.25020   +1.14513
H   −4.62605   −1.62309   +1.58960
C   −6.22777   +0.60177   +1.34043
H   −5.82441   −0.26633   +3.25977
H   −4.83692   +1.11965   +2.88373
H   −7.01824   +1.25692   +1.69900
H   −5.70615   +1.12133   +0.53821
H   −6.69900   −0.27917   +0.90741
24
 * E =   +3.525  kcal/mol  ;  (313)  062_175_065_060_263_180
H   +0.00000   +0.00000   +0.00000
O   +0.00000   +0.00000   +0.95445
C   −0.90210   +0.00000   +1.26621
C   −1.66519   −1.23448   +0.84093
H   −0.83662   +0.03603   +2.35159
H   −1.41808   +0.90819   +0.94573
C   −3.06483   −1.32065   +1.42672
H   −1.71674   −1.27652   −0.25003
H   −1.08766   −2.10450   +1.15132
C   −4.05023   −0.25006   +0.97444
H   −3.48368   −2.29598   +1.17620
H   −2.99191   −1.29680   +2.51548
C   −4.31734   −0.22229   −0.52945
H   −4.98599   −0.42814   +1.50278
H   −3.71491   +0.73622   +1.30043
C   −3.50530   +0.80192   −1.30359
H   −4.13978   −1.21619   −0.94872
H   −5.37252   −0.01200   −0.70802
C   −3.81749   +0.79449   −2.78631
H   −3.70479   +1.79254   −0.89183
H   −2.43975   +0.62604   −1.15692
H   −3.24062   +1.54462   −3.32215
H   −3.59366   −0.17482   −3.22888
H   −4.87214   +0.99878   −2.96359
24
 * E =   +3.532  kcal/mol  ;  (314)  177_176_059_264_172_061
H   +0.00000   +0.00000   +0.00000
O   +0.00000   +0.00000   +0.95352
C   −0.89855   +0.00000   +1.27259
C   −0.85113   −0.07992   +2.77628
```

H	−1.42004	+0.91185	+0.96482
H	−1.44934	−0.85211	+0.86605
C	−2.20600	+0.01605	+3.45905
H	−0.36595	−1.01697	+3.05120
H	−0.20281	+0.72064	+3.13074
C	−3.20677	−1.06553	+3.05513
H	−2.03409	−0.02707	+4.53320
H	−2.64374	+0.99857	+3.26765
C	−4.16030	−0.63730	+1.95104
H	−2.66621	−1.96341	+2.74235
H	−3.79134	−1.35971	+3.92553
C	−5.03467	−1.75767	+1.41312
H	−4.79638	+0.16543	+2.33149
H	−3.59649	−0.20215	+1.12551
C	−5.95368	−2.37389	+2.45064
H	−5.63434	−1.37109	+0.58910
H	−4.39746	−2.53432	+0.98600
H	−6.61358	−3.11291	+2.00218
H	−5.39560	−2.87319	+3.23939
H	−6.57820	−1.61383	+2.91862

24

∗ E = +3.536 kcal/mol ; (315) 058_182_267_064_058_057

H	+0.00000	+0.00000	+0.00000
O	+0.00000	+0.00000	+0.95465
C	−0.90235	+0.00000	+1.26628
C	−1.69096	−1.18855	+0.75706
H	−0.83210	−0.00557	+2.34933
H	−1.41197	+0.92588	+0.98083
C	−3.15654	−1.21681	+1.18412
H	−1.63800	−1.16919	−0.33377
H	−1.18787	−2.10473	+1.06931
C	−3.44483	−1.99592	+2.45923
H	−3.53138	−0.19440	+1.27772
H	−3.74353	−1.66813	+0.38564
C	−2.82333	−1.44524	+3.73352
H	−4.52563	−2.05959	+2.58845
H	−3.10269	−3.02266	+2.32102
C	−3.22008	−0.01659	+4.07497
H	−3.11370	−2.09403	+4.56179
H	−1.73661	−1.51617	+3.67239
C	−4.71734	+0.18501	+4.20593
H	−2.73410	+0.26375	+5.00923
H	−2.83277	+0.67080	+3.32191
H	−4.95032	+1.18979	+4.55031
H	−5.22646	+0.04150	+3.25467

H −5.14765 −0.51807 +4.91812
24
∗ E = +3.542 kcal/mol ; (316) 295_299_175_060_261_288
H +0.00000 +0.00000 +0.00000
O +0.00000 +0.00000 +0.95451
C −0.90232 +0.00000 +1.26581
C −1.65442 +1.26076 +0.89860
H −1.43615 −0.87933 +0.89784
H −0.83484 −0.09173 +2.34786
C −1.03007 +2.51123 +1.48633
H −1.69942 +1.34667 −0.19139
H −2.68652 +1.15400 +1.23687
C −1.68026 +3.81254 +1.04440
H −1.07436 +2.44410 +2.57603
H +0.02770 +2.52533 +1.22683
C −3.15984 +3.93243 +1.41240
H −1.12168 +4.63544 +1.48813
H −1.55982 +3.92312 −0.03537
C −4.12506 +3.59561 +0.28337
H −3.36294 +3.28784 +2.26999
H −3.37722 +4.94715 +1.74836
C −4.16718 +4.65679 −0.79962
H −3.85592 +2.63628 −0.15924
H −5.12683 +3.47027 +0.69461
H −4.85469 +4.38389 −1.59701
H −4.49370 +5.61253 −0.39254
H −3.18897 +4.81155 −1.25087
24
∗ E = +3.544 kcal/mol ; (317) 183_185_301_097_181_067
H +0.00000 +0.00000 +0.00000
O +0.00000 +0.00000 +0.95349
C −0.89775 +0.00000 +1.27473
C −0.84465 +0.07614 +2.77861
H −1.44897 +0.85380 +0.87224
H −1.42153 −0.91039 +0.96645
C −2.19625 −0.03141 +3.46579
H −0.18970 −0.72133 +3.12780
H −0.36424 +1.01534 +3.05452
C −3.20757 +1.04167 +3.06513
H −2.62507 −1.01828 +3.27697
H −2.02167 +0.01484 +4.53939
C −4.17592 +0.60035 +1.97965
H −3.79481 +1.33737 +3.93548
H −2.66969 +1.93816 +2.75113
C −5.16418 +1.67290 +1.55260

```
H   −3.62601   +0.25323   +1.10285
H   −4.72697   −0.26667   +2.34740
C   −4.52404   +2.85264   +0.84562
H   −5.90660   +1.22414   +0.89280
H   −5.71165   +2.02524   +2.42865
H   −5.27471   +3.56051   +0.50177
H   −3.84147   +3.39488   +1.49581
H   −3.95838   +2.52358   −0.02543
24
 * E =  +3.548 kcal/mol ; (318) 058_181_299_098_074_182
H   +0.00000   +0.00000   +0.00000
O   +0.00000   +0.00000   +0.95452
C   −0.90214   +0.00000   +1.26638
C   −1.69085   −1.18756   +0.76268
H   −0.83197   −0.02067   +2.35086
H   −1.40905   +0.93103   +0.99236
C   −3.13959   −1.21992   +1.22496
H   −1.66630   −1.18382   −0.32978
H   −1.18059   −2.09738   +1.08094
C   −3.30988   −1.28559   +2.74335
H   −3.66306   −0.34513   +0.83392
H   −3.61798   −2.08091   +0.76141
C   −3.58129   +0.05714   +3.40801
H   −4.13195   −1.95726   +2.99298
H   −2.41717   −1.73723   +3.17958
C   −4.98746   +0.57723   +3.16987
H   −3.42123   −0.03293   +4.48354
H   −2.86356   +0.80146   +3.05777
C   −5.22787   +1.93057   +3.80763
H   −5.18194   +0.64199   +2.09857
H   −5.70327   −0.14671   +3.56278
H   −6.24360   +2.27957   +3.63700
H   −5.06809   +1.88996   +4.88386
H   −4.54831   +2.67955   +3.40438
24
 * E =  +3.548 kcal/mol ; (319) 301_179_060_264_173_062
H   +0.00000   +0.00000   +0.00000
O   +0.00000   +0.00000   +0.95451
C   −0.90208   +0.00000   +1.26652
C   −1.68326   +1.19994   +0.78098
H   −1.41361   −0.92415   +0.97880
H   −0.83122   +0.00463   +2.35126
C   −3.13493   +1.23662   +1.23405
H   −1.16899   +2.10066   +1.11778
H   −1.65283   +1.21608   −0.31118
```

C −3.32732 +1.25352 +2.74997
H −3.59509 +2.12034 +0.79521
H −3.67066 +0.38229 +0.81385
C −3.59016 −0.11845 +3.35005
H −2.44704 +1.69623 +3.22428
H −4.15981 +1.90930 +2.99998
C −3.61306 −0.13964 +4.86926
H −4.54569 −0.48788 +2.97012
H −2.83795 −0.82476 +2.99842
C −4.70306 +0.71955 +5.48121
H −3.73958 −1.16915 +5.20374
H −2.64093 +0.18354 +5.24587
H −4.72976 +0.60900 +6.56275
H −4.55442 +1.77550 +5.26697
H −5.68282 +0.43928 +5.09611
24
* E = +3.550 kcal/mol ; (320) 175_058_060_263_290_295
H +0.00000 +0.00000 +0.00000
O +0.00000 +0.00000 +0.95344
C −0.89744 +0.00000 +1.27539
C −0.86231 −0.13385 +2.77652
H −1.40765 +0.92557 +0.99249
H −1.46132 −0.83188 +0.84776
C −0.08655 +0.96547 +3.48620
H −1.89264 −0.14563 +3.13636
H −0.42801 −1.10217 +3.02391
C −0.63735 +2.37170 +3.25188
H −0.09115 +0.73818 +4.55132
H +0.95417 +0.91454 +3.16925
C +0.07724 +3.15577 +2.15786
H −1.69902 +2.30250 +3.00437
H −0.59638 +2.94332 +4.17842
C +1.49281 +3.58942 +2.50961
H +0.11833 +2.55146 +1.25262
H −0.50822 +4.04577 +1.91797
C +1.57019 +4.57863 +3.65679
H +2.09938 +2.71323 +2.73972
H +1.94462 +4.03707 +1.62439
H +2.59237 +4.91351 +3.81840
H +0.96176 +5.45949 +3.45495
H +1.22107 +4.14590 +4.59179
24
* E = +3.561 kcal/mol ; (321) 059_181_301_097_181_067
H +0.00000 +0.00000 +0.00000
O +0.00000 +0.00000 +0.95452

C	−0.90252	+0.00000	+1.26527
C	−1.68698	−1.19135	+0.76388
H	−0.83334	−0.01820	+2.34998
H	−1.41020	+0.92944	+0.98793
C	−3.14093	−1.22402	+1.20931
H	−1.65104	−1.19700	−0.32825
H	−1.17895	−2.09797	+1.09437
C	−3.34117	−1.24875	+2.72414
H	−3.67063	−0.36567	+0.78983
H	−3.60267	−2.10393	+0.76450
C	−3.62011	+0.11686	+3.33082
H	−4.17800	−1.90244	+2.97313
H	−2.46364	−1.69839	+3.19222
C	−3.80036	+0.10209	+4.83971
H	−2.82017	+0.81369	+3.07430
H	−4.52359	+0.51682	+2.86766
C	−2.54281	−0.26201	+5.60616
H	−4.13944	+1.08658	+5.16150
H	−4.60090	−0.59328	+5.09866
H	−2.70168	−0.18967	+6.67960
H	−2.21749	−1.27806	+5.39509
H	−1.72262	+0.40704	+5.34878

24

* E = +3.566 kcal/mol ; (322) 067_300_093_075_186_298

H	+0.00000	+0.00000	+0.00000
O	+0.00000	+0.00000	+0.95442
C	−0.90193	+0.00000	+1.26657
C	−1.65209	−1.27994	+0.96430
H	−0.84093	+0.15343	+2.34079
H	−1.44766	+0.85094	+0.84746
C	−1.75245	−1.58906	−0.52781
H	−1.16806	−2.10516	+1.48655
H	−2.64804	−1.18096	+1.39661
C	−0.64678	−2.49283	−1.06195
H	−2.71053	−2.06260	−0.74412
H	−1.76477	−0.64779	−1.08438
C	−0.83258	−3.94750	−0.66664
H	−0.61856	−2.41587	−2.14864
H	+0.32361	−2.15130	−0.69670
C	+0.32289	−4.85230	−1.06132
H	−0.97594	−4.01284	+0.41251
H	−1.75501	−4.32135	−1.11799
C	+0.55246	−4.94085	−2.55801
H	+1.23352	−4.50179	−0.57288
H	+0.13481	−5.85182	−0.66963

```
H   +1.33385   -5.65947   -2.79411
H   -0.35378   -5.25690   -3.07346
H   +0.85428   -3.98431   -2.97877
24
 * E =  +3.568  kcal/mol ; (323) 066_302_303_188_098_301
H   +0.00000   +0.00000   +0.00000
O   +0.00000   +0.00000   +0.95458
C   -0.90186   +0.00000   +1.26742
C   -1.64998   -1.26969   +0.91664
H   -0.83845   +0.10519   +2.34696
H   -1.43770   +0.87557   +0.89199
C   -1.71169   -1.56543   -0.57311
H   -1.17589   -2.10595   +1.42906
H   -2.66366   -1.18587   +1.31358
C   -2.33540   -0.45831   -1.40861
H   -0.70623   -1.77128   -0.94810
H   -2.26458   -2.49126   -0.72365
C   -2.56041   -0.84376   -2.87024
H   -3.28589   -0.15605   -0.96043
H   -1.69940   +0.42709   -1.36213
C   -3.97700   -1.29743   -3.18873
H   -2.32540   +0.00262   -3.51433
H   -1.85770   -1.63399   -3.14480
C   -4.44668   -2.50294   -2.39884
H   -4.65932   -0.46454   -3.01008
H   -4.04254   -1.51819   -4.25417
H   -5.44458   -2.80733   -2.70600
H   -3.78152   -3.35325   -2.54392
H   -4.48611   -2.29148   -1.33170
24
 * E =  +3.568  kcal/mol ; (324) 065_303_302_183_261_059
H   +0.00000   +0.00000   +0.00000
O   +0.00000   +0.00000   +0.95497
C   -0.90304   +0.00000   +1.26560
C   -1.65836   -1.25904   +0.89232
H   -0.84248   +0.08779   +2.34689
H   -1.43229   +0.88477   +0.90277
C   -1.69087   -1.54782   -0.60027
H   -1.20557   -2.10432   +1.40918
H   -2.67894   -1.16486   +1.26859
C   -2.30015   -0.43699   -1.44109
H   -0.68339   -1.77765   -0.95054
H   -2.26500   -2.46017   -0.76452
C   -2.40544   -0.77083   -2.92916
H   -3.29014   -0.21462   -1.04069
```

H −1.72588 +0.48676 −1.32556
C −1.29155 −0.19454 −3.78980
H −2.43049 −1.85576 −3.05266
H −3.35431 −0.40409 −3.31830
C +0.10274 −0.64254 −3.40004
H −1.47633 −0.46702 −4.82885
H −1.34228 +0.89488 −3.74976
H +0.84849 −0.25782 −4.09149
H +0.37501 −0.28477 −2.40791
H +0.18311 −1.72844 −3.39157
24
* E = +3.569 kcal/mol ; (325) 179_174_092_295_302_303
H +0.00000 +0.00000 +0.00000
O +0.00000 +0.00000 +0.95353
C −0.89798 +0.00000 +1.27422
C −0.84301 −0.03722 +2.78176
H −1.42312 +0.90550 +0.95458
H −1.44453 −0.85691 +0.87777
C −2.19430 +0.09551 +3.47828
H −0.34642 −0.95726 +3.09271
H −0.18878 +0.77893 +3.08483
C −2.89792 −1.21581 +3.79712
H −2.04531 +0.62160 +4.42001
H −2.85226 +0.73891 +2.88869
C −3.32188 −2.05502 +2.60174
H −2.24009 −1.81655 +4.42696
H −3.77455 −0.99847 +4.40824
C −4.25299 −1.35284 +1.62477
H −2.43723 −2.40863 +2.07048
H −3.82007 −2.95220 +2.97356
C −5.52976 −0.84000 +2.26237
H −3.73477 −0.52063 +1.14633
H −4.50286 −2.04782 +0.82312
H −6.20914 −0.43721 +1.51485
H −6.05214 −1.63670 +2.79056
H −5.32869 −0.04711 +2.98031
24
* E = +3.576 kcal/mol ; (326) 062_053_055_058_064_270
H +0.00000 +0.00000 +0.00000
O +0.00000 +0.00000 +0.95457
C −0.90185 +0.00000 +1.26741
C −1.67453 −1.23440 +0.85326
H −0.83056 +0.05639 +2.35109
H −1.42743 +0.89838 +0.93510
C −0.98267 −2.53899 +1.21692

H −2.66246 −1.18008 +1.31290
H −1.84503 −1.20801 −0.22547
C −0.61745 −2.67306 +2.68665
H −1.61034 −3.37496 +0.90801
H −0.06507 −2.62043 +0.63551
C −1.76073 −2.55988 +3.68536
H −0.12896 −3.63782 +2.83250
H +0.13696 −1.92262 +2.92536
C −2.81882 −3.65782 +3.60398
H −1.31919 −2.57506 +4.68156
H −2.24614 −1.58454 +3.59661
C −3.98220 −3.37056 +2.67171
H −2.33948 −4.59567 +3.31614
H −3.22426 −3.82660 +4.60129
H −4.72848 −4.16054 +2.72573
H −4.47346 −2.43750 +2.94525
H −3.67357 −3.28704 +1.63336
24
* E = +3.576 kcal/mol ; (327) 060_175_284_261_060_177
H +0.00000 +0.00000 +0.00000
O +0.00000 +0.00000 +0.95442
C −0.90197 +0.00000 +1.26646
C −1.68839 −1.20527 +0.79845
H −0.83453 +0.01379 +2.35149
H −1.41759 +0.91718 +0.96595
C −3.08684 −1.25727 +1.39797
H −1.75616 −1.17534 −0.29131
H −1.12636 −2.10255 +1.05417
C −3.12851 −1.70540 +2.86005
H −3.53935 −0.26933 +1.29875
H −3.71183 −1.92587 +0.80462
C −3.47883 −3.17306 +3.04924
H −2.16519 −1.50610 +3.33405
H −3.85732 −1.10897 +3.40712
C −2.51466 −4.14934 +2.40208
H −3.53367 −3.38873 +4.11779
H −4.48178 −3.35193 +2.65380
C −2.87393 −5.59391 +2.68655
H −2.49734 −3.98791 +1.32352
H −1.50260 −3.94525 +2.75696
H −2.17737 −6.28139 +2.21251
H −2.86326 −5.79632 +3.75626
H −3.87192 −5.82790 +2.31920
24
* E = +3.579 kcal/mol ; (328) 299_304_301_097_071_066

```
H    +0.00000   +0.00000   +0.00000
O    +0.00000   +0.00000   +0.95457
C    -0.90155   +0.00000   +1.26828
C    -1.68337   +1.21810   +0.82872
H    -1.41884   -0.90953   +0.95396
H    -0.83142   -0.03294   +2.35336
C    -1.05858   +2.54582   +1.22944
H    -1.80946   +1.18792   -0.25636
H    -2.68726   +1.14336   +1.25168
C    -0.88214   +2.71825   +2.73778
H    -0.09296   +2.64528   +0.73388
H    -1.68525   +3.34316   +0.83238
C    +0.51528   +2.38690   +3.24842
H    -1.13112   +3.73956   +3.02354
H    -1.61040   +2.08993   +3.25521
C    +1.58465   +3.39751   +2.86018
H    +0.48776   +2.31417   +4.33746
H    +0.81400   +1.40891   +2.87395
C    +1.39696   +4.76837   +3.48159
H    +2.55496   +3.00175   +3.15913
H    +1.62661   +3.49107   +1.77436
H    +2.22029   +5.43186   +3.22641
H    +0.48048   +5.24744   +3.14365
H    +1.35097   +4.70120   +4.56784
24
* E =   +3.581  kcal/mol ; (329) 180_058_060_063_268_295
H    +0.00000   +0.00000   +0.00000
O    +0.00000   +0.00000   +0.95332
C    -0.89776   +0.00000   +1.27403
C    -0.85562   +0.00929   +2.78186
H    -1.43467   +0.87759   +0.90345
H    -1.43311   -0.88678   +0.92647
C    -0.09570   +1.19065   +3.36208
H    -1.87798   -0.01668   +3.15943
H    -0.37996   -0.91351   +3.11325
C    -0.62883   +2.57121   +3.00712
H    -0.08144   +1.09111   +4.44868
H    +0.93872   +1.12173   +3.02980
C    -2.03647   +2.88048   +3.51420
H    +0.06159   +3.29883   +3.43238
H    -0.57055   +2.72235   +1.92859
C    -3.18714   +2.57033   +2.56471
H    -2.19514   +2.34784   +4.45432
H    -2.09901   +3.94144   +3.76186
C    -3.17973   +3.41095   +1.30234
```

```
H   −3.19041   +1.51455   +2.29709
H   −4.12353   +2.74344   +3.09578
H   −4.04730   +3.19783   +0.68184
H   −3.19627   +4.47325   +1.54186
H   −2.29408   +3.22811   +0.69649
24
 * E =  +3.608  kcal/mol ; (330) 180_181_184_300_099_072
H   +0.00000   +0.00000   +0.00000
O   +0.00000   +0.00000   +0.95349
C   −0.89776   +0.00000   +1.27468
C   −0.86040   +0.00384   +2.78039
H   −1.43774   +0.88110   +0.91655
H   −1.43631   −0.88424   +0.92176
C   −2.24809   −0.01559   +3.39108
H   −0.28530   −0.86186   +3.10764
H   −0.31428   +0.88798   +3.11168
C   −2.26971   +0.07657   +4.90911
H   −2.83225   +0.81017   +2.98042
H   −2.76101   −0.93115   +3.08703
C   −1.53698   −1.06419   +5.61630
H   −1.84138   +1.03354   +5.21311
H   −3.31019   +0.10281   +5.22986
C   −0.12388   −0.72415   +6.07116
H   −2.10546   −1.38172   +6.49145
H   −1.50762   −1.93030   +4.95232
C   −0.09244   +0.21891   +7.25873
H   +0.39655   −1.64442   +6.33649
H   +0.43525   −0.28652   +5.24436
H   +0.92830   +0.45138   +7.55343
H   −0.59050   +1.16148   +7.03928
H   −0.59379   −0.22315   +8.11853
24
 * E =  +3.609  kcal/mol ; (331) 057_292_128_289_180_294
H   +0.00000   +0.00000   +0.00000
O   +0.00000   +0.00000   +0.95543
C   −0.90264   +0.00000   +1.26862
C   −1.70127   −1.17109   +0.74126
H   −0.83439   −0.04412   +2.35214
H   −1.39927   +0.94032   +1.00986
C   −1.95305   −1.11107   −0.76912
H   −1.16177   −2.08334   +0.99722
H   −2.64926   −1.21405   +1.27633
C   −1.55046   −2.37408   −1.51326
H   −3.00465   −0.90025   −0.95523
H   −1.41273   −0.26590   −1.20826
```

C	−0.04594	−2.56309	−1.58821
H	−2.01453	−3.23206	−1.02484
H	−1.95202	−2.34474	−2.52749
C	+0.39221	−3.81208	−2.33565
H	+0.38454	−1.68876	−2.08370
H	+0.37801	−2.58021	−0.58198
C	−0.00518	−5.10724	−1.65342
H	−0.01986	−3.78972	−3.34602
H	+1.47567	−3.78898	−2.44975
H	+0.39050	−5.96890	−2.18605
H	+0.37874	−5.14372	−0.63487
H	−1.08519	−5.22529	−1.60327

24

* E = +3.616 kcal/mol ; (332) 180_177_284_261_060_177

H	+0.00000	+0.00000	+0.00000
O	+0.00000	+0.00000	+0.95348
C	−0.89751	+0.00000	+1.27536
C	−0.85530	+0.00913	+2.78236
H	−1.43974	+0.87504	+0.90709
H	−1.43388	−0.88914	+0.93125
C	−2.24253	+0.09099	+3.40265
H	−0.34361	−0.89482	+3.10990
H	−0.24127	+0.85056	+3.10031
C	−2.88078	+1.48044	+3.34795
H	−2.88726	−0.63432	+2.90371
H	−2.19100	−0.23308	+4.44284
C	−2.74802	+2.27517	+4.63770
H	−2.44108	+2.05536	+2.53002
H	−3.94041	+1.39103	+3.11195
C	−1.32155	+2.55207	+5.07358
H	−3.27237	+3.22580	+4.52401
H	−3.26467	+1.73986	+5.43823
C	−1.25252	+3.40574	+6.32388
H	−0.80216	+1.60972	+5.25101
H	−0.78560	+3.04600	+4.26076
H	−0.22465	+3.59089	+6.62680
H	−1.73084	+4.37102	+6.16589
H	−1.76066	+2.92117	+7.15617

24

* E = +3.619 kcal/mol ; (333) 065_296_270_065_057_057

H	+0.00000	+0.00000	+0.00000
O	+0.00000	+0.00000	+0.95445
C	−0.90127	+0.00000	+1.26861
C	−1.65288	−1.26565	+0.90328
H	−0.82925	+0.07492	+2.35045

```
H   -1.42621   +0.89097   +0.92068
C   -1.85673   -1.51823   -0.58797
H   -1.09579   -2.09656   +1.33427
H   -2.62400   -1.24971   +1.40269
C   -3.12666   -0.93407   -1.18951
H   -0.98580   -1.17479   -1.15337
H   -1.88315   -2.59435   -0.75301
C   -3.23771   +0.58334   -1.19347
H   -3.22172   -1.29872   -2.21279
H   -3.98278   -1.34276   -0.65037
C   -2.10015   +1.30875   -1.89575
H   -4.17534   +0.85356   -1.68234
H   -3.32309   +0.94997   -0.17014
C   -1.90229   +0.88317   -3.33761
H   -2.29486   +2.38019   -1.85759
H   -1.16778   +1.16405   -1.34673
H   -1.14563   +1.49107   -3.82779
H   -1.58463   -0.15530   -3.41110
H   -2.82673   +0.98342   -3.90476
24
* E =  +3.628  kcal/mol ; (334) 296_300_179_072_100_300
H   +0.00000   +0.00000   +0.00000
O   +0.00000   +0.00000   +0.95453
C   -0.90242   +0.00000   +1.26559
C   -1.66119   +1.25115   +0.88156
H   -1.43178   -0.88687   +0.90900
H   -0.83439   -0.07749   +2.34864
C   -1.04203   +2.51930   +1.43832
H   -1.71249   +1.31885   -0.20915
H   -2.69172   +1.14384   +1.22399
C   -1.78343   +3.78266   +1.02533
H   -1.00771   +2.44673   +2.52661
H   -0.00665   +2.57204   +1.10496
C   -3.13862   +3.98075   +1.70674
H   -1.15589   +4.65225   +1.22886
H   -1.91272   +3.76133   -0.05769
C   -3.10905   +4.95390   +2.87550
H   -3.86815   +4.34173   +0.98279
H   -3.51927   +3.01864   +2.05649
C   -2.18305   +4.54584   +4.00429
H   -2.81334   +5.93758   +2.50617
H   -4.12108   +5.06856   +3.26437
H   -2.23755   +5.25016   +4.83130
H   -2.44400   +3.56095   +4.38993
H   -1.14578   +4.50746   +3.67712
```

```
24
 * E =   +3.629  kcal/mol ;  (335) 063_173_094_295_301_303
 H    +0.00000    +0.00000    +0.00000
 O    +0.00000    +0.00000    +0.95437
 C    −0.90171    +0.00000    +1.26701
 C    −1.65051    −1.25360    +0.86508
 H    −0.83468    +0.05237    +2.35172
 H    −1.42182    +0.89931    +0.93278
 C    −3.04599    −1.38971    +1.47105
 H    −1.72136    −1.30045    −0.22537
 H    −1.03002    −2.09633    +1.16640
 C    −4.18432    −0.86716    +0.60678
 H    −3.23867    −2.44378    +1.66443
 H    −3.06852    −0.90622    +2.45074
 C    −4.18172    +0.62901    +0.33503
 H    −4.16292    −1.39449    −0.34837
 H    −5.13016    −1.14221    +1.07420
 C    −4.24014    +1.50598    +1.57680
 H    −3.30193    +0.89360    −0.25388
 H    −5.04010    +0.86288    −0.29721
 C    −5.44125    +1.23061    +2.46066
 H    −3.32948    +1.38478    +2.16447
 H    −4.25355    +2.54954    +1.26287
 H    −5.50065    +1.94648    +3.27700
 H    −6.36940    +1.29555    +1.89413
 H    −5.39496    +0.23706    +2.90257
24
 * E =   +3.631  kcal/mol ;  (336) 299_183_184_300_099_072
 H    +0.00000    +0.00000    +0.00000
 O    +0.00000    +0.00000    +0.95436
 C    −0.90204    +0.00000    +1.26601
 C    −1.68275    +1.21714    +0.82402
 H    −1.42049    −0.91110    +0.95298
 H    −0.83694    −0.02626    +2.35141
 C    −3.08960    +1.23554    +1.39063
 H    −1.13317    +2.10659    +1.13117
 H    −1.73009    +1.23863    −0.26862
 C    −3.95371    +2.38935    +0.90584
 H    −3.58631    +0.29513    +1.14463
 H    −3.03181    +1.27171    +2.48082
 C    −3.40116    +3.77298    +1.25009
 H    −4.08820    +2.30443    −0.17430
 H    −4.94646    +2.26984    +1.33730
 C    −2.65530    +4.45845    +0.11301
 H    −4.21523    +4.42843    +1.56200
```

```
 H    −2.74336    +3.68517    +2.11684
 C    −3.57292    +4.94574    −0.99209
 H    −2.09975    +5.30727    +0.51154
 H    −1.91241    +3.77850    −0.30426
 H    −3.01167    +5.42224    −1.79259
 H    −4.14238    +4.13013    −1.43386
 H    −4.28744    +5.67311    −0.60973
24
 ∗ E =   +3.638  kcal/mol  ;  (337) 068_296_289_261_060_177
 H    +0.00000    +0.00000    +0.00000
 O    +0.00000    +0.00000    +0.95382
 C    −0.90007    +0.00000    +1.26948
 C    −1.63616    −1.28935    +0.96844
 H    −0.83142    +0.13922    +2.34487
 H    −1.44495    +0.85945    +0.86989
 C    −1.82982    −1.56076    −0.51752
 H    −1.07413    −2.10712    +1.41846
 H    −2.60434    −1.25324    +1.46878
 C    −2.83658    −0.64299    −1.21270
 H    −0.85875    −1.49814    −1.01182
 H    −2.14175    −2.59745    −0.65243
 C    −4.21830    −1.25444    −1.38578
 H    −2.92996    +0.29294    −0.65784
 H    −2.46462    −0.36611    −2.19842
 C    −4.91255    −1.64224    −0.09390
 H    −4.85154    −0.55000    −1.92804
 H    −4.13722    −2.13886    −2.02227
 C    −6.31475    −2.16900    −0.32468
 H    −4.32456    −2.39981    +0.42562
 H    −4.94998    −0.77590    +0.56967
 H    −6.79683    −2.44938    +0.60884
 H    −6.93941    −1.42023    −0.80886
 H    −6.30042    −3.04818    −0.96681
24
 ∗ E =   +3.639  kcal/mol  ;  (338) 292_060_267_285_182_295
 H    +0.00000    +0.00000    +0.00000
 O    +0.00000    +0.00000    +0.95440
 C    −0.90225    +0.00000    +1.26556
 C    −1.65022    +1.28179    +0.96592
 H    −1.44824    −0.84890    +0.84271
 H    −0.84289    −0.15705    +2.33932
 C    −1.74183    +1.59896    −0.52539
 H    −2.64880    +1.18060    +1.39154
 H    −1.16963    +2.10430    +1.49519
 C    −0.63676    +2.51088    −1.04576
```

```
H   −1.74297   +0.66081   −1.08756
H   −2.70156   +2.06704   −0.74606
C   −0.82138   +3.96150   −0.63574
H   +0.32954   +2.15308   −0.69113
H   −0.61039   +2.46071   −2.13594
C   +0.24146   +4.90109   −1.18175
H   −1.80252   +4.29566   −0.97816
H   −0.83719   +4.03922   +0.45319
C   +1.63472   +4.63470   −0.64452
H   +0.25120   +4.83656   −2.27135
H   −0.04185   +5.92602   −0.94268
H   +2.34387   +5.37468   −1.00834
H   +1.64623   +4.67239   +0.44379
H   +2.00512   +3.65637   −0.94152
24
 * E =   +3.640  kcal/mol ; (339) 299_182_177_060_261_287
H   +0.00000   +0.00000   +0.00000
O   +0.00000   +0.00000   +0.95436
C   −0.90169   +0.00000   +1.26702
C   −1.68215   +1.21776   +0.82656
H   −1.42134   −0.91042   +0.95370
H   −0.83475   −0.02761   +2.35217
C   −3.09716   +1.22357   +1.37283
H   −1.14740   +2.11136   +1.15123
H   −1.70724   +1.24654   −0.26548
C   −3.90484   +2.46555   +1.02873
H   −3.62796   +0.34364   +1.00188
H   −3.05779   +1.11824   +2.45841
C   −4.09055   +2.69592   −0.47149
H   −4.87656   +2.37899   +1.51270
H   −3.42602   +3.33725   +1.47860
C   −3.13470   +3.71479   −1.07794
H   −3.98582   +1.74149   −0.99141
H   −5.10952   +3.02981   −0.67099
C   −3.45838   +5.14247   −0.68095
H   −2.11095   +3.48053   −0.78556
H   −3.16793   +3.63370   −2.16452
H   −2.75319   +5.84548   −1.11822
H   −4.45621   +5.42112   −1.01635
H   −3.42672   +5.27815   +0.39845
24
 * E =   +3.643  kcal/mol ; (340) 297_303_293_178_059_263
H   +0.00000   +0.00000   +0.00000
O   +0.00000   +0.00000   +0.95459
C   −0.90184   +0.00000   +1.26752
```

```
C   -1.66840   +1.23929   +0.85517
H   -1.42992   -0.89293   +0.92440
H   -0.83168   -0.06884   +2.34976
C   -1.03352   +2.54526   +1.30436
H   -1.76802   +1.24090   -0.23282
H   -2.68351   +1.16267   +1.25039
C   -1.05093   +2.74744   +2.80794
H   -0.00415   +2.58277   +0.94522
H   -1.56406   +3.36768   +0.82453
C   -0.38172   +4.02766   +3.28516
H   -2.08824   +2.74095   +3.15311
H   -0.56143   +1.90435   +3.29541
C   -0.97957   +5.31521   +2.71837
H   -0.43906   +4.04715   +4.37293
H   +0.68237   +3.98807   +3.04273
C   -0.22805   +5.86945   +1.52142
H   -2.02397   +5.14080   +2.45081
H   -0.99788   +6.07995   +3.49401
H   -0.70347   +6.76841   +1.13434
H   +0.79239   +6.12931   +1.79817
H   -0.16887   +5.14868   +0.70942
24
* E =  +3.648  kcal/mol ; (341) 066_302_302_189_300_095
H   +0.00000   +0.00000   +0.00000
O   +0.00000   +0.00000   +0.95464
C   -0.90200   +0.00000   +1.26727
C   -1.64584   -1.27515   +0.92744
H   -0.83946   +0.11486   +2.34588
H   -1.44049   +0.87034   +0.88384
C   -1.70075   -1.58434   -0.56073
H   -1.17112   -2.10511   +1.44934
H   -2.66105   -1.18984   +1.31972
C   -2.33395   -0.48535   -1.39577
H   -0.69154   -1.79247   -0.92177
H   -2.25881   -2.51037   -0.70591
C   -2.58879   -0.85202   -2.84953
H   -3.27870   -0.19110   -0.93413
H   -1.70301   +0.40738   -1.37344
C   -1.33955   -1.22633   -3.64612
H   -3.30311   -1.67721   -2.89315
H   -3.08127   -0.00456   -3.32493
C   -1.06494   -2.71793   -3.71210
H   -1.43579   -0.85232   -4.66460
H   -0.47444   -0.70960   -3.22406
H   -0.16072   -2.92861   -4.27911
```

```
H    −0.94542    −3.15660    −2.72480
H    −1.88810    −3.23627    −4.20101
24
* E =   +3.649  kcal/mol ;  (342) 293_063_176_287_260_060
H    +0.00000    +0.00000    +0.00000
O    +0.00000    +0.00000    +0.95392
C    −0.90036    +0.00000    +1.26905
C    −1.64526    +1.27735    +0.94900
H    −1.44655    −0.85957    +0.86859
H    −0.83236    −0.13367    +2.34522
C    −1.80459    +1.52906    −0.53958
H    −2.62602    +1.22441    +1.42436
H    −1.11770    +2.11606    +1.40473
C    −2.64174    +2.76226    −0.85289
H    −0.81731    +1.65503    −0.98755
H    −2.25301    +0.65019    −1.00838
C    −4.14075    +2.59154    −0.60219
H    −2.25595    +3.59012    −0.25740
H    −2.48818    +3.05068    −1.89419
C    −4.95075    +2.30625    −1.85774
H    −4.30442    +1.78568    +0.11652
H    −4.54197    +3.48996    −0.13523
C    −4.54865    +1.03891    −2.58600
H    −6.00586    +2.24739    −1.59025
H    −4.85750    +3.15560    −2.53685
H    −5.19002    +0.85941    −3.44581
H    −3.52456    +1.09476    −2.95068
H    −4.62029    +0.17034    −1.93227
24
* E =   +3.663  kcal/mol ;  (343) 180_180_182_073_100_300
H    +0.00000    +0.00000    +0.00000
O    +0.00000    +0.00000    +0.95348
C    −0.89784    +0.00000    +1.27443
C    −0.86060    +0.00390    +2.77998
H    −1.43722    +0.88169    +0.91674
H    −1.43634    −0.88355    +0.91990
C    −2.24667    −0.00608    +3.39664
H    −0.29845    −0.86812    +3.11494
H    −0.29895    +0.87933    +3.10475
C    −2.22304    +0.04579    +4.91828
H    −2.82287    +0.83642    +3.01040
H    −2.77040    −0.90807    +3.07572
C    −1.83969    +1.40697    +5.50092
H    −3.20276    −0.24038    +5.30519
H    −1.53064    −0.71870    +5.27188
```

C −3.02575 +2.23969 +5.96292
H −1.16934 +1.27043 +6.34839
H −1.27117 +1.97583 +4.76240
C −4.02051 +2.57115 +4.86795
H −3.53928 +1.70602 +6.76478
H −2.65581 +3.16535 +6.40417
H −4.82103 +3.20503 +5.24265
H −3.53790 +3.09768 +4.04533
H −4.48050 +1.67275 +4.46035
24
* E = +3.663 kcal/mol ; (344) 295_299_175_286_260_060
H +0.00000 +0.00000 +0.00000
O +0.00000 +0.00000 +0.95444
C −0.90216 +0.00000 +1.26600
C −1.64956 +1.26537 +0.90597
H −1.43827 −0.87582 +0.89296
H −0.83400 −0.09870 +2.34737
C −1.03367 +2.51254 +1.51139
H −1.69130 +1.35424 −0.18280
H −2.68473 +1.16502 +1.23849
C −1.71003 +3.79767 +1.05505
H −1.09444 +2.43387 +2.59807
H +0.02706 +2.53554 +1.26556
C −1.37702 +4.21207 −0.37923
H −2.78844 +3.67637 +1.16635
H −1.43438 +4.61097 +1.72875
C −0.30819 +5.28968 −0.47990
H −1.05297 +3.33773 −0.94826
H −2.27439 +4.57508 −0.87881
C +1.02886 +4.90051 +0.11922
H −0.16985 +5.55201 −1.52906
H −0.67346 +6.19298 +0.01222
H +1.76587 +5.68800 −0.02099
H +0.94963 +4.71455 +1.18858
H +1.41896 +3.99451 −0.34284
24
* E = +3.670 kcal/mol ; (345) 061_178_182_073_100_300
H +0.00000 +0.00000 +0.00000
O +0.00000 +0.00000 +0.95434
C −0.90182 +0.00000 +1.26655
C −1.68131 −1.21744 +0.82462
H −0.83502 +0.02627 +2.35171
H −1.42022 +0.91137 +0.95420
C −3.09824 −1.23206 +1.36819
H −1.71660 −1.24554 −0.26786

H −1.13635 −2.10686 +1.13985
C −3.88447 −2.47171 +0.96361
H −3.06641 −1.15639 +2.45616
H −3.62116 −0.34177 +1.01512
C −3.46317 −3.75363 +1.68364
H −4.94735 −2.29946 +1.14235
H −3.78383 −2.60005 −0.11472
C −4.36403 −4.13512 +2.84828
H −3.44735 −4.58459 +0.97971
H −2.43790 −3.65211 +2.04536
C −4.43712 −3.09503 +3.94873
H −5.36906 −4.32600 +2.46753
H −4.01543 −5.07871 +3.26828
H −5.05580 −3.43953 +4.77424
H −3.44742 −2.87322 +4.34624
H −4.86578 −2.16132 +3.58910
24
∗ E = +3.677 kcal/mol ; (346) 299_180_284_261_061_177
H +0.00000 +0.00000 +0.00000
O +0.00000 +0.00000 +0.95440
C −0.90229 +0.00000 +1.26546
C −1.67805 +1.22100 +0.82141
H −1.41640 −0.91470 +0.95785
H −0.83854 −0.02048 +2.35116
C −3.11843 +1.20890 +1.31451
H −1.15557 +2.10374 +1.18767
H −1.66102 +1.27671 −0.26917
C −4.04513 +0.26568 +0.54465
H −3.11430 +0.94446 +2.37286
H −3.52498 +2.21998 +1.26834
C −4.89395 +0.95583 −0.51153
H −3.45702 −0.52252 +0.06937
H −4.71334 −0.24240 +1.23848
C −4.10942 +1.65605 −1.60504
H −5.55721 +0.22021 −0.97009
H −5.54457 +1.68460 −0.02198
C −5.00620 +2.26639 −2.66338
H −3.48532 +2.43619 −1.16740
H −3.42554 +0.94292 −2.07029
H −4.43011 +2.76762 −3.43760
H −5.61741 +1.50497 −3.14504
H −5.68171 +2.99998 −2.22637
24
∗ E = +3.679 kcal/mol ; (347) 067_295_180_287_260_060
H +0.00000 +0.00000 +0.00000

```
O    +0.00000    +0.00000    +0.95385
C    -0.90046    +0.00000    +1.26849
C    -1.64494    -1.27786    +0.94760
H    -0.83264    +0.13193    +2.34485
H    -1.44614    +0.86058    +0.86946
C    -1.85021    -1.50312    -0.53941
H    -1.09985    -2.11340    +1.38642
H    -2.61837    -1.24089    +1.44066
C    -2.59792    -2.79140    -0.85635
H    -2.40052    -0.65380    -0.94977
H    -0.88322    -1.52326    -1.04557
C    -1.77692    -4.06586    -0.65277
H    -3.49498    -2.82248    -0.23704
H    -2.95188    -2.75976    -1.88835
C    -1.19959    -4.64238    -1.93653
H    -0.96191    -3.87028    +0.04677
H    -2.39352    -4.83111    -0.18332
C    -0.26204    -3.70711    -2.67463
H    -0.67213    -5.56675    -1.70099
H    -2.02161    -4.92254    -2.59778
H    +0.15858    -4.18662    -3.55554
H    -0.77629    -2.80767    -3.00906
H    +0.56618    -3.39873    -2.03761
24
 * E =   +3.681  kcal/mol ; (348) 301_178_062_171_262_059
H    +0.00000    +0.00000    +0.00000
O    +0.00000    +0.00000    +0.95451
C    -0.90209    +0.00000    +1.26647
C    -1.69215    +1.19081    +0.76966
H    -1.41137    -0.92715    +0.98416
H    -0.83059    +0.01000    +2.35037
C    -3.14539    +1.18945    +1.21211
H    -1.19730    +2.10222    +1.10710
H    -1.65226    +1.20194    -0.32205
C    -3.33814    +1.25580    +2.71964
H    -3.65573    +2.02995    +0.74418
H    -3.64143    +0.29257    +0.83388
C    -4.79112    +1.46892    +3.14269
H    -2.96245    +0.33576    +3.16553
H    -2.71820    +2.05856    +3.12823
C    -5.14700    +2.91263    +3.46371
H    -5.45194    +1.10046    +2.35440
H    -5.01008    +0.86447    +4.02194
C    -4.93666    +3.88414    +2.31926
H    -6.18820    +2.95458    +3.78385
```

```
H    −4.55389   +3.23634   +4.32079
H    −5.25100   +4.88814   +2.59509
H    −3.88870   +3.94017   +2.02997
H    −5.50797   +3.58787   +1.44034
24
* E =  +3.682 kcal/mol ; (349) 299_186_299_098_074_181
H    +0.00000   +0.00000   +0.00000
O    +0.00000   +0.00000   +0.95447
C    −0.90217   +0.00000   +1.26608
C    −1.66654   +1.22719   +0.82305
H    −1.41625   −0.91022   +0.94758
H    −0.83807   −0.02916   +2.35216
C    −3.06174   +1.35029   +1.41586
H    −1.07471   +2.10269   +1.08849
H    −1.74322   +1.22128   −0.26793
C    −4.00175   +0.19567   +1.06648
H    −2.98185   +1.44439   +2.50049
H    −3.48738   +2.29063   +1.06991
C    −4.10943   −0.87990   +2.13879
H    −5.00374   +0.58153   +0.87543
H    −3.67562   −0.25454   +0.12689
C    −4.91917   −0.44956   +3.34872
H    −4.57074   −1.77163   +1.71131
H    −3.11360   −1.18357   +2.46717
C    −4.98885   −1.52064   +4.41826
H    −4.49422   +0.46024   +3.77433
H    −5.92773   −0.18802   +3.02421
H    −5.58293   −1.19834   +5.27018
H    −5.43620   −2.43387   +4.02912
H    −3.99520   −1.77349   +4.78444
24
* E =  +3.683 kcal/mol ; (350) 292_064_181_300_100_072
H    +0.00000   +0.00000   +0.00000
O    +0.00000   +0.00000   +0.95376
C    −0.89975   +0.00000   +1.27017
C    −1.64098   +1.28560   +0.97186
H    −1.45106   −0.85146   +0.85950
H    −0.83033   −0.14863   +2.34422
C    −1.82922   +1.54240   −0.51114
H    −2.61374   +1.23263   +1.46309
H    −1.09845   +2.11660   +1.42517
C    −2.54429   +2.84351   −0.84372
H    −0.85447   +1.55600   −1.00290
H    −2.38671   +0.70957   −0.94842
C    −3.95730   +2.94585   −0.26784
```

```
H   -1.94006   +3.67991   -0.48777
H   -2.57766   +2.94288   -1.92768
C   -4.06109   +3.76672   +1.01079
H   -4.62635   +3.38773   -1.00720
H   -4.34033   +1.93939   -0.08797
C   -3.89847   +5.25623   +0.77527
H   -5.03097   +3.58548   +1.47417
H   -3.31251   +3.42914   +1.72788
H   -3.96612   +5.81472   +1.70599
H   -2.93635   +5.49089   +0.32384
H   -4.67297   +5.62962   +0.10704
24
* E =   +3.683  kcal/mol  ;  (351)  294_064_175_061_261_288
H   +0.00000   +0.00000   +0.00000
O   +0.00000   +0.00000   +0.95393
C   -0.90059   +0.00000   +1.26845
C   -1.64762   +1.27384   +0.93644
H   -1.44445   -0.86407   +0.87423
H   -0.83332   -0.12456   +2.34571
C   -1.81963   +1.49461   -0.55470
H   -2.62944   +1.22961   +1.41278
H   -1.11386   +2.11334   +1.38171
C   -2.67025   +2.70026   -0.92526
H   -0.83741   +1.61693   -1.01813
H   -2.26313   +0.59910   -0.99677
C   -2.11385   +4.03482   -0.42684
H   -2.76905   +2.72283   -2.00962
H   -3.68065   +2.55407   -0.53854
C   -2.75767   +4.55204   +0.85268
H   -1.03696   +3.93575   -0.27866
H   -2.23769   +4.79664   -1.19746
C   -4.17272   +5.05687   +0.64504
H   -2.75889   +3.76700   +1.60895
H   -2.14882   +5.36115   +1.25598
H   -4.60885   +5.40994   +1.57668
H   -4.18901   +5.88385   -0.06323
H   -4.82600   +4.27915   +0.25371
24
* E =   +3.685  kcal/mol  ;  (352)  060_174_059_264_173_062
H   +0.00000   +0.00000   +0.00000
O   +0.00000   +0.00000   +0.95452
C   -0.90209   +0.00000   +1.26651
C   -1.67400   -1.21323   +0.79928
H   -0.83709   +0.00835   +2.35279
H   -1.41004   +0.91944   +0.96452
```

```
C   -3.06932   -1.34904   +1.38974
H   -1.75317   -1.18117   -0.29104
H   -1.08502   -2.09716   +1.04216
C   -4.00638   -0.17679   +1.10166
H   -3.50241   -2.26952   +1.00181
H   -2.99208   -1.48939   +2.47016
C   -4.06178   +0.85277   +2.21895
H   -3.70498   +0.31203   +0.17067
H   -5.01226   -0.55449   +0.92486
C   -4.84683   +2.10666   +1.87216
H   -4.50202   +0.38459   +3.10247
H   -3.04995   +1.13814   +2.50705
C   -6.30911   +1.85174   +1.55979
H   -4.77585   +2.80645   +2.70460
H   -4.37412   +2.60025   +1.02098
H   -6.84486   +2.78418   +1.39792
H   -6.43233   +1.24713   +0.66407
H   -6.79754   +1.32838   +2.38091
24
 * E =   +3.686  kcal/mol ; (353) 299_187_301_097_181_067
H   +0.00000   +0.00000   +0.00000
O   +0.00000   +0.00000   +0.95450
C   -0.90238   +0.00000   +1.26559
C   -1.66952   +1.21960   +0.80660
H   -1.41291   -0.91523   +0.95520
H   -0.83906   -0.01755   +2.35185
C   -3.06294   +1.35716   +1.40068
H   -1.07623   +2.09941   +1.05396
H   -1.75017   +1.19461   -0.28377
C   -4.00433   +0.18924   +1.10882
H   -2.98260   +1.49396   +2.48127
H   -3.49483   +2.27989   +1.01676
C   -4.07789   -0.83698   +2.22781
H   -5.01307   +0.56537   +0.93369
H   -3.70066   -0.28987   +0.17600
C   -4.99165   -2.01488   +1.93306
H   -3.07951   -1.21041   +2.46234
H   -4.42673   -0.33188   +3.12988
C   -4.50148   -2.90717   +0.80829
H   -5.09690   -2.61177   +2.83876
H   -5.99084   -1.64432   +1.69702
H   -5.14749   -3.77298   +0.68265
H   -4.47467   -2.38296   -0.14431
H   -3.49600   -3.27328   +1.01255
24
```

```
 * E =  +3.687  kcal/mol ; (354) 300_183_183_073_100_300
 H   +0.00000   +0.00000   +0.00000
 O   +0.00000   +0.00000   +0.95440
 C   -0.90234   +0.00000   +1.26530
 C   -1.68729   +1.20781   +0.80662
 H   -1.41650   -0.91703   +0.96265
 H   -0.83770   -0.01339   +2.35094
 C   -3.09721   +1.23433   +1.36754
 H   -1.15013   +2.10787   +1.10692
 H   -1.72091   +1.21577   -0.28562
 C   -3.92186   +2.41172   +0.86516
 H   -3.60360   +0.29972   +1.12067
 H   -3.03932   +1.27242   +2.45631
 C   -4.37965   +2.29148   -0.58927
 H   -4.79966   +2.53540   +1.50180
 H   -3.33177   +3.31915   +0.99641
 C   -5.81875   +1.82555   -0.74954
 H   -4.27520   +3.25273   -1.09061
 H   -3.72219   +1.60423   -1.12630
 C   -6.10311   +0.46069   -0.15415
 H   -6.47973   +2.56249   -0.28984
 H   -6.06969   +1.81440   -1.81033
 H   -7.13207   +0.15910   -0.33618
 H   -5.45353   -0.29975   -0.58626
 H   -5.94800   +0.45389   +0.92319
24
 * E =  +3.693  kcal/mol ; (355) 064_291_262_061_173_063
 H   +0.00000   +0.00000   +0.00000
 O   +0.00000   +0.00000   +0.95428
 C   -0.90090   +0.00000   +1.26896
 C   -1.65344   -1.25885   +0.88667
 H   -0.82769   +0.07193   +2.35083
 H   -1.42662   +0.89031   +0.91722
 C   -1.95322   -1.39318   -0.60594
 H   -1.05457   -2.10219   +1.22713
 H   -2.58868   -1.29481   +1.44913
 C   -3.35399   -0.96109   -1.01084
 H   -1.22468   -0.82067   -1.18839
 H   -1.81343   -2.42829   -0.91269
 C   -3.67314   +0.49649   -0.73220
 H   -3.48890   -1.15271   -2.07730
 H   -4.07313   -1.59903   -0.49464
 C   -5.00795   +0.95324   -1.29843
 H   -3.66717   +0.67517   +0.34520
 H   -2.87885   +1.11931   -1.14940
```

```
 C    -6.20499   +0.23637   -0.70371
 H    -5.11294   +2.02467   -1.12906
 H    -5.00249   +0.81631   -2.38116
 H    -7.13764   +0.64964   -1.08048
 H    -6.20054   -0.82487   -0.94175
 H    -6.21641   +0.33121   +0.38144
24
 * E =   +3.705  kcal/mol  ;  (356) 295_300_181_299_100_073
 H    +0.00000   +0.00000   +0.00000
 O    +0.00000   +0.00000   +0.95450
 C    -0.90215   +0.00000   +1.26625
 C    -1.65248   +1.26150   +0.89779
 H    -1.43640   -0.87911   +0.89823
 H    -0.83385   -0.09154   +2.34823
 C    -1.02389   +2.51272   +1.47919
 H    -1.70181   +1.33564   -0.19156
 H    -2.68453   +1.16529   +1.24272
 C    -1.76310   +3.80231   +1.15770
 H    -0.96020   +2.39866   +2.56287
 H    +0.00427   +2.58515   +1.12399
 C    -1.86573   +4.10961   -0.33731
 H    -2.76405   +3.76038   +1.59255
 H    -1.25383   +4.61812   +1.66863
 C    -3.20910   +3.75818   -0.96285
 H    -1.68000   +5.16999   -0.51230
 H    -1.06797   +3.58031   -0.86210
 C    -4.32018   +4.70604   -0.55357
 H    -3.11450   +3.77161   -2.04883
 H    -3.48555   +2.73845   -0.69302
 H    -5.26998   +4.42255   -1.00125
 H    -4.45932   +4.72077   +0.52580
 H    -4.09552   +5.72410   -0.86786
24
 * E =   +3.708  kcal/mol  ;  (357) 292_060_266_288_293_183
 H    +0.00000   +0.00000   +0.00000
 O    +0.00000   +0.00000   +0.95440
 C    -0.90224   +0.00000   +1.26560
 C    -1.64821   +1.28409   +0.97012
 H    -1.44927   -0.84679   +0.83991
 H    -0.84312   -0.16064   +2.33885
 C    -1.73928   +1.60625   -0.52022
 H    -2.64665   +1.18362   +1.39630
 H    -1.16553   +2.10319   +1.50284
 C    -0.63787   +2.52782   -1.03400
 H    -1.72265   +0.67109   -1.08720
```

H −2.70661 +2.05283 −0.74456
C −0.76438 +3.97582 −0.58420
H +0.33056 +2.14879 −0.70818
H −0.63177 +2.50397 −2.12521
C −1.95738 +4.71349 −1.16422
H −0.80581 +4.02604 +0.50562
H +0.14443 +4.50807 −0.86905
C −1.99313 +6.17205 −0.75350
H −1.92776 +4.63994 −2.25289
H −2.88422 +4.23177 −0.85282
H −2.85158 +6.68744 −1.17789
H −2.04923 +6.27193 +0.32925
H −1.09647 +6.69381 −1.08387
24
* E = +3.710 kcal/mol ; (358) 177_174_061_171_262_059
H +0.00000 +0.00000 +0.00000
O +0.00000 +0.00000 +0.95350
C −0.89796 +0.00000 +1.27417
C −0.85065 −0.06989 +2.77933
H −1.42027 +0.91121 +0.96580
H −1.44843 −0.85189 +0.86908
C −2.21012 +0.06565 +3.44135
H −0.38493 −1.01273 +3.06814
H −0.18895 +0.72292 +3.12580
C −3.20439 −1.02265 +3.06522
H −2.07197 +0.07159 +4.52150
H −2.64306 +1.03733 +3.19260
C −4.48262 −1.00244 +3.90267
H −3.46062 −0.91921 +2.01128
H −2.72295 −2.00015 +3.15901
C −4.49186 −1.99086 +5.05895
H −4.64185 +0.00712 +4.28900
H −5.34168 −1.21806 +3.26850
C −3.37424 −1.79636 +6.06440
H −5.45183 −1.92305 +5.57115
H −4.43609 −3.00286 +4.65393
H −3.46124 −2.50160 +6.88783
H −2.39671 −1.94672 +5.60975
H −3.39282 −0.79177 +6.48515
24
* E = +3.718 kcal/mol ; (359) 059_182_294_182_262_059
H +0.00000 +0.00000 +0.00000
O +0.00000 +0.00000 +0.95450
C −0.90222 +0.00000 +1.26606
C −1.68770 −1.19909 +0.78119

H	−0.83109	+0.00298	+2.34990
H	−1.41483	+0.92193	+0.97291
C	−3.14048	−1.20067	+1.22609
H	−1.65070	−1.21627	−0.31049
H	−1.18693	−2.10581	+1.12246
C	−3.33290	−1.35772	+2.72658
H	−3.62822	−0.28818	+0.88007
H	−3.65826	−2.02123	+0.72828
C	−4.79787	−1.39703	+3.16038
H	−2.83095	−2.27587	+3.03412
H	−2.82725	−0.55052	+3.26027
C	−5.34027	−0.07245	+3.67519
H	−5.41185	−1.73695	+2.32306
H	−4.92593	−2.14125	+3.94541
C	−5.26334	+1.06880	+2.68050
H	−6.37701	−0.21369	+3.98105
H	−4.79361	+0.20324	+4.57869
H	−5.72061	+1.97036	+3.08174
H	−4.23203	+1.31200	+2.43095
H	−5.77865	+0.81928	+1.75374

24

∗ E = +3.724 kcal/mol ; (360) 064_059_072_097_298_295

H	+0.00000	+0.00000	+0.00000
O	+0.00000	+0.00000	+0.95455
C	−0.90147	+0.00000	+1.26844
C	−1.65754	−1.25642	+0.88984
H	−0.82946	+0.09323	+2.34963
H	−1.43651	+0.88083	+0.90587
C	−1.02308	−2.52998	+1.43088
H	−2.68660	−1.15445	+1.23483
H	−1.70988	−1.31214	−0.20028
C	−1.15671	−2.73116	+2.93984
H	−1.45595	−3.39135	+0.91918
H	+0.03021	−2.51014	+1.15594
C	−2.31720	−3.62568	+3.35393
H	−0.24115	−3.17861	+3.32371
H	−1.23477	−1.76547	+3.44153
C	−3.70303	−3.12332	+2.98270
H	−2.16779	−4.60896	+2.90432
H	−2.27824	−3.77757	+4.43434
C	−4.09373	−1.83684	+3.68416
H	−3.76934	−2.99477	+1.90114
H	−4.43041	−3.89600	+3.23195
H	−5.10182	−1.52882	+3.41598
H	−4.06227	−1.96108	+4.76564

H −3.42402 −1.01599 +3.43389
24
∗ E = +3.743 kcal/mol ; (361) 065_302_305_301_296_091
H +0.00000 +0.00000 +0.00000
O +0.00000 +0.00000 +0.95459
C −0.90193 +0.00000 +1.26727
C −1.65451 −1.26559 +0.91296
H −0.83869 +0.10264 +2.34704
H −1.43696 +0.87785 +0.89391
C −1.70698 −1.55378 −0.58030
H −1.18347 −2.10683 +1.42014
H −2.66415 −1.17988 +1.31674
C −2.26934 −0.42608 −1.43328
H −0.70069 −1.79023 −0.93127
H −2.28333 −2.46290 −0.74948
C −3.68601 +0.02398 −1.10522
H −1.61104 +0.44379 −1.36761
H −2.23893 −0.73527 −2.47918
C −4.77291 −1.02695 −1.32178
H −3.73540 +0.39033 −0.07724
H −3.90299 +0.88689 −1.73417
C −5.09505 −1.88574 −0.11189
H −5.68912 −0.52238 −1.62676
H −4.49263 −1.66483 −2.16250
H −5.92804 −2.55376 −0.32044
H −4.25582 −2.50175 +0.19898
H −5.37860 −1.26443 +0.73659
24
∗ E = +3.762 kcal/mol ; (362) 059_182_297_189_300_095
H +0.00000 +0.00000 +0.00000
O +0.00000 +0.00000 +0.95452
C −0.90223 +0.00000 +1.26611
C −1.69188 −1.19113 +0.76948
H −0.83156 −0.00920 +2.35006
H −1.41157 +0.92688 +0.98299
C −3.14622 −1.18573 +1.21052
H −1.65063 −1.20303 −0.32214
H −1.19906 −2.10276 +1.10879
C −3.32991 −1.27329 +2.71577
H −3.63160 −0.28366 +0.83264
H −3.65905 −2.02636 +0.74078
C −4.76433 −1.49173 +3.17148
H −2.71029 −2.08710 +3.09791
H −2.95470 −0.36187 +3.18477
C −5.74331 −0.39238 +2.76077

H −5.12226 −2.45183 +2.79253
H −4.75735 −1.58285 +4.25706
C −6.50359 −0.68127 +1.47881
H −6.46873 −0.23893 +3.55883
H −5.20379 +0.55232 +2.66578
H −7.17798 +0.13446 +1.22677
H −5.83767 −0.83266 +0.63307
H −7.10400 −1.58331 +1.58529
24
* E = +3.764 kcal/mol ; (363) 183_186_295_183_262_059
H +0.00000 +0.00000 +0.00000
O +0.00000 +0.00000 +0.95350
C −0.89780 +0.00000 +1.27462
C −0.84907 +0.07905 +2.77956
H −1.45038 +0.84744 +0.86355
H −1.41761 −0.91480 +0.97267
C −2.20605 −0.06395 +3.44562
H −0.18268 −0.70921 +3.12742
H −0.38710 +1.02508 +3.06374
C −3.18096 +1.06175 +3.13603
H −2.64613 −1.02455 +3.17413
H −2.05947 −0.09992 +4.52564
C −4.52104 +0.93714 +3.86044
H −2.70113 +2.00374 +3.40491
H −3.36458 +1.11903 +2.06093
C −5.63967 +0.33661 +3.02263
H −4.38647 +0.33798 +4.76398
H −4.84545 +1.92005 +4.20002
C −5.35509 −1.05704 +2.49866
H −6.55227 +0.31711 +3.61857
H −5.84300 +1.00080 +2.18065
H −6.21175 −1.45669 +1.96067
H −4.51013 −1.06043 +1.81240
H −5.12326 −1.74394 +3.31164
24
* E = +3.775 kcal/mol ; (364) 298_304_300_297_092_064
H +0.00000 +0.00000 +0.00000
O +0.00000 +0.00000 +0.95459
C −0.90187 +0.00000 +1.26745
C −1.66972 +1.23605 +0.84971
H −1.42762 −0.89790 +0.93341
H −0.82898 −0.05812 +2.35096
C −1.01349 +2.53948 +1.27366
H −1.77862 +1.23201 −0.23766
H −2.68346 +1.16865 +1.24652

```
C   -0.80735   +2.72737   +2.76991
H   -0.04343   +2.61209   +0.78317
H   -1.61407   +3.36854   +0.89591
C   -2.08470   +2.76669   +3.60695
H   -0.12743   +1.96273   +3.14591
H   -0.27578   +3.66908   +2.90189
C   -2.56365   +1.44018   +4.18425
H   -1.93613   +3.44868   +4.44568
H   -2.88482   +3.20621   +3.00731
C   -1.60550   +0.83031   +5.18942
H   -3.52393   +1.60899   +4.67231
H   -2.76004   +0.72211   +3.38921
H   -2.01529   -0.08001   +5.62099
H   -0.64894   +0.57298   +4.73849
H   -1.40505   +1.52305   +6.00554
24
* E =  +3.782 kcal/mol ; (365) 177_174_062_170_060_265
H   +0.00000   +0.00000   +0.00000
O   +0.00000   +0.00000   +0.95351
C   -0.89812   +0.00000   +1.27377
C   -0.85119   -0.06492   +2.77917
H   -1.42114   +0.90971   +0.96229
H   -1.44756   -0.85363   +0.87107
C   -2.21214   +0.07791   +3.43832
H   -0.38891   -1.00786   +3.07240
H   -0.18787   +0.72777   +3.12271
C   -3.19250   -1.02467   +3.07708
H   -2.07521   +0.08971   +4.52065
H   -2.63764   +1.04849   +3.17566
C   -4.47450   -1.03300   +3.89512
H   -3.45884   -0.94724   +2.02100
H   -2.69369   -1.98920   +3.19303
C   -5.31721   +0.23674   +3.78095
H   -5.06690   -1.89002   +3.57632
H   -4.23204   -1.21033   +4.94549
C   -5.06472   +1.25355   +4.87965
H   -5.14452   +0.69628   +2.80541
H   -6.37361   -0.02877   +3.79959
H   -5.68190   +2.13977   +4.74741
H   -5.30185   +0.83009   +5.85431
H   -4.02676   +1.57493   +4.90894
24
* E =  +3.791 kcal/mol ; (366) 065_301_299_179_059_262
H   +0.00000   +0.00000   +0.00000
O   +0.00000   +0.00000   +0.95438
```

C	−0.90115	+0.00000	+1.26866
C	−1.65141	−1.26826	+0.91781
H	−0.83491	+0.09928	+2.34865
H	−1.43547	+0.87932	+0.90070
C	−1.74128	−1.55999	−0.57254
H	−1.16319	−2.10509	+1.41584
H	−2.65779	−1.19369	+1.33444
C	−2.46326	−0.49316	−1.37522
H	−0.73811	−1.71231	−0.98005
H	−2.25251	−2.51358	−0.70074
C	−2.54756	−0.76781	−2.86940
H	−3.47477	−0.37950	−0.97775
H	−1.97790	+0.47379	−1.23139
C	−3.25324	−2.07137	−3.24272
H	−3.06253	+0.07255	−3.33330
H	−1.54084	−0.77149	−3.29379
C	−2.30822	−3.22627	−3.52116
H	−3.94873	−2.34574	−2.44682
H	−3.86725	−1.91096	−4.12793
H	−2.85322	−4.13842	−3.75467
H	−1.66717	−2.99900	−4.37134
H	−1.65968	−3.43547	−2.67368

24

∗ E = +3.801 kcal/mol ; (367) 296_070_100_300_181_065

H	+0.00000	+0.00000	+0.00000
O	+0.00000	+0.00000	+0.95433
C	−0.90106	+0.00000	+1.26872
C	−1.65507	+1.25536	+0.87830
H	−1.42576	−0.89232	+0.91917
H	−0.82825	−0.06786	+2.35085
C	−1.95919	+1.37109	−0.61519
H	−2.58798	+1.29725	+1.44409
H	−1.05543	+2.10232	+1.20807
C	−3.37542	+0.97752	−1.00687
H	−1.78536	+2.39358	−0.94566
H	−1.25535	+0.76064	−1.18949
C	−3.74755	−0.45650	−0.67860
H	−4.07840	+1.64784	−0.50606
H	−3.49913	+1.15166	−2.07547
C	−5.15432	−0.84450	−1.10639
H	−3.02913	−1.13366	−1.14882
H	−3.65676	−0.61440	+0.39663
C	−5.36798	−0.84499	−2.60805
H	−5.37642	−1.83765	−0.71658
H	−5.87007	−0.16822	−0.63574

H −6.36422 −1.19951 −2.86202
H −5.25998 +0.14904 −3.03572
H −4.64968 −1.49727 −3.10307
24
∗ E = +3.802 kcal/mol ; (368) 062_173_063_171_262_059
H +0.00000 +0.00000 +0.00000
O +0.00000 +0.00000 +0.95443
C −0.90230 +0.00000 +1.26554
C −1.66753 −1.22969 +0.82778
H −0.83822 +0.02861 +2.35149
H −1.41446 +0.91092 +0.94795
C −3.05085 −1.34838 +1.44449
H −1.75738 −1.22757 −0.26216
H −1.07011 −2.10234 +1.08929
C −4.00567 −0.22365 +1.07291
H −3.48462 −2.30289 +1.15004
H −2.95945 −1.38722 +2.53222
C −5.44541 −0.46839 +1.52364
H −3.64354 +0.70745 +1.50673
H −3.98470 −0.07463 −0.01052
C −6.35768 −1.02335 +0.44024
H −5.44246 −1.14982 +2.37762
H −5.88028 +0.46211 +1.88625
C −5.91062 −2.34886 −0.14374
H −7.36242 −1.13173 +0.84901
H −6.43561 −0.28727 −0.36190
H −6.62543 −2.71469 −0.87733
H −4.94781 −2.26114 −0.64417
H −5.81308 −3.10829 +0.63106
24
∗ E = +3.832 kcal/mol ; (369) 299_186_292_182_262_059
H +0.00000 +0.00000 +0.00000
O +0.00000 +0.00000 +0.95444
C −0.90224 +0.00000 +1.26574
C −1.66956 +1.22712 +0.82368
H −1.41270 −0.91349 +0.95308
H −0.83720 −0.02310 +2.35167
C −3.06166 +1.33389 +1.42348
H −1.08060 +2.10121 +1.09897
H −1.74390 +1.23177 −0.26742
C −4.03786 +0.27144 +0.94151
H −2.98866 +1.31035 +2.51158
H −3.47457 +2.31229 +1.17545
C −5.44405 +0.41472 +1.52291
H −4.08164 +0.32707 −0.14717

H	−3.65883	−0.72600	+1.17272
C	−5.73153	−0.48800	+2.71297
H	−5.60878	+1.45540	+1.81170
H	−6.18176	+0.19914	+0.75092
C	−4.80439	−0.28677	+3.89517
H	−6.76146	−0.32838	+3.03251
H	−5.67437	−1.52792	+2.38650
H	−5.09744	−0.91333	+4.73446
H	−3.77622	−0.54145	+3.64446
H	−4.81612	+0.74851	+4.23370

24

∗ E = +3.839 kcal/mol ; (370) 067_300_297_267_062_062

H	+0.00000	+0.00000	+0.00000
O	+0.00000	+0.00000	+0.95406
C	−0.90072	+0.00000	+1.26858
C	−1.64063	−1.28565	+0.96132
H	−0.83546	+0.13396	+2.34494
H	−1.44287	+0.86394	+0.87420
C	−1.73887	−1.61603	−0.52304
H	−1.12164	−2.10076	+1.46507
H	−2.63133	−1.22012	+1.40987
C	−2.52146	−0.63056	−1.39037
H	−0.72371	−1.71711	−0.91070
H	−2.17847	−2.60880	−0.63248
C	−3.99329	−0.97329	−1.57818
H	−2.42685	+0.38221	−0.99581
H	−2.06918	−0.59694	−2.38118
C	−4.83798	−1.00437	−0.31512
H	−4.43119	−0.25383	−2.27263
H	−4.05847	−1.94596	−2.06881
C	−4.90475	+0.32701	+0.40707
H	−5.84795	−1.31303	−0.58496
H	−4.46542	−1.77676	+0.35879
H	−5.55698	+0.27455	+1.27574
H	−3.92514	+0.65087	+0.75437
H	−5.29002	+1.10575	−0.24970

24

∗ E = +3.866 kcal/mol ; (371) 180_058_270_173_261_057

H	+0.00000	+0.00000	+0.00000
O	+0.00000	+0.00000	+0.95362
C	−0.89851	+0.00000	+1.27312
C	−0.88862	−0.00220	+2.78118
H	−1.42411	+0.89155	+0.91865
H	−1.43752	−0.87596	+0.90439
C	−0.14602	+1.18100	+3.38948

H −1.92940 −0.00403 +3.10511
H −0.45073 −0.93685 +3.13434
C +1.33324 +0.92006 +3.63327
H −0.26792 +2.05083 +2.74196
H −0.60542 +1.45106 +4.34132
C +2.10529 +2.15996 +4.08038
H +1.41312 +0.13866 +4.39017
H +1.77985 +0.51161 +2.72715
C +2.86479 +2.85149 +2.95809
H +1.41445 +2.87074 +4.54133
H +2.81950 +1.89207 +4.85847
C +1.99645 +3.28317 +1.79261
H +3.38890 +3.71755 +3.36371
H +3.63679 +2.17253 +2.59189
H +2.58541 +3.79465 +1.03394
H +1.51943 +2.42507 +1.32155
H +1.21285 +3.96686 +2.11864
24
* E = +3.867 kcal/mol ; (372) 062_173_064_171_061_265
H +0.00000 +0.00000 +0.00000
O +0.00000 +0.00000 +0.95442
C −0.90225 +0.00000 +1.26564
C −1.66754 −1.22970 +0.82795
H −0.83745 +0.02842 +2.35153
H −1.41432 +0.91104 +0.94845
C −3.05058 −1.34613 +1.44752
H −1.75888 −1.22794 −0.26166
H −1.07023 −2.10228 +1.08979
C −4.00257 −0.23065 +1.05122
H −3.48565 −2.30329 +1.15599
H −2.95053 −1.37656 +2.53410
C −5.44668 −0.44350 +1.47798
H −3.65543 +0.71557 +1.47010
H −3.97039 −0.11116 −0.03399
C −5.65890 −0.56406 +2.98662
H −6.03232 +0.39200 +1.09602
H −5.84153 −1.33588 +0.98675
C −5.69372 −1.99264 +3.49914
H −4.87828 −0.00386 +3.50555
H −6.59767 −0.08260 +3.25790
H −5.83875 −2.02214 +4.57698
H −6.51428 −2.54307 +3.04174
H −4.77645 −2.53090 +3.27448
24
* E = +3.871 kcal/mol ; (373) 179_178_061_061_268_295

```
H    +0.00000   +0.00000   +0.00000
O    +0.00000   +0.00000   +0.95353
C    −0.89810   +0.00000   +1.27390
C    −0.85407   −0.02967   +2.77965
H    −1.43019   +0.89654   +0.94146
H    −1.44207   −0.86683   +0.88960
C    −2.22690   +0.01530   +3.42779
H    −0.30549   −0.91510   +3.09895
H    −0.26762   +0.82598   +3.11240
C    −3.16619   −1.14034   +3.10478
H    −2.09312   +0.05616   +4.50946
H    −2.71812   +0.95046   +3.15201
C    −2.66375   −2.52013   +3.52510
H    −4.10737   −0.94046   +3.61611
H    −3.41357   −1.13867   +2.04261
C    −1.87085   −3.30059   +2.48354
H    −2.05990   −2.41256   +4.42852
H    −3.51846   −3.13499   +3.81159
C    −2.68400   −3.68764   +1.26310
H    −0.98969   −2.74267   +2.17072
H    −1.49397   −4.20847   +2.95496
H    −2.09754   −4.29453   +0.57708
H    −3.56399   −4.26365   +1.54620
H    −3.02957   −2.81626   +0.70938
24
 * E =   +3.875  kcal/mol ;  (374)  183_284_060_266_168_061
H    +0.00000   +0.00000   +0.00000
O    +0.00000   +0.00000   +0.95364
C    −0.89843   +0.00000   +1.27342
C    −0.88597   +0.07891   +2.78085
H    −1.43785   +0.86115   +0.87263
H    −1.42567   −0.90298   +0.94938
C    −0.52095   −1.19427   +3.53045
H    −0.19942   +0.87958   +3.05915
H    −1.87893   +0.39512   +3.10216
C    +0.86094   −1.76939   +3.22946
H    −0.59936   −0.97142   +4.59424
H    −1.27787   −1.95731   +3.33202
C    +0.84973   −2.82739   +2.13862
H    +1.53418   −0.96234   +2.93797
H    +1.27212   −2.20565   +4.13937
C    +2.23103   −3.19928   +1.62977
H    +0.34588   −3.72118   +2.51611
H    +0.25539   −2.46918   +1.30019
C    +3.14135   −3.78468   +2.69255
```

H +2.12879 −3.91475 +0.81323
H +2.69298 −2.30820 +1.20169
H +4.09448 −4.09457 +2.26972
H +3.35327 −3.06519 +3.48042
H +2.68645 −4.65853 +3.15803
24
∗ E = +3.892 kcal/mol ; (375) 183_284_059_265_176_288
H +0.00000 +0.00000 +0.00000
O +0.00000 +0.00000 +0.95367
C −0.89794 +0.00000 +1.27489
C −0.87730 +0.06612 +2.78272
H −1.43626 +0.86548 +0.88206
H −1.42803 −0.89955 +0.94591
C −0.52261 −1.21963 +3.51496
H −0.17914 +0.85579 +3.06352
H −1.86403 +0.39094 +3.11420
C +0.84386 −1.81885 +3.18990
H −0.57973 −1.00546 +4.58191
H −1.29682 −1.96660 +3.32237
C +0.79788 −2.88962 +2.11281
H +1.52277 −1.02078 +2.89376
H +1.26433 −2.26888 +4.09064
C +2.16645 −3.41688 +1.71364
H +0.18483 −3.71601 +2.47777
H +0.29631 −2.49895 +1.22681
C +2.98586 −2.42831 +0.90587
H +2.71597 −3.70201 +2.61308
H +2.04284 −4.33171 +1.13354
H +3.96436 −2.83458 +0.65853
H +2.48088 −2.18326 −0.02707
H +3.13790 −1.49476 +1.44200
24
∗ E = +3.906 kcal/mol ; (376) 177_173_057_057_065_270
H +0.00000 +0.00000 +0.00000
O +0.00000 +0.00000 +0.95351
C −0.89805 +0.00000 +1.27395
C −0.85282 −0.08047 +2.77838
H −1.41964 +0.91252 +0.96828
H −1.44985 −0.85005 +0.86469
C −2.21397 +0.08563 +3.43354
H −0.39916 −1.03062 +3.05787
H −0.17878 +0.69832 +3.13277
C −3.27214 −0.91173 +2.98353
H −2.09670 +0.03100 +4.51542
H −2.58372 +1.09204 +3.22937

C −2.93253 −2.38471 +3.16273
H −4.19596 −0.70289 +3.52563
H −3.50462 −0.73568 +1.93194
C −2.76930 −2.85157 +4.60735
H −3.73672 −2.95739 +2.70120
H −2.03180 −2.63474 +2.59686
C −1.36248 −2.75109 +5.16934
H −3.46115 −2.29522 +5.24320
H −3.08128 −3.89362 +4.67251
H −1.31328 −3.17705 +6.16931
H −0.65794 −3.29879 +4.54496
H −1.01100 −1.72542 +5.23565
24
* E = +3.908 kcal/mol ; (377) 302_181_061_061_268_295
H +0.00000 +0.00000 +0.00000
O +0.00000 +0.00000 +0.95447
C −0.90202 +0.00000 +1.26653
C −1.69423 +1.18817 +0.76960
H −1.41090 −0.92800 +0.98754
H −0.83203 +0.00994 +2.35151
C −3.12692 +1.21663 +1.27536
H −1.17052 +2.10337 +1.04408
H −1.71017 +1.16362 −0.32261
C −3.30300 +1.30824 +2.78604
H −3.63778 +2.06439 +0.81717
H −3.64556 +0.32501 +0.91787
C −2.71012 +2.55829 +3.43314
H −4.37414 +1.28596 +2.98414
H −2.90610 +0.41308 +3.26562
C −1.27585 +2.44832 +3.93705
H −2.77989 +3.38660 +2.72478
H −3.33221 +2.84299 +4.28309
C −1.10373 +1.46137 +5.07581
H −0.59786 +2.18631 +3.12637
H −0.96113 +3.43478 +4.27818
H −0.08072 +1.46342 +5.44433
H −1.75866 +1.70897 +5.91015
H −1.33344 +0.44165 +4.77192
24
* E = +3.912 kcal/mol ; (378) 059_183_302_303_295_090
H +0.00000 +0.00000 +0.00000
O +0.00000 +0.00000 +0.95445
C −0.90201 +0.00000 +1.26648
C −1.69044 −1.19450 +0.77722
H −0.83057 −0.00753 +2.35118

H	−1.41210	+0.92656	+0.98357
C	−3.14888	−1.17391	+1.20710
H	−1.64593	−1.21977	−0.31405
H	−1.19437	−2.10019	+1.12429
C	−3.37184	−1.10771	+2.71153
H	−3.63954	−0.31255	+0.75027
H	−3.65424	−2.04910	+0.79989
C	−2.73134	−2.21206	+3.54054
H	−3.01604	−0.14576	+3.08320
H	−4.44659	−1.11422	+2.90019
C	−3.26480	−3.62018	+3.28820
H	−1.64714	−2.20422	+3.40560
H	−2.89644	−1.96136	+4.58808
C	−2.55053	−4.40370	+2.20145
H	−3.18983	−4.19006	+4.21391
H	−4.33149	−3.56334	+3.06139
H	−2.93334	−5.42038	+2.14137
H	−2.66549	−3.95572	+1.21846
H	−1.48345	−4.46730	+2.41009

24

* E = +3.918 kcal/mol ; (379) 066_301_302_295_092_065

H	+0.00000	+0.00000	+0.00000
O	+0.00000	+0.00000	+0.95446
C	−0.90190	+0.00000	+1.26683
C	−1.64400	−1.27529	+0.92569
H	−0.83956	+0.11562	+2.34550
H	−1.43762	+0.87192	+0.88177
C	−1.71319	−1.57022	−0.56455
H	−1.14553	−2.10620	+1.42403
H	−2.64875	−1.21870	+1.34579
C	−2.35829	−0.50232	−1.43867
H	−0.70290	−1.76352	−0.93105
H	−2.25280	−2.50709	−0.70830
C	−3.84210	−0.25354	−1.17436
H	−1.80470	+0.43606	−1.36277
H	−2.23836	−0.81967	−2.47393
C	−4.18376	+0.83264	−0.16227
H	−4.32627	+0.01507	−2.11445
H	−4.30226	−1.19311	−0.86191
C	−3.76737	+2.22478	−0.59653
H	−5.26260	+0.82209	−0.00607
H	−3.74281	+0.60747	+0.80750
H	−4.08546	+2.97300	+0.12565
H	−2.68755	+2.31569	−0.69979
H	−4.21019	+2.48196	−1.55769

```
24
 * E =   +3.918  kcal/mol  ;  (380) 181_054_051_061_096_301
 H    +0.00000    +0.00000    +0.00000
 O    +0.00000    +0.00000    +0.95339
 C    −0.89770    +0.00000    +1.27446
 C    −0.86068    +0.02998    +2.78173
 H    −1.43994    +0.87201    +0.89774
 H    −1.43107    −0.89132    +0.93637
 C    −0.03415    +1.17480    +3.35132
 H    −1.88929    +0.07385    +3.14039
 H    −0.44917    −0.91266    +3.14109
 C    −0.37299    +2.55102    +2.79339
 H    −0.14039    +1.16661    +4.43545
 H    +1.01414    +0.97371    +3.13804
 C    −1.80368    +3.03458    +3.02972
 H    +0.32161    +3.27884    +3.21782
 H    −0.15704    +2.53786    +1.72534
 C    −1.97328    +3.94026    +4.24039
 H    −2.15150    +3.58018    +2.15258
 H    −2.47511    +2.17950    +3.12951
 C    −1.56444    +3.31213    +5.55809
 H    −1.39099    +4.84988    +4.08244
 H    −3.01543    +4.25521    +4.29995
 H    −1.78045    +3.97527    +6.39287
 H    −2.09581    +2.37684    +5.73059
 H    −0.49847    +3.09590    +5.58230
24
 * E =   +3.919  kcal/mol  ;  (381) 059_181_293_179_059_262
 H    +0.00000    +0.00000    +0.00000
 O    +0.00000    +0.00000    +0.95451
 C    −0.90205    +0.00000    +1.26658
 C    −1.68949    −1.19437    +0.77333
 H    −0.83002    −0.00360    +2.35026
 H    −1.41273    +0.92461    +0.97935
 C    −3.13968    −1.20828    +1.22934
 H    −1.66069    −1.19723    −0.31871
 H    −1.18409    −2.10434    +1.09851
 C    −3.31406    −1.40191    +2.72519
 H    −3.62858    −0.27985    +0.92276
 H    −3.65631    −2.01071    +0.70360
 C    −4.75842    −1.39611    +3.20329
 H    −2.84996    −2.34906    +3.01051
 H    −2.77125    −0.62559    +3.26454
 C    −5.64959    −2.46424    +2.56957
 H    −4.75012    −1.52263    +4.28520
```

```
H   -5.19455   -0.41119   +3.02167
C   -6.48305   -1.96073   +1.40490
H   -5.03165   -3.30460   +2.24642
H   -6.32454   -2.86797   +3.32314
H   -7.08006   -2.75880   +0.96856
H   -7.16693   -1.17998   +1.73381
H   -5.86602   -1.53846   +0.61524
24
* E =  +3.952  kcal/mol ; (382) 177_174_066_180_301_098
H   +0.00000   +0.00000   +0.00000
O   +0.00000   +0.00000   +0.95350
C   -0.89792   +0.00000   +1.27427
C   -0.84814   -0.06656   +2.77975
H   -1.42014   +0.91088   +0.96546
H   -1.44778   -0.85192   +0.86913
C   -2.20622   +0.05950   +3.44907
H   -0.37100   -1.00271   +3.07118
H   -0.19352   +0.73447   +3.12059
C   -3.15270   -1.09246   +3.16154
H   -2.04758   +0.12944   +4.52477
H   -2.67660   +0.99990   +3.15014
C   -4.51792   -0.97527   +3.82295
H   -3.30071   -1.18996   +2.08569
H   -2.67969   -2.02228   +3.48699
C   -4.48608   -0.87305   +5.34788
H   -5.04209   -0.10728   +3.41651
H   -5.10624   -1.84325   +3.52743
C   -4.58930   +0.54771   +5.87263
H   -5.30655   -1.45321   +5.76832
H   -3.57242   -1.33984   +5.72169
H   -4.53399   +0.57569   +6.95885
H   -3.79733   +1.18334   +5.48369
H   -5.53750   +0.99543   +5.57936
24
* E =  +3.961  kcal/mol ; (383) 059_180_292_268_063_061
H   +0.00000   +0.00000   +0.00000
O   +0.00000   +0.00000   +0.95446
C   -0.90208   +0.00000   +1.26630
C   -1.68751   -1.19908   +0.78158
H   -0.83098   +0.00320   +2.35107
H   -1.41439   +0.92253   +0.97599
C   -3.12657   -1.22015   +1.27921
H   -1.69711   -1.18284   -0.31096
H   -1.15117   -2.10073   +1.06973
C   -3.31869   -1.44293   +2.77915
```

H −3.58967 −0.27317 +0.99663
H −3.67853 −1.98966 +0.73711
C −3.49633 −2.89661 +3.19524
H −2.49555 −0.99668 +3.33765
H −4.20906 −0.90307 +3.09968
C −2.32048 −3.82114 +2.92431
H −3.71982 −2.92425 +4.26343
H −4.37984 −3.29298 +2.69136
C −1.04793 −3.41818 +3.64369
H −2.59955 −4.82862 +3.23336
H −2.13867 −3.88378 +1.85082
H −0.25131 −4.13812 +3.47155
H −0.68186 −2.44696 +3.31543
H −1.21315 −3.35792 +4.71864
24
* E = +3.977 kcal/mol ; (384) 064_074_301_092_182_067
H +0.00000 +0.00000 +0.00000
O +0.00000 +0.00000 +0.95428
C −0.90232 +0.00000 +1.26488
C −1.67768 −1.24691 +0.89244
H −0.84120 +0.09724 +2.34743
H −1.42562 +0.88393 +0.89263
C −1.34794 −2.51439 +1.66713
H −2.73859 −1.02885 +1.02512
H −1.54343 −1.42825 −0.17779
C +0.09763 −2.99783 +1.58651
H −1.61696 −2.36804 +2.71599
H −2.01121 −3.29706 +1.29943
C +0.98559 −2.46762 +2.69899
H +0.11413 −4.08820 +1.63125
H +0.51346 −2.72564 +0.61659
C +2.43660 −2.90641 +2.59014
H +0.94957 −1.37900 +2.71052
H +0.57900 −2.80883 +3.65314
C +3.15997 −2.32310 +1.39054
H +2.96117 −2.60919 +3.49834
H +2.48526 −3.99686 +2.55570
H +4.21484 −2.58933 +1.39911
H +2.74220 −2.68306 +0.45277
H +3.08291 −1.23752 +1.38612
24
* E = +3.984 kcal/mol ; (385) 182_183_292_269_063_061
H +0.00000 +0.00000 +0.00000
O +0.00000 +0.00000 +0.95350
C −0.89820 +0.00000 +1.27350

C	−0.85252	+0.05387	+2.77954
H	−1.44562	+0.85810	+0.87517
H	−1.42454	−0.90460	+0.95436
C	−2.22801	−0.00374	+3.42883
H	−0.25743	−0.79330	+3.11897
H	−0.30752	+0.94828	+3.07332
C	−3.13845	+1.20442	+3.20853
H	−2.73140	−0.90050	+3.06355
H	−2.10348	−0.15566	+4.50220
C	−3.03632	+2.28997	+4.27129
H	−2.96493	+1.63608	+2.22233
H	−4.17298	+0.86261	+3.19424
C	−1.68641	+2.97640	+4.40310
H	−3.79246	+3.04930	+4.06231
H	−3.30476	+1.85347	+5.23509
C	−1.23971	+3.69221	+3.14324
H	−1.74746	+3.69728	+5.21859
H	−0.93080	+2.25168	+4.70747
H	−0.30181	+4.21990	+3.29957
H	−1.08840	+3.00245	+2.31472
H	−1.98328	+4.42341	+2.82836

24

* E = +4.026 kcal/mol ; (386) 298_186_292_179_059_262

H	+0.00000	+0.00000	+0.00000
O	+0.00000	+0.00000	+0.95443
C	−0.90234	+0.00000	+1.26542
C	−1.66544	+1.23248	+0.83088
H	−1.41446	−0.91053	+0.94734
H	−0.83786	−0.02986	+2.35110
C	−3.06331	+1.33641	+1.41995
H	−1.07685	+2.10240	+1.11962
H	−1.73149	+1.24954	−0.26051
C	−4.03537	+0.28982	+0.90427
H	−3.00316	+1.27361	+2.50942
H	−3.45624	+2.32762	+1.19558
C	−5.43014	+0.36373	+1.50762
H	−4.11411	+0.39263	−0.18101
H	−3.63592	−0.70819	+1.08495
C	−6.14987	+1.69644	+1.30154
H	−6.02220	−0.44170	+1.07465
H	−5.37190	+0.14921	+2.57708
C	−6.05035	+2.64110	+2.48574
H	−5.75858	+2.18340	+0.40580
H	−7.20419	+1.51197	+1.09943
H	−6.55377	+3.58452	+2.28522

H −6.51528 +2.20037 +3.36609
H −5.01768 +2.86444 +2.74296
24
∗ E = +4.043 kcal/mol ; (387) 060_171_057_057_065_270
H +0.00000 +0.00000 +0.00000
O +0.00000 +0.00000 +0.95453
C −0.90249 +0.00000 +1.26541
C −1.68200 −1.20373 +0.78461
H −0.83887 −0.00441 +2.35179
H −1.40490 +0.92615 +0.97475
C −3.05072 −1.34396 +1.43217
H −1.78465 −1.14817 −0.30156
H −1.08833 −2.09292 +0.99367
C −3.97258 −0.14461 +1.26256
H −3.53824 −2.23878 +1.04600
H −2.91429 −1.52187 +2.50008
C −4.26664 +0.28726 −0.16692
H −4.92005 −0.36735 +1.75593
H −3.55501 +0.70653 +1.80195
C −5.03823 −0.72077 −1.01504
H −4.84571 +1.20856 −0.10981
H −3.33864 +0.55704 −0.67758
C −4.18542 −1.70835 −1.79106
H −5.73976 −1.26168 −0.37672
H −5.65124 −0.17428 −1.73117
H −4.80336 −2.33661 −2.42905
H −3.47824 −1.18526 −2.43367
H −3.61480 −2.36726 −1.14267
24
∗ E = +4.066 kcal/mol ; (388) 061_175_068_091_297_299
H +0.00000 +0.00000 +0.00000
O +0.00000 +0.00000 +0.95448
C −0.90213 +0.00000 +1.26624
C −1.66955 −1.22683 +0.82380
H −0.83755 +0.02549 +2.35199
H −1.41333 +0.91429 +0.95390
C −3.07350 −1.31132 +1.40810
H −1.70222 −1.25138 −0.26612
H −1.09724 −2.10086 +1.13318
C −4.07766 −0.26145 +0.93224
H −3.48161 −2.30285 +1.20515
H −2.98149 −1.24970 +2.49356
C −4.90526 −0.66383 −0.28084
H −4.77464 −0.05550 +1.74387
H −3.57451 +0.68637 +0.73991

C −4.13500 −0.91530 −1.56702
H −5.47046 −1.56285 −0.02871
H −5.64584 +0.11631 −0.46713
C −3.36779 +0.29409 −2.06494
H −3.45729 −1.75902 −1.43314
H −4.84241 −1.22684 −2.33557
H −2.87095 +0.08739 −3.01015
H −4.03530 +1.14051 −2.22032
H −2.60477 +0.61177 −1.35578
24
* E = +4.082 kcal/mol ; (389) 180_071_286_268_063_061
H +0.00000 +0.00000 +0.00000
O +0.00000 +0.00000 +0.95353
C −0.89747 +0.00000 +1.27566
C −0.89044 +0.00395 +2.78486
H −1.43633 +0.87845 +0.90835
H −1.42465 −0.88835 +0.91977
C −0.44169 +1.31080 +3.42860
H −1.91187 −0.20622 +3.10499
H −0.28401 −0.83448 +3.12251
C +1.05129 +1.63627 +3.38315
H −0.99938 +2.11728 +2.94862
H −0.76589 +1.31237 +4.47168
C +1.84376 +1.15075 +4.58936
H +1.48201 +1.25260 +2.46153
H +1.16433 +2.71967 +3.33934
C +1.89382 −0.35471 +4.79422
H +2.86710 +1.52160 +4.50225
H +1.42964 +1.61479 +5.48713
C +2.54679 −1.10072 +3.64647
H +2.44223 −0.55990 +5.71416
H +0.88590 −0.73670 +4.96351
H +2.59566 −2.16916 +3.84668
H +2.00831 −0.96050 +2.71130
H +3.56596 −0.74831 +3.49096
24
* E = +4.090 kcal/mol ; (390) 182_062_273_265_060_177
H +0.00000 +0.00000 +0.00000
O +0.00000 +0.00000 +0.95345
C −0.89914 +0.00000 +1.27064
C −0.89461 +0.05997 +2.77747
H −1.44142 +0.86470 +0.87643
H −1.42161 −0.90136 +0.94058
C −0.25532 +1.32542 +3.34191
H −1.93375 −0.00990 +3.10070

H −0.38847 −0.82621 +3.15834
C +1.25796 +1.25315 +3.54557
H −0.49659 +2.15240 +2.67270
H −0.72514 +1.57156 +4.29621
C +1.67522 +0.86128 +4.95440
H +1.68209 +0.55863 +2.82268
H +1.69727 +2.22503 +3.32300
C +1.18421 −0.49730 +5.41817
H +2.76500 +0.87957 +5.01776
H +1.32030 +1.62036 +5.65670
C +1.69796 −0.86098 +6.79694
H +0.09336 −0.51001 +5.42558
H +1.49367 −1.25719 +4.69807
H +1.33317 −1.83375 +7.11897
H +2.78621 −0.89327 +6.81250
H +1.38233 −0.12724 +7.53717
24
* E = +4.090 kcal/mol ; (391) 061_175_062_061_268_295
H +0.00000 +0.00000 +0.00000
O +0.00000 +0.00000 +0.95452
C −0.90243 +0.00000 +1.26553
C −1.67667 −1.21681 +0.81110
H −0.83866 +0.01238 +2.35167
H −1.41079 +0.91978 +0.96444
C −3.07536 −1.30553 +1.39918
H −1.73007 −1.23095 −0.28002
H −1.10758 −2.10032 +1.09845
C −4.03163 −0.17043 +1.05359
H −3.52610 −2.24447 +1.07523
H −2.99296 −1.37238 +2.48547
C −4.33518 −0.00969 −0.43472
H −4.96442 −0.36739 +1.58088
H −3.66447 +0.76976 +1.46562
C −3.44667 +0.95749 −1.20757
H −4.29489 −0.99226 −0.90958
H −5.36449 +0.33276 −0.55102
C −3.58209 +2.40083 −0.76200
H −2.40214 +0.65528 −1.14109
H −3.70777 +0.88947 −2.26383
H −2.98044 +3.06169 −1.38166
H −4.61675 +2.73371 −0.82992
H −3.26004 +2.54210 +0.26795
24
* E = +4.106 kcal/mol ; (392) 185_284_061_264_286_178
H +0.00000 +0.00000 +0.00000

O	+0.00000	+0.00000	+0.95355
C	−0.89796	+0.00000	+1.27436
C	−0.88526	+0.11437	+2.77964
H	−1.44651	+0.84576	+0.85363
H	−1.41673	−0.91602	+0.97326
C	−0.50983	−1.14017	+3.55408
H	−0.20746	+0.92832	+3.04048
H	−1.88191	+0.42674	+3.09334
C	+0.89342	−1.68627	+3.29045
H	−0.61784	−0.90745	+4.61305
H	−1.25060	−1.91627	+3.34882
C	+0.94246	−2.80383	+2.25937
H	+1.53668	−0.86976	+2.96815
H	+1.31540	−2.06574	+4.22255
C	+0.42194	−4.13233	+2.77685
H	+0.37624	−2.50722	+1.37604
H	+1.97092	−2.93904	+1.92114
C	+0.43041	−5.21870	+1.72025
H	+1.02915	−4.44475	+3.62834
H	−0.59200	−4.01003	+3.16072
H	+0.07042	−6.16675	+2.11372
H	−0.20280	−4.94833	+0.87654
H	+1.43578	−5.38078	+1.33453

24

* E = +4.129 kcal/mol ; (393) 296_286_060_267_171_061

H	+0.00000	+0.00000	+0.00000
O	+0.00000	+0.00000	+0.95424
C	−0.90254	+0.00000	+1.26408
C	−1.67758	+1.24961	+0.89996
H	−1.42770	−0.88044	+0.88595
H	−0.84245	−0.10442	+2.34600
C	−1.33797	+2.51635	+1.67147
H	−1.55466	+1.43196	−0.17146
H	−2.73738	+1.03298	+1.04392
C	+0.10352	+3.00750	+1.56100
H	−2.01471	+3.29582	+1.32222
H	−1.58180	+2.36461	+2.72572
C	+1.00950	+2.49252	+2.66642
H	+0.51739	+2.71717	+0.59348
H	+0.10738	+4.09698	+1.58022
C	+2.48041	+2.80448	+2.45397
H	+0.68095	+2.92097	+3.61705
H	+0.89498	+1.41454	+2.75154
C	+2.79811	+4.28677	+2.39912
H	+3.05673	+2.34502	+3.25701

H +2.80981 +2.32294 +1.53200
H +3.86934 +4.45808 +2.31886
H +2.32740 +4.77001 +1.54559
H +2.44922 +4.79415 +3.29805
24
* E = +4.137 kcal/mol ; (394) 063_059_071_072_100_300
H +0.00000 +0.00000 +0.00000
O +0.00000 +0.00000 +0.95459
C −0.90204 +0.00000 +1.26693
C −1.66648 −1.24299 +0.86194
H −0.83191 +0.07507 +2.34911
H −1.43069 +0.89059 +0.91884
C −1.04533 −2.53877 +1.36137
H −2.69347 −1.14651 +1.21871
H −1.73176 −1.26912 −0.22830
C −1.17987 −2.75282 +2.86347
H −1.50496 −3.37530 +0.83586
H +0.00922 −2.53812 +1.08795
C −2.59592 −3.09416 +3.33099
H −0.50353 −3.55281 +3.16948
H −0.82050 −1.86324 +3.37964
C −2.82529 −4.57700 +3.58160
H −2.82411 −2.55539 +4.24988
H −3.32217 −2.74414 +2.59384
C −2.61424 −5.45510 +2.36403
H −2.15860 −4.90539 +4.38100
H −3.83939 −4.71913 +3.95573
H −2.83486 −6.49649 +2.58735
H −3.25966 −5.14818 +1.54175
H −1.58567 −5.40869 +2.01117
24
* E = +4.174 kcal/mol ; (395) 066_058_272_174_261_057
H +0.00000 +0.00000 +0.00000
O +0.00000 +0.00000 +0.95460
C −0.90198 +0.00000 +1.26717
C −1.66684 −1.26440 +0.93823
H −0.82876 +0.10940 +2.34719
H −1.43929 +0.87330 +0.88895
C −1.02886 −2.53535 +1.48273
H −2.67052 −1.14350 +1.34801
H −1.79445 −1.34807 −0.14464
C +0.00197 −3.16758 +0.55931
H −0.57424 −2.31842 +2.45018
H −1.80501 −3.27677 +1.67686
C +0.73360 −4.35370 +1.18508

H −0.51074 −3.48273 −0.35077
H +0.73067 −2.41518 +0.25789
C +2.10033 −4.00430 +1.75538
H +0.11172 −4.78570 +1.97312
H +0.86538 −5.14119 +0.44372
C +2.08045 −2.90979 +2.80454
H +2.54863 −4.90494 +2.17593
H +2.75100 −3.69661 +0.93480
H +3.07755 −2.73207 +3.20156
H +1.72034 −1.96933 +2.39042
H +1.43586 −3.17909 +3.64092
24
* E = +4.184 kcal/mol ; (396) 301_069_227_071_072_066
H +0.00000 +0.00000 +0.00000
O +0.00000 +0.00000 +0.95526
C −0.90180 +0.00000 +1.27035
C −1.68581 +1.19434 +0.77528
H −1.40768 −0.92868 +0.98917
H −0.82972 +0.01577 +2.35439
C −1.96196 +1.16616 −0.73044
H −2.62538 +1.25180 +1.32402
H −1.12064 +2.08870 +1.03837
C −1.66711 +2.48693 −1.42567
H −1.37665 +0.37534 −1.20751
H −2.99969 +0.88704 −0.90499
C −0.18058 +2.79759 −1.53751
H −2.11841 +2.49115 −2.41748
H −2.15865 +3.28750 −0.87111
C +0.58236 +1.94050 −2.53775
H −0.05514 +3.84404 −1.81939
H +0.28333 +2.69239 −0.55530
C +0.16397 +2.15604 −3.97955
H +1.64506 +2.15866 −2.43774
H +0.47602 +0.88175 −2.29184
H +0.78258 +1.57533 −4.65974
H −0.86955 +1.86394 −4.15163
H +0.26000 +3.20450 −4.25789
24
* E = +4.191 kcal/mol ; (397) 176_061_060_267_263_060
H +0.00000 +0.00000 +0.00000
O +0.00000 +0.00000 +0.95350
C −0.89772 +0.00000 +1.27486
C −0.86384 −0.09626 +2.77890
H −1.41647 +0.91330 +0.96803
H −1.45172 −0.84855 +0.86752

C −0.14259 +1.05193 +3.46818
H −1.89488 −0.14276 +3.13416
H −0.39121 −1.04001 +3.04989
C −0.75791 +2.42261 +3.18983
H −0.15013 +0.85301 +4.53954
H +0.90164 +1.04470 +3.15958
C −0.14131 +3.17019 +2.00553
H −1.83057 +2.29499 +3.03085
H −0.67080 +3.05073 +4.07816
C +0.92440 +4.17817 +2.40876
H +0.29758 +2.44914 +1.31483
H −0.91968 +3.69935 +1.45524
C +2.10747 +3.57123 +3.13724
H +1.27868 +4.69414 +1.51590
H +0.46673 +4.94285 +3.03961
H +2.85505 +4.32616 +3.37078
H +1.80400 +3.10992 +4.07546
H +2.58635 +2.80183 +2.53333
24
* E = +4.233 kcal/mol ; (398) 179_078_298_301_297_092
H +0.00000 +0.00000 +0.00000
O +0.00000 +0.00000 +0.95359
C −0.89839 +0.00000 +1.27331
C −0.88332 −0.02920 +2.78294
H −1.43249 +0.88889 +0.92312
H −1.42949 −0.87739 +0.89740
C −0.57153 +1.29998 +3.45405
H −1.86516 −0.36297 +3.11976
H −0.17391 −0.79747 +3.09064
C +0.79221 +1.89935 +3.14733
H −1.33878 +2.02014 +3.16208
H −0.68017 +1.17978 +4.53276
C +1.99707 +1.04518 +3.51190
H +0.84786 +2.12960 +2.08473
H +0.86864 +2.85179 +3.67633
C +2.15970 +0.73570 +4.99858
H +1.97279 +0.11918 +2.93620
H +2.88210 +1.58028 +3.16787
C +1.50275 −0.55086 +5.46707
H +3.22295 +0.66878 +5.22863
H +1.78280 +1.57580 +5.58637
H +1.72485 −0.74428 +6.51479
H +0.42123 −0.52797 +5.36386
H +1.86845 −1.40009 +4.89143
24

```
 * E =  +4.240  kcal/mol ; (399) 298_302_300_093_096_300
 H   +0.00000   +0.00000   +0.00000
 O   +0.00000   +0.00000   +0.95455
 C   −0.90166   +0.00000   +1.26789
 C   −1.67845   +1.22494   +0.83841
 H   −1.42096   −0.90468   +0.94323
 H   −0.83273   −0.04352   +2.35283
 C   −1.07072   +2.54590   +1.28438
 H   −1.77952   +1.21783   −0.24971
 H   −2.69145   +1.13728   +1.23675
 C   −0.94778   +2.68235   +2.80143
 H   −0.08857   +2.65712   +0.82617
 H   −1.68899   +3.35042   +0.88703
 C   +0.39829   +2.23744   +3.37806
 H   −1.12225   +3.72031   +3.09007
 H   −1.75674   +2.11443   +3.26527
 C   +1.37581   +3.38248   +3.59593
 H   +0.24217   +1.73827   +4.33445
 H   +0.85442   +1.50231   +2.71436
 C   +1.73538   +4.14112   +2.33344
 H   +0.95155   +4.07687   +4.32402
 H   +2.28571   +2.98721   +4.04792
 H   +2.46278   +4.92364   +2.53806
 H   +2.16366   +3.47471   +1.58598
 H   +0.86125   +4.61542   +1.89017
24
 * E =  +4.243  kcal/mol ; (400) 177_175_060_263_289_295
 H   +0.00000   +0.00000   +0.00000
 O   +0.00000   +0.00000   +0.95352
 C   −0.89811   +0.00000   +1.27383
 C   −0.84868   −0.06916   +2.77818
 H   −1.42253   +0.90879   +0.96151
 H   −1.44750   −0.85554   +0.87303
 C   −2.20484   +0.03947   +3.45644
 H   −0.36710   −1.00568   +3.06133
 H   −0.19750   +0.73238   +3.12513
 C   −3.19614   −1.05925   +3.07160
 H   −2.03706   +0.02251   +4.53194
 H   −2.63729   +1.01871   +3.24229
 C   −4.18218   −0.67065   +1.97672
 H   −2.63869   −1.94236   +2.75385
 H   −3.75788   −1.37396   +3.95009
 C   −5.23517   +0.34445   +2.39813
 H   −3.63730   −0.27402   +1.11934
 H   −4.69150   −1.56878   +1.62246
```

C −6.19709 −0.16768 +3.45301
H −4.75221 +1.25379 +2.75787
H −5.80186 +0.63931 +1.51503
H −6.97210 +0.56508 +3.66560
H −6.68817 −1.08185 +3.12213
H −5.69348 −0.38650 +4.39189
24
* E = +4.258 kcal/mol ; (401) 301_179_061_262_290_295
H +0.00000 +0.00000 +0.00000
O +0.00000 +0.00000 +0.95452
C −0.90229 +0.00000 +1.26595
C −1.68886 +1.19102 +0.76710
H −1.41053 −0.92903 +0.98769
H −0.83204 +0.01600 +2.35052
C −3.14079 +1.21737 +1.21989
H −1.18156 +2.09882 +1.09559
H −1.65741 +1.19587 −0.32518
C −3.32332 +1.26920 +2.73732
H −3.61651 +2.08245 +0.76108
H −3.65967 +0.34663 +0.81454
C −3.59893 −0.07941 +3.39084
H −2.43005 +1.70748 +3.18603
H −4.13523 +1.95060 +2.98751
C −4.96939 −0.66712 +3.08597
H −2.83498 −0.79551 +3.08665
H −3.50146 +0.02523 +4.47285
C −6.12485 +0.14338 +3.64127
H −5.09307 −0.78477 +2.00863
H −5.01119 −1.67425 +3.50032
H −7.07485 −0.35500 +3.46277
H −6.02177 +0.28321 +4.71648
H −6.18713 +1.12939 +3.18622
24
* E = +4.271 kcal/mol ; (402) 294_057_056_066_100_300
H +0.00000 +0.00000 +0.00000
O +0.00000 +0.00000 +0.95463
C −0.90198 +0.00000 +1.26729
C −1.64981 +1.27084 +0.92283
H −1.43886 −0.87387 +0.88847
H −0.83950 −0.11083 +2.34632
C −1.68323 +1.59285 −0.56510
H −2.66330 +1.18224 +1.31622
H −1.18429 +2.10372 +1.44850
C −2.24612 +0.49011 −1.45375
H −2.25857 +2.50662 −0.70709

H −0.67034 +1.83319 −0.89368
C −3.73102 +0.18714 −1.24571
H −2.08287 +0.76386 −2.49775
H −1.66441 −0.42094 −1.30280
C −4.64643 +0.83114 −2.27608
H −3.89418 −0.88951 −1.27254
H −4.03812 +0.50827 −0.24891
C −4.57834 +2.34513 −2.31260
H −4.39896 +0.43605 −3.26295
H −5.67240 +0.52239 −2.07534
H −5.29429 +2.75347 −3.02215
H −4.79966 +2.77235 −1.33525
H −3.59152 +2.69450 −2.61030
24
* E = +4.339 kcal/mol ; (403) 294_060_271_263_061_177
H +0.00000 +0.00000 +0.00000
O +0.00000 +0.00000 +0.95457
C −0.90183 +0.00000 +1.26746
C −1.66108 +1.26895 +0.94188
H −1.44243 −0.86236 +0.86522
H −0.83828 −0.13248 +2.34431
C −1.77069 +1.54263 −0.55625
H −2.65594 +1.17141 +1.37818
H −1.17830 +2.10813 +1.44140
C −0.61160 +2.34941 −1.14922
H −1.86615 +0.58544 −1.07516
H −2.70140 +2.07177 −0.76540
C −0.90343 +3.83630 −1.27946
H +0.27958 +2.21666 −0.53414
H −0.36480 +1.96647 −2.13961
C −1.20487 +4.54188 +0.02928
H −0.05078 +4.32455 −1.75458
H −1.74827 +3.97407 −1.95896
C −1.42791 +6.02986 −0.15193
H −2.08786 +4.09780 +0.49087
H −0.38099 +4.37530 +0.72576
H −1.64715 +6.52067 +0.79337
H −0.54746 +6.50848 −0.57746
H −2.26195 +6.22086 −0.82540
24
* E = +4.341 kcal/mol ; (404) 183_289_071_173_261_060
H +0.00000 +0.00000 +0.00000
O +0.00000 +0.00000 +0.95359
C −0.89903 +0.00000 +1.27152
C −0.90273 +0.07353 +2.77859

H −1.44179 +0.85831 +0.86834
H −1.42082 −0.90591 +0.94793
C −0.42947 −1.17727 +3.50196
H −0.29970 +0.93118 +3.08037
H −1.92690 +0.28955 +3.08472
C +1.05883 −1.47155 +3.39469
H −0.69974 −1.08565 +4.55420
H −0.98880 −2.03891 +3.12850
C +1.50955 −2.63147 +4.28229
H +1.30187 −1.67044 +2.35503
H +1.62137 −0.57132 +3.65470
C +2.14572 −2.20969 +5.59825
H +0.65586 −3.28202 +4.48933
H +2.22844 −3.24783 +3.74350
C +1.24672 −1.38018 +6.49358
H +2.46629 −3.10117 +6.13816
H +3.05364 −1.64390 +5.38175
H +1.73877 −1.14427 +7.43466
H +0.97640 −0.43695 +6.02227
H +0.32393 −1.91081 +6.72563
24
∗ E = +4.348 kcal/mol ; (405) 061_068_291_188_099_300
H +0.00000 +0.00000 +0.00000
O +0.00000 +0.00000 +0.95448
C −0.90195 +0.00000 +1.26676
C −1.71380 −1.20151 +0.82978
H −0.83220 +0.02509 +2.35249
H −1.40962 +0.91658 +0.95524
C −1.29714 −2.53437 +1.42960
H −2.75232 −1.00212 +1.09872
H −1.69670 −1.26645 −0.26226
C +0.04770 −3.06951 +0.96194
H −1.27985 −2.44278 +2.51819
H −2.07168 −3.26867 +1.20763
C +0.33487 −4.49026 +1.44720
H +0.08188 −3.04768 −0.13155
H +0.82788 −2.39015 +1.29352
C +0.04827 −5.57788 +0.42295
H +1.38023 −4.57196 +1.74232
H −0.24628 −4.68660 +2.35163
C −1.38960 −5.62987 −0.05444
H +0.70500 −5.43080 −0.43645
H +0.32003 −6.54411 +0.84864
H −1.54318 −6.45179 −0.75021
H −2.07668 −5.76873 +0.77945

```
H   −1.67324   −4.71232   −0.56710
24
∗ E = +4.384 kcal/mol ; (406) 182_281_063_087_299_190
H   +0.00000   +0.00000   +0.00000
O   +0.00000   +0.00000   +0.95374
C   −0.89919   +0.00000   +1.27168
C   −0.89480   +0.04062   +2.78179
H   −1.42890   +0.87489   +0.88829
H   −1.43059   −0.89080   +0.92308
C   −0.61815   −1.29029   +3.47117
H   −0.18647   +0.80803   +3.08575
H   −1.87911   +0.38513   +3.10126
C   +0.74504   −1.93616   +3.23513
H   −0.76904   −1.16285   +4.54568
H   −1.39479   −1.98586   +3.14720
C   +1.85119   −1.49481   +4.18098
H   +0.63246   −3.01509   +3.34531
H   +1.05651   −1.76059   +2.20749
C   +2.19009   −0.01546   +4.15310
H   +1.58719   −1.77834   +5.20330
H   +2.75263   −2.06122   +3.93789
C   +3.45295   +0.30031   +4.92959
H   +2.29391   +0.31156   +3.11742
H   +1.36301   +0.55670   +4.57389
H   +3.67310   +1.36553   +4.92403
H   +3.36274   −0.01353   +5.96873
H   +4.31172   −0.21777   +4.50550
24
∗ E = +4.391 kcal/mol ; (407) 061_173_060_262_290_295
H   +0.00000   +0.00000   +0.00000
O   +0.00000   +0.00000   +0.95448
C   −0.90220   +0.00000   +1.26603
C   −1.67056   −1.22019   +0.81091
H   −0.83785   +0.01850   +2.35230
H   −1.41240   +0.91516   +0.95559
C   −3.06329   −1.34943   +1.40886
H   −1.75216   −1.20016   −0.27954
H   −1.07873   −2.09972   +1.06265
C   −4.00420   −0.18864   +1.08328
H   −3.49247   −2.28353   +1.05040
H   −2.97615   −1.46248   +2.49092
C   −4.10617   +0.87257   +2.17199
H   −3.67680   +0.28323   +0.15475
H   −5.00248   −0.57100   +0.87481
C   −4.83483   +0.42548   +3.43125
```

H −3.10662 +1.21090 +2.44640
H −4.62090 +1.74610 +1.76778
C −6.30385 +0.11554 +3.21604
H −4.33862 −0.44645 +3.85912
H −4.74432 +1.21365 +4.17840
H −6.79509 −0.12601 +4.15574
H −6.82286 +0.96878 +2.78135
H −6.44844 −0.73171 +2.54938
24
* E = +4.415 kcal/mol ; (408) 059_074_299_094_073_181
H +0.00000 +0.00000 +0.00000
O +0.00000 +0.00000 +0.95441
C −0.90305 +0.00000 +1.26326
C −1.71983 −1.18272 +0.78478
H −0.84170 +0.00128 +2.35029
H −1.39865 +0.92844 +0.96863
C −1.42531 −2.52809 +1.43105
H −2.77125 −0.94375 +0.95206
H −1.60473 −1.26557 −0.30016
C −0.00853 −3.06810 +1.24237
H −1.65100 −2.46004 +2.49711
H −2.14162 −3.24285 +1.02692
C +0.95452 −2.73307 +2.37133
H −0.04934 −4.15400 +1.14178
H +0.39337 −2.69193 +0.30191
C +0.70419 −3.52800 +3.63984
H +1.97521 −2.92577 +2.03728
H +0.91293 −1.66604 +2.58618
C +1.66111 −3.16178 +4.75663
H −0.32113 −3.37582 +3.98026
H +0.79143 −4.59314 +3.41732
H +1.47665 −3.74889 +5.65356
H +2.69418 −3.33039 +4.45674
H +1.56691 −2.11022 +5.02249
24
* E = +4.438 kcal/mol ; (409) 298_293_068_068_271_188
H +0.00000 +0.00000 +0.00000
O +0.00000 +0.00000 +0.95440
C −0.90163 +0.00000 +1.26737
C −1.71048 +1.21183 +0.85345
H −1.41435 −0.90993 +0.94479
H −0.83082 −0.04029 +2.35269
C −1.26664 +2.53216 +1.46442
H −1.74165 +1.27936 −0.23514
H −2.74068 +1.02339 +1.16119

```
 C    +0.08019    +3.10625    +1.04157
 H    -2.03402    +3.27980    +1.25395
 H    -1.26059    +2.41054    +2.54955
 C    +0.18211    +3.57532    -0.40714
 H    +0.26561    +3.96589    +1.68586
 H    +0.87279    +2.39010    +1.25079
 C    +0.62351    +2.53376    -1.42241
 H    -0.77123    +4.00760    -0.72250
 H    +0.90386    +4.39262    -0.45408
 C    +0.86980    +3.13335    -2.79272
 H    +1.53476    +2.05456    -1.06051
 H    -0.12566    +1.74758    -1.51202
 H    +1.18521    +2.38045    -3.51147
 H    -0.03127    +3.60536    -3.18112
 H    +1.64567    +3.89588    -2.74968
24
 * E =   +4.442  kcal/mol  ;  (410)  182_290_073_172_061_263
 H    +0.00000    +0.00000    +0.00000
 O    +0.00000    +0.00000    +0.95359
 C    -0.89885    +0.00000    +1.27204
 C    -0.90163    +0.05460    +2.77959
 H    -1.43882    +0.86540    +0.88024
 H    -1.42454    -0.89979    +0.93797
 C    -0.40612    -1.19959    +3.48372
 H    -0.31278    +0.91751    +3.09373
 H    -1.92943    +0.24908    +3.08789
 C    +1.09472    -1.42298    +3.41867
 H    -0.70388    -1.14357    +4.53314
 H    -0.92878    -2.06412    +3.06825
 C    +1.59535    -2.56793    +4.28545
 H    +1.39131    -1.59187    +2.38533
 H    +1.59662    -0.50304    +3.72347
 C    +1.01759    -3.93907    +3.93401
 H    +2.68094    -2.59988    +4.19778
 H    +1.38659    -2.35051    +5.33608
 C    -0.16915    -4.34945    +4.78736
 H    +0.73533    -3.94936    +2.87920
 H    +1.79164    -4.69846    +4.04036
 H    -0.55707    -5.32112    +4.48800
 H    +0.11900    -4.41861    +5.83517
 H    -0.98441    -3.63301    +4.72248
24
 * E =   +4.448  kcal/mol  ;  (411)  299_293_071_172_061_264
 H    +0.00000    +0.00000    +0.00000
 O    +0.00000    +0.00000    +0.95448
```

C −0.90205 +0.00000 +1.26648
C −1.70970 +1.20772 +0.83946
H −1.41283 −0.91227 +0.94757
H −0.83263 −0.03402 +2.35198
C −1.27901 +2.53505 +1.44347
H −1.69999 +1.27798 −0.25218
H −2.74717 +1.01294 +1.11570
C +0.04484 +3.07497 +0.93082
H −2.05839 +3.27376 +1.24435
H −1.23670 +2.42526 +2.52898
C +0.36543 +4.48999 +1.38715
H +0.84897 +2.40641 +1.23317
H +0.02974 +3.05514 −0.16160
C +0.47653 +4.66494 +2.90171
H +1.30480 +4.78386 +0.91989
H −0.38974 +5.17925 +1.00104
C −0.79493 +5.16415 +3.56437
H +0.78075 +3.71744 +3.35085
H +1.27644 +5.36923 +3.12751
H −0.67052 +5.25728 +4.64132
H −1.06772 +6.14519 +3.17853
H −1.63679 +4.49995 +3.38501
24
* E = +4.455 kcal/mol ; (412) 059_180_290_289_260_060
H +0.00000 +0.00000 +0.00000
O +0.00000 +0.00000 +0.95447
C −0.90223 +0.00000 +1.26592
C −1.68806 −1.19775 +0.77837
H −0.83142 +0.00189 +2.34996
H −1.41347 +0.92278 +0.97467
C −3.12877 −1.22249 +1.26687
H −1.68548 −1.18933 −0.31393
H −1.16096 −2.10334 +1.07842
C −3.28535 −1.48937 +2.75962
H −3.59993 −0.26931 +1.01914
H −3.67462 −1.98262 +0.70933
C −2.95708 −2.92231 +3.18285
H −2.66427 −0.78713 +3.31418
H −4.31146 −1.26036 +3.05297
C −4.18035 −3.80235 +3.38993
H −2.30815 −3.38709 +2.43782
H −2.38348 −2.91276 +4.10855
C −5.04417 −3.96964 +2.15532
H −3.85399 −4.78337 +3.73541
H −4.78392 −3.38244 +4.19664

H −5.87573 −4.64434 +2.34560
H −5.46467 −3.02003 +1.82915
H −4.46853 −4.37871 +1.32578
24
∗ E = +4.473 kcal/mol ; (413) 180_065_278_177_059_263
H +0.00000 +0.00000 +0.00000
O +0.00000 +0.00000 +0.95356
C −0.89849 +0.00000 +1.27292
C −0.90169 −0.00456 +2.78128
H −1.43086 +0.88551 +0.91303
H −1.43201 −0.88113 +0.90789
C −0.31613 +1.24109 +3.43322
H −1.94057 −0.12251 +3.09074
H −0.37087 −0.89144 +3.13036
C +1.19918 +1.23941 +3.53799
H −0.64484 +2.12516 +2.88008
H −0.73887 +1.33664 +4.43397
C +1.79499 +2.51488 +4.11363
H +1.49945 +0.39657 +4.16584
H +1.62357 +1.05320 +2.55473
C +1.31318 +2.87982 +5.51778
H +2.87848 +2.40003 +4.12012
H +1.58864 +3.34748 +3.43669
C +0.18285 +3.89308 +5.54035
H +1.00518 +1.97145 +6.04008
H +2.14426 +3.28416 +6.09477
H −0.14327 +4.10372 +6.55696
H +0.50418 +4.83382 +5.09596
H −0.68256 +3.54920 +4.97896
24
∗ E = +4.494 kcal/mol ; (414) 181_064_185_093_298_089
H +0.00000 +0.00000 +0.00000
O +0.00000 +0.00000 +0.95324
C −0.89626 +0.00000 +1.27788
C −0.84363 +0.01770 +2.78379
H −1.43573 +0.87975 +0.91466
H −1.42976 −0.88833 +0.93259
C −0.21307 +1.28415 +3.33661
H −1.86011 −0.07948 +3.16803
H −0.29951 −0.86486 +3.11716
C −0.04142 +1.28306 +4.85290
H +0.74725 +1.45313 +2.85029
H −0.84768 +2.12324 +3.04619
C +1.29940 +0.79728 +5.38746
H −0.18721 +2.29729 +5.22451

H −0.83973 +0.68720 +5.30287
C +1.68823 −0.64541 +5.07549
H +2.08736 +1.46054 +5.02286
H +1.27867 +0.92807 +6.46974
C +2.44797 −0.82982 +3.77328
H +2.31477 −1.01865 +5.88570
H +0.79462 −1.27264 +5.08514
H +2.71757 −1.87375 +3.62402
H +1.88405 −0.51047 +2.90096
H +3.37244 −0.25407 +3.79249
24
* E = +4.499 kcal/mol ; (415) 177_176_067_069_100_300
H +0.00000 +0.00000 +0.00000
O +0.00000 +0.00000 +0.95349
C −0.89813 +0.00000 +1.27366
C −0.85094 −0.07598 +2.77869
H −1.41831 +0.91314 +0.96869
H −1.44946 −0.84853 +0.86279
C −2.21640 +0.01805 +3.44088
H −0.34348 −0.99791 +3.06142
H −0.21957 +0.73967 +3.12862
C −3.14265 −1.16382 +3.17651
H −2.07187 +0.12464 +4.51536
H −2.70493 +0.93660 +3.11011
C −2.71352 −2.47007 +3.84722
H −4.14738 −0.90585 +3.51676
H −3.23335 −1.31157 +2.10094
C −3.46966 −2.78563 +5.12892
H −2.84984 −3.30250 +3.15810
H −1.64435 −2.43868 +4.06532
C −3.31564 −1.74063 +6.21641
H −4.52869 −2.90545 +4.89308
H −3.13290 −3.75067 +5.50796
H −3.83287 −2.03961 +7.12529
H −2.26678 −1.58647 +6.46700
H −3.72635 −0.78039 +5.90963
24
* E = +4.560 kcal/mol ; (416) 063_072_297_300_298_093
H +0.00000 +0.00000 +0.00000
O +0.00000 +0.00000 +0.95436
C −0.90228 +0.00000 +1.26531
C −1.69285 −1.22971 +0.86684
H −0.83569 +0.06671 +2.34967
H −1.41936 +0.89811 +0.91855
C −1.31566 −2.50881 +1.59844

H −2.74656 −1.01951 +1.05702
H −1.60978 −1.36545 −0.21427
C +0.09972 −3.01786 +1.37129
H −1.45310 −2.34101 +2.66835
H −2.02903 −3.29010 +1.33225
C +0.46439 −3.36230 −0.06520
H +0.80770 −2.27774 +1.73794
H +0.23782 −3.91310 +1.98078
C −0.35489 −4.48275 −0.70286
H +0.40427 −2.46825 −0.69009
H +1.51611 −3.64683 −0.06931
C −1.57869 −4.02512 −1.47643
H +0.28308 −5.04212 −1.38659
H −0.65402 −5.19297 +0.07086
H −2.07721 −4.86634 −1.95370
H −2.31038 −3.53105 −0.84278
H −1.29745 −3.32392 −2.26135
24
* E = +4.574 kcal/mol ; (417) 062_069_289_180_059_262
H +0.00000 +0.00000 +0.00000
O +0.00000 +0.00000 +0.95447
C −0.90215 +0.00000 +1.26614
C −1.70592 −1.21850 +0.86074
H −0.83355 +0.05417 +2.35082
H −1.41689 +0.90400 +0.93064
C −1.29718 −2.53079 +1.51189
H −2.74767 −1.01076 +1.11020
H −1.67595 −1.32121 −0.22791
C +0.02788 −3.09894 +1.03578
H −1.26585 −2.39498 +2.59615
H −2.08551 −3.26001 +1.32135
C +0.42889 −4.40582 +1.70370
H −0.03184 −3.26040 −0.04437
H +0.81209 −2.36203 +1.19148
C −0.56906 −5.55198 +1.53754
H +1.39538 −4.70183 +1.29735
H +0.59677 −4.22846 +2.76853
C −1.49743 −5.73795 +2.72428
H −1.15630 −5.39053 +0.63089
H −0.02986 −6.48483 +1.37679
H −2.20858 −6.54336 +2.55214
H −0.92858 −5.98629 +3.61885
H −2.06401 −4.83558 +2.94212
24
* E = +4.581 kcal/mol ; (418) 061_176_071_072_100_300

H	+0.00000	+0.00000	+0.00000
O	+0.00000	+0.00000	+0.95442
C	−0.90230	+0.00000	+1.26551
C	−1.67214	−1.22342	+0.81777
H	−0.83745	+0.01835	+2.35125
H	−1.41139	+0.91634	+0.95818
C	−3.08587	−1.29491	+1.37614
H	−1.70171	−1.24816	−0.27452
H	−1.10760	−2.10158	+1.12835
C	−4.05546	−0.27798	+0.78381
H	−3.47713	−2.29797	+1.21070
H	−3.04017	−1.16410	+2.45882
C	−4.46528	−0.56564	−0.66186
H	−4.95274	−0.24181	+1.40417
H	−3.61676	+0.71647	+0.85465
C	−5.83073	−1.22190	−0.79937
H	−4.47305	+0.35952	−1.23675
H	−3.71711	−1.20417	−1.13671
C	−5.94955	−2.56401	−0.10452
H	−6.58835	−0.54353	−0.40295
H	−6.05828	−1.34404	−1.85845
H	−6.92840	−3.00766	−0.27141
H	−5.20085	−3.26462	−0.47241
H	−5.81424	−2.47104	+0.97155

24

∗ E = +4.590 kcal/mol ; (419) 064_276_056_170_262_059

H	+0.00000	+0.00000	+0.00000
O	+0.00000	+0.00000	+0.95329
C	−0.89840	+0.00000	+1.27210
C	−1.68210	−1.24536	+0.90057
H	−0.81112	+0.07116	+2.35265
H	−1.43040	+0.89512	+0.93305
C	−2.31220	−1.23252	−0.48275
H	−1.01190	−2.10002	+1.00371
H	−2.47549	−1.38320	+1.63588
C	−1.34792	−1.00102	−1.63690
H	−2.83530	−2.17561	−0.63651
H	−3.08113	−0.45766	−0.51943
C	−1.96824	−1.24554	−3.01228
H	−0.99249	+0.03083	−1.59252
H	−0.46933	−1.64167	−1.51771
C	−1.67387	−2.61900	−3.59613
H	−3.04924	−1.10320	−2.94521
H	−1.61050	−0.49558	−3.71641
C	−2.14802	−3.78203	−2.74744

```
H    -2.13135   -2.68438   -4.58332
H    -0.59781   -2.70777   -3.75533
H    -1.94949   -4.73123   -3.23967
H    -1.64447   -3.80537   -1.78259
H    -3.21958   -3.72327   -2.56092
24
* E =  +4.623  kcal/mol ; (420) 062_077_299_273_062_171
H    +0.00000   +0.00000   +0.00000
O    +0.00000   +0.00000   +0.95456
C    -0.90311   +0.00000   +1.26370
C    -1.69673   -1.22552   +0.85450
H    -0.83951   +0.06321   +2.34839
H    -1.41544   +0.90124   +0.91856
C    -1.39808   -2.49224   +1.64758
H    -2.75425   -0.98981   +0.98555
H    -1.56099   -1.38247   -0.21599
C    +0.02672   -3.03954   +1.60246
H    -1.65266   -2.27952   +2.68736
H    -2.09062   -3.27562   +1.33093
C    +0.33115   -3.98735   +0.45271
H    +0.73545   -2.21356   +1.59395
H    +0.20514   -3.58694   +2.52809
C    +0.20509   -3.40151   -0.94172
H    +1.34948   -4.36026   +0.57866
H    -0.31892   -4.86310   +0.52518
C    +0.73284   -4.33660   -2.01157
H    -0.83979   -3.17442   -1.15225
H    +0.74397   -2.45248   -0.98179
H    +0.61930   -3.91535   -3.00793
H    +1.78979   -4.54788   -1.85850
H    +0.20326   -5.28789   -1.99244
24
* E =  +4.731  kcal/mol ; (421) 064_276_057_169_061_265
H    +0.00000   +0.00000   +0.00000
O    +0.00000   +0.00000   +0.95331
C    -0.89810   +0.00000   +1.27303
C    -1.68116   -1.24788   +0.90845
H    -0.80969   +0.07606   +2.35317
H    -1.43177   +0.89290   +0.93083
C    -2.31886   -1.23806   -0.47240
H    -1.00919   -2.10119   +1.00933
H    -2.47058   -1.38516   +1.64804
C    -1.35075   -1.02936   -1.62434
H    -2.84258   -2.18397   -0.62109
H    -3.08391   -0.45988   -0.50117
```

C −1.93889 −1.26870 −3.00692
H −0.97519 −0.00151 −1.59956
H −0.48911 −1.68727 −1.49108
C −3.11202 −0.36081 −3.37349
H −1.13921 −1.13765 −3.73502
H −2.25030 −2.31239 −3.08813
C −4.47853 −0.96003 −3.09398
H −3.01033 +0.58996 −2.84596
H −3.05857 −0.11680 −4.43374
H −5.27459 −0.27266 −3.37209
H −4.61936 −1.87513 −3.66683
H −4.61005 −1.20995 −2.04449
24
∗ E = +4.745 kcal/mol ; (422) 059_177_268_063_268_178
H +0.00000 +0.00000 +0.00000
O +0.00000 +0.00000 +0.95462
C −0.90211 +0.00000 +1.26688
C −1.69308 −1.19335 +0.77330
H −0.83464 +0.00684 +2.35076
H −1.41498 +0.92108 +0.97405
C −3.11352 −1.26199 +1.32594
H −1.73343 −1.13401 −0.31661
H −1.14623 −2.10705 +1.00664
C −3.30741 −2.05788 +2.61007
H −3.48678 −0.24595 +1.47883
H −3.76262 −1.70058 +0.56919
C −2.57669 −1.54762 +3.84850
H −4.37756 −2.07777 +2.81494
H −3.02035 −3.09660 +2.43103
C −1.19673 −2.14542 +4.06760
H −2.50856 −0.45746 +3.81229
H −3.17541 −1.76979 +4.73305
C −0.49643 −1.56031 +5.27696
H −1.30311 −3.22437 +4.19093
H −0.57199 −2.00705 +3.18628
H +0.47463 −2.02154 +5.43963
H −0.33635 −0.48990 +5.15573
H −1.08847 −1.70271 +6.17987
24
∗ E = +4.748 kcal/mol ; (423) 181_183_092_297_091_182
H +0.00000 +0.00000 +0.00000
O +0.00000 +0.00000 +0.95357
C −0.89774 +0.00000 +1.27507
C −0.85137 +0.02828 +2.78267
H −1.43903 +0.88080 +0.91766

```
H   −1.43540  −0.88101  +0.91818
C   −2.22723  −0.03361  +3.43801
H   −0.22188  −0.78915  +3.13389
H   −0.34039  +0.94644  +3.06863
C   −2.74822  −1.41684  +3.80662
H   −2.20338  +0.55859  +4.35180
H   −2.95777  +0.45861  +2.79050
C   −2.98500  −2.38926  +2.65527
H   −2.06258  −1.87521  +4.52300
H   −3.68559  −1.27381  +4.34388
C   −1.79453  −3.26490  +2.30092
H   −3.81462  −3.05018  +2.91121
H   −3.31631  −1.83768  +1.77165
C   −2.07071  −4.16571  +1.11439
H   −0.91535  −2.65565  +2.09656
H   −1.53988  −3.87199  +3.17100
H   −1.22208  −4.80817  +0.89186
H   −2.93252  −4.80527  +1.29937
H   −2.28547  −3.58035  +0.22107
24
 * E =   +4.800  kcal/mol  ;  (424)  298_300_270_063_267_176
H   +0.00000  +0.00000  +0.00000
O   +0.00000  +0.00000  +0.95461
C   −0.90188  +0.00000  +1.26748
C   −1.66461  +1.23997  +0.84210
H   −1.42797  −0.89408  +0.92351
H   −0.82722  −0.06777  +2.34841
C   −1.07025  +2.55272  +1.33993
H   −1.68895  +1.24703  −0.25032
H   −2.70558  +1.14712  +1.15675
C   −1.56678  +3.06105  +2.68707
H   +0.01531  +2.44816  +1.36905
H   −1.27752  +3.32890  +0.60369
C   −1.26372  +2.18529  +3.90021
H   −1.12017  +4.04297  +2.84116
H   −2.64489  +3.23217  +2.63210
C   −2.36738  +1.20680  +4.26606
H   −0.32677  +1.64771  +3.73896
H   −1.08796  +2.82107  +4.76910
C   −1.98102  +0.29421  +5.41199
H   −3.26078  +1.77409  +4.53174
H   −2.64716  +0.60581  +3.40112
H   −2.79273  −0.37635  +5.68440
H   −1.11870  −0.31752  +5.15030
H   −1.71485  +0.87002  +6.29703
```

24
∗ E = +4.807 kcal/mol ; (425) 298_086_304_186_261_059
H +0.00000 +0.00000 +0.00000
O +0.00000 +0.00000 +0.95345
C −0.89823 +0.00000 +1.27320
C −1.69690 +1.22169 +0.85554
H −1.41894 −0.91361 +0.96698
H −0.80824 −0.03182 +2.35531
C −2.36989 +1.12524 −0.50468
H −2.46778 +1.40435 +1.60464
H −1.02503 +2.08056 +0.88758
C −1.43820 +0.82765 −1.66926
H −3.15306 +0.36719 −0.46390
H −2.87889 +2.06859 −0.70733
C −2.11807 +0.87871 −3.03850
H −0.61317 +1.54165 −1.64061
H −0.99796 −0.16728 −1.54728
C −2.51317 −0.47840 −3.60002
H −3.00438 +1.51333 −2.97267
H −1.45653 +1.36232 −3.75588
C −3.46921 −1.26586 −2.72615
H −2.96124 −0.33229 −4.58280
H −1.60968 −1.06810 −3.76526
H −3.76682 −2.19413 −3.20827
H −3.01573 −1.52807 −1.77193
H −4.37306 −0.69507 −2.51720
24
∗ E = +4.815 kcal/mol ; (426) 064_080_303_083_054_054
H +0.00000 +0.00000 +0.00000
O +0.00000 +0.00000 +0.95422
C −0.90258 +0.00000 +1.26387
C −1.67558 −1.24580 +0.87754
H −0.84309 +0.08884 +2.34764
H −1.42150 +0.88817 +0.89633
C −1.44474 −2.48649 +1.72757
H −2.73952 −1.00662 +0.91002
H −1.45432 −1.47130 −0.16940
C −0.01158 −2.99588 +1.84243
H −1.85059 −2.30844 +2.72497
H −2.05925 −3.28155 +1.30473
C +0.86922 −2.32407 +2.89097
H −0.05273 −4.06279 +2.06433
H +0.47958 −2.91190 +0.87208
C +0.29629 −2.30113 +4.30021
H +1.81881 −2.86266 +2.91464

```
H    +1.10928   −1.31130   +2.57815
C    −0.10825   −3.66537   +4.82383
H    +1.04211   −1.86555   +4.96508
H    −0.56178   −1.62787   +4.34461
H    −0.42210   −3.61488   +5.86403
H    −0.93505   −4.08523   +4.25323
H    +0.72145   −4.36872   +4.76122
24
 * E =  +4.817  kcal/mol ; (427) 177_076_299_095_070_066
H    +0.00000   +0.00000   +0.00000
O    +0.00000   +0.00000   +0.95363
C    −0.89836   +0.00000   +1.27357
C    −0.88420   −0.07373   +2.78130
H    −1.42625   +0.90226   +0.94798
H    −1.43764   −0.86215   +0.87467
C    −0.53695   +1.20989   +3.52084
H    −1.87315   −0.40027   +3.10465
H    −0.18778   −0.86470   +3.06287
C    +0.84942   +1.78683   +3.23495
H    −1.30131   +1.95896   +3.30361
H    −0.63044   +1.00061   +4.58616
C    +0.87234   +2.86540   +2.16033
H    +1.26684   +2.19721   +4.15417
H    +1.51277   +0.97825   +2.93294
C    +0.23376   +4.18705   +2.56244
H    +1.90839   +3.05567   +1.87410
H    +0.38303   +2.48215   +1.26600
C    +0.96841   +4.91540   +3.67187
H    +0.19152   +4.83187   +1.68429
H    −0.80334   +4.02602   +2.86072
H    +0.51521   +5.88303   +3.87587
H    +0.96091   +4.35194   +4.60227
H    +2.00935   +5.08777   +3.40128
24
 * E =  +4.833  kcal/mol ; (428) 289_289_073_094_298_297
H    +0.00000   +0.00000   +0.00000
O    +0.00000   +0.00000   +0.95417
C    −0.90127   +0.00000   +1.26748
C    −1.63636   +1.30895   +1.05764
H    −1.46145   −0.81408   +0.80136
H    −0.83966   −0.22212   +2.33115
C    −1.17535   +2.46075   +1.94256
H    −1.58536   +1.57863   +0.00168
H    −2.69060   +1.11605   +1.26504
C    +0.17379   +3.09864   +1.61008
```

H −1.94143 +3.23930 +1.92957
H −1.15403 +2.08960 +2.96849
C +0.08426 +4.32301 +0.70894
H +0.64458 +3.41751 +2.53962
H +0.83998 +2.35043 +1.18671
C −0.49498 +4.08986 −0.67689
H −0.51742 +5.08035 +1.21545
H +1.08241 +4.75217 +0.60163
C +0.33769 +3.15554 −1.53221
H −1.51367 +3.70882 −0.59028
H −0.58317 +5.05077 −1.18414
H −0.09962 +3.01986 −2.51896
H +1.34527 +3.54626 −1.66569
H +0.43372 +2.17417 −1.07172
24
* E = +4.862 kcal/mol ; (429) 058_295_298_094_094_300
H +0.00000 +0.00000 +0.00000
O +0.00000 +0.00000 +0.95468
C −0.90160 +0.00000 +1.26857
C −1.70594 −1.17784 +0.76426
H −0.83067 −0.03778 +2.35215
H −1.40084 +0.93923 +1.01203
C −1.92730 −1.20407 −0.74229
H −1.20414 −2.09372 +1.07542
H −2.67791 −1.16267 +1.26102
C −2.70842 −0.00360 −1.27802
H −0.96775 −1.28612 −1.25662
H −2.45750 −2.12442 −0.98354
C −1.86305 +1.16700 −1.78194
H −3.35817 −0.32582 −2.09316
H −3.37996 +0.34329 −0.49125
C −1.58643 +1.13382 −3.27722
H −2.36512 +2.10603 −1.55128
H −0.91271 +1.20657 −1.24470
C −0.84716 −0.10011 −3.75537
H −2.53725 +1.20855 −3.80777
H −1.01749 +2.02272 −3.54965
H −0.64816 −0.04788 −4.82317
H +0.11153 −0.20863 −3.24849
H −1.42106 −1.00688 −3.57369
24
* E = +4.865 kcal/mol ; (430) 298_285_055_064_101_299
H +0.00000 +0.00000 +0.00000
O +0.00000 +0.00000 +0.95440
C −0.90232 +0.00000 +1.26537

C	−1.69025	+1.22385	+0.84382
H	−1.41486	−0.90536	+0.93168
H	−0.83630	−0.05057	+2.35091
C	−1.36129	+2.50004	+1.60565
H	−1.55400	+1.36916	−0.23046
H	−2.75127	+1.00785	+0.97566
C	+0.10600	+2.90793	+1.61007
H	−1.96916	+3.30869	+1.19812
H	−1.68613	+2.36930	+2.63962
C	+0.68452	+3.25585	+0.23824
H	+0.22098	+3.76837	+2.27277
H	+0.68639	+2.10256	+2.05321
C	+0.79310	+4.74840	−0.03306
H	+1.67389	+2.81150	+0.14031
H	+0.07831	+2.80122	−0.54830
C	−0.53386	+5.48082	+0.00110
H	+1.46773	+5.19243	+0.70115
H	+1.26251	+4.89945	−1.00554
H	−0.41206	+6.53224	−0.24962
H	−1.23820	+5.04970	−0.70972
H	−0.99048	+5.43248	+0.98793

24

* E = +4.868 kcal/mol ; (431) 292_297_175_267_062_270

H	+0.00000	+0.00000	+0.00000
O	+0.00000	+0.00000	+0.95449
C	−0.90235	+0.00000	+1.26564
C	−1.62311	+1.29670	+0.97025
H	−1.45289	−0.84572	+0.84752
H	−0.83860	−0.15536	+2.34077
C	−1.00476	+2.48954	+1.67875
H	−1.64404	+1.45330	−0.10981
H	−2.66580	+1.19391	+1.27713
C	−1.63007	+3.83344	+1.31477
H	−1.11394	+2.32320	+2.75148
H	+0.06910	+2.50899	+1.49250
C	−0.98071	+4.60865	+0.17567
H	−2.68785	+3.68401	+1.08381
H	−1.60992	+4.47845	+2.19297
C	−0.97731	+3.94167	−1.19701
H	−1.50124	+5.56342	+0.10011
H	+0.04780	+4.85196	+0.45227
C	+0.23988	+3.07478	−1.46636
H	−1.89290	+3.36063	−1.32110
H	−1.01545	+4.71393	−1.96502
H	+0.17835	+2.59823	−2.44308

```
H    +1.14600   +3.67809   -1.45271
H    +0.36615   +2.29704   -0.71746
24
* E =  +4.871  kcal/mol ; (432) 062_177_091_297_092_184
H    +0.00000   +0.00000   +0.00000
O    +0.00000   +0.00000   +0.95445
C    -0.90183   +0.00000   +1.26697
C    -1.66428   -1.23833   +0.84438
H    -0.83505   +0.02950   +2.35227
H    -1.41739   +0.91061   +0.95487
C    -3.08775   -1.30816   +1.39013
H    -1.68197   -1.30962   -0.24604
H    -1.09026   -2.09569   +1.19337
C    -4.18969   -0.71096   +0.52461
H    -3.34037   -2.35347   +1.56296
H    -3.11511   -0.83403   +2.37449
C    -4.10864   +0.78858   +0.25490
H    -4.22132   -1.24305   -0.42917
H    -5.13704   -0.93041   +1.01625
C    -3.36822   +1.16607   -1.01711
H    -5.11778   +1.19628   +0.18106
H    -3.65422   +1.29188   +1.11164
C    -3.23713   +2.66501   -1.19265
H    -2.37848   +0.71023   -1.02954
H    -3.90045   +0.73972   -1.86876
H    -2.73718   +2.91946   -2.12425
H    -4.21485   +3.14419   -1.19956
H    -2.66391   +3.10498   -0.37777
24
* E =  +4.880  kcal/mol ; (433) 068_275_060_058_064_268
H    +0.00000   +0.00000   +0.00000
O    +0.00000   +0.00000   +0.95313
C    -0.89737   +0.00000   +1.27435
C    -1.65144   -1.28894   +1.00285
H    -0.80878   +0.15223   +2.34646
H    -1.45219   +0.85425   +0.87327
C    -2.30118   -1.38780   -0.36962
H    -0.96257   -2.11695   +1.17127
H    -2.43693   -1.38606   +1.75330
C    -1.36460   -1.28710   -1.56390
H    -2.85799   -2.32306   -0.43237
H    -3.04811   -0.59640   -0.45505
C    -0.24731   -2.31940   -1.63901
H    -1.96227   -1.36391   -2.47385
H    -0.93531   -0.28179   -1.60273
```

```
C  −0.71338  −3.76498  −1.80033
H  +0.37874  −2.05662  −2.49164
H  +0.39651  −2.24535  −0.76028
C  −0.91175  −4.52636  −0.50190
H  −1.63618  −3.78265  −2.38402
H  +0.02302  −4.30400  −2.39537
H  −1.17218  −5.56469  −0.69611
H  +0.00249  −4.52212  +0.08963
H  −1.70090  −4.10310  +0.11335
24
* E =  +4.946  kcal/mol ; (434) 291_061_268_178_263_060
H  +0.00000  +0.00000  +0.00000
O  +0.00000  +0.00000  +0.95433
C  −0.90152  +0.00000  +1.26740
C  −1.64729  +1.29103  +1.00448
H  −1.45479  −0.83604  +0.82897
H  −0.83974  −0.18098  +2.33740
C  −1.78248  +1.65044  −0.47173
H  −2.63651  +1.18253  +1.45059
H  −1.15277  +2.10442  +1.53688
C  −0.66125  +2.53261  −1.00523
H  −1.86637  +0.73614  −1.06460
H  −2.72111  +2.18176  −0.63135
C  −0.77273  +2.84898  −2.49504
H  −0.67695  +3.45947  −0.43142
H  +0.31240  +2.08657  −0.79473
C  +0.06544  +1.95993  −3.40122
H  −1.82062  +2.78797  −2.79864
H  −0.47176  +3.88112  −2.67025
C  −0.26406  +0.48264  −3.32511
H  −0.05581  +2.29693  −4.43074
H  +1.11942  +2.10488  −3.15848
H  +0.32091  −0.08907  −4.04163
H  −0.04951  +0.07201  −2.33925
H  −1.31752  +0.29995  −3.53215
24
* E =  +4.970  kcal/mol ; (435) 178_177_060_267_264_060
H  +0.00000  +0.00000  +0.00000
O  +0.00000  +0.00000  +0.95351
C  −0.89825  +0.00000  +1.27341
C  −0.84823  −0.06217  +2.77798
H  −1.42319  +0.90652  +0.95575
H  −1.44790  −0.85761  +0.87691
C  −2.20765  −0.00357  +3.45590
H  −0.33480  −0.98145  +3.06201
```

H −0.22550 +0.76154 +3.12520
C −3.15067 −1.14143 +3.06430
H −2.04039 −0.01938 +4.53204
H −2.68073 +0.95566 +3.23992
C −4.09194 −0.82877 +1.89932
H −2.54829 −2.02043 +2.82970
H −3.75723 −1.42715 +3.92518
C −5.47537 −0.37091 +2.33572
H −3.65099 −0.05825 +1.26369
H −4.20713 −1.71052 +1.26934
C −5.47713 +0.88921 +3.17847
H −6.09160 −0.21293 +1.45038
H −5.95064 −1.17822 +2.89584
H −6.49054 +1.18719 +3.43739
H −4.92945 +0.74878 +4.10870
H −5.01383 +1.71911 +2.64609
24
* E = +4.979 kcal/mol ; (436) 180_066_279_288_260_061
H +0.00000 +0.00000 +0.00000
O +0.00000 +0.00000 +0.95354
C −0.89792 +0.00000 +1.27443
C −0.89619 −0.00053 +2.78302
H −1.43271 +0.88312 +0.91216
H −1.43001 −0.88360 +0.91334
C −0.33825 +1.26248 +3.42755
H −1.93119 −0.13806 +3.09743
H −0.35041 −0.87888 +3.12911
C +1.18329 +1.32624 +3.50301
H −0.71226 +2.12748 +2.87606
H −0.74693 +1.34719 +4.43464
C +1.79745 +0.36970 +4.52671
H +1.58298 +1.12348 +2.51380
H +1.48031 +2.34702 +3.75335
C +2.18402 +1.02810 +5.84236
H +1.10268 −0.44813 +4.73171
H +2.68605 −0.09859 +4.10562
C +1.02534 +1.66322 +6.58572
H +2.65831 +0.28535 +6.48452
H +2.94308 +1.78760 +5.64583
H +1.34813 +2.07936 +7.53758
H +0.57846 +2.47245 +6.01104
H +0.24320 +0.93306 +6.79106
24
* E = +5.001 kcal/mol ; (437) 059_179_300_094_096_300
H +0.00000 +0.00000 +0.00000

```
O    +0.00000    +0.00000    +0.95452
C    −0.90208    +0.00000    +1.26654
C    −1.68719    −1.19200    +0.76759
H    −0.83297    −0.01491    +2.35138
H    −1.40990    +0.92909    +0.98812
C    −3.12745    −1.24602    +1.25426
H    −1.68007    −1.18209    −0.32505
H    −1.16149    −2.09750    +1.07307
C    −3.26304    −1.31753    +2.77565
H    −3.67075    −0.37816    +0.87731
H    −3.59996    −2.11624    +0.80058
C    −3.44489    +0.03198    +3.47289
H    −4.10951    −1.95412    +3.03762
H    −2.38389    −1.82577    +3.17458
C    −4.89764    +0.38531    +3.75318
H    −2.90427    +0.03475    +4.41890
H    −2.99938    +0.82441    +2.86853
C    −5.76831    +0.47273    +2.51519
H    −5.31426    −0.36015    +4.43294
H    −4.93380    +1.33532    +4.28651
H    −6.78696    +0.75543    +2.77060
H    −5.38446    +1.21465    +1.81584
H    −5.81518    −0.48039    +1.99120
24
 * E =   +5.020  kcal/mol ; (438) 299_087_305_183_059_260
H    +0.00000    +0.00000    +0.00000
O    +0.00000    +0.00000    +0.95342
C    −0.89857    +0.00000    +1.27214
C    −1.69813    +1.21813    +0.84560
H    −1.41679    −0.91654    +0.97077
H    −0.80949    −0.02534    +2.35444
C    −2.39350    +1.10319    −0.50300
H    −2.45577    +1.42056    +1.60294
H    −1.02081    +2.07316    +0.85138
C    −1.48699    +0.73839    −1.66423
H    −3.19126    +0.35960    −0.43493
H    −2.88197    +2.05416    −0.71455
C    −2.17733    +0.69632    −3.02019
H    −0.65840    +1.45055    −1.71102
H    −1.05194    −0.24860    −1.48636
C    −2.82449    +2.01367    −3.44872
H    −1.43932    +0.39137    −3.76091
H    −2.93463    −0.09050    −3.01150
C    −4.32270    +2.06724    −3.21077
H    −2.33789    +2.84154    −2.92906
```

H −2.63922 +2.18201 −4.50863
H −4.73739 +3.03088 −3.49875
H −4.83038 +1.30178 −3.79529
H −4.57386 +1.89614 −2.16645
24
* E = +5.022 kcal/mol ; (439) 070_295_177_267_062_271
H +0.00000 +0.00000 +0.00000
O +0.00000 +0.00000 +0.95382
C −0.89996 +0.00000 +1.26980
C −1.61578 −1.30755 +1.01239
H −0.83386 +0.18668 +2.33813
H −1.46132 +0.82930 +0.82893
C −1.81292 −1.60513 −0.46419
H −1.06059 −2.10844 +1.49809
H −2.59337 −1.26708 +1.49630
C −2.47494 −2.95069 −0.75044
H −2.42281 −0.80486 −0.88757
H −0.85649 −1.56626 −0.99104
C −1.54278 −4.12985 −0.99417
H −3.15039 −3.19440 +0.07284
H −3.10758 −2.85158 −1.63217
C −0.61400 −4.52993 +0.14933
H −2.17018 −4.98388 −1.24963
H −0.94065 −3.92861 −1.88362
C +0.73260 −3.82758 +0.15315
H −1.12290 −4.37694 +1.10278
H −0.43020 −5.60227 +0.08551
H +1.36104 −4.19363 +0.96239
H +1.26033 −4.01607 −0.78081
H +0.65086 −2.75047 +0.27329
24
* E = +5.023 kcal/mol ; (440) 181_061_178_268_062_270
H +0.00000 +0.00000 +0.00000
O +0.00000 +0.00000 +0.95329
C −0.89758 +0.00000 +1.27441
C −0.86176 +0.03549 +2.78057
H −1.43942 +0.87369 +0.89897
H −1.42733 −0.89345 +0.93589
C −0.18138 +1.27869 +3.32715
H −1.88380 −0.04910 +3.14720
H −0.32936 −0.84831 +3.13437
C −0.16732 +1.36582 +4.85033
H +0.83922 +1.27918 +2.95024
H −0.65201 +2.17264 +2.91094
C −1.32575 +2.10334 +5.50927

```
H    −0.10942    +0.35566    +5.26304
H    +0.74969    +1.86374    +5.16480
C    −2.72383    +1.52936    +5.29400
H    −1.12058    +2.13138    +6.57953
H    −1.31784    +3.14498    +5.17907
C    −3.45090    +2.08146    +4.08066
H    −2.66612    +0.44094    +5.23803
H    −3.33149    +1.74500    +6.17267
H    −4.43890    +1.63752    +3.97607
H    −3.58267    +3.15819    +4.17476
H    −2.90979    +1.90342    +3.15482
24
 * E =  +5.137  kcal/mol ; (441) 060_175_060_266_264_060
H    +0.00000    +0.00000    +0.00000
O    +0.00000    +0.00000    +0.95450
C    −0.90253    +0.00000    +1.26517
C    −1.67190    −1.21639    +0.80132
H    −0.83900    +0.01237    +2.35155
H    −1.41230    +0.91779    +0.96101
C    −3.08007    −1.32973    +1.36548
H    −1.72941    −1.20033    −0.29087
H    −1.09396    −2.10039    +1.06916
C    −3.99195    −0.15380    +1.01377
H    −3.51384    −2.25657    +0.99231
H    −3.02428    −1.43965    +2.44937
C    −4.03518    +0.96555    +2.05584
H    −3.67757    +0.24874    +0.04933
H    −5.00989    −0.51444    +0.85812
C    −5.20533    +0.85726    +3.02178
H    −3.10549    +0.97531    +2.62767
H    −4.09218    +1.93294    +1.55752
C    −5.22792    −0.42341    +3.83272
H    −5.18489    +1.71155    +3.69855
H    −6.13516    +0.94056    +2.45616
H    −6.06614    −0.43465    +4.52557
H    −5.32030    −1.29998    +3.19376
H    −4.31486    −0.53590    +4.41617
24
 * E =  +5.156  kcal/mol ; (442) 299_185_187_093_298_089
H    +0.00000    +0.00000    +0.00000
O    +0.00000    +0.00000    +0.95440
C    −0.90208    +0.00000    +1.26606
C    −1.68091    +1.22022    +0.83264
H    −1.42386    −0.90735    +0.94501
H    −0.83822    −0.03683    +2.35139
```

C −3.07594 +1.25918 +1.43303
H −1.12916 +2.11169 +1.13116
H −1.73409 +1.24283 −0.25708
C −3.95886 +2.38035 +0.89192
H −3.57589 +0.29916 +1.28570
H −2.97058 +1.37239 +2.51244
C −4.84950 +2.03488 −0.29426
H −4.61088 +2.73215 +1.69091
H −3.32815 +3.23244 +0.62828
C −4.15496 +1.61088 −1.58534
H −5.54981 +1.25074 +0.00358
H −5.46042 +2.91361 −0.50092
C −3.88027 +0.12173 −1.69935
H −4.78337 +1.89735 −2.42834
H −3.22965 +2.17728 −1.70449
H −3.40237 −0.11851 −2.64696
H −3.23780 −0.24859 −0.90434
H −4.81083 −0.44134 −1.64981
24
* E = +5.157 kcal/mol ; (443) 181_183_186_093_298_089
H +0.00000 +0.00000 +0.00000
O +0.00000 +0.00000 +0.95348
C −0.89747 +0.00000 +1.27545
C −0.86086 +0.01856 +2.78041
H −1.44101 +0.87732 +0.91099
H −1.43508 −0.88781 +0.92959
C −2.24833 −0.05257 +3.39376
H −0.26669 −0.82859 +3.12307
H −0.33194 +0.91381 +3.10175
C −2.26547 +0.11923 +4.91025
H −2.90277 +0.69322 +2.93667
H −2.68111 −1.02021 +3.13687
C −2.47647 +1.53367 +5.43417
H −3.06040 −0.49909 +5.32657
H −1.33489 −0.27813 +5.32172
C −1.42217 +2.57507 +5.06940
H −3.45510 +1.89199 +5.10531
H −2.53664 +1.46551 +6.52048
C −1.67681 +3.30676 +3.76346
H −1.38020 +3.31947 +5.86433
H −0.43667 +2.10682 +5.05345
H −0.90504 +4.04953 +3.57310
H −1.70274 +2.63807 +2.90693
H −2.63291 +3.82684 +3.79900
24

∗ E = +5.165 kcal/mol ; (444) 069_300_093_187_301_096

H	+0.00000	+0.00000	+0.00000
O	+0.00000	+0.00000	+0.95427
C	−0.90135	+0.00000	+1.26764
C	−1.64662	−1.28944	+0.99464
H	−0.84004	+0.17535	+2.33850
H	−1.45316	+0.83876	+0.83263
C	−1.74891	−1.64366	−0.48644
H	−1.16124	−2.10399	+1.53342
H	−2.64275	−1.18064	+1.42438
C	−0.63416	−2.55618	−0.97329
H	−2.70470	−2.12978	−0.67846
H	−1.76409	−0.72507	−1.08312
C	−0.61309	−2.80125	−2.47365
H	+0.33092	−2.15884	−0.65894
H	−0.73251	−3.51563	−0.46045
C	−1.88771	−3.42518	−3.04098
H	−0.41305	−1.86138	−2.99435
H	+0.23586	−3.44811	−2.69187
C	−2.87324	−2.41971	−3.60892
H	−1.62532	−4.12790	−3.83086
H	−2.37274	−4.02064	−2.26460
H	−3.76746	−2.91131	−3.98624
H	−3.18614	−1.68963	−2.86650
H	−2.42584	−1.87096	−4.43613

24

∗ E = +5.227 kcal/mol ; (445) 299_180_174_267_062_271

H	+0.00000	+0.00000	+0.00000
O	+0.00000	+0.00000	+0.95435
C	−0.90206	+0.00000	+1.26591
C	−1.68303	+1.21590	+0.82383
H	−1.42061	−0.91245	+0.95617
H	−0.83710	−0.02391	+2.35194
C	−3.11546	+1.20017	+1.32966
H	−1.15497	+2.10709	+1.15795
H	−1.69683	+1.25942	−0.26835
C	−3.89903	+2.47446	+1.02660
H	−3.62351	+0.34847	+0.87566
H	−3.12966	+1.01123	+2.40521
C	−3.88596	+3.55449	+2.10028
H	−3.53465	+2.90019	+0.08875
H	−4.94147	+2.21283	+0.84666
C	−2.53260	+4.16219	+2.45854
H	−4.54615	+4.35156	+1.75815
H	−4.34643	+3.15790	+3.00840

C −1.78266 +3.43738 +3.56216
H −1.91367 +4.22919 +1.56217
H −2.68913 +5.19060 +2.78342
H −0.83935 +3.93102 +3.78547
H −2.37231 +3.42483 +4.47751
H −1.55640 +2.40505 +3.30831
24
∗ E = +5.235 kcal/mol ; (446) 070_301_093_181_261_059
H +0.00000 +0.00000 +0.00000
O +0.00000 +0.00000 +0.95419
C −0.90218 +0.00000 +1.26491
C −1.63796 −1.29844 +1.00948
H −0.84592 +0.19271 +2.33309
H −1.45719 +0.82865 +0.81483
C −1.72390 −1.68456 −0.46447
H −1.15538 −2.09896 +1.57118
H −2.63951 −1.18591 +1.42576
C −0.59891 −2.59603 −0.93512
H −2.67657 −2.17289 −0.66288
H −1.73460 −0.77861 −1.07935
C −0.68113 −2.96974 −2.41508
H +0.35842 −2.12490 −0.71727
H −0.61001 −3.50537 −0.32933
C −1.28924 −4.33711 −2.68819
H −1.25679 −2.20942 −2.94856
H +0.31575 −2.95069 −2.85386
C −2.70588 −4.50765 −2.17663
H −1.27037 −4.52141 −3.76245
H −0.65170 −5.10055 −2.23906
H −3.10749 −5.48066 −2.45034
H −2.75049 −4.43243 −1.09166
H −3.36809 −3.74747 −2.58938
24
∗ E = +5.242 kcal/mol ; (447) 294_061_172_267_062_271
H +0.00000 +0.00000 +0.00000
O +0.00000 +0.00000 +0.95405
C −0.90090 +0.00000 +1.26803
C −1.65265 +1.26770 +0.92781
H −1.44262 −0.86731 +0.87619
H −0.83521 −0.12108 +2.34581
C −1.77255 +1.51128 −0.56790
H −2.64011 +1.21084 +1.38502
H −1.14425 +2.11594 +1.38713
C −2.68740 +2.67508 −0.94112
H −0.77463 +1.71304 −0.95944

H	−2.11749	+0.60231	−1.06858
C	−4.14341	+2.33313	−1.22909
H	−2.64675	+3.42356	−0.14659
H	−2.29141	+3.16627	−1.82949
C	−4.95603	+1.72689	−0.08831
H	−4.62903	+3.25478	−1.54971
H	−4.18706	+1.66096	−2.08951
C	−4.89792	+0.21204	−0.00110
H	−4.64575	+2.17308	+0.85810
H	−6.00013	+2.01160	−0.21649
H	−5.51243	−0.15801	+0.81695
H	−5.27089	−0.23618	−0.92063
H	−3.88890	−0.16141	+0.15401

24

* E = +5.269 kcal/mol ; (448) 065_059_181_269_062_268

H	+0.00000	+0.00000	+0.00000
O	+0.00000	+0.00000	+0.95452
C	−0.90230	+0.00000	+1.26592
C	−1.65674	−1.25858	+0.89680
H	−0.83418	+0.08988	+2.34826
H	−1.43565	+0.88071	+0.90033
C	−1.01740	−2.51851	+1.45355
H	−2.68626	−1.15242	+1.23757
H	−1.71029	−1.33698	−0.19287
C	−1.72991	−3.81309	+1.07344
H	+0.00935	−2.54807	+1.09290
H	−0.94260	−2.44278	+2.54055
C	−2.80501	−4.31562	+2.02810
H	−2.16589	−3.69970	+0.07768
H	−0.98812	−4.60612	+0.98036
C	−4.01093	−3.40594	+2.24982
H	−3.15371	−5.27342	+1.64187
H	−2.34655	−4.53587	+2.99510
C	−3.86724	−2.44995	+3.42065
H	−4.22425	−2.85334	+1.33318
H	−4.89051	−4.02361	+2.42978
H	−4.74750	−1.81937	+3.52779
H	−3.74481	−3.00409	+4.34979
H	−3.00388	−1.79698	+3.32063

24

* E = +5.297 kcal/mol ; (449) 067_293_271_063_266_175

H	+0.00000	+0.00000	+0.00000
O	+0.00000	+0.00000	+0.95401
C	−0.90026	+0.00000	+1.26970
C	−1.63296	−1.28847	+0.94601

```
H    -0.82709   +0.11367   +2.34813
H    -1.43486   +0.87215   +0.88977
C    -1.88767   -1.54531   -0.53609
H    -1.02653   -2.09785   +1.35095
H    -2.57895   -1.31020   +1.48955
C    -3.17714   -0.98778   -1.12400
H    -1.04304   -1.17585   -1.12502
H    -1.89220   -2.62187   -0.70223
C    -3.33385   +0.53052   -1.11188
H    -3.24120   -1.34351   -2.15190
H    -4.02627   -1.43609   -0.60279
C    -4.07748   +1.07849   +0.09457
H    -2.35171   +1.00404   -1.18756
H    -3.87483   +0.84579   -2.00497
C    -4.10199   +2.59267   +0.12621
H    -5.09784   +0.69269   +0.07689
H    -3.63530   +0.70186   +1.01634
H    -4.67007   +2.96828   +0.97387
H    -3.09327   +2.99750   +0.19824
H    -4.55160   +2.99595   -0.77984
24
 * E =   +5.300  kcal/mol ;  (450) 291_060_267_182_300_098
H    +0.00000   +0.00000   +0.00000
O    +0.00000   +0.00000   +0.95419
C    -0.90189   +0.00000   +1.26574
C    -1.64417   +1.29203   +0.99742
H    -1.45427   -0.83594   +0.82598
H    -0.84351   -0.18020   +2.33600
C    -1.74852   +1.65514   -0.48168
H    -2.64142   +1.18208   +1.42452
H    -1.15903   +2.10271   +1.54199
C    -0.63204   +2.56145   -0.97475
H    -1.77644   +0.73616   -1.07440
H    -2.69986   +2.15362   -0.67258
C    -0.74389   +2.97398   -2.43369
H    -0.62118   +3.45837   -0.35360
H    +0.33562   +2.08510   -0.80993
C    -0.73218   +1.81444   -3.43025
H    -1.65651   +3.55756   -2.57521
H    +0.08026   +3.65190   -2.65206
C    -2.11221   +1.39008   -3.89896
H    -0.14354   +2.08976   -4.30429
H    -0.21468   +0.96083   -2.98597
H    -2.05891   +0.54358   -4.58033
H    -2.75516   +1.10819   -3.06841
```

```
H    -2.60362   +2.20640   -4.42559
24
* E =  +5.536  kcal/mol ; (451) 072_288_076_058_262_290
H    +0.00000   +0.00000   +0.00000
O    +0.00000   +0.00000   +0.95320
C    -0.89769   +0.00000   +1.27373
C    -1.63829   -1.30747   +1.08115
H    -0.82084   +0.21767   +2.33598
H    -1.46804   +0.81604   +0.81955
C    -2.04643   -1.64074   -0.34722
H    -1.05073   -2.11826   +1.51206
H    -2.54689   -1.24049   +1.68190
C    -0.96392   -2.13207   -1.30296
H    -2.80979   -2.41910   -0.30392
H    -2.53954   -0.76822   -0.78230
C    -0.23440   -3.39297   -0.83974
H    -1.45055   -2.32720   -2.25809
H    -0.24008   -1.34520   -1.51980
C    +1.10042   -3.15647   -0.14530
H    -0.89499   -3.95285   -0.17466
H    -0.05770   -4.04403   -1.69702
C    +2.18392   -2.66115   -1.08375
H    +0.98187   -2.44112   +0.66621
H    +1.42828   -4.09277   +0.30675
H    +3.12359   -2.51497   -0.55679
H    +2.35972   -3.37046   -1.89159
H    +1.92231   -1.70767   -1.54049
24
* E =  +5.539  kcal/mol ; (452) 182_063_075_093_297_089
H    +0.00000   +0.00000   +0.00000
O    +0.00000   +0.00000   +0.95325
C    -0.89699   +0.00000   +1.27588
C    -0.85342   +0.04967   +2.78356
H    -1.44516   +0.86083   +0.88333
H    -1.42154   -0.90257   +0.95437
C    -0.21173   +1.32284   +3.31939
H    -1.87156   -0.06149   +3.15548
H    -0.29301   -0.82001   +3.12604
C    -1.08936   +2.56846   +3.22331
H    +0.08903   +1.17527   +4.35678
H    +0.70923   +1.47629   +2.75982
C    -1.93032   +2.90284   +4.44854
H    -0.45628   +3.43195   +3.02058
H    -1.75071   +2.48420   +2.35743
C    -2.99567   +1.88831   +4.85540
```

H −1.26650 +3.07772 +5.29878
H −2.41834 +3.85789 +4.25281
C −2.51466 +0.80354 +5.80282
H −3.81203 +2.41913 +5.34507
H −3.43285 +1.44118 +3.96052
H −3.32667 +0.13242 +6.07541
H −1.71868 +0.19903 +5.37706
H −2.13141 +1.24473 +6.72160
24
* E = +5.550 kcal/mol ; (453) 180_286_061_268_263_060
H +0.00000 +0.00000 +0.00000
O +0.00000 +0.00000 +0.95354
C −0.89805 +0.00000 +1.27408
C −0.88101 −0.00123 +2.78325
H −1.42345 +0.88991 +0.91962
H −1.44223 −0.87540 +0.90546
C −0.50805 −1.31448 +3.45561
H −0.19647 +0.78531 +3.10414
H −1.87446 +0.29249 +3.12391
C +0.89056 −1.84090 +3.13624
H −0.60102 −1.16201 +4.53105
H −1.25274 −2.06915 +3.19623
C +0.95947 −2.80057 +1.94838
H +1.54516 −0.99042 +2.95553
H +1.28974 −2.35300 +4.01402
C +0.94288 −4.26947 +2.34192
H +0.12926 −2.60066 +1.26911
H +1.86255 −2.60437 +1.37291
C −0.29796 −4.69965 +3.09988
H +1.04384 −4.87898 +1.44327
H +1.82394 −4.48065 +2.95108
H −0.27069 −5.76232 +3.33093
H −0.39614 −4.16287 +4.04194
H −1.19987 −4.51039 +2.51842
24
* E = +5.580 kcal/mol ; (454) 069_299_092_066_057_264
H +0.00000 +0.00000 +0.00000
O +0.00000 +0.00000 +0.95415
C −0.90185 +0.00000 +1.26571
C −1.64109 −1.29389 +0.99761
H −0.84344 +0.18128 +2.33574
H −1.45487 +0.83497 +0.82479
C −1.75076 −1.64543 −0.48416
H −1.15281 −2.10118 +1.54288
H −2.63739 −1.18960 +1.42932

C −0.63818 −2.53443 −1.02609
H −2.70509 −2.13334 −0.67986
H −1.78451 −0.72047 −1.06716
C −0.58669 −3.94591 −0.45517
H −0.75931 −2.61018 −2.10818
H +0.33112 −2.06590 −0.85593
C −1.87176 −4.75462 −0.62329
H +0.23604 −4.46267 −0.94772
H −0.31782 −3.91330 +0.60182
C −2.78423 −4.72509 +0.58976
H −2.41242 −4.39739 −1.50234
H −1.61974 −5.79313 −0.83461
H −3.69208 −5.29944 +0.41772
H −2.28251 −5.15355 +1.45586
H −3.07960 −3.71314 +0.85661
24
* E = +5.598 kcal/mol ; (455) 180_182_093_296_091_072
H +0.00000 +0.00000 +0.00000
O +0.00000 +0.00000 +0.95355
C −0.89766 +0.00000 +1.27521
C −0.84986 +0.00077 +2.78299
H −1.43287 +0.89098 +0.93412
H −1.44077 −0.87091 +0.90350
C −2.22536 −0.05304 +3.44061
H −0.23170 −0.83191 +3.11859
H −0.32499 +0.90610 +3.08428
C −2.76136 −1.43540 +3.79096
H −2.19128 +0.52547 +4.36282
H −2.95141 +0.45771 +2.80237
C −3.03225 −2.37548 +2.61866
H −2.07328 −1.91227 +4.49133
H −3.68845 −1.29017 +4.34507
C −1.86984 −3.27704 +2.22021
H −3.87694 −3.02035 +2.86666
H −3.35818 −1.78593 +1.75974
C −1.59794 −4.37823 +3.22762
H −2.09311 −3.73202 +1.25476
H −0.96343 −2.69068 +2.07519
H −0.77050 −5.00807 +2.90936
H −1.34276 −3.97748 +4.20649
H −2.47204 −5.01571 +3.35226
24
* E = +5.603 kcal/mol ; (456) 058_178_269_064_267_288
H +0.00000 +0.00000 +0.00000
O +0.00000 +0.00000 +0.95461

```
C   -0.90215  +0.00000  +1.26672
C   -1.69409  -1.18891  +0.76400
H   -0.83438  -0.00069  +2.35019
H   -1.41316  +0.92368  +0.97864
C   -3.12424  -1.25135  +1.29243
H   -1.71770  -1.12980  -0.32649
H   -1.15581  -2.10582  +1.00514
C   -3.34154  -2.03653  +2.57952
H   -3.50001  -0.23387  +1.42893
H   -3.75871  -1.69703  +0.52741
C   -2.64973  -1.49330  +3.82805
H   -4.41614  -2.07012  +2.75694
H   -3.03925  -3.07244  +2.41503
C   -1.27394  -2.08245  +4.11554
H   -2.58324  -0.40656  +3.75328
H   -3.27856  -1.68661  +4.69850
C   -1.33387  -3.51478  +4.61169
H   -0.64564  -2.03555  +3.22684
H   -0.77676  -1.46984  +4.86769
H   -0.33840  -3.90651  +4.80705
H   -1.90488  -3.58205  +5.53651
H   -1.80639  -4.17495  +3.88696
24
 * E =   +5.633  kcal/mol ;  (457)  292_060_267_285_261_060
H   +0.00000  +0.00000  +0.00000
O   +0.00000  +0.00000  +0.95436
C   -0.90214  +0.00000  +1.26573
C   -1.64769  +1.28743  +0.98337
H   -1.45108  -0.84189  +0.83313
H   -0.84320  -0.17033  +2.33755
C   -1.73747  +1.63173  -0.50217
H   -2.64669  +1.17936  +1.40662
H   -1.16745  +2.10250  +1.52557
C   -0.62034  +2.55028  -0.99025
H   -1.73927  +0.70529  -1.08395
H   -2.69533  +2.10521  -0.70996
C   -0.78406  +4.00622  -0.55029
H   +0.33248  +2.17017  -0.62567
H   -0.56796  +2.51405  -2.08029
C   -1.39199  +4.91315  -1.60966
H   -1.39886  +4.04740  +0.35174
H   +0.18423  +4.41656  -0.26753
C   -2.77614  +4.50219  -2.07196
H   -1.43121  +5.93074  -1.22064
H   -0.72184  +4.94444  -2.47063
```

H −3.17536 +5.21134 −2.79355
H −2.76465 +3.52421 −2.54983
H −3.47229 +4.45387 −1.23539
24
∗ E = +5.637 kcal/mol ; (458) 064_061_077_094_297_088
H +0.00000 +0.00000 +0.00000
O +0.00000 +0.00000 +0.95457
C −0.90221 +0.00000 +1.26639
C −1.66103 −1.25108 +0.87417
H −0.83263 +0.08086 +2.34877
H −1.43276 +0.88634 +0.91088
C −1.04674 −2.52125 +1.44718
H −2.69584 −1.13771 +1.19801
H −1.68917 −1.30938 −0.21672
C −1.33751 −2.74817 +2.92888
H −1.38194 −3.38886 +0.87864
H +0.02939 −2.45674 +1.29210
C −2.54612 −3.61484 +3.25779
H −0.46867 −3.21492 +3.39171
H −1.44978 −1.78397 +3.43033
C −3.91312 −3.09281 +2.82373
H −2.39694 −4.61015 +2.83205
H −2.55645 −3.75406 +4.33893
C −4.33017 −3.48633 +1.41765
H −4.66574 −3.47573 +3.51278
H −3.94020 −2.00717 +2.93542
H −5.32364 −3.10933 +1.18333
H −3.64984 −3.11024 +0.65861
H −4.35917 −4.57040 +1.31954
24
∗ E = +5.725 kcal/mol ; (459) 063_177_091_296_093_073
H +0.00000 +0.00000 +0.00000
O +0.00000 +0.00000 +0.95442
C −0.90190 +0.00000 +1.26666
C −1.65595 −1.24971 +0.86286
H −0.83563 +0.04626 +2.35142
H −1.42205 +0.90214 +0.93975
C −3.07780 −1.32667 +1.41258
H −1.67526 −1.33549 −0.22656
H −1.07339 −2.09737 +1.22123
C −4.18654 −0.75167 +0.54054
H −3.31893 −2.37257 +1.59789
H −3.10843 −0.84135 +2.39145
C −4.12807 +0.75149 +0.27657
H −4.20260 −1.28992 −0.40928

```
H   −5.13266   −0.98883   +1.02607
C   −3.40653   +1.15403   −1.00370
H   −5.14332   +1.14690   +0.21926
H   −3.67045   +1.24599   +1.13468
C   −4.20177   +0.84212   −2.25729
H   −3.19762   +2.22347   −0.97441
H   −2.43768   +0.65786   −1.06084
H   −3.65979   +1.13425   −3.15378
H   −4.42316   −0.21996   −2.34155
H   −5.15129   +1.37489   −2.25556
24
 * E =   +5.917  kcal/mol ; (460) 300_185_078_094_296_088
H   +0.00000   +0.00000   +0.00000
O   +0.00000   +0.00000   +0.95444
C   −0.90215   +0.00000   +1.26603
C   −1.68435   +1.21242   +0.80879
H   −1.42044   −0.91282   +0.95733
H   −0.83552   −0.02322   +2.35098
C   −3.08108   +1.26206   +1.41460
H   −1.11408   +2.10282   +1.06903
H   −1.75426   +1.19123   −0.28115
C   −3.12316   +1.73702   +2.86631
H   −3.72791   +1.89841   +0.81110
H   −3.51319   +0.26191   +1.34727
C   −3.37608   +3.22339   +3.08129
H   −3.90794   +1.19562   +3.39393
H   −2.19129   +1.46424   +3.36634
C   −2.32310   +4.19042   +2.54695
H   −4.34720   +3.48497   +2.65377
H   −3.47442   +3.37819   +4.15579
C   −2.52507   +4.62431   +1.10592
H   −2.33053   +5.08632   +3.16743
H   −1.33054   +3.75460   +2.67365
H   −1.76453   +5.34112   +0.80348
H   −2.48419   +3.79267   +0.40804
H   −3.49505   +5.10433   +0.98521
24
 * E =   +5.961  kcal/mol ; (461) 180_176_282_266_064_272
H   +0.00000   +0.00000   +0.00000
O   +0.00000   +0.00000   +0.95348
C   −0.89737   +0.00000   +1.27573
C   −0.85464   +0.00474   +2.78290
H   −1.43897   +0.87718   +0.91149
H   −1.43513   −0.88710   +0.92879
C   −2.24394   +0.09763   +3.39925
```

```
H    −0.34971    −0.90388    +3.10847
H    −0.23144    +0.83986    +3.09884
C    −2.85250    +1.49939    +3.38537
H    −2.90069    −0.58874    +2.86149
H    −2.21958    −0.27047    +4.42460
C    −2.64415    +2.33624    +4.64074
H    −2.46778    +2.05480    +2.52697
H    −3.92745    +1.41939    +3.22485
C    −1.20694    +2.70534    +4.99784
H    −3.21503    +3.25578    +4.51039
H    −3.09962    +1.82224    +5.49082
C    −0.47757    +1.68731    +5.85640
H    −0.64285    +2.89941    +4.08384
H    −1.21890    +3.65017    +5.54103
H    +0.52066    +2.03638    +6.11196
H    −1.01549    +1.52015    +6.78841
H    −0.36874    +0.72482    +5.36486
24
 * E =  +6.025  kcal/mol  ;  (462)  292_065_072_094_297_089
H    +0.00000    +0.00000    +0.00000
O    +0.00000    +0.00000    +0.95379
C    −0.89977    +0.00000    +1.27024
C    −1.63349    +1.29354    +0.97985
H    −1.44756    −0.85517    +0.86541
H    −0.83037    −0.14664    +2.34458
C    −1.83505    +1.56993    −0.50452
H    −2.59571    +1.25750    +1.49068
H    −1.06445    +2.10663    +1.42980
C    −2.86917    +0.68042    −1.19222
H    −2.10746    +2.61525    −0.64962
H    −0.87032    +1.46677    −1.00532
C    −4.28524    +1.23509    −1.27695
H    −2.54183    +0.47699    −2.21162
H    −2.90385    −0.29239    −0.69669
C    −5.01224    +1.48070    +0.04262
H    −4.26940    +2.16473    −1.85091
H    −4.86931    +0.53109    −1.86965
C    −4.79887    +2.85980    +0.64115
H    −6.08177    +1.35141    −0.12140
H    −4.73161    +0.70924    +0.76209
H    −5.36584    +2.97918    +1.56202
H    −3.75608    +3.06014    +0.87151
H    −5.13280    +3.63047    −0.05178
24
 * E =  +6.032  kcal/mol  ;  (463)  061_181_078_095_296_088
```

```
H    +0.00000   +0.00000   +0.00000
O    +0.00000   +0.00000   +0.95439
C    −0.90187   +0.00000   +1.26663
C    −1.68002   −1.21994   +0.82317
H    −0.83628   +0.01970   +2.35216
H    −1.41667   +0.91491   +0.96051
C    −3.12031   −1.19838   +1.31926
H    −1.66002   −1.27486   −0.26716
H    −1.15911   −2.10376   +1.18956
C    −4.05421   −0.29079   +0.51936
H    −3.52624   −2.20957   +1.32907
H    −3.11071   −0.88018   +2.36308
C    −4.85055   −0.95806   −0.59421
H    −4.77136   +0.16910   +1.19863
H    −3.48224   +0.53710   +0.09398
C    −4.05772   −1.55473   −1.75373
H    −5.48440   −1.73598   −0.16161
H    −5.53334   −0.20773   −0.99303
C    −3.60385   −2.98949   −1.55105
H    −4.68041   −1.52663   −2.64771
H    −3.20044   −0.91572   −1.97473
H    −3.08650   −3.36376   −2.43201
H    −2.93064   −3.10203   −0.70601
H    −4.46058   −3.63784   −1.37423
24
 * E =   +6.281  kcal/mol ;  (464) 068_297_262_060_085_299
H    +0.00000   +0.00000   +0.00000
O    +0.00000   +0.00000   +0.95458
C    −0.90128   +0.00000   +1.26909
C    −1.63335   −1.29521   +0.97308
H    −0.83340   +0.13941   +2.34490
H    −1.44111   +0.85987   +0.86834
C    −1.81050   −1.62591   −0.50838
H    −1.07238   −2.09217   +1.45888
H    −2.60957   −1.26105   +1.46165
C    −3.16799   −1.25932   −1.09904
H    −1.00487   −1.17590   −1.08756
H    −1.68153   −2.69869   −0.64494
C    −3.58781   +0.20605   −1.06107
H    −3.21110   −1.61070   −2.13231
H    −3.91566   −1.84230   −0.55889
C    −3.08919   +1.07582   −2.20610
H    −4.67698   +0.24557   −1.07949
H    −3.30072   +0.65018   −0.10742
C    −1.58320   +1.20034   −2.34338
```

```
H  −3.49801  +0.68946  −3.14134
H  −3.51459  +2.07199  −2.08360
H  −1.32329  +1.95385  −3.08343
H  −1.12098  +1.49764  −1.40198
H  −1.12970  +0.26614  −2.66582
24
∗ E =  +6.901  kcal/mol ; (465) 295_284_063_087_298_096
H  +0.00000  +0.00000  +0.00000
O  +0.00000  +0.00000  +0.95444
C  −0.90264  +0.00000  +1.26460
C  −1.67981  +1.25392  +0.91252
H  −1.42884  −0.87741  +0.88126
H  −0.83894  −0.11178  +2.34530
C  −1.35213  +2.48002  +1.75637
H  −1.55273  +1.45167  −0.15229
H  −2.73934  +1.02787  +1.04540
C  +0.06809  +3.03565  +1.67782
H  −2.06230  +3.27532  +1.52005
H  −1.55571  +2.20792  +2.79375
C  +0.35364  +4.03653  +0.56631
H  +0.27893  +3.54105  +2.62071
H  +0.77450  +2.21019  +1.61115
C  +0.19634  +3.53065  −0.86652
H  −0.28260  +4.91509  +0.70115
H  +1.37541  +4.38769  +0.70860
C  −1.15644  +3.84194  −1.48114
H  +0.96373  +3.98133  −1.49518
H  +0.39084  +2.45727  −0.89225
H  −1.25218  +3.41878  −2.47924
H  −1.97883  +3.46297  −0.87877
H  −1.29315  +4.91866  −1.56650
```

3.6 Conformers of L-serine

```
14
* E =   +0.000  kcal/mol ; (1) 145_355_293_082_092
C    +0.00000    +0.00000    +0.00000
C    +0.00000    +0.00000    +1.54639
C    +1.44870    +0.00000    -0.54091
H    -0.48357    -0.94051    -0.29633
N    -0.82791    +1.11951    -0.47285
O    -1.00284    +0.69958    +2.08974
O    +0.82725    -0.59368    +2.20513
H    -1.46184    +1.12788    +1.32643
O    +2.11206    +1.22432    -0.23071
H    +1.99276    -0.86224    -0.14205
H    +1.42811    -0.06880    -1.63294
H    +2.44283    +1.17078    +0.67687
H    -0.24480    +1.94353    -0.61400
H    -1.29553    +0.90788    -1.34827
14
* E =   +0.075  kcal/mol ; (2) 289_178_181_044_278
C    +0.00000    +0.00000    +0.00000
C    +0.00000    +0.00000    +1.52459
C    +1.43010    +0.00000    -0.52838
H    -0.47367    -0.92431    -0.34709
N    -0.70223    +1.14059    -0.57977
O    +0.40356    -1.18827    +2.03262
O    -0.29051    +0.94965    +2.22209
H    +0.41487    -1.10522    +3.00193
O    +1.42242    +0.02325    -1.93993
H    +1.96367    +0.87382    -0.11908
H    +1.95481    -0.90484    -0.21327
H    +0.75622    +0.68765    -2.18770
H    -1.70455    +0.98240    -0.61281
H    -0.55103    +1.97127    -0.01027
14
* E =   +0.235  kcal/mol ; (3) 145_356_064_305_092
C    +0.00000    +0.00000    +0.00000
C    +0.00000    +0.00000    +1.54128
C    +1.43936    +0.00000    -0.55116
H    -0.47156    -0.94481    -0.29805
N    -0.84358    +1.10602    -0.47838
O    -0.99585    +0.69672    +2.09640
O    +0.82877    -0.59695    +2.20175
H    -1.47575    +1.11528    +1.34219
O    +2.15424    -1.16837    -0.21272
H    +1.39450    +0.04127    -1.64443
```

```
H    +1.95851    +0.90899    −0.20127
H    +2.11148    −1.25421    +0.75595
H    −0.30323    +1.95604    −0.62736
H    −1.31373    +0.87995    −1.34873
14
* E =  +1.046  kcal/mol ; (4) 306_180_294_314_217
C    +0.00000    +0.00000    +0.00000
C    +0.00000    +0.00000    +1.52399
C    +1.41408    +0.00000    −0.56826
H    −0.48858    −0.94166    −0.29960
N    −0.69710    +1.19439    −0.47765
O    +0.73347    −1.01349    +2.03213
O    −0.60853    +0.78669    +2.21887
H    +0.68582    −0.94886    +3.00104
O    +2.07509    +1.21123    −0.26115
H    +2.00412    −0.81656    −0.14679
H    +1.34060    −0.14843    −1.65728
H    +1.43721    +1.91696    −0.46556
H    −1.24793    +1.00705    −1.30794
H    −1.29935    +1.57732    +0.24633
14
* E =  +1.564  kcal/mol ; (5) 194_183_296_068_308
C    +0.00000    +0.00000    +0.00000
C    +0.00000    +0.00000    +1.53161
C    +1.43377    +0.00000    −0.53865
H    −0.49949    −0.92036    −0.32817
N    −0.73212    +1.14130    −0.55120
O    −1.21627    −0.31142    +2.03830
O    +0.94389    +0.28201    +2.24859
H    −1.15909    −0.23970    +3.00709
O    +2.14122    +1.18498    −0.19679
H    +1.96301    −0.89511    −0.18411
H    +1.38807    −0.03056    −1.62992
H    +2.26944    +1.16876    +0.76447
H    −1.66303    +1.22880    −0.15573
H    −0.21355    +2.00141    −0.39061
14
* E =  +1.587  kcal/mol ; (6) 108_001_181_179_149
C    +0.00000    +0.00000    +0.00000
C    +0.00000    +0.00000    +1.54246
C    +1.46585    +0.00000    −0.48008
H    −0.46314    −0.93885    −0.32757
N    −0.70079    +1.19263    −0.50043
O    −0.38136    +1.16551    +2.08524
O    +0.34361    −0.95856    +2.19713
```

```
H   −0.59894   +1.75061   +1.31845
O   +1.44002   +0.02119   −1.90894
H   +1.98460   +0.88815   −0.08999
H   +1.96031   −0.89818   −0.09553
H   +2.34503   +0.03799   −2.24469
H   −0.29633   +1.48718   −1.38645
H   −1.68311   +0.99596   −0.67273
14
* E =  +1.606 kcal/mol ; (7) 106_003_182_281_148
C   +0.00000   +0.00000   +0.00000
C   +0.00000   +0.00000   +1.54072
C   +1.46353   +0.00000   −0.48156
H   −0.47239   −0.93997   −0.31674
N   −0.68947   +1.19381   −0.50529
O   −0.33371   +1.17926   +2.08559
O   +0.31182   −0.97095   +2.19342
H   −0.54582   +1.76985   +1.32263
O   +1.55171   +0.04695   −1.90359
H   +1.96658   +0.90476   −0.12552
H   +1.98009   −0.87044   −0.06495
H   +1.38226   −0.83326   −2.26461
H   −0.28142   +1.48829   −1.39000
H   −1.67705   +1.01841   −0.66843
14
* E =  +1.641 kcal/mol ; (8) 100_183_177_043_283
C   +0.00000   +0.00000   +0.00000
C   +0.00000   +0.00000   +1.52550
C   +1.43924   +0.00000   −0.50570
H   −0.44994   −0.94838   −0.30849
N   −0.71435   +1.08945   −0.66327
O   −0.20699   +1.23289   +2.05718
O   +0.20585   −0.97868   +2.21094
H   −0.14527   +1.14316   +3.02363
O   +1.45727   −0.07878   −1.91527
H   +1.94645   +0.91302   −0.15176
H   +1.97618   −0.86766   −0.11532
H   +0.76944   +0.53513   −2.22512
H   −1.71949   +0.94963   −0.64379
H   −0.51890   +1.98278   −0.21864
14
* E =  +1.797 kcal/mol ; (9) 145_357_175_185_091
C   +0.00000   +0.00000   +0.00000
C   +0.00000   +0.00000   +1.54284
C   +1.44099   +0.00000   −0.55128
H   −0.47026   −0.94579   −0.29853
```

```
N    -0.85808    +1.08862    -0.49169
O    -1.00674    +0.70369    +2.08051
O    +0.81354    -0.60325    +2.20683
H    -1.48475    +1.10536    +1.31542
O    +1.34100    -0.11631    -1.97193
H    +1.94694    +0.93682    -0.26936
H    +1.99459    -0.83508    -0.11008
H    +2.22613    -0.20340    -2.34677
H    -0.32088    +1.94424    -0.62153
H    -1.25412    +0.85499    -1.39678
14
* E =  +2.232  kcal/mol ; (10) 181_180_058_291_297
C    +0.00000    +0.00000    +0.00000
C    +0.00000    +0.00000    +1.53181
C    +1.43162    +0.00000    -0.54490
H    -0.48761    -0.93114    -0.31386
N    -0.74580    +1.11631    -0.58102
O    -1.25352    -0.01909    +2.04200
O    +0.98620    +0.03086    +2.24548
H    -1.17627    -0.01031    +3.01168
O    +2.19499    -1.10466    -0.09152
H    +1.37544    -0.07099    -1.63308
H    +1.92073    +0.95320    -0.29196
H    +2.34963    -0.97636    +0.85641
H    -1.72994    +1.07804    -0.33453
H    -0.37838    +2.01229    -0.27021
14
* E =  +2.510  kcal/mol ; (11) 139_358_176_272_097
C    +0.00000    +0.00000    +0.00000
C    +0.00000    +0.00000    +1.54486
C    +1.44636    +0.00000    -0.54281
H    -0.47986    -0.94406    -0.28972
N    -0.83784    +1.10350    -0.49608
O    -0.91754    +0.81120    +2.08764
O    +0.74840    -0.69035    +2.20144
H    -1.36745    +1.24327    +1.32074
O    +1.47357    -0.09831    -1.96578
H    +1.92874    +0.95253    -0.29353
H    +2.01889    -0.79842    -0.06174
H    +1.46555    -1.02910    -2.22207
H    -0.26935    +1.91410    -0.73380
H    -1.33524    +0.84178    -1.34074
14
* E =  +2.616  kcal/mol ; (12) 161_178_066_295_190
C    +0.00000    +0.00000    +0.00000
```

```
C    +0.00000    +0.00000    +1.52182
C    +1.42675    +0.00000    -0.52945
H    -0.45417    -0.96909    -0.28023
N    -0.72014    +1.16430    -0.51694
O    -1.17921    +0.39979    +2.04667
O    +0.92950    -0.35996    +2.21942
H    -1.10272    +0.34629    +3.01507
O    +2.12497    -1.19470    -0.22916
H    +1.38882    +0.07246    -1.62098
H    +1.94950    +0.88884    -0.15174
H    +2.22011    -1.23093    +0.73508
H    -0.85682    +1.07824    -1.52000
H    -1.63273    +1.26065    -0.08228
14
 * E =   +2.617  kcal/mol ; (13) 306_181_297_187_312
C    +0.00000    +0.00000    +0.00000
C    +0.00000    +0.00000    +1.53732
C    +1.43270    +0.00000    -0.55742
H    -0.45224    -0.95711    -0.29685
N    -0.76304    +1.08618    -0.58700
O    +0.73196    -1.02307    +2.04789
O    -0.61256    +0.77581    +2.23713
H    +0.66690    -0.96750    +3.01653
O    +2.04979    +1.21940    -0.13636
H    +1.98177    -0.86974    -0.17865
H    +1.36904    -0.05098    -1.65144
H    +2.91620    +1.30276    -0.55221
H    -1.67999    +1.15208    -0.15394
H    -0.28780    +1.96645    -0.40116
14
 * E =   +2.704  kcal/mol ; (14) 323_184_299_083_317
C    +0.00000    +0.00000    +0.00000
C    +0.00000    +0.00000    +1.53608
C    +1.41971    +0.00000    -0.58648
H    -0.46307    -0.95836    -0.28431
N    -0.77977    +1.08343    -0.57345
O    +0.99731    -0.76242    +2.06361
O    -0.80553    +0.57678    +2.23272
H    +0.88408    -0.75425    +3.03012
O    +2.15762    +1.16240    -0.20790
H    +1.94983    -0.91511    -0.30390
H    +1.32982    +0.03628    -1.67473
H    +2.53980    +1.01584    +0.66743
H    -1.66112    +1.19066    -0.07854
H    -0.26924    +1.95783    -0.46755
```

```
14
 * E =  +2.752  kcal/mol  ;  (15)  214_184_292_062_085
 C   +0.00000   +0.00000   +0.00000
 C   +0.00000   +0.00000   +1.52821
 C   +1.43525   +0.00000   -0.52488
 H   -0.50653   -0.91322   -0.32893
 N   -0.80050   +1.15358   -0.42387
 O   -1.02264   -0.69721   +2.05642
 O   +0.82394   +0.57591   +2.21857
 H   -0.97875   -0.59614   +3.02351
 O   +2.11062   +1.21638   -0.24207
 H   +1.98401   -0.86530   -0.12203
 H   +1.41128   -0.09310   -1.61537
 H   +2.14469   +1.30312   +0.72482
 H   -0.21131   +1.98342   -0.44690
 H   -1.17583   +1.00816   -1.35589
14
 * E =  +2.890  kcal/mol  ;  (16)  127_179_290_316_206
 C   +0.00000   +0.00000   +0.00000
 C   +0.00000   +0.00000   +1.52480
 C   +1.43632   +0.00000   -0.51186
 H   -0.45897   -0.96335   -0.27595
 N   -0.68901   +1.16069   -0.57423
 O   -0.74703   +0.98928   +2.07242
 O   +0.58130   -0.82564   +2.19331
 H   -0.68640   +0.89183   +3.03817
 O   +2.06169   +1.24101   -0.25021
 H   +2.01507   -0.77937   -0.01182
 H   +1.41520   -0.21540   -1.59240
 H   +1.40940   +1.92183   -0.48961
 H   -1.04644   +0.95031   -1.50097
 H   -1.45738   +1.48105   +0.00515
14
 * E =  +3.011  kcal/mol  ;  (17)  302_179_072_303_294
 C   +0.00000   +0.00000   +0.00000
 C   +0.00000   +0.00000   +1.53172
 C   +1.42875   +0.00000   -0.55211
 H   -0.45881   -0.94091   -0.32756
 N   -0.74374   +1.11135   -0.57019
 O   +0.67412   -1.06771   +2.05074
 O   -0.50879   +0.83979   +2.24053
 H   +0.64314   -0.98860   +3.02039
 O   +2.11566   -1.22659   -0.38213
 H   +1.34773   +0.16755   -1.62747
 H   +1.99437   +0.84117   -0.11919
```

H +2.13378 −1.44800 +0.55974
H −1.73233 +1.03323 −0.34930
H −0.43328 +1.99297 −0.16793
14
∗ E = +3.078 kcal/mol ; (18) 318_181_075_296_208
C +0.00000 +0.00000 +0.00000
C +0.00000 +0.00000 +1.52116
C +1.39903 +0.00000 −0.59721
H −0.46599 −0.96048 −0.28618
N −0.72699 +1.17487 −0.46399
O +0.93936 −0.83420 +2.04481
O −0.75336 +0.63822 +2.22299
H +0.85761 −0.79130 +3.01322
O +2.06062 −1.25067 −0.51205
H +1.29753 +0.21905 −1.66331
H +1.98792 +0.81196 −0.14860
H +2.19912 −1.45735 +0.42254
H −1.15265 +1.01383 −1.37025
H −1.44713 +1.44560 +0.19959
14
∗ E = +3.236 kcal/mol ; (19) 316_006_065_189_224
C +0.00000 +0.00000 +0.00000
C +0.00000 +0.00000 +1.54277
C +1.42336 +0.00000 −0.59514
H −0.47373 −0.95245 −0.29256
N −0.71616 +1.16764 −0.49361
O +0.86078 −0.84403 +2.13448
O −0.75132 +0.69483 +2.19062
H +1.43377 −1.26490 +1.45570
O +2.06467 −1.24023 −0.23050
H +1.34898 +0.08146 −1.68476
H +1.98511 +0.86156 −0.21500
H +2.99952 −1.21374 −0.47056
H −1.34053 +0.93498 −1.25755
H −1.26147 +1.57477 +0.26329
14
∗ E = +3.350 kcal/mol ; (20) 043_177_288_066_310
C +0.00000 +0.00000 +0.00000
C +0.00000 +0.00000 +1.53256
C +1.40579 +0.00000 −0.61032
H −0.49170 −0.93791 −0.27994
N −0.76841 +1.10969 −0.55842
O +0.92119 +0.85338 +2.07453
O −0.75663 −0.64043 +2.22968
H +0.81537 +0.81754 +3.04120

O +2.08474 +1.24124 −0.46896
H +1.99435 −0.82860 −0.19077
H +1.29954 −0.16186 −1.68579
H +2.25333 +1.38736 +0.47286
H −1.69434 +1.18619 −0.14943
H −0.26965 +1.98833 −0.44933
14
* E = +3.422 kcal/mol ; (21) 126_179_292_181_309
C +0.00000 +0.00000 +0.00000
C +0.00000 +0.00000 +1.53942
C +1.45719 +0.00000 −0.49640
H −0.42485 −0.97473 −0.27589
N −0.75190 +1.04597 −0.67167
O −0.73698 +0.99797 +2.08069
O +0.57089 −0.83021 +2.21596
H −0.67664 +0.90049 +3.04654
O +2.01597 +1.27169 −0.15305
H +2.00723 −0.81621 −0.01466
H +1.44913 −0.14354 −1.58394
H +2.93000 +1.31449 −0.45827
H −1.70232 +1.10247 −0.31973
H −0.31013 +1.94707 −0.50981
14
* E = +3.433 kcal/mol ; (22) 144_358_170_077_090
C +0.00000 +0.00000 +0.00000
C +0.00000 +0.00000 +1.54417
C +1.44343 +0.00000 −0.54860
H −0.46427 −0.94787 −0.29908
N −0.86389 +1.08451 −0.49346
O −0.98812 +0.72510 +2.09206
O +0.80384 −0.61987 +2.20321
H −1.47357 +1.12862 +1.33555
O +1.48004 −0.22420 −1.95469
H +1.94587 +0.94262 −0.28263
H +2.00018 −0.81519 −0.08529
H +1.22889 +0.58456 −2.41822
H −0.35657 +1.95592 −0.63184
H −1.33754 +0.84399 −1.35734
14
* E = +3.605 kcal/mol ; (23) 027_003_298_308_201
C +0.00000 +0.00000 +0.00000
C +0.00000 +0.00000 +1.54535
C +1.41294 +0.00000 −0.62586
H −0.52442 −0.92103 −0.28957
N −0.68504 +1.22754 −0.45332

O +1.06667 +0.53438 +2.16147
O −0.93810 −0.44462 +2.16900
H +1.69013 +0.90284 +1.49355
O +2.12834 +1.18257 −0.24799
H +2.00436 −0.85062 −0.27662
H +1.33447 −0.06545 −1.71977
H +1.54339 +1.92867 −0.47072
H −1.00945 +1.11693 −1.41164
H −1.50599 +1.41610 +0.11685
14
* E = +3.615 kcal/mol ; (24) 282_177_182_283_039
C +0.00000 +0.00000 +0.00000
C +0.00000 +0.00000 +1.51816
C +1.43875 +0.00000 −0.48456
H −0.45848 −0.94304 −0.32881
N −0.79127 +1.11745 −0.48801
O +0.25503 −1.23015 +2.02619
O −0.15675 +0.97949 +2.21525
H +0.28582 −1.14304 +2.99455
O +1.49506 +0.05051 −1.90740
H +1.94103 +0.90745 −0.13528
H +1.97586 −0.87482 −0.09512
H +1.28631 −0.82273 −2.26525
H −0.63991 +1.93792 +0.09406
H −0.52387 +1.33621 −1.44397
14
* E = +3.746 kcal/mol ; (25) 261_177_051_066_287
C +0.00000 +0.00000 +0.00000
C +0.00000 +0.00000 +1.52941
C +1.44325 +0.00000 −0.50609
H −0.49758 −0.91341 −0.34390
N −0.68391 +1.15157 −0.57700
O −0.19483 −1.23257 +2.05237
O +0.15483 +0.99037 +2.21337
H −0.12799 −1.15339 +3.02015
O +2.25250 −1.01478 +0.07192
H +1.41294 −0.06472 −1.60072
H +1.91526 +0.94843 −0.23240
H +1.93648 −1.88188 −0.21424
H −1.69056 +1.07735 −0.46239
H −0.38798 +2.00327 −0.10415
14
* E = +3.761 kcal/mol ; (26) 280_177_179_174_045
C +0.00000 +0.00000 +0.00000
C +0.00000 +0.00000 +1.51988

C +1.44164 +0.00000 −0.48138
H −0.44972 −0.93983 −0.34131
N −0.80207 +1.11361 −0.47895
O +0.22229 −1.23452 +2.03108
O −0.13644 +0.98377 +2.21518
H +0.24817 −1.14603 +2.99949
O +1.38538 −0.03079 −1.90779
H +1.94556 +0.91150 −0.12808
H +1.96991 −0.88021 −0.09035
H +2.27608 +0.06793 −2.26591
H −0.58379 +1.96079 +0.03985
H −0.61846 +1.26835 −1.46596
14
* E = +3.778 kcal/mol ; (27) 169_180_175_172_178
C +0.00000 +0.00000 +0.00000
C +0.00000 +0.00000 +1.52185
C +1.42507 +0.00000 −0.53403
H −0.46305 −0.95999 −0.29855
N −0.72015 +1.16868 −0.50854
O −1.23838 +0.23712 +2.01910
O +0.95650 −0.22036 +2.23382
H −1.16981 +0.21621 +2.98880
O +1.31783 −0.12784 −1.95650
H +1.91496 +0.94204 −0.25814
H +1.98502 −0.83619 −0.10136
H +2.18892 −0.00407 −2.35208
H −0.68128 +1.15903 −1.52438
H −1.69415 +1.15135 −0.22157
14
* E = +3.835 kcal/mol ; (28) 321_184_174_168_192
C +0.00000 +0.00000 +0.00000
C +0.00000 +0.00000 +1.52157
C +1.39689 +0.00000 −0.60322
H −0.45862 −0.96989 −0.27719
N −0.74434 +1.15612 −0.47513
O +0.97732 −0.77838 +2.04871
O −0.81305 +0.57785 +2.20969
H +0.86302 −0.76191 +3.01446
O +1.21549 −0.14720 −2.01553
H +1.88767 +0.95328 −0.36800
H +1.98729 −0.82895 −0.19808
H +2.04889 +0.03917 −2.46437
H −0.92432 +1.06856 −1.46967
H −1.62087 +1.25929 +0.02691
14

```
 * E =   +3.910  kcal/mol  ;  (29)  318_007_061_083_221
 C    +0.00000    +0.00000    +0.00000
 C    +0.00000    +0.00000    +1.54302
 C    +1.42469    +0.00000    −0.59259
 H    −0.48358    −0.95271    −0.28557
 N    −0.71738    +1.16799    −0.48812
 O    +0.90111    −0.79839    +2.13720
 O    −0.78943    +0.65412    +2.18760
 H    +1.50578    −1.18437    +1.46419
 O    +2.17944    −1.16628    −0.20332
 H    +1.36590    +0.07591    −1.68305
 H    +1.98072    +0.86520    −0.22423
 H    +1.96525    −1.90710    −0.78542
 H    −1.30184    +0.95199    −1.28772
 H    −1.30131    +1.54468    +0.25560
14
 * E =   +3.925  kcal/mol  ;  (30)  245_179_058_190_286
 C    +0.00000    +0.00000    +0.00000
 C    +0.00000    +0.00000    +1.53134
 C    +1.44471    +0.00000    −0.50775
 H    −0.49644    −0.90775    −0.35255
 N    −0.68874    +1.16535    −0.55363
 O    −0.52215    −1.12607    +2.06345
 O    +0.39297    +0.92899    +2.20884
 H    −0.47182    −1.04084    +3.03172
 O    +2.09867    −1.14937    +0.02705
 H    +1.41759    −0.01965    −1.60406
 H    +1.93769    +0.92589    −0.17975
 H    +3.04457    −1.09167    −0.15349
 H    −1.69521    +1.08348    −0.44515
 H    −0.39319    +2.01168    −0.07157
14
 * E =   +3.961  kcal/mol  ;  (31)  125_182_067_309_058
 C    +0.00000    +0.00000    +0.00000
 C    +0.00000    +0.00000    +1.52037
 C    +1.44594    +0.00000    −0.46986
 H    −0.42375    −0.96819    −0.29714
 N    −0.83228    +1.05675    −0.55499
 O    −0.69784    +1.00098    +2.08769
 O    +0.62703    −0.81077    +2.18295
 H    −0.59224    +0.91082    +3.05108
 O    +2.14046    −1.19229    −0.16583
 H    +1.44048    +0.08990    −1.56082
 H    +1.96076    +0.88738    −0.06414
 H    +2.01628    −1.37517    +0.78026
```

```
H   −0.54978   +1.97946   −0.23779
H   −0.80775   +1.04054   −1.57003
14
* E =  +4.088 kcal/mol ; (32) 293_177_074_306_041
C   +0.00000   +0.00000   +0.00000
C   +0.00000   +0.00000   +1.51824
C   +1.43418   +0.00000   −0.49816
H   −0.45021   −0.94245   −0.33745
N   −0.80038   +1.12223   −0.45981
O   +0.48707   −1.16904   +2.02389
O   −0.32231   +0.92538   +2.22874
H   +0.49932   −1.08080   +2.99298
O   +2.09755   −1.24296   −0.35148
H   +1.40756   +0.19911   −1.57382
H   +1.99108   +0.82121   −0.01934
H   +2.04035   −1.52319   +0.57331
H   −0.63944   +1.95333   +0.10148
H   −0.63470   +1.33087   −1.43860
14
* E =  +4.375 kcal/mol ; (33) 061_178_285_062_085
C   +0.00000   +0.00000   +0.00000
C   +0.00000   +0.00000   +1.52608
C   +1.42293   +0.00000   −0.55153
H   −0.47791   −0.94301   −0.27976
N   −0.83265   +1.10830   −0.47054
O   +0.61611   +1.10832   +2.03443
O   −0.45454   −0.86505   +2.23858
H   +0.55475   +1.05522   +3.00394
O   +2.06398   +1.26311   −0.43808
H   +2.01485   −0.79609   −0.07436
H   +1.37603   −0.21310   −1.62400
H   +2.13646   +1.48881   +0.50096
H   −0.28160   +1.96283   −0.51041
H   −1.17886   +0.91870   −1.40646
14
* E =  +4.387 kcal/mol ; (34) 067_181_048_066_293
C   +0.00000   +0.00000   +0.00000
C   +0.00000   +0.00000   +1.52945
C   +1.43077   +0.00000   −0.54047
H   −0.49393   −0.93309   −0.29072
N   −0.73108   +1.11051   −0.60790
O   +0.48699   +1.16144   +2.03683
O   −0.40216   −0.90325   +2.23106
H   +0.46933   +1.08567   +3.00571
O   +2.26020   −0.97375   +0.08038
```

```
H    +1.38212    −0.12854    −1.62911
H    +1.90073    +0.96540    −0.33142
H    +1.94997    −1.85883    −0.15267
H    −1.72843    +1.04443    −0.42837
H    −0.40544    +2.00377    −0.24767
14
* E =   +4.523  kcal/mol ; (35) 167_179_177_278_175
C    +0.00000    +0.00000    +0.00000
C    +0.00000    +0.00000    +1.52325
C    +1.42425    +0.00000    −0.54018
H    −0.47015    −0.96391    −0.28546
N    −0.71471    +1.16493    −0.51474
O    −1.22374    +0.29311    +2.02565
O    +0.94726    −0.27118    +2.23027
H    −1.15337    +0.26496    +2.99523
O    +1.42127    −0.07559    −1.96854
H    +1.90575    +0.94743    −0.28638
H    +2.00091    −0.81533    −0.09273
H    +1.31650    −0.99734    −2.23711
H    −0.63440    +1.18391    −1.52803
H    −1.69730    +1.14581    −0.26074
14
* E =   +4.527  kcal/mol ; (36) 318_183_176_278_187
C    +0.00000    +0.00000    +0.00000
C    +0.00000    +0.00000    +1.52275
C    +1.39873    +0.00000    −0.60193
H    −0.46616    −0.97179    −0.26808
N    −0.73272    +1.15783    −0.47841
O    +0.93071    −0.84201    +2.03958
O    −0.77058    +0.62438    +2.21783
H    +0.82803    −0.82105    +3.00650
O    +1.33131    −0.08314    −2.02707
H    +1.88181    +0.95479    −0.37855
H    +1.99903    −0.81408    −0.18363
H    +1.21591    −1.00721    −2.28368
H    −0.85013    +1.11230    −1.48495
H    −1.63462    +1.25046    −0.02263
14
* E =   +4.593  kcal/mol ; (37) 093_182_177_280_053
C    +0.00000    +0.00000    +0.00000
C    +0.00000    +0.00000    +1.51920
C    +1.44140    +0.00000    −0.47993
H    −0.44392    −0.96016    −0.28921
N    −0.81913    +1.07236    −0.55235
O    −0.05689    +1.24875    +2.04340
```

O +0.10758 −0.99326 +2.20674
H −0.02258 +1.15844 +3.01102
O +1.50063 −0.06645 −1.90214
H +1.92043 +0.94471 −0.20377
H +1.99774 −0.82571 −0.01716
H +1.36437 −0.98086 −2.18325
H −0.54694 +1.97193 −0.16386
H −0.67606 +1.11213 −1.55879
14
* E = +4.708 kcal/mol ; (38) 041_354_297_180_305
C +0.00000 +0.00000 +0.00000
C +0.00000 +0.00000 +1.54825
C +1.41299 +0.00000 −0.63288
H −0.48541 −0.93817 −0.28535
N −0.78792 +1.10035 −0.56352
O +0.91363 +0.78086 +2.16574
O −0.82244 −0.62018 +2.18505
H +1.51852 +1.17386 +1.50209
O +2.07120 +1.22553 −0.24739
H +1.98862 −0.86581 −0.28134
H +1.30883 −0.04245 −1.72231
H +2.96084 +1.25972 −0.61999
H −1.73561 +1.09059 −0.19693
H −0.37116 +2.00221 −0.34786
14
* E = +4.772 kcal/mol ; (39) 055_178_055_186_292
C +0.00000 +0.00000 +0.00000
C +0.00000 +0.00000 +1.53004
C +1.41744 +0.00000 −0.57610
H −0.50078 −0.92479 −0.29746
N −0.74305 +1.13108 −0.56192
O +0.72698 +1.03143 +2.03962
O −0.62065 −0.76681 +2.23349
H +0.64910 +0.98610 +3.00741
O +2.10797 −1.11809 −0.02223
H +1.33682 −0.07129 −1.66802
H +1.91989 +0.94304 −0.31990
H +3.03363 −1.08925 −0.29232
H −1.73752 +1.04951 −0.37364
H −0.41925 +2.01449 −0.17641
14
* E = +4.803 kcal/mol ; (40) 103_359_300_301_166
C +0.00000 +0.00000 +0.00000
C +0.00000 +0.00000 +1.53980
C +1.44921 +0.00000 −0.52036

```
H   -0.45609   -0.95096   -0.30709
N   -0.71251   +1.18715   -0.50394
O   -0.27745   +1.18556   +2.11128
O   +0.23920   -0.99401   +2.18402
H   -0.40551   +1.83147   +1.38616
O   +2.20225   +1.12670   -0.10077
H   +1.96488   -0.87820   -0.12684
H   +1.42773   -0.07791   -1.61972
H   +1.76931   +1.93459   -0.40788
H   -0.53220   +1.33244   -1.49505
H   -1.71993   +1.08498   -0.40409
14
* E =   +4.966  kcal/mol ; (41) 260_177_057_062_051
C   +0.00000   +0.00000   +0.00000
C   +0.00000   +0.00000   +1.52213
C   +1.43832   +0.00000   -0.49812
H   -0.49853   -0.91368   -0.34443
N   -0.77204   +1.15564   -0.44772
O   -0.22203   -1.22422   +2.04547
O   +0.19905   +0.98632   +2.19934
H   -0.15238   -1.14351   +3.01285
O   +2.21810   -1.08765   -0.02061
H   +1.41678   +0.02742   -1.59856
H   +1.93784   +0.90683   -0.14456
H   +1.84065   -1.91973   -0.33579
H   -0.44106   +2.00102   +0.01151
H   -0.70419   +1.27964   -1.45374
14
* E =   +5.122  kcal/mol ; (42) 242_180_064_193_060
C   +0.00000   +0.00000   +0.00000
C   +0.00000   +0.00000   +1.52616
C   +1.43925   +0.00000   -0.50766
H   -0.50112   -0.90626   -0.35054
N   -0.77882   +1.16427   -0.42288
O   -0.58385   -1.09059   +2.05900
O   +0.46491   +0.89950   +2.19655
H   -0.53836   -0.99983   +3.02688
O   +2.06061   -1.21269   -0.08661
H   +1.41989   +0.07434   -1.60542
H   +1.95834   +0.87952   -0.10275
H   +3.01486   -1.14844   -0.21496
H   -0.35935   +2.02456   -0.07866
H   -0.84976   +1.21424   -1.43474
14
* E =   +5.132  kcal/mol ; (43) 294_178_299_179_048
```

C +0.00000 +0.00000 +0.00000
C +0.00000 +0.00000 +1.52555
C +1.43685 +0.00000 −0.51261
H −0.43731 −0.95673 −0.31708
N −0.84249 +1.08894 −0.46697
O +0.51076 −1.15724 +2.02072
O −0.38466 +0.90153 +2.23337
H +0.51026 −1.07614 +2.98948
O +2.07594 +1.18539 −0.04765
H +1.95342 −0.90074 −0.15480
H +1.40710 −0.02700 −1.61260
H +2.99337 +1.19517 −0.34609
H −0.60976 +1.94763 +0.02482
H −0.71302 +1.24476 −1.46224
14
∗ E = +5.316 kcal/mol ; (44) 115_182_292_173_065
C +0.00000 +0.00000 +0.00000
C +0.00000 +0.00000 +1.52880
C +1.45265 +0.00000 −0.47649
H −0.42152 −0.97231 −0.28318
N −0.84568 +1.04782 −0.55468
O −0.51806 +1.11440 +2.08070
O +0.45023 −0.91427 +2.19000
H −0.43423 +1.01642 +3.04460
O +2.02384 +1.25880 −0.12095
H +1.99079 −0.83219 −0.00516
H +1.46159 −0.13938 −1.56793
H +2.96935 +1.24898 −0.31197
H −0.47913 +1.96475 −0.31300
H −0.86787 +0.98006 −1.56869
14
∗ E = +5.419 kcal/mol ; (45) 089_182_176_079_064
C +0.00000 +0.00000 +0.00000
C +0.00000 +0.00000 +1.52076
C +1.44480 +0.00000 −0.47461
H −0.44329 −0.95203 −0.30513
N −0.83439 +1.07446 −0.52531
O +0.02843 +1.25483 +2.03650
O +0.04024 −0.99264 +2.21494
H +0.05410 +1.16898 +3.00472
O +1.52837 −0.09020 −1.89140
H +1.95829 +0.89960 −0.10543
H +1.95995 −0.88048 −0.08012
H +1.35103 +0.77880 −2.27321
H −0.48082 +1.99687 −0.28771

```
H    −0.94840    +0.99455    −1.53034
14
 ∗ E =   +5.698  kcal/mol ; (46) 072_181_051_060_065
C    +0.00000    +0.00000    +0.00000
C    +0.00000    +0.00000    +1.52338
C    +1.42770    +0.00000    −0.53137
H    −0.49283    −0.93415    −0.28983
N    −0.81341    +1.11280    −0.49119
O    +0.38609    +1.20052    +2.02259
O    −0.28631    −0.94395    +2.22468
H    +0.37564    +1.12997    +2.99204
O    +2.25219    −1.01039    +0.03256
H    +1.38304    −0.07941    −1.62883
H    +1.90786    +0.95035    −0.28022
H    +1.87674    −1.88100    −0.15630
H    −0.40045    +2.00817    −0.24324
H    −0.91748    +1.07252    −1.50101
14
 ∗ E =   +5.920  kcal/mol ; (47) 285_356_180_046_278
C    +0.00000    +0.00000    +0.00000
C    +0.00000    +0.00000    +1.53524
C    +1.43919    +0.00000    −0.53451
H    −0.48725    −0.91279    −0.36657
N    −0.68972    +1.14832    −0.57753
O    +0.32464    −1.17440    +2.13804
O    −0.22933    +0.98023    +2.20174
H    +0.45318    −1.88090    +1.48753
O    +1.43191    −0.00951    −1.94531
H    +1.96248    +0.88519    −0.13959
H    +1.98767    −0.88924    −0.20808
H    +0.77771    +0.66078    −2.20870
H    −1.69472    +1.00362    −0.59931
H    −0.52332    +1.97064    +0.00111
14
 ∗ E =   +6.304  kcal/mol ; (48) 062_178_058_187_070
C    +0.00000    +0.00000    +0.00000
C    +0.00000    +0.00000    +1.52641
C    +1.42046    +0.00000    −0.55878
H    −0.49765    −0.92716    −0.29491
N    −0.81266    +1.12994    −0.45549
O    +0.59188    +1.12179    +2.01959
O    −0.47329    −0.85506    +2.23714
H    +0.53236    +1.07506    +2.98859
O    +2.09658    −1.15182    −0.06292
H    +1.35496    −0.01801    −1.65740
```

```
 H    +1.92924   +0.92553   -0.25489
 H    +3.02813   -1.11311   -0.31224
 H    -0.36097   +2.01864   -0.25692
 H    -0.99930   +1.07241   -1.45197
14
 * E =  +7.417 kcal/mol ; (49) 053_181_057_065_180
 C    +0.00000   +0.00000   +0.00000
 C    +0.00000   +0.00000   +1.52890
 C    +1.42441   +0.00000   -0.55551
 H    -0.49094   -0.94674   -0.27740
 N    -0.70157   +1.19041   -0.49137
 O    +0.73727   +0.99606   +2.06009
 O    -0.60832   -0.80430   +2.20483
 H    +0.68439   +0.90855   +3.02683
 O    +2.19255   -1.10144   -0.09007
 H    +1.37316   +0.01509   -1.65544
 H    +1.94323   +0.90125   -0.22491
 H    +1.81355   -1.92504   -0.42478
 H    -0.71235   +1.20016   -1.50820
 H    -1.66864   +1.20343   -0.17850
14
 * E =  +7.637 kcal/mol ; (50) 190_183_073_072_183
 C    +0.00000   +0.00000   +0.00000
 C    +0.00000   +0.00000   +1.52888
 C    +1.42417   +0.00000   -0.55607
 H    -0.50099   -0.93302   -0.31556
 N    -0.70752   +1.21190   -0.43638
 O    -1.25496   -0.23100   +2.00355
 O    +0.94452   +0.19978   +2.25427
 H    -1.21038   -0.18074   +2.97377
 O    +2.07224   -1.25599   -0.39447
 H    +1.38842   +0.29570   -1.61515
 H    +2.02842   +0.73116   -0.01693
 H    +1.68598   -1.89581   -1.00641
 H    -0.77908   +1.23798   -1.45005
 H    -1.65069   +1.23698   -0.05889
14
 * E =  +7.648 kcal/mol ; (51) 051_351_292_173_082
 C    +0.00000   +0.00000   +0.00000
 C    +0.00000   +0.00000   +1.54524
 C    +1.42877   +0.00000   -0.58854
 H    -0.47945   -0.94164   -0.27874
 N    -0.84615   +1.09893   -0.47025
 O    +0.76835   +0.94080   +2.14101
 O    -0.65268   -0.77821   +2.19837
```

```
H   +1.32259   +1.39031   +1.47233
O   +2.02541   +1.27340   −0.27155
H   +2.02293   −0.81943   −0.16211
H   +1.37469   −0.12524   −1.67769
H   +2.95728   +1.27893   −0.52244
H   −0.34571   +1.98323   −0.47128
H   −1.18692   +0.92413   −1.41054
14
 * E =  +7.784 kcal/mol ; (52) 208_007_294_063_311
C   +0.00000   +0.00000   +0.00000
C   +0.00000   +0.00000   +1.54029
C   +1.44552   +0.00000   −0.53194
H   −0.48846   −0.91916   −0.35668
N   −0.71339   +1.15074   −0.55664
O   −1.07193   −0.57455   +2.14757
O   +0.85670   +0.52237   +2.22134
H   −1.64778   −1.00607   +1.49911
O   +2.13420   +1.19878   −0.21700
H   +1.97763   −0.88215   −0.14825
H   +1.40866   −0.06195   −1.62258
H   +2.18497   +1.24887   +0.75198
H   −1.62776   +1.29734   −0.13941
H   −0.15529   +1.99214   −0.42982
14
 * E =  +8.295 kcal/mol ; (53) 154_353_077_070_083
C   +0.00000   +0.00000   +0.00000
C   +0.00000   +0.00000   +1.54689
C   +1.42510   +0.00000   −0.60163
H   −0.49178   −0.93860   −0.29122
N   −0.86667   +1.09652   −0.46619
O   −1.11079   +0.54562   +2.08281
O   +0.88716   −0.46064   +2.22228
H   −1.62022   +0.92356   +1.33221
O   +2.07340   −1.25932   −0.55403
H   +1.35209   +0.36830   −1.63674
H   +2.06696   +0.68590   −0.04116
H   +1.64830   −1.86362   −1.17657
H   −0.37282   +1.98684   −0.48104
H   −1.22760   +0.92757   −1.40001
14
 * E =  +9.316 kcal/mol ; (54) 310_008_300_196_312
C   +0.00000   +0.00000   +0.00000
C   +0.00000   +0.00000   +1.55120
C   +1.42610   +0.00000   −0.61030
H   −0.47083   −0.95025   −0.29611
```

N −0.76907 +1.09004 −0.57353
O +0.77874 −0.92909 +2.16833
O −0.70443 +0.72442 +2.20884
H +1.33517 −1.40018 +1.53173
O +2.09641 +1.16857 −0.14883
H +1.98441 −0.90806 −0.32739
H +1.31979 +0.00053 −1.70154
H +2.87405 +1.33892 −0.69408
H −1.67990 +1.14347 −0.12516
H −0.29934 +1.96918 −0.36670
14
* E = +9.324 kcal/mol ; (55) 267_352_177_170_045
C +0.00000 +0.00000 +0.00000
C +0.00000 +0.00000 +1.53143
C +1.45515 +0.00000 −0.47729
H −0.46075 −0.92501 −0.37551
N −0.79245 +1.12545 −0.46778
O −0.05632 −1.21080 +2.14259
O +0.10228 +1.00869 +2.18617
H −0.21437 −1.91524 +1.49702
O +1.41061 −0.06151 −1.90227
H +1.94450 +0.92028 −0.13120
H +1.99680 −0.86580 −0.06673
H +2.29309 +0.09724 −2.25946
H −0.55391 +1.96122 +0.06166
H −0.60792 +1.29097 −1.45330
14
* E = +9.390 kcal/mol ; (56) 039_353_177_041_290
C +0.00000 +0.00000 +0.00000
C +0.00000 +0.00000 +1.53754
C +1.38905 +0.00000 −0.65922
H −0.51172 −0.92042 −0.29223
N −0.77036 +1.12014 −0.56538
O +0.94577 +0.75514 +2.16552
O −0.83045 −0.58313 +2.19077
H +1.58348 +1.12277 +1.53498
O +1.27086 −0.06451 −2.06323
H +1.95293 +0.90950 −0.36901
H +1.97074 −0.86856 −0.33549
H +0.53569 +0.52732 −2.29895
H −1.76657 +0.99980 −0.40761
H −0.49886 +2.01631 −0.16610
14
* E = +9.906 kcal/mol ; (57) 271_351_184_282_038
C +0.00000 +0.00000 +0.00000

C +0.00000 +0.00000 +1.53051
C +1.45335 +0.00000 −0.47984
H −0.47223 −0.92833 −0.36228
N −0.77661 +1.13142 −0.47700
O +0.01768 −1.21374 +2.14013
O +0.04955 +1.01108 +2.18724
H −0.11603 −1.92569 +1.49753
O +1.52394 +0.08338 −1.89969
H +1.95325 +0.89528 −0.09976
H +1.99188 −0.88362 −0.10705
H +1.33139 −0.78154 −2.28525
H −0.60813 +1.93815 +0.12051
H −0.49996 +1.36345 −1.42758
14
* E = +10.304 kcal/mol ; (58) 308_001_172_156_200
C +0.00000 +0.00000 +0.00000
C +0.00000 +0.00000 +1.53389
C +1.41029 +0.00000 −0.60325
H −0.46849 −0.96018 −0.29419
N −0.71828 +1.16666 −0.47843
O +0.75181 −0.95760 +2.13971
O −0.65824 +0.75810 +2.20141
H +1.20835 −1.51255 +1.49039
O +1.25059 −0.18883 −2.00755
H +1.88660 +0.96150 −0.37765
H +2.03197 −0.81131 −0.19096
H +2.01966 +0.16483 −2.47049
H −1.01345 +1.04171 −1.43995
H −1.51455 +1.37826 +0.11452
14
* E = +11.658 kcal/mol ; (59) 313_004_177_284_192
C +0.00000 +0.00000 +0.00000
C +0.00000 +0.00000 +1.53652
C +1.40262 +0.00000 −0.62734
H −0.48106 −0.96424 −0.27565
N −0.71708 +1.16837 −0.46656
O +0.82395 −0.89238 +2.14887
O −0.72722 +0.69635 +2.19870
H +1.36057 −1.38024 +1.50758
O +1.33027 −0.06781 −2.04743
H +1.89585 +0.94673 −0.39460
H +2.02536 −0.82377 −0.24272
H +1.10454 −0.96889 −2.31493
H −0.91684 +1.11026 −1.45845
H −1.56546 +1.32789 +0.06636

```
14
 * E = +12.036 kcal/mol ; (60) 290_355_300_182_037
 C    +0.00000    +0.00000    +0.00000
 C    +0.00000    +0.00000    +1.53795
 C    +1.44330    +0.00000    -0.53121
 H    -0.44445    -0.95314    -0.33203
 N    -0.83203    +1.09706    -0.45973
 O    +0.41441    -1.15338    +2.13023
 O    -0.32226    +0.94405    +2.21137
 H    +0.58425    -1.84434    +1.47357
 O    +2.09949    +1.16577    -0.05935
 H    +1.96687    -0.91335    -0.20419
 H    +1.39974    -0.01888    -1.63115
 H    +2.99828    +1.19856    -0.40911
 H    -0.71475    +1.89668    +0.15778
 H    -0.58795    +1.37777    -1.40439
```
